# Synthesis of aryl-fused bicyclo[3.1.1]heptanes and validation as naphthyl bioisosteres

**DOI:** 10.1038/s41557-026-02129-2

**Published:** 2026-05-06

**Authors:** Aidan Kerckhoffs, Maud Tregear, Pol Hernández-Lladó, Massimiliano Runfola, Holly Shearsmith, Nils Frank, Marc Panosetti, Sarah E. Squire, Lee Moir, Kirsten E. Christensen, Fernanda Duarte, Kay E. Davies, Angela J. Russell

**Affiliations:** 1 Department of Chemistry, Chemistry Research Laboratory, University of Oxford, Oxford, UK; 2 Department of Pharmacology, University of Oxford, Oxford, UK; 3 Department of Physiology, Anatomy and Genetics, University of Oxford, Oxford, UK

**Keywords:** Drug discovery and development, Synthetic chemistry methodology

## Abstract

Although naphthalene motifs are frequently encountered in drugs and lead compounds, their flat, *sp*^2^-rich nature and susceptibility to cytochrome P450-mediated metabolism often limit their developability. Here we report the study of derivatizable aryl-fused bicyclo[3.1.1]heptanes (BCHeps) as *sp*^3^-rich bioisosteric replacements for naphthalene and other fused bicyclic (hetero)aromatics, including underrepresented β-naphthyl units. The BCHeps were efficiently accessed via an intramolecular crossed [2+2] photocycloaddition enabled by visible light energy transfer and subsequently diversified to provide a range of different scaffolds. Here we show that the incorporation of BCHep-based naphthyl isosteres into the AhR antagonist ezutromid preserves key geometric exit vectors while reducing the fraction of *sp*^2^ carbon atoms. Importantly, these analogues retain biological activity and display improved metabolic stability towards CYP1A-mediated metabolism. Solid-state structures, cellular assays and microsomal studies confirm that BCHep substitution mitigates reactive metabolite formation, validating aryl-fused BCHeps as true bioisosteric replacements for *meta*-substituted arenes and 2-naphthalenes.

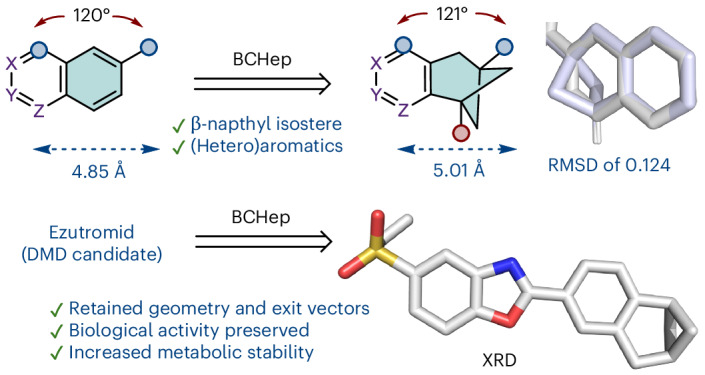

## Abstract (web summary)

Naphthalene rings are common in drug candidates but suffer from metabolic liabilities and limited three-dimensionality. Aryl-fused bicyclo[3.1.1]heptanes (BCHeps) have been presented as derivatizable bioisosteres of β-naphthalene. Now it has been shown that aryl-fused BCHeps can be synthesized and validated as viable naphthyl replacements with promising structural and drug-like properties.

## Main

The naphthalene ring is a frequently encountered structural motif within lead compounds and commercially available drugs and drug candidates, including propranolol, naproxen, ezutromid and terbinafine^1–3^. However, naphthyl groups are susceptible to metabolic degradation, often proceeding via a 1,2-naphthalene oxide intermediate^4^, which is transformed into glutathione adducts^5^, hydroxynaphthalene derivatives^6^ or *trans*-1,2-dihydro-1,2-dihydroxy naphthyl products^7^. The diols can be further oxidized to the highly electrophilic 1,4-naphthoquinone and 1,2-naphthoquinones, which covalently bind proteins and can lead to toxic side effects^8^. These oxidative pathways have been observed for both α-naphthyl^9^ and β-naphthyl derivatives^10^ and usually originate through oxidation by multiple cytochrome P450 (CYP) enzymes, including subfamilies CYP1A1 and CYP1B1^11^. Furthermore, the *sp*^2^-rich naphthyl group exhibits a ‘flat’ structure lattice, resulting in higher melting points and poor solubility, limiting its utility as a structural motif within pharmaceuticals^12^.

**Fig. 1:**
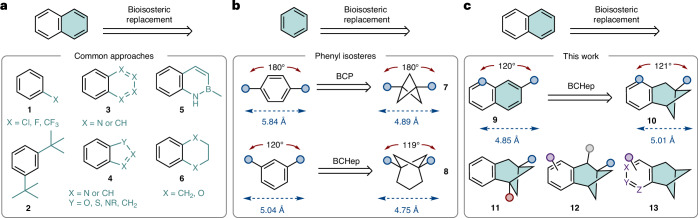
Bioisosteric replacements for aromatic ring systems. **a**, The representative bioisosteric replacement strategies for naphthyl rings, where substitution typically involves replacement of one benzene ring with alternative functional groups or ring systems. **b**, The representative bioisosteric replacements for phenyl rings, including the BCP motif and the BCHep motif. **c**, This work: development of BCHep naphthyl bioisosteres.

Consequently, the bioisosteric replacement of naphthalene is frequently undertaken in drug discovery programmes, where typically one of the benzene rings is exchanged for an alternative functionality (Fig. 1a). For example, Bristol Myers Squibb replaced the naphthalene ring with a trifluoromethyl-containing benzene during the development of BMS-641988 for the treatment of prostate cancer^13^. Vertex’s early stage naphthyl compounds were replaced with a 1,3-di-*tert*-butylbenzene unit **2** towards the development of the FDA-approved cystic fibrosis drug Ivacaftor^14^. Similarly, previous efforts in our group during the development of second generation utrophin modulators for the treatment of Duchenne muscular dystrophy (DMD) involved the replacement of a 2-naphthyl group with halo-phenyl or trifluoromethyl-phenyl derivatives. These replacements resulted in similarly active compounds^15^.

Other replacement strategies include the substitution of naphthyl rings to heterobiaryls **3** and **4** such as quinoline, isoquinoline^16^, phthalazine^16^ indene^17^, benzimidazole^18^, benzothiophene^19^, indole^20^ and, more recently, benzazaborinine **5**^21^. Saturated analogues, such as tetrahydronaphthalenes, have also been used, which retain the rigidity and size of the original naphthyl ring system while introducing more three-dimensionality and potential increased solubility^22^.

Nonetheless, the current bioisosteric replacements for naphthyl rings lack generality and suffer from poor physicochemical properties. Fused heterocyclic ring systems (**3**–**5**) may have substantially different properties to the parent compound, such as lipophilicity, polar surface area and hydrogen-bonding groups, which can lead to loss of biological activity^23^. These ring systems also suffer from a high degree of planarity, often linked to poor physicochemical properties. Indeed, a higher degree of saturation (higher fraction of *sp*^3^-hybridized carbon atoms) in drug molecules has been demonstrated to increase the likelihood of clinical success, often rationalized by increased solubility or more efficient filling of three-dimensional target space^24^. The replacement of naphthyl groups with substituted phenyl rings (**1** and **2**) shows poor geometric overlap with the parent structure and has limited substitution possibilities. Finally, naphthyl substitution with saturated ring counterparts **6** may dramatically impact the original exit vectors of the aryl ring substituents and limit their application as true geometrical isosteres.

The isosteric replacement of *sp*^2^-heavy aromatic rings with *sp*^3^-rich small-ring cage hydrocarbon systems has emerged as a promising strategy towards improving the physicochemical and pharmacokinetic properties of drug candidates^25,26^. Notably, the bicyclo[1.1.1]pentane (BCP) motif **7** represents a popular approach towards the replacement of *para*-substituted arenes, with several examples displaying improved drug-like profiles^26,27^ (Fig. 1b).

Recently, Anderson and coworkers reported the synthesis and structural analysis of bicyclo[3.1.1]heptane (BCHep) derivatives **8**, which represent the *meta*-substituted arene counterpart for BCPs. The authors validated the BCHep scaffold as an alternative motif for improving the pharmacokinetic and physicochemical properties of *meta*-substituted drug candidates^28^. However, further investigation on the biological activity of BCHep-containing lead compounds is needed to evaluate them as true bioisosteres for the *meta*-substituted arene motif. Furthermore, we considered there could be scope to fuse the BCHep scaffold with six-membered aromatic rings, which would represent geometrical bioisosteres for fused ring systems such as naphthalene, quinoline, isoquinoline or quinazoline (Fig. 1c). Aryl-fused BCHeps are extremely rare and are currently limited to the patent literature^29^ or virtual molecules (for example, Enamine REAL), with no reported studies formally utilizing them as bioisosteres.

Herein, we report the preparation of aryl-fused BCHep scaffolds and establish that the geometry of the β-naphthyl **9** is conserved in BCHep **10**. We extend the scope of our systems to a range of ring substitution patterns **11** and **12** and heterocycles **13**, providing a chemical toolbox for bioisosteric replacement of naphthalene, (iso)quinoline and quinazoline. Finally, as a proof-of-concept study, we bioisosterically replace the naphthyl ring of utrophin modulator ezutromid, with a range of aryl-fused BCHeps, and demonstrate the retainment of bioactivity while improving metabolic stability to CYP enzymes. This study validates that aryl-fused BCHep scaffolds can function as ‘true’ bioisosteric replacements for *meta*-substituted arenes and naphthalene.

## Results and discussion

**Fig. 2:**
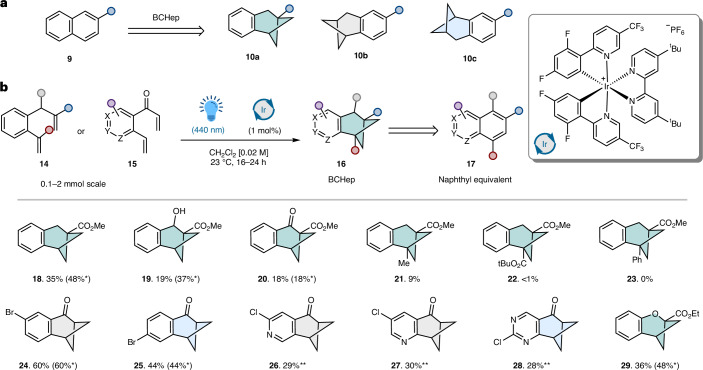
Synthesis and regiochemical diversity of aryl-fused BCHep scaffolds. **a**, Three possible regioisomeric bioisosteres are possible using aryl-fused BCHeps (colour-coded by regioisomer). **b**, The general strategy and conditions towards generating the BCHep core and list of [2+2] substrates prepared. Isolated yield: one asterisk is with 15 mol% pyrene, and two asterisks is with 25 mol% diphenyl phosphoric acid.

Compared with ‘simple’ *meta* arene isosteres **8**, we identified that BCHep derivatives of 2-naphthalene **9** benefit from three possible isostere regioisomers **10a**–**c** (Fig. 2a, colour-coded), allowing the fine-tuning of molecular properties. We envisaged a general preparation of these fused ring systems via an intramolecular crossed [2+2] cycloaddition with a divinyl precursor **14** or **15** (Fig. 2b) (see the Supplementary Information for full synthetic efforts). To our delight, it was possible to generate the aryl-fused BCHep framework **16** (functioning as isosteres of **17**) via an iridium-catalysed [2+2] photocycloaddition through visible light-induced energy transfer, adapting the methodology developed by Kwon and coworkers^30^. The authors reason that these reactions proceed via an excited styrene intermediate, which undergoes a formal [2+2] addition with an electron-deficient alkene, where the high selectivity arises from a stabilized benzyl radical intermediate. The iridium catalyst exhibits a suitable triplet energy for these substrates (60.1 kcal mol^−1^) and a long-lived triplet state (*τ* = 2,300 ns)^30^.

During our studies, we prepared β-naphthyl isostere **18**, which features a convenient bridgehead ester group for further functional group transformations. Although the ^1^H NMR spectra of the crude reaction mixtures for these reactions appeared to contain few impurities (and no observable regioisomeric bicyclo[3.2.0]heptane side product), yields were relatively modest. This prompted us to investigate the reaction progress via ^1^H NMR spectroscopy.

Using dimethyl sulfone as an internal standard, the ^1^H NMR yields ranged from 28% to 40% after 18 h of reaction time. This value could not be improved upon changing the catalyst or solvent (Supplementary Table 1). When monitored over several time points, the rate of starting material consumption was notably more rapid than product formation (Supplementary Fig. 175). This is consistent with oligomerization of the starting material and is supported by the presence of broad peaks across the full range of the crude ^1^H NMR spectra. It was not possible to suppress oligomerization by running the reaction under more dilute conditions (Supplementary Table 2).

Interestingly, it was possible to increase the ^1^H NMR yield to 56% when the reaction was performed in the presence of substoichiometric amounts of pyrene for longer reaction times (Supplementary Table 2). In the presence of pyrene, we hypothesize a triplet–triplet annihilation upconversion mechanism takes place, and the absorption of the P-type delayed fluorescence of pyrene by the diene substrates leads to the direct formation of the singlet excited states that can undergo intramolecular cyclization^31^. In the absence of pyrene, the excited substrates must undergo intersystem crossing from their excited triplet states to access the excited singlet state needed for ring closure to occur, thereby making oligomerization a competing pathway.

With suitable conditions in hand, we prepared a range of aryl-fused BCHeps with varied substitution patterns to diversify the potential exit vectors of the system (and covering the three possible 2-naphthyl regioisomers), while incorporating key functional handles for further derivatization (up to 2-mmol scale) (Fig. 2b). The cycloaddition was tolerant to substitution at the benzylic position, as exemplified with alcohol **19** and ketone **20**. Fused BCHeps with doubly substituted bridgehead positions (**21** and **22**) were obtained in poor yields and much more complicated crude mixtures, which led to troublesome purifications. In the case of **23**, the undesired linear [2+2] regioisomer was observed, rather than the desired crossed-isomer. Changes to the photocatalyst or reaction solvent did not improve product yields (Supplementary Fig. 175 and Supplementary Table 3). Therefore, we turned our attention to less heavily substituted BCHep derivatives. In the case of bridgehead-unsubstituted substrates **24** and **25**, yields were higher, in line with Kwon’s observations^30^. Under these conditions, the authors reported that pyridine-based substrates were not capable of undergoing [2+2] cycloadditions^30^. We speculated that this may be due to the Lewis basicity of the pyridine nitrogen. Gratifyingly, we observed that upon addition of 25 mol% diphenyl phosphoric acid to facilitate protonation of the pyridine nitrogen^32^, the corresponding heterocyclic derivatives **26**–**28** could be generated. Finally, it was possible to prepare ether derivative **29** in similar yields to the other substrates, blocking the potentially metabolically labile benzylic CH_2_ site^33^ and showcasing the versatility of the [2+2] methodology. The addition of pyrene allowed us to improve the yields for esters **18**, **19** and **29** by up to ~20%, whereas the triplet annihilator did not impact the yields of ketone substrates **20**, **24** and **25**.

**Fig. 3:**
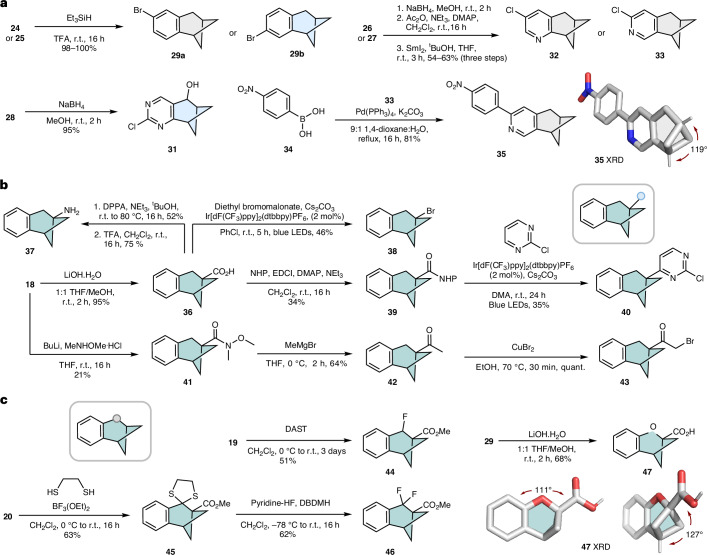
Chemical diversification and solid-state geometry of BCHep scaffold. **a**, The synthesis of ketone reduction substrates. **b**, The synthesis of substrates following bridgehead derivatization. **c**, The synthesis of substrates following CH_2_ replacement. r.t., room temperature; quant., quantitative yield.

With aryl-fused BCHeps **18**–**28** in hand, we explored their derivatization to provide further building blocks (Fig. 3a–c). It was possible to reduce aryl ketones **24** and **25** (Fig. 3a) in high yields under acidic conditions to generate **29a** and **29b**, which feature a convenient halide handle for cross-coupling. For heterocyclic derivatives **26**–**28** (Fig. 3a), it was necessary to reduce the ketone via alcohols such as **31**, followed by a deoxygenation protocol to afford saturated cross-coupling partners **32** and **33**. Isoquinoline isostere **33** was subjected to Suzuki cross-coupling conditions with **34** to afford the highly crystalline nitroarene **35**, which was characterized via X-ray crystallography. The angle across the bridgehead was 119°, consistent with the original reports of the BCHep frameworks **8** by Anderson and coworkers^28^, highlighting that the bridgehead isostere geometry was not affected by the addition of a fused arene.

To complement the functionality tolerated on the (hetero)aryl ring, we sought to derivatize the bridgehead to generate a library of the underrepresented β-naphthyl isostere building blocks (Fig. 3b). The hydrolysis of **18** afforded key intermediate **36**, which was successfully amide coupled to a complex aniline towards the preparation of an ezutromid derivative (Supplementary Scheme 13). It was also possible to convert acid **36** into primary amine **37**, which is expected to display an improved safety profile relative to the commonly toxic aryl amine motif^34^; **37** could also be converted to an amide derivative (Supplementary Scheme 12). The BCHep derivatives are also compatible with radical chemistry at the bridgehead, with acid **36** yielding bromide **38** via an iridium-catalysed decarboxylative bromination^35^ and *N*-hydroxyphthalimide **39** undergoing a decarboxylative Minisci reaction to provide heterocyclic derivative **40** (ref. ^36^). We also successfully prepared Weinreb amide **41** from **18**, which readily generated ketone **42**. This substrate could be brominated under mild conditions to afford bromoketone **43**, a versatile building block towards the synthesis of heterocycles^37^.

We also explored the possibility of blocking the benzylic CH_2_ (Fig. 3c) site by preparing mono- and difluoro derivates **44** and **46** from dithiane **45**, highlighting the potential to further suppress the metabolism of these aryl-fused BCHeps via CYP-mediated benzylic oxidation^33^. Finally, we generated the free acid **47** of ether **29** to elucidate the effect of the oxygen on bond angles via X-ray diffraction (XRD). We observed that the β-naphthyl angle is reduced from 120° to 111°, whereas the bridgehead distances are increased to 127°. This highlights the importance of a full-carbon framework to facilitate geometrical isosterism but allows the opportunity to modulate the angles within the naphthyl isostere framework.

**Fig. 4:**
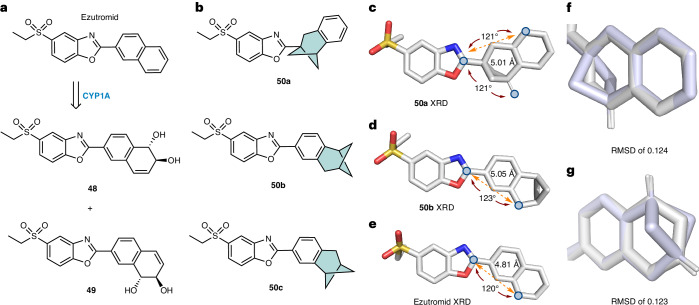
Metabolic liability of ezutromid and structural comparison with BCHep analogues. **a**, The CYP1A-mediated oxidation of ezutromid. **b**, The list of BCHep-ezutromid derivatives. **c**, The X-ray solid-state structure of **50a**. **d**, The X-ray solid-state structure of **50b**. **e**, The X-ray solid-state structure of ezutromid (unit cell contained two ‘downward’ facing naphthyl conformers, where each conformer exhibits a unique sulfone conformation). **f**, The stacked solid-state structures of the BCHep unit of **50a** (green) with ezutromid (grey). **g**, The stacked solid-state structures of the BCHep unit of **50b** (green) with ezutromid (grey). RMSD values were calculated using the ‘pair-fit’ function in Pymol and mapping the naphthyl and BCHep carbon atoms.

We then sought to validate these scaffolds as true naphthyl isosteres by replacing the naphthalene ring of ezutromid with the BCHep motif (Fig. 4). Ezutromid was a first-in-class utrophin modulator that was evaluated in a phase 2 clinical study for the treatment of DMD^38^. DMD is a disease caused by multiple different loss-of-function mutations in the gene encoding the structural muscle protein dystrophin. Ezutromid is known to function by upregulating the protein utrophin in muscle, which can compensate for the missing dystrophin in patients with DMD regardless of the disease mutation type^10^. Clinical studies with repeated dosing of ezutromid displayed promising 24-week data, with a statistically significant reduction in muscle fibre damage and increased levels of utrophin, providing the first evidence of target engagement and proof of mechanism. However, ezutromid failed to meet its primary or secondary end points after 48 weeks of treatment, and further development was discontinued^39^. Subsequent studies on ezutromid revealed that the lack of sustained therapeutic efficacy could be ascribed to the naphthalene unit, which is susceptible to hepatic CYP-mediated oxidation^10^ (Fig. 4a). We previously reported that the prolonged dosing of ezutromid induces a paradoxical increase in CYP1A activity resulting in a reduced exposure of the drug over time and increased production of inactive dihydrodiol metabolites **48** and **49**, accounting for its reduction of efficacy over time^10^. Therefore, we envisaged the replacement of the naphthyl ring with the isosteric BCHep counterparts **50a**–**c** could suppress CYP1A-mediated oxidation of the naphthalene (Fig. 4b), potentially circumventing the issue observed with ezutromid. **50a** was prepared by the amide-coupling of **36** followed by condensation into the benzoxazole, whereas regioisomers **50b** and **50c** were accessed by a C–H activation/cross-coupling protocol from **29a** and **29b** (Supplementary Scheme 13).

To compare the geometry of the BCHep motif relative with the parent compound, we prepared single crystals of regioisomers **50a** and **50b** and ezutromid and analysed them by X-ray crystallography (Fig. 4c–e). To our delight, the β-naphthalene and bridgehead substituents retained the desired ~120° angle, whereas the distance between the *meta-*substitution pattern was within 5% of the original geometry of the naphthalene isostere. Overlaying the BCHep portion of **50a** and **50b** with the naphthyl unit of ezutromid revealed a low root mean square deviation (RMSD) of ~0.1 Å (Fig. 4f,g), highlighting the high geometrical isosterism of these motifs. Overlaying the full solid-state structures still resulted in a good structural similarity across the entire drug molecule (Supplementary Fig. 177). Moreover, the calculated electrostatic potential maps for **50a** and ezutromid showed high similarity (Supplementary Figs. 180 and 181).

**Fig. 5:**
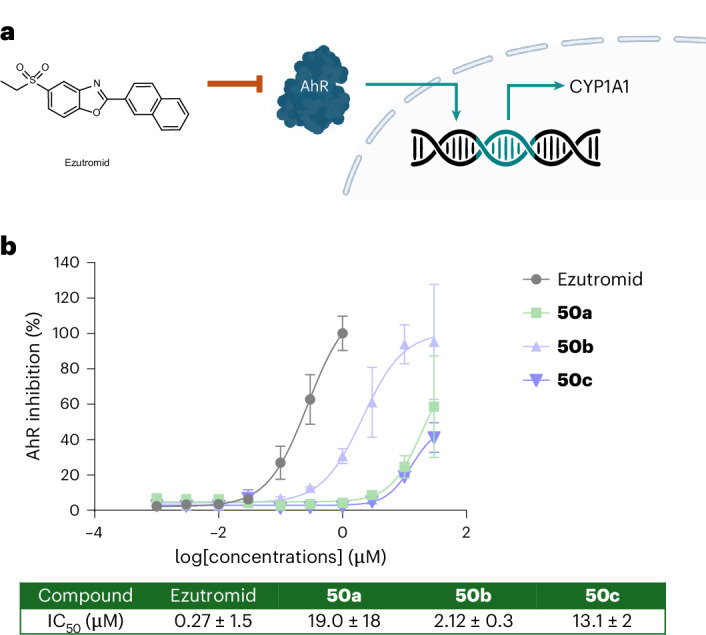
AhR antagonism and biological evaluation of BCHep analogues. **a**, A schematic representation of how ezutromid antagonizes AhR preventing its translocation into the nuclei and its subsequent upregulation of CYP1A1 enzyme. The inhibition of AhR can therefore be evaluated as a decrease in CYP1A1 expression. **b**, The evaluation of **50a–c** as AhR antagonists compared with ezutromid. Data have been obtained by quantitative RT-qPCR and analysed using the 2^−∆∆Ct^ method. Values obtained have been normalized to control (DMSO 1%) and presented as mean + s.e.m. The IC_50_ values were determined from biological triplicates, except for compounds **50c** and ezutromid, which were measured from duplicate experimental replicates.

Encouraged by these results, we sought to assess the derivatives **50a**–**c** for their biological activities relative to ezutromid. To this aim, we decided to measure the activity of ezutromid and the analogues against ezutromid’s primary target, which has recently been identified as the aryl hydrocarbon receptor (AhR)^40^. This nuclear transcription factor plays a pivotal role in the metabolism of exogenous small molecules by targeting nuclear DNA to upregulate the expression of several isoforms of CYP enzymes responsible for the phase I metabolism of drugs, such as CYP1A isoforms (Fig. 5a). Therefore, AhR inhibition can be monitored by measuring the relative expression of these genes, with lower *CYP1A1* mRNA levels being proportional to AhR antagonism.

To determine the activity of compounds **50a–c** as AhR antagonists, we quantified *CYP1A1* mRNA levels in human liver cell line HepG2 by quantitative real-time polymerase chain reaction (RT-qPCR) after 4 h of incubation with test compounds. All analogues retained biological activity when tested at a 10 μM concentration, showing a statistically significant reduction of *CYP1A1* expression (Supplementary Fig. 178), albeit with a reduced potency when compared with ezutromid (Fig. 5). Interestingly, a concentration–response analysis showed there was a notable difference in activity between each regioisomer of **50a–c**, suggesting that there may be variations in conformational preferences and/or a preference for retaining a 2-aryl substituent on the 2-position of the benzoxazole, consistent with previous SAR studies^38^. The most potent regioisomer was **50b** (half maximal inhibitory concentration (IC_50_) 2.12 ± 0.3 μM versus ezutromid, IC_50_ 0.27 ± 1.5 μM). The solid-state structures reveal that the BCHep unit of the most active derivative **50b** points downwards (on the oxygen side of the oxazole), whereas the less active **50a** resides in a conformation where the naphthyl isostere faces upwards (on the nitrogen side of the oxazole); suggesting that the downwards conformation may be preferable for achieving higher activity. This is in agreement with the highly active ezutromid, whose solid-state unit cell is a disordered structure containing two ‘downward’-facing naphthyl conformers (Supplementary Fig. 183).

Similarly, the computed structures of the less active **50a** and **50c** exhibit an energetic preference towards an ‘upward’ BCHep conformation, whereas the most active **50b** is downwards, consistent with the the solid-state structures (Supplementary Fig. 183). For ezutromid, both naphthyl conformers were computationally indiscriminate in energy.

Finally, we subjected the most promising derivatives to metabolic stability studies in mouse liver microsomes (MLMs), a validated model for understanding phase I metabolism of drugs (Table 1). To investigate whether the analogues would be susceptible to CYPs metabolism, we repeated these tests in the presence of 3 μM α-naphthoflavone (ANF). ANF is a well-known inhibitor of CYP1A enzymes, which is responsible for the CYP oxidative metabolism of naphthalene ring in ezutromid^41^. Interestingly, these derivatives showed a similar or higher metabolic stability relative to ezutromid, with comparable half-lives. In particular, compound **50a** showed a ~2-fold increase in metabolic half-life compared with ezutromid (Table 1). The most promising compound **50b** showed a slight decrease in half-life compared with ezutromid. Importantly, the stability of BCHep analogues in MLMs was not affected by the presence of ANF, demonstrating that the bioisosteres successfully avoid the CYP1A-mediated metabolism that is responsible for hampering ezutromid’s sustained efficacy (Table 1). The metabolic profiling of **50b** confirmed that its predominant biotransformation involves the mono-oxygenation of the BCHep moiety (Supplementary Figs. 179 and 180), most likely via other enzymes (for example, CYP3A4) that are not under control of AhR. These findings support the strategy that BCHep substitution mitigates the metabolic liabilities associated with ezutromid, by both preserving metabolic stability and avoiding CYP1A-mediated metabolism.

**Table 1:** Metabolic stability of analogues **50a–c**

Drug	*T*_1/2_ (min)	CL_int(mic)_ (μl min^−1 ^mg^−1^)	CL_int_(liver) (ml min^−1 ^kg^−1^)
Ezutromid	60 ± 5	23.3 ± 2.0	92.4 ± 8.3
Ezutromid + ANF	89 ± 6	15.8 ± 1.1	62.6 ± 4.3
**50a**	94 ± 0.2	14.7 ± 0.1	58.4 ± 0.4
**50a** + ANF	108 ± 11	12.8 ± 1.4	50.6 ± 5.4
**50b**	40 ± 7	35.6 ± 7.0	140.9 ± 27.9
**50b** + ANF	42 ± 4	33.6 ± 2.7	133 ± 10.4
**50c**	48 ± 8	29.9 ± 4.9	118.3 ± 19.3
**50c** + ANF	56 ± 10	25.7 ± 4.6	102.0 ± 18.1

Stability of analogues was tested in MLM at a concentration of 1 μM with or without 3 μM ANF, a known inhibitor of CYPs enzymes. CL_int(mic)_ refers to intrinsic clearance in MLM. CL_int_(liver) is calculated from CL_int_ and represents an estimated intrinsic clearance in mouse liver. Measurements were performed in triplicate; errors reported as s.d.

## Conclusion

Current bioisosteric replacements for naphthyl rings lack generality and suffer from poor physicochemical properties. We describe the preparation of a diverse series of easily derivatizable *sp*^3^-rich aryl-fused BCHeps, serving as bioisosteres for a range of bicyclic (hetero)aromatics, including naphthalene, (iso)quinoline and quinazoline, and the underrepresented β-naphthyl unit. The ring frameworks were efficiently accessed via an intramolecular crossed [2+2] photocycloaddition through visible light-induced energy transfer and can be readily transformed into a library of bicyclic isostere building blocks with a range of substitution patterns across the framework.

The incorporation of the BCHep isosteres within the AhR antagonist ezutromid, a compound limited by high susceptibility to CYP1A-mediated metabolism arising from its naphthyl ring, led to analogues with improved metabolic stability and, more particularly, which limit CYP1A metabolism. With the aid of solid-state structures, cellular assays and metabolic stability studies in MLM, we demonstrate that the aryl-fused BCHep derivatives exhibit geometrically similar substituent vectors to the parent compound, retain interesting biological activity and display increased metabolic stability relative to their naphthyl counterpart. Metabolic profiling confirmed that BCHep substitution mitigates the formation of reactive metabolites characteristic of the parent compound. Among the series, compound **50b** retained meaningful AhR antagonistic activity while successfully addressing the key metabolic liabilities, making it a promising candidate for further optimization.

This study represents the successful incorporation of a BCHep isostere into a potential pharmaceutical candidate, while simultaneously improving pharmacokinetic properties and maintaining cellular target engagement. These findings validate BCHep as a promising bioisosteric motif for *meta*-substituted arenes and naphthalenes, offering a balanced optimization of metabolic stability and biological activity and opening avenues to a wide range of potential applications in future medicinal chemistry programs.

## Methods

All reagents and solvents were purchased from commercial sources and used without further purification. Where necessary, solvents were dried by passing through an MBraun MPSP-800 column and degassed with nitrogen. Triethylamine was distilled from and stored over potassium hydroxide. Column chromatography was carried out on Merck silica gel 60 under a positive pressure of nitrogen. Where mixtures of solvents were used, ratios are reported by volume. NMR spectra were recorded on a Bruker AVIII 400, Bruker AVII 500 (with cryoprobe), Bruker NEO 600 with broadband helium cryoprobe and Bruker AVIII 500 spectrometers. Chemical shifts are reported as *δ* values in parts per million. Mass spectra were carried out on a Waters Micromass LCT and Bruker microTOF spectrometers.

### NMR time course experiments

Cyclization substrate, Ir[dF(CF_3_)ppy]_2_(dtbbpy)PF_6_ (1 mol%) and internal standard (dimethyl sulfone, 1 equivalent) were dissolved in degassed CD_2_Cl_2_ (0.70 ml) and transferred into an NMR tube. The vessel was irradiated with a light-emitting diode (LED) lamp (Kessil PR160L-440nm—highest setting) (~2-cm distance) while being cooled by direct exposure to a constant stream of N_2_. The ^1^H NMR spectra were acquired at regular intervals and yields determined through integration relative to the internal standard.

### Photochemistry reaction set-up

The reaction flasks or vials are clamped above a stir plate. The LED lamp (Kessil PR160L-440nm—highest setting) is placed perpendicular (~2-cm distance) to the side wall of reaction flask. The stir plate and LEDs are surrounded by a light-protective shield and aluminium foil. The reaction flask is cooled by direct exposure to a constant stream of N_2_. See the Supplementary Information for a picture of the set-up.

### Crystallographic data

Crystals were grown by vapour diffusion. Compounds (2–5 mg) were dissolved in minimal amounts of CDCl_3_, then filtered over cotton into a small vial. The vial was capped and punctured with a small needle, then placed into a larger vial containing pentane or hexane. Single crystal XRD data were collected on a Rigaku Synergy-DW diffractometer at 100 K for all structures. CrysAlisPro was used for data integration and absorption correction. Structures were solved using Superflip^15^ before refinement with CRYSTALS^16,17^ as per the Supplementary Information (CIF).

### Cell culture and treatment

Human hepatocellular carcinoma (HepG2) cell lines were acquired from Abcam. Cells were cultured in Dulbecco’s modified Eagle medium or minimum essential medium Eagle with Earle′s salts supplemented with 10% fetal bovine serum in 5% CO_2_ atmosphere at 37 °C. For treatment, cells were seeded at 50,000 cells per well in 96-well transparent plates and allowed to grow to 80% confluence. After 48 h, cells were washed with phosphate-buffered saline, and test compounds (**50a–c**, at concentrations of 0.1, 1 or 10 μM with 1% dimethyl sulfoxide (DMSO); technical duplicate), positive control (ezutromid, at concentrations of 0.1, 1 or 10 μM with 1% DMSO; technical duplicate) or vehicle (1% DMSO, technical quadruplicate) were administered. For dose response analyses, cells were seeded at 250,000 cells per well in six-well plates. After 24 h, media were removed, and cell test compounds (**50a–c**, at concentrations of 1 nM, 3 nM, 10 nM, 30 nM, 100 nM, 300 nM, 1 μM, 3 μM, 10 μM or 30 μM with 0.3% DMSO; technical duplicate), positive control (ezutromid, at concentrations of 10 μM with 0.3% DMSO; technical duplicate) or vehicle (0.3% DMSO, technical duplicate) were administered. Cells were incubated for 4 h before cell lysis and RNA extraction.

### RNA extraction and cDNA synthesis

The total RNA was extracted using the MagMAX™-96 Total RNA Isolation Kit (Thermo Fisher Scientific) or using the RNeasy Plus Mini Kit (QIAGEN, 74136) following manufacturer’s instructions. In brief, cells were lysed using the provided lysis buffer, and RNA was extracted using nucleic acid capturing magnetic beads. Genomic DNA was removed on-beads using the TurboDNAse kit (Thermo Fisher Scientific), and RNA was purified by repeated washing steps, before being eluted into eight-tube PCR strips and stored at −20 °C overnight. The quality and quantity of extracted RNA were assessed using a NanoDrop One Microvolume or Lite Plus UV–Visible Spectrophotometer (Thermo Fisher Scientific). Complementary DNA (cDNA) was synthesized from 200 ng of total RNA using the High-Capacity cDNA Reverse Transcription Kit (Thermo Fisher) or from 250 ng of total RNA using QuantiTect Reverse Transcription Kit (200) (QIAGEN, 205313) according to the manufacturer’s protocol.

### Quantitative reverse transcription PCR analysis

The RT-qPCR analysis was performed using Fast SYBR Green Master Mix (Thermo Fisher Scientific) in 384-well plate on a LightCycler 480 II (Roche) or in a 96-well plate on a Applied BiosystemsTM 7500 Fast RT-qPCR System (Thermo Fisher Scientific). Each reaction was run in duplicate using cDNA synthesized from two individual wells. Amplification was carried out using the following custom primer pairs obtained by Merck: hS13 (housekeeping gene): forward—CTGATCTTCCTGAAGATCTCTAC, reverse—GGCAGAGGCTGTAGATGATTCA; *hCYP1A1* (gene of interest) forward—GCTCCAAGAGTCCACCCTTCCC, reverse—CTGAGGTCTTGAGGCCCTGATTACC.

### Data analysis

Values obtained from the quantitative PCR amplification were analysed using the 2^−∆∆CT^ method to determine fold difference compared with the vehicle^18^. For enhancing data visualization, values were normalized against vehicle control and converted to percentage to provide a precise estimate of AhR inhibition as directly proportional to *CYP1A1* downregulation. A total of four independent experiments were performed (*n* = 4). One-way analysis of variance followed by Dunnett’s multiple comparisons test was performed using GraphPad Prism v.8.0.2 for Windows (GraphPad Software).

### Reporting summary

Further information on research design is available in the Nature Portfolio Reporting Summary linked to this article.

## Online content

Any methods, additional references, Nature Portfolio reporting summaries, source data, extended data, supplementary information, acknowledgements, peer review information; details of author contributions and competing interests; and statements of data and code availability are available at 10.1038/s41557-026-02129-2.

## Supplementary information

Supplementary Information Supplementary methods, full reaction schemes and biological data.

Reporting summary

## Data Availability

All data supporting the findings of this study are available within the Article and its Supplementary Information. The crystallographic data have been deposited at the Cambridge Crystallographic Data Centre, under deposition numbers CCDC 2368065 (**35**), CCDC 2368066 (**47**), CCDC 2368067 (**50a**), CCDC 2368068 (**50b**) and CCDC 2368069 (**Ezutromid**). Copies of these data are available free of charge from the Cambridge Crystallographic Data Centre at www.ccdc.cam.ac.uk. The X-ray.cif files were opened with the Pymol Educational Version. Bond angles and distances were measured using the measurement wizard, and structural overlays were performed using the pair-fit function in Pymol.
