# Supplementary material for: Synthesis of aryl-fused bicyclo[3.1.1]heptanes and validation as naphthyl bioisosteres
Source: Nat Chem. 2026 May 6;18(6):1075–81. doi: 10.1038/s41557-026-02129-2 (PMC13236579; doi:10.1038/s41557-026-02129-2)
Supplement: Supplementary file 1 — Supplementary methods, full reaction schemes and biological data. [file 41557_2026_2129_MOESM1_ESM.pdf]

nature chemistry

Supplementary information https://doi.org/10.1038/s41557-026-02129-2

Synthesis of aryl-fused bicyclo[3.1.1] heptanes and validation as naphthyl bioisosteres

In the format provided by the authors and unedited

Table of contents

1. Materials and Methods 2
2. Full Reaction Schemes 4
3. Synthetic procedures and Characterisation Data 14
4. NMR Spectra 49
5. Condition Screening for [2+2] 136
6. Crystallographic Data 138
7. Biological Methods and Data 146
8. Computational Studies 150
9. References 157

1. Materials and Methods

All reagents and solvents were purchased from commercial sources and used without further purification. Where necessary, solvents were dried by passing through an MBraun MPSP-800 column and degassed with nitrogen. Triethylamine was distilled from and stored over potassium hydroxide. Column chromatography was carried out on Merck® silica gel 60 under a positive pressure of nitrogen. Where mixtures of solvents were used, ratios are reported by volume. NMR spectra were recorded on a Bruker AVIII 400, Bruker AVII 500 (with cryoprobe), Bruker NEO 600 with broadband helium cryoprobe and Bruker AVIII 500 spectrometers. Chemical shifts are reported as δ values in ppm. Mass spectra were carried out on a Waters Micromass LCT and Bruker microTOF spectrometers.

Abbreviations

δ: chemical shift; Δ: change in; λ: wavelength; μ: micro; μW: Microwave; br.: Broad; Boc: tert-butyloxycarbonyl; BINAP: (2,2′-bis(diphenylphosphino)-1,1′-binaphthyl); Bu: Butyl; Bz: benzoyl; cat: catalytic; conc.: concentrated; °C: degrees Celcius; CSA: camphor sulfonic acid; CYP1A1: Cytochrome P450, family 1, subfamily A, polypeptide 1; *d*: deuterated; d: doublet; DABCO: 1,4-diazabicyclo[2.2. 2]octane; DAST: Diethylaminosulfur trifluoride; DBDMH: 1,3-Dibromo-5,5-Dimethylhydantoin; DBU: 1,8-Diazabicyclo[5.4.0]undec-7-ene; DCM: Dichloromethane; DIBAL: Diisobutyl aluminium hydride; DIPA: diisopropylamine; DIPEA: N,N-Diisopropylethylamine; DMA: dimethylacetamide; DMAP: 4-Dimethylaminopyridine; DMF: *N*,*N*-Dimethylformamide; DMP: Dess-Martin Periodinane; DMSO: Dimethylsulfoxide; DPPA; Diphenylphosphoryl azide; dppf: 1,1'-Bis(diphenylphosphino)ferrocene; EDCI: 1-Ethyl-3-(3-dimethylaminopropyl)carbodiimide hydrochloride; eq.: equivalent; Et: ethyl; h: hour(s); HATU: Hexafluorophosphate Azabenzotriazole Tetramethyl Uronium; HMDS: hexamethyldisilazide; HRMS: High resolution mass spectrometry; Hz: hertz; Ir[dF(CF_3)ppy]bio$_2$(dtbpy)PF_6: [4,4′-*Bis*(1,1-dimethylethyl)-2,2′-bipyridine-*N*1,*N*1′]*bis*[3,5-difluoro-2-[5-(trifluoromethyl)-2-pyridinyl-*N*]phenyl-C]Iridium(III) hexafluorophosphate L: litre; m: metre, milli or multiplet; m: meta; M: molar concentration; MeCN: Acetonitrile; MeOH: Methanol; min: minute(s); MLM: mouse liver microsomes; MS: mass spectrometry; NBS: *N*-Bromosuccinimide; NHP: N-hydroxy phthalimide; *o*: ortho; p: para; Ph: Phenyl; Pr: propyl; q: quartet; Quant: Quantitative; rt: Room temperature; RT-qPCR: real time quantitative polymerase chain reaction; s: singlet or second; sat.: saturated; t: tertiary alkyl group; TFA: Trifluoroacetic acid; THF: Tetrahydrofuran; TLC: thin layer chromatography; Ts: toluenesulfonyl; X,Y, Z: heteroatom.

NMR time course experiments

Cyclisation substrate, Ir[dF(CF_3)ppy]$_2$(dtbbpy)PF_6 (1 mol%), and internal standard (dimethyl sulfone, 1 eq) were dissolved in degassed CD_2Cl_2 (0.70 mL) and transferred into an NMR tube. The vessel was irradiated with an LED lamp (Kessil PR160L-440nm – highest setting) (~2 cm distance) while being cooled by direct exposure to a constant stream of N_2. ^{1}H NMR spectra were acquired at regular intervals and yields determined through integration relative to the internal standard.

Photochemistry reaction Setup

The reaction flasks or vials are clamped above a stir plate. The LED lamp (Kessil PR160L-440nm – highest setting) is placed perpendicular (~2 cm distance) to the sidewall of reaction flask. The stir plate/LED's are surrounded by a light-protective shield and aluminium foil. The reaction flask is cooled by direct exposure to a constant stream of N_2. (See overleaf)

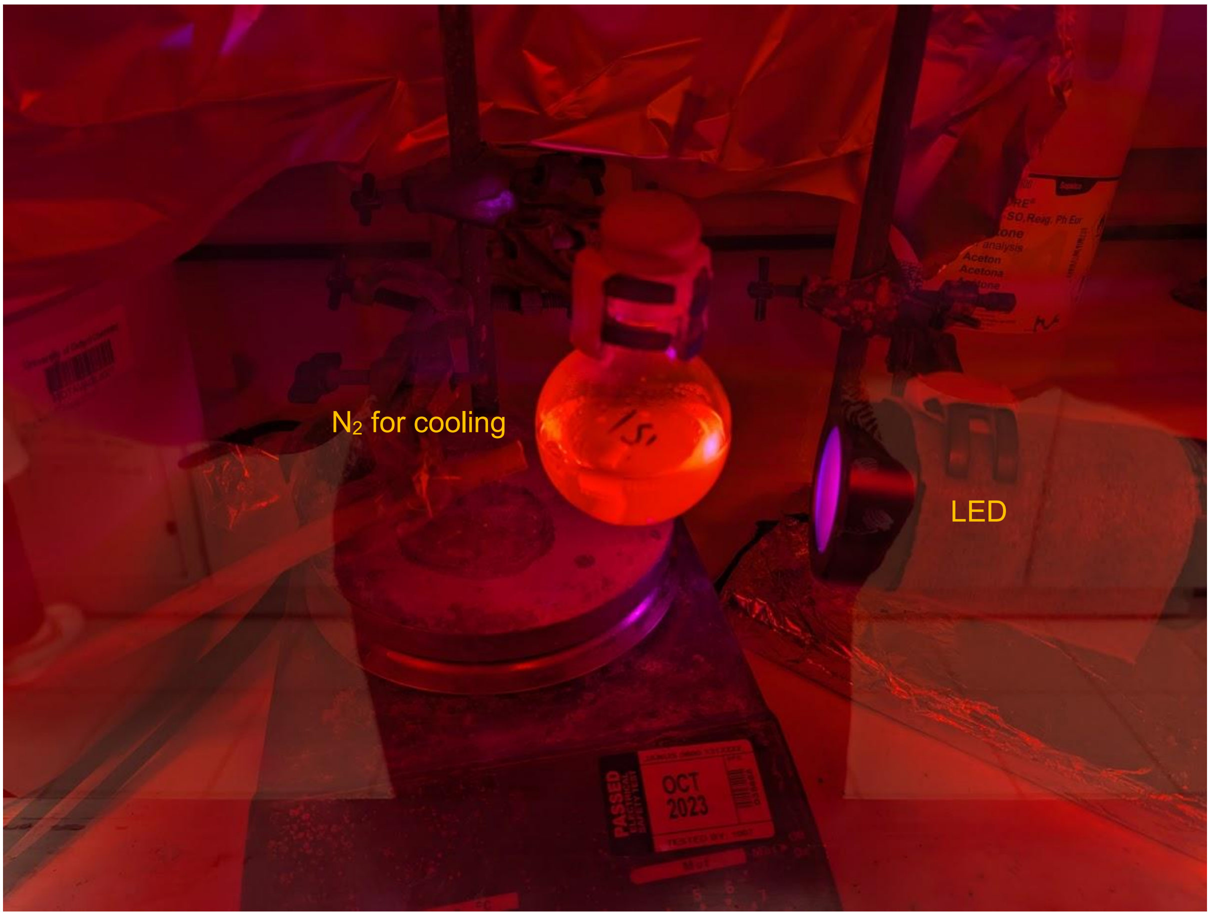
N2 for cooling
LED

2. Full Reaction Schemes

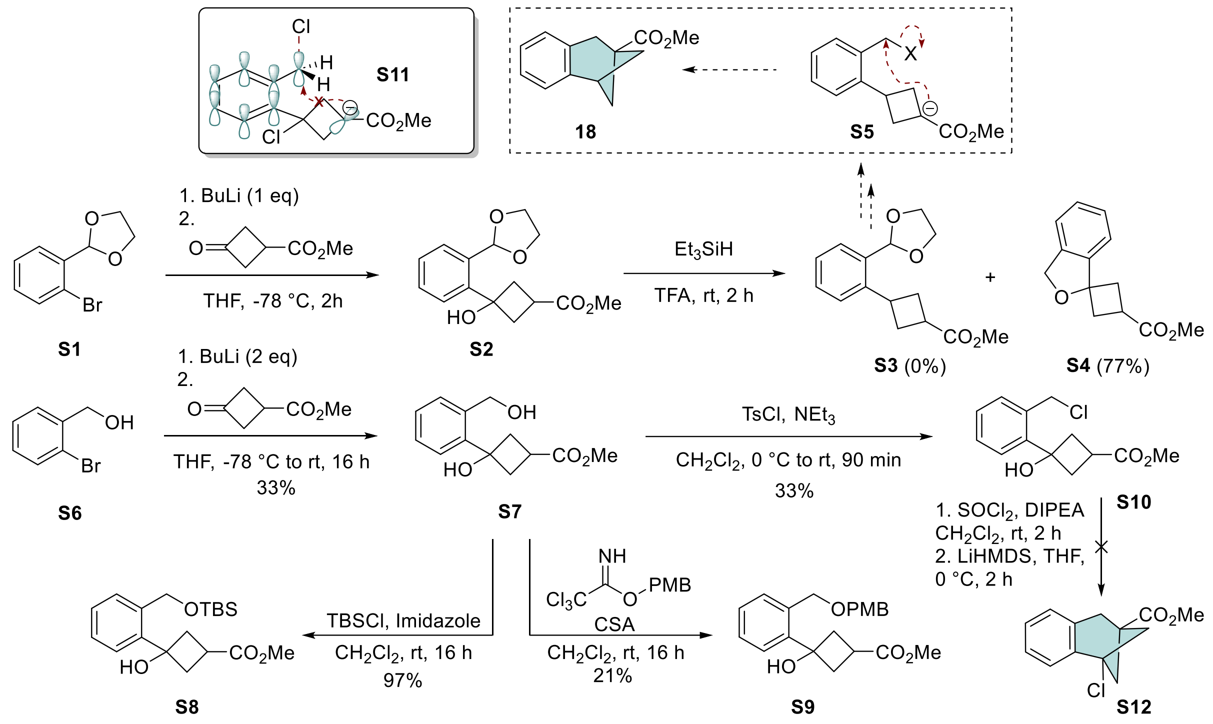

Scheme 1. Unsuccessful strategy towards intramolecular S_N2 substrate **S5** towards the preparation of **18**. The first route involved treatment of **S1** with BuLi and trapping with methyl 3-oxocyclobutane-1-carboxylate to afford tertiary alcohol **S2**. Attempts to deoxygenate **S2** afforded **S4** rather than the desired **S3**. We also attempted to access **S5**-type substrates by converting **S6** to **S7** and protecting the primary alcohol to **S8** and **S9**. Deoxygenation of the tertiary alcohol of **S8-9** was unsuccessful, often affording complex mixtures containing **S4**. Tosylation of **S7** provided unexpected product **S10**, which was converted to the tertiary chloride and treated with Base to probe whether intramolecular ring close was possible to **S12**. No cyclisation product was detected, with the CH_2Cl unit present in crude NMR mixtures, perhaps due to the inability of the **S11** to form the required benzylic S_N2 transition state. Therefore, we turned our attention to a photochemical [2+2] strategy.

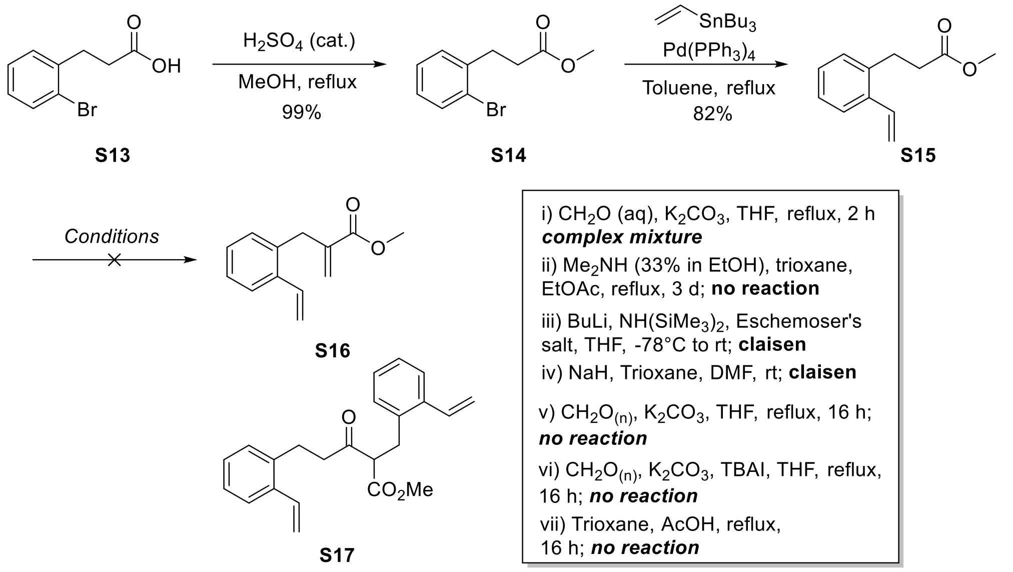

Scheme 2. Initial route towards Diene substrate **S16**. It was possible to install the methyl ester **S14** from **S13**, and build in the styrene unit via a Stille reaction to afford **S15**. However, **S15** proved a challenging substrate for methylenation. A series of conditions were screened but no reaction occurred under acidic conditions, whereas under basic conditions, Claisen condensation to **S17** typically took place. Therefore, we devised an alternative route to **S16**.

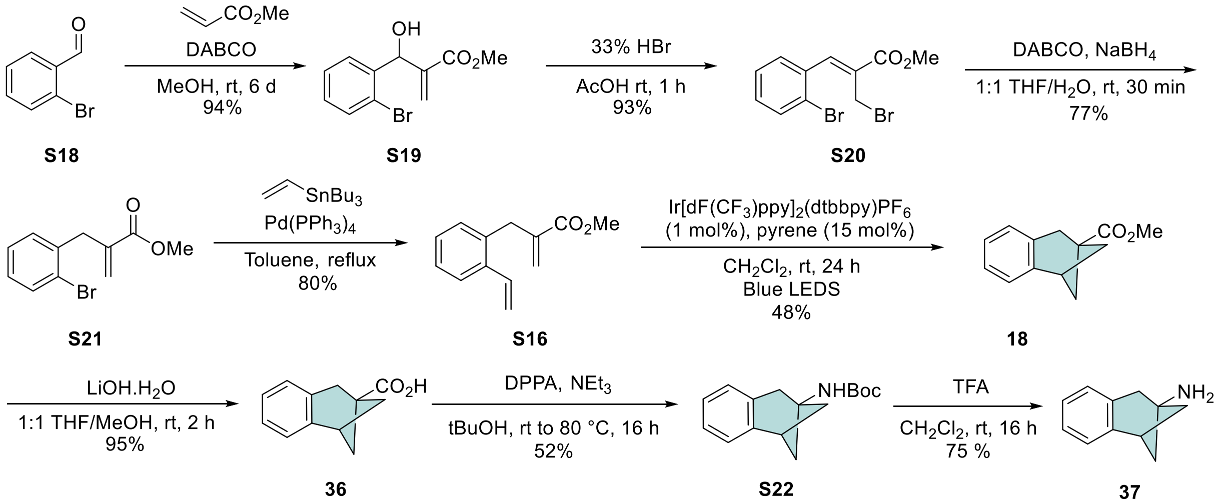

Scheme 3. Successful route to fused BCHeps. A Bayliss-Hillman reaction using **S18** and methyl acyrlate afforded **S19,** followed by an SN2' reaction to **S20** and reduction to **S21**. The Stille reaction afforded key intermediate **S16**, which was subjected to the photocatalysis conditions to give rise to **18**. This was successfully converted to acid **36**, which underwent a Curtius rearrangement to **S22**, allowing access to **37** by removal of the boc protecting group.

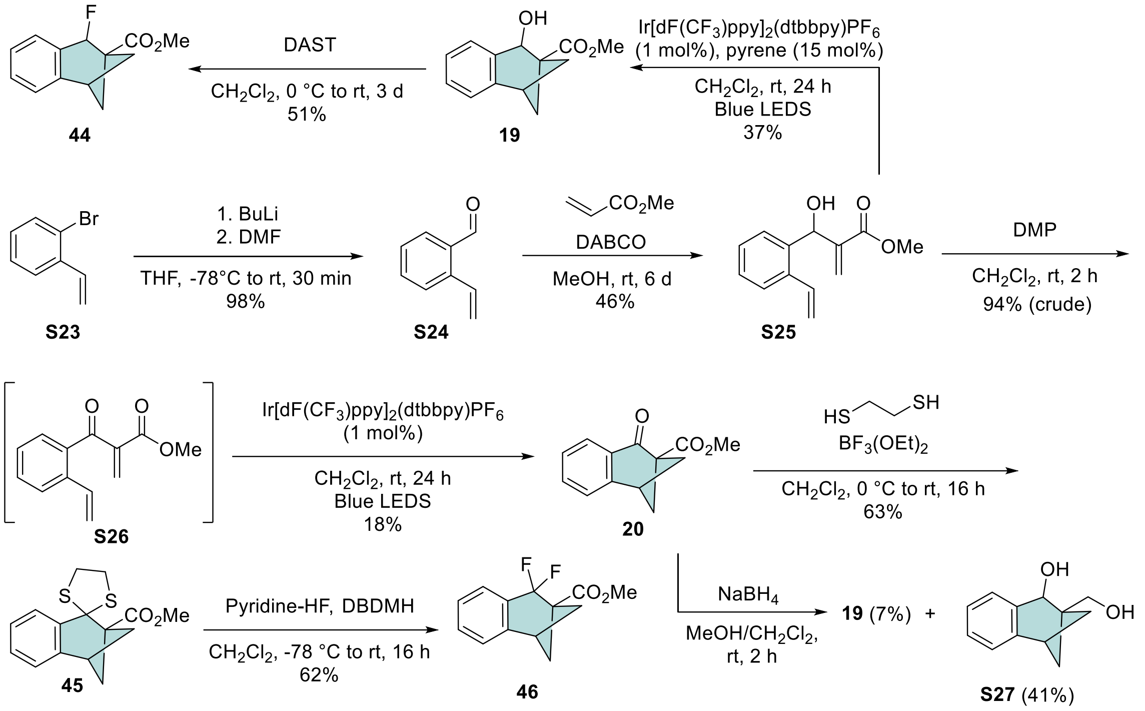

Scheme 4. Preparation of substrates with substitution at the benzylic position. Lithiation and formylation of **S23** provided vinyl aldehyde **S24**, which was subjected to Bayliss-Hillman conditions to provide **S25**. **S25** was successfully converted to the BCHep derivative **19**, which was converted to the fluorinated substrate **44** with DAST. It was also possible to generate **S26** from **S25**, which was unstable to silica-gel chromatography. The crude mixture was used to prepare ketone **20**. Direct reduction of **20** resulted in overreduction to side product **S27**. Direct difluorination of **20** was not possible, with complete recovery of starting material using standard fluorination conditions (DAST, cat. EtOH) and heating. Therefore, we transformed **20** into dithiane **45**, which was smoothly fluorinated to provide **46**.

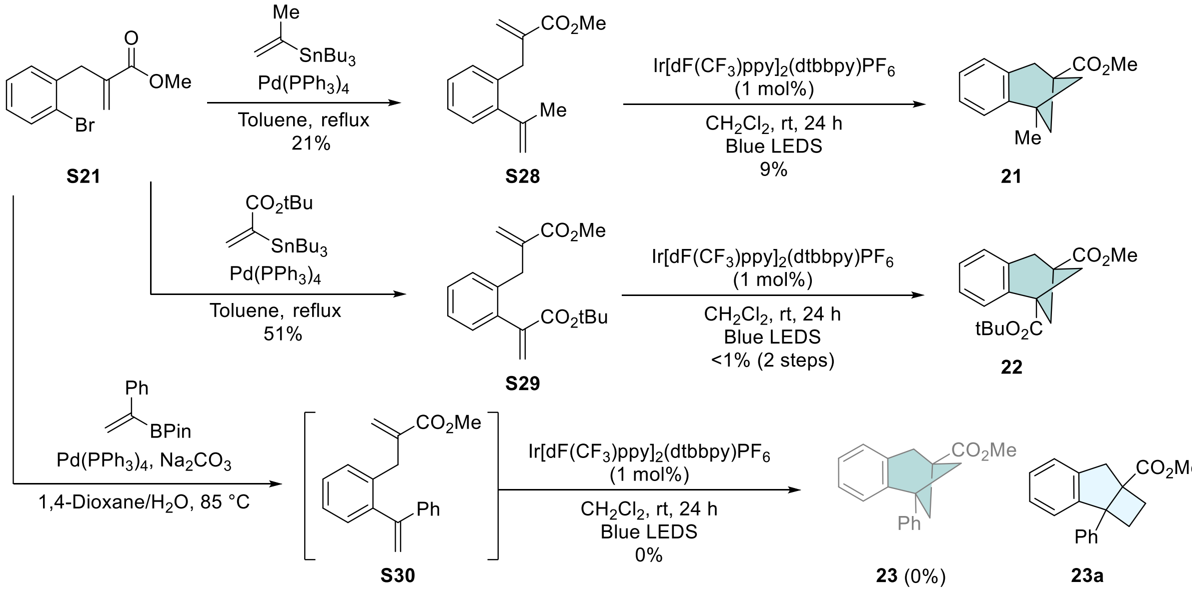

Scheme 5. Preparation of disubstituted substrates. Key intermediate **S21** was subjected to Stille or Suzuki coupling conditions to provide **S28-S30**. **S30** was not isolable in high enough purity for characterisation. Each diene was cyclised to the corresponding BCHeps **21-22**. Only **23a** was observed when **S30** was subjected to the conditions.

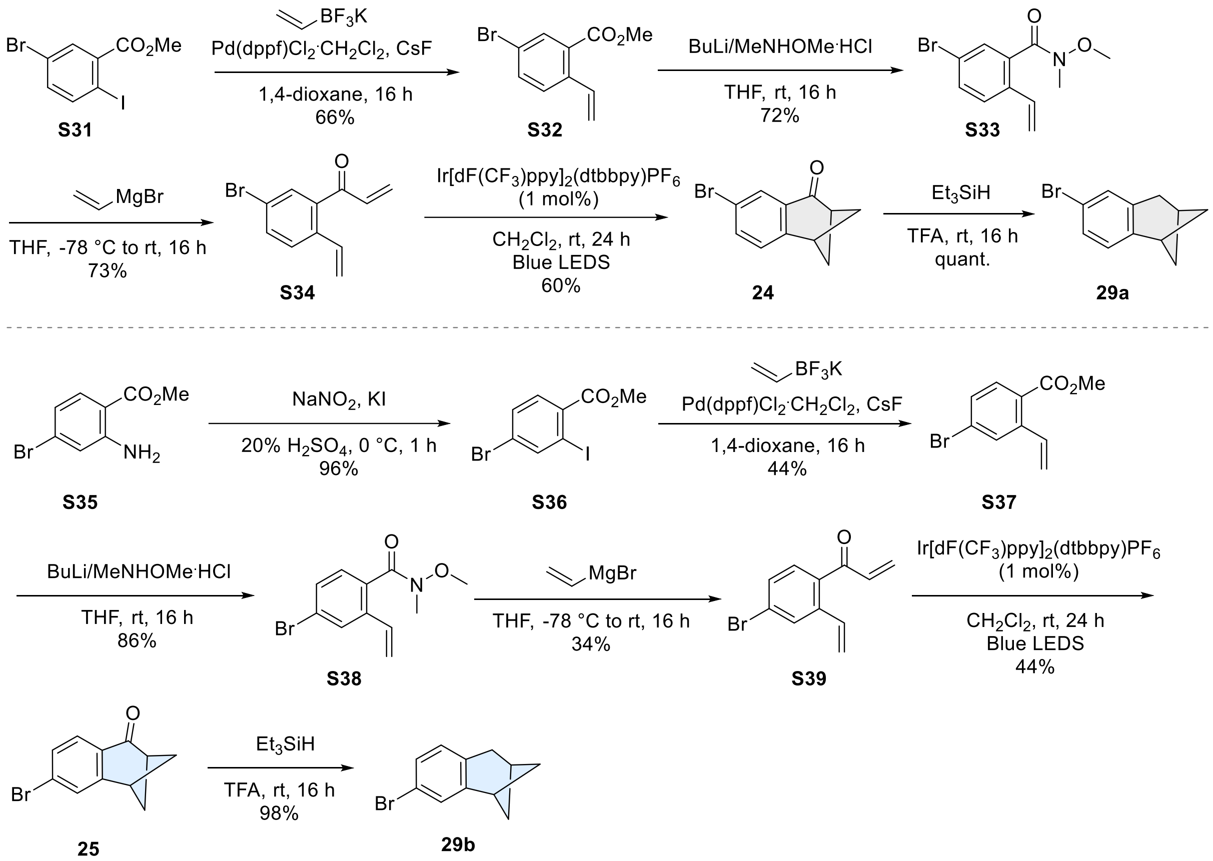

Scheme 6. Preparation of unsubstituted Naphthyl isostere derivatives. **S31** was subjected to a selective Suzuki coupling to afford **S32**, followed by direct Weinreb amide formation to **S33**. This was treated with vinylmagnesium bromide to afford **S34**, which was cyclised into BCHep derivative **24**. This substrate was reduced using triethylsilane/TFA to generate building block **29a**. Similarly, **S35** was converted into the corresponding iodide **S36** using a Sandmeyer reaction. **S36** was subjected to a selective Suzuki coupling to afford **S37**, followed by direct Weinreb amide formation to **S38**. This was treated with vinylmagnesium bromide to afford **S39**, which was cyclised into BCHep derivative **25**. This substrate was reduced using triethylsilane/TFA to generate building block **29b**.

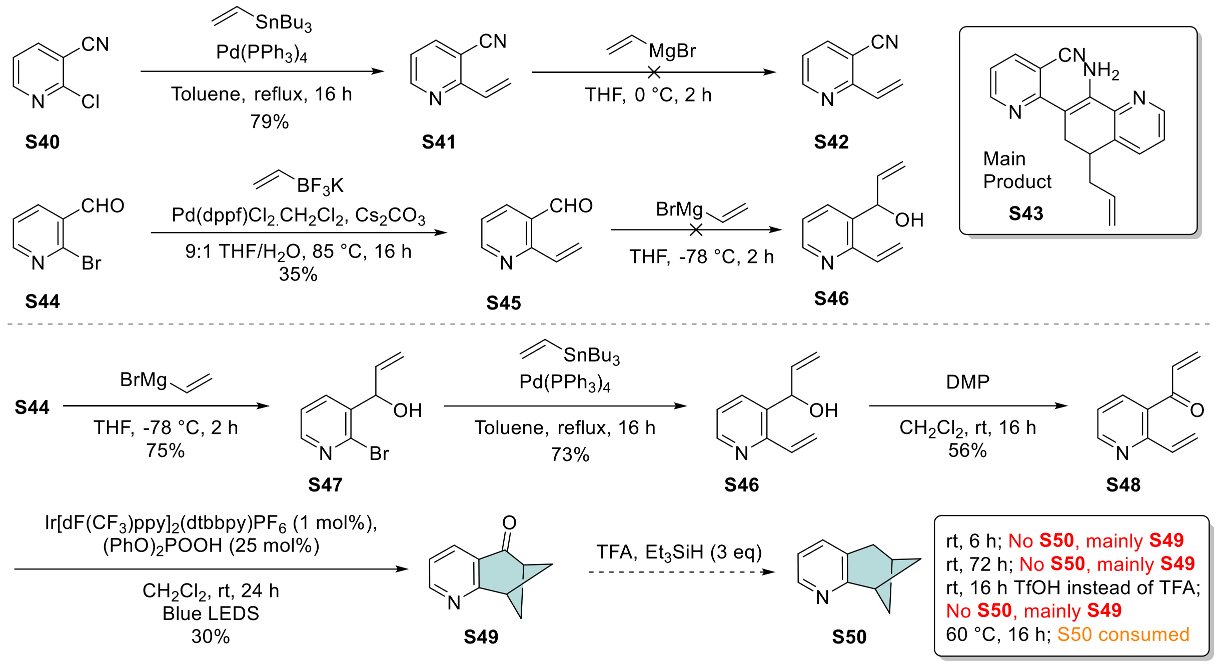

Scheme 7. Validation of pyridine substrates for cycloaddition reaction. Initial attempts involved first forming the vinyl pyridine **S41** from **S40**, however, treatment of the nitrile with vinylmagnesium bromide forms a complex biproduct **S43**, indicating that conjugate addition into the vinyl group is favoured. Therefore, a similar strategy was employed using **S44**, starting with a Suzuki reaction to generate **S45**. A complex mixture was observed when this was treated with the Grignard reagent to form **S46**, perhaps due to the high reactivity of the vinyl group. The successful route started from performing the Grignard addition first to obtain **S47**, followed by Stille coupling to **S46**. This was treated with DMP to give rise to **S48**. When this was treated with the cycloaddition conditions in the presence of catalytic acid, **S49** was obtained in moderate yields. Reduction to **S50** was unfortunately unsuccessful using various silane reduction conditions, most likely a consequence of preferential protonation of the pyridine nitrogen over the ketone. Prolonged heating at 60 °C resulted in partial reduction of the pyridine ring.

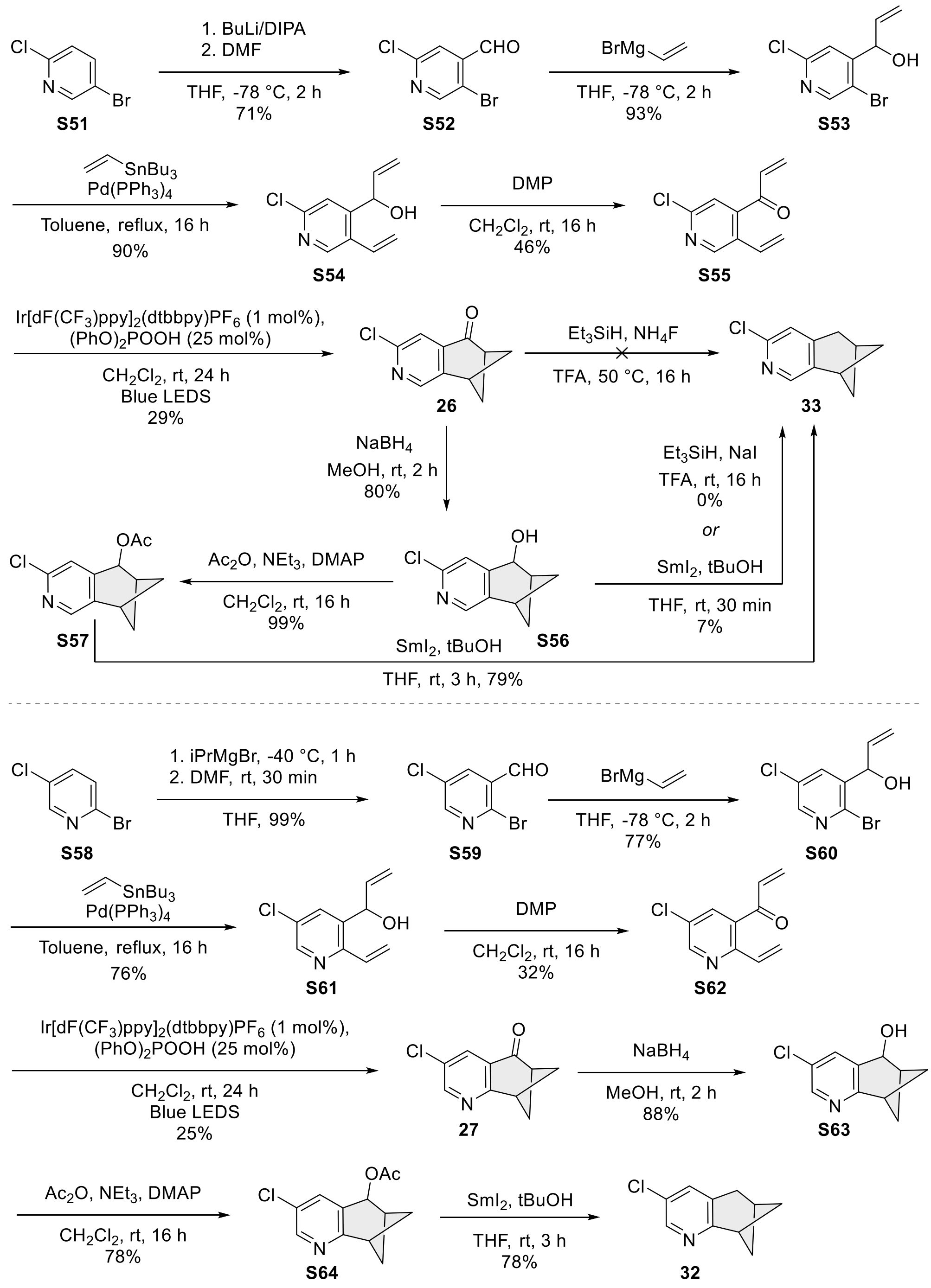

Scheme 8. The route to the substituted pyridine derivatives was analogous to **Scheme 7**. Formylation of **S51** provided **S52**, followed by Grignard addition to generate **S53**. This was subjected to Stille coupling conditions to afford **S54**, which was oxidised smoothly to **S55**. The cycloaddition provided **26**. Direct reduction to **33** was not possible, so it was necessary to proceed via alcohol **S56**. This could directly be reduced to **33** using SmI_2, [1] however, the yields could be improved when **S57** was prepared first. For the quinoline isostere, Formylation of **S58** provided **S59**, followed by Grignard addition to generate **S60**. This was subjected to Stille coupling conditions to generate **S61**, which was oxidised to **S62** using DMP. The cycloaddition afforded **27**. This was reduced to alcohol **S63**, which was converted to **S64** before reduction to provide **32** in good yields.

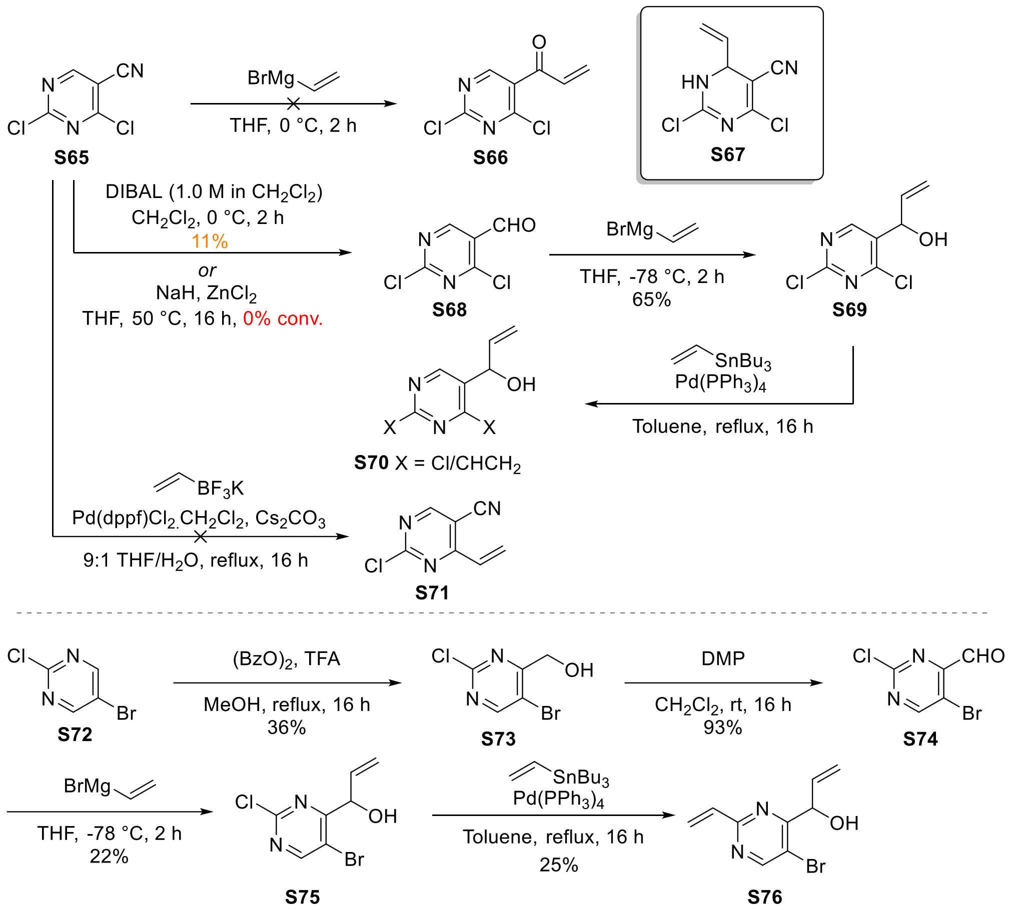

Scheme 9. Unsuccessful route to pyrimidine substrates. Grignard addition into **S65** resulted in formation of **S67** rather than the desired **S66**. Therefore, we attempted to use the more electrophilic **S68** to promote the desired Grignard addition. DIBAL reduction provided poor yields of **S68**, whilst Zinc hydrides did not reduce this system. Gratifyingly, it was possible to generate **S69**, however Stille coupling resulted in a complex mixture of **S70**-like products. A similar complex mixture was observed during the preparation of **S71**. Therefore, we envisaged preparing the alternative pyrimidine regioisomer. Minisci reaction of **S72** provided alcohol **S73**, which was subjected to DMP oxidation to **S74**. Grignard addition to **S75** proceeded smoothly, however Stille coupling provided undesired regioisomer **S76**. No other divinyl products were observed or isolated from this reaction.

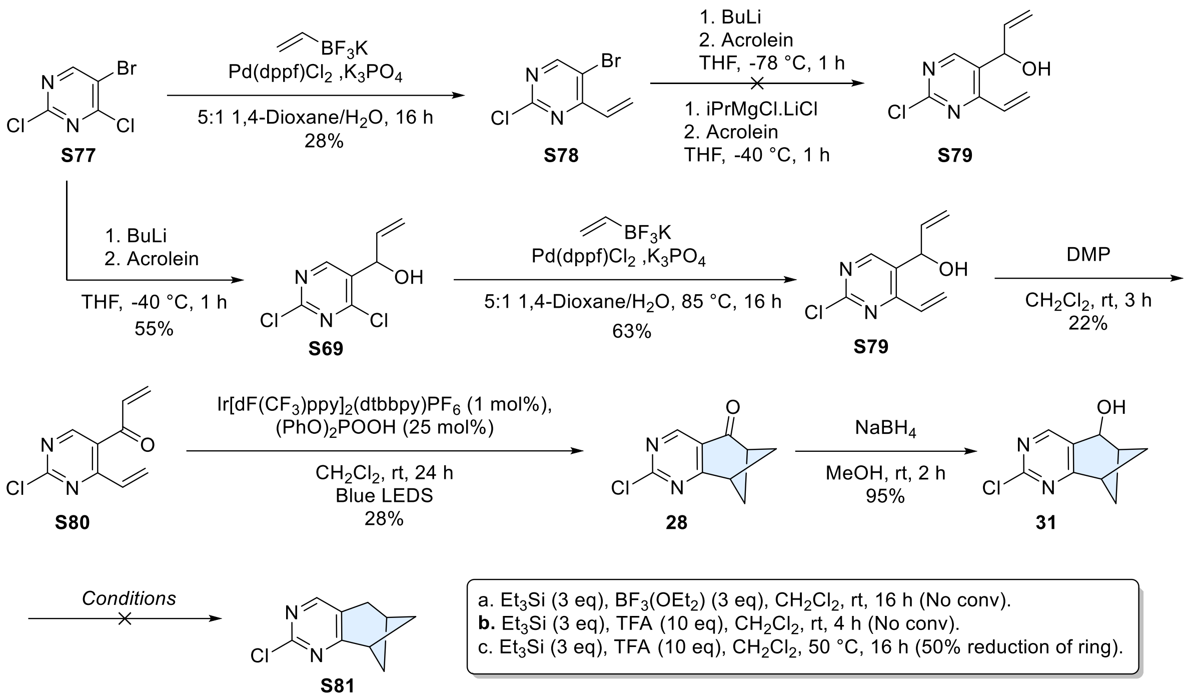

Scheme 10. Successful route to pyrimidine substrates. Commercially available **S77** was initially subjected to selective Suzuki coupling conditions to provide **S78**. Unfortunately, Metal/Halogen exchange followed by trapping with acrolein to generate **S79** was unsuccessful using BuLi or TurboGrignard. Instead, the order of steps was reversed, proceeding via **S69**. Selective Suzuki coupling was successful when the reaction was conducted at 85 °C. **S79** was oxidised to provide **S80,** which was successfully converted to the BCHep derivative **28**. Reduction to **31** proceeded smoothly. $SiEt_3H$-based reductions in the presence of $BF_3.OEt_2$ and TFA were unsuccessful in reducing this substrate to **S81**.

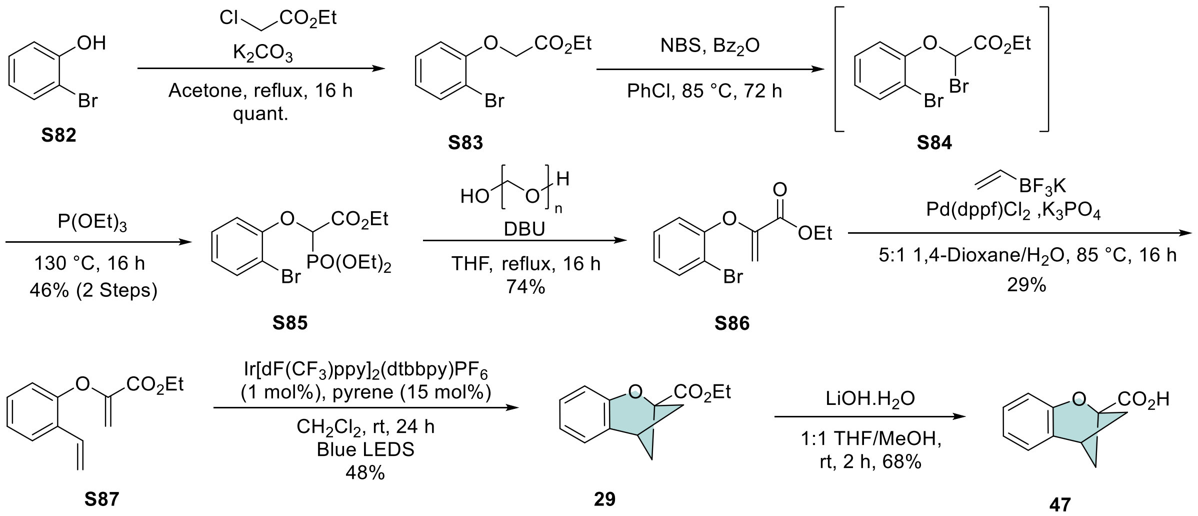

Scheme 11. Preparation of oxygenated substrate. The synthesis commenced by alkyation of **S82** with ethyl chloroacetate to provide **S83** in quantitative yield. This was treated with NBS and benzoyl peroxide to generate bromide **S84**, which was immediately used in an Arbuzov reaction to afford **S85** in good yields. This was subjected to Horner-Wadsworth-Emmons reaction with paraformaldehyde to furnish **S86**, which was converted into **S87** under Stille conditions. Finally, the diene intermediate was converted to the oxa-BCHep derivative **29** in good yield. This was hydrolysed to afford the crystalline derivative **47**.

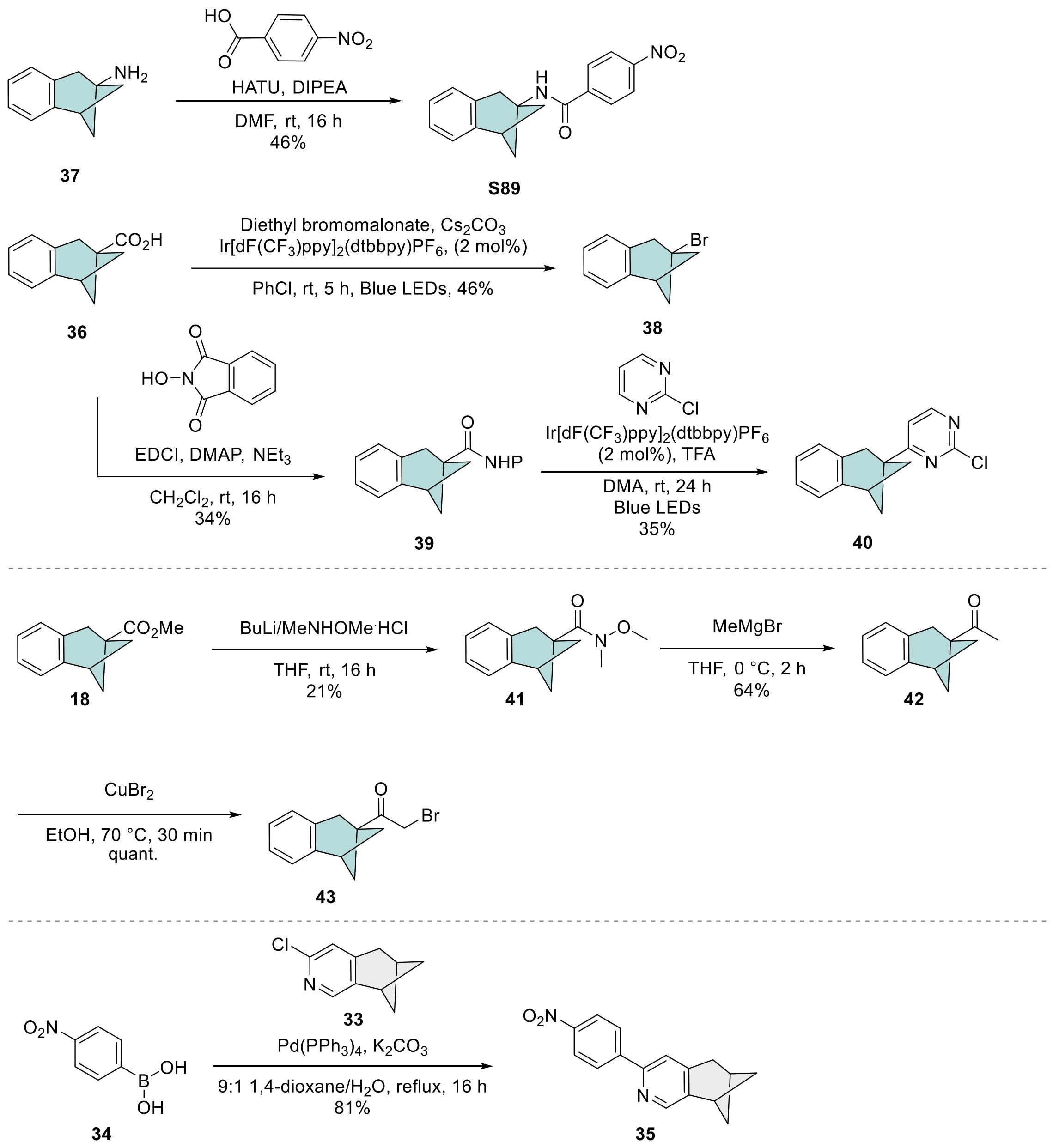

Scheme 12. Further Derivatisation of BCHep Building Blocks. It was possible to amide couple **37** to generate crystalline **S89**. **36** was smoothly converted to halide **38**. **36** also underwent esterification to generate NHP ester **39**, which could be subjected to a decarboxylative Minisci reaction to provide heterocyclic derivative **40**. Finally, ester derivative **18** was converted to the versatile Weinreb amide **41**, which was transformed into ketone **42**, which could be subjected to mild bromination conditions to furnish **43**. Finally, the pyridine derivative **33** was subjected to Suzuki coupling conditions with **34** to generate **35**. The p-nitro functionality to chosen to improve the crystallinity of the product.

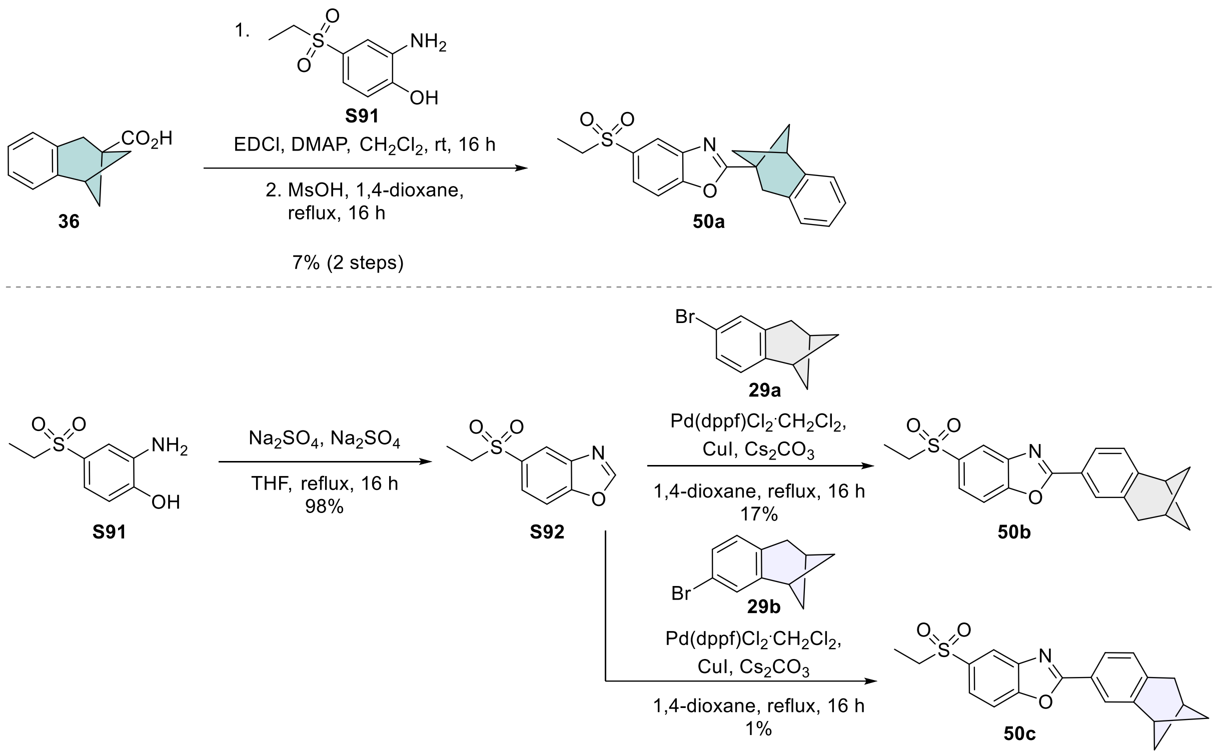

Scheme 13. Preparation of **Ezutromid** derivatives. Carboxylic acid derivative **36** was coupled to **S91** to generate the intermediate amide, which was immediately cyclised under acidic conditions to furnish the target compound **50a**. Isomers **50b** and **50c** were prepared by converting **S91** to benzoxazole **S92**, which was subjected to C-H activation conditions with **29a-b** to afford the **ezutromid** derivatives. Yields were relatively low due to extensive purification.

3. Synthetic procedures and Characterisation Data

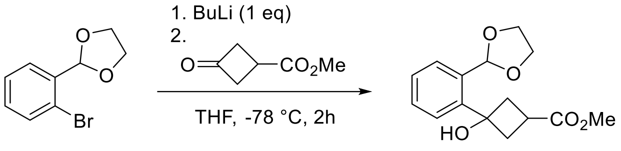

S1 **S2**

S2. 2-(2-bromophenyl)-1,3-dioxolane (5.0 g, 3.3 mL, 21.83 mmol, 1.0 eq) was dissolved in dry THF (75 mL) and cooled to -78 °C. Then n-butyllithium 2.5M in hexanes (8.73 mL, 21.83 mmol, 1.0 eq) was added dropwise at this temperature, after which the solution went from clear to orange. The mixture was stirred at this temperature for 1 hour, and was transferred dropwise by canula into a solution of methyl 3-oxocyclobutane-1-carboxylate (3.08 g, 2.52 mL, 24 mmol, 1.1 eq) in Et_2O (100 mL) at -78 °C. The mixture was stirred for 2 hours at this temperature, after which it was quenched with sat. NH_4Cl solution. The mixture was allowed to warm to room temperature and the organic layer was collected and dried with $MgSO_4$, then concentrated *in vacuo*. The residue was purified by silica gel chromatography (8:1 CH_2Cl_2:EtOAc to 6:1 CH_2Cl_2:EtOAc) to afford the title compound as a clear oil (654 mg, 2.41 mmol, 11%). ^{1}H NMR (600 MHz, $CDCl_3$) δ 7.48 – 7.33 (m, 4H), 6.21 (s, 1H), 3.79 (dtd, *J* = 7.4, 3.4, 2.1 Hz, 2H), 3.77 – 3.73 (m, 5H), 3.16 – 3.08 (m, 1H), 3.02 – 2.93 (m, 2H), 2.74 – 2.64 (m, 2H), 2.52 (br s 1H). ^{13}C NMR (151 MHz, $CDCl_3$) δ 174.80, 145.53, 136.86, 130.28, 128.48, 123.00, 119.69, 106.17, 83.29, 69.99, 62.37, 41.89, 40.24, 29.56. HRMS-ESI (m/z) Calculated for $C_{15}H_{18}O_5Na$ $[M+Na]^+$, 301.1046; found 301.1045.

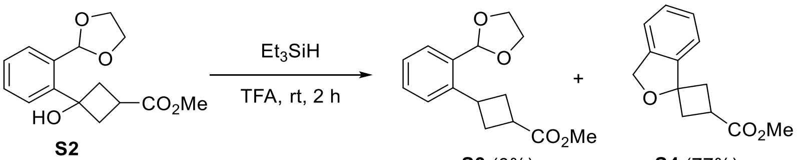

S2 **S3** (0%) **S4** (77%)

S4. Alcohol **S2** (100 mg, 0.36 mmol) was dissolved in TFA (2 mL). Then triethylsilane (344 µL, 2.16 mmol, 6 eq) was added at 0 °C. The mixture was allowed to warm to room temperature for 2 hours. The volatiles were removed under a stream of nitrogen, then the residue was dissolved in CH_2Cl_2 (15 mL). The organic layer was washed with sat. $NaHCO_3$, then concentrated. The residue was purified by silica gel chromatography (20:1 Pentane to afford the title compound as a clear oil (60.5 mg, 0.28 mmol, 77%). ^{1}H NMR (600 MHz, $CDCl_3$) δ 7.41 (dt, *J* = 7.5, 1.0 Hz, 1H), 7.38 – 7.34 (m, 1H), 7.32 (td, *J* = 7.4, 1.2 Hz, 1H), 7.22 (dp, *J* = 7.3, 1.0 Hz, 1H), 5.10 (d, *J* = 1.0 Hz, 2H), 3.76 (s, 3H), 3.10 (dq, *J* = 9.9, 8.7 Hz, 1H), 2.96 – 2.89 (m, 2H), 2.71 – 2.65 (m, 2H). ^{13}C NMR (151 MHz, $CDCl_3$) δ 175.17, 144.62, 138.82, 128.08, 127.89, 120.97, 119.75, 84.08, 72.42, 52.07, 41.65, 29.36. HRMS-ESI (m/z) Calculated for $C_{13}H_{15}O_3$ $[M+H]^+$, 219.1016; found 219.1016.

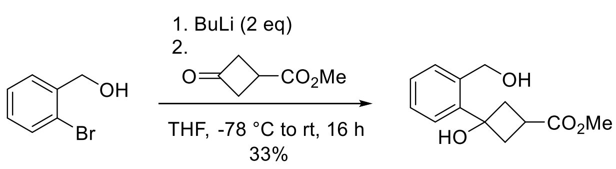

S6 **S7**

S7. (2-bromophenyl)-methanol **S6** (5.0 g, 26.7 mmol, 1.0 eq) was dissolved in dry THF (75 mL) and cooled to -78 °C. Then n-butyllithium 2.5 M in hexanes (21.4 mL, 53.5 mmol, 2.0 eq) was added dropwise at this temperature, after which the solution went from clear to an orange precipitate. The mixture was stirred at this temperature for 1 hour, and was transferred

dropwise by a thick canula into a solution of methyl 3-oxocyclobutane-1-carboxylate (3.77 g, 3.09 mL, 29.4 mmol, 1.1 eq) in THF (100 mL) at -78 °C. The mixture was allowed to warm up to room temperature for 16 hours, after which it was quenched with sat. NH_4Cl solution. The mixture was extracted with EtOAc, then the organic layer dried with $MgSO_4$ and concentrated *in vacuo*. The residue was purified by silica gel chromatography (4:1 Pentane:(3:1 EtOAc:EtOH) to afford the title compound as a clear oil (2.10 g, 8.89 mmol, 33%). ^{1}H NMR (600 MHz, $CDCl_3$) δ 7.39 – 7.29 (m, 4H), 4.65 (s, 2H), 3.76 (s, 3H), 3.74 – 3.60 (m, 2H), 3.02 – 2.96 (m, 2H), 2.91 – 2.85 (m, 1H), 2.72 – 2.67 (m, 2H). ^{13}C NMR (151 MHz, $CDCl_3$) δ 177.65, 141.94, 139.43, 131.68, 128.61, 128.14, 125.49, 76.10, 64.35, 52.49, 39.75, 31.08. HRMS-ESI (m/z) Calculated for $C_{13}H_{17}O_4$ $[M+H]^+$, 237.1121; found 237.0793.

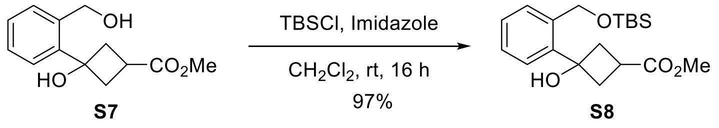

S8. Alcohol **S7** (302 mg, 1.28 mmol) was dissolved in CH_2Cl_2 (4 mL). Then, imidazole (130 mg, 1.92 mmol, 1.5 eq) and TBSCl (193 mg, 1.28 mmol, 1.0 eq) were added and the reaction was stirred at room temperature for 16 hours. The mixture was diluted in CH_2Cl_2, then filtered. The organic layer was washed with brine, dried and concentrated to afford the title compound as a clear oil which was left without further purification (433 mg, 1.24 mmol, 97%). ^{1}H NMR (400 MHz, $CDCl_3$) δ 7.43 – 7.39 (m, 1H), 7.38 – 7.33 (m, 1H), 7.33 – 7.28 (m, 2H), 4.81 (s, 2H), 3.73 (s, 3H), 2.97 – 2.86 (m, 2H), 2.80– 2.71 (m, 3H), 0.91 (s, 9H), 0.12 (s, 6H). ^{13}C NMR (151 MHz, $CDCl_3$) δ 175.98, 142.47, 138.45, 130.71, 128.15, 128.10, 125.40, 74.61, 64.98, 52.10, 39.66, 30.48, 25.98, 18.38, -5.07. HRMS-ESI (m/z) Calculated for $C_{19}H_{31}O_4Si$ $[M+H]^+$, 351.1986; found 351.1991.

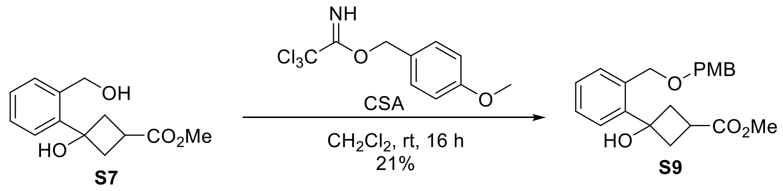

S9. To a solution of **S7** (150 mg, 634.9 µmol) in CH_2Cl_2 (5 mL) was added p-methoxybenzyl trichloroacetimidate (215 mg, 761.9 µmol, 1.2 eq) and camphorsulfonic acid (11.8 mg, 51 µmol, 8 mol%). The mixture was stirred at room temperature for 16 hours, after which it was diluted in CH_2Cl_2. The organic layer wash washed with sat. $NaHCO_3$, then back-extracted with CH_2Cl_2. The organic layer was concentrated, then purified by silica gel chromatography (6:1 Pentane:EtOAc) to afford the title compound as a clear oil (48.4 mg, 135 µmol, 21%). ^{1}H NMR (700 MHz, $CDCl_3$) δ 7.42 (dd, J = 7.7, 1.4 Hz, 1H), 7.37 – 7.33 (m, 2H), 7.30 (dd, J = 7.4, 1.4 Hz, 1H), 7.29 – 7.26 (m, 2H), 6.90 – 6.87 (m, 2H), 4.72 (d, J = 13.4 Hz, 1H), 4.63 (s, 2H), 4.51 (s, 2H), 3.80 (s, 3H), 3.74 (s, 3H), 2.95 – 2.88 (m, 2H), 2.78 – 2.71 (m, 3H). ^{13}C NMR (176 MHz, $CDCl_3$) δ 175.99, 159.64, 143.31, 135.61, 132.06, 130.00, 129.27, 128.65, 128.06, 125.67, 114.09, 74.64, 72.46, 71.30, 55.44, 52.13, 39.56, 30.55. HRMS-ESI (m/z) Calculated for $C_{21}H_{24}O_5Na$ $[M+Na]^+$, 379.1516; found 379.1510.

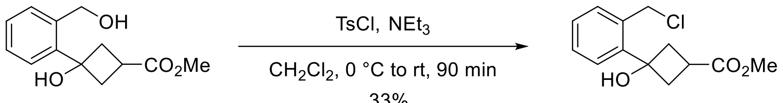

S7 S10

S10. Alcohol **S7** (235 mg, 0.95 mmol) was dissolved in CH_2Cl_2 (5 mL). Then, triethylamine (146 µL, 1.04 mmol, 1.05 eq) and TsCl (199 mg, 1.04 mmol, 1.05) eq were added at 0 °C, and the solution was allowed to warm to room temperature for 90 minutes. The solution with diluted with CH_2Cl_2, then washed with brine. The organic layer was dried with $MgSO_4$, then concentrated. The residue was purified by The residue was purified by silica gel chromatography (6% EtOAc in pentane) to afford the title compound as a clear oil (main product). ^{1}H NMR (600 MHz, $CDCl_3$) δ 7.52 (dt, *J* = 7.2, 1.0 Hz, 1H), 7.36 – 7.30 (m, 3H), 4.82 (s, 2H), 3.77 (s, 3H), 3.69 (d, *J* = 12.8 Hz, 1H), 3.15 – 3.09 (m, 2H), 2.95 – 2.89 (m, 1H), 2.71 – 2.65 (m, 2H). ^{13}C NMR (151 MHz, $CDCl_3$) δ 177.74, 141.77, 136.80, 132.49, 128.81, 128.42, 125.48, 75.58, 52.48, 43.84, 40.21, 31.29. HRMS-ESI (m/z) Calculated for $C_{13}H_{15}O_3ClNa$ $[M+Na]^+$, 277.0602; found 277.0601.

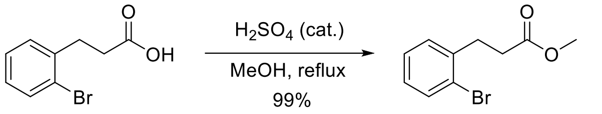

S13 S14

S14. To a solution of **S13** (5.30 g, 23.1 mmol) in MeOH (110 mL) was added 15 drops of H_2SO_4 The reaction was stirred at reflux temperature for 16 hours, after which the reaction was cooled. Saturated $NaHCO_3$ was added, and the mixture was extracted with Et_2O (3x 100 mL). The combined organic layers were dried with $MgSO_4$, and concentrated to afford the title compound as a clear oil (5.58 g, 23 mmol, 99%). ^{1}H NMR (400 MHz, $CDCl_3$) δ 7.58 – 7.52 (m, 1H), 7.30 – 7.21 (m, 2H), 7.10 (ddd, *J* = 7.9, 6.5, 2.6 Hz, 1H), 3.70 (s, 3H), 3.09 (dd, *J* = 8.5, 7.2 Hz, 2H), 2.68 (dd, *J* = 8.5, 7.2 Hz, 2H). Data consistent with that given in the literature.[2]

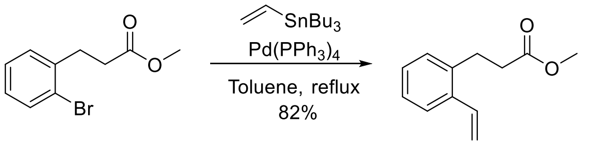

S14 S15

S15. Bromide **S14** (2 g, 8.2 mmol) was dissolved in toluene (50 mL) and degassed with nitrogen for 5 minutes. Then, vinyltributyltin (2.65 mL, 9.1 mmol, 1.1 eq) was added and the solution was degassed for a further 5 minutes. Finally, $Pd(PPh_3)_4$ (475 mg, 0.4 mmol, 5 mol%) was added and the mixture was degassed for a further 5 minutes, then heated at reflux temperature for 16 hours. The reaction mixture was cooled, filtered over celite, then concentrated. The residue was purified by silica gel chromatography with 10% crushed KF (0 to 3% EtOAc in pentane) to afford the title compound as a clear oil (1.29 g, 6.8 mmol, 82%). ^{1}H NMR (400 MHz, $CDCl_3$) δ 7.52 – 7.45 (m, 1H), 7.24 – 7.14 (m, 3H), 6.98 (dd, *J* = 17.3, 11.0 Hz, 1H), 5.66 (dd, *J* = 17.4, 1.4 Hz, 1H), 5.33 (dd, *J* = 10.9, 1.4 Hz, 1H), 3.06 – 2.98 (m, 2H), 2.61 – 2.54 (m, 2H). ^{13}C NMR (101 MHz, $CDCl_3$) δ 173.42, 137.87, 136.71, 134.30, 129.42, 128.08, 126.93, 126.13, 116.25, 51.76, 35.26, 28.56. HRMS-ESI (m/z) Calculated for $C_{12}H_{14}O_2Na$ $[M+Na]^+$, 213.0886; found 213.0887.

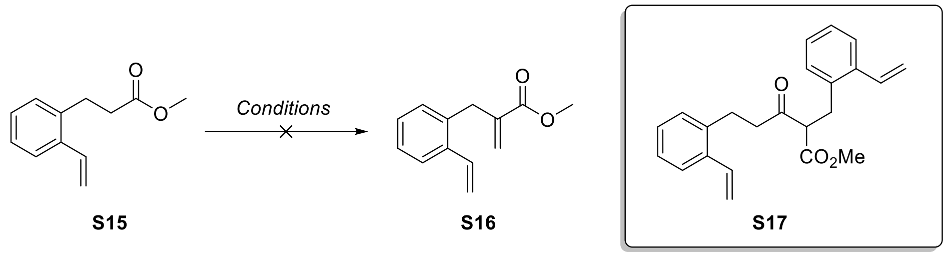

S17. ^{1}H NMR (400 MHz, $CDCl_3$) δ 7.41 – 7.35 (m, 2H), 7.17 – 7.05 (m, 4H), 7.03 – 6.94 (m, 2H), 6.85 (dd, *J* = 17.3, 10.9 Hz, 1H), 6.72 (dd, *J* = 17.3, 11.0 Hz, 1H), 5.56 (td, *J* = 17.3, 1.4 Hz, 2H), 5.23 (ddd, *J* = 25.0, 11.0, 1.4 Hz, 2H), 3.69 (t, *J* = 7.5 Hz, 1H), 3.56 (s, 3H), 3.16 (d, *J* = 7.6 Hz, 2H), 2.85 – 2.60 (m, 3H), 2.44 – 2.33 (m, 1H).^{13}C NMR (101 MHz, $CDCl_3$) δ 204.09, 169.53, 137.92, 136.87, 136.58, 135.28, 134.27, 134.17, 130.32, 129.55, 128.08, 128.06, 127.41, 126.84, 126.37, 126.07, 116.77, 116.17, 59.13, 52.60, 44.38, 31.63, 26.94.

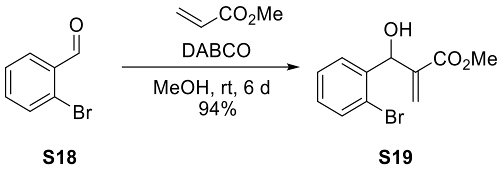

S19. 2-bromobenzaldehyde **S18** (4.5 g, 2.85 mL, 24.3 mmol) was dissolved in MeOH (10 mL), then DABCO (2.73 g, 24.3 mmol, 1.0 eq) and methyl acrylate (3.14 g, 3.31 mL, 36.5 mmol, 1.5 eq) was added the reaction was allowed to stir at room temperature for 6 days. Then, ammonium chloride was added and the mixture was extracted with EtOAc. The organic layer was dried with $MgSO_4$ and concentrated to afford the title compound as a yellow oil (6.5 g, 24.0 mmol, 94%). ^{1}H NMR (400 MHz, $CDCl_3$) δ 7.57 – 7.53 (dt, *J* = 7.9, 1.4 Hz, 2H), 7.35 (td, *J* = 7.6, 1.3 Hz, 1H), 7.17 (ddd, *J* = 8.0, 7.3, 1.7 Hz, 1H), 6.35 (t, *J* = 0.8 Hz, 1H), 5.94 (dd, *J* = 4.8, 1.2 Hz, 1H), 5.56 (q, *J* = 1.3 Hz, 1H), 3.79 (s, 3H), 3.22 (d, *J* = 4.5 Hz, 1H). Data consistent with that given in the literature. [3]

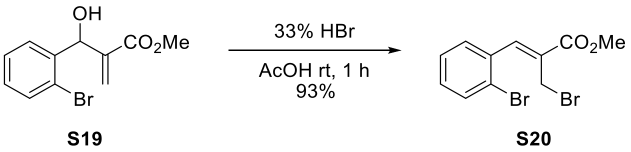

S20. Baylis hillman adduct **S19** (6.5 g, 24 mmol) was treated with HBr (33% in AcOH) (13 mL, 71.9 mmol, 3 eq) and stirred at room temperature for 1 hour. Then a 1:1 mixture of distilled water water and CH_2Cl_2 were added (100 mL). The mixture was extracted, then the organic layer was dried with $MgSO_4$ and concentrated to afford the title compound as a yellow oil (7.8 g, 22.3 mmol, 93%). ^{1}H NMR (400 MHz, $CDCl_3$) δ 7.86 (d, *J* = 0.7 Hz, 1H), 7.69 (dd, *J* = 7.8, 1.5 Hz, 1H), 7.65 (dd, *J* = 8.0, 1.2 Hz, 1H), 7.43 (ddd, *J* = 7.6, 7.6, 1.1 Hz, 1H), 7.26 (ddd, *J* = 7.6, 7.6, 1.5 Hz, 1H), 4.25 (s, 2H), 3.91 (s, 3H). Data consistent with that given in the literature. [4]

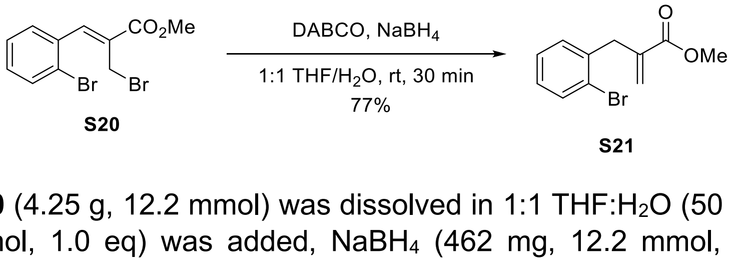

S21. Bromide **S20** (4.25 g, 12.2 mmol) was dissolved in 1:1 THF:H_2O (50 mL) then DABCO (1.37 g, 12.2 mmol, 1.0 eq) was added, $NaBH_4$ (462 mg, 12.2 mmol, 1 eq) was added

portionwise at room temperature and the reaction was allowed to stir at room temperature for 30 minutes. Then, ammonium chloride was added and the mixture was extracted with EtOAc. The organic layer was dried with $MgSO_4$ and concentrated. The residue was purified by silica gel chromatography with (0 to 2% EtOAc in pentane) to afford the title compound as a clear oil (2.41 g, 9.5 mmol, 77%). ^{1}H NMR (400 MHz, $CDCl_3$) δ 7.56 (dd, *J* = 8.0, 1.2 Hz, 1H), 7.26 (td, *J* = 7.1, 1.3 Hz, 1H), 7.24 – 7.20 (m, 1H), 7.10 (ddd, *J* = 7.9, 6.9, 2.2 Hz, 1H), 6.28 (q, *J* = 1.2 Hz, 1H), 5.32 (td, *J* = 1.7, 1.1 Hz, 1H), 3.78 (s, 3H), 3.77 (t, *J* = 1.5 Hz, 2H). Data consistent with that given in the literature. [5]

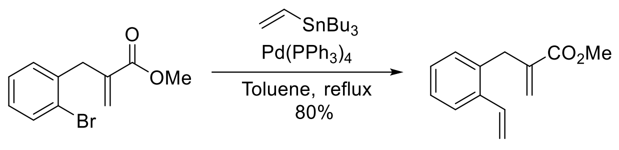

S21 **S16**

S16. Bromide **S21** (500 mg, 1.96 mmol) was dissolved in toluene (10 mL) and degassed with nitrogen for 5 minutes. Then, vinyltributyltin (0.63 mL, 2.2 mmol, 1.1 eq) was added and the solution was degassed for a further 5 minutes. Finally, $Pd(PPh_3)_4$ (113 mg, 0.1 mmol, 5 mol%) was added and the mixture was degassed for a further 5 minutes, then heated at reflux temperature for 16 hours. The reaction mixture was cooled, filtered over celite, then concentrated. The residue was purified by silica gel chromatography with 10% crushed KF (1.5 to 2.5% EtOAc in pentane) to afford the title compound as a clear oil (320 mg, 1.58 mmol, 80%).^{1}H NMR (400 MHz, $CDCl_3$) δ 7.56 – 7.50 (m, 1H), 7.27 – 7.21 (m, 2H), 7.16 – 7.10 (m, 1H), 6.85 (dd, *J* = 17.4, 11.1 Hz, 1H), 6.23 (q, *J* = 1.4 Hz, 1H), 5.64 (dd, *J* = 17.4, 1.4 Hz, 1H), 5.27 (dd, *J* = 10.9, 1.4 Hz, 1H), 5.17 (td, *J* = 1.9, 1.1 Hz, 1H), 3.79 (s, 3H), 3.70 (t, *J* = 1.7 Hz, 2H). ^{13}C NMR (101 MHz, $CDCl_3$) δ 167.61, 139.45, 137.29, 135.67, 134.54, 130.64, 128.05, 127.16, 126.58, 125.97, 115.94, 52.10, 35.09. HRMS-ESI (m/z) Calculated for $C_{13}H_{14}O_2Na$ $[M+Na]^+$, 225.0886; found 225.0885.

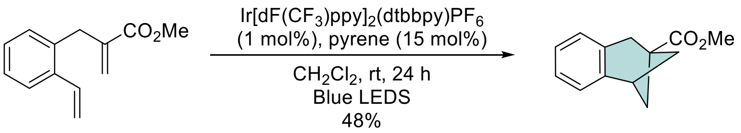

S16 **18**

18. Diene **S16** (100 mg, 494 μmol) in CH_2Cl_2 (30 mL) was added to a vial charged with $Ir[dF(CF_3)ppy]_2(dtbbpy)PF_6$ (9 mg, 1 mol%) and pyrene (15 mg, 74.2 μmol, 15 mol %). under a N_2 atmosphere. The solution was stirred vigorously while irradiating with a blue LED lamp (440 nm) at a distance of 2 cm, at 25 °C using a stream of nitrogen to cool the flask. After 24 hours, the reaction was concentrated and purified by silica gel chromatography (10 to 25% CH_2Cl_2 in pentane) to afford the title compound as a clear oil (48 mg, 237 μmol, 48%). ^{1}H NMR (400 MHz, $CDCl_3$) δ 7.21 (ddt, *J* = 7.4, 1.6, 0.7 Hz, 1H), 7.17 (td, *J* = 7.2, 1.5 Hz, 1H), 7.09 (tdt, *J* = 6.9, 1.6, 0.8 Hz, 1H), 7.03 (dd, *J* = 7.4, 1.5 Hz, 1H), 3.75 (s, 3H), 3.26 (s, 2H), 3.04 (t, *J* = 5.8 Hz, 1H), 2.68 (td, *J* = 6.0, 2.6 Hz, 2H), 1.74 – 1.67 (m, 2H). ^{13}C NMR (101 MHz, $CDCl_3$) δ 175.54, 146.27, 133.37, 128.71, 126.49, 125.52, 125.22, 51.98, 46.05, 37.41, 36.12, 35.88. HRMS-ESI (m/z) Calculated for $C_{13}H_{14}O_2Na$ $[M+Na]^+$, 225.0886; found 225.0887.

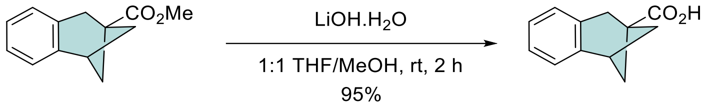

18 **36**

36. Ester **18** (42 mg, 208 μmol) was dissolved in 1:1 THF/MeOH (2 mL). Lithium hydroxide monohydrate (19.2 mg, 458 μmol, 2.5 eq) in water (1 mL) was added and the mixture was stirred for 2 hours. The mixture was diluted in $CHCl_3$ (15 mL), and the mixture was acidified with dilute HCl until pH ~ 3. The organic layer was dried with $MgSO_4$ and concentrated to afford the title compound as a white solid (40 mg, 208 μmol, 100%). ^{1}H NMR (400 MHz, $CDCl_3$) δ 7.24 – 7.21 (m, 1H), 7.18 (td, *J* = 7.2, 1.5 Hz, 1H), 7.12 – 7.07 (m, 1H), 7.05 (dd, *J* = 7.4, 1.5 Hz, 1H), 3.30 (s, 2H), 3.07 (t, *J* = 5.8 Hz, 1H), 2.73 (td, *J* = 6.0, 2.5 Hz, 2H), 1.77 – 1.71 (m, 2H). ^{13}C NMR (151 MHz, $CDCl_3$) δ 179.74, 146.05, 133.13, 128.75, 126.60, 125.63, 125.30, 45.76, 37.34, 35.91, 35.78. HRMS-ESI (m/z) Calculated for $C_{12}H_{12}O_2Na$ $[M+Na]^+$, 211.0730; found 211.0939.

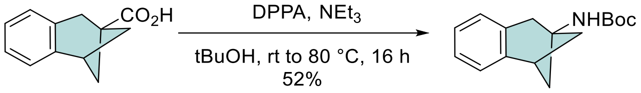

36 **S22**

S22. Acid **36** (20 mg, 106 μmol) was dissolved in tBuOH (0.2 mL). Then triethylamine (19.4 μL, 139.5 μmol. 1.05 eq) and DPPA (30 μL, 139.5 μmol. 1.05 eq) were added. The mixture was stirred for 30 minutes at room temperature, after which it was stirred at reflux temperature for 16 hours. The reaction was cooled, then diluted with EtOAc, then washed with saturated bicarbonate solution. The organic layer was dried with $MgSO_4$ and concentrated. The residue was purified by silica gel chromatography with (40% CH_2Cl_2 in pentane) to afford the title compound as a white solid (18 mg, 69 μmol, 52%). ^{1}H NMR (600 MHz, $CDCl_3$) δ 7.21 – 7.18 (m, 1H), 7.16 (td, *J* = 7.3, 1.5 Hz, 1H), 7.11 – 7.07 (m, 1H), 7.05 (dd, *J* = 7.4, 1.4 Hz, 1H), 4.84 (s, 1H), 3.21 (s, 2H), 3.08 (t, *J* = 6.3 Hz, 1H), 2.68 (br s, 2H), 1.86 – 1.80 (m, 2H), 1.49 (s, 9H). ^{13}C NMR (151 MHz, $CDCl_3$) δ 154.69, 146.24, 128.47, 126.04, 125.44, 124.97, 79.52, 54.77, 40.25, 40.25, 34.49, 28.62. HRMS-ESI (m/z) Calculated for $C_{16}H_{21}NO_2Na$ $[M+Na]^+$, 282.1465; found 282.1460.

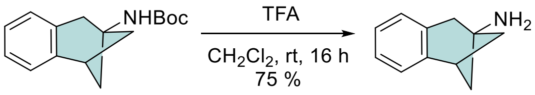

S22 **37**

37. Boc-protected amine **S22** (5 mg, 19.3 μmol) was dissolved in CH_2Cl_2 (0.3 mL). Then TFA (22 μL, 289.2 μmol, 15 eq) was added and the mixture was stirred at room temperature for 16 hours. The mixture was diluted in CH_2Cl_2, then washed with saturated bicarbonate solution. The organic layer was dried with $MgSO_4$ and concentrated to afford the title compound as a clear oil (2.3 mg, 14.4 μmol, 75%). ^{1}H NMR (600 MHz, $CDCl_3$) δ 7.17 (dd, *J* = 7.5, 1.5 Hz, 1H), 7.14 (td, *J* = 7.2, 1.5 Hz, 1H), 7.07 (td, *J* = 7.2, 1.6 Hz, 1H), 7.03 (dd, *J* = 7.3, 1.4 Hz, 1H), 4.67 (br s, 2H), 3.25 (s, 2H), 3.10 (t, *J* = 6.3 Hz, 1H), 2.61 (td, *J* = 6.2, 2.5 Hz, 2H), 1.91 – 1.84 (m, 2H). ^{13}C NMR (151 MHz, $CDCl_3$) δ 146.02, 134.67, 128.49, 126.12, 125.50, 125.02, 55.06, 40.79, 40.53, 34.91. HRMS-ESI (m/z) Calculated for $C_{11}H_{14}N$ $[M+H]^+$, 160.1121; found 160.1120.

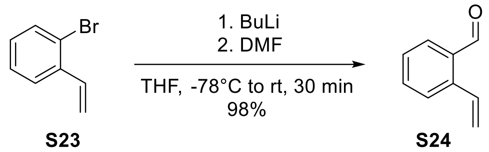

S24. Bromide **S23** (3.4 g, 18.6 mmol) was dissolved in dry THF (20 mL) under N_2 and cooled to -78 °C.Then, n-butyllithium 2.5M in hexanes (7.8 mL, 19.5 mmol, 1.05 eq) was added dropwise to the solution and the mixture was stirred for 1 hour. DMF (2.9 mL, 37.15 mmol, 2.0 eq) was added, and the mixture was allowed to warm up to room temperature for 1 hour. The mixture was poured into water, extracted with EtOAc (2 x 20 mL). The organic layer was dried with $MgSO_4$ and concentrated to afford the title compound as a yellow oil (2.4 g, 18.2 mmol, 98%). ^{1}H NMR (400 MHz, $CDCl_3$) δ 10.30 (s, 1H), 7.83 (dt, *J* = 7.6, 1.1 Hz, 1H), 7.57 (dd, *J* = 3.9, 1.3 Hz, 2H), 7.56 – 7.49 (m, 1H), 7.44 (ddd, *J* = 7.6, 5.4, 3.1 Hz, 1H), 5.70 (dd, *J* = 17.4, 1.2 Hz, 1H), 5.52 (dd, *J* = 11.0, 1.2 Hz, 1H). [6]

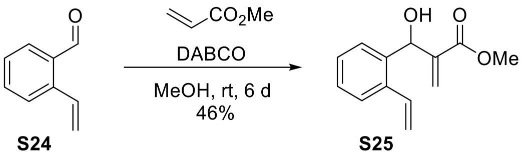

S25. 2-vinylbenzaldehyde **S24** (2.4 g, 18.2 mmol) was dissolved in MeOH (5 mL), then DABCO (2.04 g, 18.2 mmol, 1.0 eq) and methyl acrylate (2.35 g, 2.47 mL, 27.2 mmol, 1.5 eq) was added the reaction was allowed to stir at room temperature for 6 days. Then, ammonium chloride was added and the mixture was extracted with EtOAc. The organic layer was dried with $MgSO_4$ and concentrated. The residue was purified by silica gel chromatography with (10% EtOAc in pentane) to afford the title compound as a clear oil (1.94 g, 8.35 mmol, 46%). ^{1}H NMR (400 MHz, $CDCl_3$) δ 7.52 – 7.48 (m, 1H), 7.47 – 7.43 (m, 1H), 7.34 – 7.27 (m, 2H), 7.00 (dd, *J* = 17.3, 11.0 Hz, 1H), 6.34 (t, *J* = 1.0 Hz, 1H), 5.91 (s, 1H), 5.63 (dd, *J* = 17.3, 1.4 Hz, 1H), 5.60 (t, *J* = 1.3 Hz, 1H), 5.32 (dd, *J* = 11.0, 1.4 Hz, 1H), 3.77 (s, 3H), 2.91 (d, *J* = 3.8 Hz, 1H). Data consistent with that given in the literature. [3]

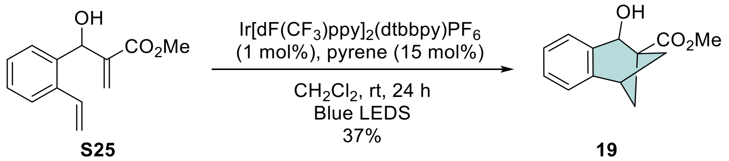

19. Diene **S25** (49.6 mg, 227 µmol) in CH_2Cl_2 (15 mL) was added to a vial charged with $Ir[dF(CF_3)ppy]_2(dtbbpy)PF_6$ (2.5 mg, 2.27 µmol, 1 mol%) and pyrene (7 mg, 34.1 µmol, 15 mol%) under a N_2 atmosphere. The solution was stirred vigorously while irradiating with a blue LED lamp (440 nm) at a distance of 2 cm, at 25 °C using a stream of nitrogen to cool the flask. After 24 hours, the reaction was concentrated and purified by silica gel chromatography (50% CH_2Cl_2 in pentane) to afford the title compound as a yellow oil (18.3 mg, 84.0 µmol, 37%). ^{1}H NMR (600 MHz, $CDCl_3$) δ 7.54 (ddd, *J* = 7.5, 1.4, 0.7 Hz, 1H), 7.27 (dd, *J* = 7.5, 1.3 Hz, 2H), 7.17 (tdd, *J* = 7.5, 1.3, 0.5 Hz, 1H), 7.04 (dd, *J* = 7.4, 1.3 Hz, 1H), 5.35 (s, 1H), 3.80 (s, 3H), 3.07 (t, *J* = 5.8 Hz, 1H), 2.76 – 2.67 (m, 2H), 1.99 (dd, *J* = 9.3, 7.7 Hz, 1H), 1.82 (dd, *J* = 9.2, 7.7 Hz, 1H). ^{13}C NMR (151 MHz, $CDCl_3$) δ 174.69, 146.41, 135.22, 129.09, 127.57, 124.67, 71.93, 52.27, 51.00, 41.05, 36.44, 34.05. HRMS-ESI (m/z). Calculated for $C_{13}H_{15}O_3$ $[M+H]^+$, 219.1016; found 219.1014.

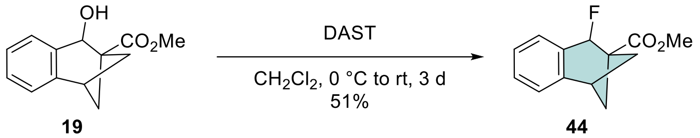

44. To a solution of **19** (18.5 mg, 84.8 µmol) in CH_2Cl_2 (2 mL), DAST (22.4 mL, 170 µmol, 2 eq) was added at 0 ºC. After 1 hour, the reaction mixture was left to warm to room temperature and stirred for 3 days. After this time, the reaction mixture was quenched with saturated aqueous $NaHCO_3$ at 0ºC. The aqueous layer was extracted with CH_2Cl_2 and the combined organic layers were dried with $MgSO_4$ and concentrated. The product was purified by preparative thin layer chromatography (5% EtOAc in pentane) to yield the desired product (9.6 mg, 51%). ^{1}H NMR (600 MHz, $CDCl_3$) δ 7.55 – 7.50 (m, 1H), 7.29 (tt, *J* = 7.5, 1.1 Hz, 1H), 7.24 (ddd, *J* = 7.5, 5.7, 1.6 Hz, 1H), 7.09 (dt, *J* = 7.4, 1.4 Hz, 1H), 6.14 (d, *J* = 53.4 Hz, 1H), 3.80 (s, 3H), 3.08 (t, *J* = 5.8 Hz, 1H), 2.84 (ddd, *J* = 9.6, 6.0, 0.9 Hz, 1H), 2.68 (ddd, *J* = 9.3, 5.7, 2.3 Hz, 1H), 2.05 (dd, *J* = 9.5, 7.7 Hz, 1H), 1.94 (ddd, *J* = 9.3, 7.7, 2.4 Hz, 1H). ^{19}F NMR (565 MHz, $CDCl_3$) δ -167.51 (d, *J* = 53.2 Hz). ^{13}C NMR (151 MHz, $CDCl_3$) δ 172.97, 147.00 (d, *J* = 4.0 Hz), 131.41 (d, *J* = 17.4 Hz), 129.57 (d, *J* = 2.7 Hz), 128.79 (d, *J* = 3.5 Hz), 127.14 (d, *J* = 3.1 Hz), 124.83, 91.35 (d, *J* = 177.0 Hz), 52.32, 50.46 (d, *J* = 20.0 Hz), 43.23 (d, *J* = 5.4 Hz), 35.98 (d, *J* = 2.7 Hz), 34.24. HRMS-ESI (m/z). Calculated for $C_{13}H_{13}FO_2Na$ $[M+Na]^+$, 243.0792; found 243.0788.

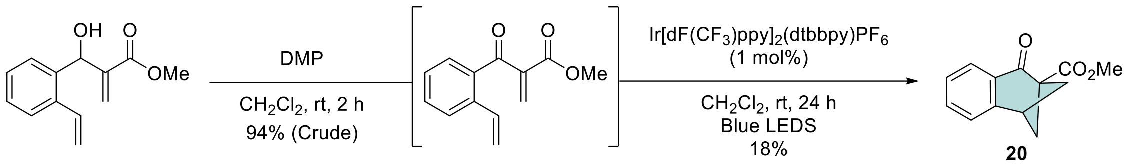

20. To a solution of **S25** (366 mg, 1.68 mmol, 1 eq) in CH_2Cl_2 (10 mL), DMP (890 mg, 2.10 mmol, 1.25 eq) was added, and the reaction mixture stirred at room temperature for 2 hours. The reaction mixture was diluted with CH_2Cl_2, quenched with saturated aqueous $Na_2S_2O_3$. The organic layer was dried with Na_2SO_4, then concentrated and left without further purification to afford the desired product as a yellow oil (340 mg, 1.57 mmol, 94%). Compound unstable to chromatography. ^{1}H NMR (400 MHz, $CDCl_3$) δ 7.53 (dd, *J* = 8.1, 3.7 Hz, 1H), 7.43 – 7.35 (m, 2H), 7.23 (t, *J* = 7.6 Hz, 1H), 7.05 – 6.97 (m, 1H), 6.58 (d, *J* = 3.3 Hz, 1H), 6.06 (d, *J* = 3.4 Hz, 1H), 5.66 – 5.57 (m, 1H), 5.31 – 5.24 (m, 1H), 3.68 (d, *J* = 3.9 Hz, 3H). **S26** (340 mg, 1.57 mmol) was dissolved in CH_2Cl_2 (60 mL) and degassed with N_2 for 10 minutes. Ir[dF(CF3)ppy]$_2$(dtbbpy)PF_6 (17.6 mg, 15.7 µmol, 1 mol%) was added to the reaction mixture, which was degassed for a further 10 minutes. The solution was stirred vigorously while irradiating with a blue LED lamp (440 nm) at a distance of 2 cm at 25 ºC, using a stream of nitrogen to cool the flask. After 16 hours, the reaction was concentrated and purified by silica gel chromatography (5% EtOAc in pentane) to afford the product as a colourless oil (59.8 mg, 0.283 mmol,18%). ^{1}H NMR (600 MHz, $CDCl_3$) δ 8.00 (dd, *J* = 7.7, 1.3 Hz, 1H), 7.46 (td, *J* = 7.5, 1.4 Hz, 1H), 7.36 (td, *J* = 7.6, 1.2 Hz, 1H), 7.24 (dd, *J* = 7.5, 1.1 Hz, 1H), 3.81 (s, 3H), 3.24 (t, *J* = 6.0 Hz, 1H), 3.05 (td, *J* = 6.3, 2.6 Hz, 2H), 2.67 – 2.61 (m, 2H). ^{13}C NMR (151 MHz, $CDCl_3$) δ 196.12, 171.37, 151.36, 133.86, 128.03, 127.49, 125.06, 59.85, 52.26, 46.62, 35.20. HRMS-ESI (m/z). Calculated for $C_{13}H_{13}O_3$ $[M+H]^+$, 217.0859; found 217.0858.

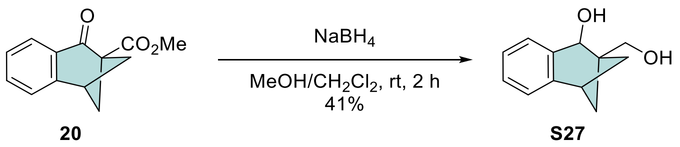

S27. To a solution of **20** (29.9 mg, 138 μmol, 1 eq) in 2:1 MeOH/CH_2Cl_2 (4.5 mL), $NaBH_4$ (26.2 mg, 691 μmol, 5 eq) was added, and the reaction mixture stirred at room temperature for 5 hours. Saturated aqueous NH_4Cl (5 mL) was added, and the aqueous layer extracted with EtOAc. The combined organic layers were washed with brine, dried with $MgSO_4$ and the solvent removed in vacuo. The crude product was purified by silica gel chromatography (0 to 50% EtOAc in CH_2Cl_2) to yield **19** (2 mg, 9.7 μmol, 7%) and **S27** (10.7 mg, 57 μmol, 41%). **S27**: ^{1}H NMR (600 MHz, $CDCl_3$) δ 7.49 (d, *J* = 7.5 Hz, 1H), 7.27 – 7.23 (m, 1H), 7.16 (td, *J* = 7.5, 1.3 Hz, 1H), 7.05 (dd, *J* = 7.4, 1.3 Hz, 1H), 5.04 (s, 1H), 3.85 (d, *J* = 11.1 Hz, 1H), 3.77 (d, *J* = 11.1 Hz, 1H), 3.10 (t, *J* = 5.7 Hz, 1H), 2.36 (s, 2H), 2.17 (ddd, *J* = 20.4, 9.3, 5.7 Hz, 2H), 1.82 (dd, *J* = 9.4, 7.7 Hz, 1H), 1.56 (dd, *J* = 9.2, 7.7 Hz, 1H). ^{13}C NMR (151 MHz, $CDCl_3$) δ 147.65, 136.71, 128.44, 127.54, 126.85, 124.69, 75.23, 69.80, 47.51, 39.94, 36.78, 33.70. HRMS-ESI (m/z). Calculated for $C_{12}H_{14}O_2Na$ $[M+Na]^+$, 213.0886; found 217.0883.

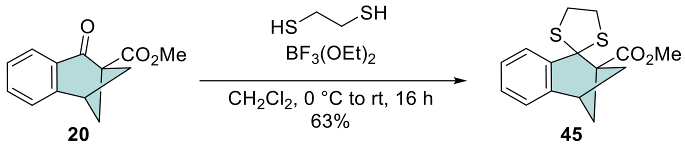

45. **20** (15 mg, 69.4 μmol, 1 eq) and 1,2-diethanethiol (23.3 mL, 277 μmol, 4 eq) in CH_2Cl_2 (0.4 mL) were cooled to 0 ºC. $BF_3.OEt_2$ (34.2 mL, 277 μmol, 4 eq) was added over the course of 1 minute. The reaction mixture was allowed to reach room temperature, and stirred for 16 hours. Saturated aqueous $NaHCO_3$ was used to quench the reaction mixture, and the aqueous layer was extracted with EtOAc. The combined organic layers were dried with Na_2SO_4 and concentrated. The crude product was purified by silica gel chromatography (5% EtOAc in pentane) to yield the title compound as a white powder (12.8 mg, 43.7 μmol, 63%). ^{1}H NMR (600 MHz, $CDCl_3$) δ 7.86 (d, *J* = 7.9 Hz, 1H), 7.24 (dd, *J* = 7.7, 1.4 Hz, 1H), 7.08 (td, *J* = 7.4, 1.2 Hz, 1H), 6.90 (dd, *J* = 7.5, 1.4 Hz, 1H), 3.77 (s, 3H), 3.64 (td, *J* = 6.8, 3.8 Hz, 2H), 3.39 (td, *J* = 6.7, 3.7 Hz, 2H), 3.01 (t, *J* = 5.8 Hz, 1H), 2.80 – 2.73 (m, 2H), 2.06 (dt, *J* = 7.8, 5.0 Hz, 2H). ^{13}C NMR (151 MHz, $CDCl_3$) δ 173.67, 145.34, 139.52, 131.43, 127.16, 127.08, 123.87, 73.85, 56.95, 52.26, 42.45, 41.14, 34.28. HRMS-ESI (m/z). Calculated for $C_{15}H_{17}O_2S_2$ $[M+H]^+$, 293.0665; found 293.0659.

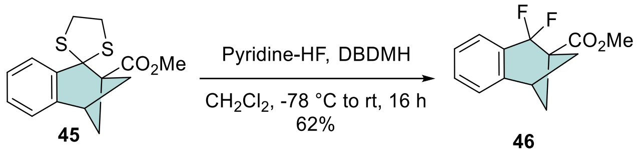

46. DBDMH (11.4 mg, 39.8 μmol, 1 eq) was dissolved in CH_2Cl_2 (0.2 mL) under an N_2 atmosphere and cooled to -78 ºC. 70% pyridine-HF (102 mL, 795 μmol, 20 eq) was added at this temperature. **45** (11.6 mg, 39.8 μmol, 1 eq) in CH_2Cl_2 (0.5 mL) was added dropwise and the reaction mixture was left to stir at room temperature for 16 hours. The reaction was quenched with saturated aqueous $NaHCO_3$, and the aqueous layer extracted with CH_2Cl_2. The organic layer was dried with Na_2SO_4 and the solvent removed in vacuo. The crude product was purified by preparatory thin layer chromatography (5% EtOAc in pentane) to yield the title compound as a clear oil (6.5 mg, 24.7 μmol, 62%). ^{1}H NMR (600 MHz, $CDCl_3$) δ 7.70 (dd, *J* = 7.5, 1.1 Hz, 1H), 7.37 – 7.34 (m, 1H), 7.34 – 7.32 (m, 1H), 7.12 (d, *J* = 7.4 Hz, 1H), 3.83 (s, 3H), 3.09 (t, *J* = 5.9 Hz, 1H), 2.88 (dddd, *J* = 8.5, 5.9, 2.5, 1.1 Hz, 2H), 2.32 – 2.27 (m, 2H).

^{19}F NMR (565 MHz, $CDCl_3$) δ -96.42. ^{13}C NMR (151 MHz, $CDCl_3$) δ 170.55, 145.79 (t, J = 6.3 Hz), 130.43, 130.13 (t, J = 25.1 Hz), 127.53, 126.46, 124.57, 121.42 (t, J = 246.0 Hz), 52.74 (t, J = 22.1 Hz), 52.56, 41.77 (t, J = 2.5 Hz), 35.03. HRMS-ESI (m/z) Calculated for $C_{13}H_{13}F_2O_2$ $[M+H]^+$, 239.0878; found 239.0595.

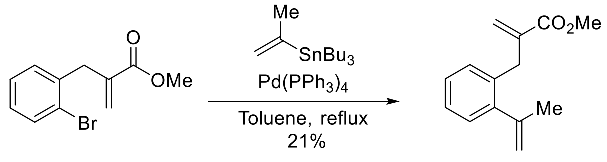

S28. Bromide **S21** (150 mg, 0.59 mmol) was dissolved in toluene (3 mL) and was degassed with nitrogen for 5 minutes. Then, tributyl(prop-1-en-2-yl)stannane (214 mg, 0.65 mmol, 1.1 eq) was added and the solution was degassed for a further 5 minutes. Finally, $Pd(PPh_3)_4$ (34 mg, 27.4 µmol, 5 mol%) was added and the mixture was degassed for a further 5 minutes, then heated at reflux temperature for 16 hours. The reaction mixture was cooled, filtered over celite, then concentrated. The residue was purified by silica gel chromatography with 10% crushed KF (2% EtOAc in pentane) to afford the title compound as a clear oil (27.2 mg, 123.5 µmol, 21%). ^1H NMR (600 MHz, $CDCl_3$) δ 7.21 (d, J = 3.4 Hz, 1H), 7.20 (d, J = 3.4 Hz, 1H), 7.17 – 7.15 (m, 1H), 7.15 – 7.12 (m, 1H), 6.25 (q, J = 1.3 Hz, 1H), 5.27 (q, J = 1.6 Hz, 1H), 5.16 (p, J = 1.6 Hz, 1H), 4.82 (dd, J = 2.1, 1.1 Hz, 1H), 3.75 (s, 3H), 3.67 (t, J = 1.6 Hz, 2H), 2.02 (t, J = 1.2 Hz, 3H). ^{13}C NMR (151 MHz, $CDCl_3$) δ 167.70, 145.21, 144.34, 140.78, 135.00, 130.11, 128.38, 127.05, 126.50, 126.48, 115.09, 52.03, 35.04, 25.16. HRMS-ESI (m/z) Calculated for $C_{14}H_{17}O_2$ $[M+H]^+$, 217.1223; found 217.1223.

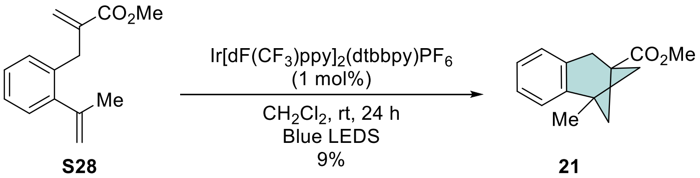

21. **S28** (60 mg, 0.28 mmol) was dissolved in CH_2Cl_2 (10 mL) and degassed with N_2 for 10 minutes. $Ir[dF(CF3)ppy]_2(dtbbpy)PF_6$ (3.1 mg, 2.8 µmol, 1 mol%) was added to the reaction mixture, which was degassed for a further 10 minutes. The solution was stirred vigorously while irradiating with a blue LED lamp (440 nm) at a distance of 2 cm at 25 ºC, using a stream of nitrogen to cool the flask. After 16 hours, the reaction was concentrated and purified by silica gel chromatography (0 to 2% EtOAc in pentane), followed by preparative thin layer chromatography (2% EtOAc in pentane) to yield to afford the product as a colourless oil (5.2 mg, 25 µmol, 9%). ^1H NMR (600 MHz, $CDCl_3$) δ 7.22 – 7.20 (m, 1H), 7.19 (dd, J = 6.1, 2.4 Hz, 1H), 7.18 – 7.15 (m, 1H), 7.15 – 7.13 (m, 1H), 3.75 (s, 3H), 3.27 (s, 2H), 2.40 – 2.35 (m, 2H), 1.82 – 1.77 (m, 2H), 1.47 (s, 3H).^{13}C NMR (151 MHz, $CDCl_3$) δ 175.69, 148.59, 133.83, 128.43, 126.28, 125.48, 121.91, 51.99, 42.71, 42.46, 38.04, 37.06, 22.42. HRMS-ESI (m/z) Calculated for $C_{14}H_{17}O_2$ $[M+H]^+$, 217.1223; found 217.1225.

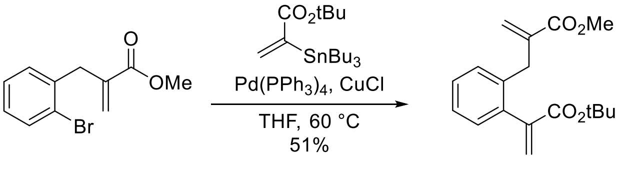

S29. Bromide **S21** (460 mg, 1.80 mmol) was dissolved in THF (15 mL) and degassed with nitrogen for 5 minutes. Then, *tert*-butyl 2-(tributylstannyl)acrylate (1.0 g, 2.4 mmol, 1.3 eq) was

added and the solution was degassed for a further 5 minutes. Finally, $Pd(PPh_3)_4$ (208 mg, 0.18 mmol, 10 mol%) and CuCl (202 mg, 2.0 mmol, 1.1 eq) were added and the mixture was degassed for a further 5 minutes, then heated at 60 °C for 16 hours. The reaction mixture was cooled, filtered over celite, then concentrated. The residue was purified by silica gel chromatography with 10% crushed KF (5 to 10% EtOAc in pentane) to afford the title compound as a clear oil (279 mg, 0.92 mmol, 51%). ^{1}H NMR (600 MHz, $CDCl_3$) δ 7.26 (td, *J* = 8.7, 7.2, 1.5 Hz, 1H), 7.21 (td, *J* = 7.4, 1.5 Hz, 1H), 7.17 – 7.13 (m, 2H), 6.38 (d, *J* = 1.8 Hz, 1H), 6.21 (q, *J* = 1.3 Hz, 1H), 5.61 (d, *J* = 1.7 Hz, 1H), 5.26 (q, *J* = 1.6 Hz, 1H), 3.72 (s, 3H), 3.57 (t, *J* = 1.5 Hz, 2H), 1.46 (s, 9H). ^{13}C NMR (151 MHz, $CDCl_3$) δ 167.53, 165.87, 142.68, 139.68, 138.07, 136.46, 130.21, 129.77, 128.07, 127.78, 126.85, 126.39, 81.22, 52.00, 35.38, 28.09. HRMS-ESI (m/z) Calculated for $C_{18}H_{23}O_4$ $[M+H]^+$, 303.1591; found 303.1604.

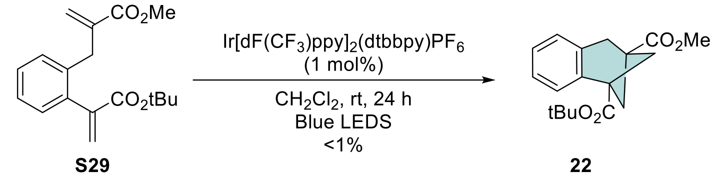

22. Diene **S29** (100 mg, 458 µmol) in CH_2Cl_2 (20 mL) was added to a vial charged with $Ir[dF(CF_3)ppy]_2(dtbbpy)PF_6$ (5 mg, 4.58 µmol, 1 mol%) under a N_2 atmosphere. The solution was stirred vigorously while irradiating with a blue LED lamp (440 nm) at a distance of 2 cm, at 25 °C using a stream of nitrogen to cool the flask. After 24 hours, the reaction was concentrated and purified by silica gel chromatography (50% CH_2Cl_2 in pentane) to afford the title compound as a yellow oil (19 mg, 87 µmol, 19%). ^{1}H NMR (600 MHz, $CDCl_3$) δ 7.24 – 7.18 (m, 2H), 7.12 (td, *J* = 7.3, 1.9 Hz, 1H), 7.07 – 7.04 (m, 1H), 3.76 (s, 3H), 3.28 (s, 2H), 2.83 – 2.75 (m, 2H), 2.00 – 1.95 (m, 2H), 1.55 (s, 9H). ^{13}C NMR (151 MHz, $CDCl_3$) δ 174.89, 172.86, 143.67, 133.15, 129.02, 127.05, 125.71, 123.12, 81.37, 52.18, 47.59, 41.04, 40.30, 36.41, 28.33. HRMS-ESI (m/z) Calculated for $C_{18}H_{23}O_4$ $[M+H]^+$, 303.1591; found 303.1586.

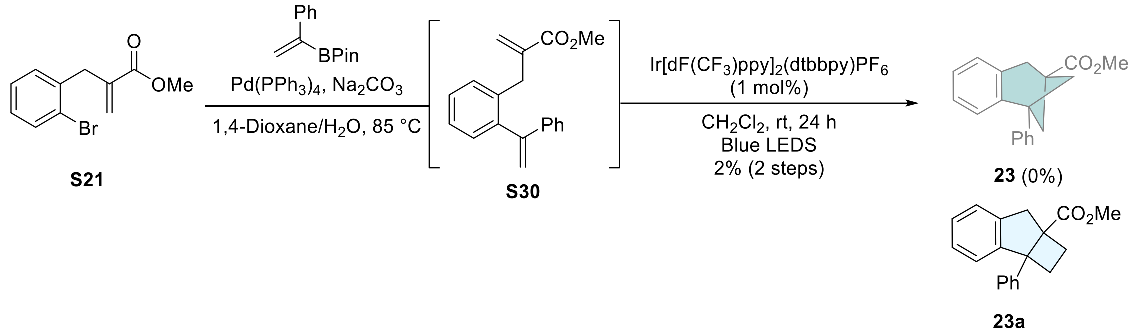

23a. Bromide **S21**, 4,4,5,5-tetramethyl-2-(1-phenylvinyl)-1,3,2-dioxaborolane (352 mg, 1.53 mmol, 1.1 eq), $Pd(PPh_3)_4$ (80.4 mg, 70 µmol, 5 mol%) and Na_2CO_3 (295 mg, 2.78 mmol, 2 eq) were added to a vial charged with N_2. A 5:1 mixture of 1,4-dioxane/H_2O (10 mL) was degassed with N_2 for 15 minutes. The reaction mixture was heated at 85 °C for 15 hours. The reaction mixture was diluted with EtOAc and the organic layer was washed with brine, dried over Na_2SO_4 and concentrated under reduced pressure. The crude product was purified by silica gel chromatography (0 to 2% EtOAc in pentane) to afford an impure mixture of **S30** (217 mg). This was immediately dissolved in CH_2Cl_2 (30 mL) and degassed with N_2 for 10 minutes. $Ir[dF(CF3)ppy]_2(dtbbpy)PF_6$ (8.7 mg, 7.79 µmol) was added to the reaction mixture, which was degassed for a further 10 minutes. The solution was stirred vigorously while irradiating with a blue LED lamp (440 nm) at a distance of 2 cm at 25 ºC, using a stream of nitrogen to cool the flask. After 16 hours, the reaction was concentrated and purified by silica gel chromatography

(0 to 2% EtOAc in pentane), followed by preparative thin layer chromatography (2% EtOAc in pentane) to yield to afford undesired **23a** as a colourless oil (2 mg, 6 µmol, 2%). ^{1}H NMR (600 MHz, $CDCl_3$) δ 7.38 (dt, *J* = 7.6, 1.0 Hz, 1H), 7.28 (dd, *J* = 7.4, 1.9 Hz, 1H), 7.27 – 7.24 (m, 2H), 7.21 (t, *J* = 7.4 Hz, 1H), 7.19 – 7.15 (m, 1H), 7.07 – 7.03 (m, 2H), 6.97 (d, *J* = 7.6 Hz, 1H), 3.94 (d, *J* = 16.5 Hz, 1H), 3.35 (s, 3H), 3.30 (q, *J* = 10.2 Hz, 1H), 3.06 (d, *J* = 16.5 Hz, 1H), 2.62 (ddd, *J* = 12.4, 10.0, 2.8 Hz, 1H), 2.27 (ddd, *J* = 10.9, 9.2, 2.8 Hz, 1H), 1.97 (dt, *J* = 11.8, 9.5 Hz, 1H). ^{13}C NMR (151 MHz, $CDCl_3$) δ 174.45, 149.70, 144.03, 142.16, 128.08, 127.56, 127.44, 126.53, 126.42, 125.35, 125.12, 63.41, 59.79, 51.59, 41.56, 30.79, 25.96. HRMS-ESI (m/z) Calculated for $C_{19}H_{19}O_2$ $[M+H]^+$, 279.1380; found 279.1373.

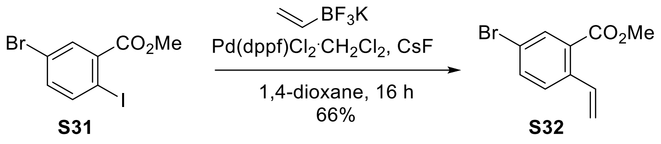

S32. Iodide **S31** (2.80 g, 8.2 mmol), potassium vinyltrifluoroborate (1.1 g, 8.2 mmol, 1.0 eq), CsF (5.0 g, 32.9 mmol, 4.0 eq) and Pd(dppf)Cl_2·DCM (669 mg, 0.8 mmol, 0.1 eq) were placed in a flask under N_2. Then degassed 1,4-dioxane (50 mL) was added and the reaction was stirred at 95 °C bath temperature for 16 hours. The mixture was then cooled, filtered over celite and washed with EtOAc. The solution was concentrated and purified by silica gel chromatography (0.5 to 1.5% EtOAc in pentane) to afford the title compound as a pale yellow oil (1.3 g, 5.4 mmol, 66%). ^{1}H NMR (600 MHz, $CDCl_3$) δ 8.02 (d, *J* = 2.2 Hz, 1H), 7.60 (ddd, *J* = 8.4, 2.2, 0.7 Hz, 1H), 7.45 (d, *J* = 8.4 Hz, 1H), 7.40 (ddt, *J* = 17.4, 11.0, 0.6 Hz, 1H), 5.65 (dt, *J* = 17.4, 0.8 Hz, 1H), 5.38 (dt, *J* = 11.0, 0.8 Hz, 1H), 3.91 (s, 3H). ^{13}C NMR (151 MHz, $CDCl_3$) δ 166.61, 138.65, 135.22, 134.97, 133.31, 130.17, 128.94, 121.25, 117.32, 52.51. HRMS-ESI (m/z) Calculated for $C_{10}H_9BrO_2Na$ $[M+Na]^+$, 262.9678; found 262.9680.

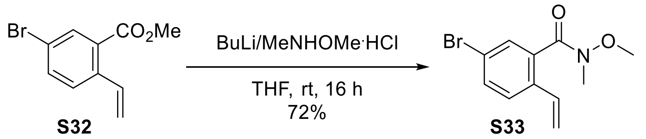

S33. N,O-Dimethylhydroxylamine hydrochloride (789 mg, 8.1 mmol, 1.5 eq) was placed in THF (30 mL), then n-butyllithium 2.5M in hexanes (6.5 mL, 16.2 mmol, 3.0 eq) was added with ice cooling. The mixture was stirred until the suspension was dissolved. Then Ester **S32** (1.3 g, 5.4 mmol) in THF (5 mL) was added, and the solution was stirred at room temperature for 16 hours. The mixture was quenched with ammonium chloride, then extracted with EtOAc. The combined organic layer was dried with $MgSO_4$. The solution was concentrated and purified by silica gel chromatography (80% to 90% CH_2Cl_2 in pentane) to afford the title compound as a white solid (1.3 g, 5.4 mmol, 66%). ^{1}H NMR (400 MHz, $CDCl_3$) δ 7.49 (d, *J* = 1.9 Hz, 1H), 7.47 (s, 1H), 7.44 (d, *J* = 2.0 Hz, 1H), 6.71 (dd, *J* = 17.5, 11.0 Hz, 1H), 5.75 (dd, *J* = 17.5, 0.8 Hz, 1H), 5.36 (d, *J* = 11.0 Hz, 1H), 3.50 (d, *J* = 43.1 Hz, 3H), 3.36 (s, 3H). Data consistent with that given in the literature. [7]

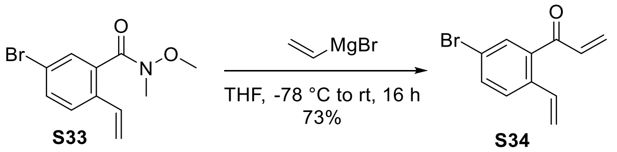

S34. Weinreb amide **S33** (460 mg, 1.7 mmol) was dissolved in THF (6 mL). Then vinylmagnesium bromide 1.0 M in THF (1.8 mL, 1.8 mol, 1.05 eq) was slowly added at -78 °C

and allowed to warm to room temperature for 16 hours. The mixture was quenched with ammonium choride solution at -78 °C. The mixture was diluted and extracted with EtOAc, then the combined organic layers were dried with $MgSO_4$. The solution was concentrated and purified by silica gel chromatography (5% EtOAc in pentane) to afford the title compound as a clear oil (293 mg, 1.2 mmol, 73%). ^{1}H NMR (400 MHz, $CDCl_3$) δ 7.57 (d, *J* = 1.9, 8.3 Hz, 2H), 7.54 (d, *J* = 1.9 Hz, 1H), 7.48 (d, *J* = 8.3 Hz, 1H), 6.83 (dd, *J* = 17.4, 11.0 Hz, 1H), 6.72 (dd, *J* = 17.5, 10.6 Hz, 1H), 6.15 (dd, *J* = 17.5, 1.1 Hz, 1H), 6.06 (dd, *J* = 10.6, 1.1 Hz, 1H), 5.69 (dd, *J* = 17.4, 0.9 Hz, 1H), 5.35 (dd, *J* = 11.0, 0.9 Hz, 1H). Data consistent with that given in the literature. [7]

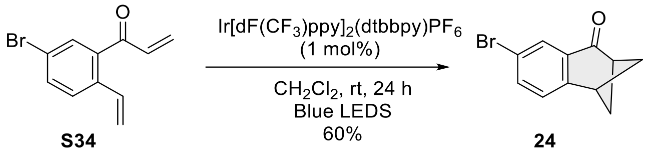

24. Diene **S34** (290 mg, 1.22 mmol) in CH_2Cl_2 (60 mL) was added to a vial charged with Ir[dF(CF_3)ppy]$_2$(dtbbpy)PF_6 (14 mg, 1 mol%) under a N_2 atmosphere. The solution was stirred vigorously while irradiating with a blue LED lamp (440 nm) at a distance of 2 cm, at 25 °C using a stream of nitrogen to cool the flask. After 24 hours, the reaction was concentrated and purified by silica gel chromatography (20 to 35% CH_2Cl_2 in pentane) to afford the title compound as a clear oil (173 mg, 731 μmol, 60%). ^{1}H NMR (400 MHz, $CDCl_3$) δ 8.09 (d, *J* = 2.1 Hz, 1H), 7.54 (dd, *J* = 8.0, 2.1 Hz, 1H), 7.12 (d, *J* = 7.9 Hz, 1H), 3.30 (q, *J* = 5.6 Hz, 1H), 3.23 (q, *J* = 5.8 Hz, 1H), 2.95 (dtd, *J* = 6.8, 5.7, 2.7 Hz, 3H), 2.36 – 2.29 (m, 2H). Data consistent with that given in the literature. [7]

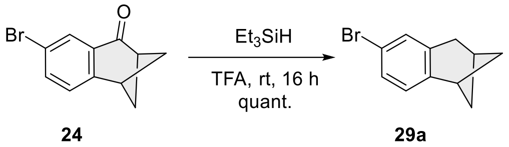

29a. Ketone **24** (160 mg, 675 μmol) was dissolved in TFA (1.6 mL). Then triethylsilane (256 μL, 1.62 mmol, 2.4 eq) was added and the reaction was stirred at room temperature for 16 hours. The mixture was dried under a stream of nitrogen, then purified by silica gel chromatography (100% pentane) to afford the title compound as a white solid (150 mg, 675 μmol, 100%). ^{1}H NMR (500 MHz, $CDCl_3$) δ 7.28 (d, *J* = 1.8 Hz, 1H), 7.17 (ddt, *J* = 8.0, 2.0, 0.9 Hz, 1H), 6.84 (d, *J* = 7.9 Hz, 1H), 3.06 (d, *J* = 5.5 Hz, 1H), 3.03 (d, *J* = 2.9 Hz, 2H), 2.78 (ddq, *J* = 8.9, 5.9, 2.9 Hz, 1H), 2.41 (qd, *J* = 6.1, 2.6 Hz, 2H), 1.45 – 1.39 (m, 2H). ^{13}C NMR (151 MHz, $CDCl_3$) δ 146.99, 136.09, 131.72, 128.03, 126.73, 119.41, 39.33, 34.84, 34.39, 33.60. HRMS-MSS (m/z) Calculated for $C_{11}H_{11}Br$ $[M]^+$, 222.0044; found 222.0311.

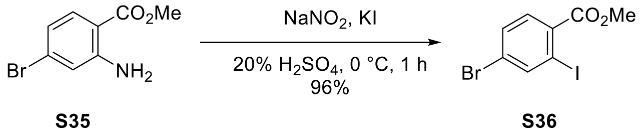

S36. Aniline **S35** (7.50 g, 32.6 mmol) was suspended in 20% sulphuric acid (aq) and cooled to 0 °C. Then sodium nitrite (2.70 g, 39.1 mmol, 1.2 eq) in cold water (20 mL) was added dropwise whilst keeping the temperature below 5 °C. The mixture was stirred with ice cooling for 40 minutes, after which KI (10.8 g, 65.2 mmol, 2.0 eq) was added dropwise whilst keeping the temperature below 5 °C. The mixture was stirred with ice cooling for 1 hour, after which the mixture was poured onto ice water. The aqueous layer was extracted with EtOAc 3x. The combined organic layers were washed sequentially with water, sodium thiosulphate, then

brine. The combined organic layers were dried with $MgSO_4$, then concentrated to afford the title compound as a yellow oil (10.63 g, 31.2 mmol, 96%). ^1H NMR (400 MHz, $CDCl_3$) δ 8.17 (d, *J* = 1.9 Hz, 1H), 7.69 (d, *J* = 8.4 Hz, 1H), 7.54 (dd, *J* = 8.3, 1.9 Hz, 1H), 3.92 (s, 3H). Data consistent with that given in the literature.[8]

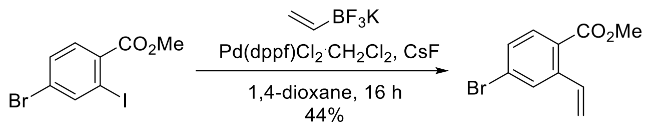

S36 **S37**

S37. Iodide **S36** (5.5 g, 16.1 mmol), potassium vinyltrifluoroborate (2.16 g, 16.1 mmol, 1.0 eq), CsF (9.8 g, 64.5 mmol, 4.0 eq) and $Pd(dppf)Cl_2 \cdot DCM$ (1.32 g, 1.6 mmol, 0.1 eq) were placed in a flask under N_2. Then degassed 1,4-dioxane (100 mL) was added and the reaction was stirred at 95 °C bath temperature for 16 hours. The mixture was then cooled, filtered over celite and washed with EtOAc. The solution was concentrated and purified by silica gel chromatography (0.5 to 1.5% EtOAc in pentane) to afford the title compound as a pale yellow oil (1.7 g, 7.1 mmol, 44%). ^1H NMR (600 MHz, $CDCl_3$) δ 7.76 (d, *J* = 8.4 Hz, 1H), 7.72 (d, *J* = 2.0 Hz, 1H), 7.45 (dd, *J* = 8.4, 2.0 Hz, 1H), 7.42 (dd, *J* = 17.4, 11.0 Hz, 1H), 5.66 (dd, *J* = 17.4, 1.0 Hz, 1H), 5.40 (dd, *J* = 11.0, 1.0 Hz, 1H), 3.89 (s, 3H). ^{13}C NMR (151 MHz, $CDCl_3$) δ 167.19, 141.74, 134.92, 132.10, 130.59, 130.43, 127.36, 127.18, 117.91, 52.39. HRMS-ESI (m/z) Calculated for $C_{10}H_9BrO_2Na$ $[M+Na]^+$, 262.9678; found 262.9926.

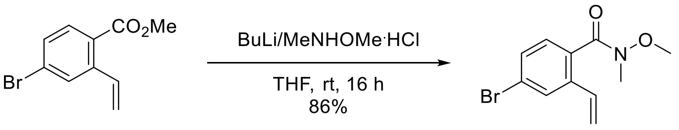

S37 **S38**

S38. N,O-Dimethylhydroxylamine hydrochloride (1.04 g, 10.6 mmol, 1.5 eq) was placed in THF (60 mL), then n-butyllithium 2.5M in hexanes (8.5 mL, 21.3 mmol, 3.0 eq) was added with ice cooling. The mixture was stirred until the suspension was dissolved. Then Ester **S37** (1.7 g, 7.1 mmol) in THF (10 mL) was added, and the solution was stirred at room temperature for 16 hours. The mixture was quenched with ammonium chloride, then extracted with EtOAc. The combined organic layer was dried with $MgSO_4$. The solution was concentrated and purified by silica gel chromatography (100% CH_2Cl_2 to afford the title compound as a white solid (1.64 g, 6.1 mmol, 86%). ^1H NMR (600 MHz, $CDCl_3$) δ 7.73 (d, *J* = 1.9 Hz, 1H), 7.42 (dd, *J* = 8.2, 1.9 Hz, 1H), 7.18 (d, *J* = 8.1 Hz, 1H), 6.73 (dd, *J* = 17.4, 11.0 Hz, 1H), 5.76 (d, *J* = 17.4 Hz, 1H), 5.38 (d, *J* = 11.0 Hz, 1H), 3.54 (br s, 3H), 3.36 (br s, 3H). ^{13}C NMR (151 MHz, $CDCl_3$) δ 168.77, 136.95, 133.02, 132.66, 130.35, 128.34, 128.34, 123.64, 117.73, 61.14, 32.37. HRMS-ESI (m/z) Calculated for $C_{11}H_{12}BrNO_2Na$ $[M+Na]^+$, 291.9944; found 291.9945.

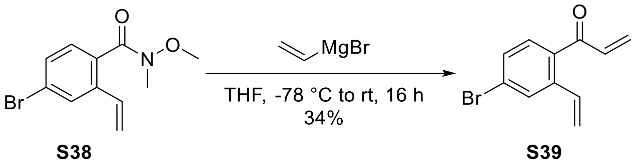

S38 **S39**

S39. Weinreb amide **S38** (1.64 g, 6.1 mmol) was dissolved in THF (25 mL). Then vinylmagnesium bromide 1.0 M in THF (6.4 mL, 6.4 mol, 1.05 eq) was slowly added at -78 °C and allowed to warm to room temperature for 16 hours. The mixture was quenched with ammonium choride solution at -78 °C. The mixture was diluted and extracted with EtOAc, then

the combined organic layers were dried with $MgSO_4$. The solution was concentrated and purified by silica gel chromatography (5% EtOAc in pentane) to afford the title compound as a clear oil (500 mg, 2.1 mmol, 34%). ^{1}H NMR (600 MHz, $CDCl_3$) δ 7.75 (d, J = 1.9 Hz, 1H), 7.46 (dd, J = 8.2, 1.9 Hz, 1H), 7.32 (d, J = 8.2 Hz, 1H), 6.88 (dd, J = 17.4, 11.0 Hz, 1H), 6.74 (dd, J = 17.5, 10.6 Hz, 1H), 6.15 (dd, J = 17.4, 1.1 Hz, 1H), 6.03 (dd, J = 10.6, 1.1 Hz, 1H), 5.71 (dd, J = 17.4, 0.8 Hz, 1H), 5.38 (dd, J = 10.9, 0.8 Hz, 1H). ^{13}C NMR (151 MHz, $CDCl_3$) δ 195.51, 139.21, 136.50, 136.16, 133.67, 132.15, 130.43, 130.11, 129.76, 125.69, 118.28. HRMS-ESI (m/z) Calculated for $C_{11}H_9BrONa$ $[M+Na]^+$, 258.9729; found 258.9732.

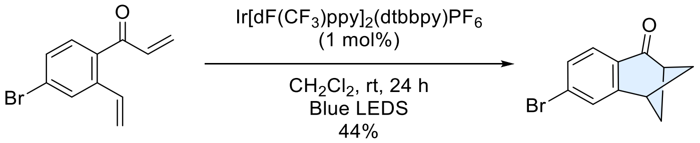

S39 **25**

25. Diene **S39** (470 mg, 2.0 mmol) in CH_2Cl_2 (85 mL) was added to a vial charged with Ir[dF(CF_3)ppy]$_2$(dtbbpy)PF_6 (22 mg, 1 mol%) under a N_2 atmosphere. The solution was stirred vigorously while irradiating with a blue LED lamp (440 nm) at a distance of 2 cm, at 25 °C using a stream of nitrogen to cool the flask. After 24 hours, the reaction was concentrated and purified by silica gel chromatography (20 to 30% CH_2Cl_2 in pentane) to afford the title compound as a clear oil (206 mg, 0.87 mmol, 44%). ^{1}H NMR (600 MHz, $CDCl_3$) δ 7.83 (d, J = 8.1 Hz, 1H), 7.49 (dd, J = 8.1, 1.9 Hz, 1H), 7.40 (d, J = 1.9 Hz, 1H), 3.28 (q, J = 5.6 Hz, 1H), 3.21 (q, J = 5.8 Hz, 1H), 2.98 – 2.92 (m, 2H), 2.36 – 2.31 (m, 2H). ^{13}C NMR (151 MHz, $CDCl_3$) δ 200.71, 153.50, 130.59, 128.47, 128.30, 128.03, 127.57, 49.60, 43.63, 40.11. HRMS-ESI (m/z) Calculated for $C_{11}H_9BrONa$ $[M+Na]^+$, 258.9729; found 258.9795.

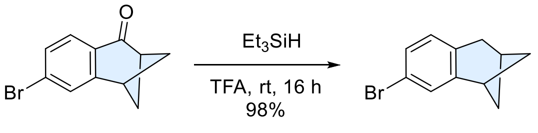

25 **29b**

29b. Ketone **25** (200 mg, 675 µmol) was dissolved in TFA (2.0 mL). Then triethylsilane (320 µL, 2.02 mmol, 2.4 eq) was added and the reaction was stirred at room temperature for 16 hours. The mixture was dried under a stream of nitrogen, then purified by silica gel chromatography (100% pentane) to afford the title compound as a white solid (185 mg, 0.83 mmol, 98%). ^{1}H NMR (600 MHz, $CDCl_3$) δ 7.26 (dd, J = 8.0, 2.0 Hz, 1H), 7.12 (d, J = 2.1 Hz, 1H), 7.01 (d, J = 8.0 Hz, 1H), 3.03 (q, J = 5.5 Hz, 1H), 2.99 (d, J = 2.9 Hz, 2H), 2.79 (qt, J = 5.9, 2.9 Hz, 1H), 2.41 (qd, J = 6.0, 2.6 Hz, 2H), 1.46 – 1.42 (m, 2H). ^{13}C NMR (151 MHz, $CDCl_3$) δ 150.25, 132.66, 130.48, 129.11, 128.09, 118.68, 39.67, 34.56, 34.35, 33.69. HRMS-MSS (m/z) Calculated for $C_{11}H_{11}Br$ $[M]^+$, 222.0044; found 222.0311.

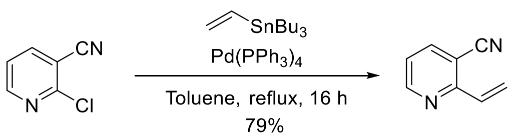

S40 **S41**

S41. Chloride **S40** (500 mg, 3.6 mmol) was dissolved in toluene (20 mL) and degassed with nitrogen for 5 minutes. Then, vinyltributyltin (1.16 mL, 4.0 mmol, 1.1 eq) was added and the solution was degassed for a further 5 minutes. Finally, $Pd(PPh_3)_4$ (417 mg, 0.36 mmol, 10 mol%) was added and the mixture was degassed for a further 5 minutes, then heated at reflux temperature for 16 hours. The reaction mixture was cooled, filtered over celite, then

concentrated. The residue was purified by silica gel chromatography with 10% crushed KF (8% EtOAc in pentane) to afford the title compound as a clear oil (370 mg, 2.84 mmol, 79%). ^{1}H NMR (400 MHz, $CDCl_3$) δ 8.76 (dd, *J* = 4.8, 1.8 Hz, 1H), 7.92 (dd, *J* = 7.9, 1.8 Hz, 1H), 7.28 (dd, *J* = 7.8, 4.7 Hz, 1H), 7.19 (dd, *J* = 16.8, 10.7 Hz, 1H), 6.66 (dd, *J* = 16.9, 1.5 Hz, 1H), 5.75 (dd, *J* = 10.7, 1.6 Hz, 1H). Data consistent with that given in the literature. [9]

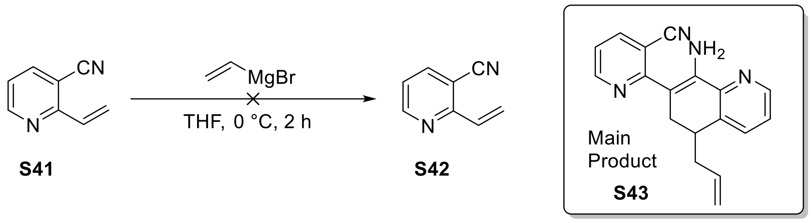

S43. Nitrile **S41** (250 mg, 1.92 mmol) was dissolved in THF (30 mL), then cooled to 0 °C. Then vinylmagnesium bromide 1.0M in THF (2.3 mL, 2.3 mmol, 1.2 eq) was added slowly at this temperature. The mixture was stirred under ice cooling for 2 hours, after which it was quenched with sat. ammonium chloride. The mixture was extracted with EtOAc, the combined organic layers dried with $MgSO_4$, then concentrated. No product was observed. Many attempts always afforded the thermodynamic sink of the reaction, which involved dimerization: ^{1}H NMR (400 MHz, $CDCl_3$) δ 9.05 (dd, *J* = 4.3, 1.7 Hz, 1H), 8.56 (dd, *J* = 7.8, 1.8 Hz, 1H), 8.52 (dd, *J* = 4.9, 1.8 Hz, 1H), 8.15 (dd, *J* = 8.4, 1.7 Hz, 1H), 7.39 (dd, *J* = 8.3, 4.3 Hz, 1H), 7.30 – 7.26 (m, 1H), 5.91 (dddd, *J* = 16.8, 10.1, 7.8, 6.5 Hz, 1H), 5.19 (s, 2H), 5.00 (ddt, *J* = 10.1, 2.2, 1.1 Hz, 1H), 4.93 (dq, *J* = 17.0, 1.6 Hz, 1H), 3.72 (dd, *J* = 16.4, 4.9 Hz, 1H), 3.38 (dd, *J* = 16.3, 6.5 Hz, 1H), 3.30 (ddt, *J* = 9.5, 6.5, 5.0 Hz, 1H), 2.58 (dddt, *J* = 13.1, 6.5, 5.1, 1.4 Hz, 1H), 2.33 (dddt, *J* = 13.9, 8.9, 7.8, 1.1 Hz, 1H). ^{13}C NMR (101 MHz, $CDCl_3$) δ 161.33, 155.31, 153.72, 151.46, 148.88, 145.53, 136.53, 132.36, 131.53, 129.84, 122.25, 120.50, 117.13, 116.84, 113.06, 39.92, 37.43, 24.77. HRMS-ESI (m/z) Calculated for $C_{18}H_{17}N_4$ $[M+H]^+$, 289.1448; found 289.1441.

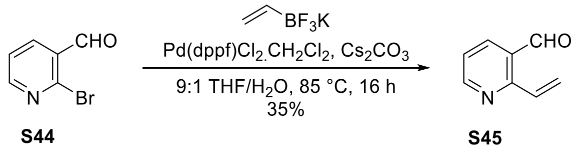

S45. Bromide **S44** (680 mg, 3.66 mmol), potassium vinyltrifluoroborate (500 mg, 3.73 mmol, 1.02 eq), Cs_2CO_3 (3.57 g, 11.0 mmol, 3.0 eq) and Pd(dppf)Cl_2·DCM (150 mg, 0.18 mmol, 0.05 eq) were placed in a flask under N_2. Then degassed 9:1 THF/H_2O (7.5 mL) was added and the reaction was stirred at 85 °C bath temperature for 16 hours. The mixture was then cooled, filtered over celite and washed with EtOAc. The organic layer was dried with $MgSO_4$, concentrated and purified by silica gel chromatography (CH_2Cl_2) to afford the title compound as a brown oil (170 mg, 1.28 mmol, 35%). ^{1}H NMR (400 MHz, $CDCl_3$) δ 10.38 (s, 1H), 8.77 (dd, *J* = 4.7, 1.8 Hz, 1H), 8.13 (dd, *J* = 7.8, 1.9 Hz, 1H), 7.59 (dd, *J* = 16.9, 10.8 Hz, 1H), 7.36 (dd, *J* = 7.8, 4.7 Hz, 1H), 6.49 (dd, *J* = 17.0, 1.8 Hz, 1H), 5.77 (dd, *J* = 10.8, 1.8 Hz, 1H). Data consistent with that given in the literature. [10]

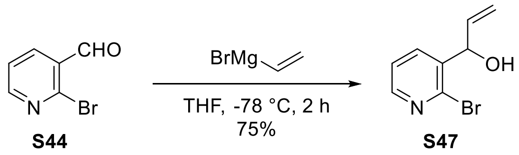

S47. Aldehyde **S44** (660 mg, 3.55 mmol) was dissolved in THF (25 mL), then cooled to -78 °C. Then vinylmagnesium bromide 1.0M in THF (5.3 mL, 5.3 mmol, 1.5 eq) was added slowly at this temperature. The mixture was stirred at to -78 °C for 2 hours, after which it was quenched with sat. ammonium chloride. The mixture was extracted with EtOAc, the combined

organic layers dried with $MgSO_4$, then concentrated. The residue was purified by silica gel chromatography (0 to 3% EtOAc in CH_2Cl_2) to afford the title compound as a white solid (570 mg, 2.66 mmol, 75%). ^{1}H NMR (600 MHz, $CDCl_3$) δ 8.28 (dd, *J* = 4.7, 2.0 Hz, 1H), 7.86 (ddd, *J* = 7.7, 2.0, 0.6 Hz, 1H), 7.30 (ddd, *J* = 7.6, 4.7, 0.5 Hz, 1H), 6.00 (ddd, *J* = 17.1, 10.4, 5.5 Hz, 1H), 5.55 (dt, *J* = 5.6, 1.5 Hz, 1H), 5.44 (dt, *J* = 17.2, 1.3 Hz, 1H), 5.27 (dt, *J* = 10.4, 1.3 Hz, 1H). ^{13}C NMR (151 MHz, $CDCl_3$) δ 149.22, 142.15, 139.15, 137.67, 136.64, 123.43, 116.75, 72.76. HRMS-ESI (m/z) Calculated for C_8H_9BrNO $[M+H]^+$, 213.9862; found 213.9861.

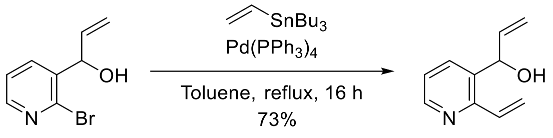

S46. Bromide **S47** (280 mg, 1.31 mmol) was dissolved in toluene (10 mL) and degassed with nitrogen for 5 minutes. Then, vinyltributyltin (0.4 mL, 1.37 mmol, 1.1 eq) was added and the solution was degassed for a further 5 minutes. Finally, $Pd(PPh_3)_4$ (75 mg, 65.4 μmol, 5 mol%) was added and the mixture was degassed for a further 5 minutes, then heated at reflux temperature for 16 hours. The reaction mixture was cooled, filtered over celite, then concentrated. The residue was purified by silica gel chromatography with 10% crushed KF (10% EtOAc in CH_2Cl_2) to afford the title compound as a clear oil (154 mg, 0.96 mmol, 73%). ^{1}H NMR (600 MHz, $CDCl_3$) δ 8.59 – 8.49 (m, 1H), 7.83 (d, *J* = 7.9 Hz, 1H), 7.23 (dd, *J* = 7.9, 4.7 Hz, 1H), 7.08 (dd, *J* = 16.9, 10.8 Hz, 1H), 6.47 – 6.35 (m, 1H), 6.04 (ddd, *J* = 16.3, 10.4, 5.5 Hz, 1H), 5.62 – 5.49 (m, 2H), 5.39 – 5.31 (m, 1H), 5.27 (d, *J* = 10.3 Hz, 1H), 2.06 (br s, 1H). ^{13}C NMR (151 MHz, $CDCl_3$) δ 152.39, 148.46, 138.95, 135.08, 134.92, 132.29, 122.97, 120.96, 116.48, 70.97. HRMS-ESI (m/z) Calculated for $C_{10}H_{12}NO$ $[M+H]^+$, 162.0913; found 162.0910.

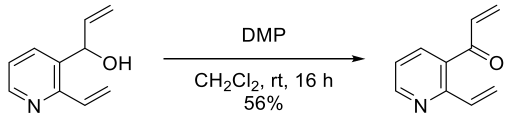

S48. To a solution of alcohol **S46** (153 mg, 0.95 mmol) in CH_2Cl_2 (5 mL) was added Dess-Martin periodinane (403 mg, 0.95 mmol, 1.0 eq). The mixture was stirred at room temperature for 16 hours, after which the suspension was diluted with CH_2Cl_2 (20 mL). Then, a saturated aqueous solution of $NaHCO_3$ and $Na_2S_2O_3$ (1:1, 20 mL) was added, and the mixture was stirred until the layers separated. The organic layer was dried with $MgSO_4$, then concentrated. The residue was purified by silica gel chromatography (0 to 3% EtOAc in CH_2Cl_2) to afford the title compound as a clear oil (85 mg, 0.53 mmol, 56%). ^{1}H NMR (600 MHz, $CDCl_3$) δ 8.68 (dd, *J* = 4.8, 1.7 Hz, 1H), 7.71 (dd, *J* = 7.7, 1.8 Hz, 1H), 7.28 – 7.22 (m, 1H), 6.95 (dd, *J* = 16.9, 10.7 Hz, 1H), 6.74 (dd, *J* = 17.5, 10.5 Hz, 1H), 6.44 (dd, *J* = 16.9, 1.8 Hz, 1H), 6.14 (dd, *J* = 17.5, 0.9 Hz, 1H), 6.08 (dd, *J* = 10.6, 1.0 Hz, 1H), 5.54 (dd, *J* = 10.7, 1.8 Hz, 1H). ^{13}C NMR (151 MHz, $CDCl_3$) δ 195.60, 153.22, 151.20, 136.61, 136.34, 133.51, 132.88, 132.75, 121.93, 121.59. HRMS-ESI (m/z) Calculated for $C_{10}H_{10}NO$ $[M+H]^+$, 160.0757; found 160.0755.

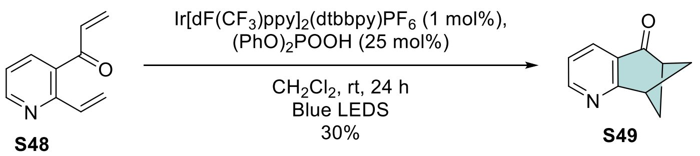

S49. Ketone **S48** (80 mg, 502 μmol) and diphenyl phosphoric acid (31.4 mg, 215 μmol, 0.25 eq) in CH_2Cl_2 (25 mL) was added to a vial charged with $Ir[dF(CF_3)ppy]_2(dtbbpy)PF_6$ (5.6 mg,

5 µmol, 1 mol%) under a N_2 atmosphere. The solution was stirred vigorously while irradiating with a blue LED lamp (440 nm) at a distance of 2 cm, at 25 °C using a stream of nitrogen to cool the flask. After 24 hours, the reaction was washed with sat. bicarbonate solution. The organic layer was dried with $MgSO_4$, then concentrated and purified by silica gel chromatography (CH_2Cl_2) to afford the title compound as a clear oil (24 mg, 150 µmol, 30%). ^{1}H NMR (600 MHz, $CDCl_3$) δ 8.61 – 8.48 (m, 1H), 8.23 (dd, *J* = 7.6, 1.7 Hz, 1H), 7.33 (dd, *J* = 7.6, 5.0 Hz, 1H), 3.52 (q, *J* = 5.8 Hz, 1H), 3.27 (q, *J* = 5.8 Hz, 1H), 3.03 (dtd, *J* = 7.1, 5.8, 2.7 Hz, 2H), 2.46 – 2.41 (m, 2H). ^{13}C NMR (151 MHz, $CDCl_3$) δ 200.39, 171.21, 152.40, 134.58, 124.12, 122.98, 49.53, 43.05, 42.80. HRMS-ESI (m/z) Calculated for $C_{10}H_{10}NO$ $[M+H]^+$, 160.0757; found 160.0754.

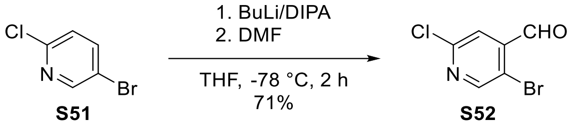

S52. DIPA (4.04 mL, 28.6 mmol, 1.72 eq) was dissolved in dry THF (52 mL) under nitrogen. Then n-Butyllithium 2.5 M in hexanes (10 mL, 24.9 mmol, 1.5 eq) was added at -78 °C. The mixture was stirred at this temperature for 30 minutes, after which bromide **S51** (3.2 g, 16.6 mmol, 1.0 eq) in dry THF (20 mL) was added, and the mixture was stirred for a further 30 minutes at this temperature. Then, DMF (3.86 mL, 49.9 mmol, 3.0 eq) was added and the solution was stirred for 1 hour at -78 °C. The reaction was quenched with sat. ammonium chloride, then extracted with EtOAc. The combined organic layers were dried with $MgSO_4$, concentrated and purified by silica gel chromatography (1 to 10% EtOAc in pentane) to afford the title compound as a beige solid (2.6 g, 11.8 mmol, 71%). ^{1}H NMR (400 MHz, $CDCl_3$) δ 10.30 (s, 1H), 8.68 (d, *J* = 0.6 Hz, 1H), 7.72 (d, *J* = 0.6 Hz, 1H). Data consistent with that given in the literature.[11]

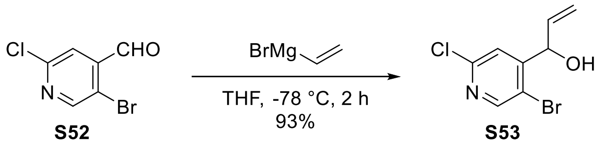

S53. Aldehyde **S52** (1.3 g, 6.6 mmol) was dissolved in THF (40 mL), then cooled to -78 °C. Then vinylmagnesium bromide 1.0M in THF (8.9 mL, 8.9 mmol, 1.5 eq) was added slowly at this temperature. The mixture was stirred at to -78 °C for 2 hours, after which it was quenched with sat. ammonium chloride. The mixture was extracted with EtOAc, the combined organic layers dried with $MgSO_4$, then concentrated. The residue was purified by silica gel chromatography (0 to 5% EtOAc in CH_2Cl_2) to afford the title compound as a yellow oil (1.53 g, 6.2 mmol, 93%). ^{1}H NMR (400 MHz, $CDCl_3$) δ 8.37 (d, *J* = 1.7 Hz, 1H), 7.56 (s, 1H), 5.91 (ddd, *J* = 16.6, 10.3, 5.9 Hz, 1H), 5.47 (d, *J* = 4.6 Hz, 1H), 5.43 (d, *J* = 13.0 Hz, 1H), 5.33 – 5.25 (m, 1H), 3.23 (s, 1H). ^{13}C NMR (101 MHz, $CDCl_3$) δ 153.71, 151.25, 151.03, 136.30, 123.24, 118.79, 117.90, 72.52. HRMS-ESI (m/z) Calculated for $C_8H_8BrClNO$ $[M+H]^+$, 247.9472; found 247.9469.

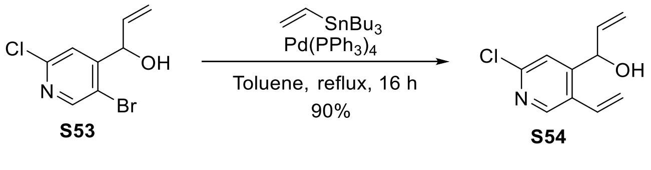

S54. Bromide **S53** (1.45 g, 5.83 mmol) was dissolved in toluene (45 mL) and degassed with nitrogen for 5 minutes. Then, vinyltributyltin (1.7 mL, 5.83 mmol, 1.0 eq) was added and the solution was degassed for a further 5 minutes. Finally, $Pd(PPh_3)_4$ (337 mg, 292 µmol, 5 mol%)

was added and the mixture was degassed for a further 5 minutes, then heated at reflux temperature for 16 hours. The reaction mixture was cooled, filtered over celite, then concentrated. The residue was purified by silica gel chromatography with 10% crushed KF (100% CH_2Cl_2) to afford the title compound as a clear oil (1.03 g, 5.26 mmol, 90%). ^1H NMR (500 MHz, $CDCl_3$) δ 8.36 (s, 1H), 7.49 (s, 1H), 6.82 (dd, J = 17.5, 11.1 Hz, 1H), 5.98 – 5.89 (m, 1H), 5.67 (dd, J = 17.5, 0.9 Hz, 1H), 5.44 (dd, J = 11.1, 1.0 Hz, 1H), 5.41 (dd, J = 5.9, 1.5 Hz, 1H), 5.33 (dt, J = 17.3, 1.3 Hz, 1H), 5.27 (dd, J = 10.3, 1.3 Hz, 1H), 2.66 (s, 1H). ^{13}C NMR (126 MHz, $CDCl_3$) δ 151.13, 151.10, 147.44, 137.56, 130.77, 130.34, 120.93, 119.38, 117.53, 70.87. HRMS-ESI (m/z) Calculated for $C_{10}H_{11}ClNO$ $[M+H]^+$, 196.0524; found 196.0522.

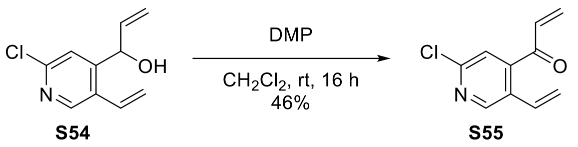

S55. To a solution of alcohol **S54** (1.0 g, 5.1 mmol) in CH_2Cl_2 (40 mL) was added Dess-Martin periodinane (2.28 g, 5.4 mmol, 1.0 eq). The mixture was stirred at room temperature for 16 hours, after which the suspension was diluted with CH_2Cl_2 (50 mL). Then, a saturated aqueous solution of $NaHCO_3$ and $Na_2S_2O_3$ (1:1, 40 mL) was added, and the mixture was stirred until the layers separated. The organic layer was dried with $MgSO_4$, then concentrated. The residue was purified by silica gel chromatography (100% CH_2Cl_2) to afford the title compound as a clear oil (460 mg, 2.38 mmol, 46%). ^1H NMR (600 MHz, $CDCl_3$) δ 8.60 (s, J = 0.7 Hz, 1H), 7.26 (s, J = 0.7 Hz, 1H), 6.73 – 6.68 (m, 1H), 6.68 – 6.64 (m, 1H), 6.17 (dq, J = 10.6, 0.6 Hz, 1H), 6.13 (dq, J = 17.5, 0.6 Hz, 1H), 5.76 (dq, J = 17.6, 0.6 Hz, 1H), 5.45 (dq, J = 11.1, 0.6 Hz, 1H). ^{13}C NMR (151 MHz, $CDCl_3$) δ 193.82, 150.55, 148.50, 146.45, 135.62, 134.36, 130.31, 130.14, 121.84, 119.93. HRMS-ESI (m/z) Calculated for $C_{10}H_9ClNO$ $[M+H]^+$, 194.0367; found 194.0366.

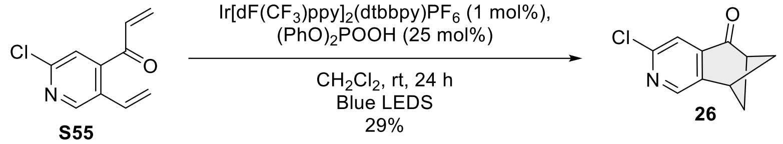

26. Diene **S55** (300 mg, 1.55 mmol) and diphenyl phosphoric acid (97 mg, 387 μmol, 0.25 eq) in CH_2Cl_2 (90 mL) was added to a vial charged with $Ir[dF(CF_3)ppy]_2(dtbbpy)PF_6$ (17.4 mg, 15.5 μmol, 1 mol%) under a N_2 atmosphere. The solution was stirred vigorously while irradiating with a blue LED lamp (440 nm) at a distance of 2 cm, at 25 °C using a stream of nitrogen to cool the flask. After 24 hours, the reaction was washed with sat. bicarbonate solution. The organic layer was dried with $MgSO_4$, then concentrated and purified by silica gel chromatography (50% to 100% pentane in CH_2Cl_2) to afford the title compound as a white solid (87 mg, 449 μmol, 29%). ^1H NMR (600 MHz, $CDCl_3$) δ 8.38 (s, 1H), 7.78 (s, 1H), 3.39 (q, J = 5.6 Hz, 1H), 3.30 (q, J = 5.8 Hz, 1H), 3.04 (dtd, J = 7.0, 5.8, 2.7 Hz, 2H), 2.36 – 2.30 (m, 2H). ^{13}C NMR (151 MHz, $CDCl_3$) δ 198.94, 151.19, 146.49, 143.63, 137.60, 120.62, 49.74, 43.09, 36.53. HRMS-ESI (m/z) Calculated for $C_{10}H_9ClNO$ $[M+H]^+$, 194.0367; found 194.0366.

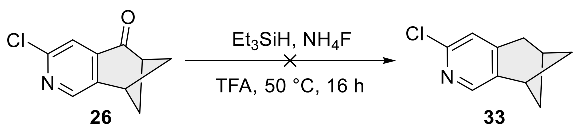

Ketone **S26** (8 mg, 41.3 μmol) was treated with TFA (32 μL, 495 μmol, 12.0 eq), triethylsilane (20 μL, 124 μmol, 3.0 eq), and ammonium fluoride (4.6 mg, 124 μmol, 3.0 eq), and stirred at

50 °C for 16 hours. The mixture was diluted in CH_2Cl_2, washed with sat. bicarb solution, then dried over $MgSO_4$ and concentrated. The crude NMR showed little conversion to product, and some reduction of pyridine imine.

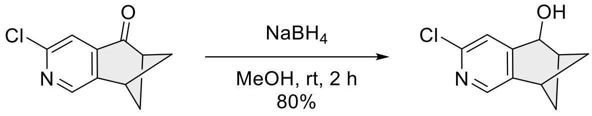

26 S56

S56. Ketone **26** (40 mg, 206.6 µmol) was dissolved in MeOH (2 mL), then sodium borohydride (23.5 mg, 619.7 µmol, 3.0 eq) was added. The mixture was stirred at room temperature for 2 hours, after which the mixture was diluted with EtOAc. The organic layer was washed with saturated bicarbonate solution, then dried with $MgSO_4$ and concentrated. The residue was purified by silica gel chromatography (5% EtOAc in CH_2Cl_2) to afford the title compound as a white solid (32.5 mg, 166.1 µmol, 80%). ^1H NMR (600 MHz, $CDCl_3$) δ 8.06 (s, 1H), 7.48 (s, 1H), 5.01 (d, J = 3.4 Hz, 1H), 3.20 (q, J = 5.4 Hz, 1H), 2.82 (qd, J = 5.9, 3.3 Hz, 1H), 2.64 (dt, J = 9.6, 6.1 Hz, 1H), 2.46 (dt, J = 9.5, 5.7 Hz, 1H), 1.65 (dd, J = 9.5, 8.1 Hz, 1H), 1.60 (dd, J = 9.5, 8.0 Hz, 1H). ^{13}C NMR (151 MHz, $CDCl_3$) δ 149.89, 147.74, 144.99, 141.82, 124.45, 70.91, 40.21, 37.71, 36.58, 31.20. HRMS-ESI (m/z) Calculated for $C_{10}H_9ClNO$ $[M+H]^+$, 196.0524; found 196.0522.

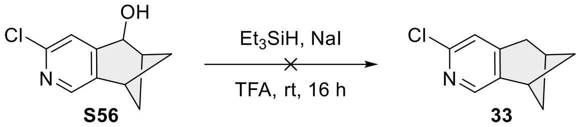

S56 33

Alcohol **S56** (8 mg, 41 µmol) was dissolved in TFA (0.2 mL). Then triethylsilane (26 µL, 163.6 µmol, 4.0 eq) and NaI (24.5 mg, 163.6 µmol, 4.0 eq) were added and the mixture was stirred at room temperature for 16 hours. The mixture was diluted with CH_2Cl_2, and the organic layer was washed with saturated bicarbonate solution, then dried with $MgSO_4$ and concentrated. No product was observed in the crude NMR.

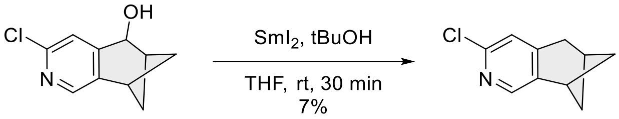

S56 33

31. Alcohol **S56** (25 mg, 127.8 µmol) and tBuOH (14.2 mg, 191.7 µmol, 1.5 eq) were placed under Argon. Then SmI_2 0.1M in THF (3.8 mL, 383 µmol, 3.0 eq) were added and the mixture was stirred at room temperature for 30 minutes. The mixture was diluted with EtOAc, and the organic layer was washed with saturated bicarbonate solution, then dried with $MgSO_4$ and concentrated. The residue was purified by silica gel chromatography (CH_2Cl_2) to afford the title compound as a white solid (1.5 mg, 8 µmol, 7%). ^1H NMR (600 MHz, $CDCl_3$) δ 7.98 (s, 1H), 7.13 (s, 1H), 3.15 (q, J = 5.4 Hz, 1H), 3.03 (d, J = 2.8 Hz, 2H), 2.80 (qt, J = 5.8, 2.8 Hz, 1H), 2.49 (qd, J = 6.0, 2.6 Hz, 2H), 1.41 (dt, J = 8.2, 4.1 Hz, 2H). ^{13}C NMR (151 MHz, $CDCl_3$) δ 149.10, 146.85, 144.79, 142.67, 124.29, 36.15, 34.64, 34.19, 33.05. HRMS-ESI (m/z) Calculated for $C_{10}H_{11}ClN$ $[M+H]^+$, 180.0575; found 180.0582.

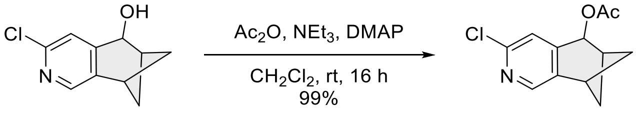

S56 S57

S57. Alcohol **S56** (20 mg, 102.2 μmol) was dissolved in CH_2Cl_2 (3 mL) under N_2. Then, triethylamine (42.8 μL, 306.7 μmol, 3.0 eq), acetic anhydride (19 μL, 204.5 μmol, 2.0 eq), and DMAP (3 mg, 25.6 μmol, 0.25 eq) were added and the solution was stirred at room temperature for 16 hours. The mixture was diluted with EtOAc, and the organic layer was washed with saturated bicarbonate solution, then brine, then dried with $MgSO_4$ and concentrated to afford the title compound as a white solid without further purification. ^{1}H NMR (600 MHz, $CDCl_3$) δ 8.09 (s, 1H), 7.30 (s, 1H), 6.14 (d, *J* = 3.4 Hz, 1H), 3.20 (q, *J* = 5.4 Hz, 1H), 2.84 (qd, *J* = 5.8, 3.5 Hz, 1H), 2.64 (ddd, *J* = 9.8, 6.6, 5.4 Hz, 1H), 2.46 (dt, *J* = 9.5, 5.7 Hz, 1H), 2.14 (s, 3H), 1.70 (dd, *J* = 9.6, 8.1 Hz, 1H), 1.64 (dd, *J* = 9.8, 8.1 Hz, 1H). ^{13}C NMR (151 MHz, $CDCl_3$) δ 170.91, 149.91, 145.22, 144.37, 142.26, 124.37, 71.86, 37.68, 37.60, 36.21, 32.00, 21.34. HRMS-ESI (m/z) Calculated for $C_{12}H_{13}ClNO_2$ $[M+H]^+$, 238.0629; found 238.0628.

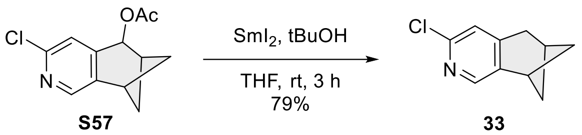

33. Ester **S57** (25 mg, 105.2 μmol) and tBuOH (15.6 mg, 210.4 μmol, 2.0 eq) were placed under Argon. Then SmI_2 0.1M in THF (6.3 mL, 631 μmol, 6.0 eq) were added and the mixture was stirred at room temperature for 3 hours. The mixture was diluted with EtOAc, and the organic layer was washed with saturated bicarbonate solution, then dried with $MgSO_4$ and concentrated. The residue was purified by silica gel chromatography (CH_2Cl_2) to afford the title compound as a white solid (15 mg, 83 μmol, 79%). ^{1}H NMR (600 MHz, $CDCl_3$) δ 7.98 (s, 1H), 7.13 (s, 1H), 3.15 (q, *J* = 5.4 Hz, 1H), 3.03 (d, *J* = 2.8 Hz, 2H), 2.80 (qt, *J* = 5.8, 2.8 Hz, 1H), 2.49 (qd, *J* = 6.0, 2.6 Hz, 2H), 1.41 (dt, *J* = 8.2, 4.1 Hz, 2H). ^{13}C NMR (151 MHz, $CDCl_3$) δ 149.10, 146.85, 144.79, 142.67, 124.29, 36.15, 34.64, 34.19, 33.05. HRMS-ESI (m/z) Calculated for $C_{10}H_{11}ClN$ $[M+H]^+$, 180.0575; found 180.0582.

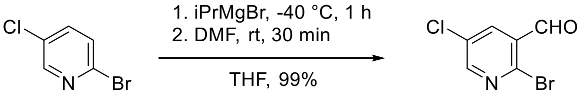

S59. Bromide **S58** (5.0 g, 18.4 mmol, 1.0 eq) was dissolved in dry in THF (40 mL) and the mixture was cooled to -40 °C with dry ice/MeCN. Then isopropyl magnesium bromide 2.0M in THF (10 mL, 20.1 mmol, 1.09 eq) was added, and the solution was stirred for 1 hour at this temperature. Then, DMF (4.2 mL, 54.4 mmol, 3.0 eq) was added and the solution was allowed to warm to room temperature for 30 minutes. The reaction was quenched with sat. ammonium chloride, then extracted with EtOAc. The combined organic layers were dried with $MgSO_4$, concentrated to afford the title compound as a beige solid which was left without further purification (4.03 g, 18.3 mmol, 99%). ^{1}H NMR (400 MHz, $CDCl_3$) δ 10.29 (s, 1H), 8.54 (d, *J* = 2.7 Hz, 1H), 8.13 (d, *J* = 2.7 Hz, 1H). Data consistent with that given in the literature.[12]

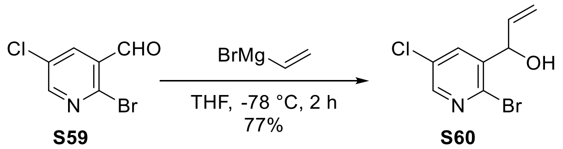

S60. Aldehyde **S59** (2.5 g, 11.3 mmol) was dissolved in THF (70 mL), then cooled to -78 °C. Then vinylmagnesium bromide 1.0M in THF (17 mL, 17 mmol, 1.5 eq) was added slowly at this temperature. The mixture was stirred at to -78 °C for 2 hours, after which it was quenched with sat. ammonium chloride. The mixture was extracted with EtOAc, the combined organic layers dried with $MgSO_4$, then concentrated. The residue was purified by silica gel chromatography (0 to 5% EtOAc in CH_2Cl_2) to afford the title compound as a yellow oil (2.18 g, 8.8 mmol, 77%). ^{1}H NMR (600 MHz, $CDCl_3$) δ 8.25 (d, *J* = 2.6 Hz, 1H), 7.87 (d, *J* = 2.7 Hz, 1H), 5.96 (ddd, *J* = 17.1, 10.4, 5.7 Hz, 1H), 5.51 – 5.48 (m, 1H), 5.48 – 5.45 (m, 1H), 5.30 (dt, *J* = 10.4, 1.2 Hz, 1H), 2.49 (br s, 1H). ^{13}C NMR (151 MHz, $CDCl_3$) δ 147.76, 140.33, 139.14, 137.01, 136.40, 132.31, 117.42, 72.42. HRMS-ESI (m/z) Calculated for $C_8H_8BrClNO$ $[M+H]^+$, 247.9472; found 247.9470.

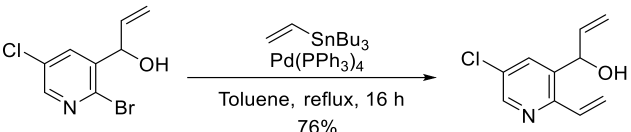

S61. Bromide **S60** (2.17 g, 8.73 mmol) was dissolved in toluene (60 mL) and degassed with nitrogen for 5 minutes. Then, vinyltributyltin (2.55 mL, 8.73 mmol, 1.0 eq) was added and the solution was degassed for a further 5 minutes. Finally, $Pd(PPh_3)_4$ (405 mg, 436 µmol, 5 mol%) was added and the mixture was degassed for a further 5 minutes, then heated at reflux temperature for 16 hours. The reaction mixture was cooled, filtered over celite, then concentrated. The residue was purified by silica gel chromatography with 10% crushed KF (1 to 3% EtOAc in CH_2Cl_2) to afford the title compound as a clear oil (1.3 g, 6.64 mmol, 76%). ^{1}H NMR (500 MHz, $CDCl_3$) δ 8.42 (d, *J* = 2.4 Hz, 1H), 7.82 (d, *J* = 2.4 Hz, 1H), 6.95 (dd, *J* = 16.8, 10.7 Hz, 1H), 6.34 (dd, *J* = 16.8, 1.9 Hz, 1H), 5.98 (ddd, *J* = 17.1, 10.3, 5.6 Hz, 1H), 5.53 (dd, *J* = 10.7, 1.9 Hz, 1H), 5.48 (d, *J* = 5.7 Hz, 1H), 5.34 (dt, *J* = 17.1, 1.2 Hz, 1H), 5.27 (dt, *J* = 10.4, 1.2 Hz, 1H), 2.49 (s, 1H). ^{13}C NMR (126 MHz, $CDCl_3$) δ 150.45, 147.32, 138.39, 136.02, 134.37, 131.47, 131.14, 121.18, 116.99, 70.38. HRMS-ESI (m/z) Calculated for $C_{10}H_{11}ClNO$ $[M+H]^+$, 196.0524; found 196.0522.

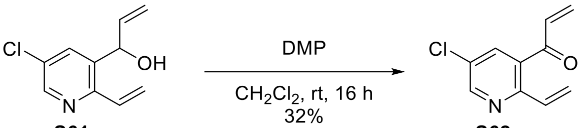

S62. To a solution of alcohol **S61** (1.28 g, 6.5 mmol) in CH_2Cl_2 (25 mL) was added Dess-Martin periodinane (2.91 g, 6.87 mmol, 1.0 eq). The mixture was stirred at room temperature for 16 hours, after which the suspension was diluted with CH_2Cl_2 (30 mL). Then, a saturated aqueous solution of $NaHCO_3$ and $Na_2S_2O_3$ (1:1, 30 mL) was added, and the mixture was stirred until the layers separated. The organic layer was dried with $MgSO_4$, then concentrated. The residue was purified by silica gel chromatography (25% pentane in CH_2Cl_2) to afford the title compound as a yellow oil (400 mg, 2.07 mmol, 32%). ^{1}H NMR (500 MHz, $CDCl_3$) δ 8.62 (d, *J* = 2.5 Hz, 1H), 7.68 (d, *J* = 2.4 Hz, 1H), 6.87 (dd, *J* = 16.8, 10.7 Hz, 1H), 6.72 (dd, *J* = 17.5, 10.6 Hz, 1H), 6.44 (dd, *J* = 16.9, 1.7 Hz, 1H), 6.20 – 6.14 (m, 1H), 6.13 (dd, *J* = 10.6, 0.8 Hz, 1H), 5.56 (dd, *J* = 10.7, 1.8 Hz, 1H). ^{13}C NMR (126 MHz, $CDCl_3$) δ 194.05, 151.25, 150.06, 136.17, 135.61, 133.52, 133.36, 132.41, 130.28, 122.22. HRMS-ESI (m/z) Calculated for $C_{10}H_9ClNO$ $[M+H]^+$, 194.0367; found 194.0367.

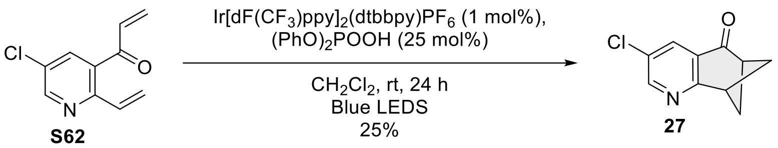

27. Diene **S62** (100 mg, 1.55 mmol) and diphenyl phosphoric acid (32.3 mg, 129 μmol, 0.25 eq) in CH_2Cl_2 (30 mL) was added to a vial charged with Ir[dF(CF_3)ppy]$_2$(dtbbpy)PF_6 (5.8 mg, 5.2 μmol, 1 mol%) under a N_2 atmosphere. The solution was stirred vigorously while irradiating with a blue LED lamp (440 nm) at a distance of 2 cm, at 25 °C using a stream of nitrogen to cool the flask. After 24 hours, the reaction was washed with sat. bicarbonate solution. The organic layer was dried with $MgSO_4$, then concentrated and purified by silica gel chromatography (50% to 100% pentane in CH_2Cl_2) to afford the title compound as a white solid (25 mg, 129.11 μmol, 25%). ^{1}H NMR (600 MHz, $CDCl_3$) δ 8.51 (d, *J* = 2.5 Hz, 1H), 8.18 (d, *J* = 2.5, 1H), 3.52 (q, *J* = 5.7 Hz, 1H), 3.28 (q, *J* = 5.8 Hz, 1H), 3.03 (dtd, *J* = 7.1, 5.9, 2.7 Hz, 2H), 2.43 – 2.38 (m, 2H). ^{13}C NMR (151 MHz, $CDCl_3$) δ 199.09, 169.01, 151.11, 149.71, 134.09, 131.48, 124.83, 49.30, 42.56, 42.49. HRMS-ESI (m/z) Calculated for $C_{10}H_9ClNO$ $[M+H]^+$, 194.0367; found 194.0368.

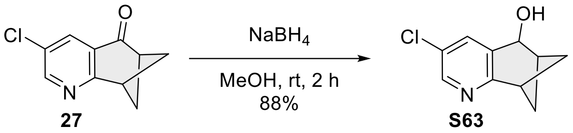

S63. Ketone **27** (23.5 mg, 121.4 μmol) was dissolved in MeOH (1.5 mL), then sodium borohydride (23.5 mg, 619.7 μmol, 3.0 eq) was added. The mixture was stirred at room temperature for 2 hours, after which the mixture was diluted with EtOAc. The organic layer was washed with saturated bicarbonate solution, then dried with $MgSO_4$ and concentrated. to afford the title compound as a white solid without further purification (21 mg, 107 μmol, 88%). ^{1}H NMR (600 MHz, $CDCl_3$) δ 8.26 (d, *J* = 2.4 Hz, 1H), 7.79 (dd, *J* = 2.4, 0.9 Hz, 1H), 5.02 (dd, *J* = 7.1, 3.5 Hz, 1H), 3.31 (q, *J* = 5.5 Hz, 1H), 2.81 (qd, *J* = 5.9, 3.5 Hz, 1H), 2.66 (ddd, *J* = 9.7, 6.5, 5.6 Hz, 1H), 2.48 (dt, *J* = 9.7, 5.8 Hz, 1H), 2.07 (d, *J* = 7.5 Hz, 1H), 1.73 (dd, *J* = 9.7, 8.1 Hz, 1H), 1.69 – 1.65 (m, 1H). ^{13}C NMR (151 MHz, $CDCl_3$) δ 165.07, 146.07, 136.83, 132.23, 130.34, 70.45, 42.37, 40.23, 37.07, 30.69. HRMS-ESI (m/z) Calculated for $C_{10}H_9ClNO$ $[M+H]^+$, 196.0524; found 196.0524.

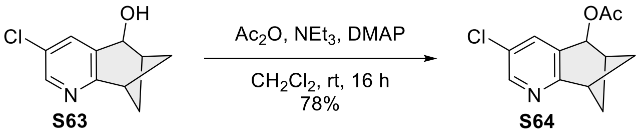

S64. Alcohol **S63** (20 mg, 102.2 μmol) was dissolved in CH_2Cl_2 (3 mL) under N_2. Then, triethylamine (42.8 μL, 306.7 μmol, 3.0 eq), acetic anhydride (19 μL, 204.5 μmol, 2.0 eq), and DMAP (3 mg, 25.6 μmol, 0.25 eq) were added and the solution was stirred at room temperature for 16 hours. The mixture was diluted with EtOAc, and the organic layer was washed with saturated bicarbonate solution, then brine, then dried with $MgSO_4$ and concentrated to afford the title compound as a white solid without further purification. ^{1}H NMR (600 MHz, $CDCl_3$) δ 8.30 (d, *J* = 2.4 Hz, 1H), 7.66 (d, *J* = 2.3 Hz, 1H), 6.17 (d, *J* = 3.6 Hz, 1H), 3.33 (q, *J* = 5.5 Hz, 1H), 2.86 (qd, *J* = 5.9, 3.6 Hz, 1H), 2.67 (dt, *J* = 9.9, 6.0 Hz, 1H), 2.51 (dt, *J* = 9.7, 5.8 Hz, 1H), 2.13 (s, 3H), 1.83 (dd, *J* = 9.7, 8.2 Hz, 1H), 1.71 (dd, *J* = 9.9, 8.2 Hz, 1H). ^{13}C NMR (151 MHz, $CDCl_3$) δ 171.15, 165.52, 146.46, 136.91, 130.25, 129.14, 71.73, 41.99,

37.54, 36.97, 31.59, 21.42. HRMS-ESI (m/z) Calculated for $C_{12}H_{13}ClNO_2$ [M+H]$^+$, 238.0629; found 238.0629.

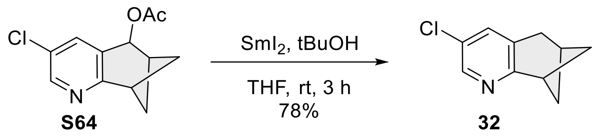

32. Ester **S64** (17 mg, 71.5 μmol) and tBuOH (10.6 mg, 143.1 μmol, 2.0 eq) were placed under Argon. Then SmI_2 0.1M in THF (4.3 mL, 429 μmol, 6.0 eq) were added and the mixture was stirred at room temperature for 3 hours. The mixture was diluted with EtOAc, and the organic layer was washed with saturated bicarbonate solution, then dried with $MgSO_4$ and concentrated. The residue was purified by silica gel chromatography (20% pentane in CH_2Cl_2) to afford the title compound as a white solid (15 mg, 83 μmol, 79%). ^{1}H NMR (600 MHz, $CDCl_3$) δ 8.19 (s, 1H), 7.46 (s, 1H), 3.31 (d, *J* = 5.8 Hz, 1H), 3.01 (d, *J* = 2.9 Hz, 2H), 2.83 (ddq, *J* = 8.8, 5.9, 2.9 Hz, 1H), 2.54 (qd, *J* = 6.0, 2.6 Hz, 2H), 1.52 – 1.46 (m, 2H). ^{13}C NMR (151 MHz, $CDCl_3$) δ 165.34, 143.67, 136.41, 130.54, 129.69, 41.80, 33.48, 33.23, 33.09. HRMS-ESI (m/z) Calculated for $C_{10}H_{11}ClN$ [M+H]$^+$, 180.0575; found 180.0575.

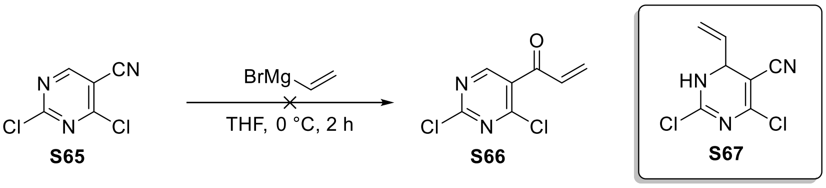

S66. Nitrile **S65** (48 mg, 287.4 μmol) was dissolved in THF (4 mL), then cooled to 0 °C. Then vinylmagnesium bromide 1.0M in THF (340 μL, 340 μmol, 1.2 eq) was added slowly at this temperature. The mixture was stirred at to 0 °C for 2 hours, after which it was quenched with sat. ammonium chloride. The mixture was extracted with EtOAc, the combined organic layers dried with $MgSO_4$, then concentrated. The main product was addition into the pyrimidine, so the reaction was abandoned. ^{1}H NMR (400 MHz, $CDCl_3$) δ 6.39 (br s, 1H), 5.98 (ddd, *J* = 17.1, 10.1, 7.2 Hz, 1H), 5.40 (dd, *J* = 10.1, 0.8 Hz, 1H), 5.35 (*J* = 17.1, 0.8 Hz, 1H), 4.88 (d, *J* = 7.2 Hz, 1H).

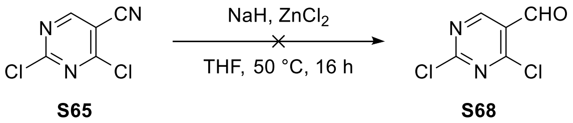

NaH 60% dispersion in mineral oil (60 mg, 1.5 mmol, 3.0 eq) and $ZnCl_2$ (102 mg, 0.75 mmol, 1.5 eq) were added to a dry vial under N_2. Nitrile **S65** (87 mg, 0.5 mmol) was dissolved in THF (2 mL), and added to the vial under nitrogen. Then the mixture was stirred at 50 °C for 16 hours, after which it was quenched with ammonium chloride. The mixture was extracted with EtOAc dried with $MgSO_4$, then concentrated to afford recovered starting material.

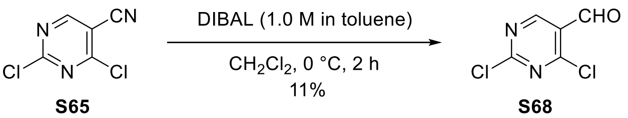

S68. Nitrile **S65** (1.1 g, 6.3 mmol) was dissolved in CH_2Cl_2 (20 mL), then cooled to 0 °C under nitrogen. Then DIBAL 1.0M in toluene (6.6 mL, 6.9 mmol, 1.2 eq) was added slowly at this temperature. The mixture was stirred at 0 °C for 2 hours, after which the mixture was diluted with CH_2Cl_2 (80 mL). A saturated solution of Rochelle's salt was added, and the mixture was

stirred until 2 clear layers formed. The organic layer was dried with $MgSO_4$, then concentrated. The residue was purified by silica gel chromatography (30% CH_2Cl_2 in pentane) to afford the title compound as a beige solid (125 mg, 0.7 mmol, 11%). ^{1}H NMR (400 MHz, $CDCl_3$) δ 10.39 (s, 1H), 9.01 (s, 1H). Data consistent with that given in the literature. [13]

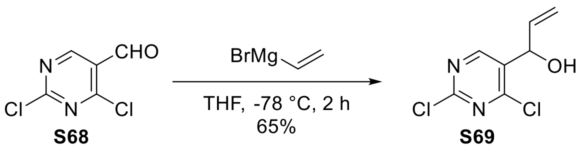

S69. Aldehyde **S68** (125 mg, 0.7 mmol) was dissolved in THF (5 mL), then cooled to -78 °C. Then vinylmagnesium bromide 1.0M in THF (1.06 mL, 1.06 mmol, 1.5 eq) was added slowly at this temperature. The mixture was stirred at to -78 °C for 2 hours, after which it was quenched with sat. ammonium chloride. The mixture was extracted with EtOAc, the combined organic layers dried with $MgSO_4$, then concentrated. The residue was purified by silica gel chromatography (0 to 3% EtOAc in CH_2Cl_2) to afford the title compound as a light yellow oil (94 mg, 0.46 mmol, 65%). ^{1}H NMR (600 MHz, $CDCl_3$) δ 8.76 (s, 1H), 6.00 – 5.93 (m, 1H), 5.52 (d, *J* = 6.1 Hz, 1H), 5.50 – 5.46 (m, 1H), 5.36 (dd, *J* = 10.3, 1.1 Hz, 1H), 2.26 (br s, 1H). ^{13}C NMR (151 MHz, $CDCl_3$) δ 160.36, 159.63, 159.10, 136.34, 132.91, 118.42, 69.85. HRMS-ESI (m/z) Calculated for $C_7H_7Cl_2N_2O$ $[M+H]^+$, 204.9930; found 204.9927.

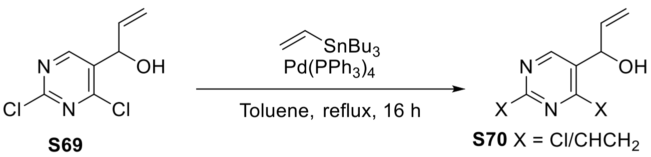

S70. Chloride **S69** (90 mg, 0.44 mmol) was dissolved in toluene (3 mL) and degassed with nitrogen for 5 minutes. Then, vinyltributyltin (129 µL, 0.44 mmol, 1.0 eq) was added and the solution was degassed for a further 5 minutes. Finally, $Pd(PPh_3)_4$ (25 mg, 22 µmol, 5 mol%) was added and the mixture was degassed for a further 5 minutes, then heated at reflux temperature for 16 hours. The reaction mixture was cooled, filtered over celite, then concentrated. The residue was purified by silica gel chromatography with 10% crushed KF (1 to 3% EtOAc in CH_2Cl_2) to afford an inseperable mixture of mono and divinylated products.

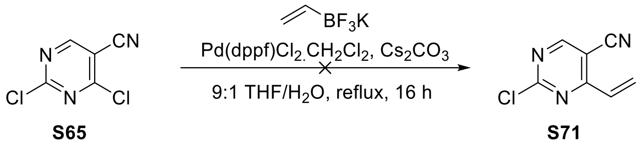

S71. Pyrimidine **S65** (200 mg, 1.15 mmol), potassium vinyltrifluoroborate (154 mg, 1.15 mmol, 1.0 eq), Cs_2CO_3 (1.12 g, 3.45 mmol, 3.0 eq) and $Pd(dppf)Cl_2$·DCM (47mg, 57 µmol, 0.05 eq) were placed in a flask under N_2. Then degassed 9:1 THF/H_2O (5 mL) was added and the reaction was stirred at reflux temperature for 16 hours. The mixture was then cooled, filtered over celite and washed with EtOAc. The organic layer was dried with $MgSO_4$, concentrated to afford a complex mixture that was abandoned.

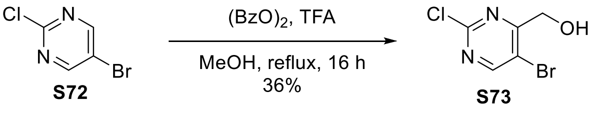

S73. Pyrimidine **S72** (5.0 g, 25.9 mmol) was dissolved in MeOH (100 mL). Then TFA (2.35 mL, 31.0 mmol, 1.2 eq) was added, followed by benzoyl peroxide (7.51 g, 31.0 mmol, 1.2 eq). The mixture was stirred at reflux temperature for 16 hours, after which the solvent was

removed under a stream of nitrogen. The residue was dissolved in EtOAc, and the organic layer was washed with saturated bicarbonate solution, then dried with $MgSO_4$ and concentrated. The residue was purified by silica gel chromatography (10% EtOAc in pentane) to afford the title compound as a white solid (2.1 g, 9.4 mmol, 36%). ^{1}H NMR (600 MHz, $CDCl_3$) δ 8.62 (s, 1H), 4.77 (d, J = 4.8 Hz, 2H), 3.53 (t, J = 5.2 Hz, 1H). ^{13}C NMR (151 MHz, $CDCl_3$) δ 168.97, 160.22, 159.54, 116.60, 63.13. HRMS-ESI (m/z) Calculated for $C_5H_5BrClN_2O$ $[M+H]^+$, 222.9268; found 222.9268.

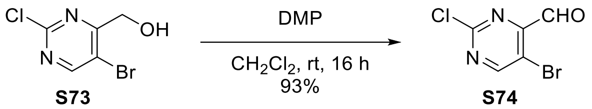

S73 **S74**

S74. To a solution of alcohol **S73** (1.3 g, 5.8 mmol) in CH_2Cl_2 (30 mL) was added Dess-Martin periodinane (2.47 g, 5.8 mmol, 1.0 eq). The mixture was stirred at room temperature for 16 hours, after which the suspension was diluted with CH_2Cl_2 (50 mL). Then, a saturated aqueous solution of $NaHCO_3$ and $Na_2S_2O_3$ (1:1, 40 mL) was added, and the mixture was stirred until the layers separated. The organic layer was dried with $MgSO_4$, then concentrated. The residue was purified by silica gel chromatography (100% CH_2Cl_2) to afford the title compound as a yellow (1.2 g, 5.4 mmol, 93%). ^{1}H NMR (500 MHz, $CDCl_3$) δ 10.02 (d, J = 0.6 Hz, 1H), 8.95 (d, J = 0.6 Hz, 1H). ^{13}C NMR (126 MHz, $CDCl_3$) δ 188.77, 165.08, 160.77, 155.46, 116.53. HRMS-ESI (m/z) Calculated for $C_5H_3BrClN_2O$ $[M+H]^+$, 220.9112; found 220.9109.

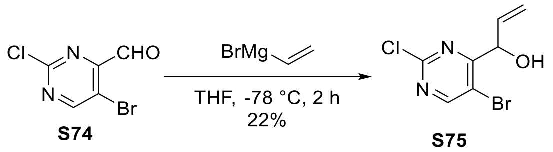

S74 **S75**

S75. Aldehyde **S74** (1.2 g, 5.4 mmol) was dissolved in THF (25 mL), then cooled to -78 °C. Then vinylmagnesium bromide 1.0M in THF (6.5 mL, 6.5 mmol, 1.5 eq) was added slowly at this temperature. The mixture was stirred at to -78 °C for 2 hours, after which it was quenched with sat. ammonium chloride. The mixture was extracted with EtOAc, the combined organic layers dried with $MgSO_4$, then concentrated. The residue was purified by silica gel chromatography (25% pentane in CH_2Cl_2) to afford the title compound as a yellow oil (300 mg, 1.2 mmol, 22%). ^{1}H NMR (500 MHz, $CDCl_3$) δ 8.66 (s, 1H), 5.92 (ddd, J = 17.0, 10.3, 5.8 Hz, 1H), 5.52 (dt, J = 17.1, 1.3 Hz, 1H), 5.43 (ddt, J = 8.9, 5.7, 1.6 Hz, 1H), 5.32 (dt, J = 10.3, 1.2 Hz, 1H), 3.84 (d, J = 9.1 Hz, 1H). ^{13}C NMR (126 MHz, $CDCl_3$) δ 169.73, 161.41, 159.71, 134.98, 118.56, 117.10, 72.39. HRMS-ESI (m/z) Calculated for $C_7H_7BrClN_2O$ $[M+H]^+$, 248.9425; found 248.9425.

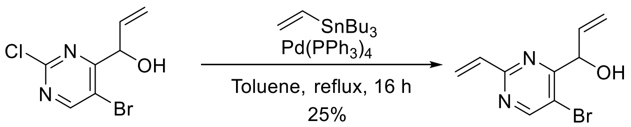

S75 **S76**

S76. Bromide **S75** (300 g, 1.2 mmol) was dissolved in toluene (10 mL) and degassed with nitrogen for 5 minutes. Then, vinyltributyltin (0.35 mL, 1.2 mmol, 1.0 eq) was added and the solution was degassed for a further 5 minutes. Finally, $Pd(PPh_3)_4$ (70 mg, 60 μmol, 5 mol%) was added and the mixture was degassed for a further 5 minutes, then heated at reflux temperature for 16 hours. The reaction mixture was cooled, filtered over celite, then concentrated. The residue was purified by silica gel chromatography with 10% crushed KF (50% to 75% pentane in CH_2Cl_2) to afford the title compound as a clear oil (60 mg, 0.31 mmol,

25%). ^{1}H NMR (600 MHz, $CDCl_3$) δ 8.72 (s, 1H), 6.84 (dd, J = 17.3, 10.5 Hz, 1H), 6.66 (dd, J = 17.3, 1.5 Hz, 1H), 5.98 – 5.92 (m, 1H), 5.81 (dd, J = 10.6, 1.5 Hz, 1H), 5.53 (dt, J = 17.1, 1.4 Hz, 1H), 5.40 (s, 1H), 5.29 (dt, J = 10.3, 1.3 Hz, 1H), 4.40 (d, J = 7.7 Hz, 1H). ^{13}C NMR (151 MHz, $CDCl_3$) δ 165.87, 162.31, 159.32, 135.78, 135.06, 125.49, 117.85, 116.49, 72.03. HRMS-ESI (m/z) Calculated for $C_9H_{10}BrN_2O$ $[M+H]^+$, 240.9971; found 240.9972

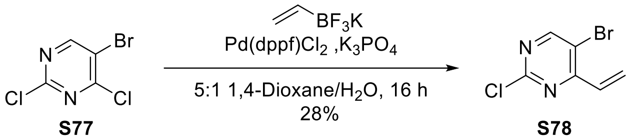

S77 **S78**

S78. Pyrimidine **S77** (2.0 g, 8.8 mmol), potassium vinyltrifluoroborate (1.18 g, 8.8 mmol, 1.0 eq), crushed K_3PO_4 (5.6 g, 26.3 mmol, 3.0 eq) and Pd(dppf)Cl_2 (257 mg, 0.35 mmol, 0.04 eq) were placed in a flask under N_2. Then degassed 5:1 1,4-dioxane:H_2O (40 mL) was added and the reaction was stirred at 85 °C bath temperature for 16 hours. The mixture was then cooled, diluted with EtOAc and water. The organic layer was dried with $MgSO_4$, concentrated and purified by silica gel chromatography (25% CH_2Cl_2 in pentane) to afford the title compound as a pale yellow oil (530 mg, 2.4 mmol, 28%). ^{1}H NMR (600 MHz, $CDCl_3$) δ 8.67 (s, 1H), 7.11 (dd, J = 16.8, 10.6 Hz, 1H), 6.84 (dd, J = 16.8, 1.5 Hz, 1H), 5.89 (dd, J = 10.6, 1.5 Hz, 1H). ^{13}C NMR (151 MHz, $CDCl_3$) δ 162.35, 161.97, 159.75, 130.94, 128.31, 117.78. HRMS-ESI (m/z) Calculated for $C_6H_5BrClN_2$ $[M+H]^+$, 218.9319; found 218.9318

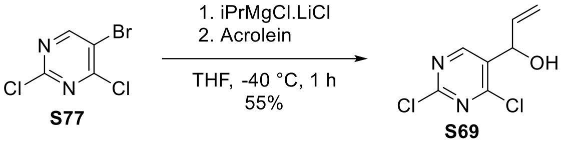

S77 **S69**

S69. Bromide **S77** (1.0 g, 4.4 mmol, 1.0 eq) was dissolved in dry in THF (30 mL) and the mixture was cooled to -40 °C with dry ice/MeCN. Then TurboGrignard 1.3M in THF (3.7 mL, 4.83 mmol, 1.1 eq) was added, and the solution was stirred for 30 minutes at this temperature. Then, freshly distilled acrolein (380 µL, 5.7 mmol, 1.3 eq) was added and the solution was allowed to warm to room temperature for 1 hour. The reaction was quenched with sat. ammonium chloride, then extracted with EtOAc. The combined organic layers were dried with $MgSO_4$, concentrated and purified by silica gel chromatography (0 to 3% EtOAc in CH_2Cl_2) to afford the title compound as a clear oil (494 mg, 2.4 mmol, 55%). ^{1}H NMR (500 MHz, $CDCl_3$) δ 8.76 (s, 1H), 5.96 (ddd, J = 16.6, 10.3, 6.1 Hz, 1H), 5.52 (d, J = 6.1 Hz, 1H), 5.48 (dt, J = 17.1, 1.1 Hz, 1H), 5.35 (dt, J = 10.3, 1.1 Hz, 1H). ^{13}C NMR (126 MHz, $CDCl_3$) δ 160.35, 159.59, 159.09, 136.33, 132.93, 118.42, 69.84. HRMS-ESI (m/z) Calculated for $C_7H_7Cl_2N_2O$ $[M+H]^+$, 204.9930; found 204.9927.

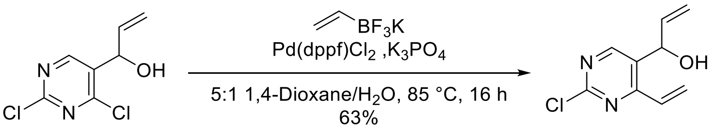

S69 **S79**

S79. Chloride **S69** (250 mg, 1.2 mmol), potassium vinyltrifluoroborate (163 mg, 1.2 mmol, 1.0 eq), crushed K_3PO_4 (776 mg, 3.6 mmol, 3.0 eq) and Pd(dppf)Cl_2 (36 mg, 49 µmol, 0.04 eq) were placed in a flask under N_2. Then degassed 5:1 1,4-dioxane:H_2O (5 mL) was added and the reaction was stirred at 85 °C bath temperature for 16 hours. The mixture was then cooled, diluted with EtOAc and water. The organic layer was dried with $MgSO_4$, concentrated and purified by silica gel chromatography (10% EtOAc in CH_2Cl_2) to afford the title compound as a pale yellow oil (150 mg, 63 mmol, 63%). ^{1}H NMR (600 MHz, $CDCl_3$) δ 8.67 (d, J = 0.6 Hz, 1H), 6.96 (dd, J = 16.8, 10.6 Hz, 1H), 6.74 (dd, J = 16.8, 1.6 Hz, 1H), 6.03 (ddd, J = 17.2, 10.4,

5.8 Hz, 1H), 5.78 (dd, J = 10.6, 1.6 Hz, 1H), 5.47 (dq, J = 5.8, 1.2 Hz, 1H), 5.38 (ddd, J = 17.2, 1.5, 0.9 Hz, 1H), 5.34 (ddd, J = 10.3, 1.3, 0.8 Hz, 1H), 2.04 (s, 1H). ^{13}C NMR (126 MHz, $CDCl_3$) δ 162.22, 160.55, 159.21, 137.82, 129.85, 129.84, 126.68, 117.81, 69.76. HRMS-ESI (m/z) Calculated for $C_9H_{10}ClN_2O$ $[M+H]^+$, 197.0476; found 197.0474.

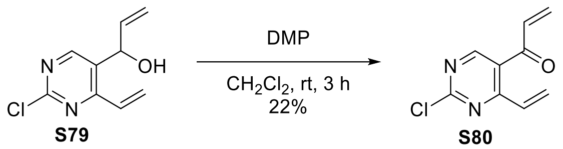

S80. To a solution of alcohol **S79** (144 mg, 702 μmol) in CH_2Cl_2 (5 mL) was added Dess-Martin periodinane (297 mg, 702 μmol, 1.0 eq). The mixture was stirred at room temperature for 3 hours, after which the suspension was diluted with CH_2Cl_2 (20 mL). Then, a saturated aqueous solution of $NaHCO_3$ and $Na_2S_2O_3$ (1:1, 15 mL) was added, and the mixture was stirred until the layers separated. The organic layer was dried with $MgSO_4$, then concentrated. The residue was purified by silica gel chromatography (30% Pentane in CH_2Cl_2) to afford the title compound as a yellow oil (30 mg, 154 μmol, 22%). ^{1}H NMR (600 MHz, $CDCl_3$) δ 8.68 (s, 1H), 6.97 (dd, *J* = 16.8, 10.5 Hz, 1H), 6.84 (dd, *J* = 16.8, 1.6 Hz, 1H), 6.78 (dd, *J* = 17.5, 10.6 Hz, 1H), 6.24 (dd, *J* = 17.4, 0.7 Hz, 1H), 6.18 (dd, *J* = 10.6, 0.6 Hz, 1H), 5.85 (dd, *J* = 10.5, 1.6 Hz, 1H). ^{13}C NMR (151 MHz, $CDCl_3$) δ 191.74, 163.43, 162.51, 159.91, 135.76, 133.88, 130.72, 128.38, 127.85. HRMS-ESI (m/z) Calculated for $C_9H_8ClN_2O$ $[M+H]^+$, 195.0320; found 195.0317.

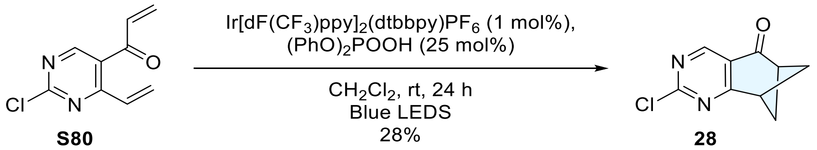

28. S80 (30 mg, 154 μmol) and diphenyl phosphoric acid (9.6 mg, 39 μmol, 0.25 eq) in CH_2Cl_2 (18 mL) was added to a vial charged with Ir[dF(CF_3)ppy]$_2$(dtbbpy)PF_6 (2 mg, 1.5 μmol, 1 mol%) under a N_2 atmosphere. The solution was stirred while irradiating with a blue LED lamp (440 nm) at a distance of 2 cm, at 25 °C using a stream of nitrogen to cool the system. After 24 hours, the reaction was washed with sat. bicarbonate solution. The organic layer was dried with $MgSO_4$, then concentrated. The residue was purified by silica gel chromatography (30% Pentane in CH_2Cl_2) to afford the title compound as a white solid (8.5 mg, 44 μmol, 28%). ^{1}H NMR (600 MHz, $CDCl_3$) δ 9.01 (s, 1H), 3.47 (q, *J* = 5.6 Hz, 1H), 3.31 (td, *J* = 6.0, 5.3 Hz, 1H), 3.09 (dtd, *J* = 7.3, 5.9, 2.7 Hz, 2H), 2.47 – 2.41 (m, 2H). ^{13}C NMR (151 MHz, $CDCl_3$) δ 197.25, 182.12, 163.60, 157.53, 120.10, 49.41, 42.42, 41.40. HRMS-ESI (m/z) Calculated for $C_9H_8ClN_2O$ $[M+H]^+$, 195.0320; found 195.0319.

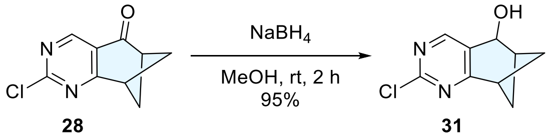

31. Ketone **28** (7 mg, 36 μmol) was dissolved in MeOH (0.4 mL), then sodium borohydride (4.1 mg, 108 μmol, 3.0 eq) was added. The mixture was stirred at room temperature for 2 hours, after which the mixture was diluted with EtOAc. The organic layer was washed with saturated bicarbonate solution, then dried with $MgSO_4$ and concentrated. to afford the title compound as a white gum (6.7 mg, 34 μmol, 95%). ^{1}H NMR (600 MHz, $CDCl_3$) δ 8.64 (s, 1H), 5.09 (d, *J* = 3.5 Hz, 1H), 3.49 (s, 1H), 3.26 (q, *J* = 5.4 Hz, 1H), 2.85 (qd, *J* = 6.0, 3.6 Hz, 1H), 2.71 (dt, *J* = 10.0, 6.1 Hz, 1H), 2.54 (dt, *J* = 10.0, 5.8 Hz, 1H), 1.78 (dd, *J* = 10.0, 8.2 Hz, 1H), 1.70 (dd, *J* = 10.0, 8.2 Hz, 1H). ^{13}C NMR (151 MHz, $CDCl_3$) δ 179.02, 159.35, 159.20, 127.38,

68.38, 42.23, 40.20, 36.80, 29.97. HRMS-ESI (m/z) Calculated for $C_9H_{10}ClN_2O$ $[M+H]^+$, 197.0476; found 197.0475.

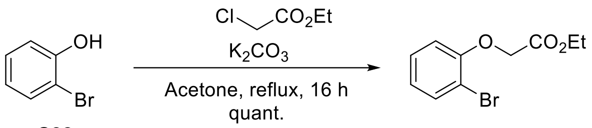

S82 **S83**

S83. 2-bromophenol **S82** (3.00 g, 17.3 mmol) and chloroethyl acetate (2.38 mL, 22.2 mmol, 1.3 eq) in the presence of anhydrous potassium carbonate (7.19 g, 52.0 mmol, 3 eq) was refluxed in dry acetone (35 mL) for 16 h. The reaction mixture was cooled, poured into cold water, then extracted with diethyl ether. The organic layer was washed with 10% sodium hydroxide solution, followed by water, then dried over Na_2SO_4. The solvent was removed under reduced pressure to afford the title compound as an orange oil (4.5 g, 17.3 mmol, 100%). ^{1}H NMR (400 MHz, $CDCl_3$) δ 7.56 (dd, *J* = 7.9, 1.6 Hz, 1H), 7.24 (ddd, *J* = 8.2, 7.4, 1.6 Hz, 2H), 6.89 (td, *J* = 7.7, 1.4 Hz, 1H), 6.81 (dd, *J* = 8.2, 1.4 Hz, 1H), 4.70 (s, 2H), 4.27 (q, *J* = 7.1 Hz, 2H), 1.29 (t, *J* = 7.1 Hz, 3H). Data consistent with that given in the literature. [14]

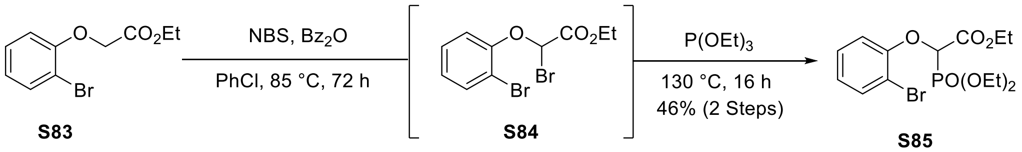

S83 **S84** **S85**

S85. A solution of **S83** (2.00 g, 7.72 mmol) in chlorobenzene (40 mL) was degassed with N_2 for 10 minutes. N-bromosuccinimide (1.51 g, 8.49 mmol, 1.1 eq) and dibenzoyl peroxide (187 mg, 0.772 mmol, 10 mol%) were added and the solution degassed for a further 10 minutes before heating to 85 °C for 72 hours. The reaction mixture was cooled, filtered, dried with Na_2SO_4 and the solvent removed under reduced pressure to afford crude **S84** as a brown oil in (2.72 g, 69% purity, 5.36 mmol; 31% of **S83**). This was left crude due to instability to silica. ^{1}H NMR (400 MHz, $CDCl_3$) δ 7.63 – 7.59 (m, 1H), 7.36 – 7.33 (m, 1H), 7.31 – 7.27 (m, 1H), 7.09 (ddd, *J* = 7.9, 7.3, 1.6 Hz, 1H), 6.46 (s, 1H), 4.41 (qd, *J* = 7.1, 2.3 Hz, 2H), 1.39 (t, *J* = 7.1 Hz, 3H). The residue was mixed with triethylphosphite (1.19 mL, 6.96 mmol) and heated to 130 ºC for 22 hours. After cooling to room temperature, the crude product was purified by silica gel chromatography (0 to 20% EtOAc in CH_2Cl_2) to yield the title compound as a pale yellow oil (1.4 g, 3.54 mmol, 46% over 2 steps). ^{1}H NMR (600 MHz, $CDCl_3$) δ 7.56 (dd, *J* = 7.9, 1.6 Hz, 1H), 7.25 – 7.21 (m, 1H), 6.91 (td, *J* = 7.6, 1.3 Hz, 1H), 6.80 (dd, *J* = 8.3, 1.3 Hz, 1H), 5.08 (d, *J* = 18.4 Hz, 1H), 4.41 – 4.33 (m, 4H), 4.30 (qd, *J* = 7.1, 5.0 Hz, 2H), 1.39 (t, *J* = 7.1 Hz, 6H), 1.28 (t, *J* = 7.1 Hz, 3H). ^{31}P NMR (243 MHz, $CDCl_3$) δ 11.58. ^{13}C NMR (151 MHz, $CDCl_3$) δ 166.13, 154.32 (d, *J* = 13.6 Hz), 134.12, 128.55, 123.83, 113.92, 112.68, 75.33 (d, *J* = 155.9 Hz), 64.64 (dd, *J* = 6.5, 4.4 Hz), 62.48, 16.58 (dd, *J* = 6.3, 3.0 Hz), 14.18. HRMS-ESI (m/z) Calculated for $C_{14}H_{21}BrO_6P$ [M+H]+, 395.0254; found 395.0246.

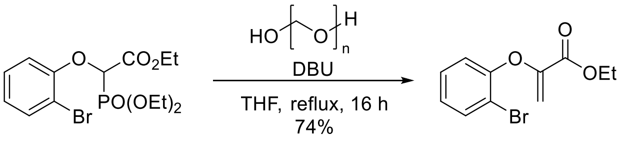

S85 **S86**

S86. A suspension of **S85** (590 mg, 1.49 mmol) and DBU (0.58 mL, 3.73 mmol, 2.5 eq) in anhydrous THF (15 mL) was stirred for 10 minutes at room temperature. To this mixture was added paraformaldehyde (67.1 mg, 2.24 mmol, 1.5 eq) and the mixture was heated at reflux temperature for 16 hours. The solvent was removed under reduced pressure and the crude

residue dissolved in EtOAc. The organic layer was washed with saturated aqueous NH_4Cl solution, brine, dried with $MgSO_4$, filtered and the solvent removed under reduced pressure. The residue was purified by silica gel chromatography (5% EtOAc in pentane) to yield the title compound as a colourless oil (302 mg, 1.1 mmol, 74%). ^{1}H NMR (500 MHz, $CDCl_3$) δ 7.61 (dd, *J* = 8.4, 1.6 Hz, 1H), 7.30 (td, *J* = 7.6, 1.6 Hz, 1H), 7.08 – 7.02 (m, 2H), 5.69 (d, *J* = 2.4 Hz, 1H), 4.76 (d, *J* = 2.4 Hz, 1H), 4.31 (q, *J* = 7.1 Hz, 2H), 1.32 (t, *J* = 7.1 Hz, 3H). ^{13}C NMR (126 MHz, $CDCl_3$) δ 162.33, 152.27, 150.11, 134.07, 128.86, 126.11, 121.20, 115.14, 103.36, 61.91, 14.27. HRMS-ESI (m/z) Calculated for $C_{11}H_{12}BrO_3$ $[M+H]^+$, 270.9964; found 270.9960.

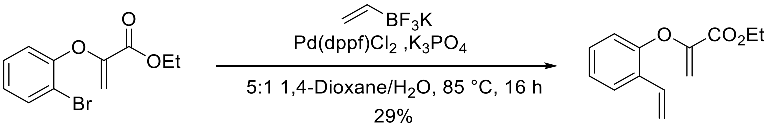

S86 **S87**

S87. Bromide **S86** (150 mg, 553 µmol), potassium trifluoro(vinyl)borate (74.1 mg, 553 µmol, 1.0 eq), K_3PO_4 (352 mg, 1.66 mmol, 3.0 eq) and $Pd(dppf)Cl_2$ (16.2 mg, 22.1 µmol, 4 mol%) were placed in a flask under N_2. Degassed 5:1 1,4-dioxane/ H_2O (2.4 mL) was added and the reaction mixture stirred at 85 ºC for 16 hours. The reaction mixture was cooled, diluted with ethyl acetate and washed with water. The organic layer was dried with $MgSO_4$ and the solvent removed under reduced pressure. The residue was purified by silica gel chromatography (2 to 3% EtOAc in pentane) to yield the title compound as a colourless oil (34.8 mg, 160 µmol, 29%). ^{1}H NMR (600 MHz, $CDCl_3$) δ 7.61 (dd, *J* = 7.8, 1.7 Hz, 1H), 7.27 (dd, *J* = 7.6, 1.6 Hz, 1H), 7.20 – 7.16 (m, 1H), 7.00 (dd, *J* = 8.1, 1.2 Hz, 1H), 6.91 (dd, *J* = 17.7, 11.1 Hz, 1H), 5.80 (dd, *J* = 17.7, 1.2 Hz, 1H), 5.61 (d, *J* = 2.3 Hz, 1H), 5.32 (dd, *J* = 11.1, 1.2 Hz, 1H), 4.66 (d, *J* = 2.3 Hz, 1H), 4.34 (q, *J* = 7.1 Hz, 2H), 1.35 (t, *J* = 7.1 Hz, 3H). ^{13}C NMR (151 MHz, $CDCl_3$) δ 162.76, 152.19, 151.20, 130.73, 129.91, 129.13, 126.77, 125.17, 120.57, 115.78, 102.03, 61.81, 14.29. HRMS-ESI (m/z) Calculated for $C_{13}H_{15}O_3$ $[M+H]^+$, 219.1016; found 219.1013.

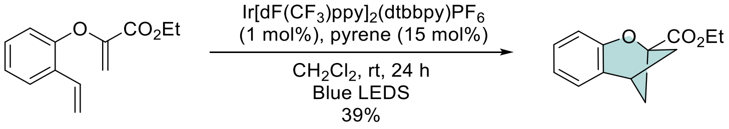

S87 **29**

29. **S87** (49.6 mg, 243 µmol) in CH_2Cl_2 (20 mL) was added to a vial charged with $Ir[dF(CF_3)ppy]_2(dtbbpy)PF_6$ (3 mg, 2.4 µmol, 1 mol%) and pyrene (7.4 mg, 36.4 µmol) under a N_2 atmosphere. The solution was stirred while irradiating with a blue LED lamp (440 nm) at a distance of 2 cm, at 25 °C using a stream of nitrogen to cool the system. After 24 hours, the reaction was washed with sat. bicarbonate solution. The organic layer was dried with $MgSO_4$, then concentrated. The residue was purified by silica gel chromatography (5% EtOAc in pentane) to afford the title compound as a white solid (19.5 mg, 95 µmol, 39%). ^{1}H NMR (600 MHz, $CDCl_3$) δ 7.16 (dd, *J* = 7.8, 1.6 Hz, 1H), 7.05 (dd, *J* = 7.5, 1.5 Hz, 1H), 7.01 (d, *J* = 8.0 Hz, 1H), 6.85 (td, *J* = 7.4, 1.1 Hz, 1H), 4.32 (q, *J* = 7.1 Hz, 2H), 3.34 (t, *J* = 5.5 Hz, 1H), 2.78 (ddd, *J* = 7.0, 5.6, 2.7 Hz, 2H), 1.79 – 1.73 (m, 2H), 1.36 (t, *J* = 7.1 Hz, 3H). ^{13}C NMR (151 MHz, $CDCl_3$) δ 169.47, 152.02, 132.96, 127.81, 124.94, 120.25, 115.40, 83.43, 61.77, 34.79, 34.25, 14.34. HRMS-ESI (m/z) Calculated for $C_{13}H_{15}O_3$, 219.1016; found 219.1014.

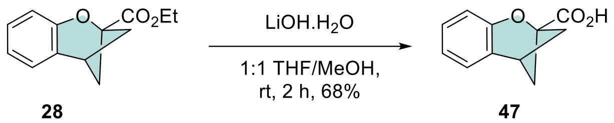

28 **47**

47. 28. (11.2 mg, 54.8 μmol) was dissolved in 1:1 THF/MeOH (0.5 mL). Lithium hydroxide monohydrate (5.75 mg, 137 μmol, 2.5 eq) dissolved in water (0.25 mL) was added and the reaction stirred at room temperature for 3 hours. The reaction mixture was diluted with $CHCl_3$ and acidified with dilute aqueous HCl until the pH was approximately 3. The organic layer was dried with $MgSO_4$, filtered, and the solvent removed under reduced pressure to yield the desired product as a white solid (7.1 mg, 37.3 μmol, 68%) without the need for further purification. ^{1}H NMR (600 MHz, $CDCl_3$) δ 7.19 (ddd, *J* = 8.1, 7.5, 1.6 Hz, 1H), 7.09 (dd, *J* = 7.4, 1.6 Hz, 1H), 7.02 – 6.99 (m, 1H), 6.90 (td, *J* = 7.4, 1.1 Hz, 1H), 3.39 (t, *J* = 5.5 Hz, 1H), 2.90 – 2.85 (m, 2H), 1.83 – 1.74 (m, 2H). ^{13}C NMR (151 MHz, $CDCl_3$) δ 171.68, 151.42, 132.65, 128.04, 125.33, 120.93, 115.20, 83.21, 35.04, 34.28. HRMS-ESI (m/z) Calculated for $C_{11}H_{10}O_3Na$ [M+Na]+, 213.0522; found 213.0518.

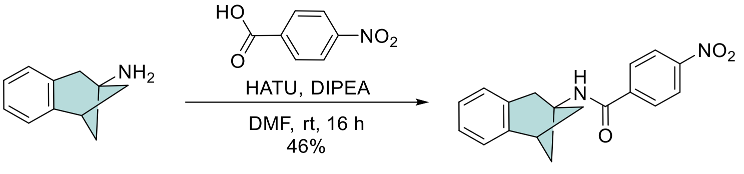

37 **S89**

S89. Amine **37** (1.8 mg, 11.3 μmol). 4-nitrobenzoic acid (1.89 mg, 11.3 μmol, 1.0 eq), HATU (4.3 mg, 11.3 μmol, 1.0 eq) and DIPEA (5.9 μL, 33.9 μmol, 3.0 eq) were dissolved in DMF (0.2 mL) and stirred for at room temperature 16 hours. The mixture was diluted with EtOAc, then washed sequentially with sat. Bicarb solution, 1N HCl, then brine. The organic layer was dried with $MgSO_4$, filtered, then concentrated. The residue was purified by preparative TLC (100% CH2Cl2) to afford the title compound as a white/yellow solid (1.6 mg, 5.2 μmol, 46%). ^{1}H NMR (400 MHz, $CDCl_3$) δ 8.31 – 8.23 (m, 2H), 7.54 – 7.44 (m, 2H), 7.19 – 7.11 (m, 2H), 7.10 – 7.01 (m, 2H), 3.25 (s, 2H), 3.11 (t, *J* = 6.3 Hz, 1H), 2.61 (td, *J* = 6.2, 2.5 Hz, 2H), 1.91 – 1.84 (m, 2H). ^{13}C NMR (151 MHz, $CDCl_3$) δ 168.62, 147.74, 146.04, 138.97, 134.68, 128.48, 126.72, 126.11, 125.49, 125.02, 124.19, 55.05, 53.56, 40.80, 40.54, 34.91. HRMS-ESI (m/z) Calculated for $C_{18}H_{17}N_2O_3$ $[M+H]^+$, 309.1234; found 309.1667.

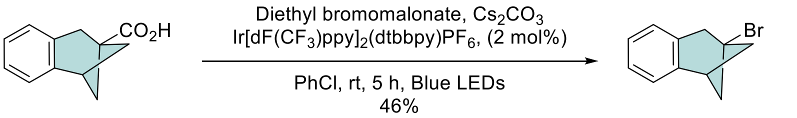

36 **38**

38. According to the procedure of Glorius, to a mixture of acid **36** (50 mg, 0.27 mmol), Ir[dF(CF3)ppy]$_2$(dtbbpy)PF$_6$ (5 mg, 4 μmol) and Cs_2CO_3 (85 mg, 0.26 mmol) in chlorobenzene (5 mL) was added diethyl bromomalonate (0.12 mL, 0.67 mmol). The resulting mixture was degassed with nitrogen for 10 min and then irradiated with a blue LED lamp (467 nm) at a distance of 2 cm, at 25 °C using a stream of nitrogen to cool the system. After 5 hours, the reaction was filtered and volatiles evaporated *in vacuo*. The residue was purified by silica gel chromatography (0 to 10% EtOAc in pentane) to afford the title compound as a colourless oil (26 mg, 0.12 mmol, 46%). ^{1}H NMR (600 MHz, $CDCl_3$) δ 7.22 – 7.14 (m, 2H), 7.12 – 7.07 (m, 1H), 7.04 – 6.99 (m, 1H), 3.59 (s, 2H), 3.32 (t, J = 6.8 Hz, 1H), 2.97 – 2.87 (m, 2H), 2.19 – 2.13 (m, 2H); ^{13}C NMR (151 MHz, $CDCl_3$) δ 144.3, 134.6, 127.9, 126.6, 125.8, 125.2, 56.9, 46.7, 46.0, 38.7.

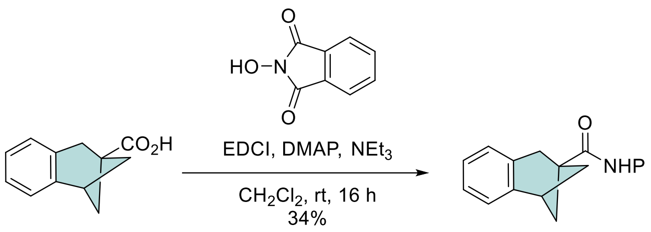

34 39

39. To a solution of acid **34** (30 mg, 0.16 mmol) EDCI (40 mg, 0.21 mmol, 1.3 eq), DMAP (2 mg, 16 µmol, 10 mol%) and CH_2Cl_2 (2.0 mL) was added *N*-hydroxyphthalimide (34 mg, 0.21 mmol, 1.3 eq) and triethylamine (70 µL, 0.48 mmol, 3.0 eq). The resulting mixture was allowed to stir at room temperature for 16 h. The mixture was diluted with CH_2Cl_2 (5 mL), and the organic layer was washed with aqueous HCl (1 M, 5 mL) and dried over anhydrous $MgSO_4$. The solution was concentrated and purified by silica gel chromatography (0 to 100% EtOAc in pentane) to afford the title compound as a white solid (18 mg, 54 µmol, 34%). ^{1}H NMR (600 MHz, $CDCl_3$) δ 7.93 – 7.89 (m, 2H), 7.83 – 7.79 (m, 2H), 7.27 – 7.24 (m, 1H), 7.21 (td, J = 7.4, 1.4 Hz, 1H), 7.12 (t, J = 8.1 Hz, 1H), 7.07 (dd, J = 7.5, 1.4 Hz, 1H), 3.49 (s, 2H), 3.16 (t, J = 5.9 Hz, 1H), 2.98 – 2.88 (m, 2H), 1.95 – 1.89 (m, 2H). ^{13}C NMR (151 MHz, $CDCl_3$) δ 170.74, 162.16, 145.48, 134.91, 132.24, 129.20, 128.74, 126.89, 125.87, 125.42, 124.13, 44.64, 37.58, 36.11, 35.79.δ 171.88, 153.91, 145.74, 142.09, 135.13, 132.71, 128.79, 126.86, 125.87, 125.44, 125.33, 121.18, 111.47, 51.21, 41.84, 38.93, 36.78, 36.39, 7.77. HRMS-ESI (m/z) Calculated for $C_{20}H_{16}NO_4$ $[M+H]^+$, 334.1074; found 334.1074.

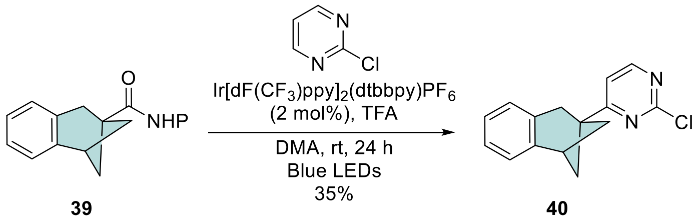

39 40

40. 39 (65 mg, 0.20 mmol), 2-chloropyrimidine (46 mg, 0.40 mmol, 2.0 eq), and TFA (30 µL, 0.40 mmol, 2 eq) in DMA (2 mL) were added to a vial charged with $Ir[dF(CF_3)ppy]_2(dtbbpy)PF_6$ (4 mg, 4 µmol, 2 mol%) under a N_2 atmosphere. The solution was degassed with nitrogen for 10 min and then irradiated with a blue LED lamp (440 nm) at a distance of 2 cm, at 25 °C using a stream of nitrogen to cool the system. After 24 hours, the reaction was quenched with triethylamine (0.10 mL), diluted with water (10 mL) and extracted with EtOAc. The organic layer was washed with water (10 mL), dried with Na_2SO_4, then concentrated. The residue was purified by silica gel chromatography (0 to 20% EtOAc in pentane) to afford the title compound as a white solid (18 mg, 70 µmol, 35%). ^{1}H NMR (600 MHz, $CDCl_3$) δ 8.64 (d, J = 5.1 Hz, 1H), 7.42 (d, J = 5.1 Hz, 1H), 7.24 – 7.20 (m, 1H), 7.18 – 7.13 (m, 1H), 7.09 – 7.06 (m, 2H), 3.26 (s, 2H), 3.15 (t, J = 5.9 Hz, 1H), 2.71 – 2.65 (m, 2H), 1.89 – 1.83 (m, 2H). ^{13}C NMR (151 MHz, $CDCl_3$) δ 179.73, 162.32, 161.30, 147.64, 134.74, 129.47, 127.42, 126.56, 126.00, 117.31, 40.36, 39.31, 36.93. HRMS-ESI (m/z) Calculated for $C_{15}H_{14}{}^{35}ClN_2$ $[M+H]^+$, 257.0840; found 257.0848.

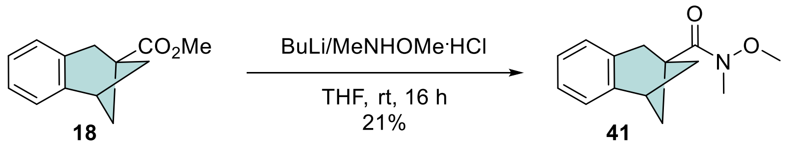

18 41

41. The mixture was stirred until the suspension was dissolved. Then Ester (150 mg, 0.74 mmol) in THF (4 mL) was added, and the solution was stirred at room temperature for 16 hours. The mixture was quenched with ammonium chloride, then extracted with EtOAc. The

combined organic layer was dried with $MgSO_4$. The solution was concentrated and purified by silica gel chromatography (80% to 100% CH_2Cl_2 in pentane) to afford the title compound as a white solid (53 mg, 0.23 mmol, 21%). ^{1}H NMR (600 MHz, $CDCl_3$) δ 7.18 – 7.17 (m, 1H), 7.17 – 7.15 (m, 1H), 7.10 – 7.06 (m, 1H), 7.04 – 7.02 (m, 1H), 3.66 (s, 3H), 3.25 (s, 2H), 3.22 (s, 3H), 3.00 (t, *J* = 5.8 Hz, 1H), 2.61 – 2.55 (m, 2H), 1.85 – 1.80 (m, 2H). ^{13}C NMR (151 MHz, $CDCl_3$) δ 177.07, 146.66, 133.18, 128.58, 126.40, 125.49, 125.20, 61.41, 47.59, 37.95, 36.28, 35.07, 32.89. HRMS-ESI (m/z) Calculated for $C_{14}H_{18}NO_2$ $[M+H]^+$, 232.1332; found 232.1327.

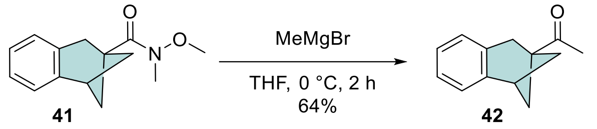

42. Amide **41** (60 mg, 259.41 µmol) was dissolved in THF (2 mL) under N_2 and cooled to 0°C. MeMgBr (3M in diethyl ether, 0.35 mL, 1.04 mmol, 4.0 eq) was added over 10 mins. The reaction mixture was stirred for 2 hours at 0°C. Then, the mixture was quenched with NH_4Cl, extracted with ethyl acetate and concentrated. The residue was purified by silica gel column chromatography (3% ethyl acetate in pentane) to afford the title compound as a clear oil (31 mg, 166.98 µmol, 64%). ^{1}H NMR (600 MHz, $CDCl_3$) δ 7.23 – 7.16 (m, 2H), 7.10 (td, *J* = 7.2, 1.5 Hz, 1H), 7.05 (dd, *J* = 7.4, 1.4 Hz, 1H), 3.18 (s, 2H), 3.02 (t, *J* = 5.8 Hz, 1H), 2.54 (td, *J* = 6.0, 2.5 Hz, 2H), 2.19 (s, 3H), 1.71 – 1.65 (m, 2H). ^{13}C NMR (151 MHz, $CDCl_3$) δ 211.25, 146.48, 133.01, 128.73, 126.57, 125.68, 125.29, 53.13, 36.82, 35.96, 34.84, 24.82. HRMS-ESI (m/z) Calculated for $C_{13}H_{15}O$ $[M+H]^+$, 187.1117; found 187.1115.

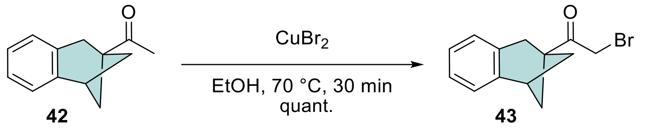

43. Ketone **42** (31 mg, 166.4 µmol) was dissolved in EtOH (0.5 mL). Copper dibromide (69.2 mg, 310 µmol, 1.85 eq) was added to the reaction mixture at room temperature. The mixture was stirred at 70°C for 30 minutes, then cooled to room temperature. The reaction mixture was tdiluted with water, then extracted with ethyl acetate. The organic layer was washed with $NaHCO_3$, followed by brine. The solution was then concentrated and left crude for the next step without further purification. ^{1}H NMR (600 MHz, $CDCl_3$) δ 7.22 – 7.20 (m, 1H), 7.18 (dd, *J* = 7.5, 1.4 Hz, 1H), 7.11 (td, *J* = 7.2, 1.8 Hz, 1H), 7.06 – 7.04 (m, 1H), 4.11 (s, 2H), 3.28 (s, 2H), 3.07 (t, *J* = 5.9 Hz, 1H), 2.64 (td, *J* = 6.1, 2.6 Hz, 2H), 1.80 – 1.75 (m, 2H). ^{13}C NMR (151 MHz, $CDCl_3$) δ 203.33, 145.94, 132.44, 128.69, 126.78, 125.87, 125.37, 51.88, 37.58, 36.32, 35.37, 30.93. HRMS-ESI (m/z) Calculated for $C_{13}H_{14}OBr$ $[M+H]^+$, 265.0223; found 265.0575.

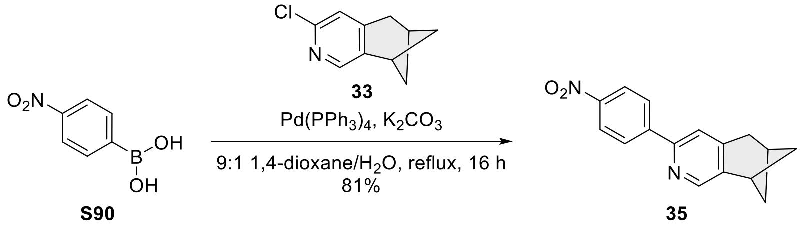

35. Chloride **33** (10 mg, 55.7 µmol, 1.0 eq), boronic acid **S90** (12 mg, 72.4 µmol, 1.3 eq), $Pd(PPH_3)_4$ (6.43 mg, 5.6 µmol, 0.1 eq), potassium carbonate (23 mg, 167 µmol, 3.0 eq) were placed in a microwave vial under N_2. Then, degassed 9:1 1,4-dioxane/water was added (0.5 mL), and the suspension was degassed for a further 5 minutes. The reaction was stirred at reflux temperature for 16 hours, then diluted in EtOAc. The organic layer dried with $MgSO_4$,

concentrated and purified by silica gel chromatography (20% pentane in CH_2Cl_2) to afford the title compound as an off-white solid (12 mg, 45 μmol, 81%). ^1H NMR (600 MHz, $CDCl_3$) δ 8.35 (s, 1H), 8.33 – 8.30 (m, 2H), 8.16 – 8.13 (m, 2H), 7.62 (d, J = 1.0 Hz, 1H), 3.24 (q, J = 5.4 Hz, 1H), 3.14 (dd, J = 2.8, 0.9 Hz, 2H), 2.87 (ddq, J = 8.8, 5.8, 2.8 Hz, 1H), 2.55 (qd, J = 6.1, 2.7 Hz, 2H), 1.51 – 1.46 (m, 2H). ^{13}C NMR (151 MHz, $CDCl_3$) δ 153.23, 148.01, 145.53, 144.84, 144.25, 127.57, 124.15, 121.85, 36.51, 34.94, 34.27, 33.34. HRMS-ESI (m/z) Calculated for $C_{16}H_{15}N_2O_2$ $[M+H]^+$, 267.1128; found 267.1127.

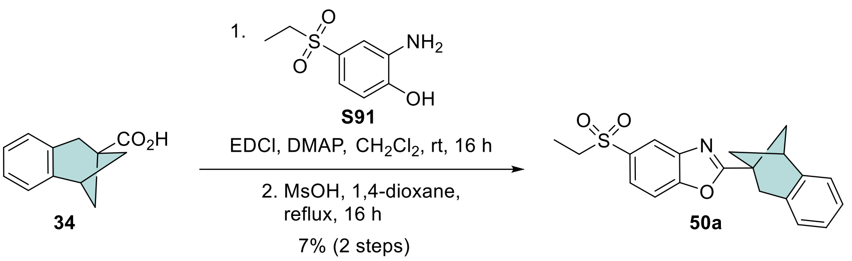

50a. To a solution of acid **34** (40 mg, 208 μmol) EDCI (45 mg, 235 μmol, 1.1 eq), DMAP (29 mg, 235 μmol, 1.1 eq) and CH_2Cl_2 (1.5 mL) was added aminophenol **S91** (43 mg, 208 μmol, 1.0 eq). The resulting mixture was allowed to stir at room temperature for 16 h. The mixture was diluted with CH_2Cl_2 (20 mL), and the organic layer was washed with aqueous HCl (1 M, 10 mL) and dried over anhydrous $MgSO_4$. After removal of the solvent, the residue was redissolved in 1,4-dioxane (1.5mL) followed by addition of MsOH (166 μL). The resulting mixture was allowed to stir at 90 ˚C for 16 h. The reaction was diluted in CH_2Cl_2, then basified with NaHCO3.The organic phase was ried over anhydrous $MgSO_4$. The solution was concentrated and purified by silica gel chromatography (30% EtOAc in pentane) to afford the title compound as a white solid (7 mg, 20 μmol, 7%). ^1H NMR (600 MHz, $CDCl_3$) δ 8.30 (d, J = 1.3 Hz, 1H), 7.93 (dd, J = 8.5, 1.3 Hz, 1H), 7.70 (d, J = 8.5 Hz, 1H), 7.30 (d, J = 7.5 Hz, 1H), 7.24 (t, J = 7.2 Hz, 1H), 7.15 (t, J = 7.4 Hz, 1H), 7.11 (d, J = 7.3 Hz, 1H), 3.60 (s, 2H), 3.25 (t, J = 5.9 Hz, 1H), 3.16 (q, J = 7.4 Hz, 2H), 2.99 (td, J = 6.0, 2.5 Hz, 2H), 1.99 (dt, J = 6.5, 3.9 Hz, 2H), 1.29 (t, J = 6.5 Hz, 3H). ^{13}C NMR (151 MHz, $CDCl_3$) δ 171.88, 153.91, 145.74, 142.09, 135.13, 132.71, 128.79, 126.86, 125.87, 125.44, 125.33, 121.18, 111.47, 51.21, 41.84, 38.93, 36.78, 36.39, 7.77. HRMS-ESI (m/z) Calculated for $C_{20}H_{20}NO_3S$ $[M+H]^+$, 354.1153; found 354.1158.

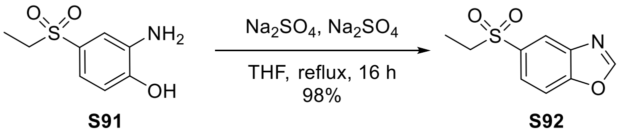

S92. Aniline **S91** (1.5 g, 7.45 mmol) was dissolved in THF (10 mL), then triethyl orthoformate (2.45 mL, 22.36 mmol, 3.0 eq) and Na_2SO_4 (211 mg, 1.49 mmol, 0.2 eq) were added and the mixture was stirred at reflux temperature for 16 hours. The mixture was diluted with EtOAc, then washed with water and brine. The organic layer was dried and concentrated to afford the title compound as a brown solid (1.55 g, 7.3 mmol, 98%). ^1H NMR (600 MHz, $CDCl_3$) δ 8.40 (d, J = 1.7 Hz, 1H), 8.26 (s, 1H), 7.99 (dd, J = 8.6, 1.8 Hz, 1H), 7.78 (d, J = 8.5 Hz, 1H), 3.18 (q, J = 7.4 Hz, 2H), 1.30 (t, J = 7.4 Hz, 3H). ^{13}C NMR (151 MHz, $CDCl_3$) δ 154.70, 153.10, 140.77, 135.70, 126.11, 122.14, 112.14, 51.16, 7.73. HRMS-ESI (m/z) Calculated for $C_9H_9NO_3SNa$ $[M+Na]^+$, 234.0195; found 234.0197.

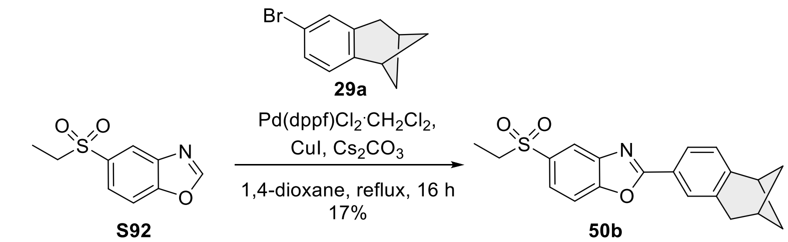

50b. Benzoxazole **S92** (40 mg, 189 µmol), bromide **29a** (42.3 mg, 189 µmol, 1.0 eq), $Pd(dppf)Cl_2 \cdot CH_2Cl_2$ (15.5 mg, 19 µmol, 0.1 eq), CuI (1.8 mg, 9.5 µmol, 0.05 eq) and Cs_2CO_3 (123.4 mg, 379 µmol, 2.0 eq) were placed in a microwave vial and purged with N_2 for 10 minutes. Then, degassed 1,4-dioxane (1.25 mL) was added and the mixture was stirred at reflux temperature for 16 hours. Then, water was added, and the mixture was extracted with CH_2Cl_2. The combined organic layers were concentrated and purified by silica gel chromatography (20% EtOAc in pentane) to afford the title compound as a white solid (11.2 mg, 31.7 µmol, 17%). ^{1}H NMR (400 MHz, $CDCl_3$) δ 8.30 (dd, *J* = 1.7, 0.6 Hz, 1H), 8.07 (d, *J* = 1.7 Hz, 1H), 7.96 (ddt, *J* = 7.7, 1.6, 0.8 Hz, 1H), 7.91 (dd, *J* = 8.4, 1.8 Hz, 1H), 7.72 (dd, *J* = 8.5, 0.6 Hz, 1H), 7.16 (d, *J* = 7.8 Hz, 1H), 3.22 – 3.14 (m, 5H), 2.86 (ddq, *J* = 8.8, 5.9, 2.9 Hz, 1H), 2.49 (qd, *J* = 6.1, 2.6 Hz, 2H), 1.53 – 1.47 (m, 2H), 1.31 (t, *J* = 7.4 Hz, 3H). ^{13}C NMR (151 MHz, $CDCl_3$) δ 166.19, 153.91, 153.56, 143.07, 135.23, 135.01, 128.50, 125.96, 125.31, 125.13, 124.14, 120.82, 111.31, 51.20, 40.05, 34.95, 34.28, 33.68, 7.77. HRMS-ESI (m/z) Calculated for $C_{20}H_{19}NO_3SNa$ $[M+Na]^+$, 376.0978; found 376.0981.

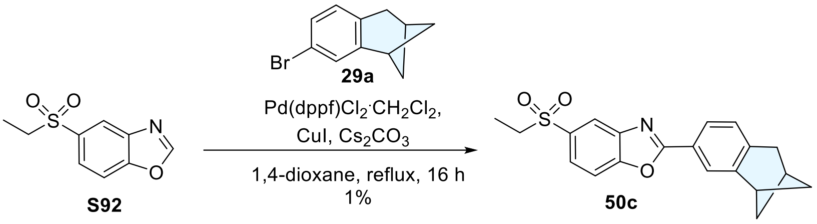

50c. Benzoxazole **S92** (40 mg, 189 µmol), bromide **29a** (42.3 mg, 189 µmol, 1.0 eq), $Pd(dppf)Cl_2 \cdot CH_2Cl_2$ (15.5 mg, 19 µmol, 0.1 eq), CuI (1.8 mg, 9.5 µmol, 0.05 eq) and Cs_2CO_3 (123.4 mg, 379 µmol, 2.0 eq) were placed in a microwave vial and purged with N_2 for 10 minutes. Then, degassed 1,4-dioxane (1.25 mL) was added and the mixture was stirred at reflux temperature for 16 hours. Then, water was added, and the mixture was extracted with CH_2Cl_2. The combined organic layers were dried with $MgSO_4$, concentrated and purified by silica gel chromatography (25% EtOAc in pentane), then by reverse phase chromatography (0-100% MeCN in H_2O with 0.1% formic acid) to afford the title compound as a white solid (0.7 mg, 1.9 µmol, 1%). ^{1}H NMR (600 MHz, $CDCl_3$) δ 8.30 (d, *J* = 1.8 Hz, 1H), 8.06 (dd, *J* = 7.8, 1.8 Hz, 1H), 7.91 (dd, *J* = 8.5, 1.8 Hz, 1H), 7.89 (d, *J* = 1.8 Hz, 1H), 7.72 (d, *J* = 8.5 Hz, 1H), 7.33 (d, *J* = 7.8 Hz, 1H), 3.24 (q, *J* = 5.5 Hz, 1H), 3.18 (q, *J* = 7.4 Hz, 2H), 3.15 (d, *J* = 2.9 Hz, 2H), 2.85 (qt, *J* = 5.8, 2.8 Hz, 1H), 2.50 (qd, *J* = 6.0, 2.6 Hz, 2H), 1.52 – 1.48 (m, 3H), 1.31 (t, *J* = 7.4 Hz, 3H). ^{13}C NMR (151 MHz, $CDCl_3$) δ 166.21, 153.90, 149.15, 143.05, 139.56, 135.22, 129.57, 126.22, 125.16, 124.55, 122.93, 120.85, 111.32, 51.20, 39.82, 35.42, 34.27, 33.66, 7.77. HRMS-ESI (m/z) Calculated for $C_{20}H_{19}NO_3SNa$ $[M+Na]^+$, 376.0978; found 376.0979.

4. NMR Spectra

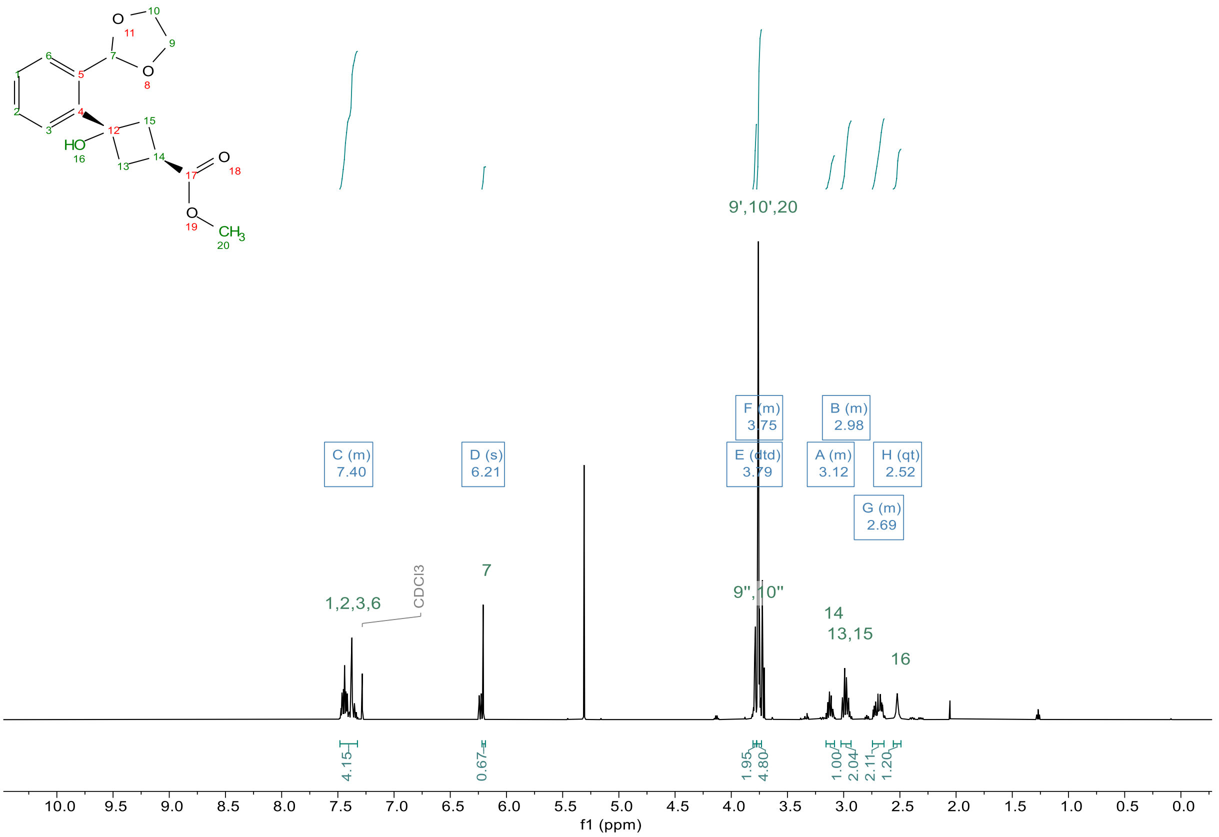

Fig.S1. ^{1}H NMR Spectrum of **S2** (Chloroform-d, 298 K).

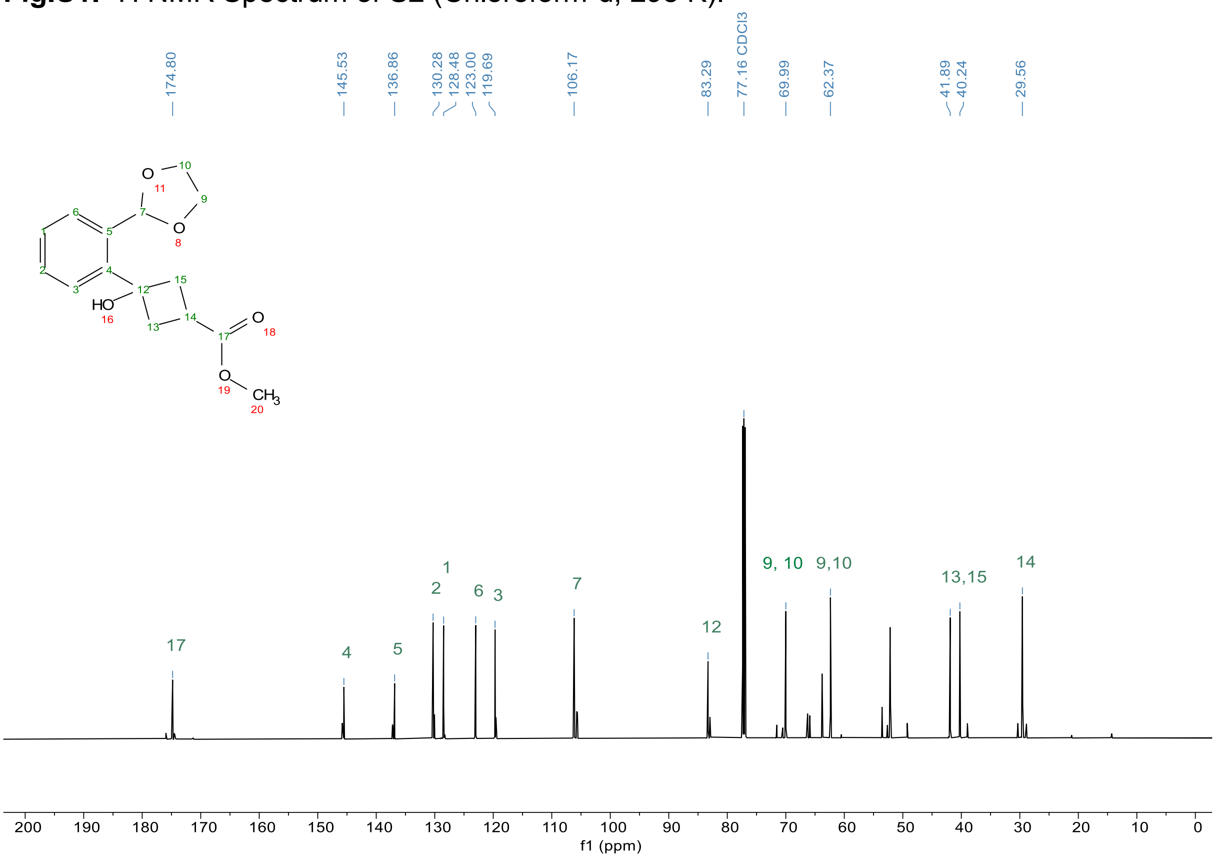

Fig.S2. ^{13}C NMR Spectrum of **S2** (Chloroform-d, 298 K).

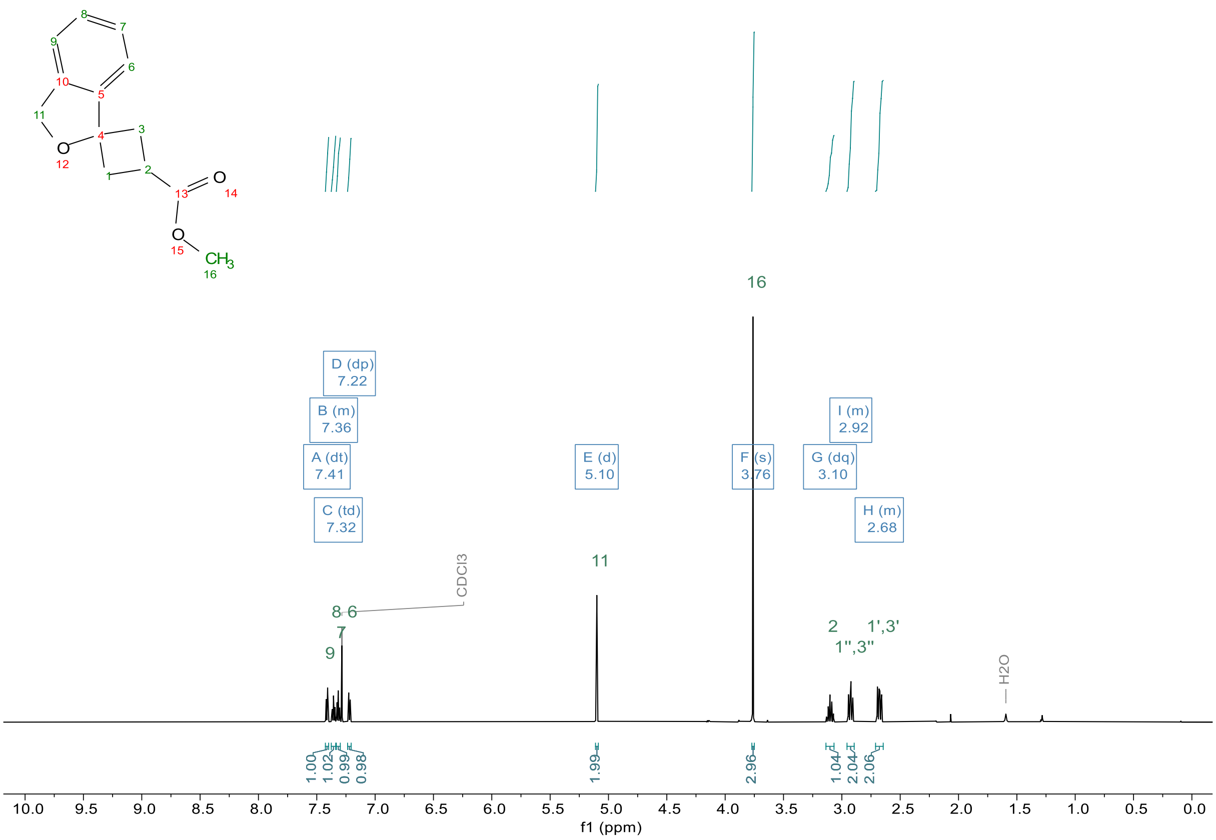

Fig.S3. ¹H NMR Spectrum of **S4** (Chloroform-d, 298 K).

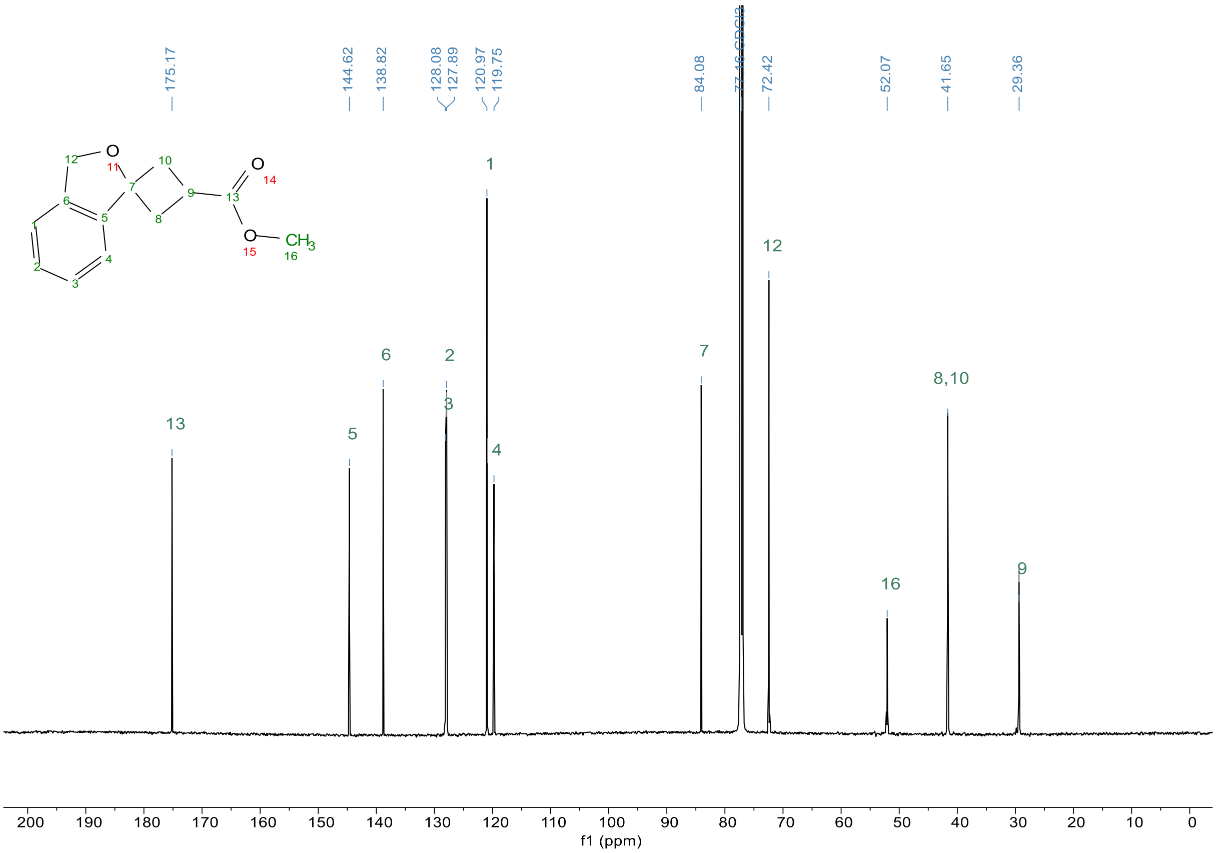

Fig.S4. ¹H NMR Spectrum of **S4** (Chloroform-d, 298 K).

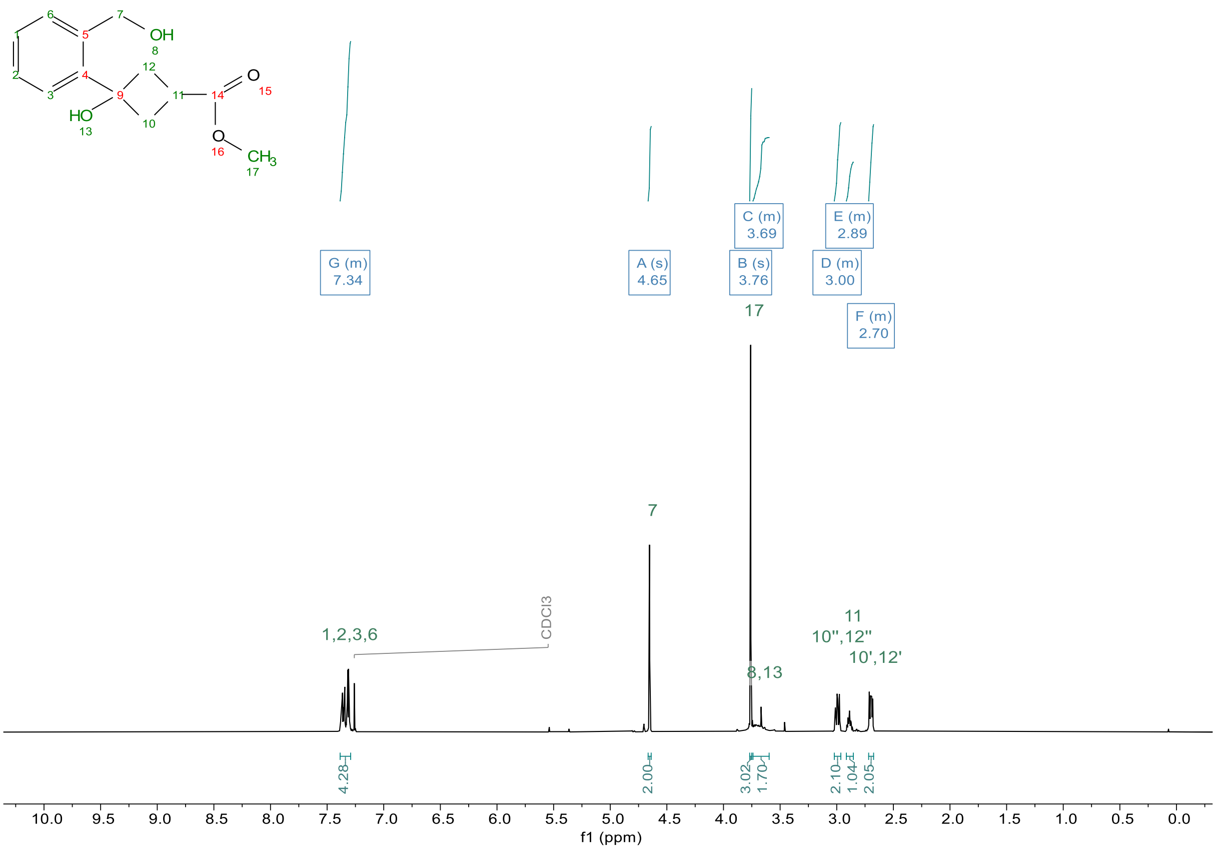

Fig.S5. ^{1}H NMR Spectrum of **S7** (Chloroform-d, 298 K).

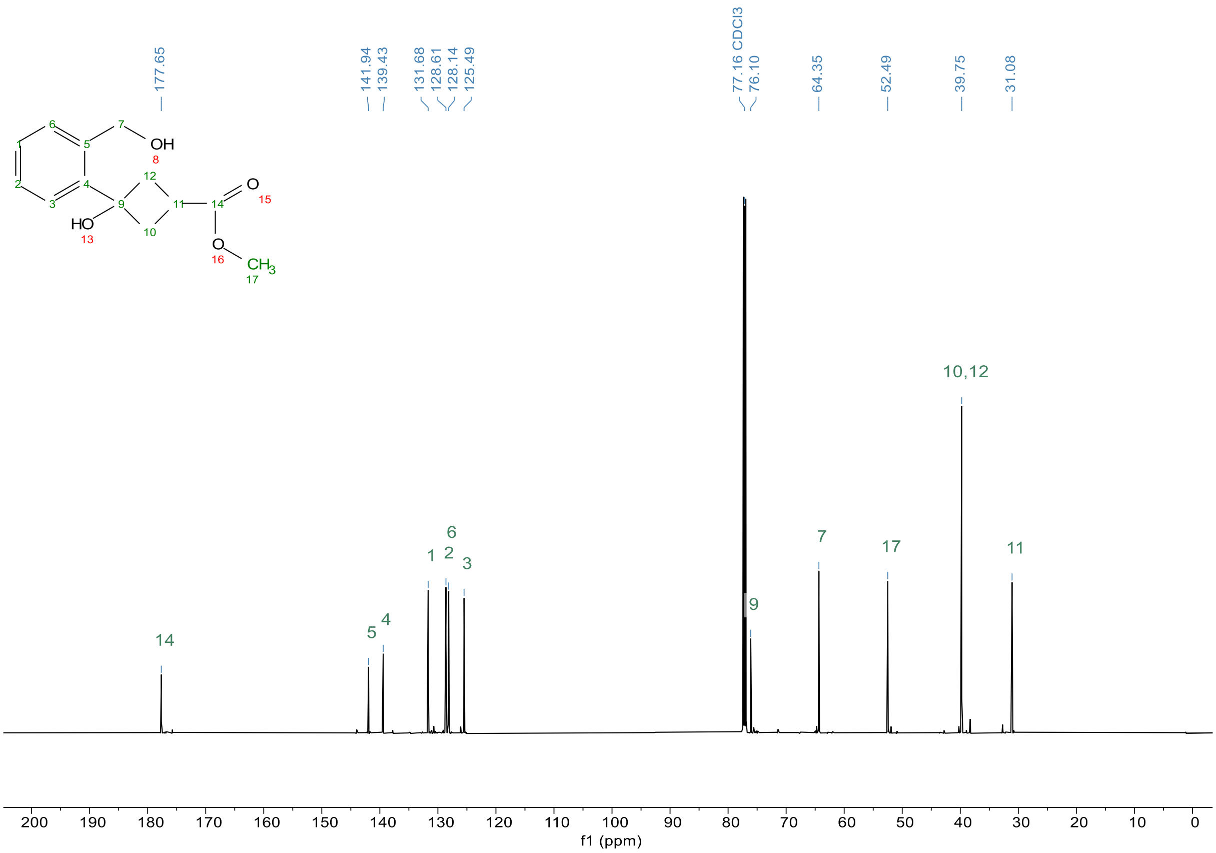

Fig.S6. ^{13}C NMR Spectrum of **S7** (Chloroform-d, 298 K).

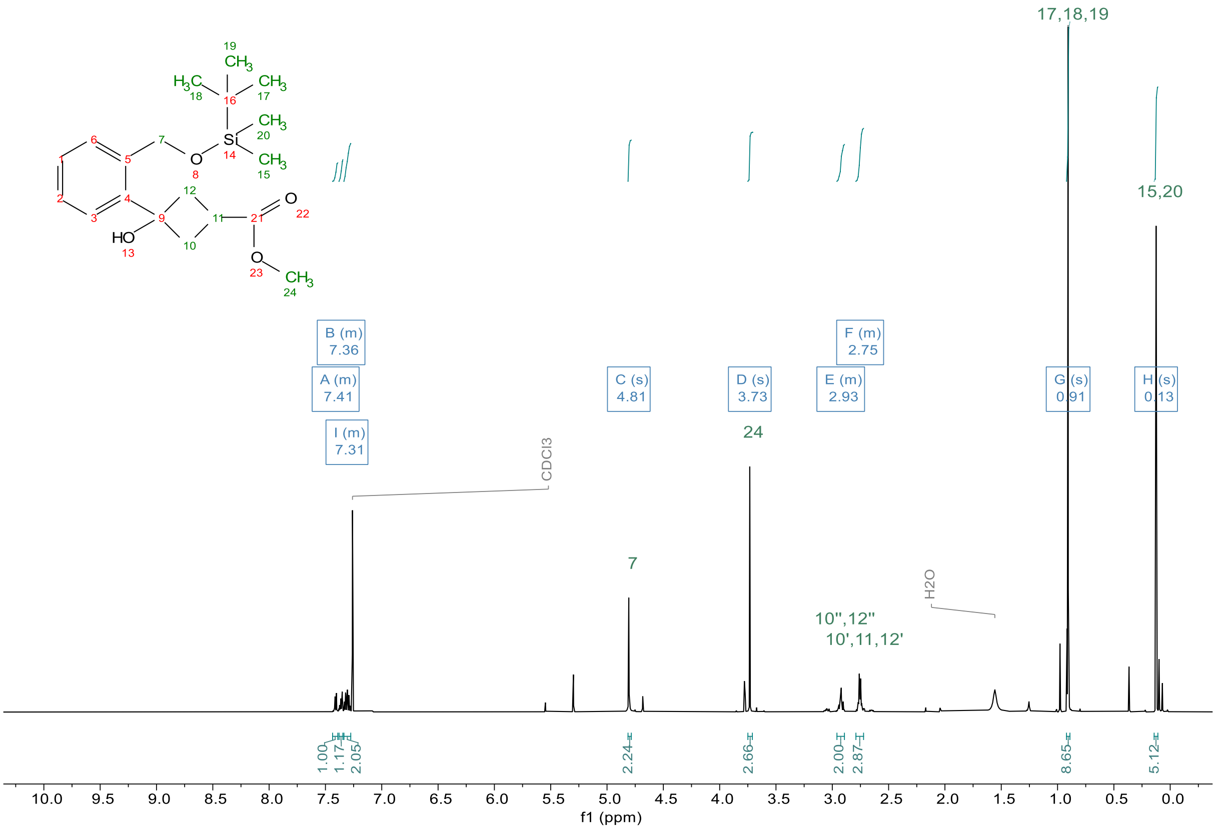

Fig.S7. ^{1}H NMR Spectrum of **S8** (Chloroform-d, 298 K).

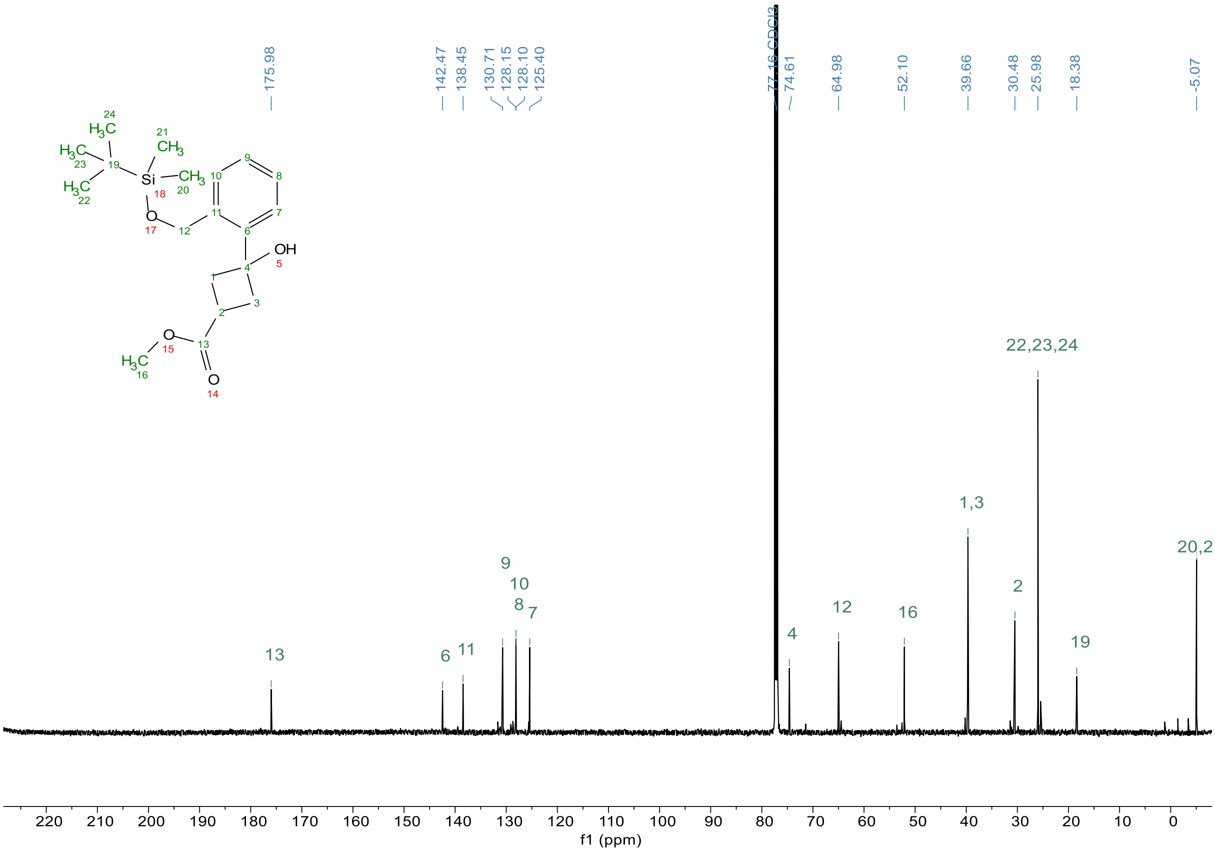

Fig.S8. ^{13}C NMR Spectrum of **S8** (Chloroform-d, 298 K).

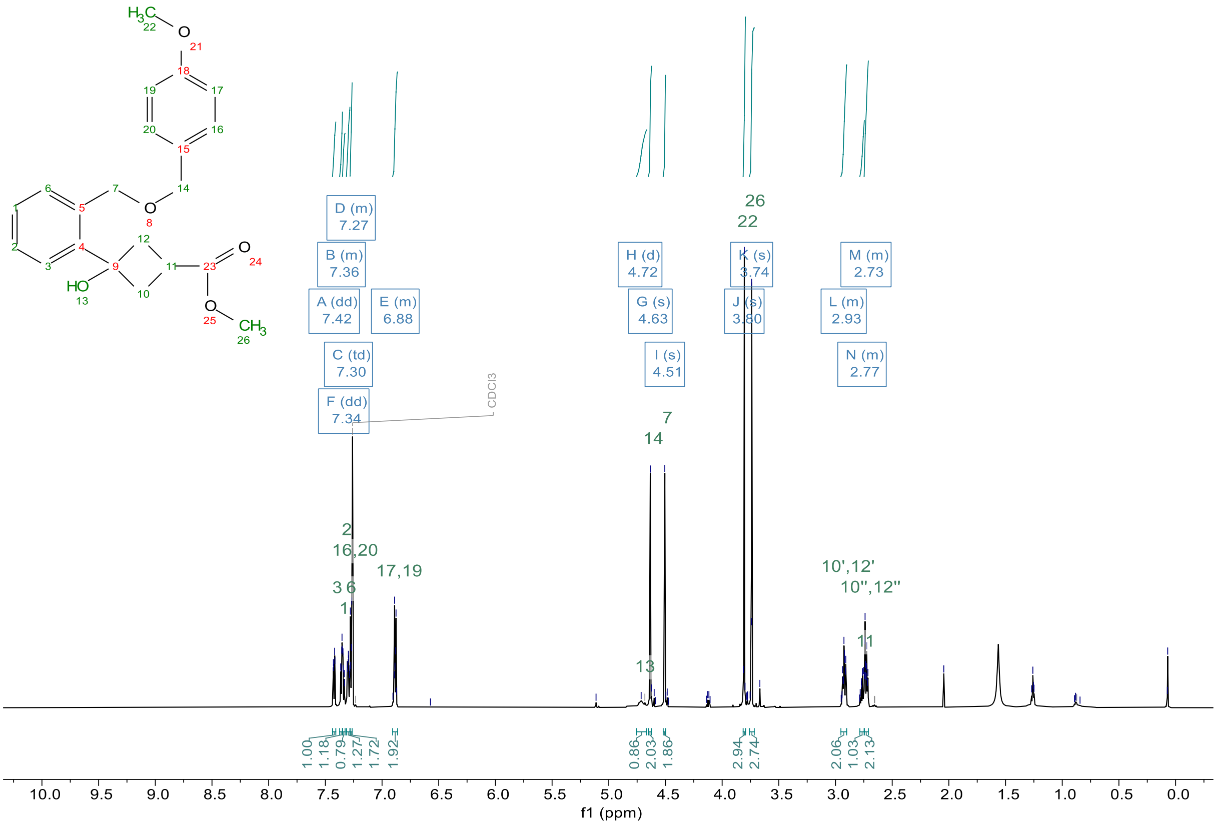

Fig.S9. ^{1}H NMR Spectrum of **S9** (Chloroform-d, 298 K).

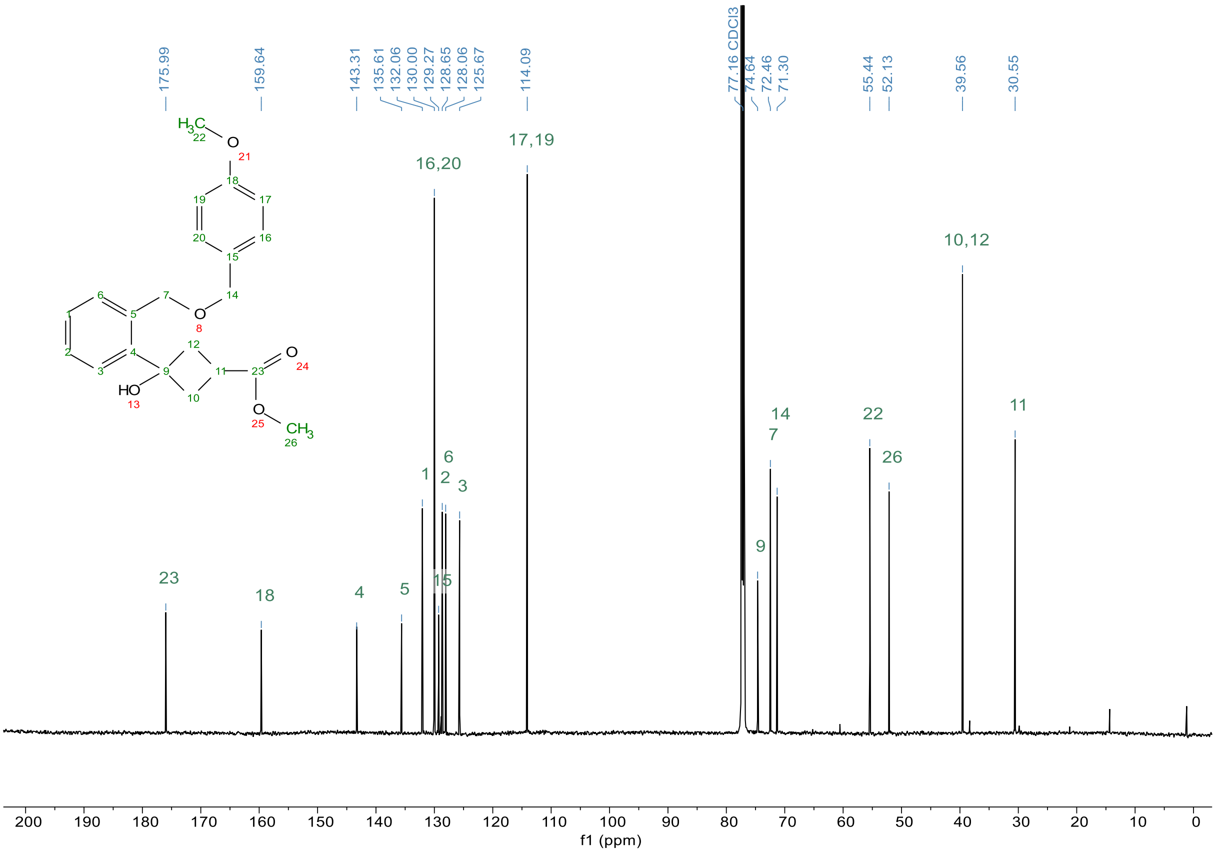

Fig.S10. ^{13}C NMR Spectrum of **S9** (Chloroform-d, 298 K).

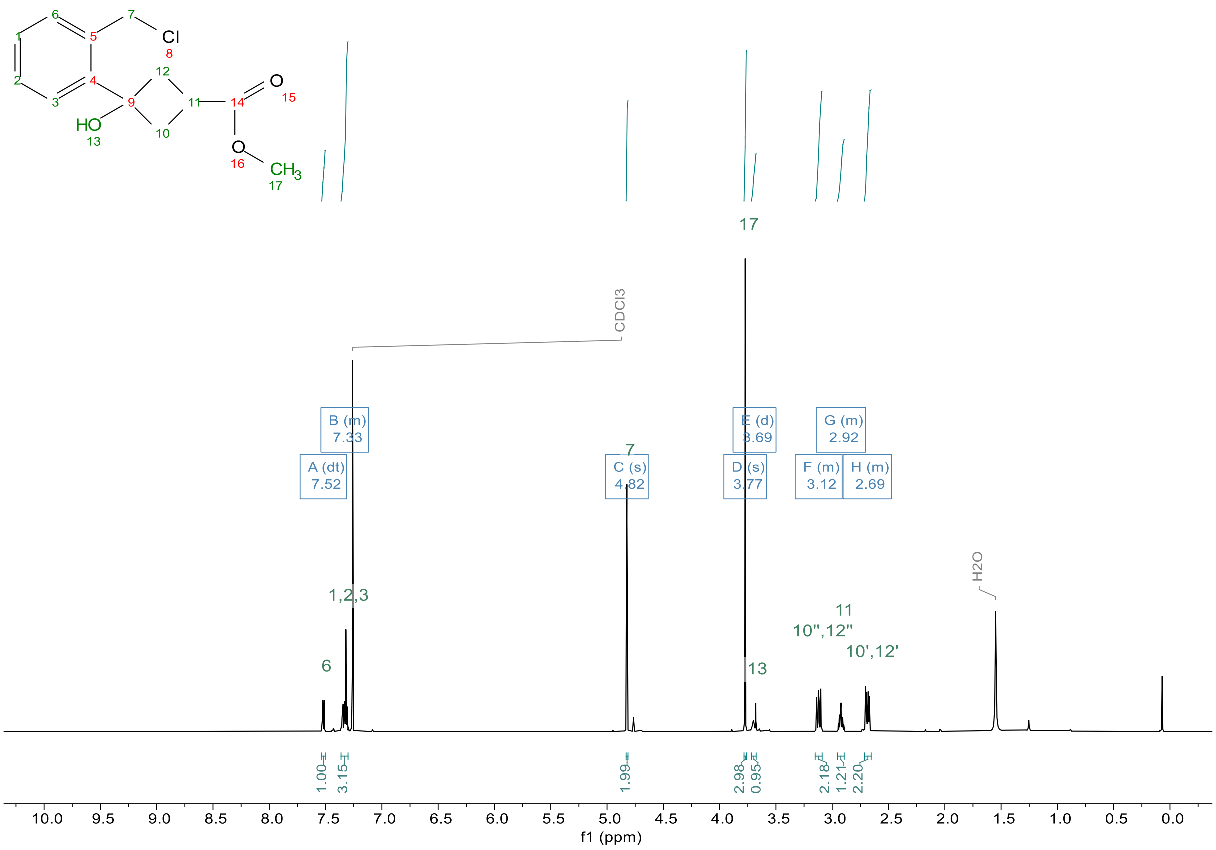

Fig.S11. ^{1}H NMR Spectrum of **S10** (Chloroform-d, 298 K).

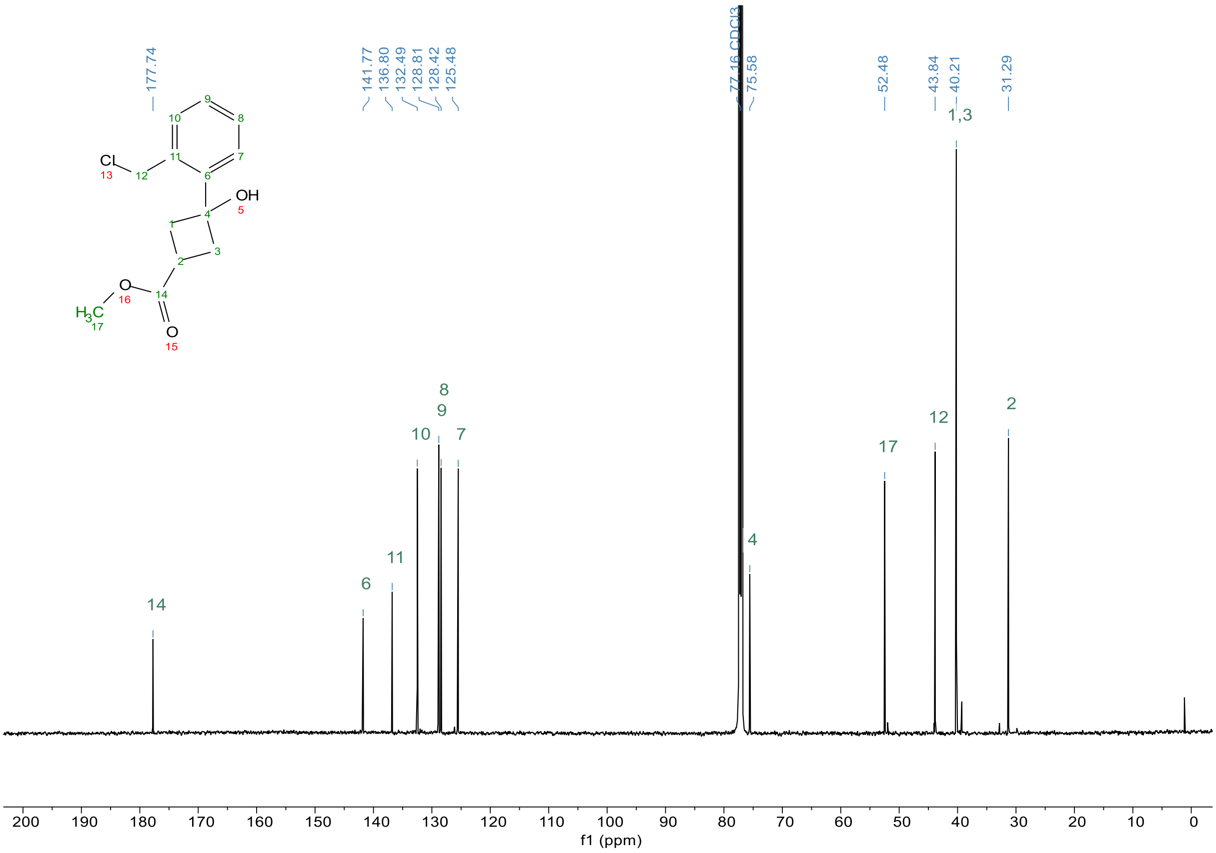

Fig.S12. ^{1}H NMR Spectrum of **S10** (Chloroform-d, 298 K).

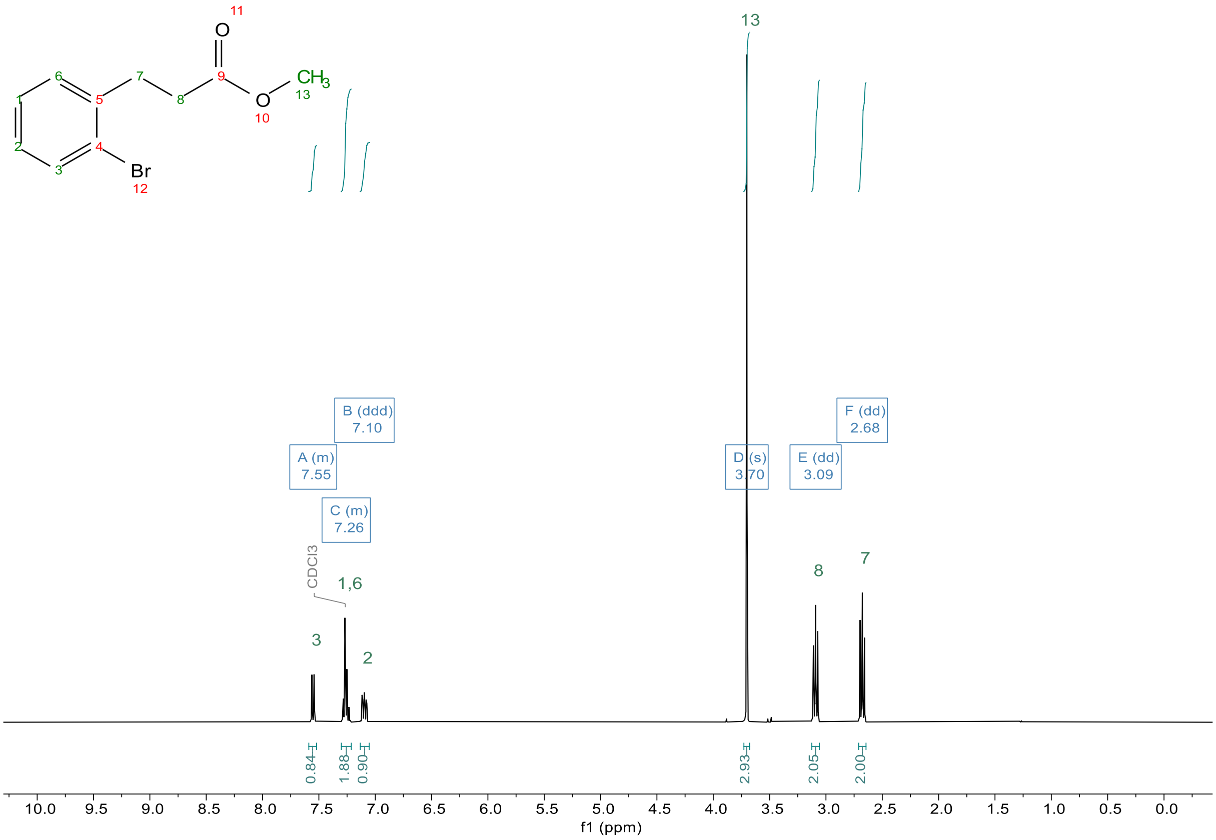

Fig.S13. ^{1}H NMR Spectrum of **S14** (Chloroform-d, 298 K).

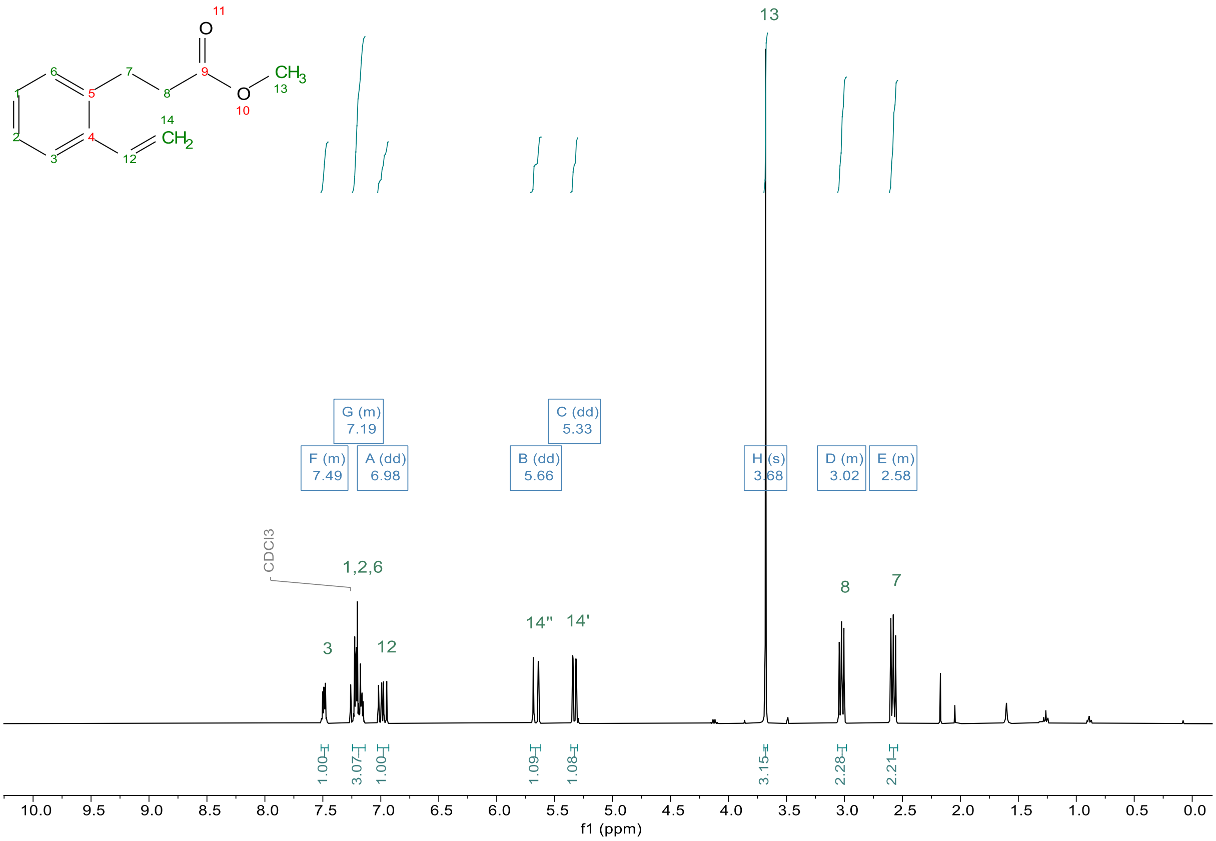

Fig.S14. ^{1}H NMR Spectrum of **S15** (Chloroform-d, 298 K).

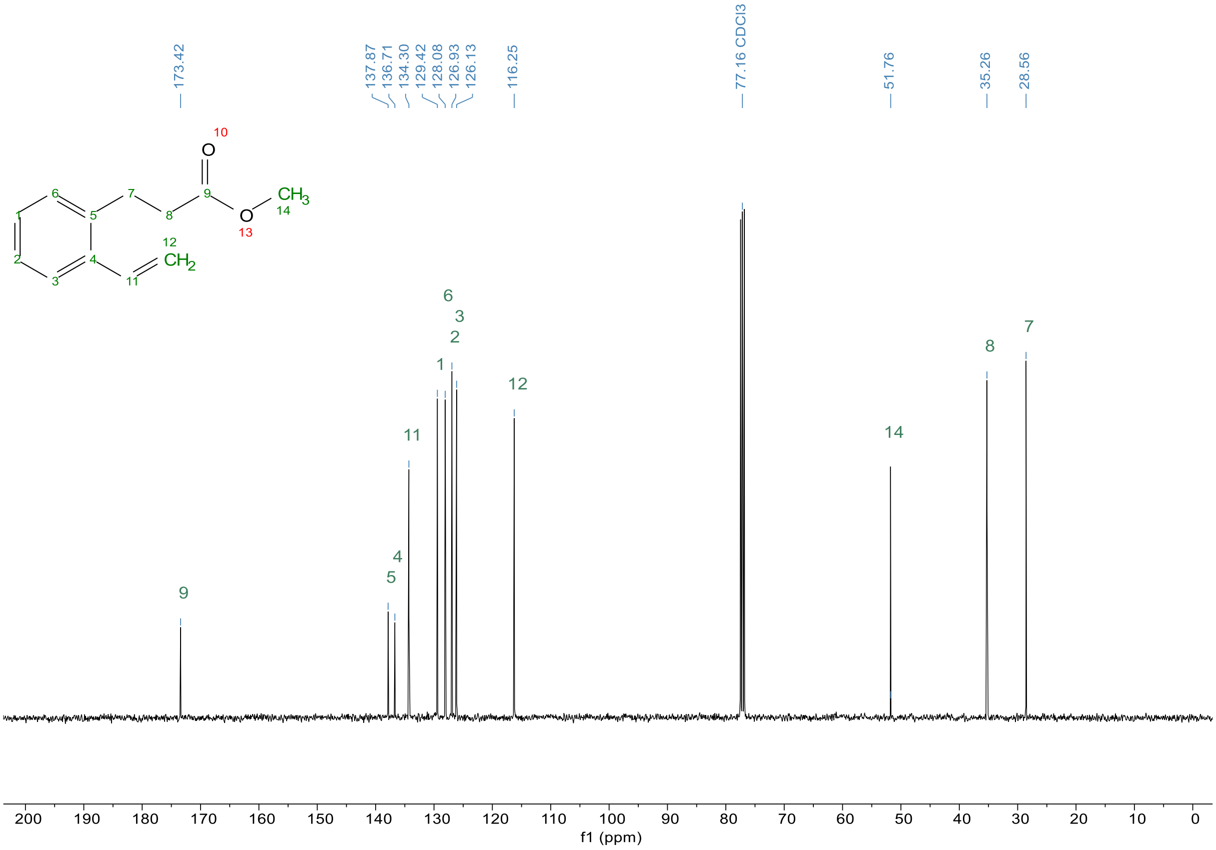

Fig.S15. ^{13}C NMR Spectrum of **S15** (Chloroform-d, 298 K).

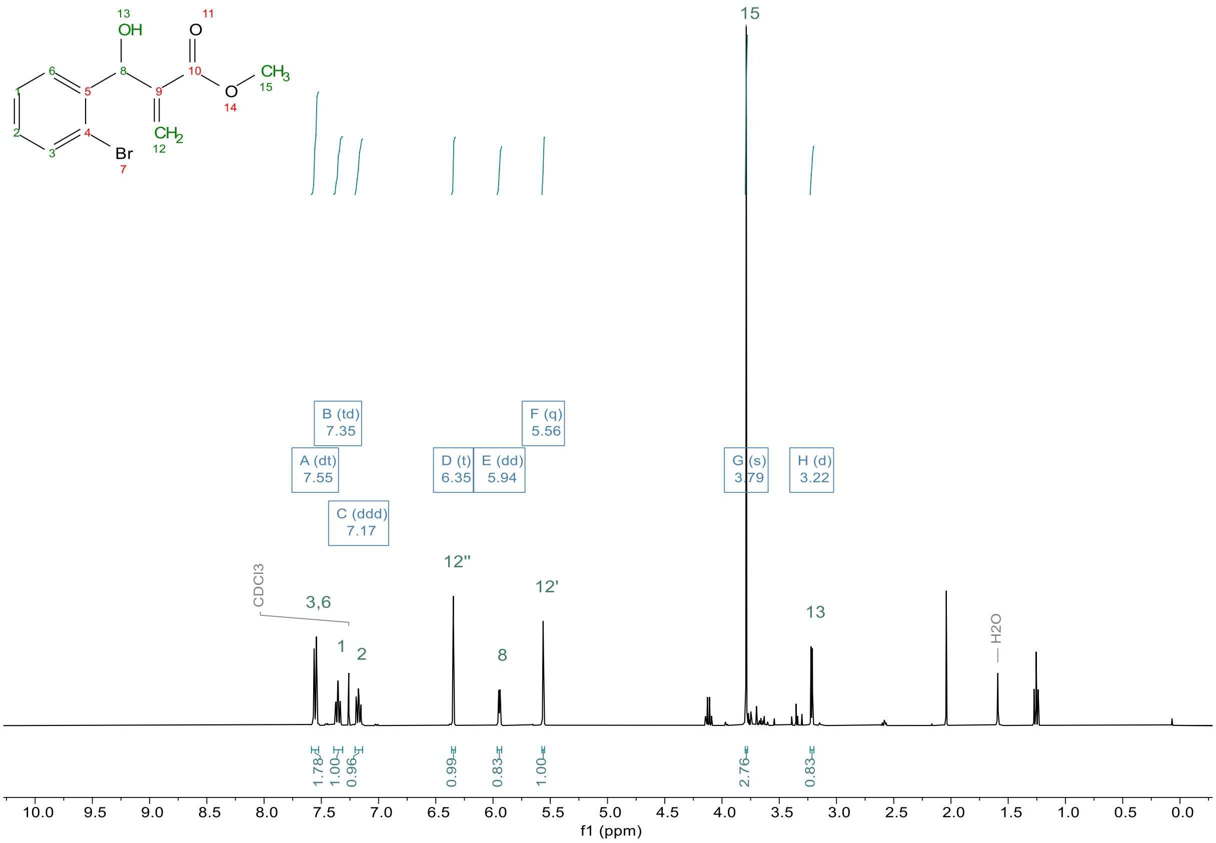

Fig.S16. ^{1}H NMR Spectrum of **S19** (Chloroform-d, 298 K).

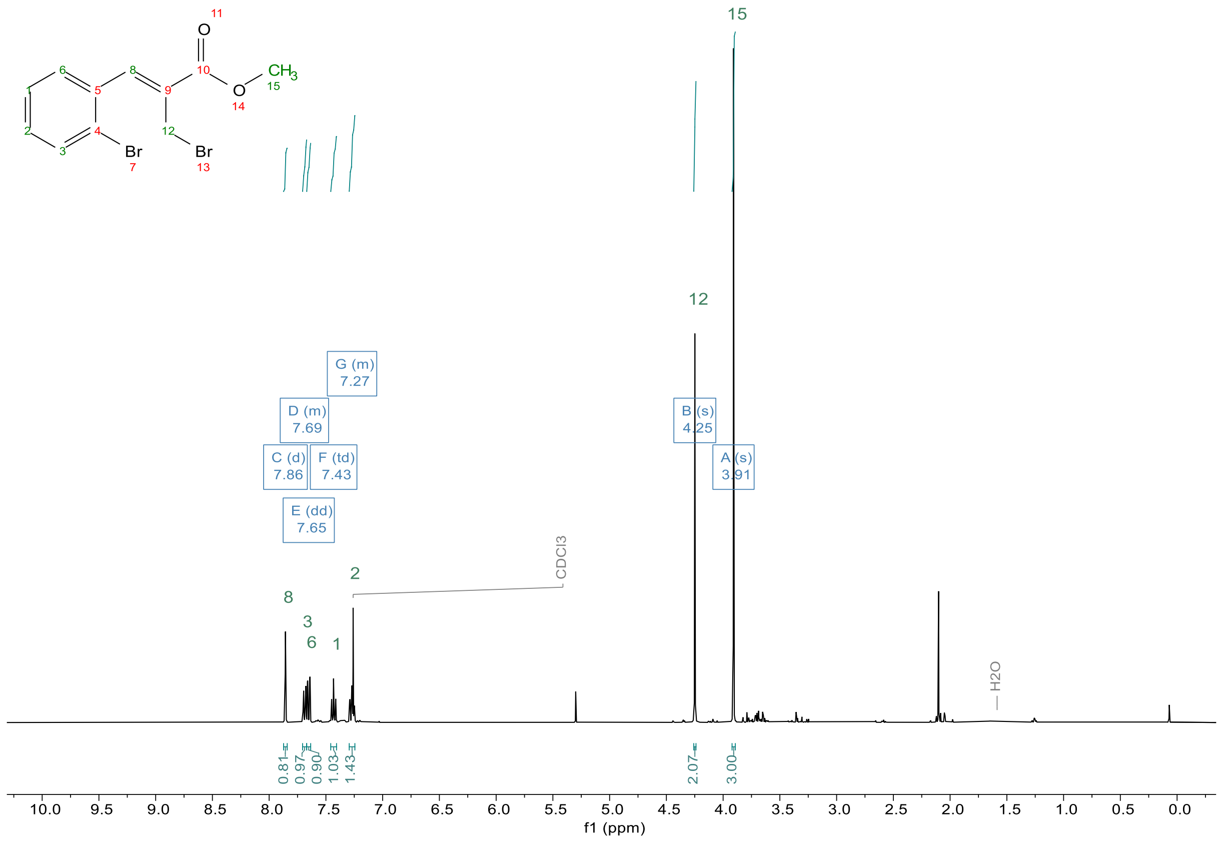

Fig.S17. ^{1}H NMR Spectrum of **S20** (Chloroform-d, 298 K).

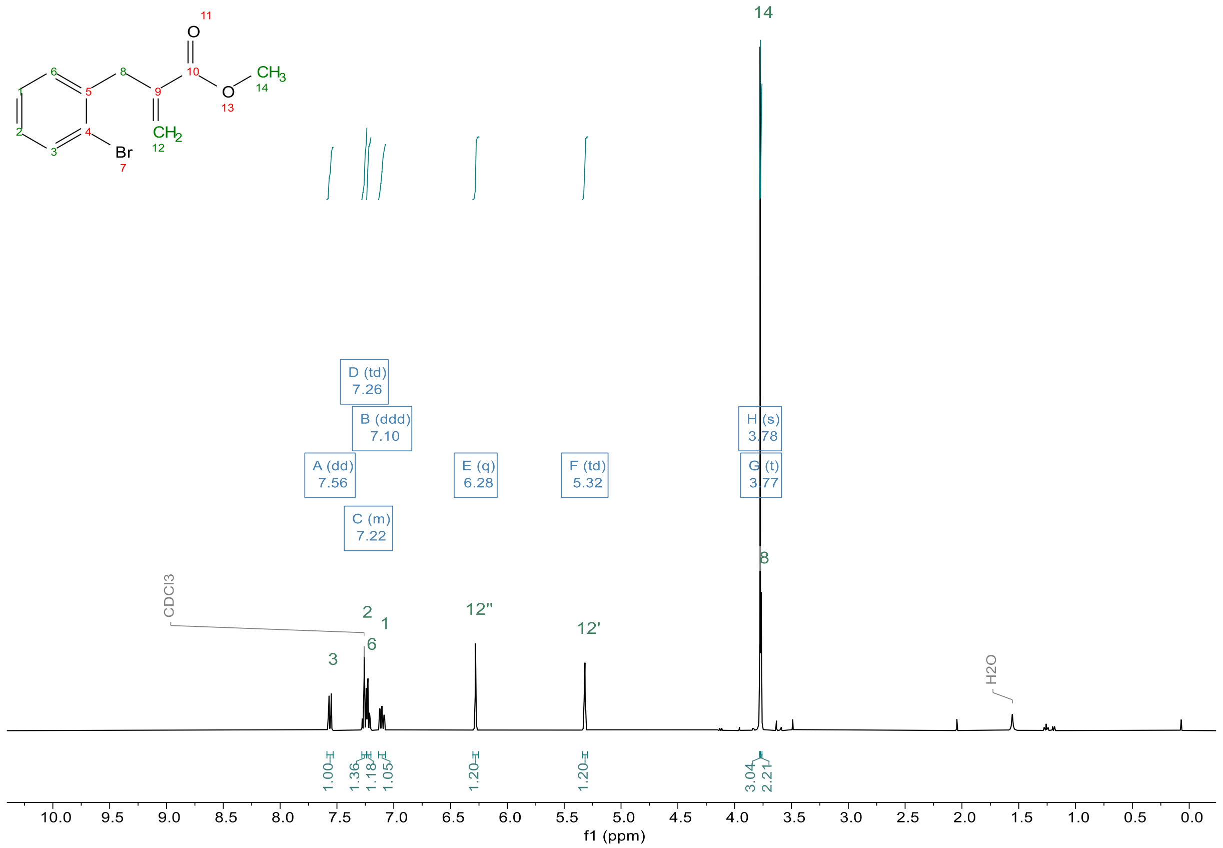

Fig.S18. ^{1}H NMR Spectrum of **S21** (Chloroform-d, 298 K).

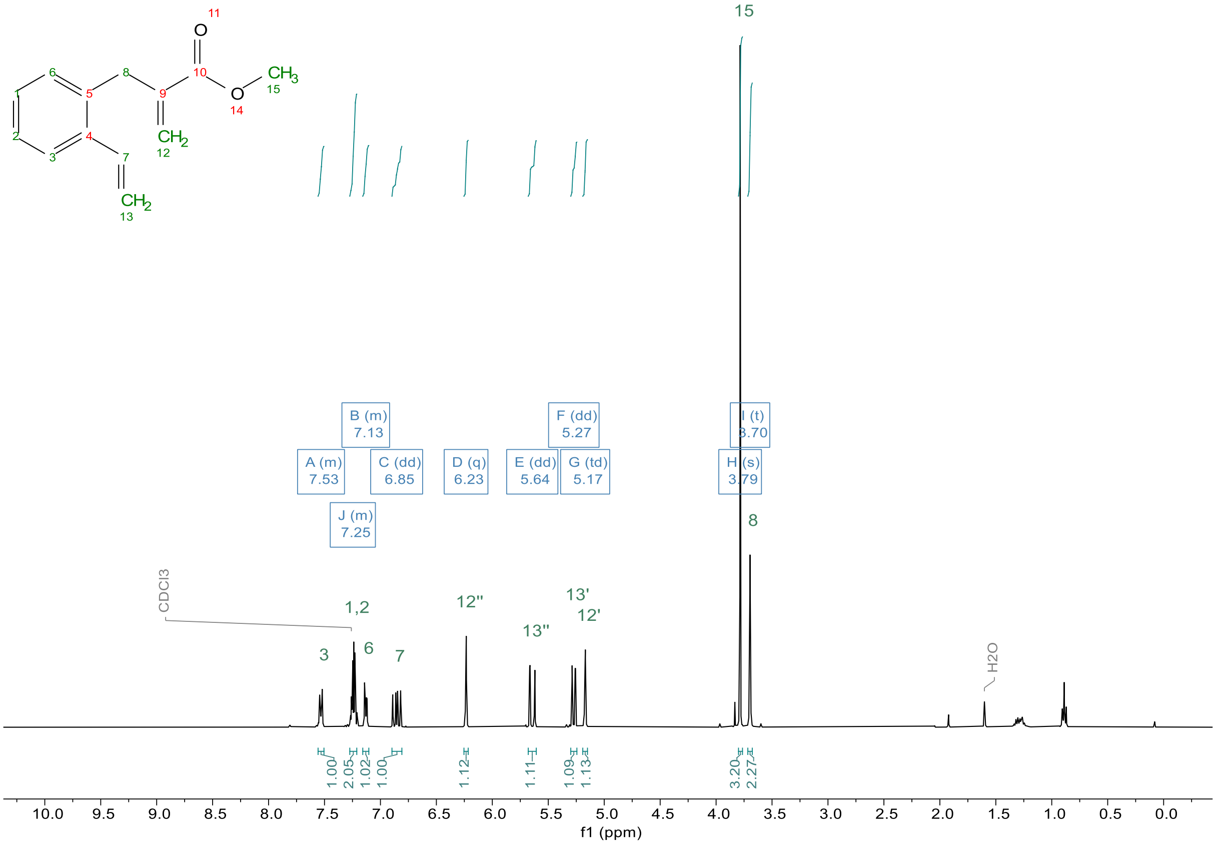

Fig.S19. ^{1}H NMR Spectrum of **S16** (Chloroform-d, 298 K).

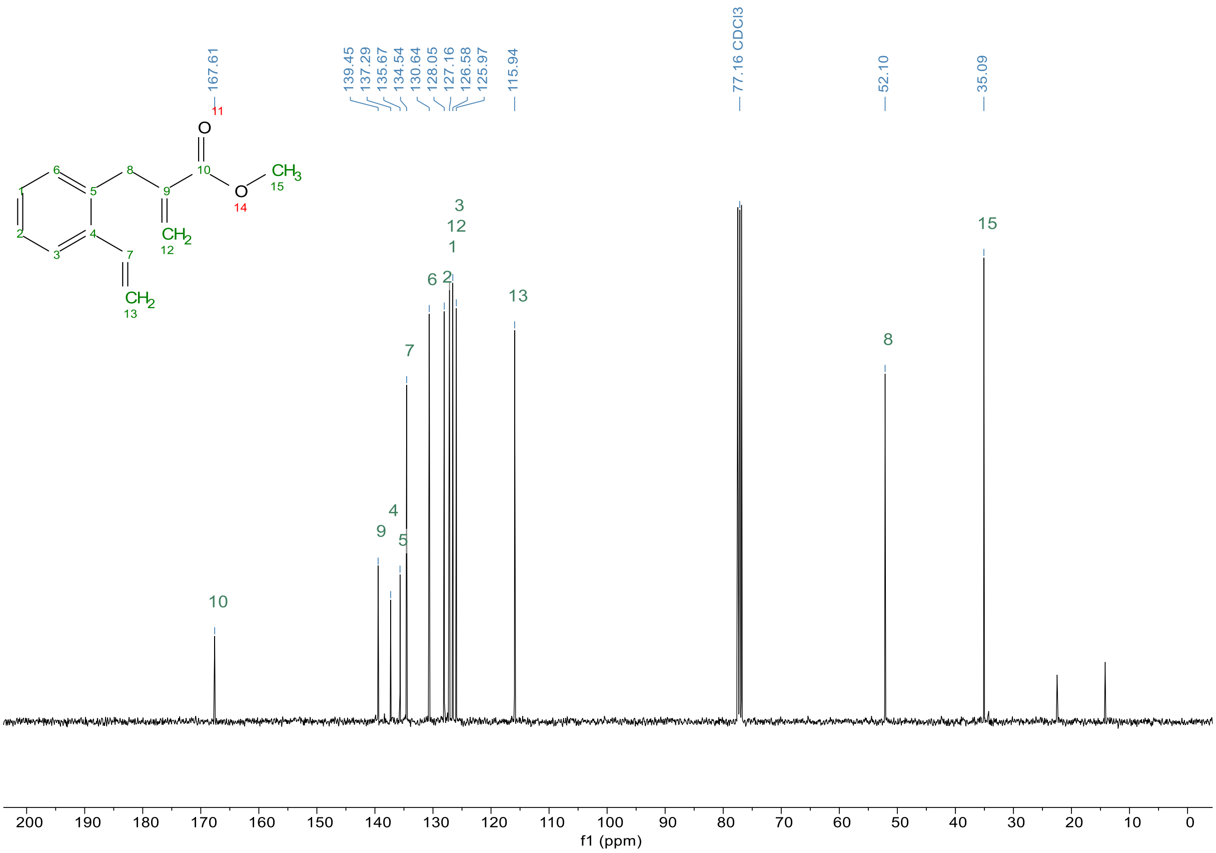

Fig.S20. ^{13}C NMR Spectrum of **S16** (Chloroform-d, 298 K).

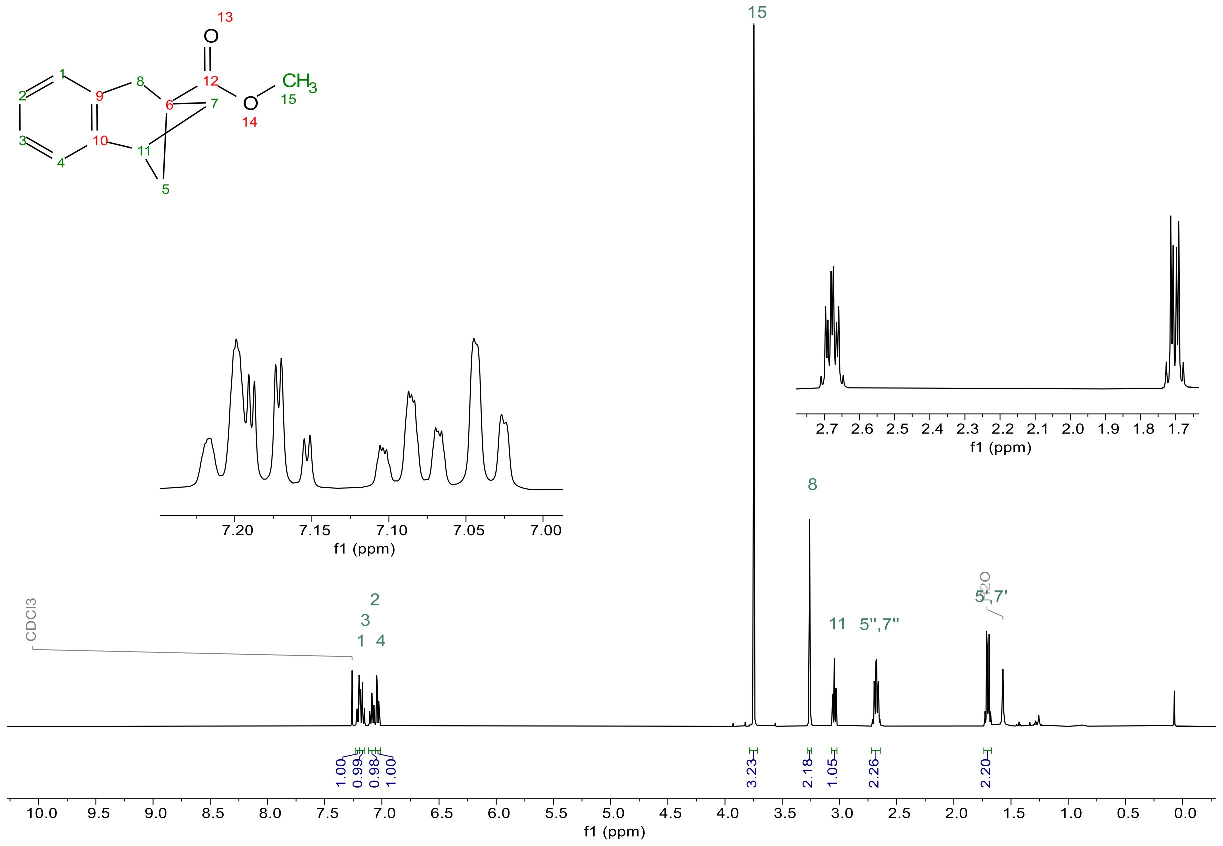

Fig.S21. ^{1}H NMR Spectrum of **18** (Chloroform-d, 298 K).

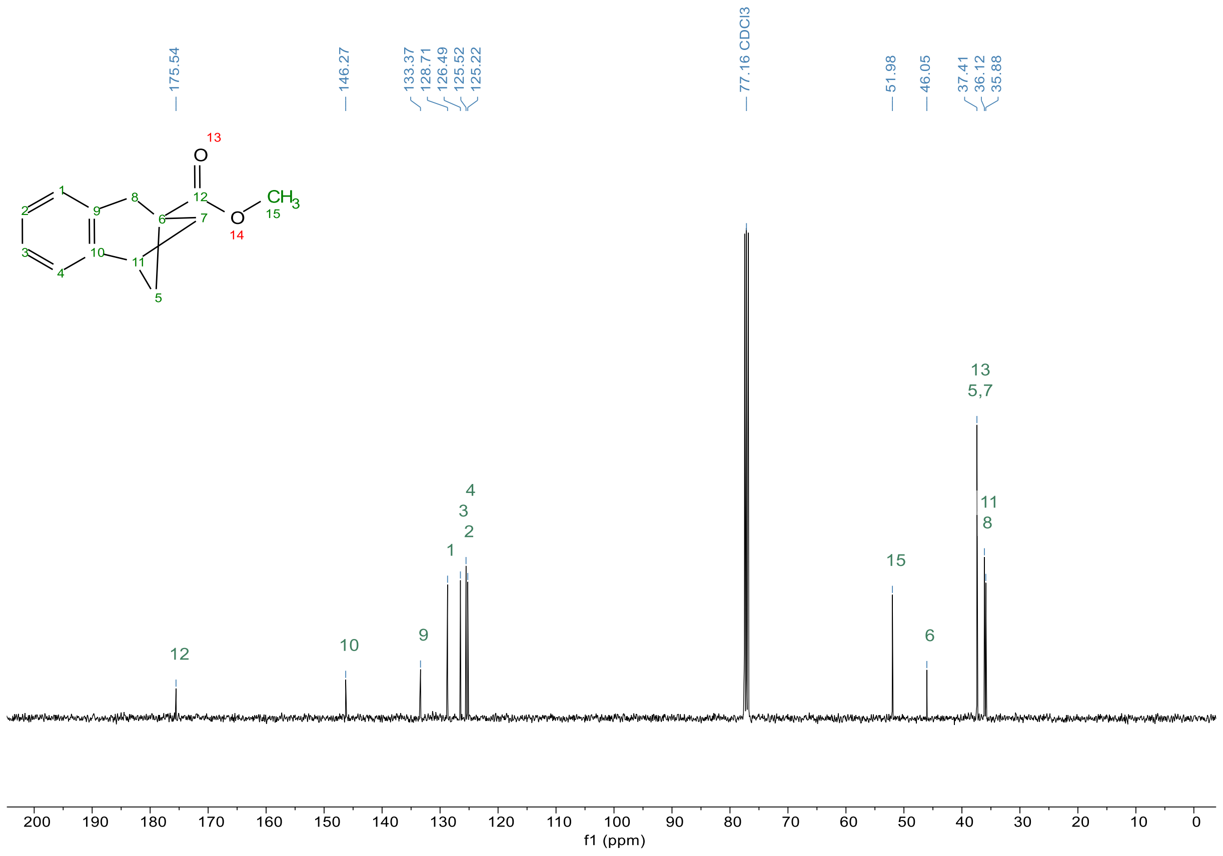

Fig.S22. ^{13}C NMR Spectrum of **18** (Chloroform-d, 298 K).

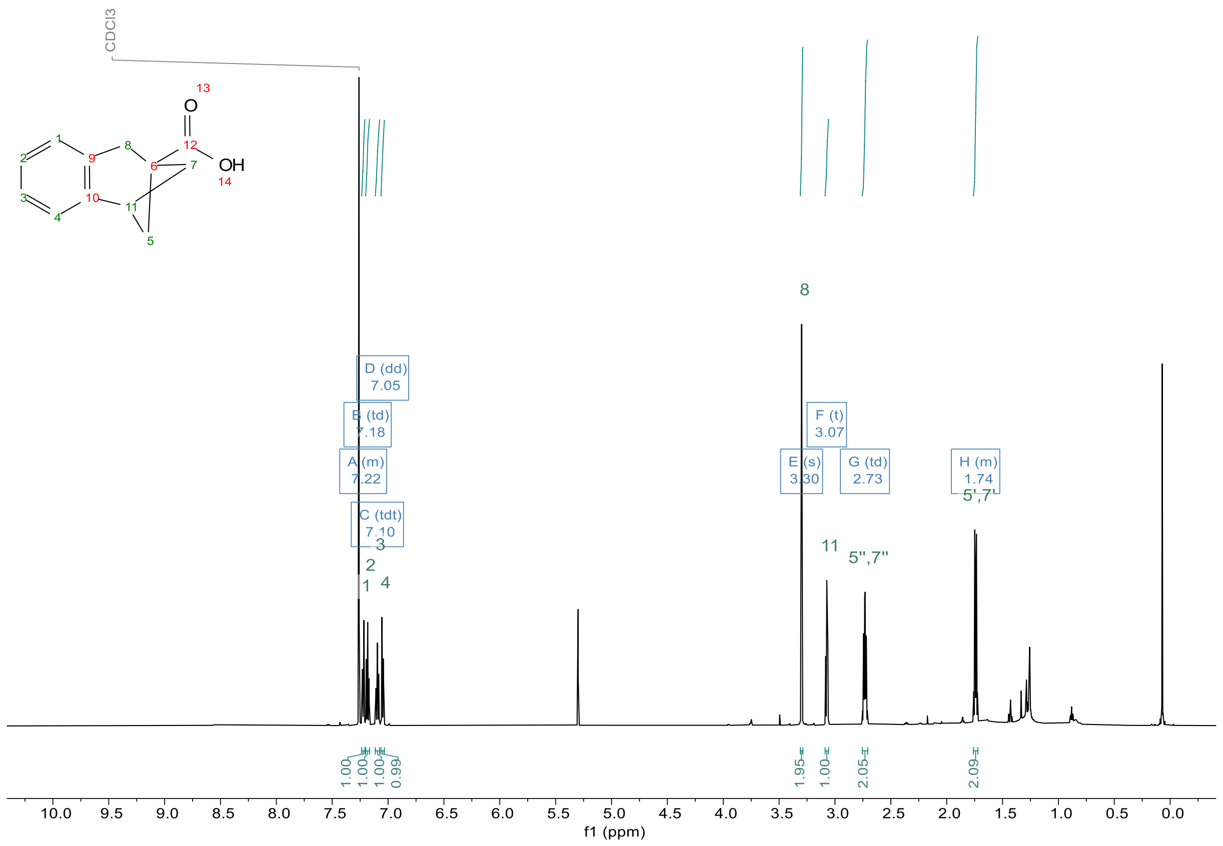

Fig.S23. ^{1}H NMR Spectrum of **36** (Chloroform-d, 298 K).

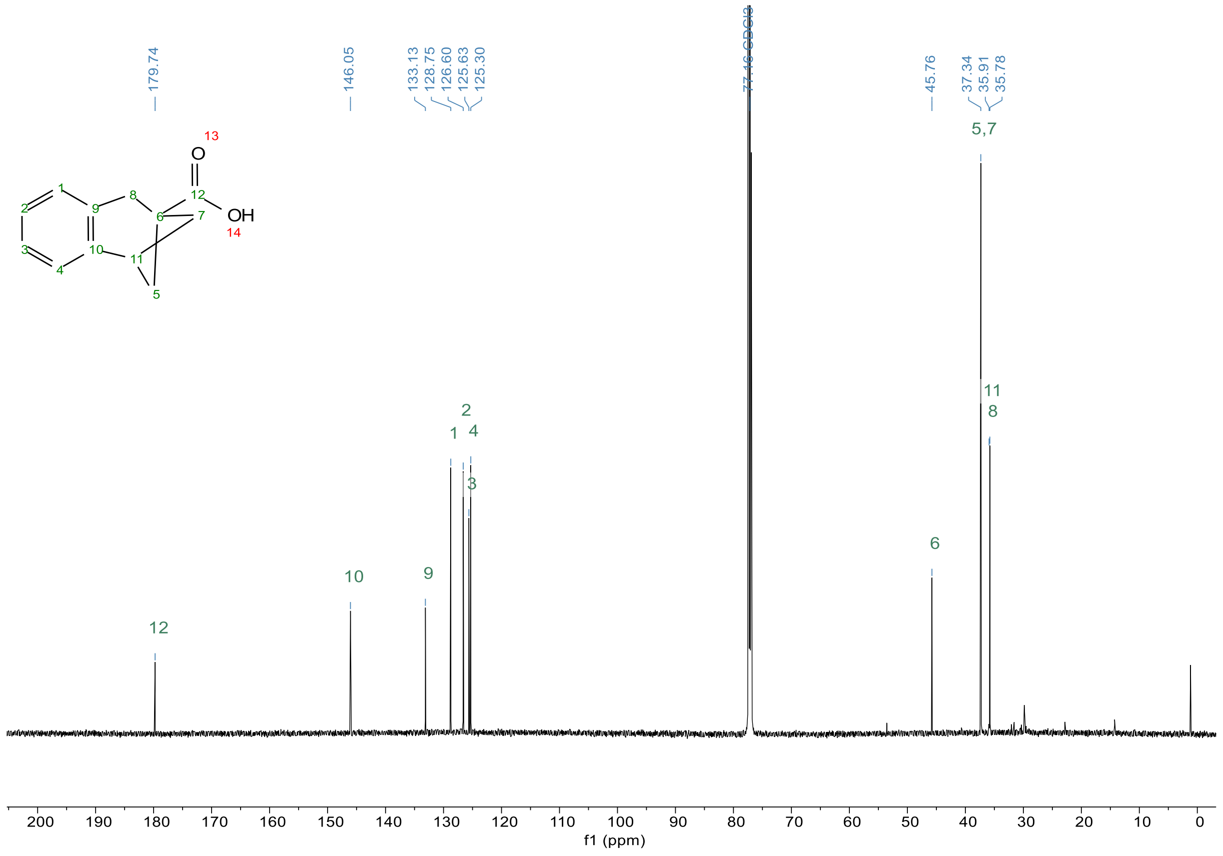

Fig.S24. ^{13}C NMR Spectrum of **36** (Chloroform-d, 298 K).

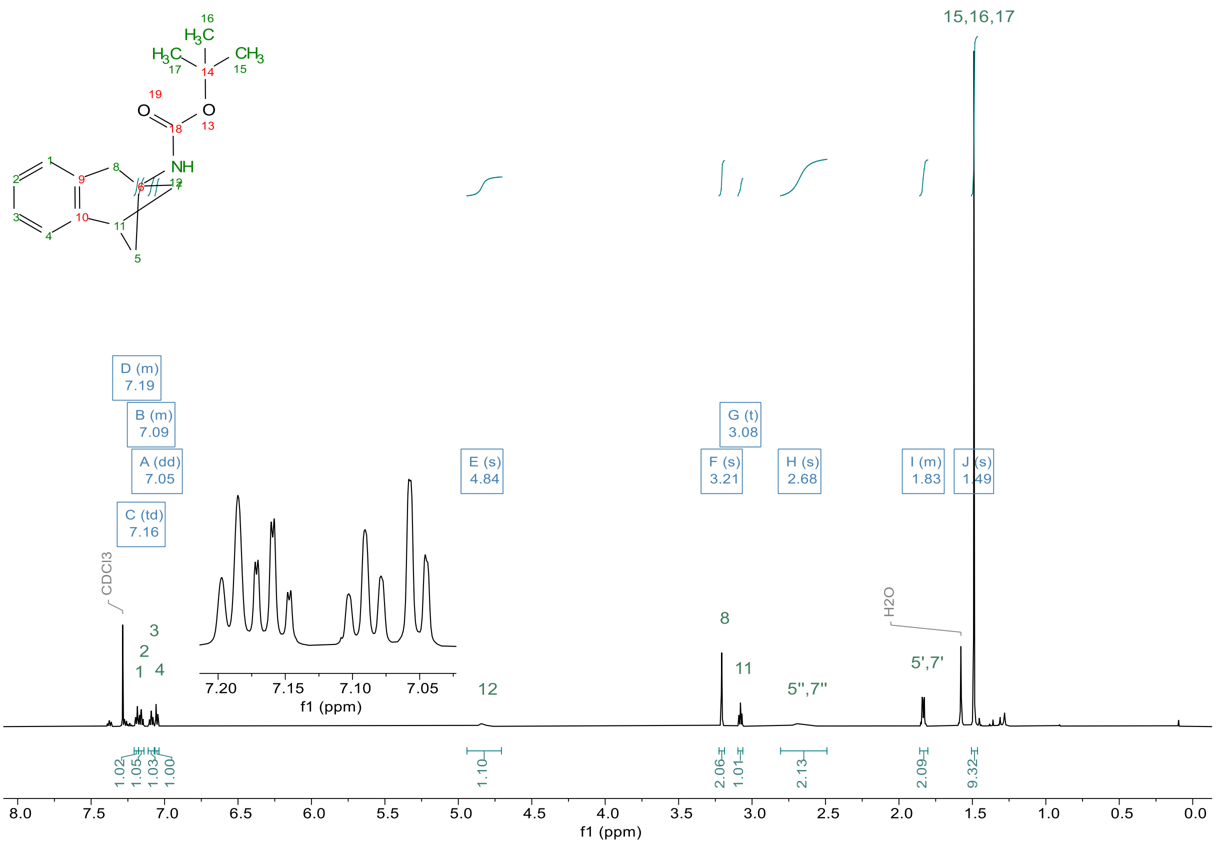

Fig.S25. ^{1}H NMR Spectrum of **S22** (Chloroform-d, 298 K).

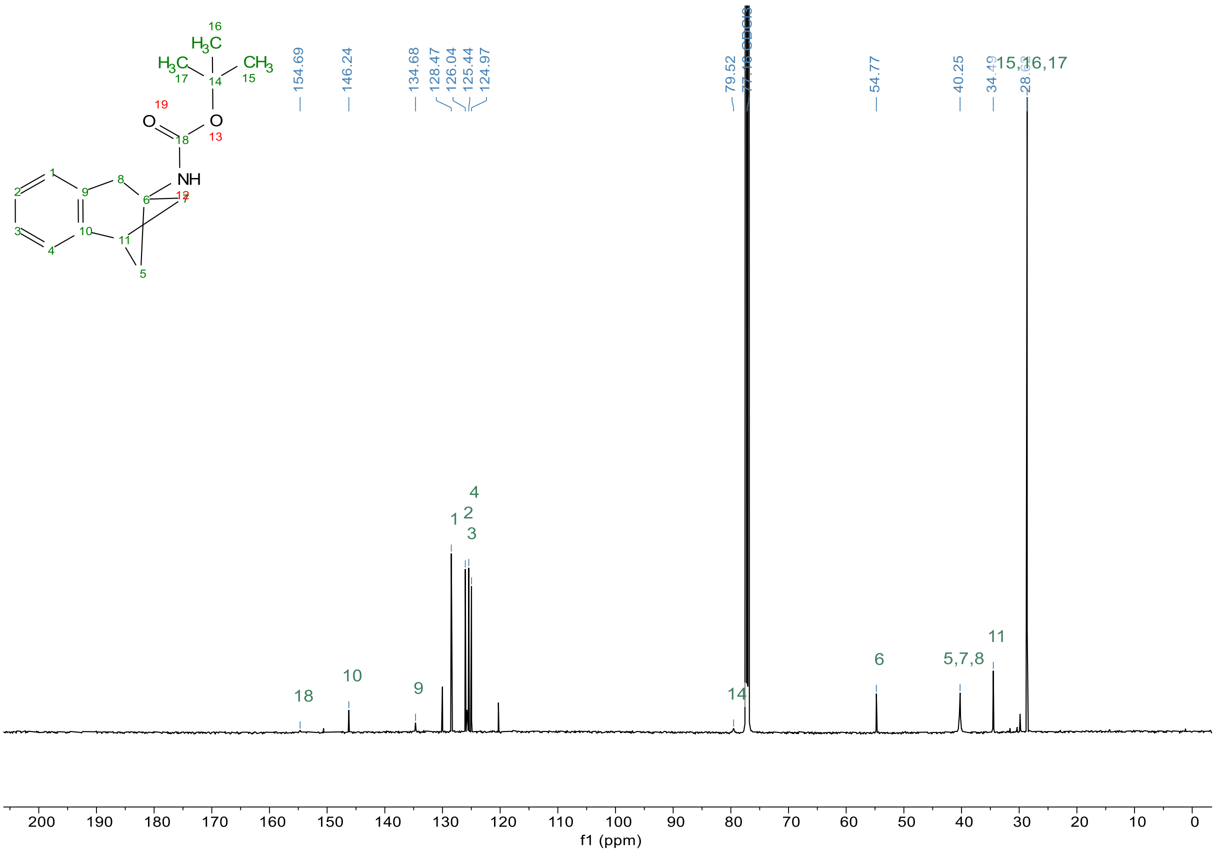

Fig.S26. ^{13}C NMR Spectrum of **S22** (Chloroform-d, 298 K).

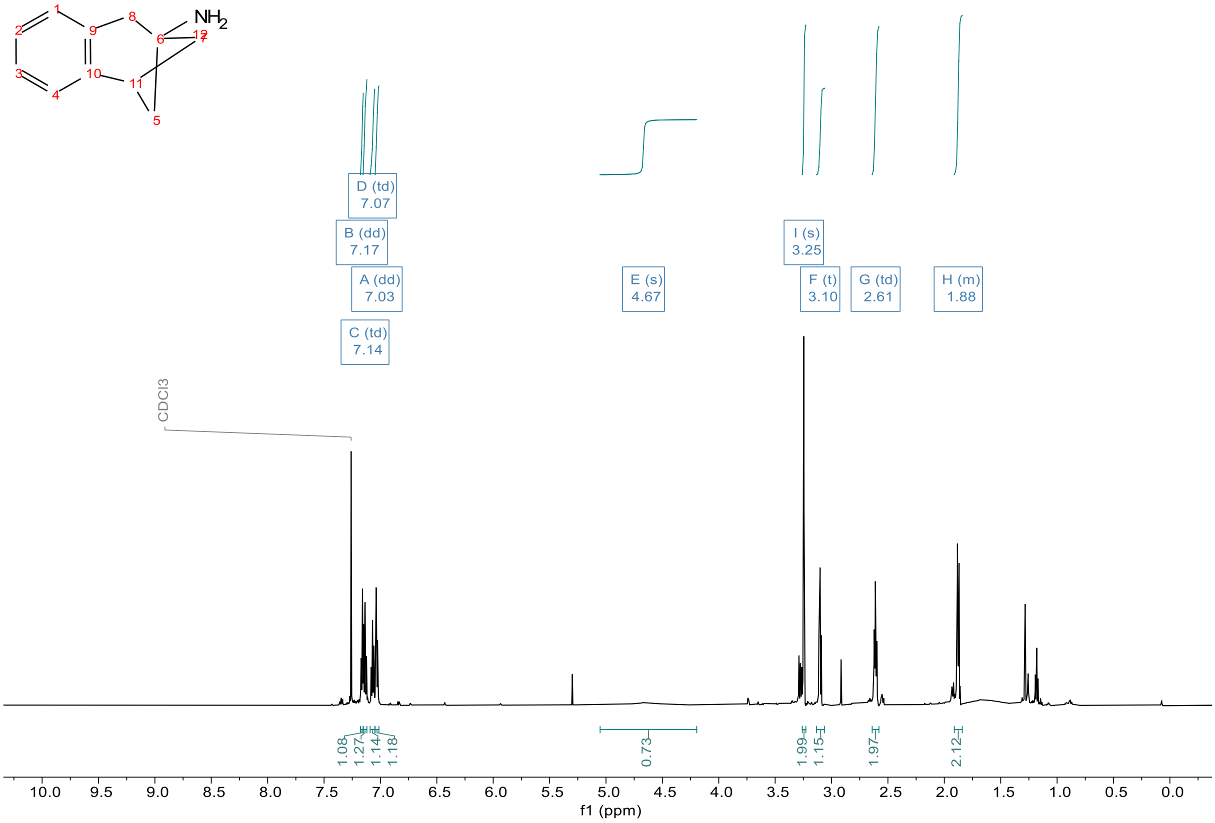

Fig.S27. ^{1}H NMR Spectrum of **37** (Chloroform-d, 298 K).

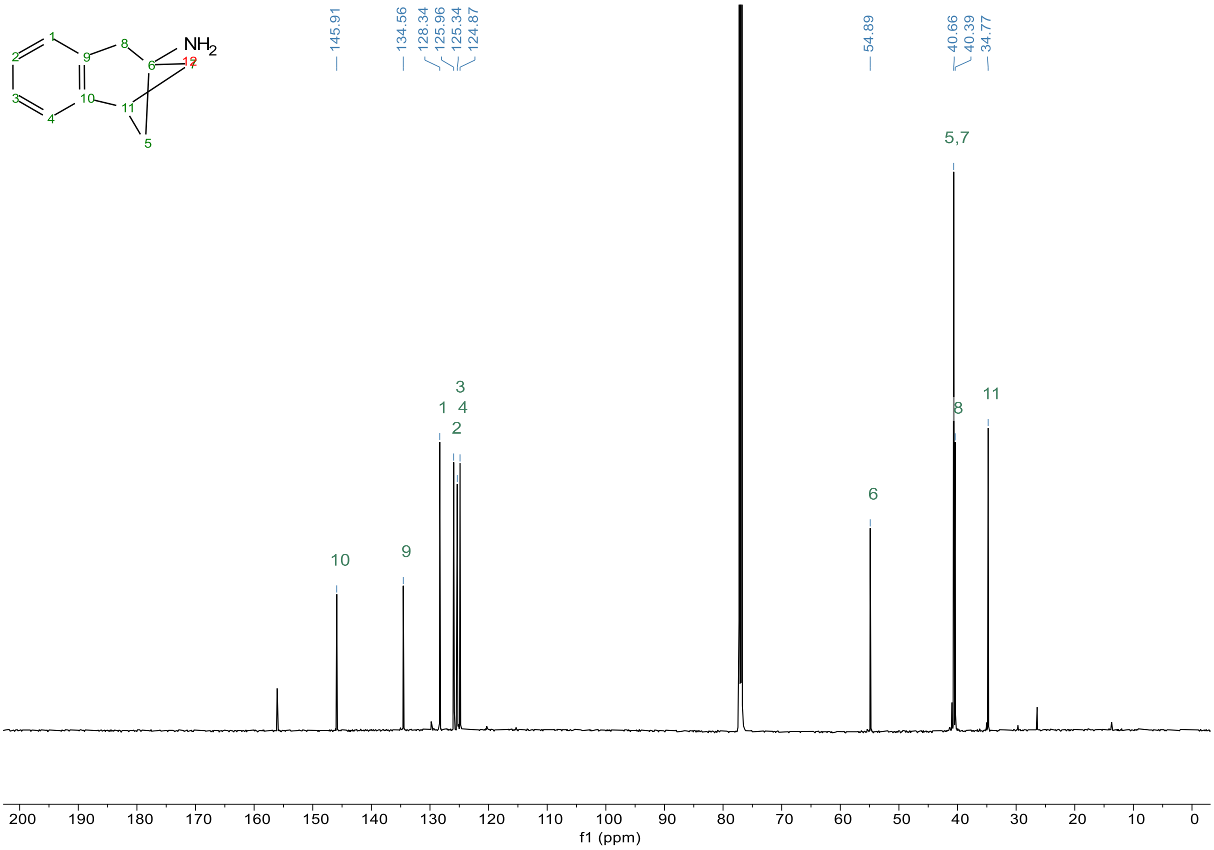

Fig.S28. ^{13}C NMR Spectrum of **37** (Chloroform-d, 298 K).

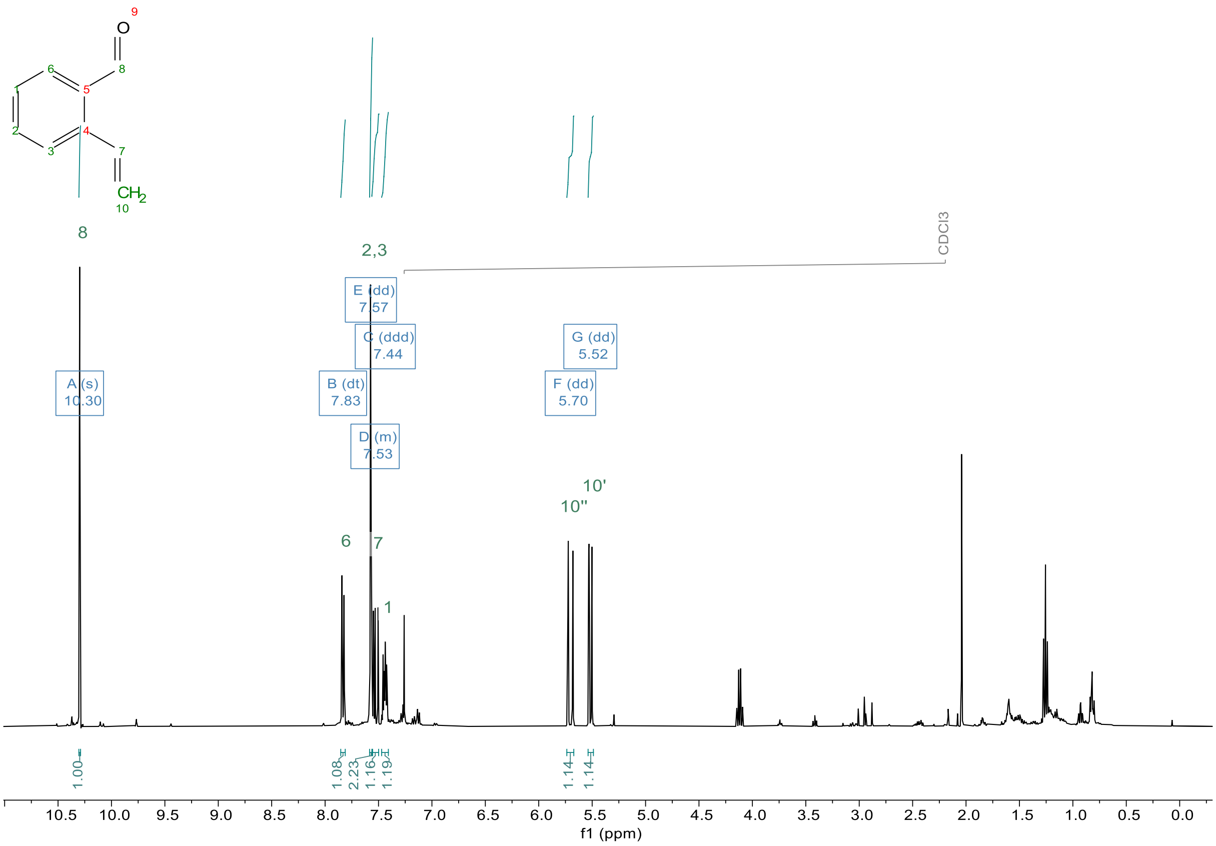

Fig.S29. ^{1}H NMR Spectrum of **S24** (Chloroform-d, 298 K).

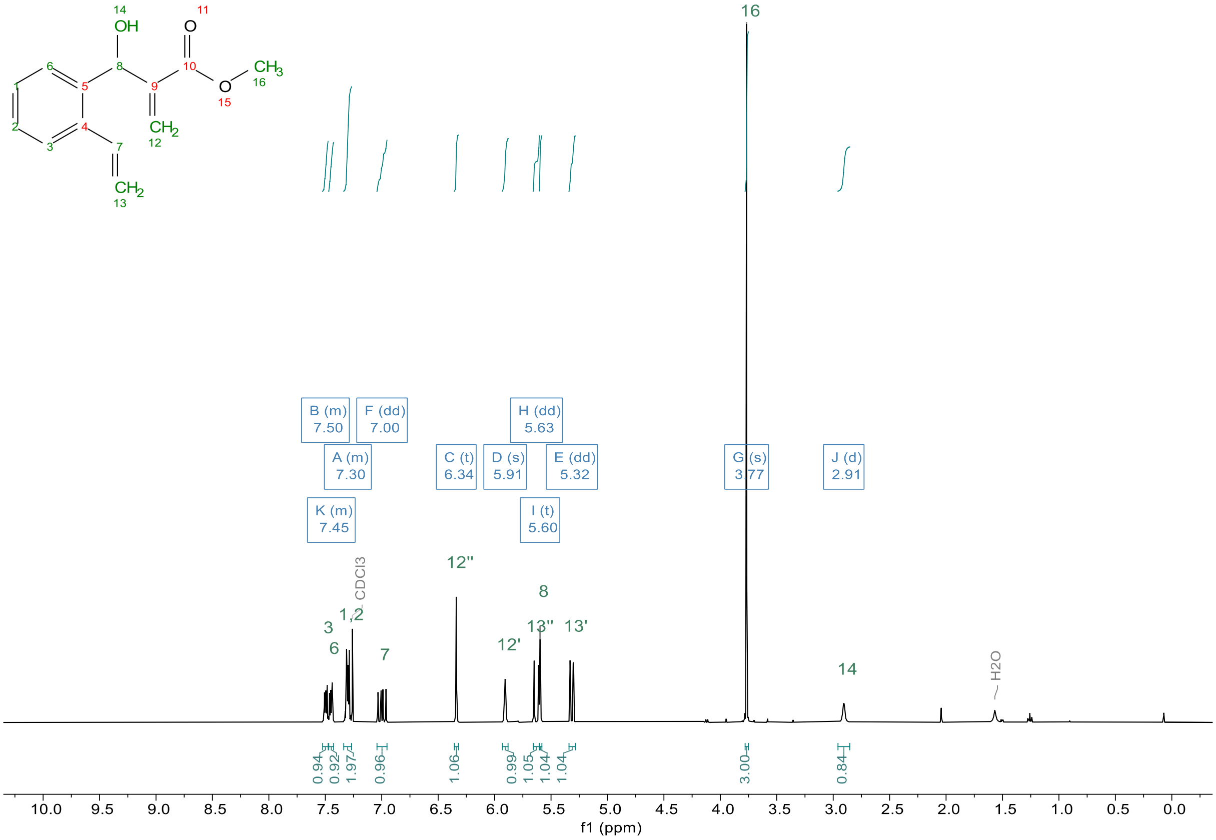

Fig.S30. ^{1}H NMR Spectrum of **S25** (Chloroform-d, 298 K).

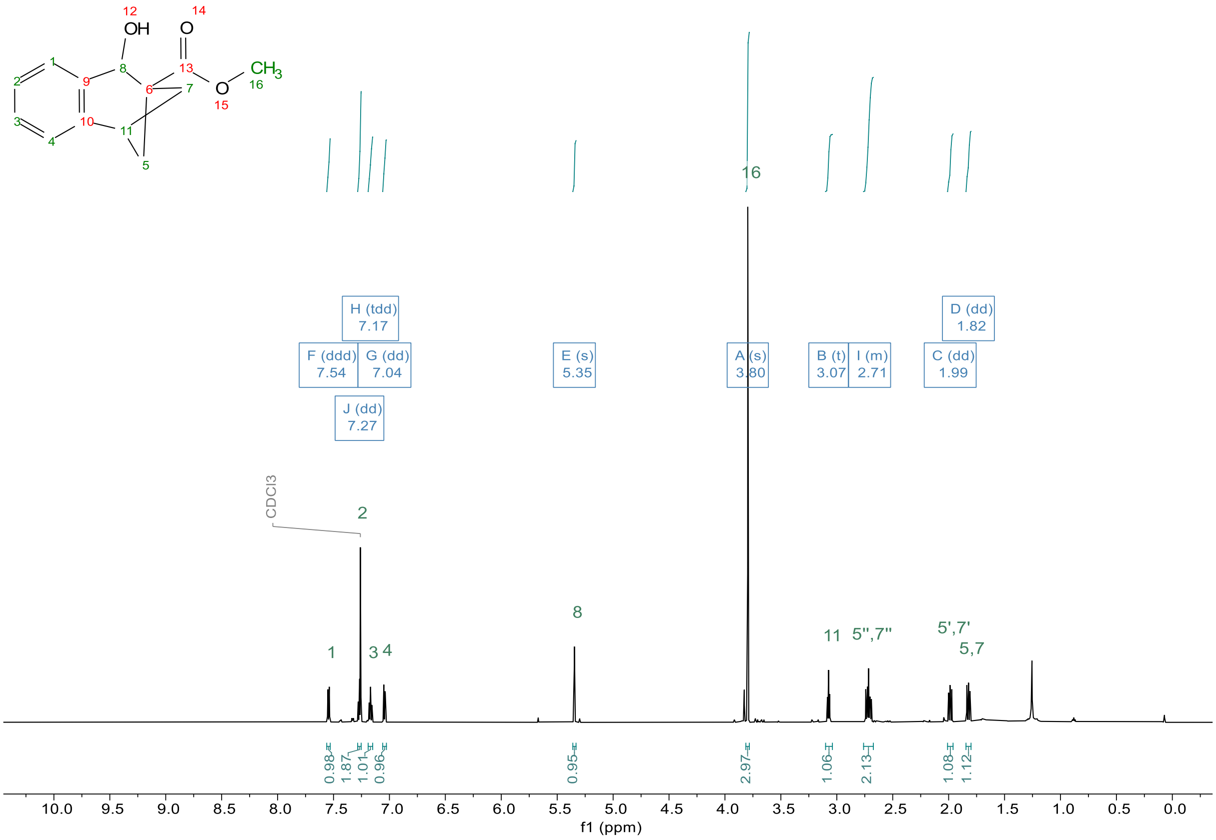

Fig.S31. ^{1}H NMR Spectrum of **19** (Chloroform-d, 298 K).

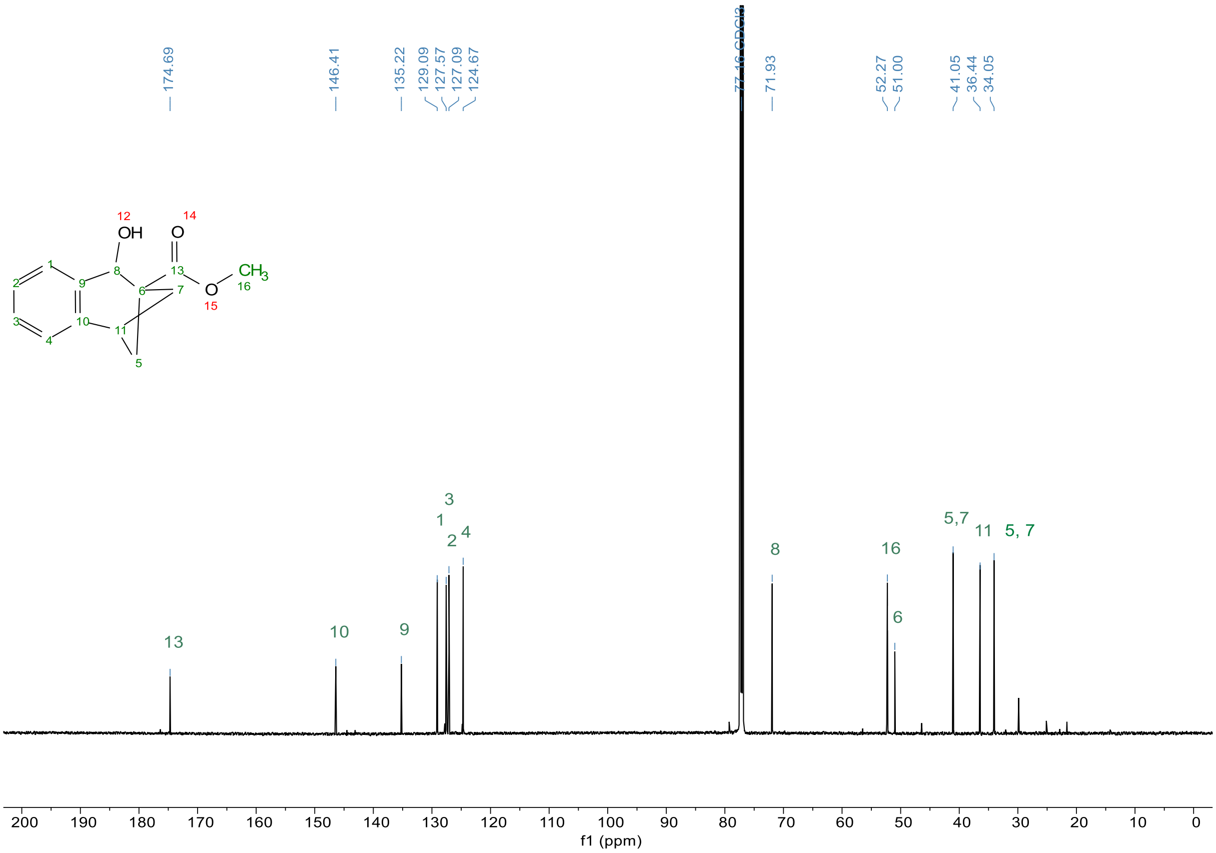

Fig.S32. ^{13}C NMR Spectrum of **19** (Chloroform-d, 298 K).

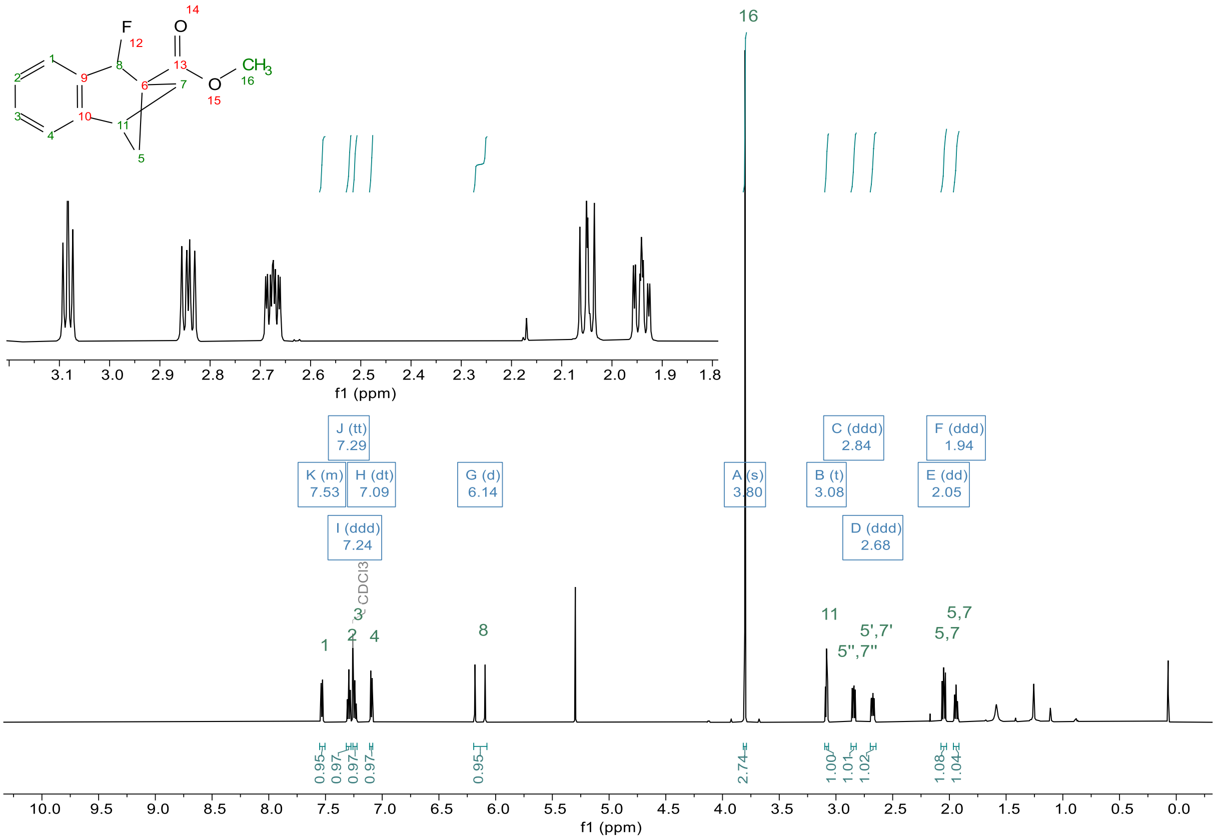

Fig.S33. ^{1}H NMR Spectrum of **44** (Chloroform-d, 298 K).

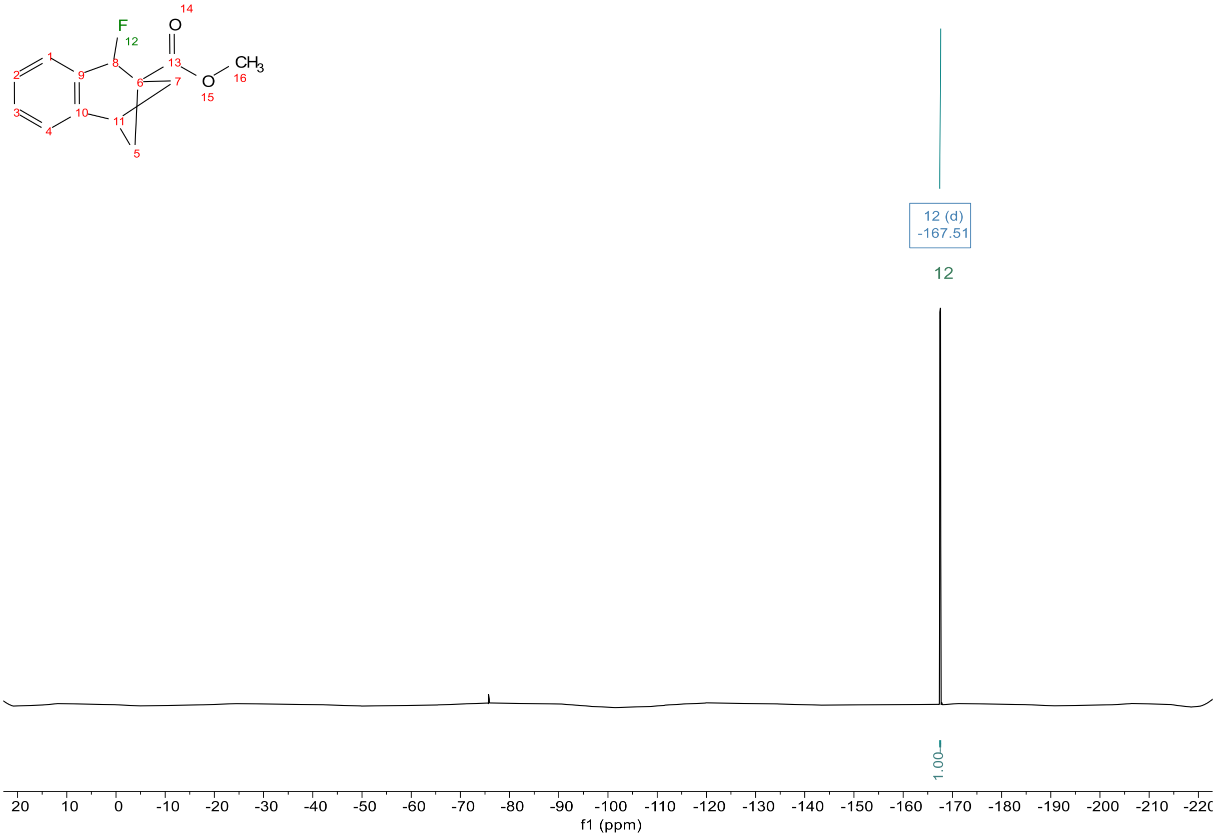

Fig.S34. ^{19}F NMR Spectrum of **44** (Chloroform-d, 298 K).

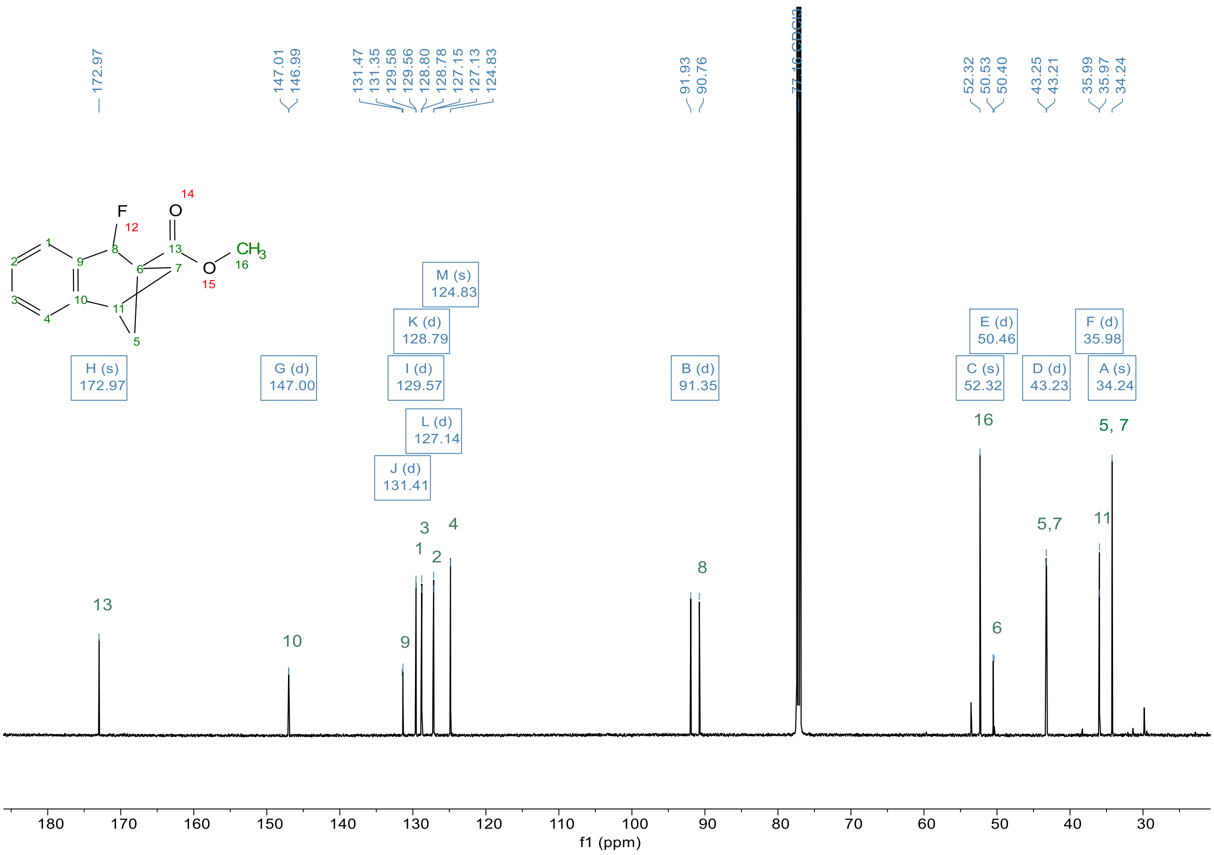

Fig.S35. ^{13}C NMR Spectrum of **44** (Chloroform-d, 298 K).

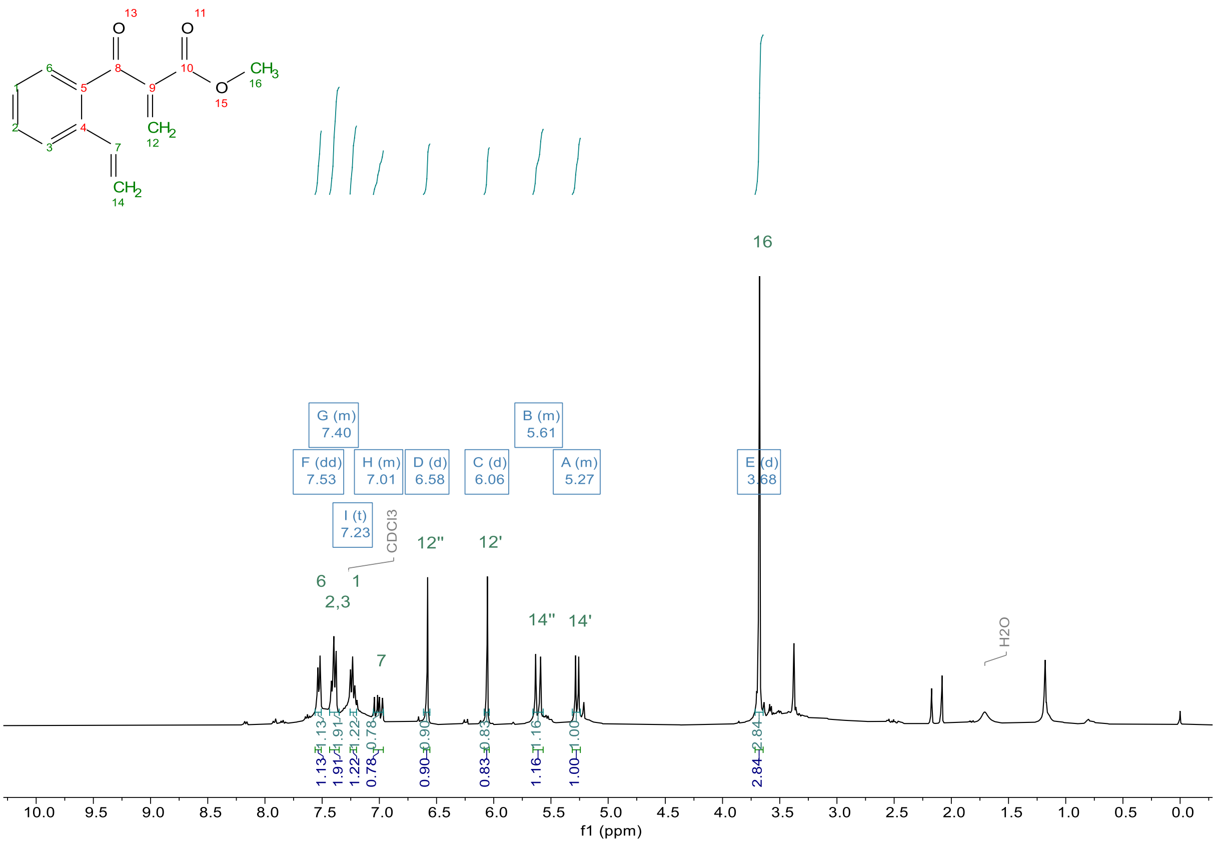

Fig.S36. ^{1}H NMR Spectrum of **S26** (Chloroform-d, 298 K).

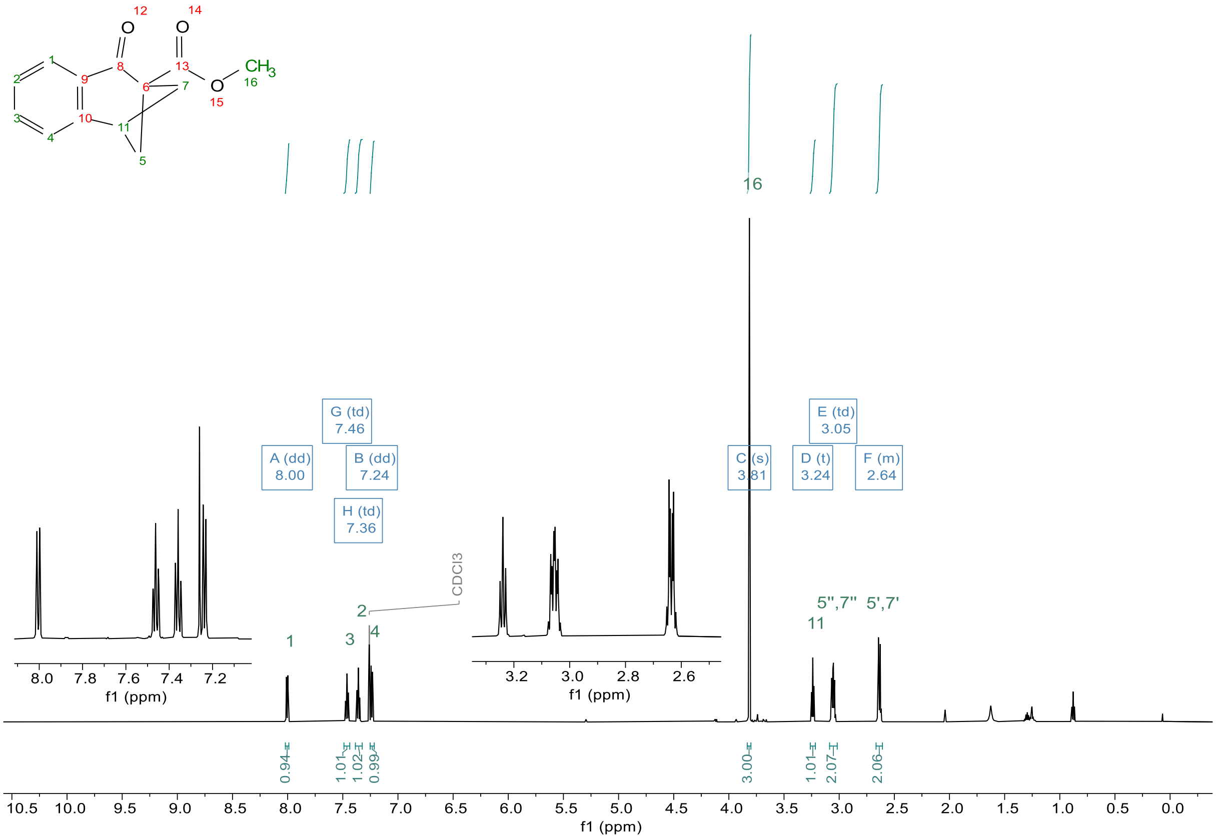

Fig.S37. ^{1}H NMR Spectrum of **20** (Chloroform-d, 298 K).

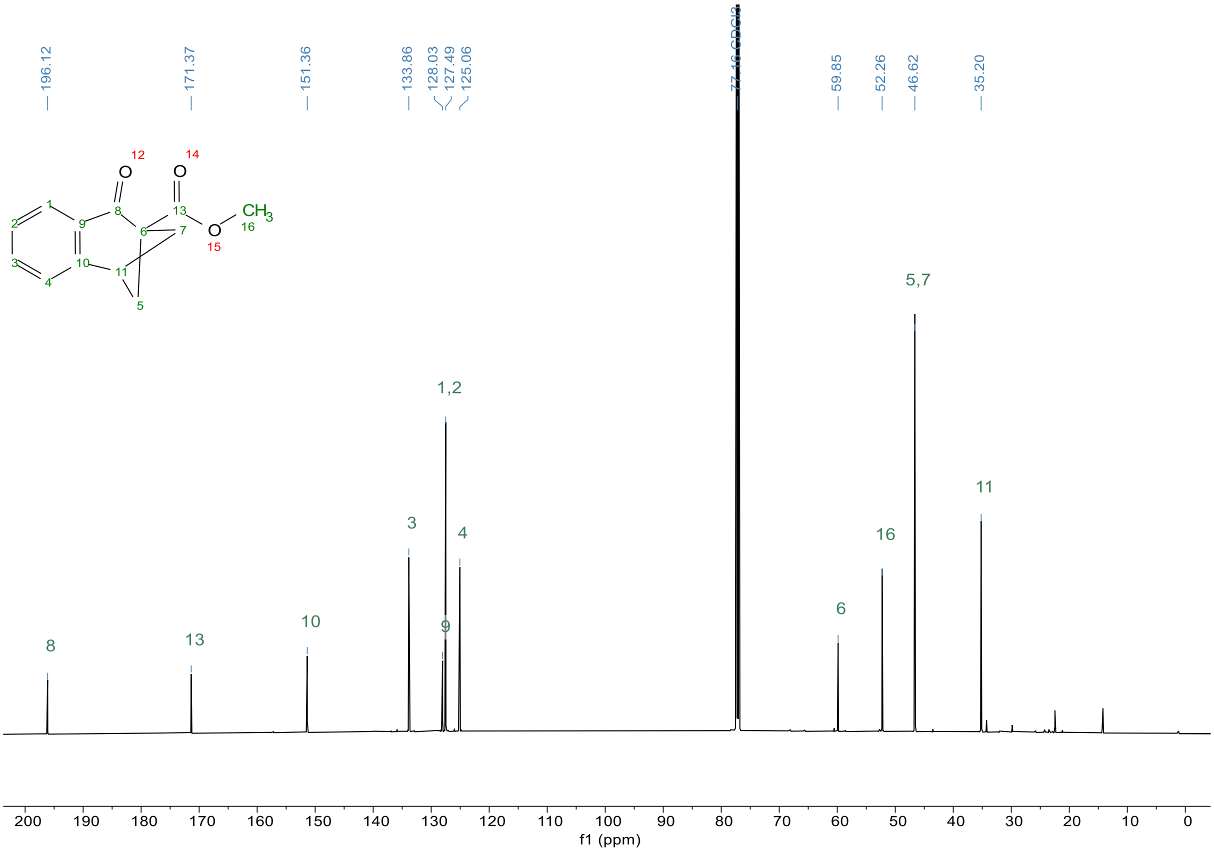

Fig.S38. ^{13}C NMR Spectrum of **20** (Chloroform-d, 298 K).

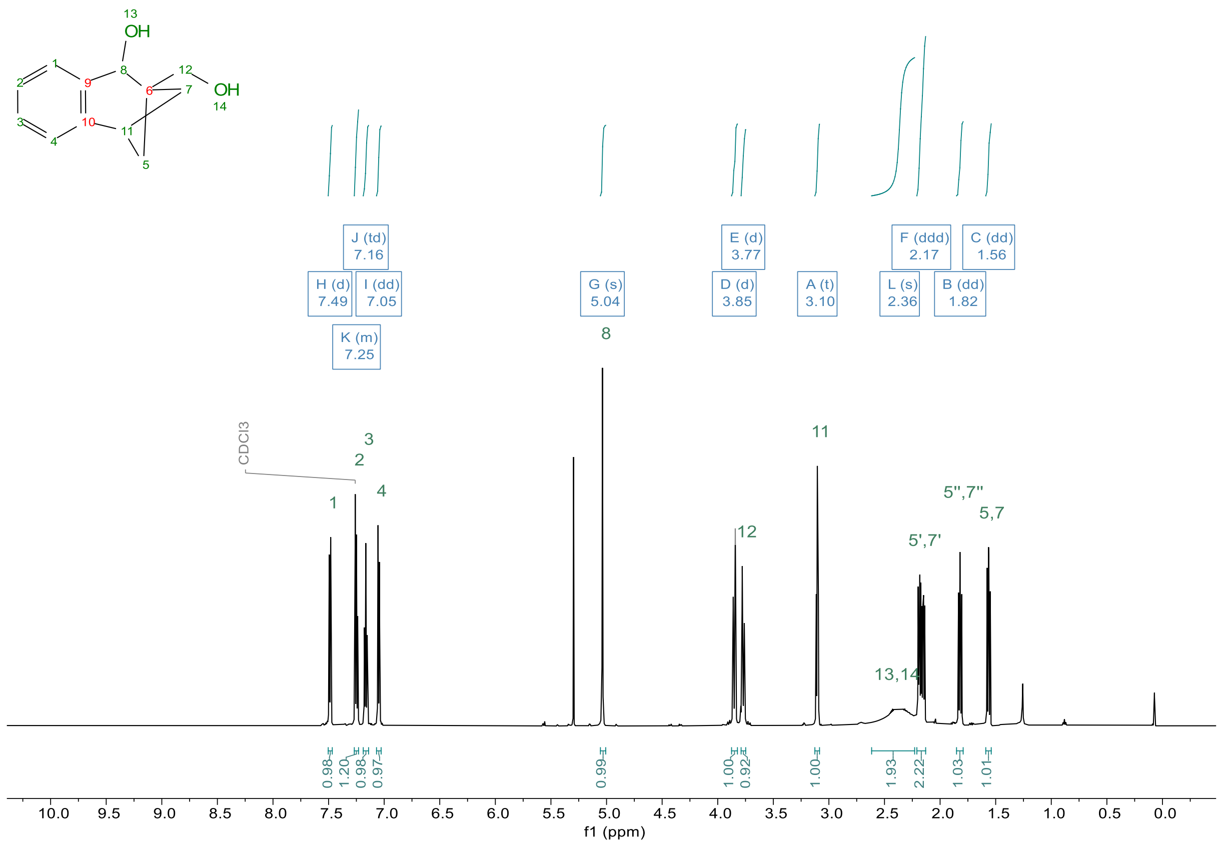

Fig.S39. ^{1}H NMR Spectrum of **S27** (Chloroform-d, 298 K).

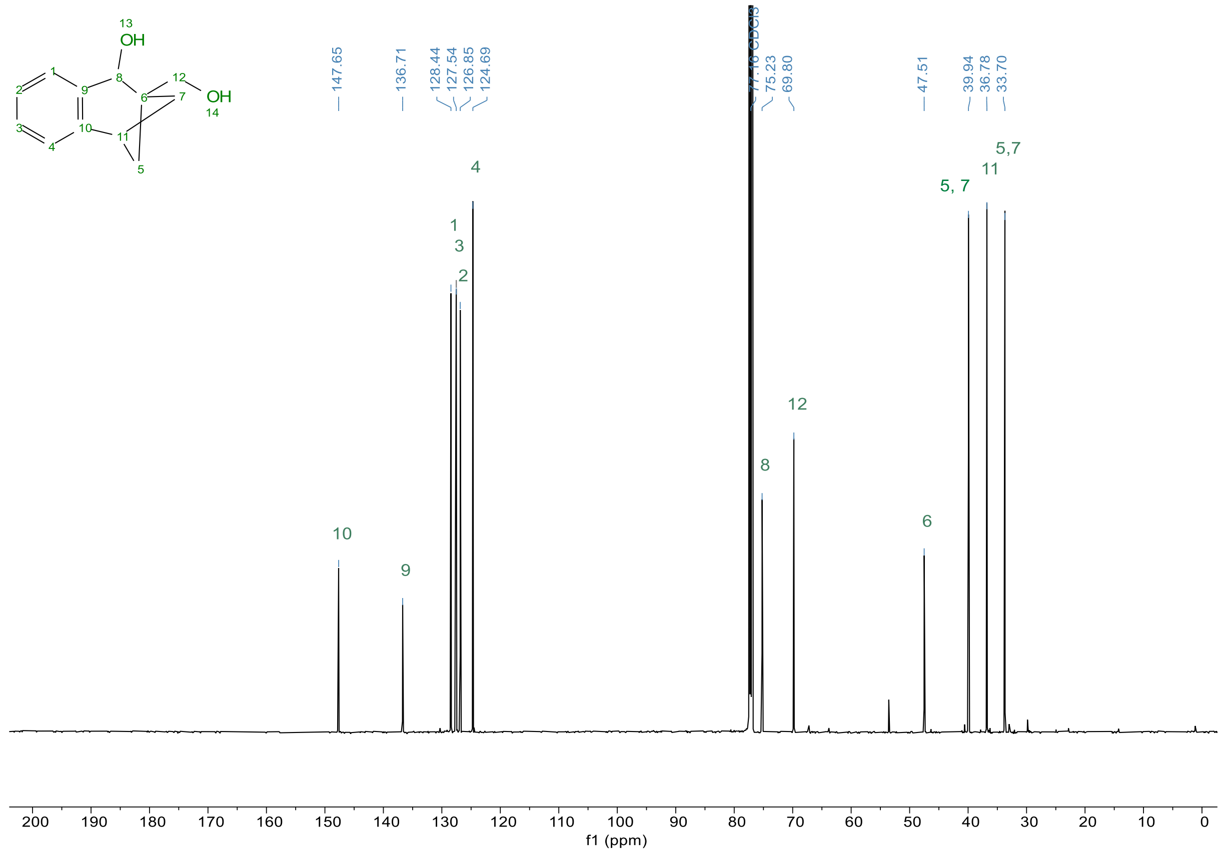

Fig.S40. ^{13}C NMR Spectrum of **S27** (Chloroform-d, 298 K).

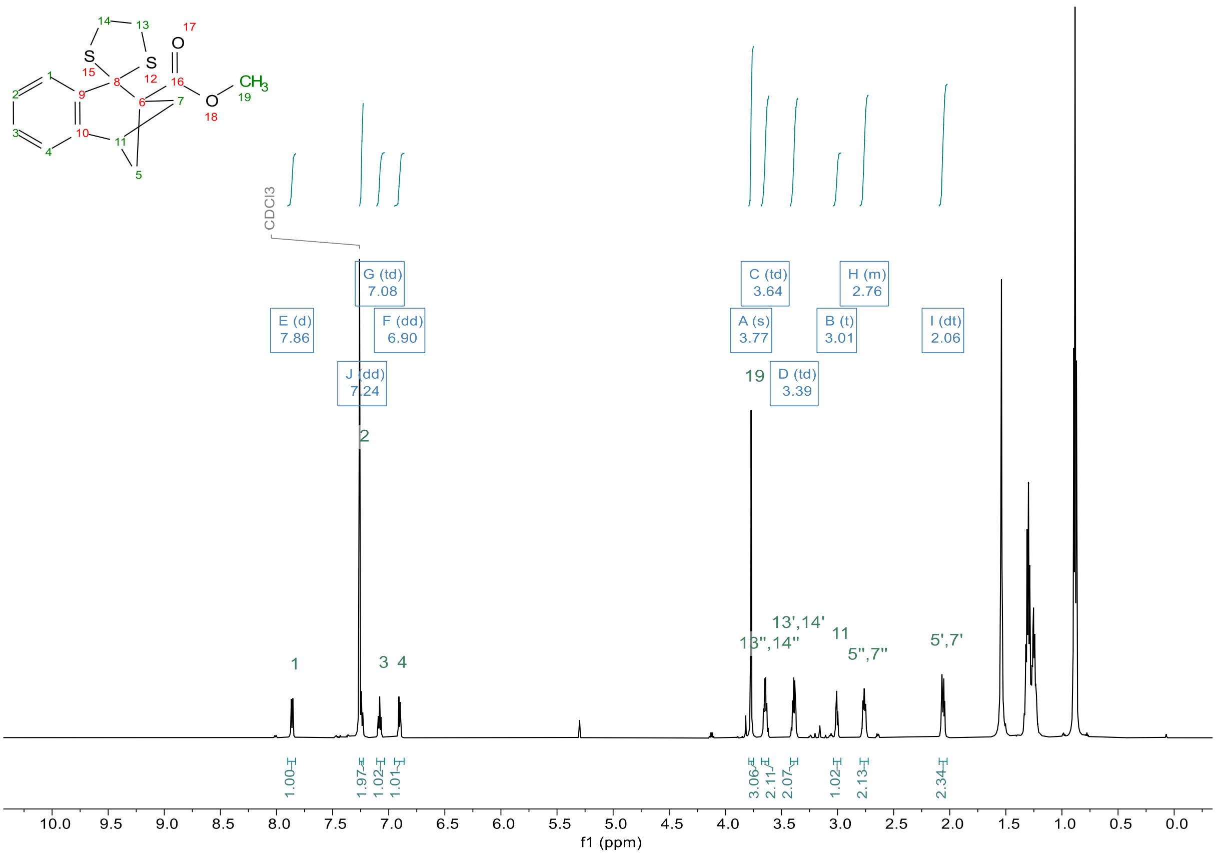

Fig.S41. ^{1}H NMR Spectrum of **45** (Chloroform-d, 298 K).

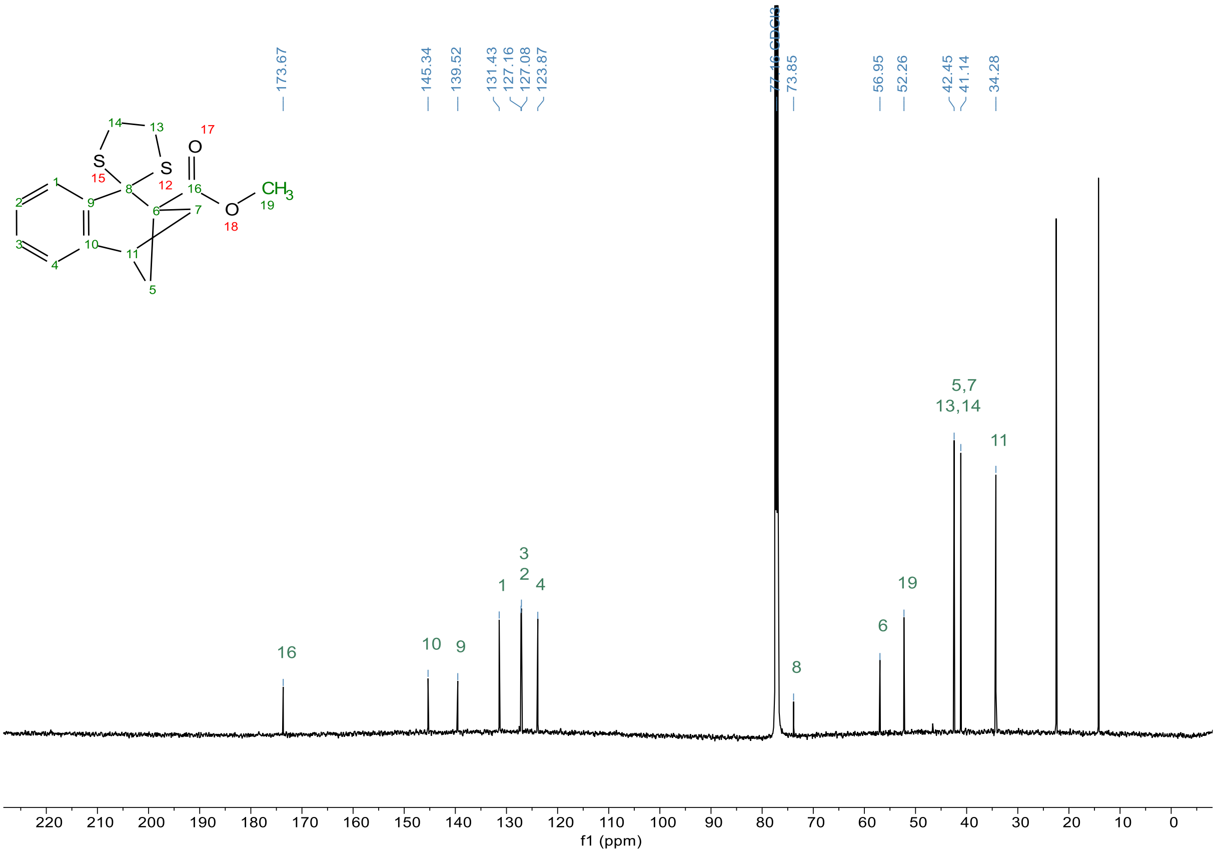

Fig.S42. ^{13}C NMR Spectrum of **45** (Chloroform-d, 298 K).

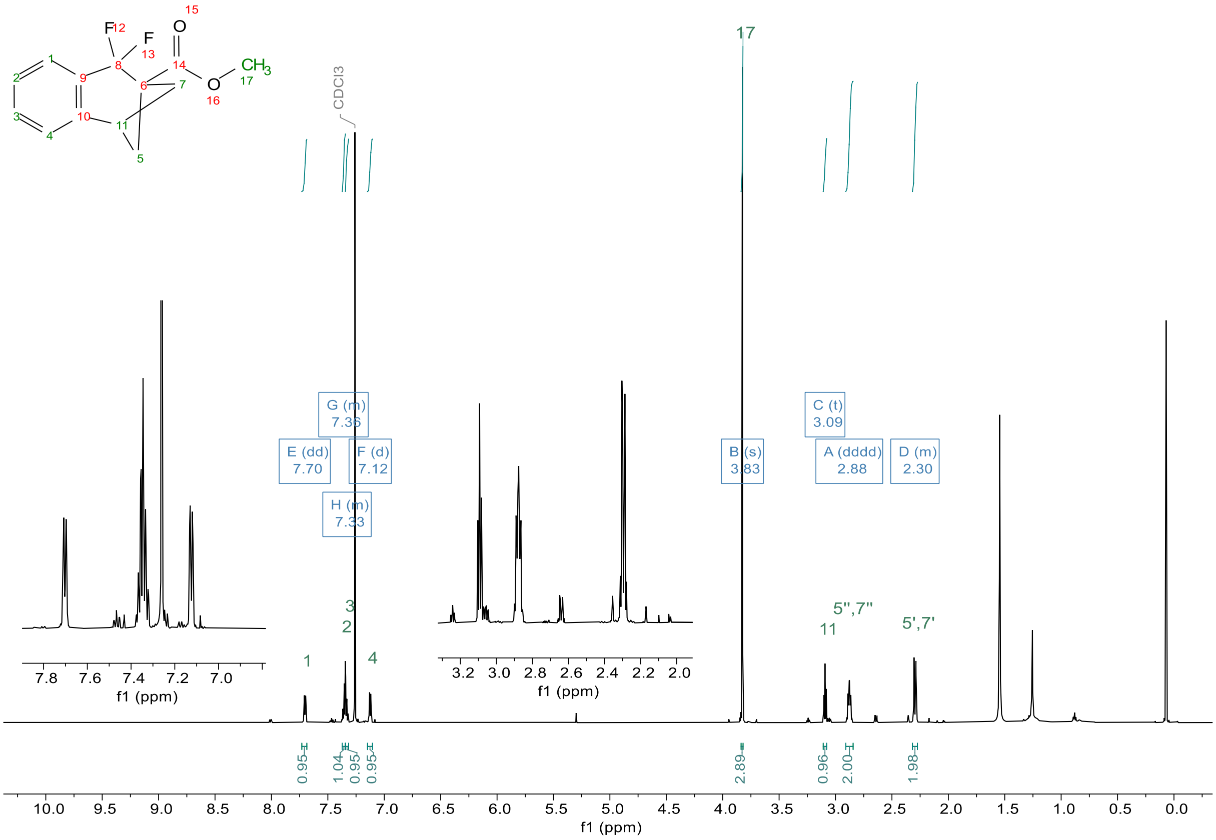

Fig.S43. ^{1}H NMR Spectrum of **46** (Chloroform-d, 298 K).

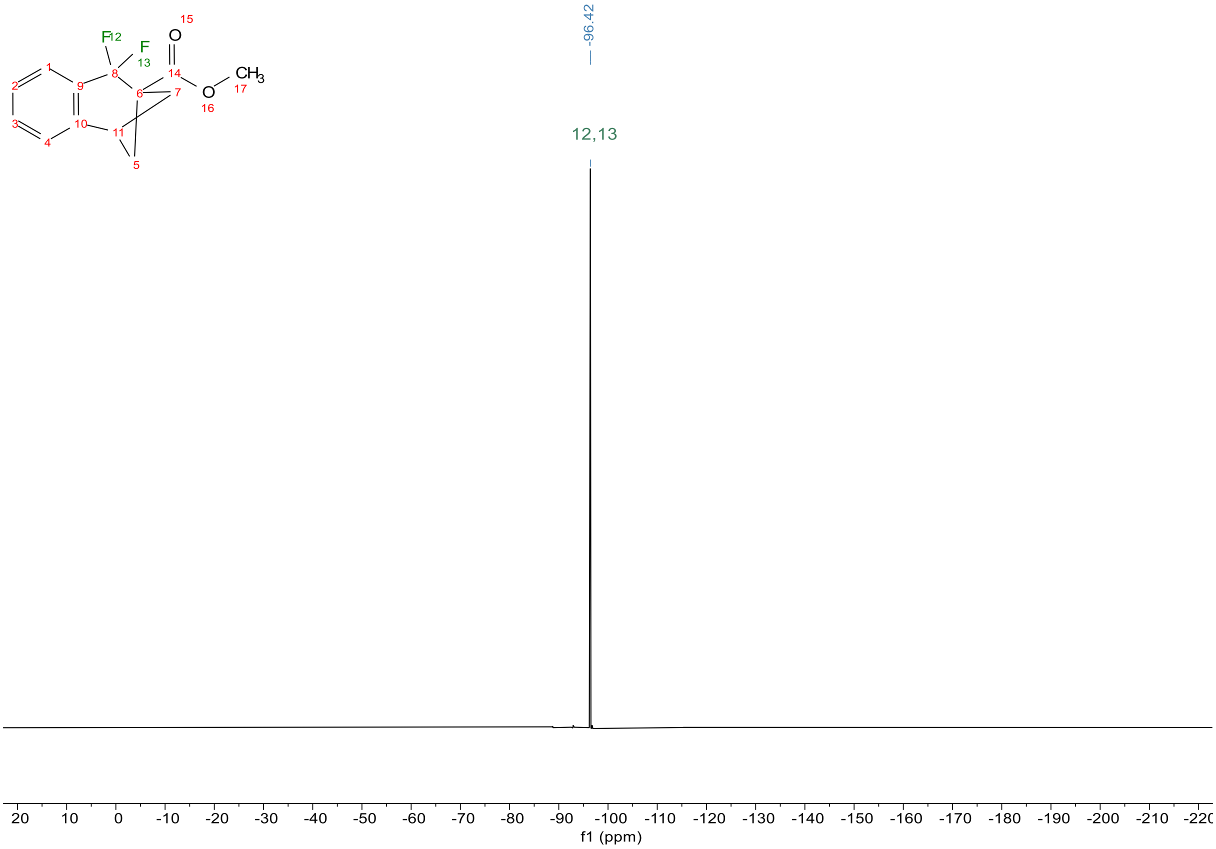

Fig.S44. ^{19}F NMR Spectrum of **46** (Chloroform-d, 298 K).

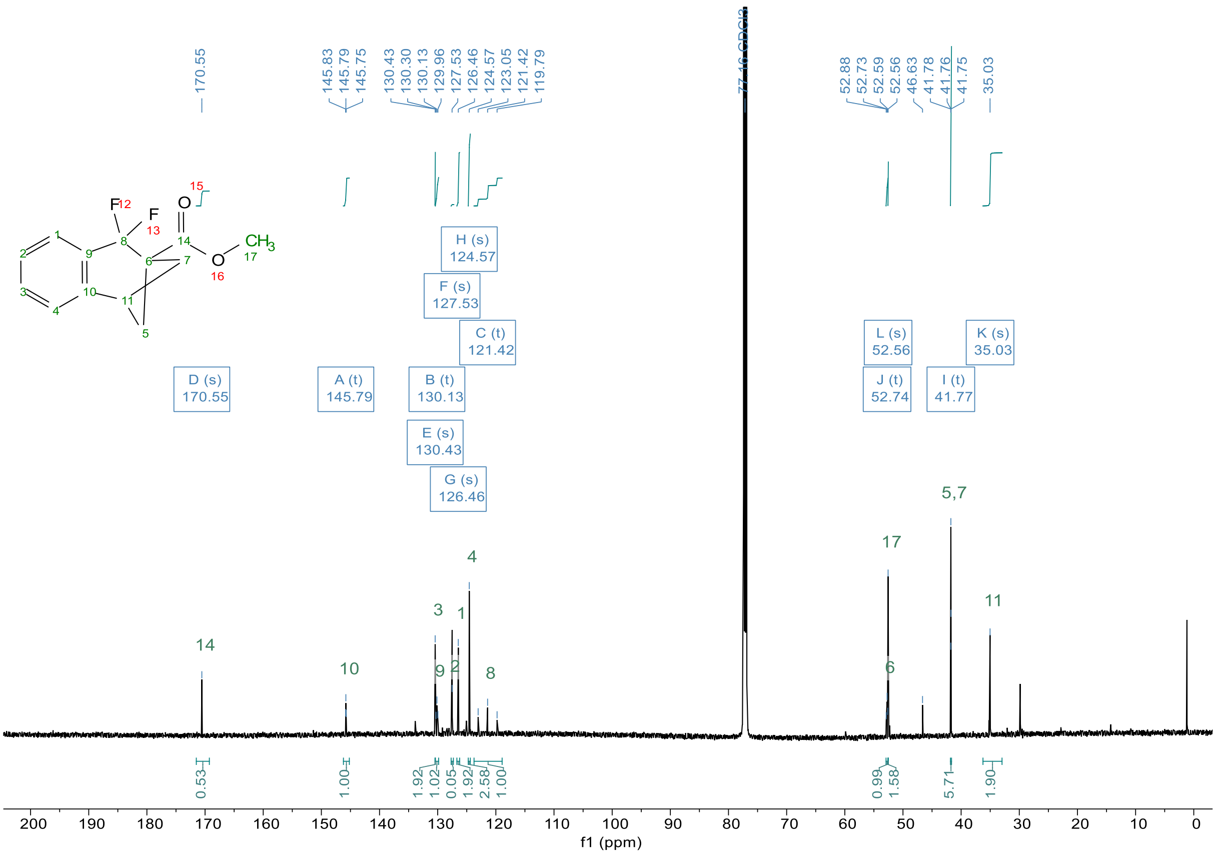

Fig.S45. ^{13}C NMR Spectrum of **46** (Chloroform-d, 298 K).

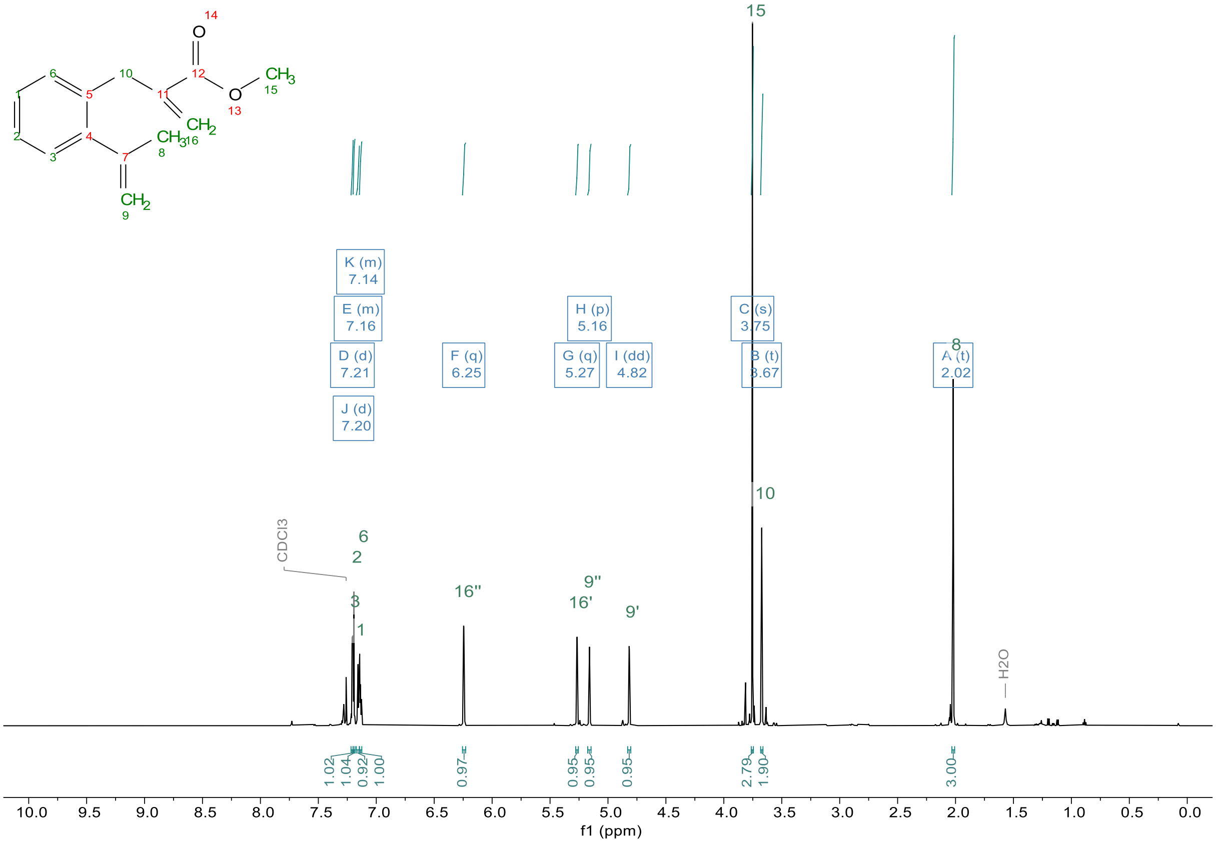

Fig.S46. ^{1}H NMR Spectrum of **S28** (Chloroform-d, 298 K).

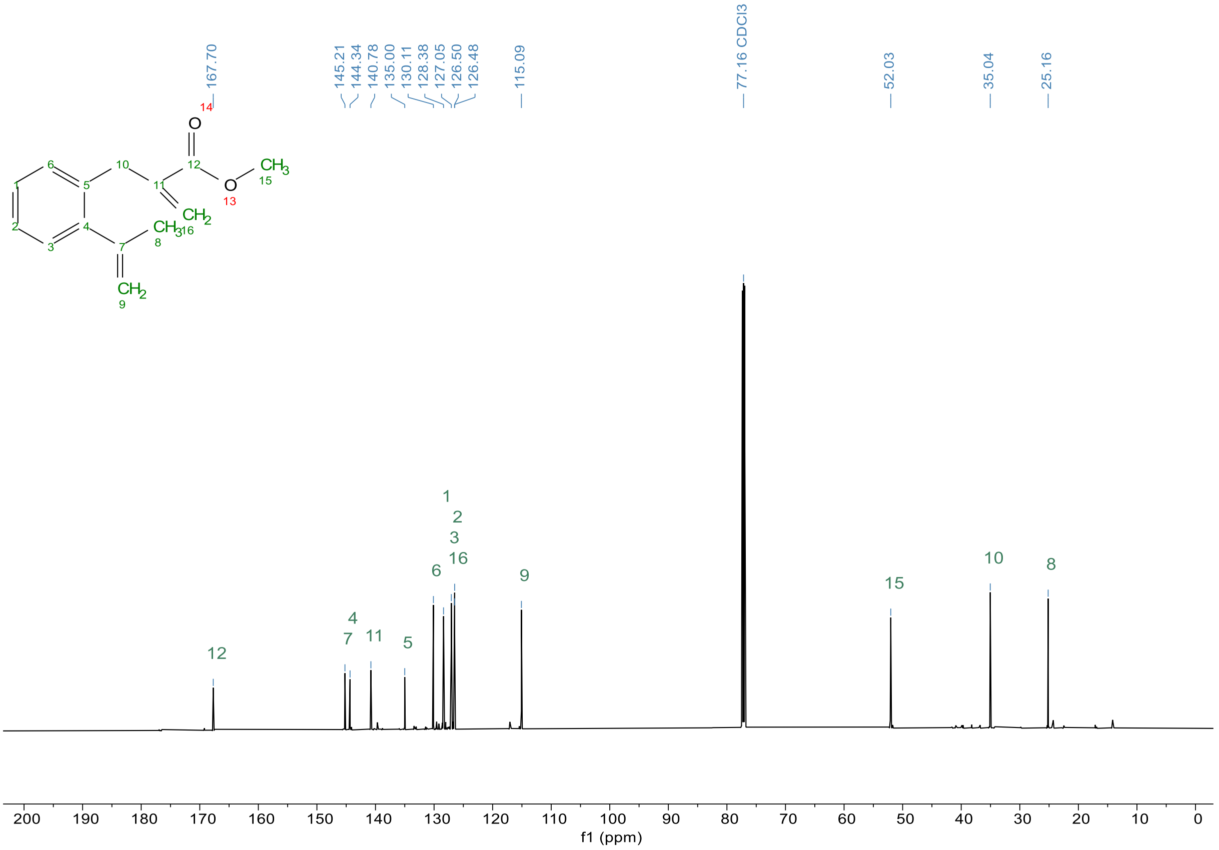

Fig.S47. ^{13}C NMR Spectrum of **S28** (Chloroform-d, 298 K).

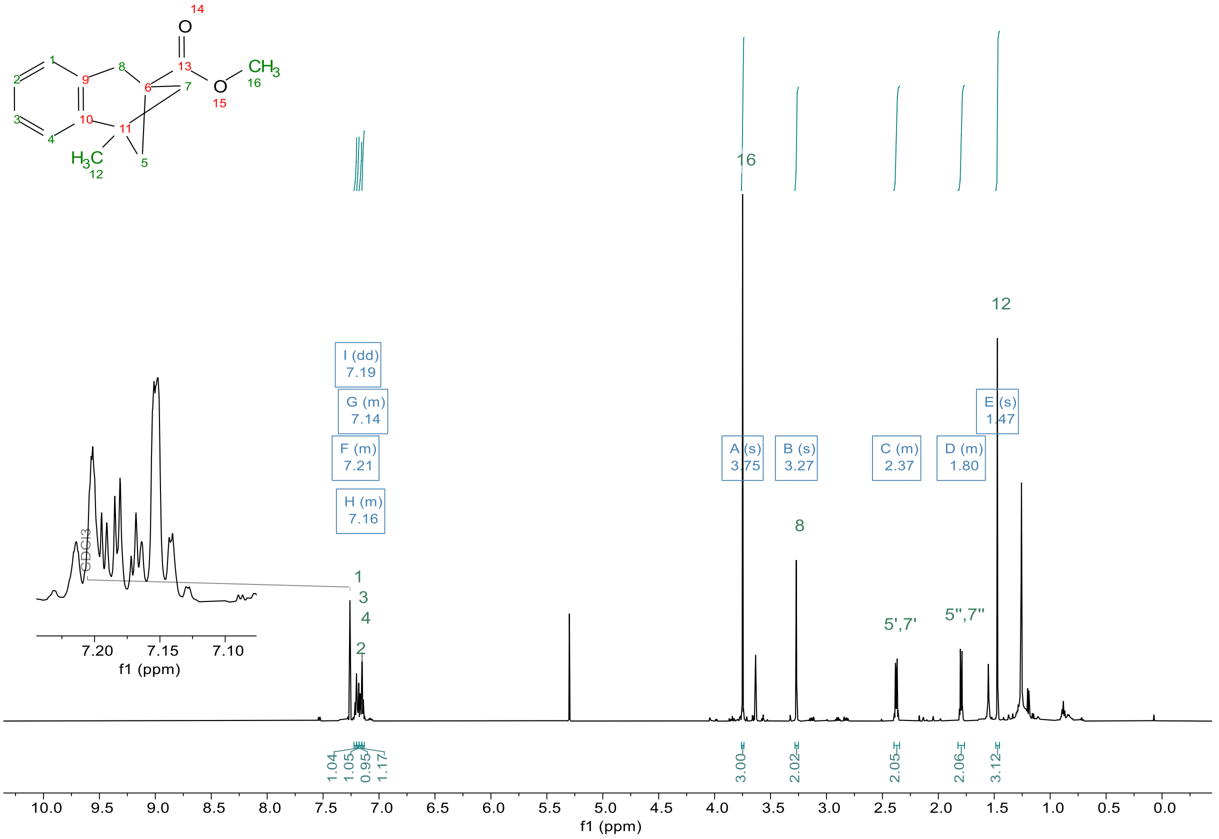

Fig.S48. ^{1}H NMR Spectrum of **21** (Chloroform-d, 298 K).

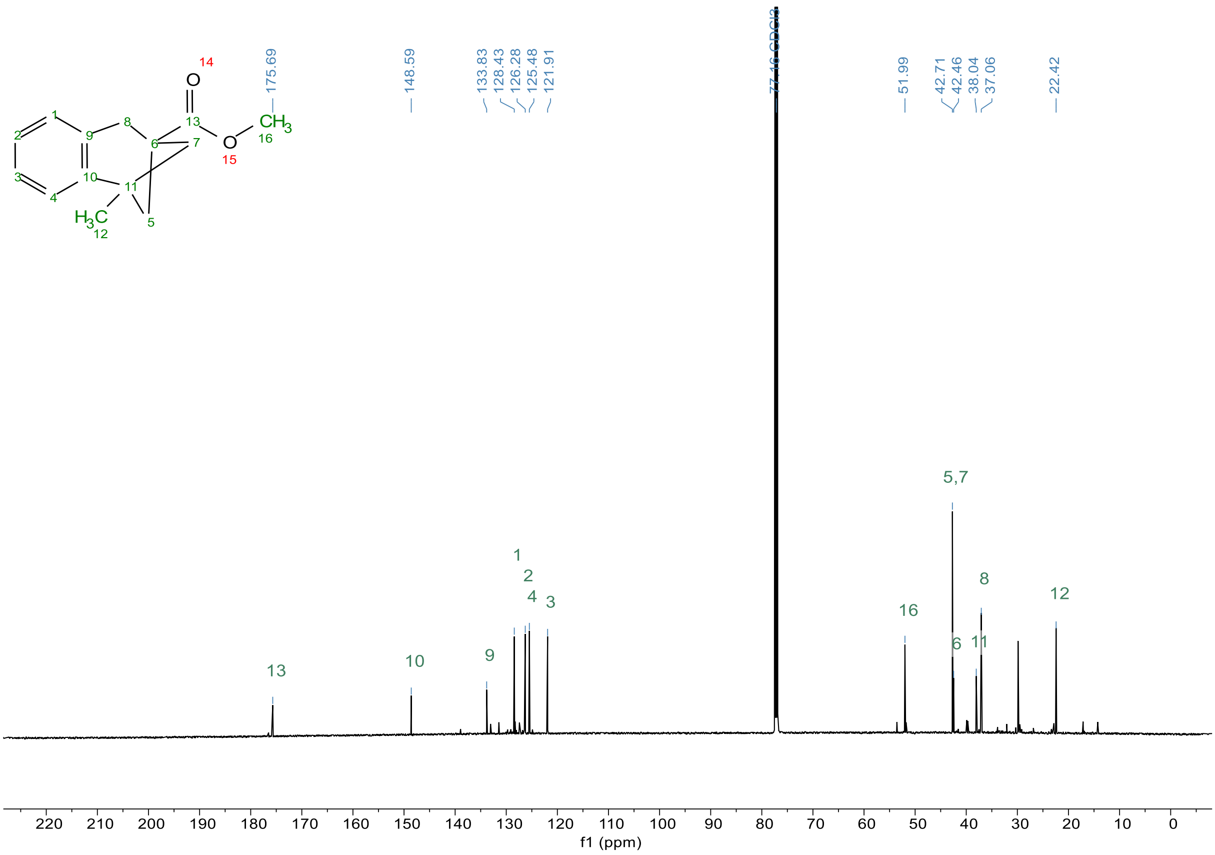

Fig.S49. ^{13}C NMR Spectrum of **21** (Chloroform-d, 298 K).

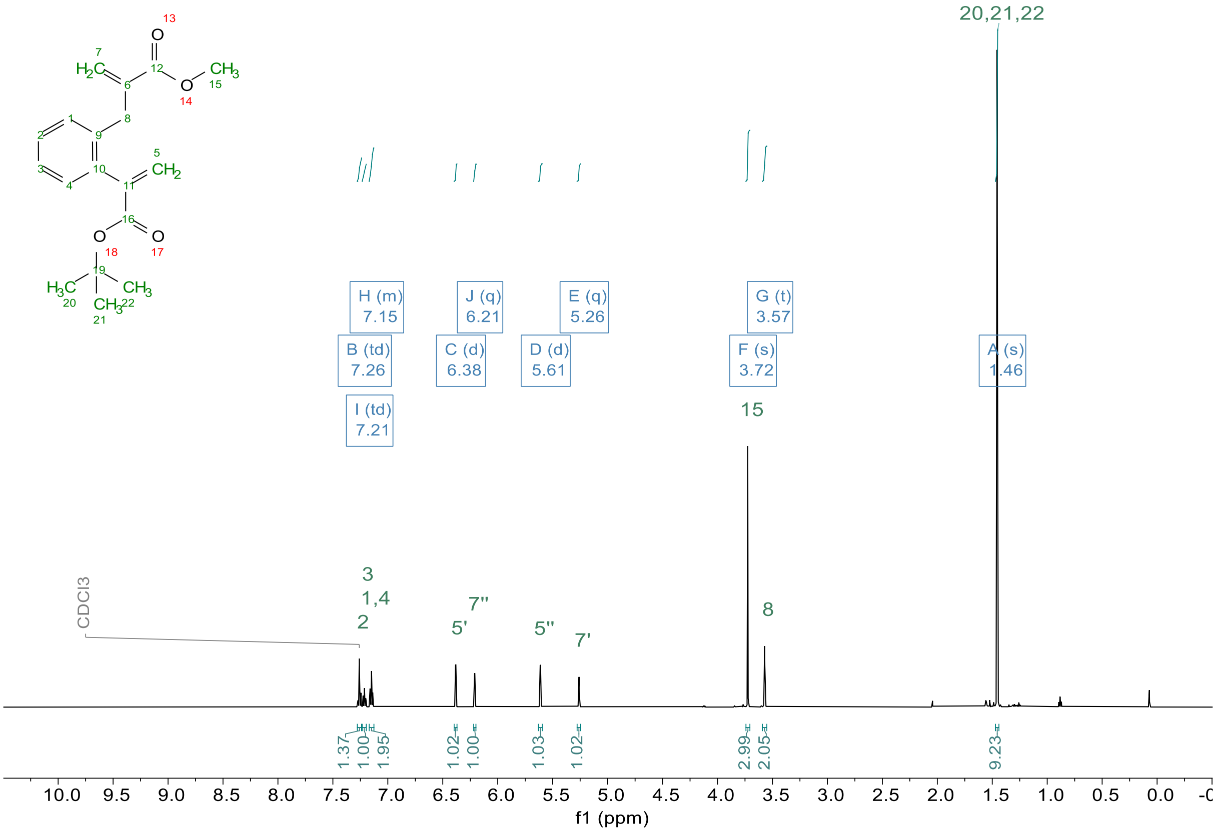

Fig.S50. ^{1}H NMR Spectrum of **S29** (Chloroform-d, 298 K).

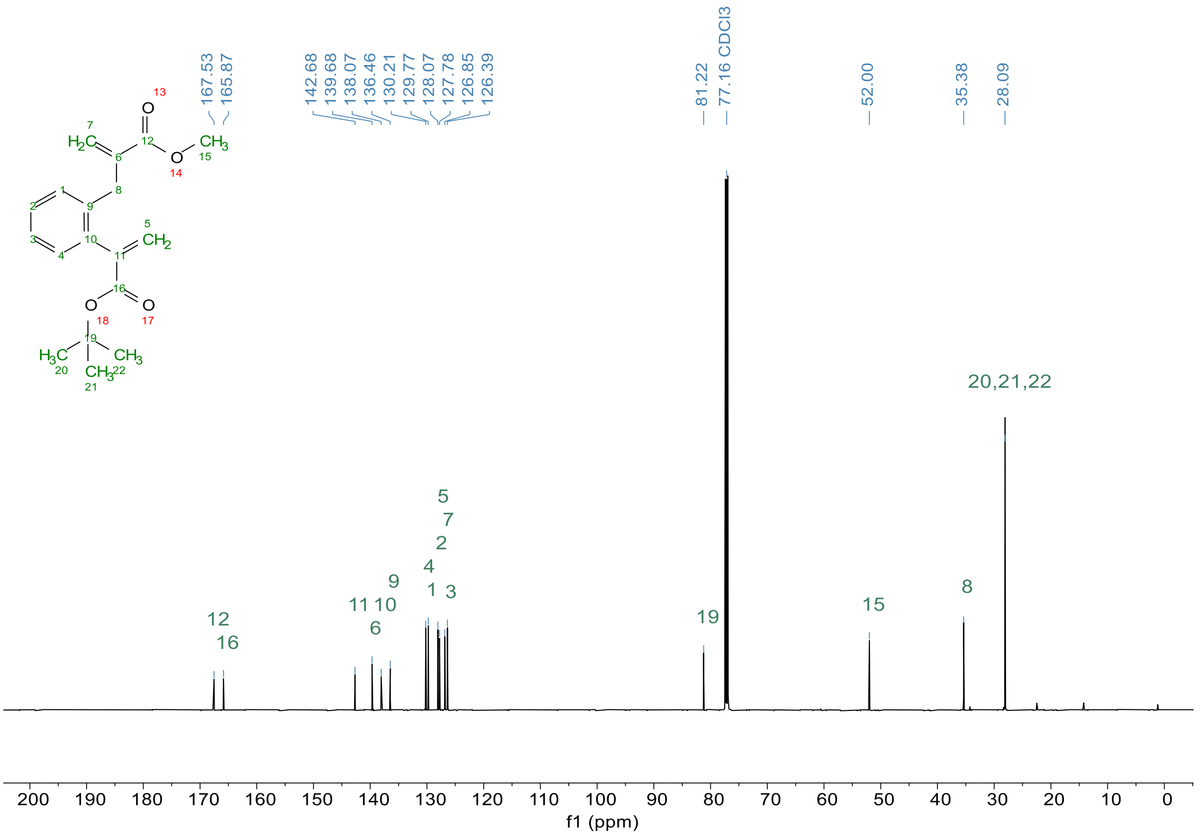

Fig.S51. ^{13}C NMR Spectrum of **S29** (Chloroform-d, 298 K).

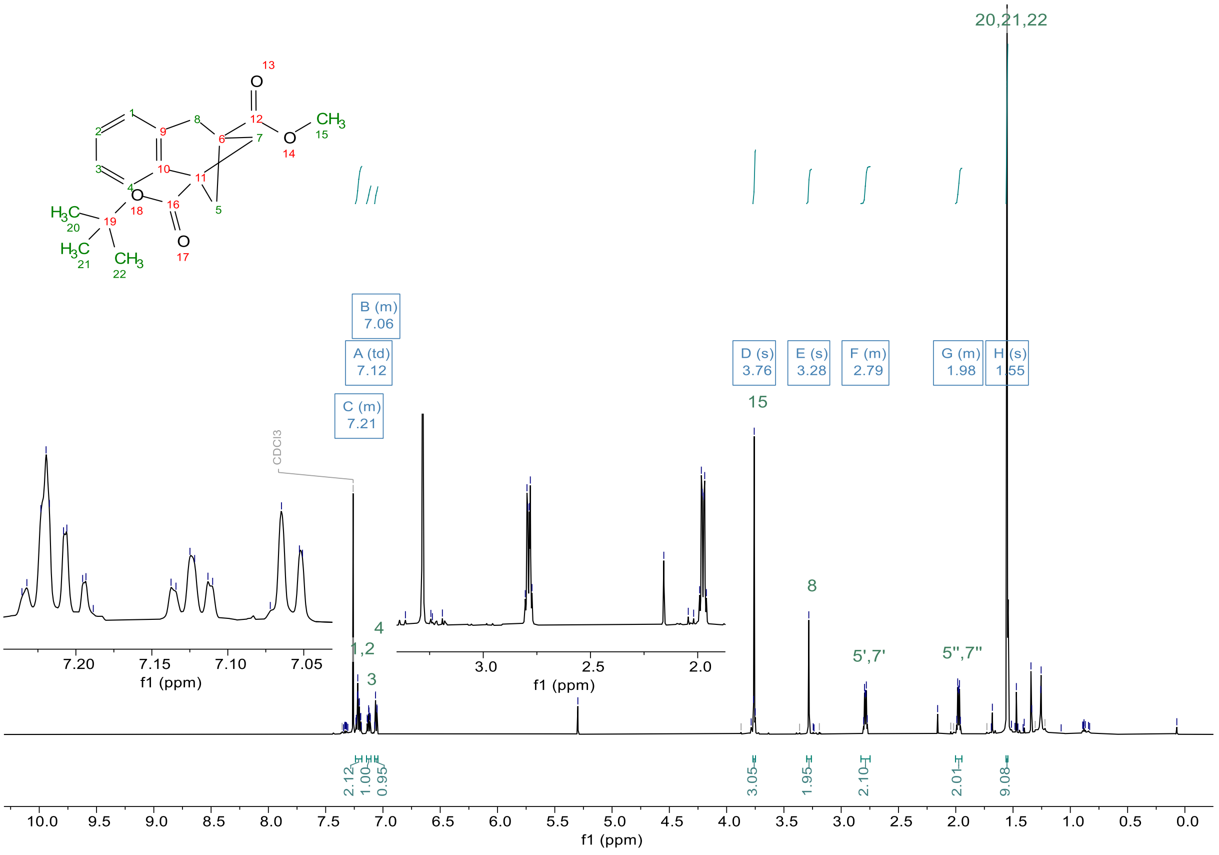

Fig.S52. ^{1}H NMR Spectrum of **22** (Chloroform-d, 298 K).

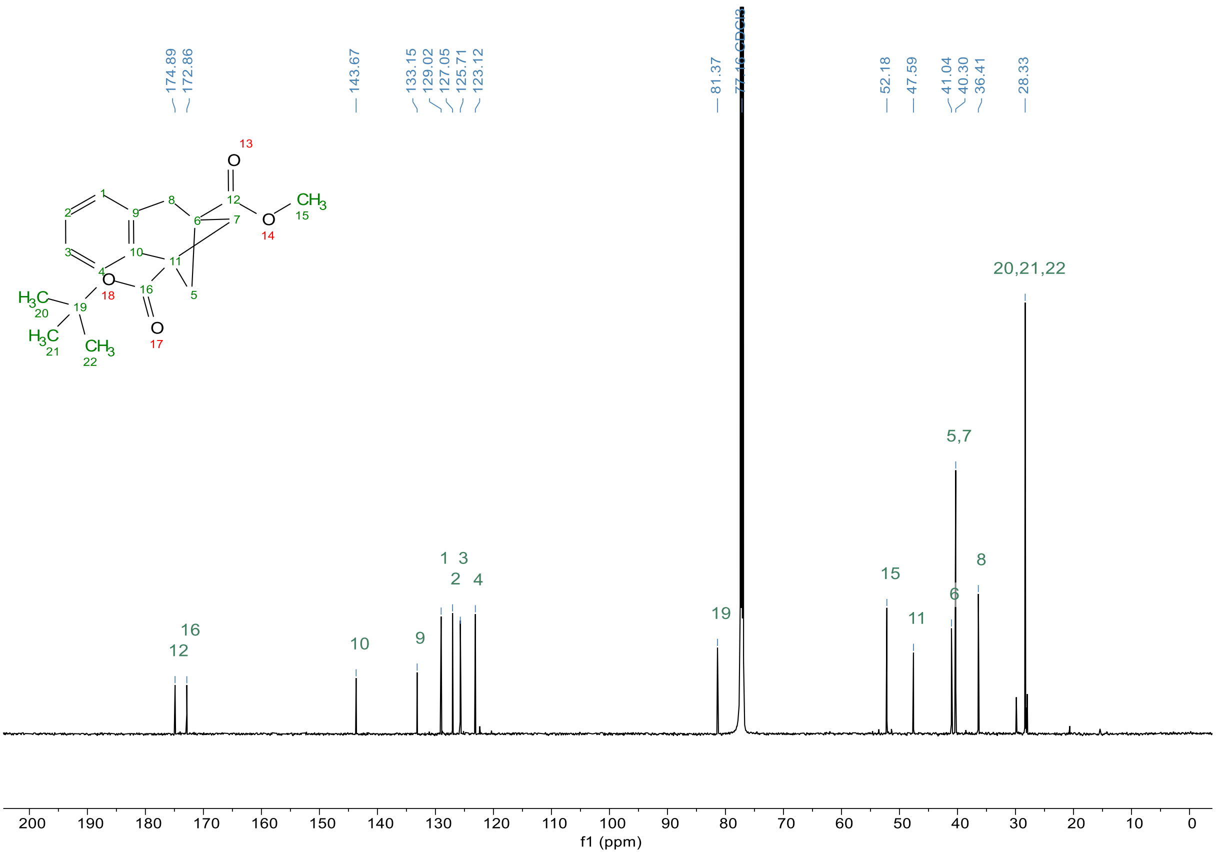

Fig.S53 ^{13}C NMR Spectrum of **22** (Chloroform-d, 298 K).

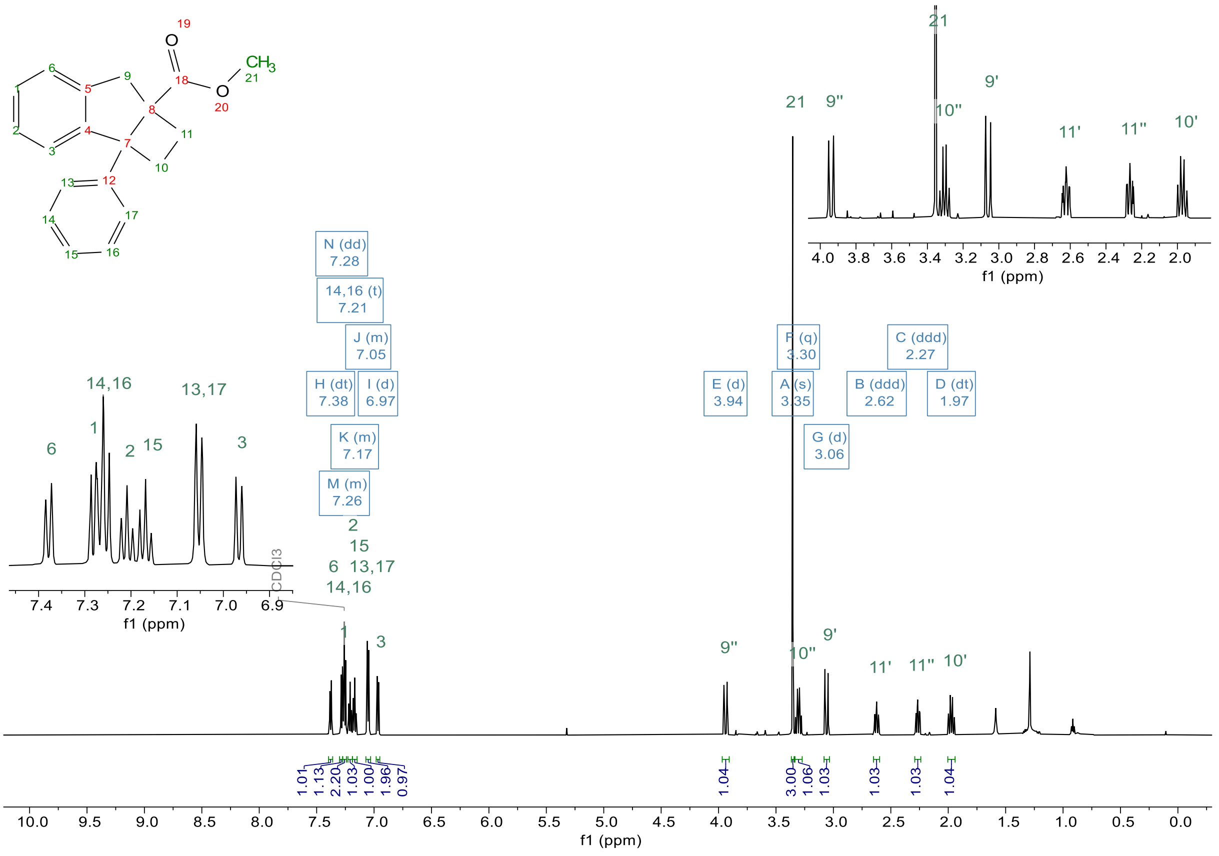

Fig.S54. ^{1}H NMR Spectrum of **23a** (Chloroform-d, 298 K).

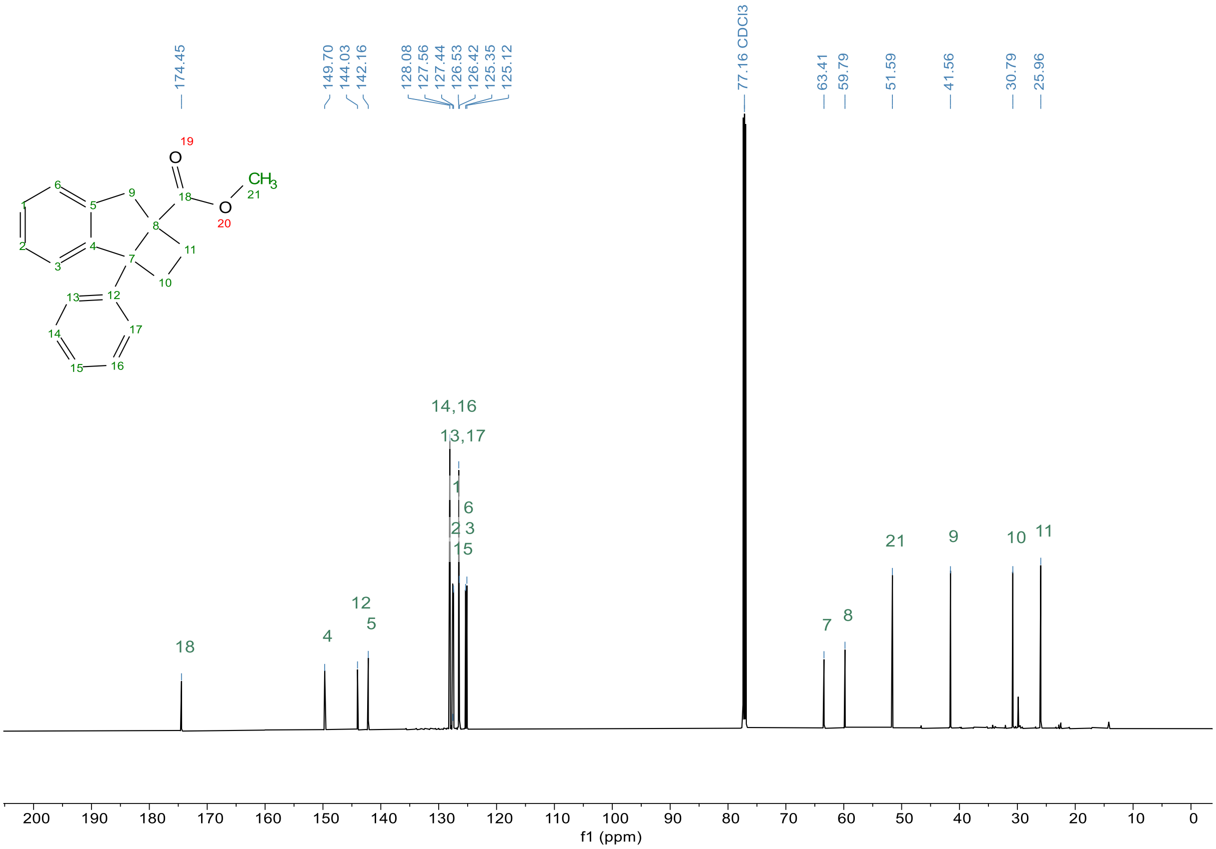

Fig.S55. ^{13}C NMR Spectrum of **23a** (Chloroform-d, 298 K).

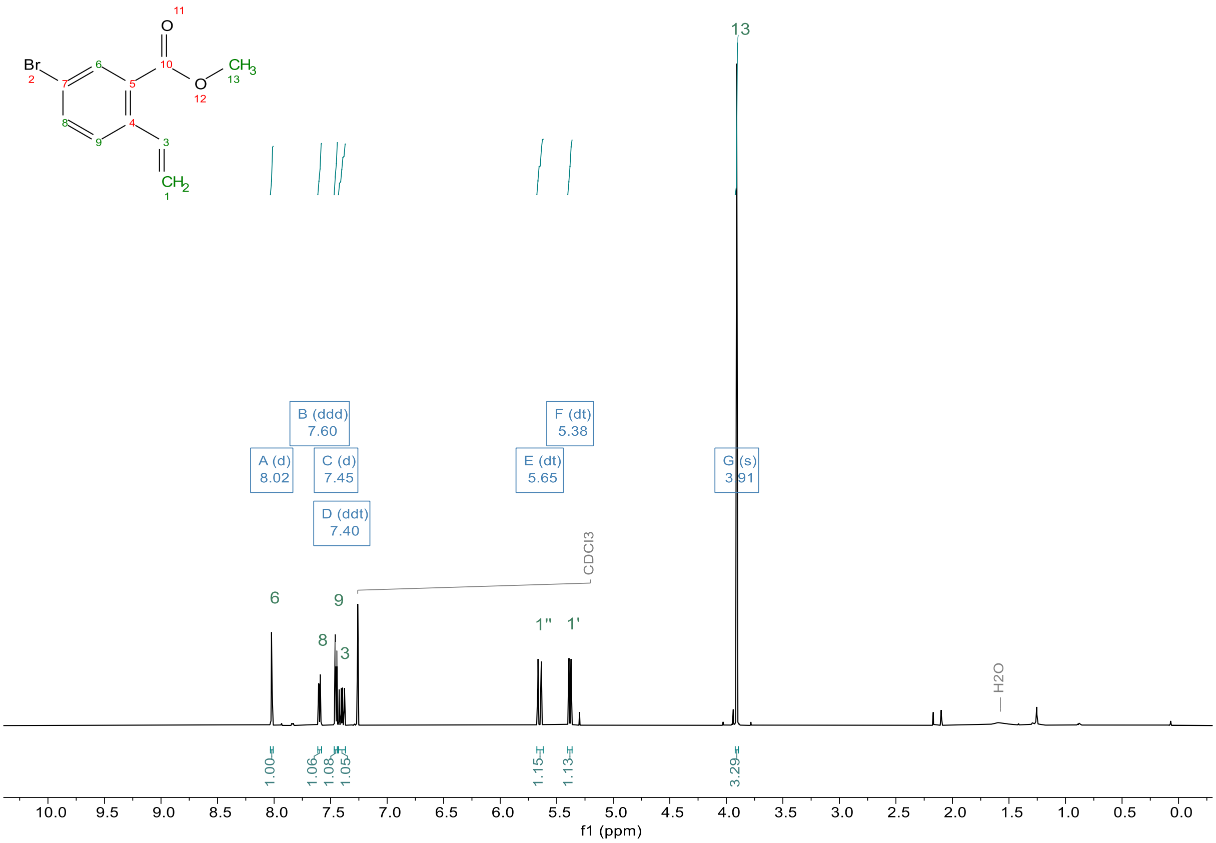

Fig.S56. ^{1}H NMR Spectrum of **S32** (Chloroform-d, 298 K).

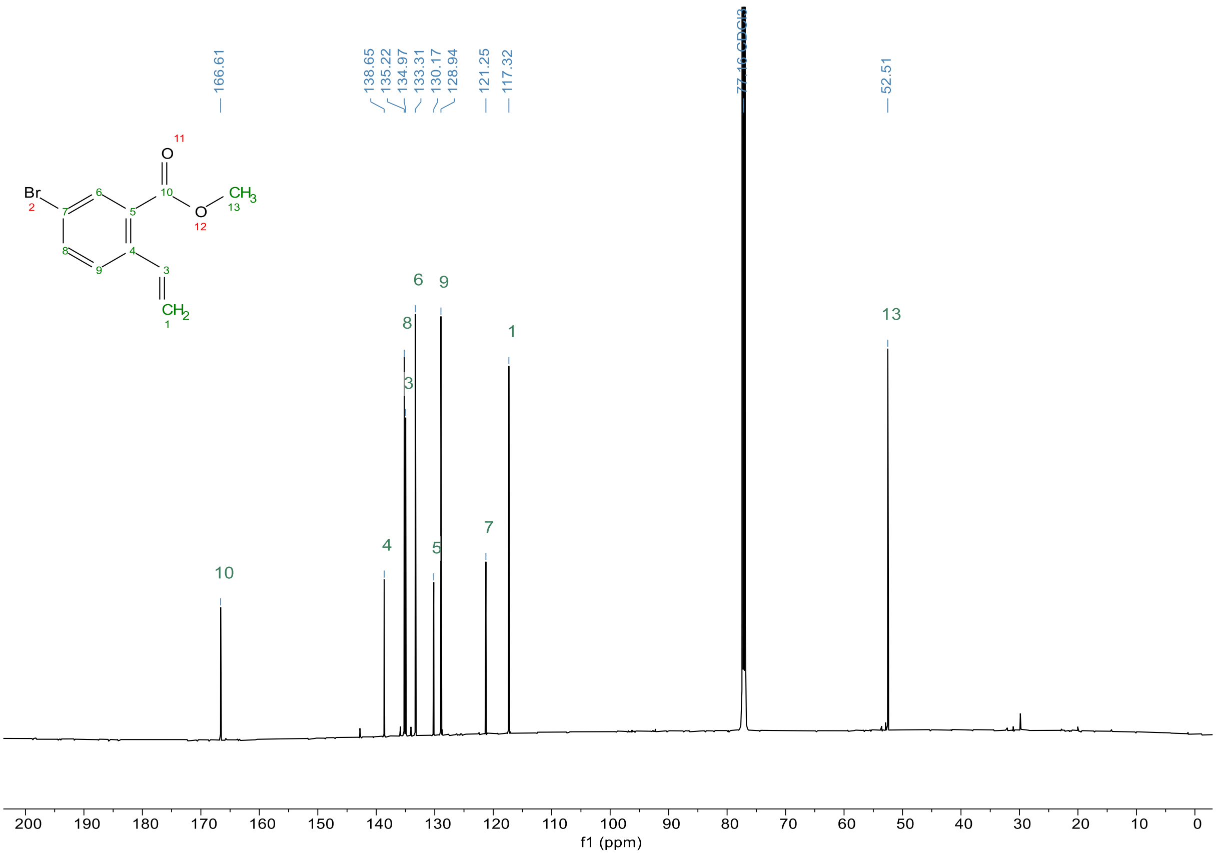

Fig.S57. ^{13}C NMR Spectrum of **S32** (Chloroform-d, 298 K).

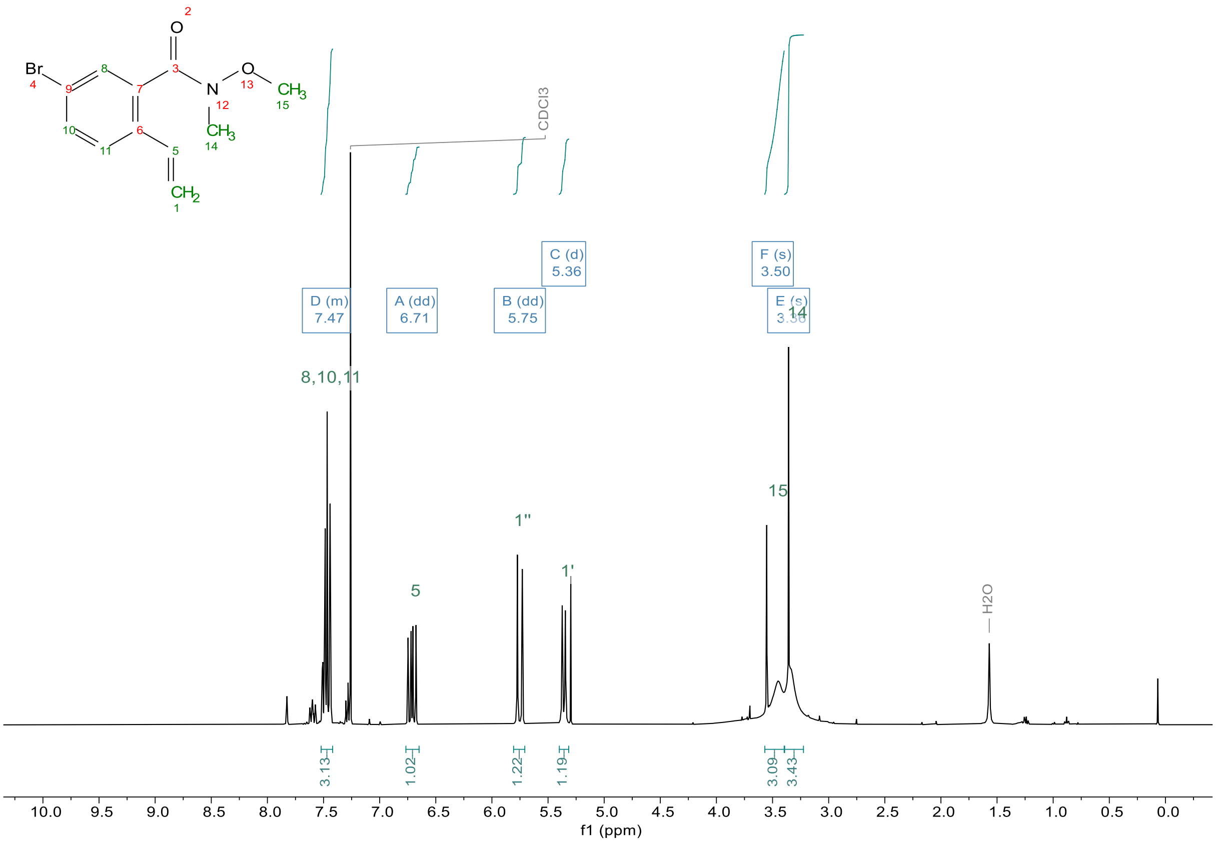

Fig.S58. ^{1}H NMR Spectrum **S33** (Chloroform-d, 298 K).

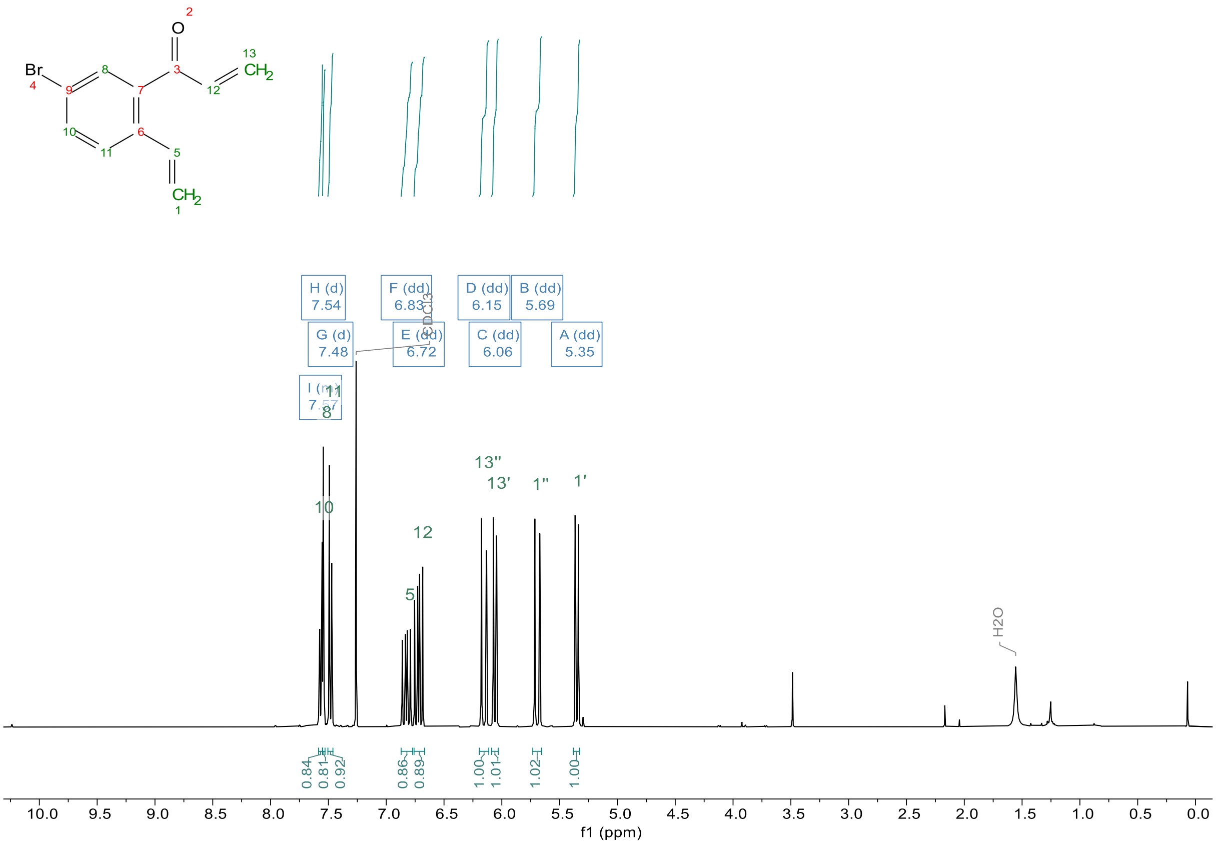

Fig.S59. ^{1}H NMR Spectrum of **S34** (Chloroform-d, 298 K).

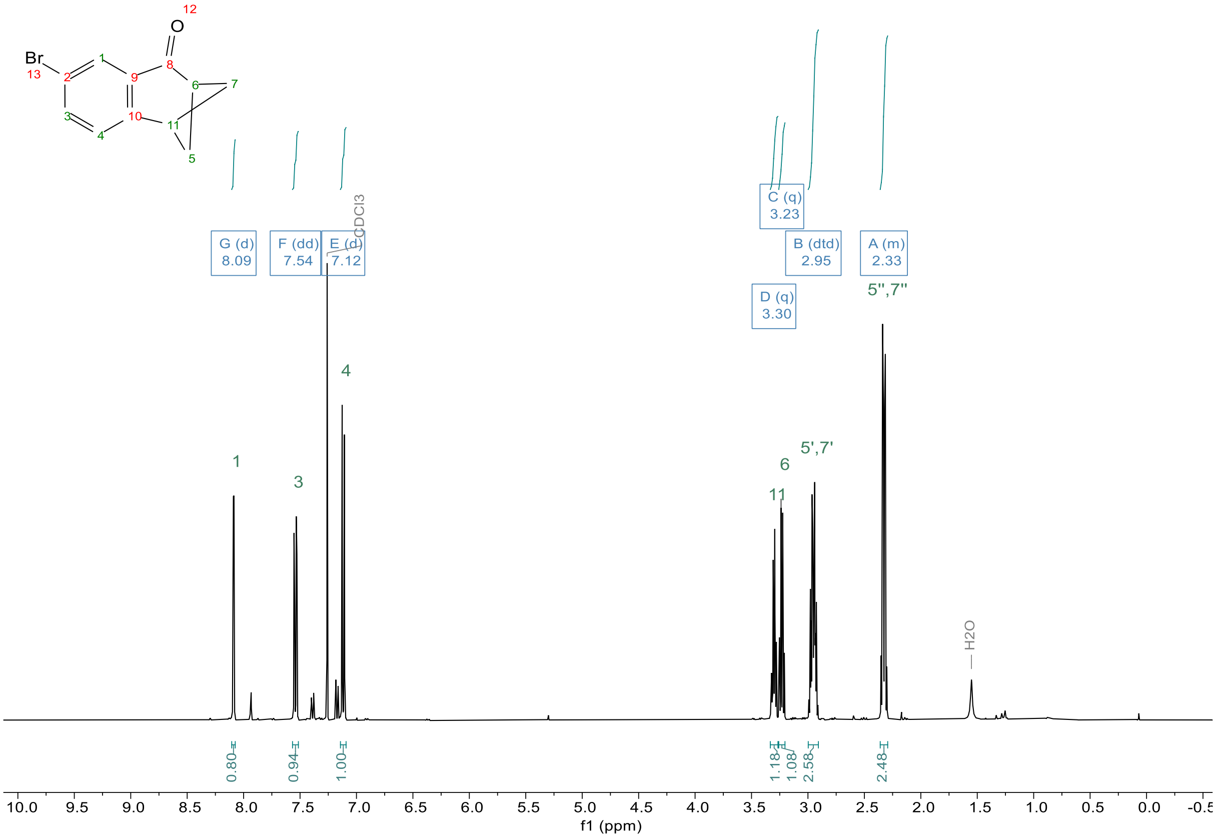

Fig.S60. ^{1}H NMR Spectrum of **24** (Chloroform-d, 298 K).

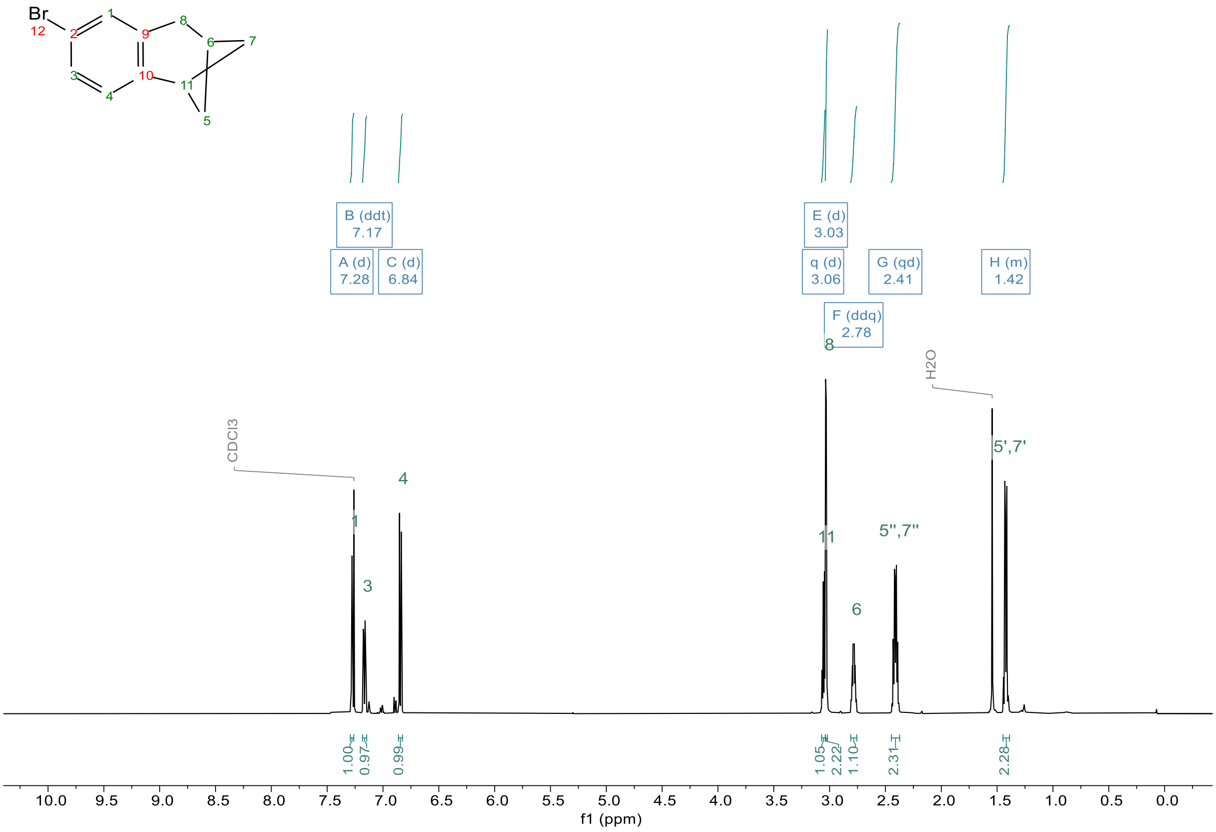

Fig.S61. ^{1}H NMR Spectrum of **29a** (Chloroform-d, 298 K).

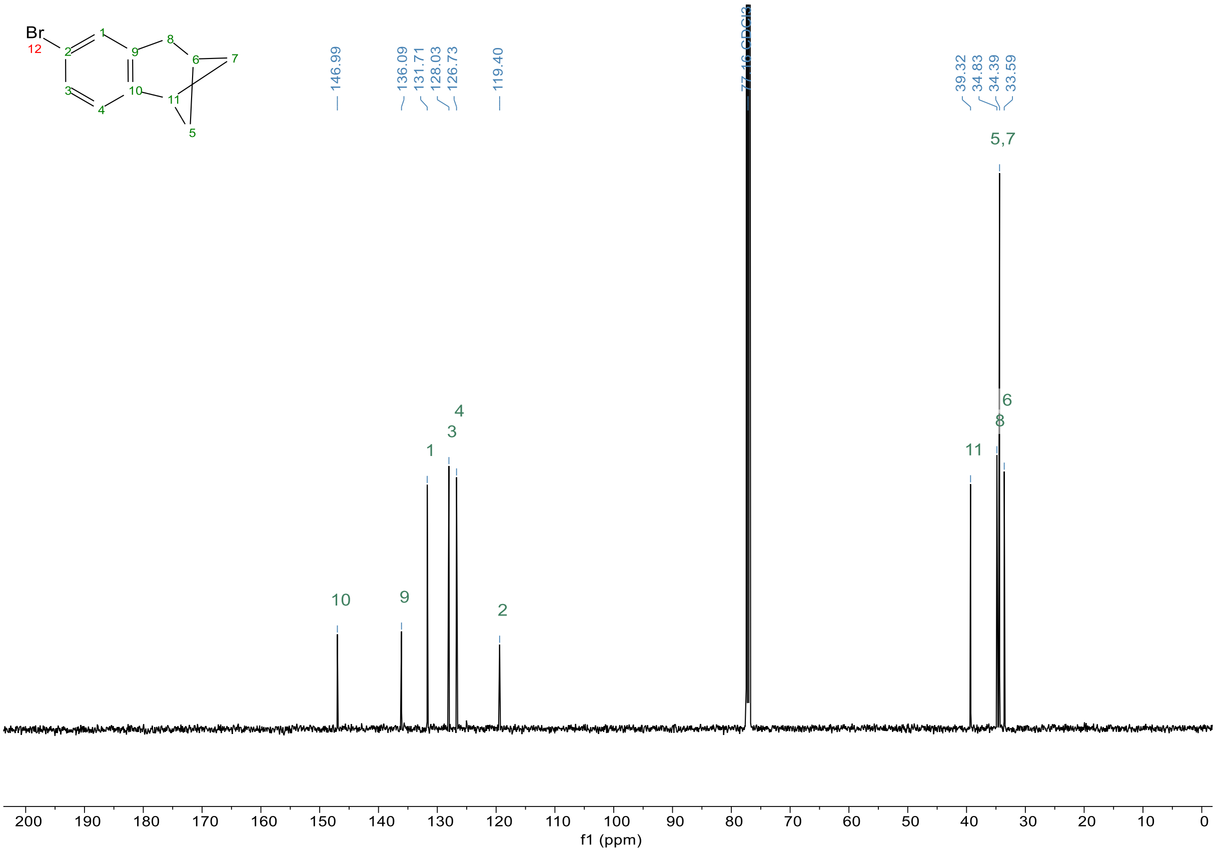

Fig.S62. ^{13}C NMR Spectrum of **29a** (Chloroform-d, 298 K).

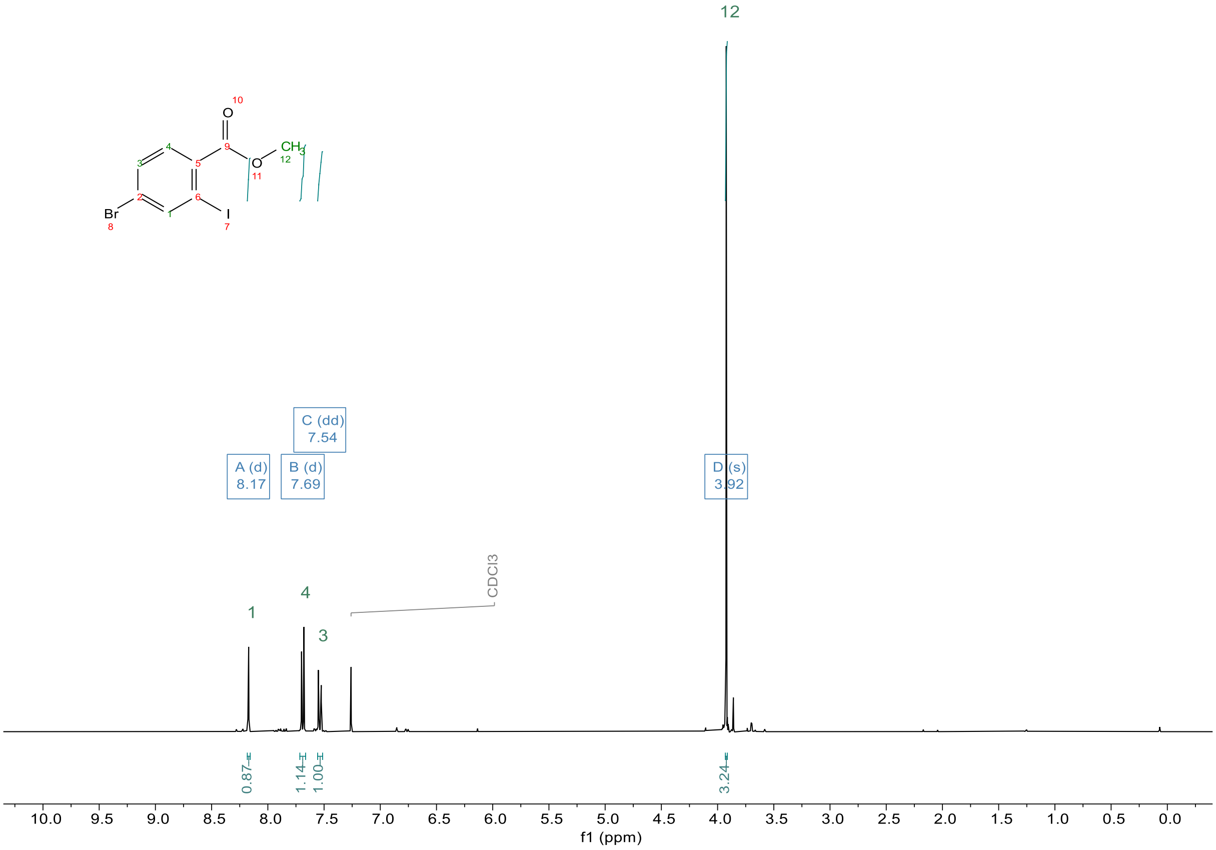

Fig.S63. ^{13}C NMR Spectrum of **S36** (Chloroform-d, 298 K).

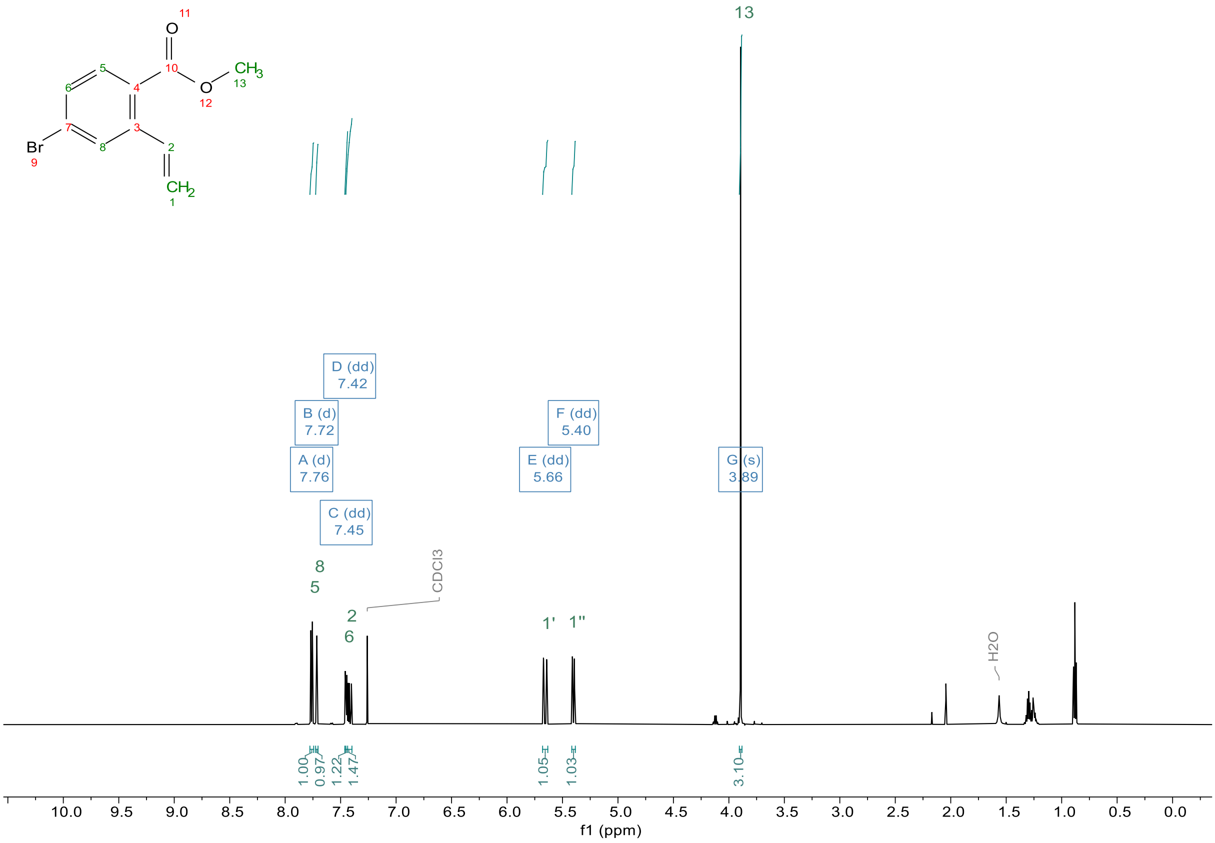

Fig.S64. ^{1}H NMR Spectrum of **S37** (Chloroform-d, 298 K).

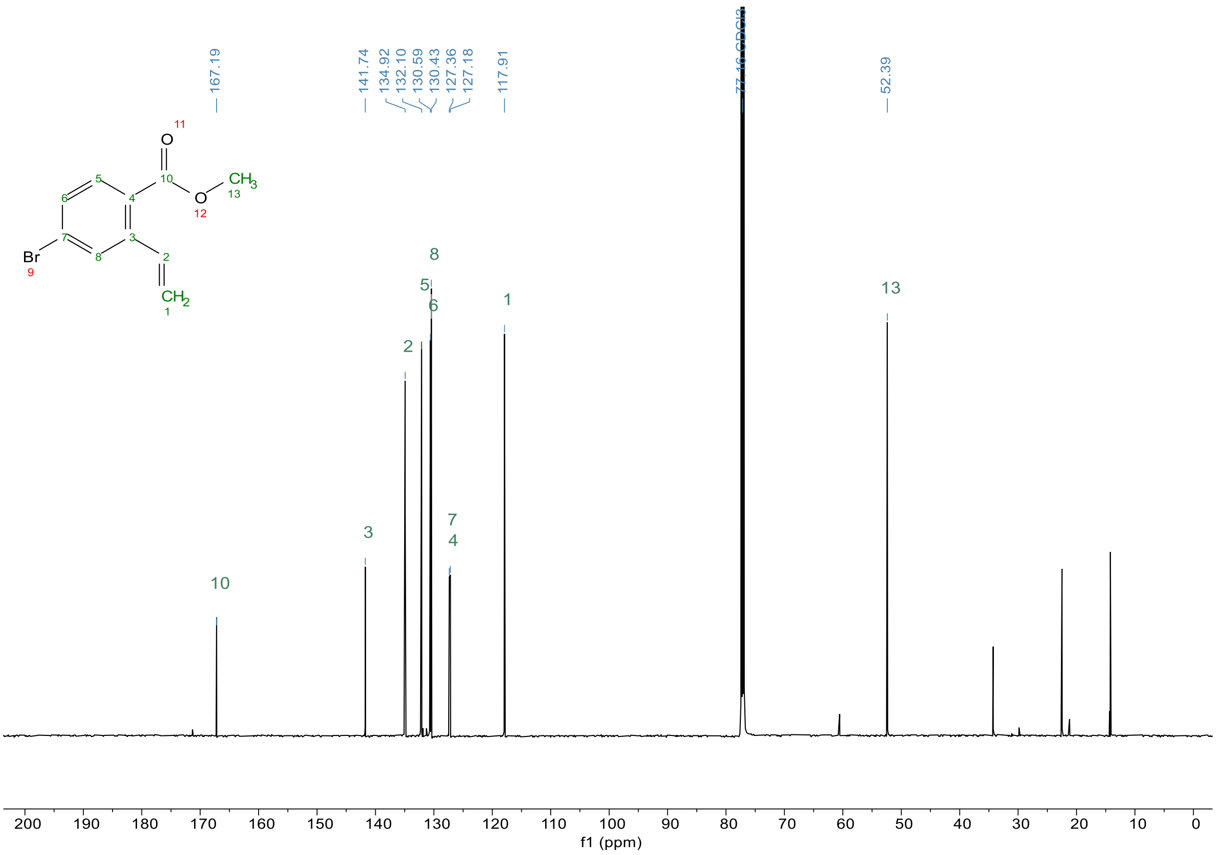

Fig.S65. ^{13}C NMR Spectrum of **S37** (Chloroform-d, 298 K).

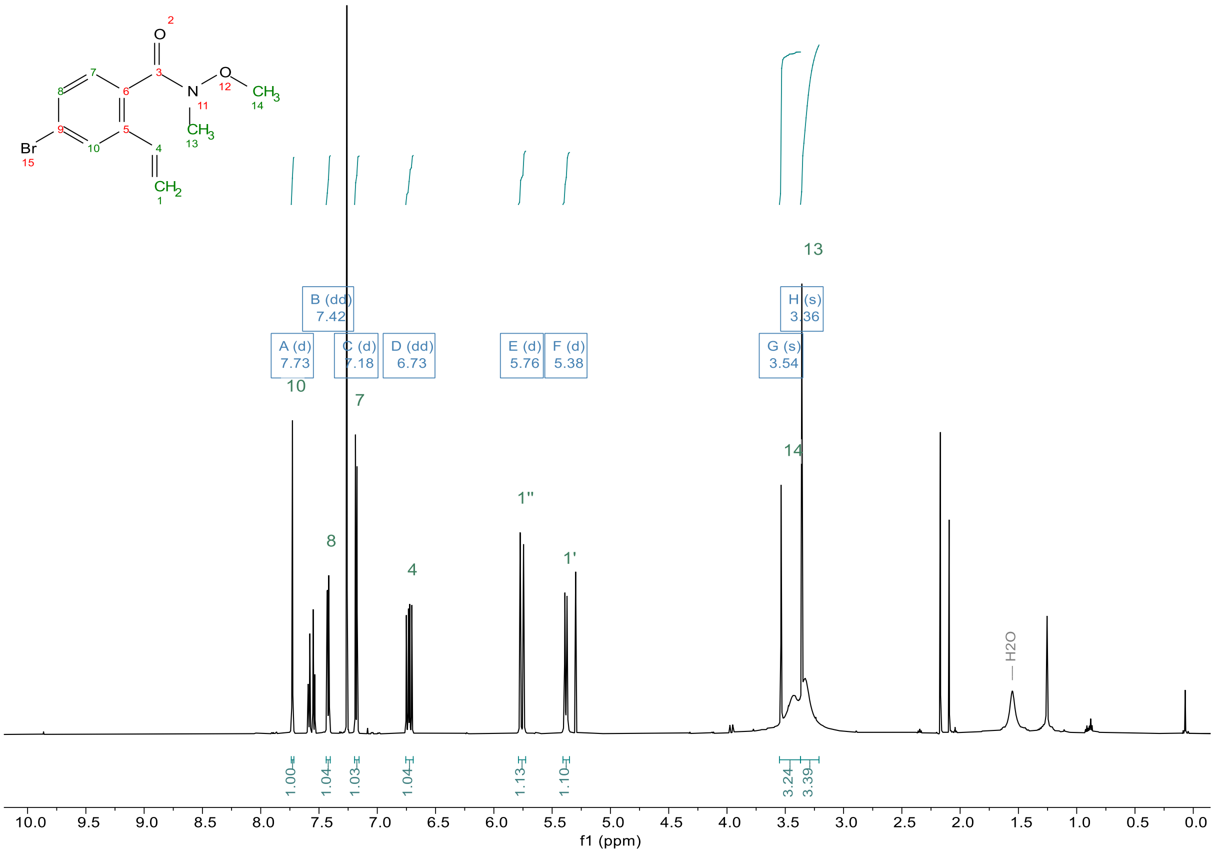

Fig.S66. ^{1}H NMR Spectrum of **S38** (Chloroform-d, 298 K).

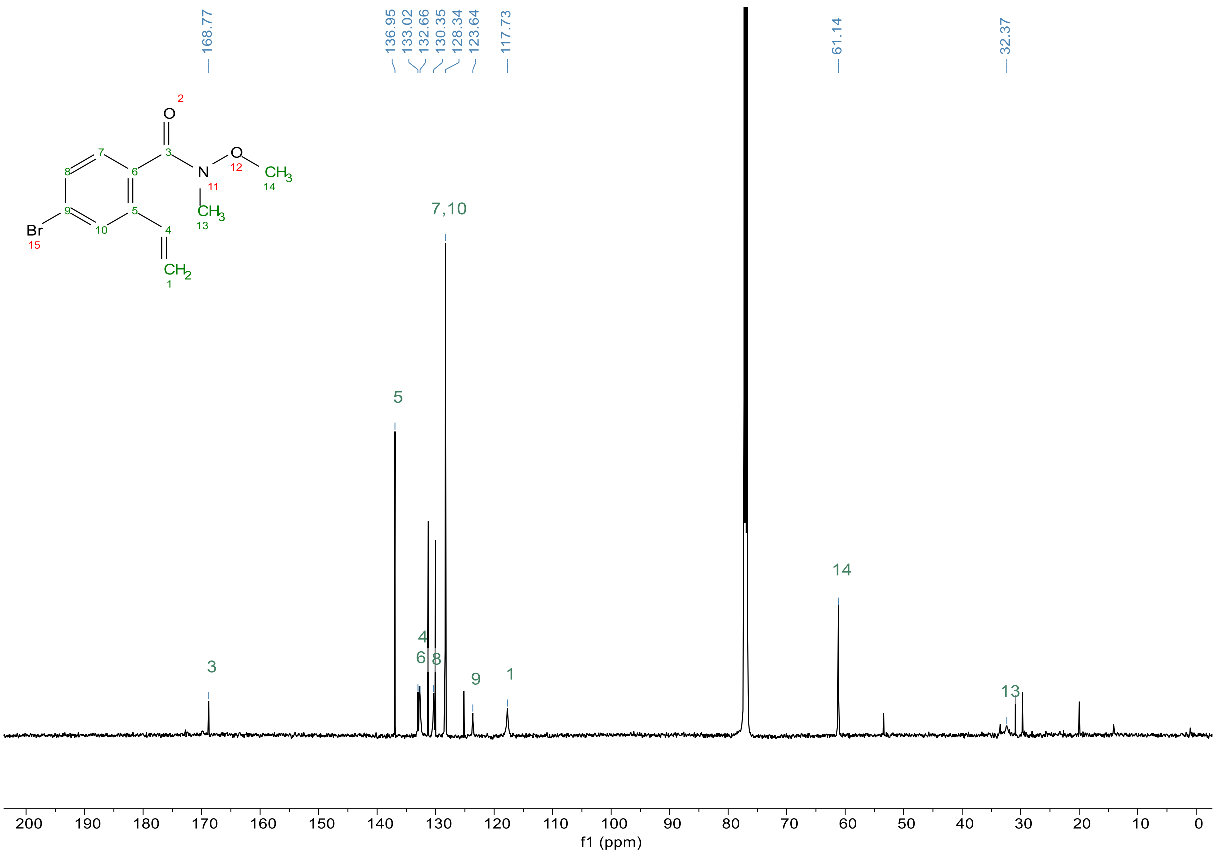

Fig.S67. ^{13}C NMR Spectrum of **S38** (Chloroform-d, 298 K).

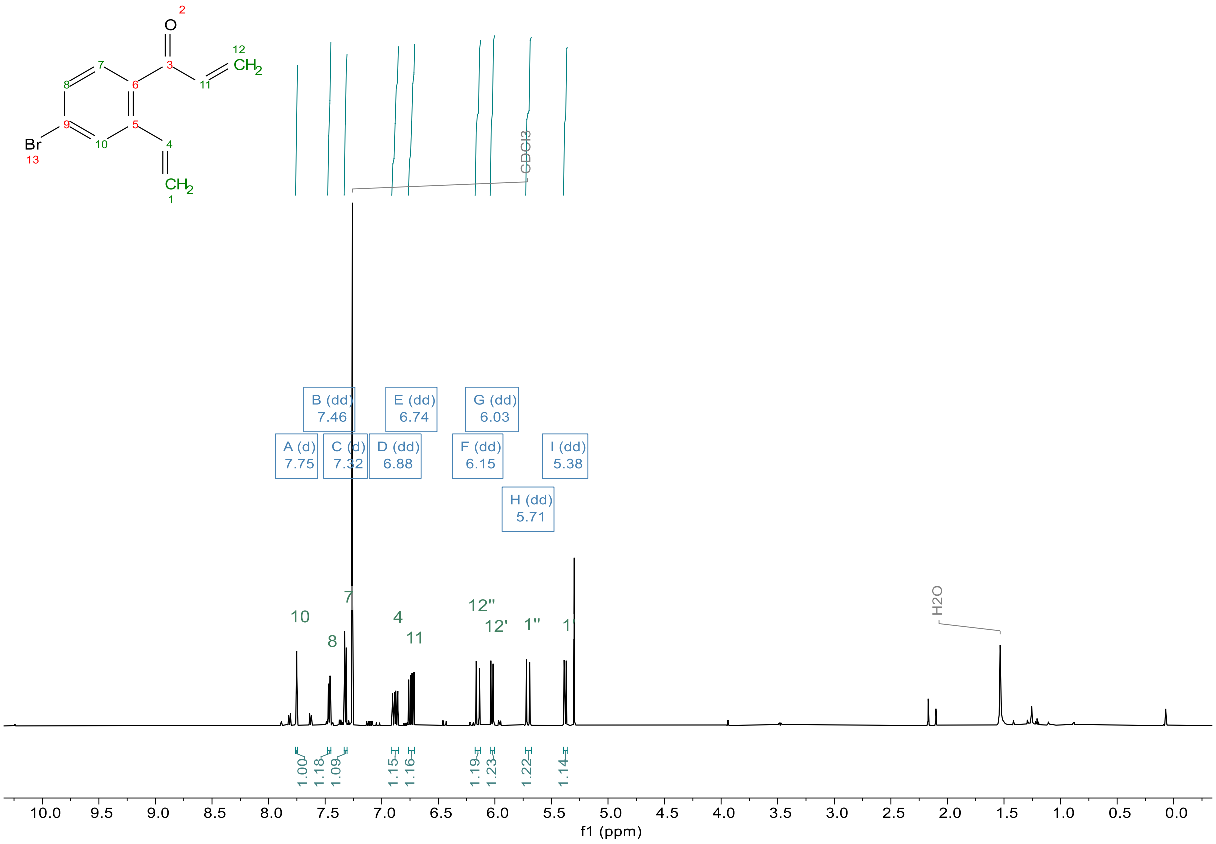

Fig.S68. ^{1}H NMR Spectrum of **S39** (Chloroform-d, 298 K).

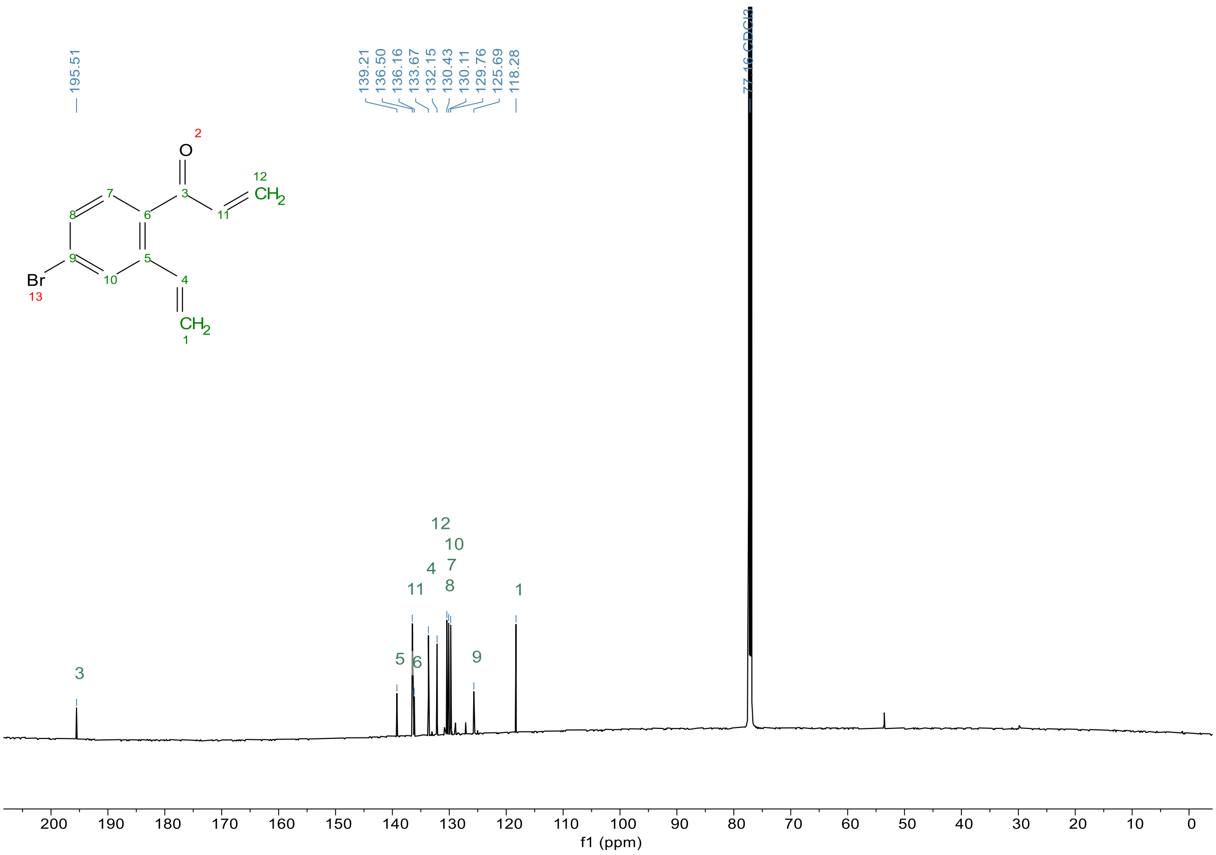

Fig.S69. ^{13}C NMR Spectrum of **S39** (Chloroform-d, 298 K).

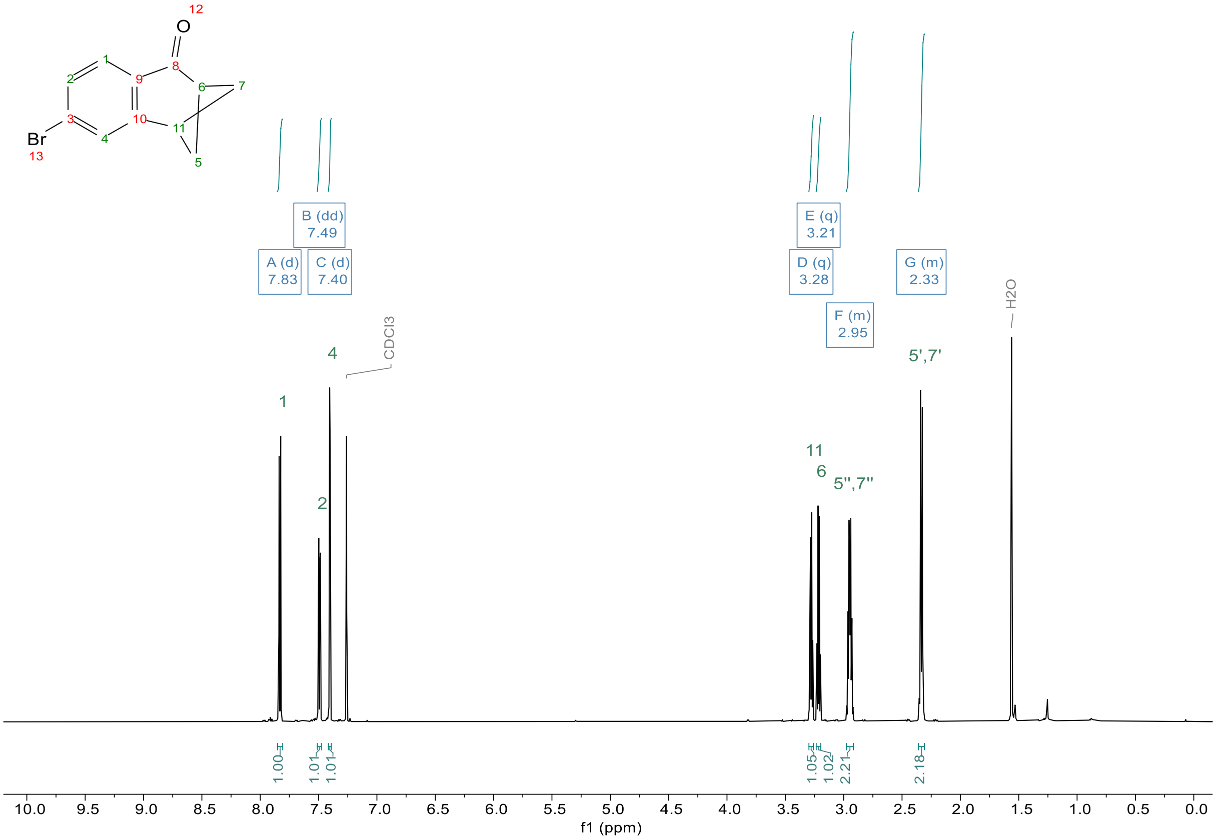

Fig.S70. ^{1}H NMR Spectrum of **25** (Chloroform-d, 298 K).

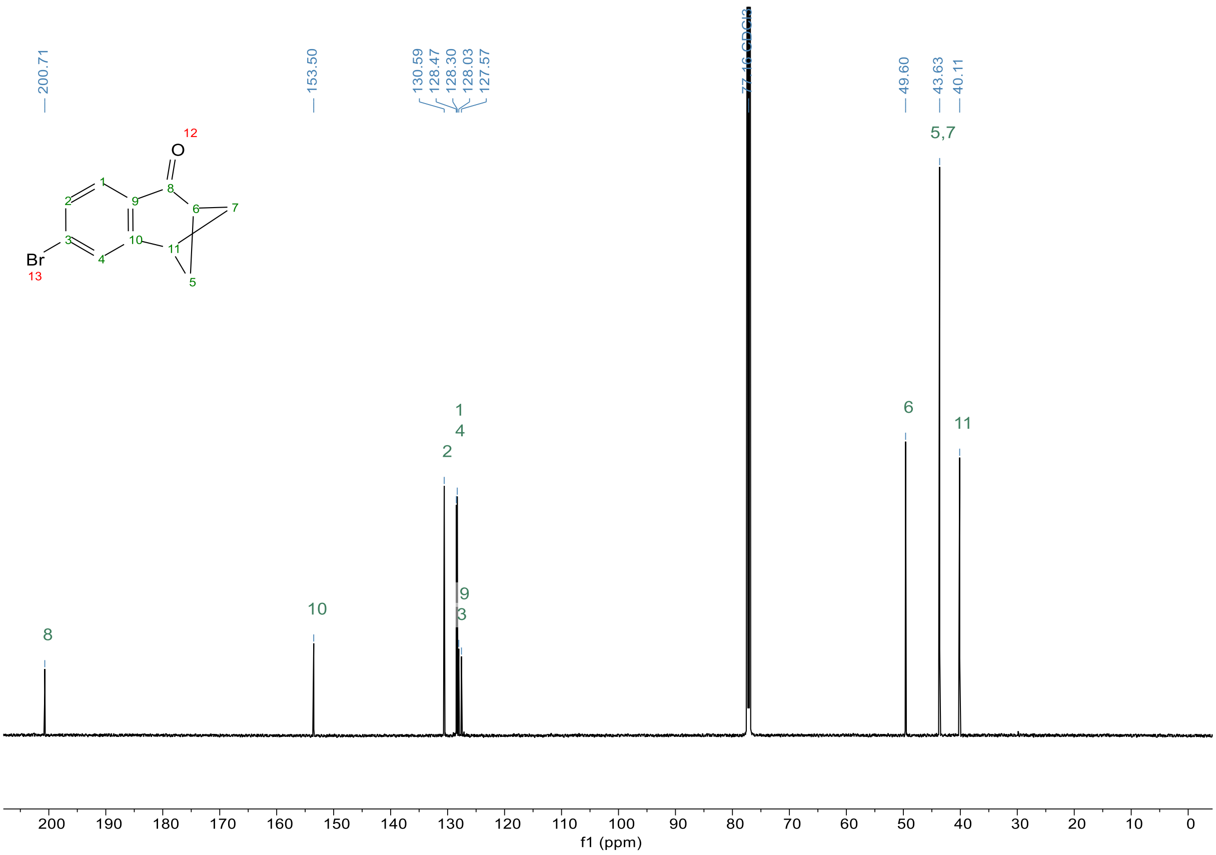

Fig.S71. ^{13}C NMR Spectrum of **25** (Chloroform-d, 298 K).

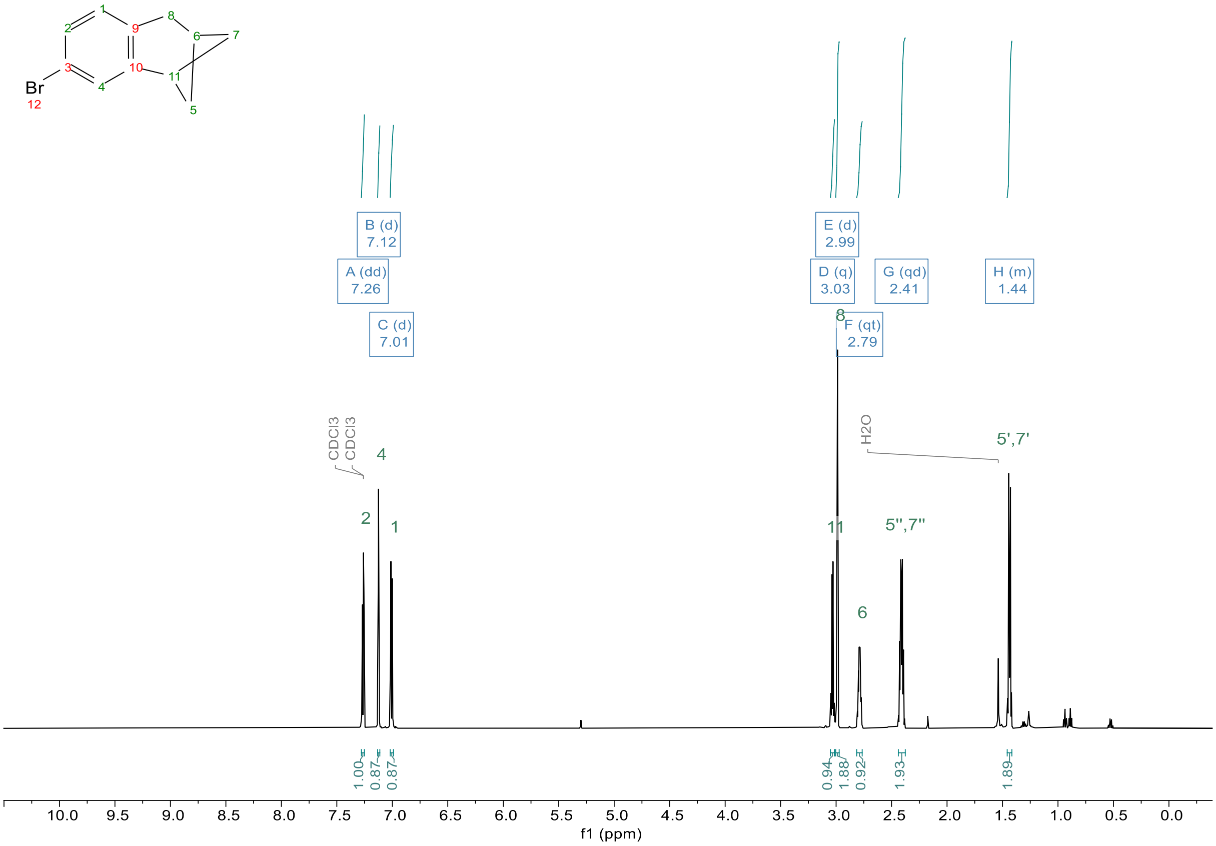

Fig.S72. ^{1}H NMR Spectrum of **29b** (Chloroform-d, 298 K).

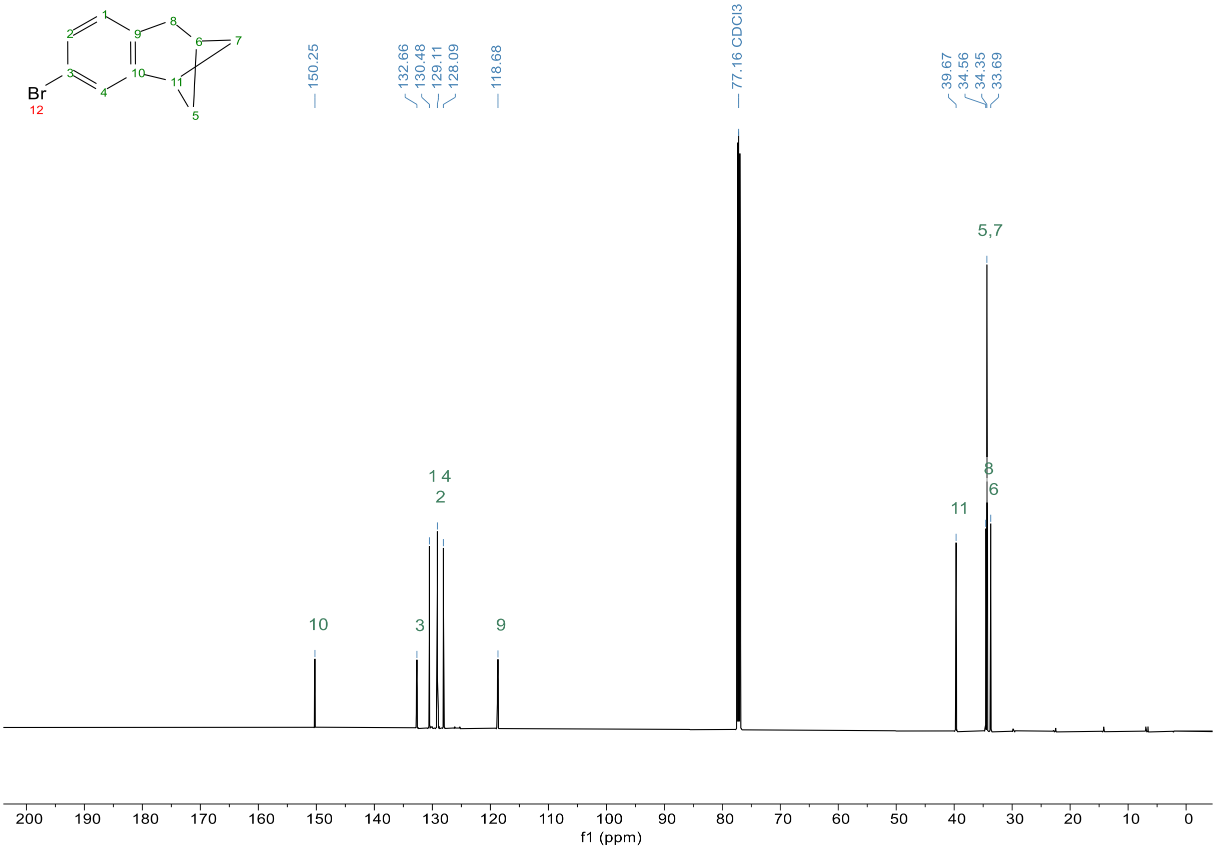

Fig.S73. ^{13}C NMR Spectrum of **29b** (Chloroform-d, 298 K).

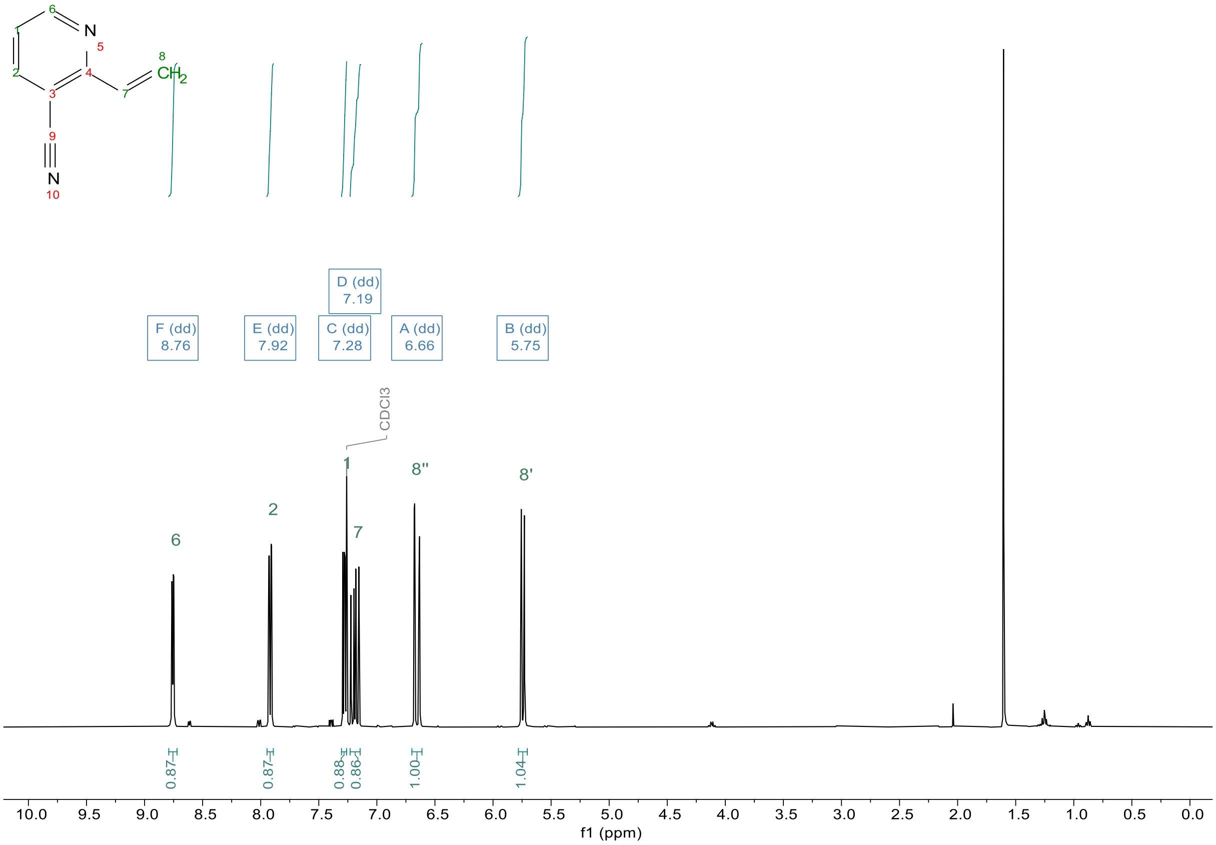

Fig.S74. ^{1}H NMR Spectrum of **S41** (Chloroform-d, 298 K).

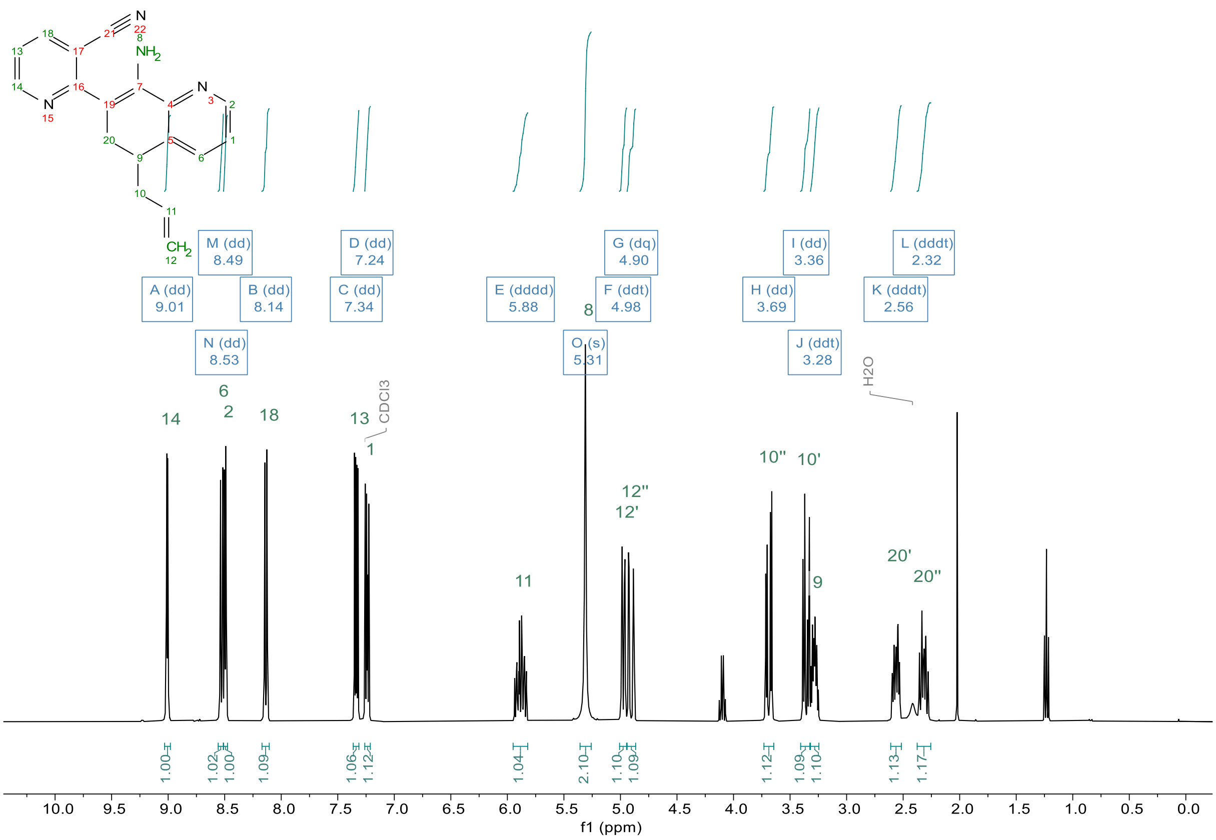

Fig.S75. ^{1}H NMR Spectrum of **S43** (Chloroform-d, 298 K).

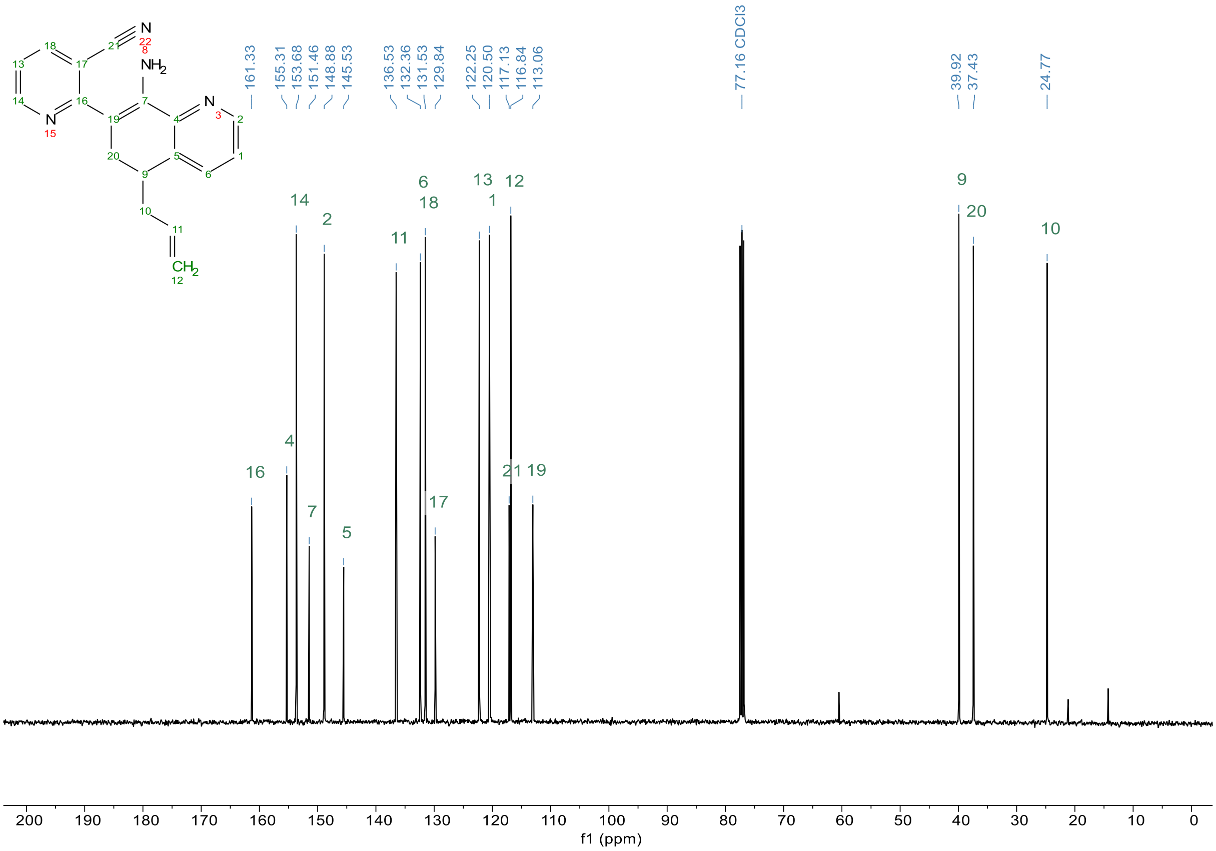

Fig.S76. ^{1}H NMR Spectrum of **S43** (Chloroform-d, 298 K).

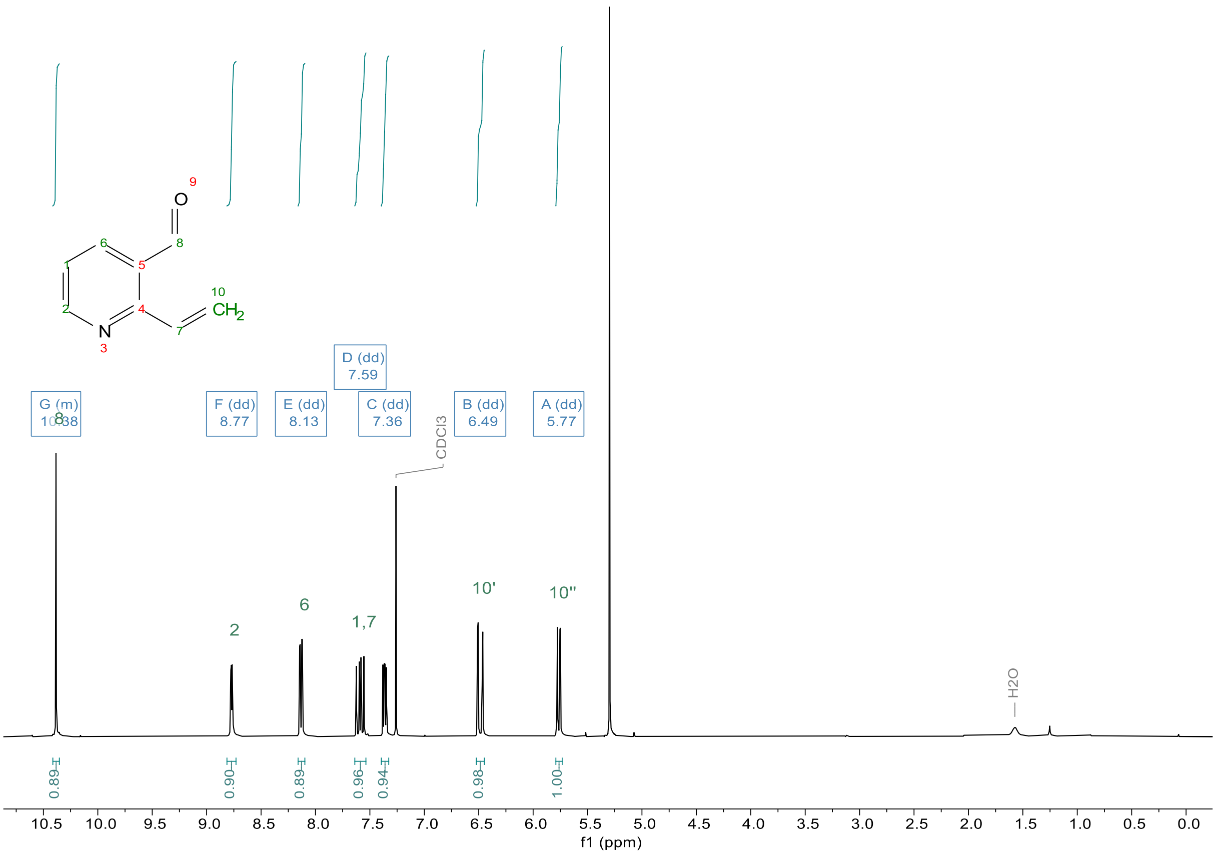

Fig.S77. ^{1}H NMR Spectrum of **S45** (Chloroform-d, 298 K).

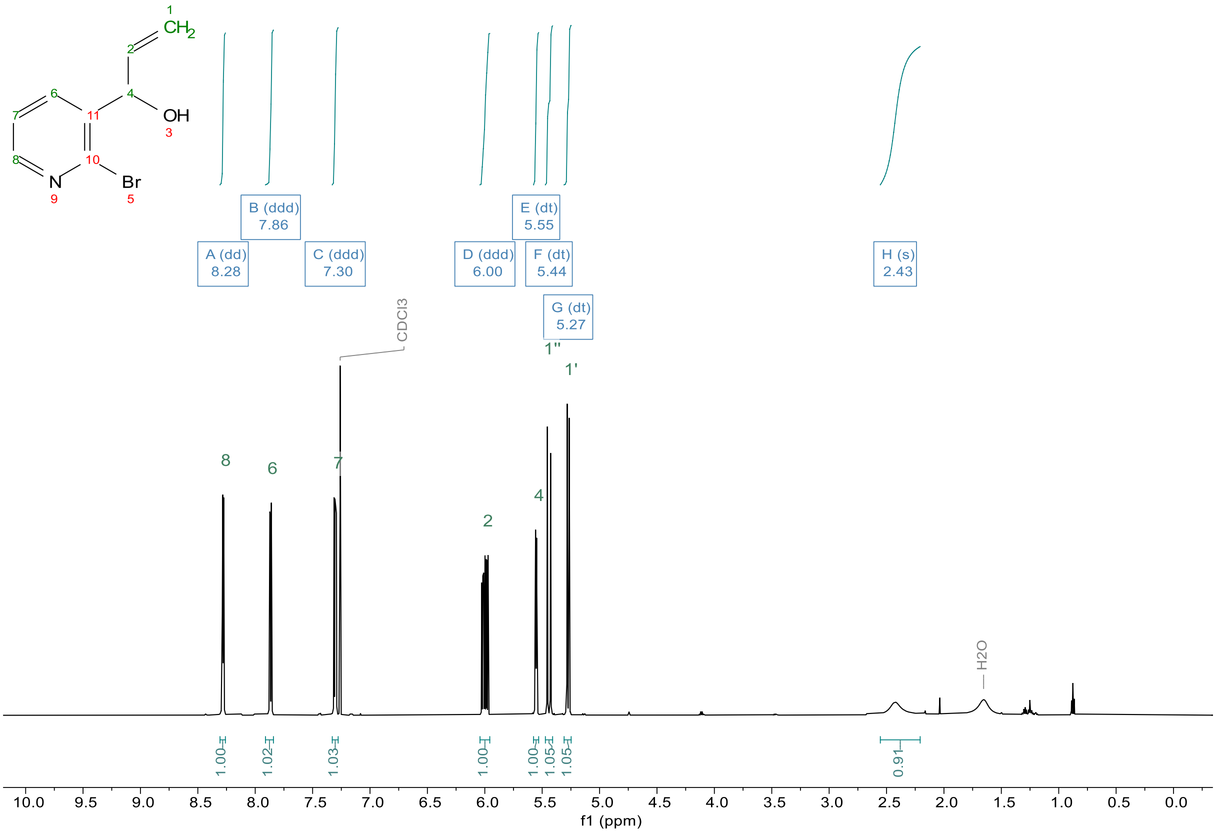

Fig.S78. ^{1}H NMR Spectrum of **S47** (Chloroform-d, 298 K).

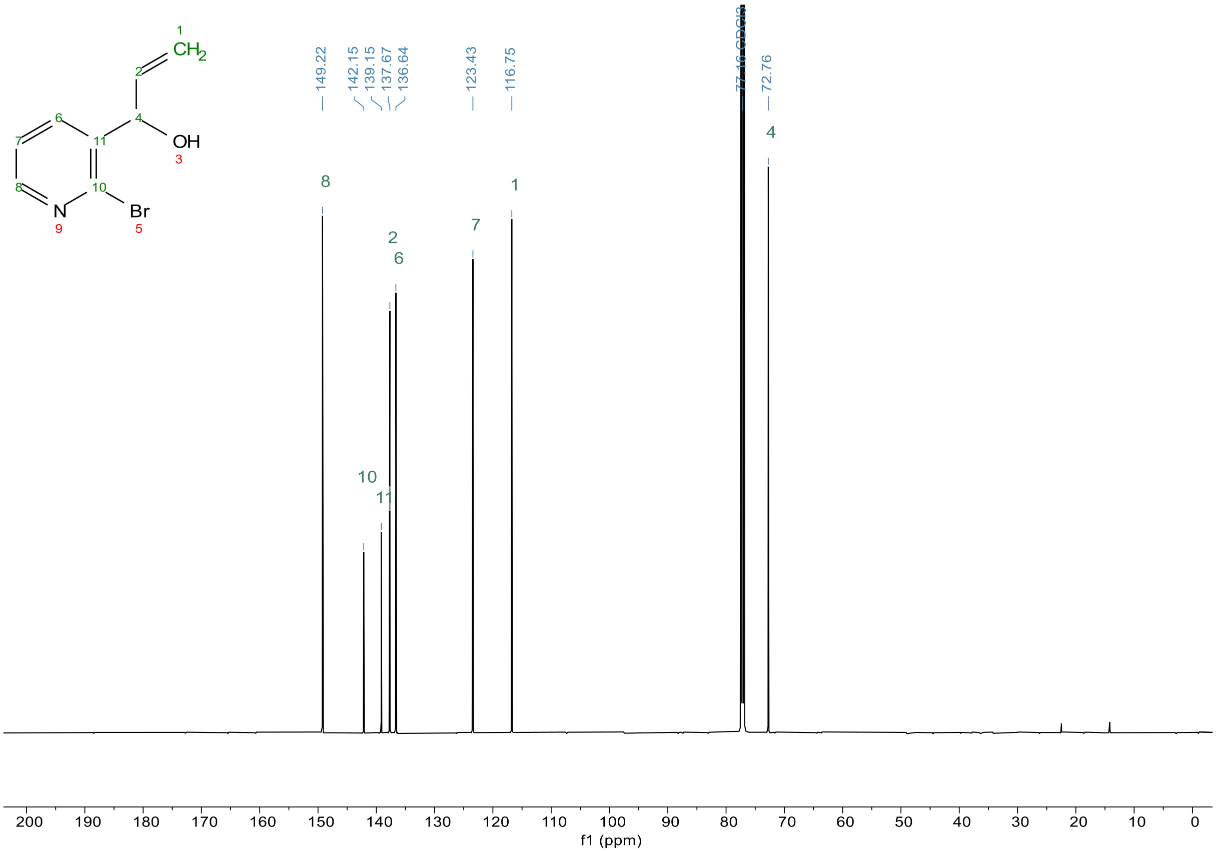

Fig.S79. ^{13}C NMR Spectrum of **S47** (Chloroform-d, 298 K).

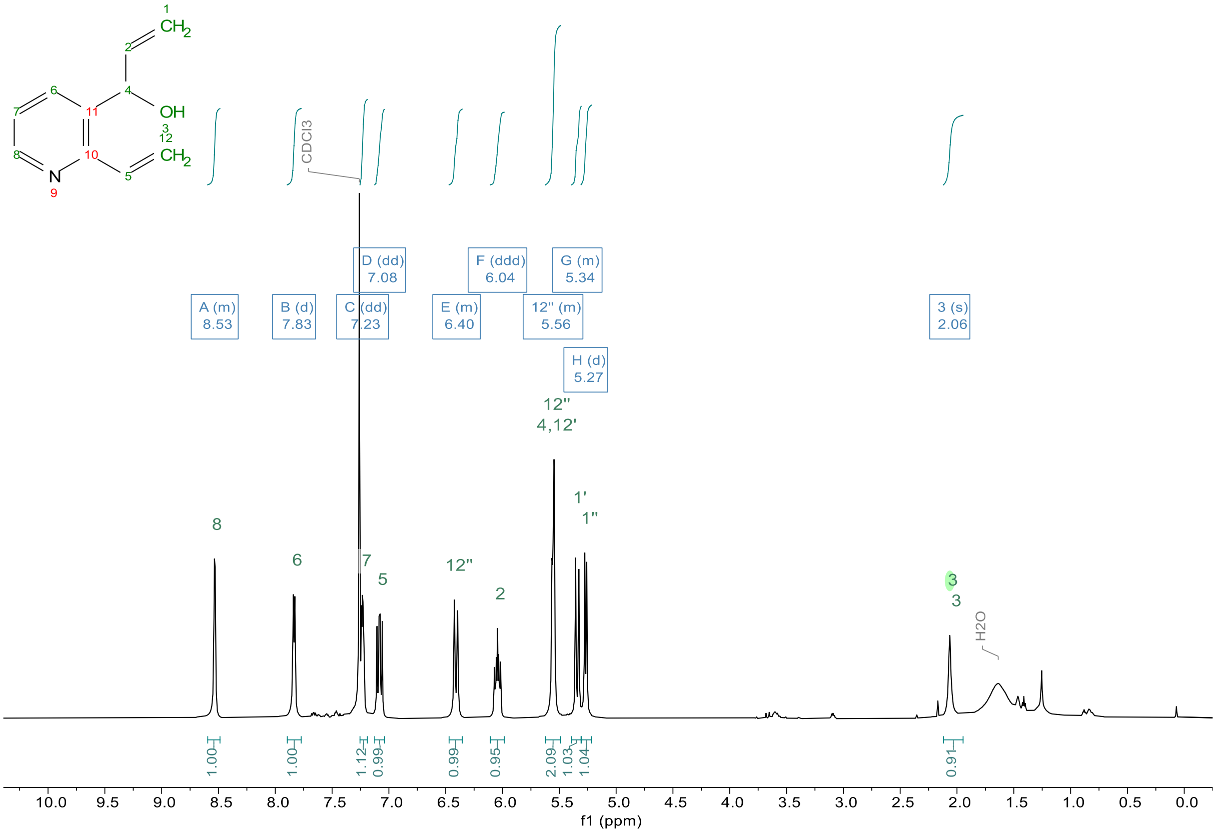

Fig.S80. ^{1}H NMR Spectrum of **S46** (Chloroform-d, 298 K).

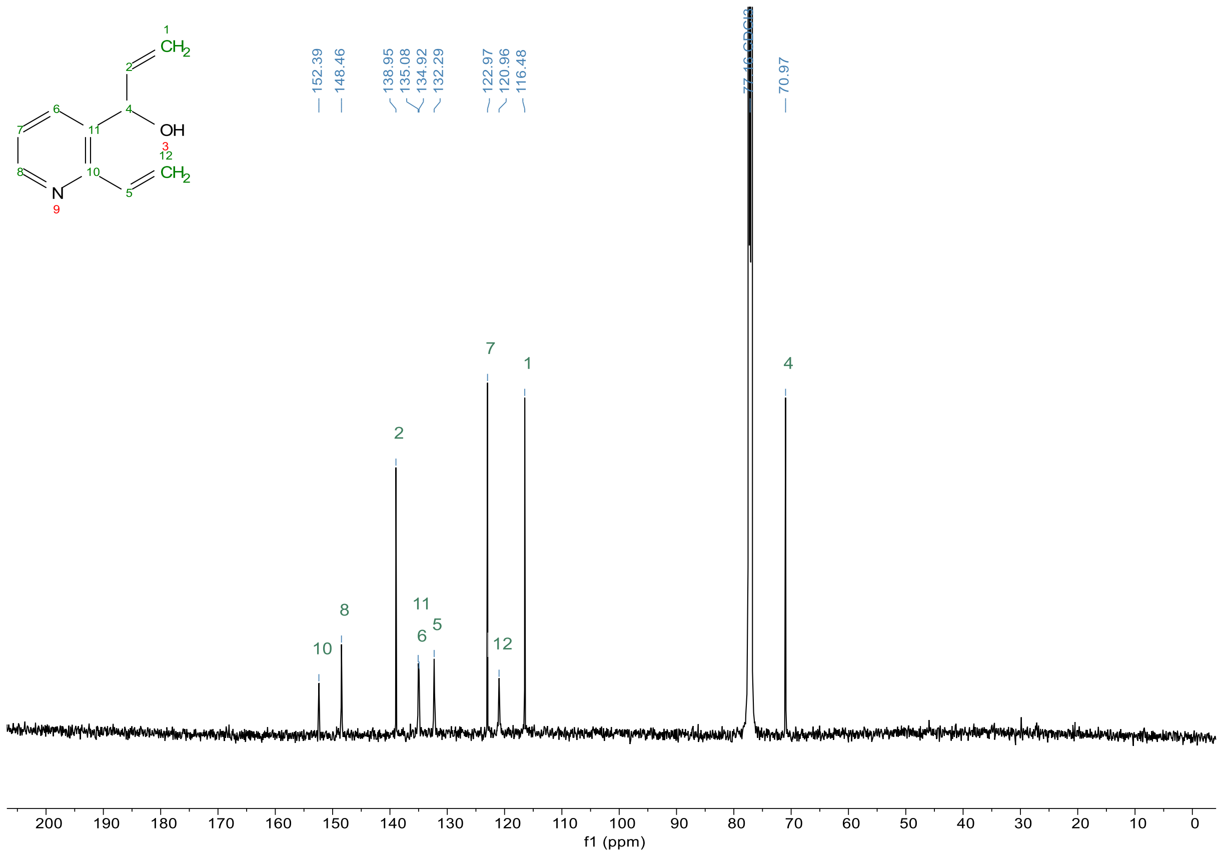

Fig.S81. ^{1}H NMR Spectrum of **S46** (Chloroform-d, 298 K).

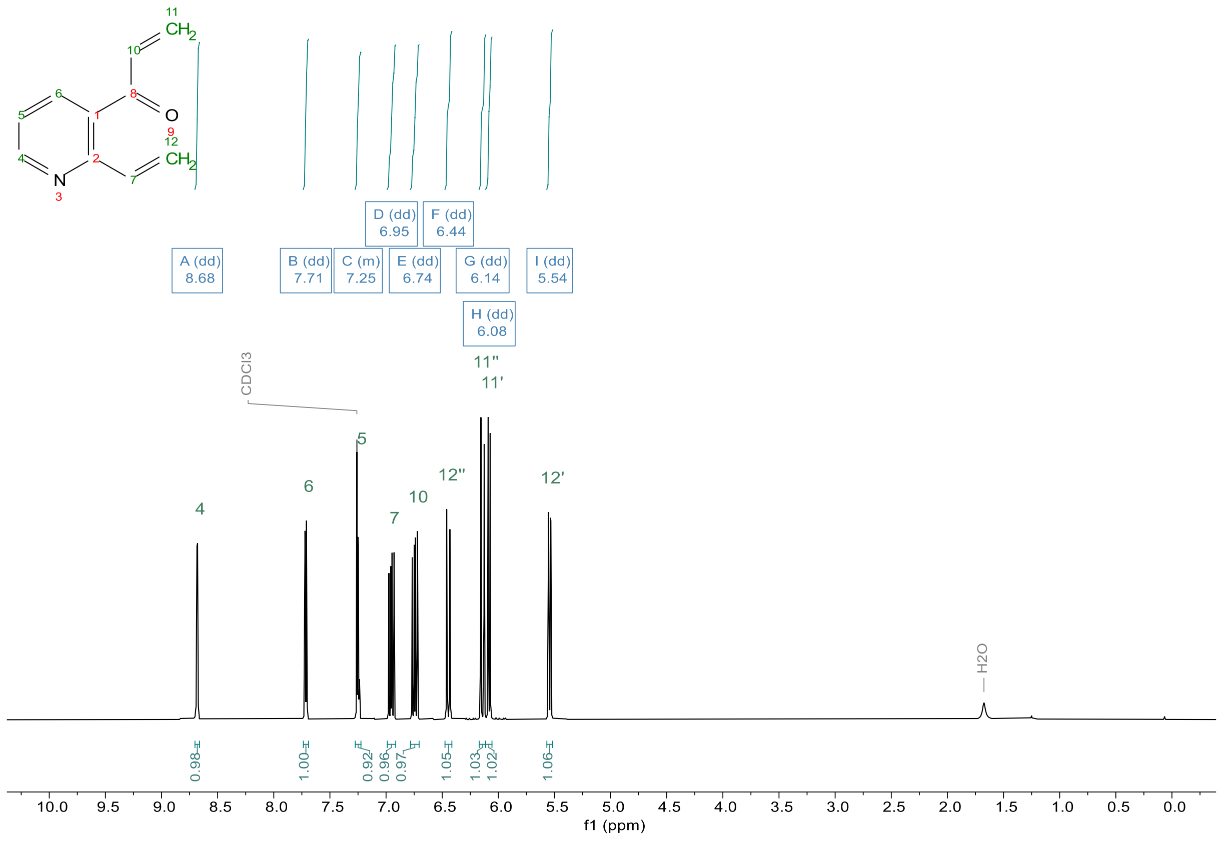

Fig.S82. ^{1}H NMR Spectrum of **S48** (Chloroform-d, 298 K).

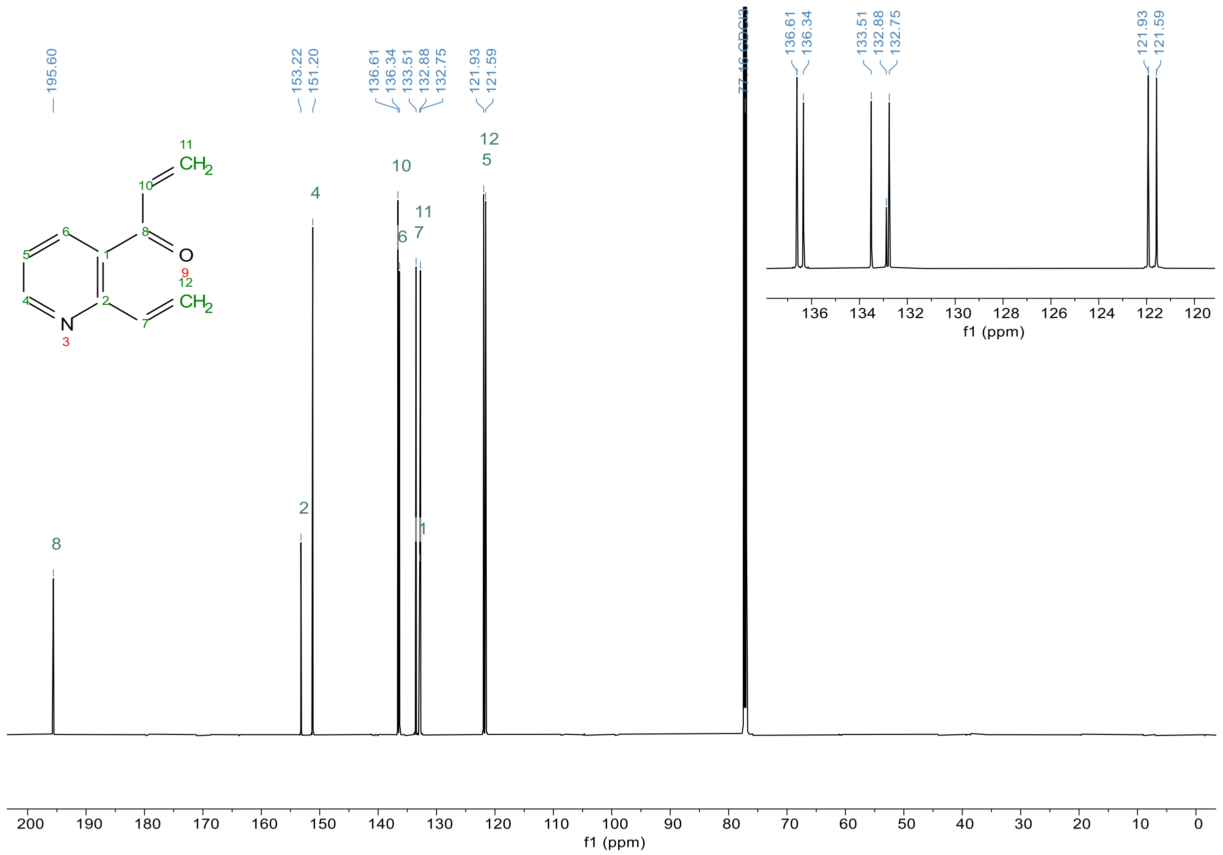

Fig.S83. ^{13}C NMR Spectrum of **S48** (Chloroform-d, 298 K).

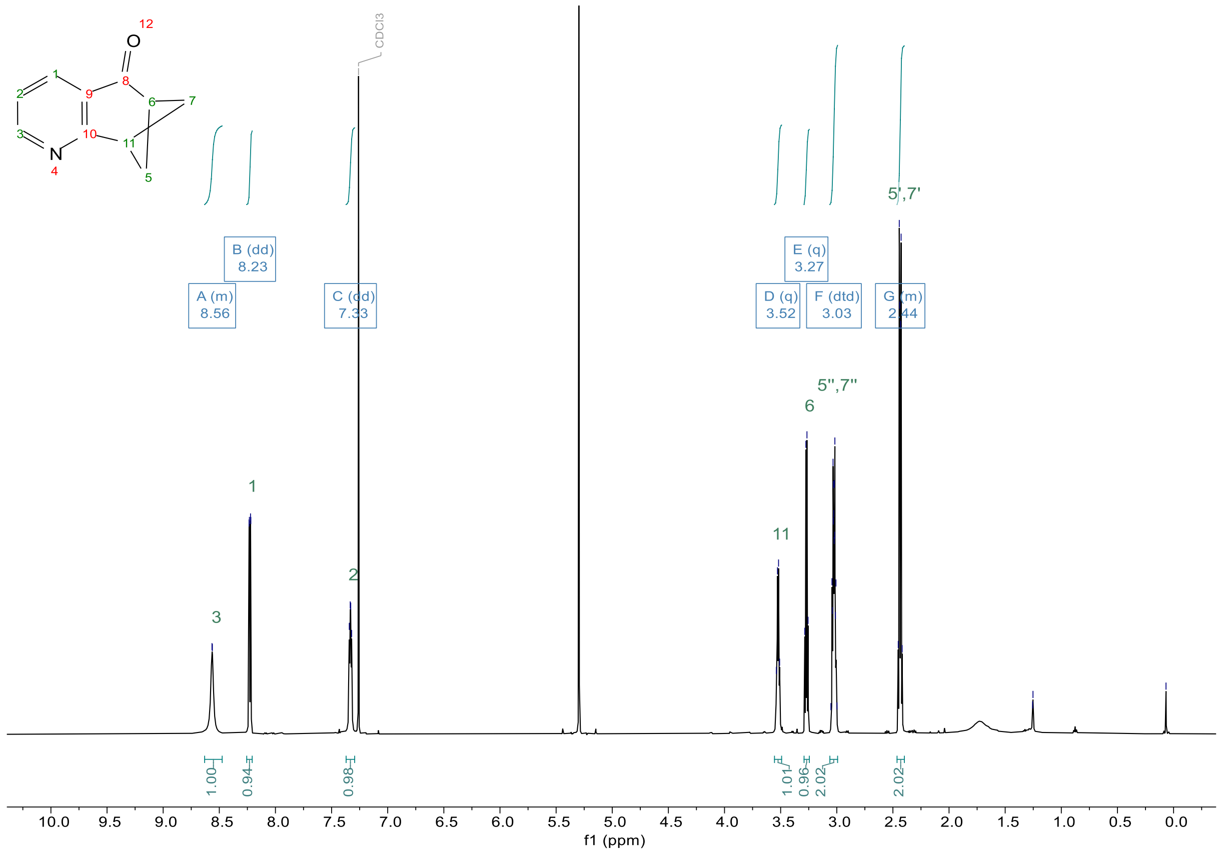

Fig.S84. ^{1}H NMR Spectrum of **S49** (Chloroform-d, 298 K).

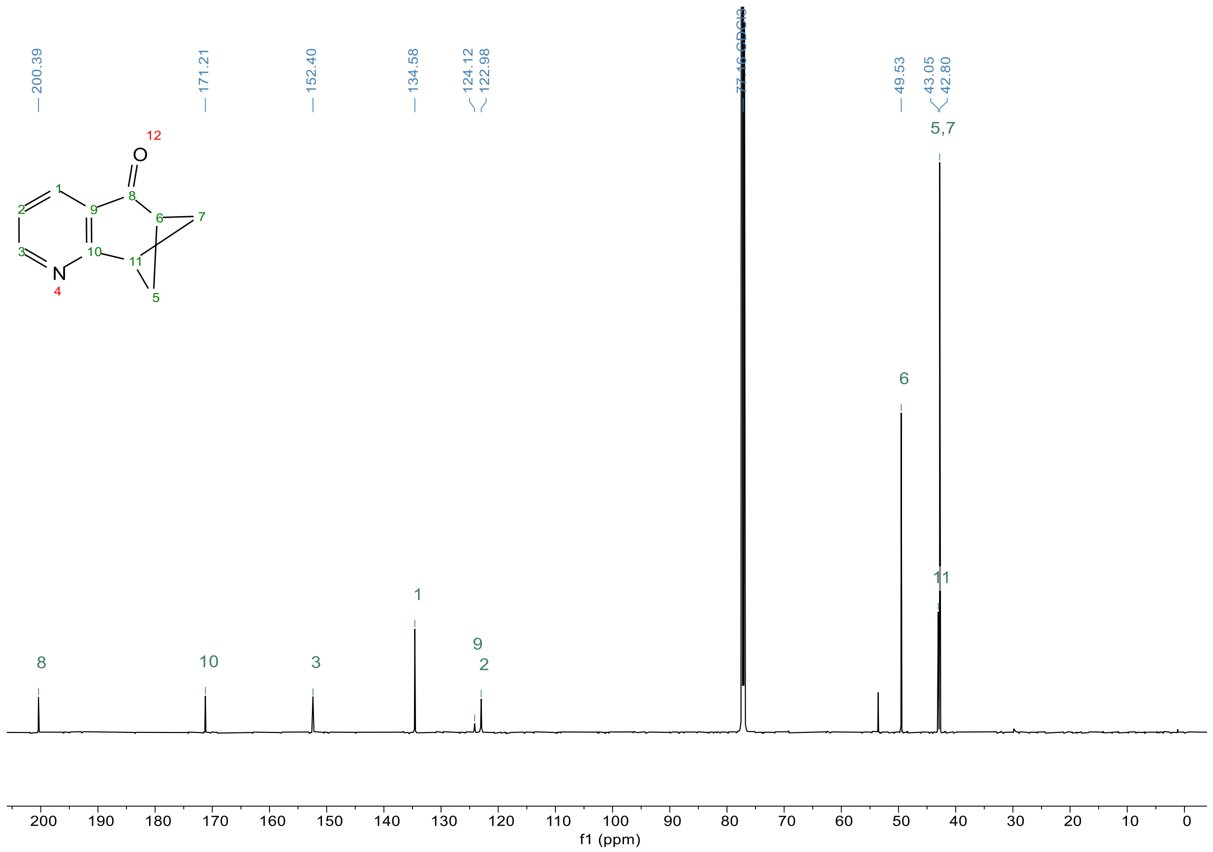

Fig.S85. ^{13}C NMR Spectrum of **S49** (Chloroform-d, 298 K).

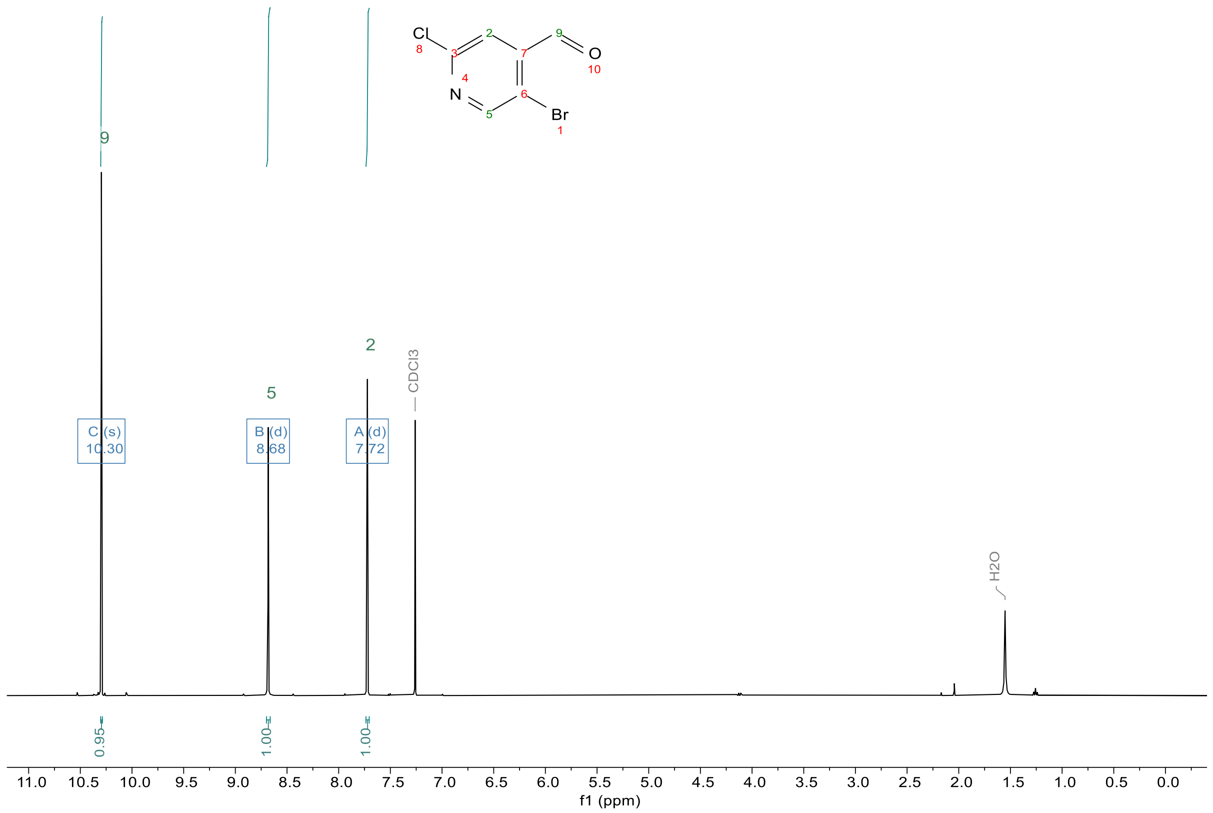

Fig.S86. ^{1}H NMR Spectrum of **S52** (Chloroform-d, 298 K).

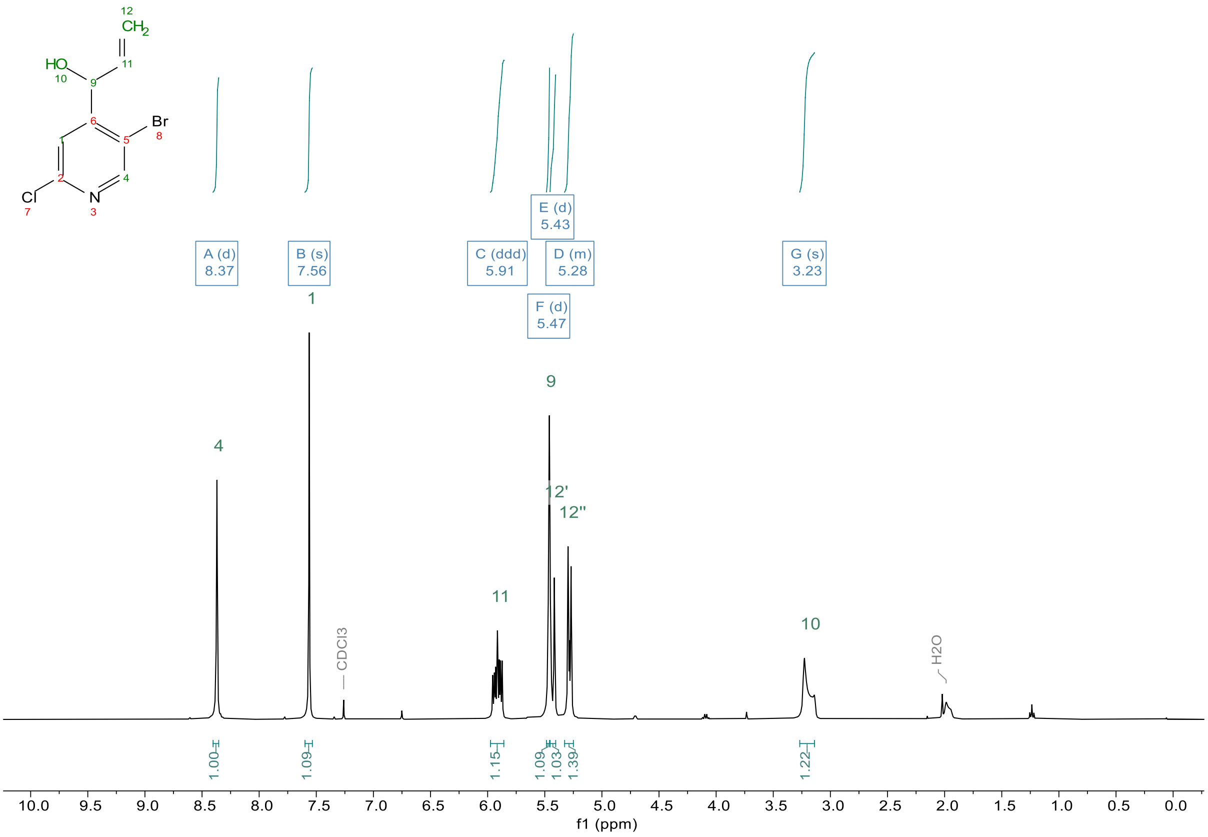

Fig.S87. ^{1}H NMR Spectrum of **S53** (Chloroform-d, 298 K).

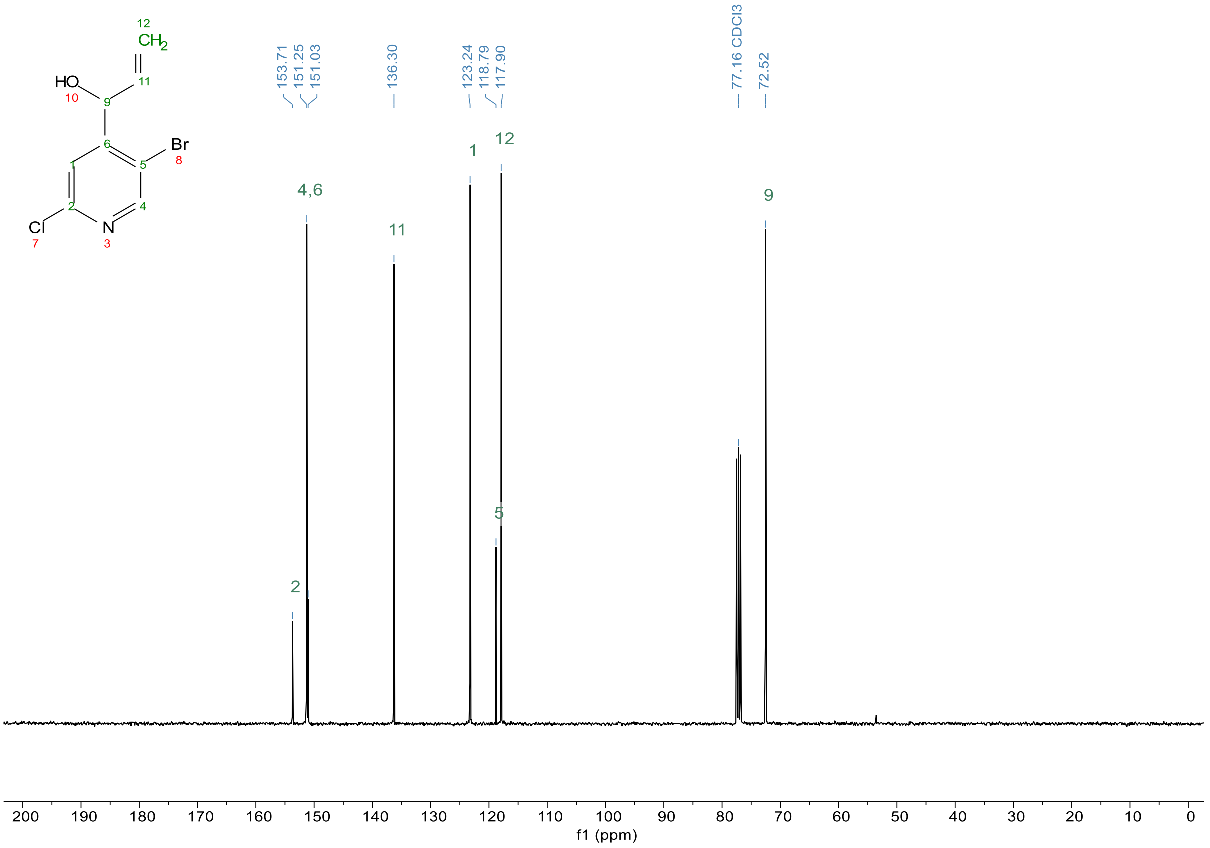

Fig.S88. ^{13}C NMR Spectrum of **S53** (Chloroform-d, 298 K).

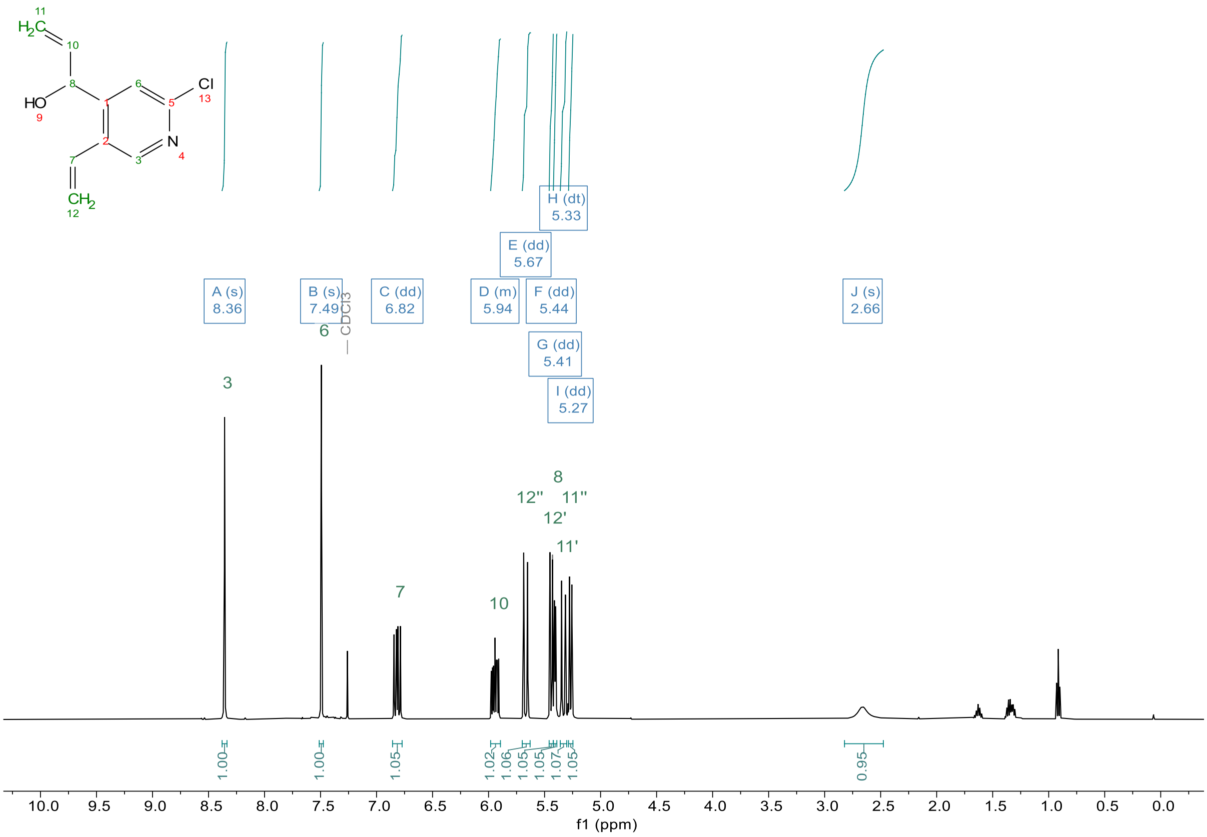

Fig.S89. ^{1}H NMR Spectrum of **S54** (Chloroform-d, 298 K).

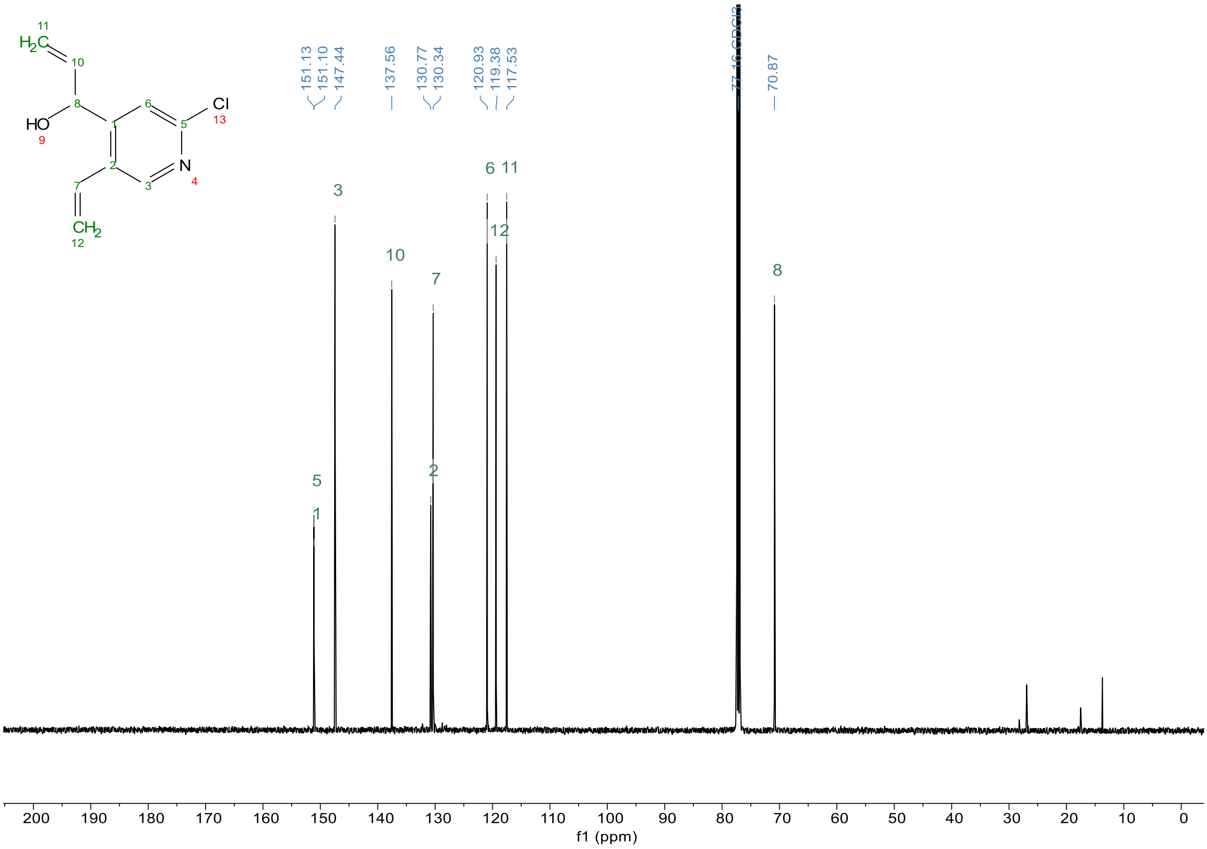

Fig.S90. ^{13}C NMR Spectrum of **S54** (Chloroform-d, 298 K).

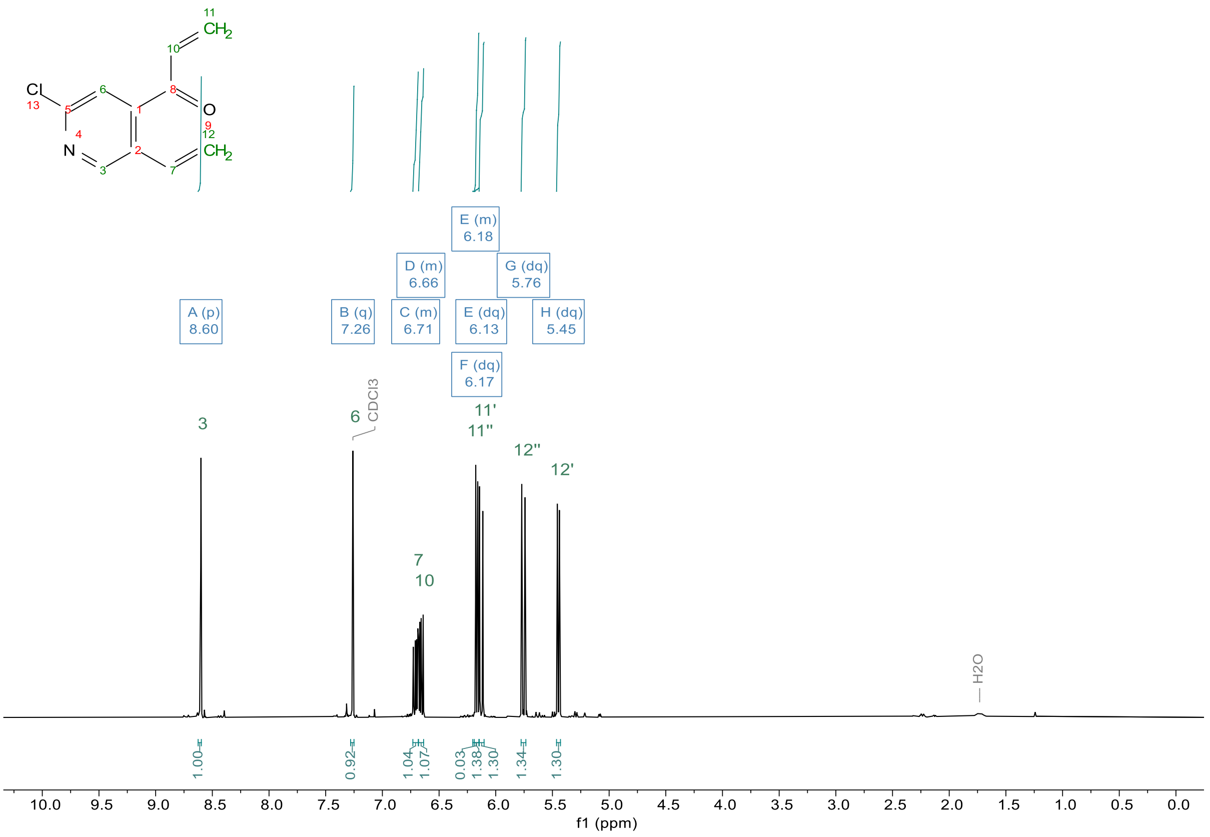

Fig.S91. ^{1}H NMR Spectrum of **S55** (Chloroform-d, 298 K).

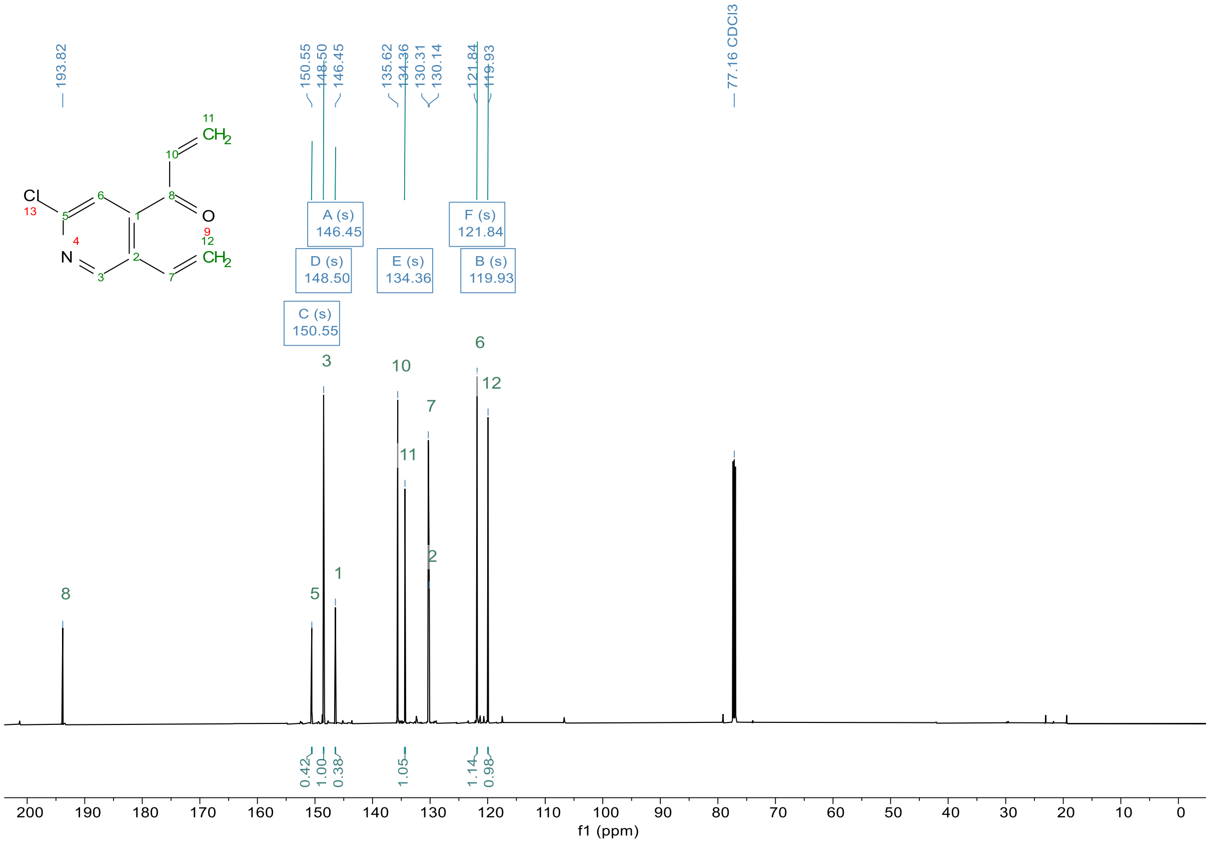

Fig.S92. ^{13}C NMR Spectrum of **S55** (Chloroform-d, 298 K).

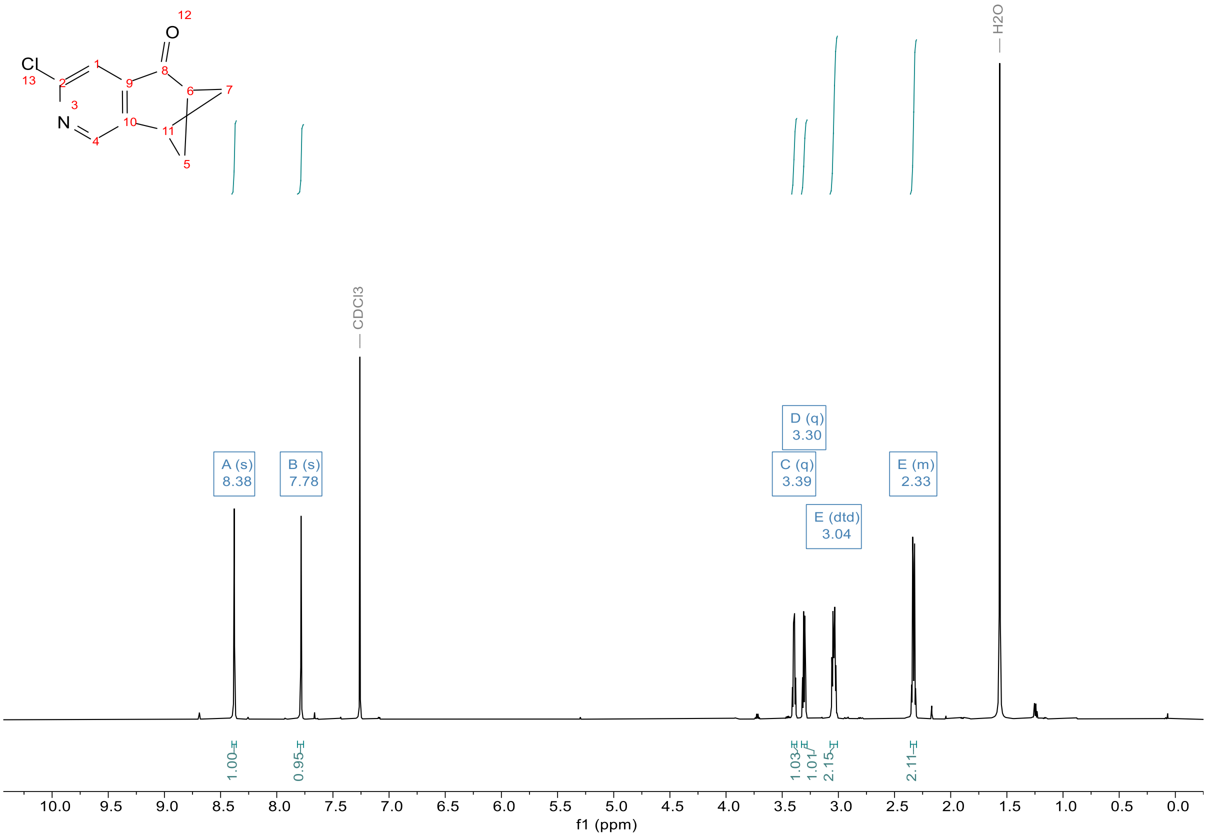

Fig.S93. ^{1}H NMR Spectrum of **26** (Chloroform-d, 298 K).

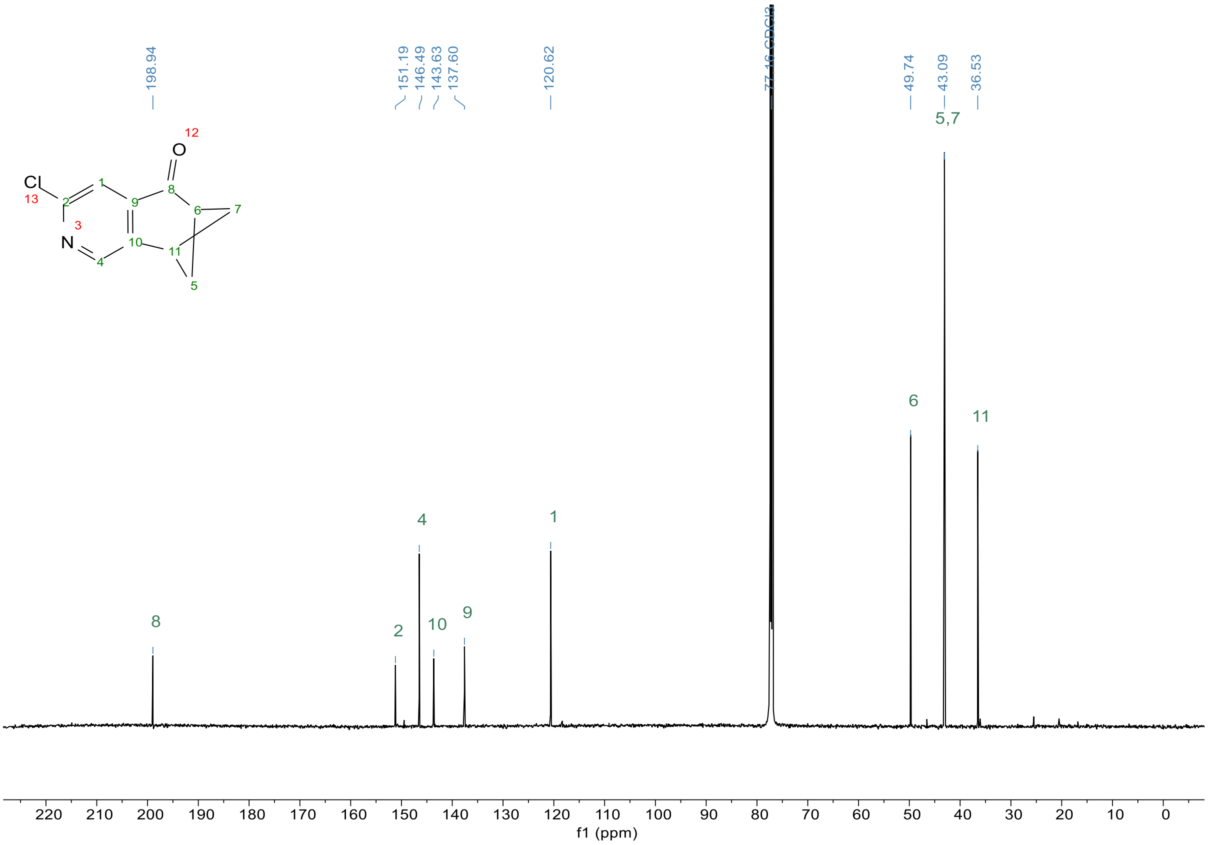

Fig.S94. ^{13}C NMR Spectrum of **26** (Chloroform-d, 298 K).

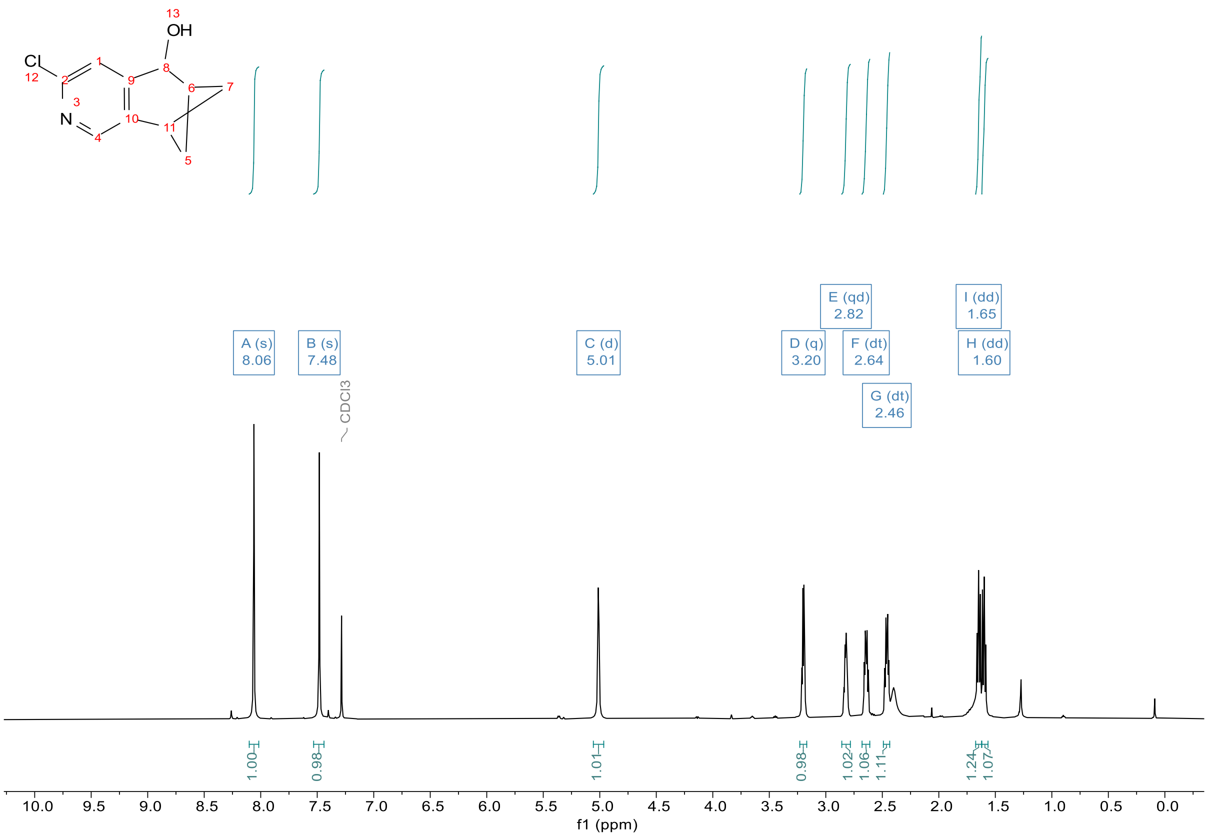

Fig.S95. ^{1}H NMR Spectrum of **S56** (Chloroform-d, 298 K).

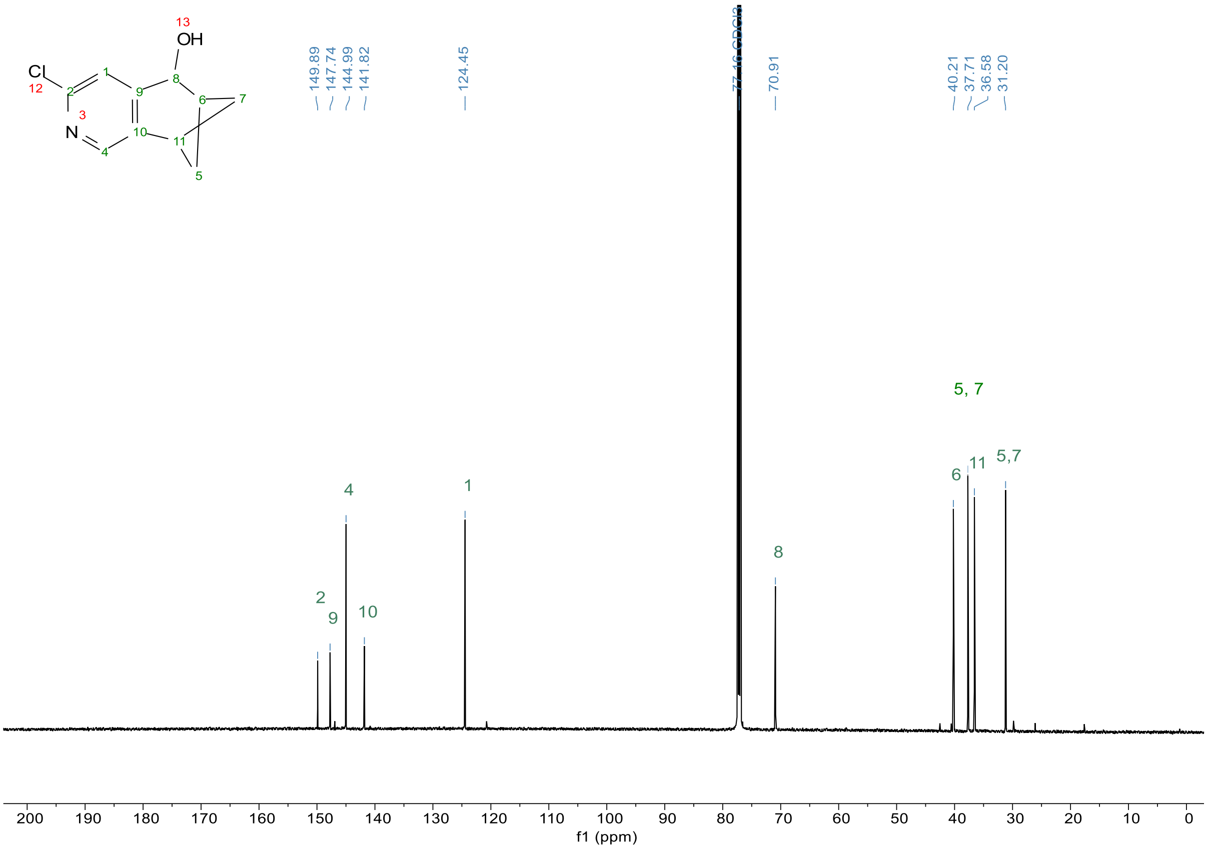

Fig.S96. ^{13}C NMR Spectrum of **S56** (Chloroform-d, 298 K).

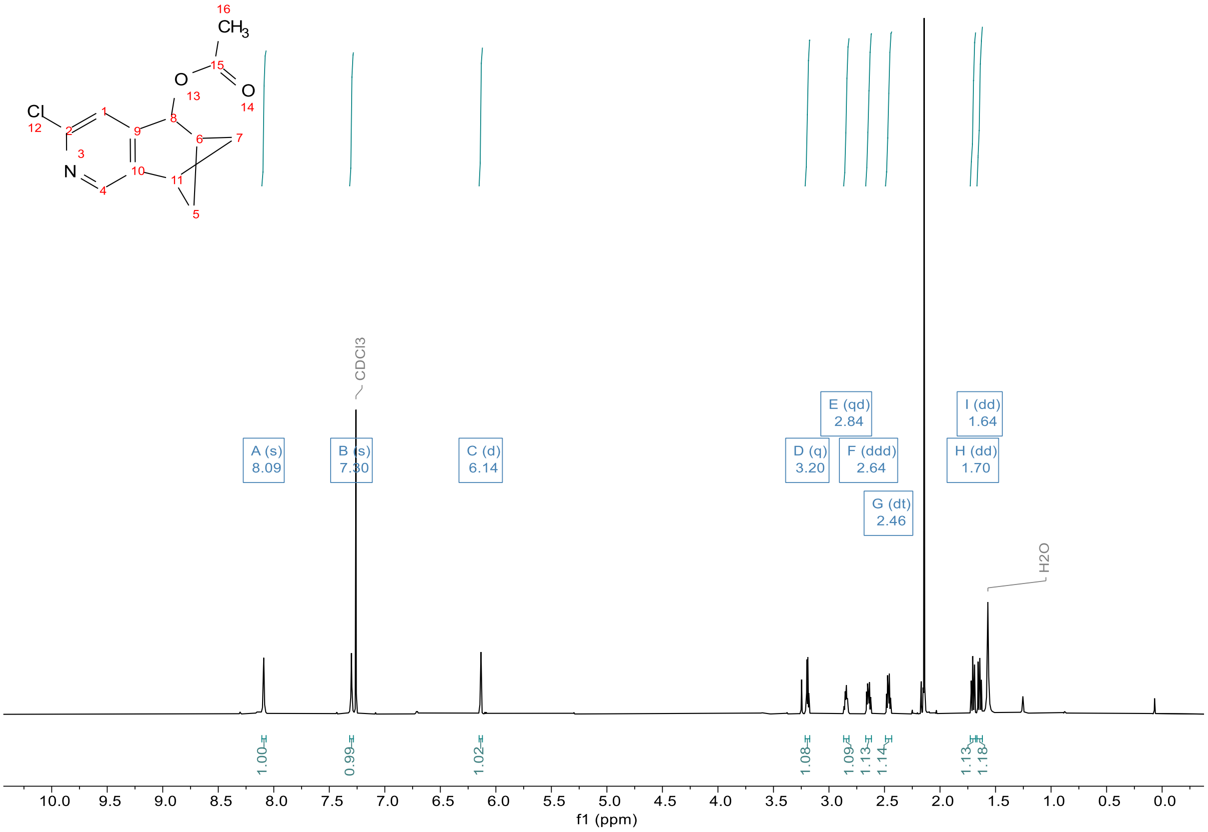

Fig.S97. ^{1}H NMR Spectrum of **S57** (Chloroform-d, 298 K).

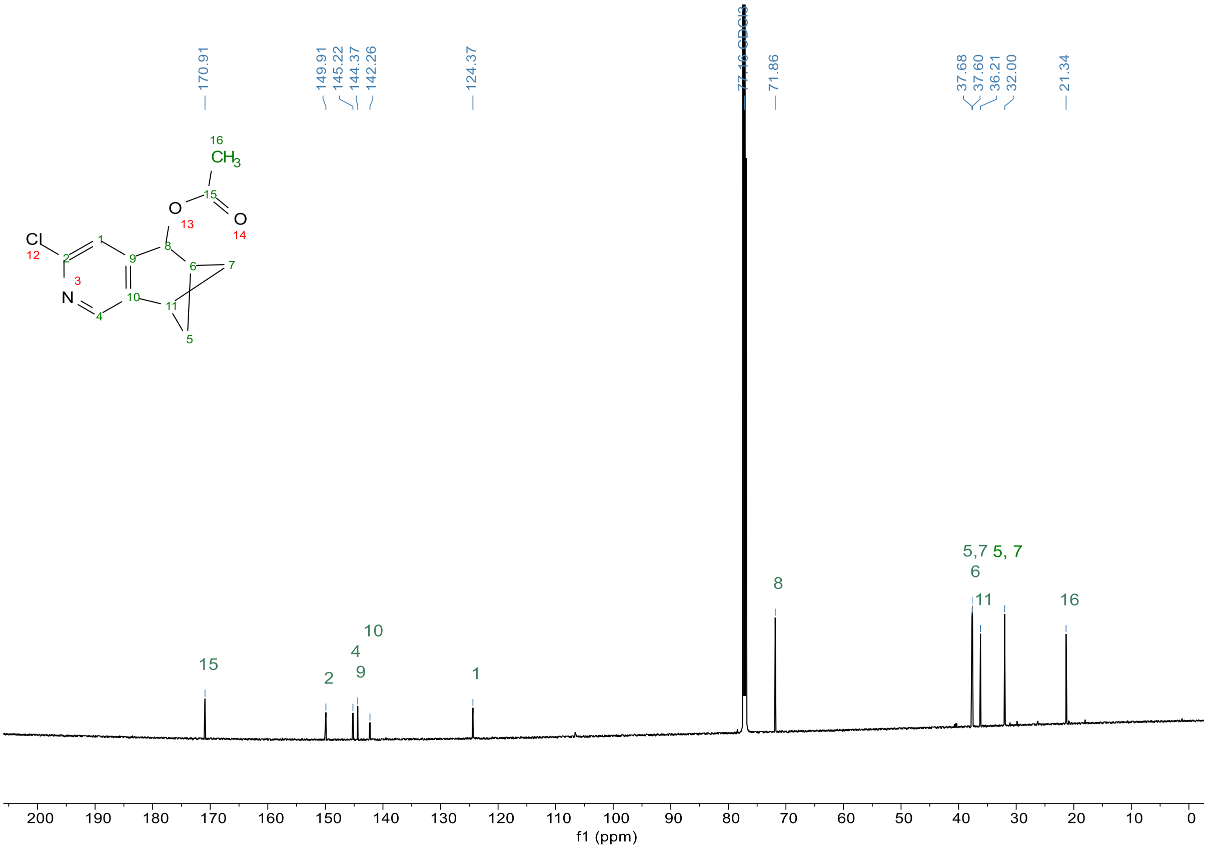

Fig.S98. ^{13}C NMR Spectrum of **S57** (Chloroform-d, 298 K).

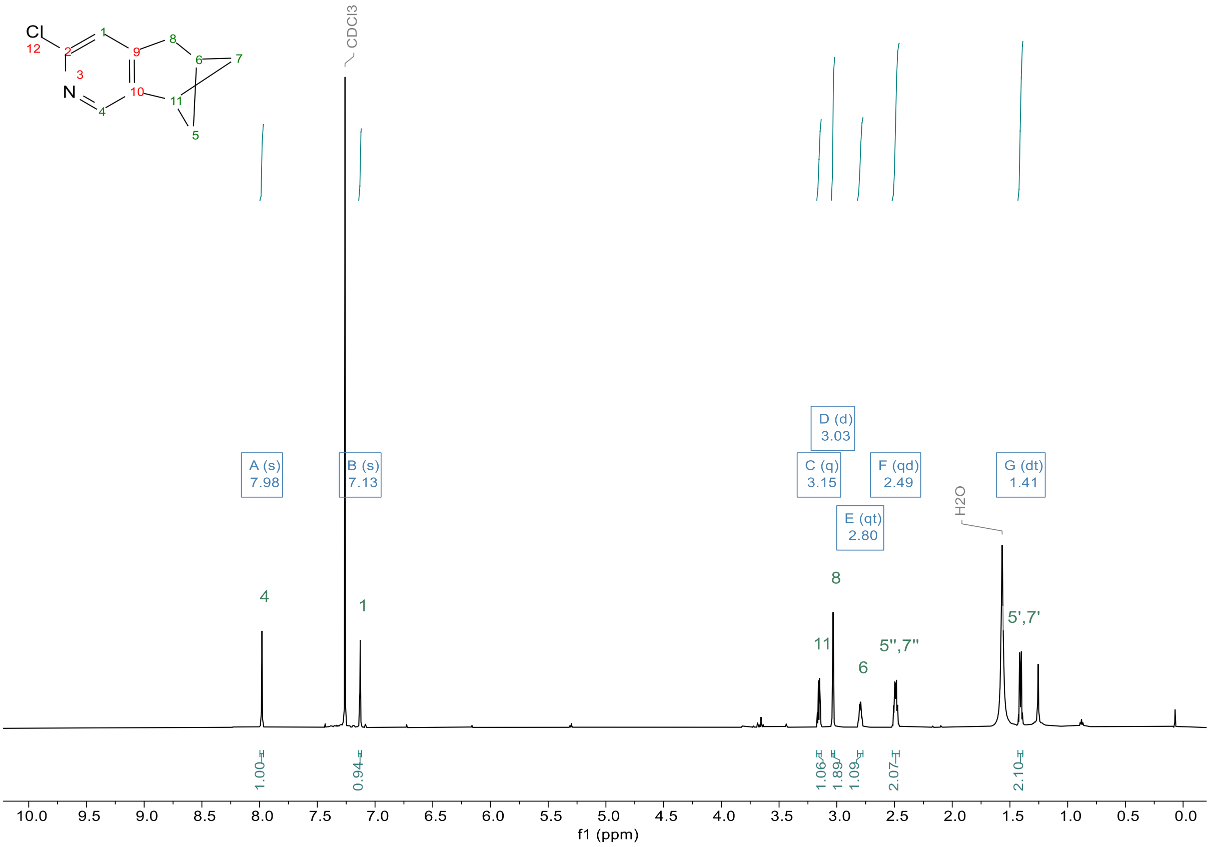

Fig.S99. ^{1}H NMR Spectrum of **33** (Chloroform-d, 298 K).

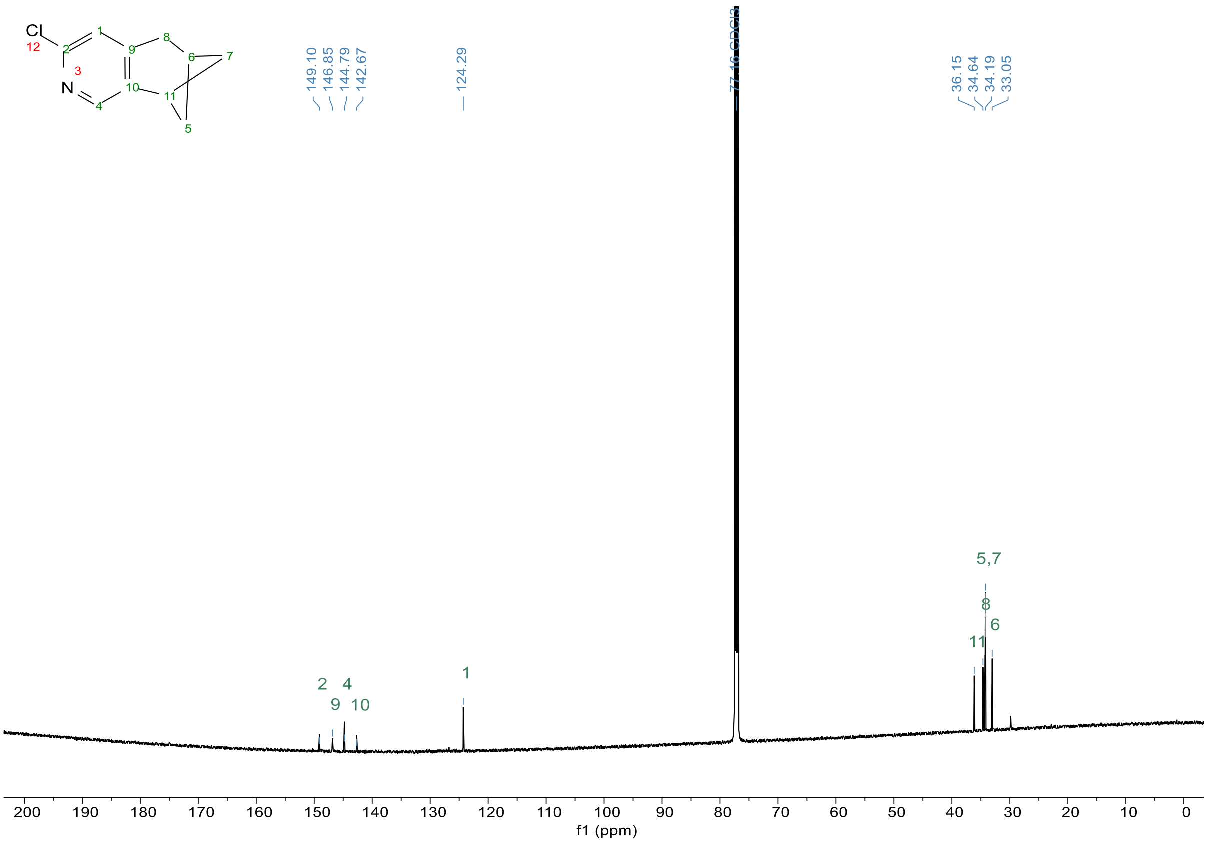

Fig.S100. ^{13}C NMR Spectrum of **33** (Chloroform-d, 298 K).

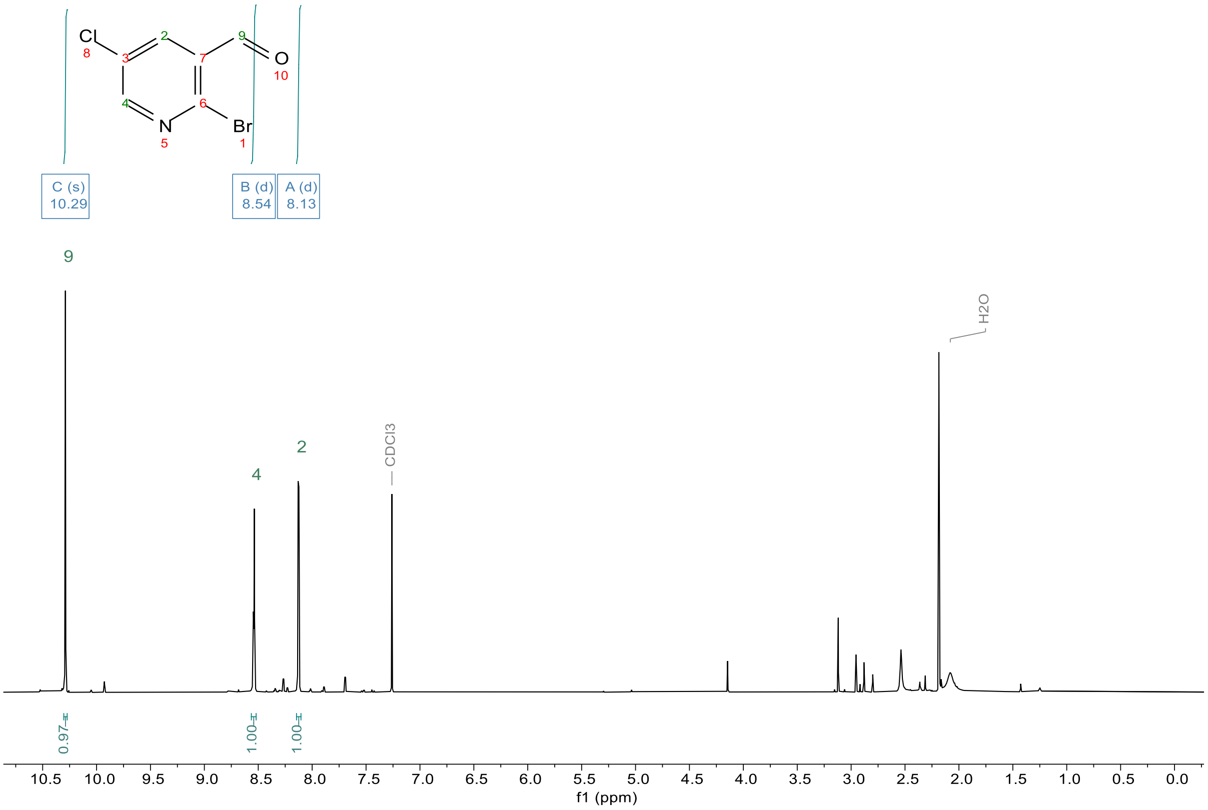

Fig.S101. ^{1}H NMR Spectrum of **S59** (Chloroform-d, 298 K).

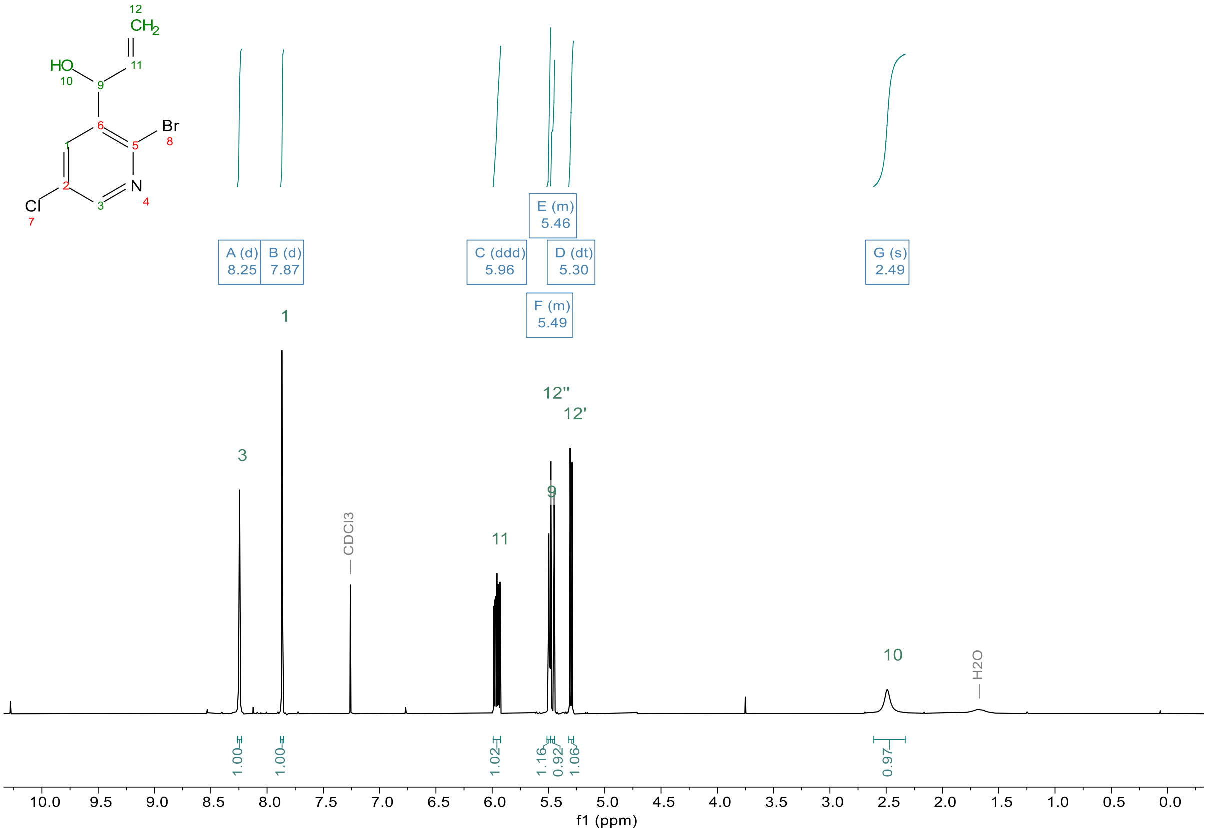

Fig.S102. ^{1}H NMR Spectrum of **S60** (Chloroform-d, 298 K).

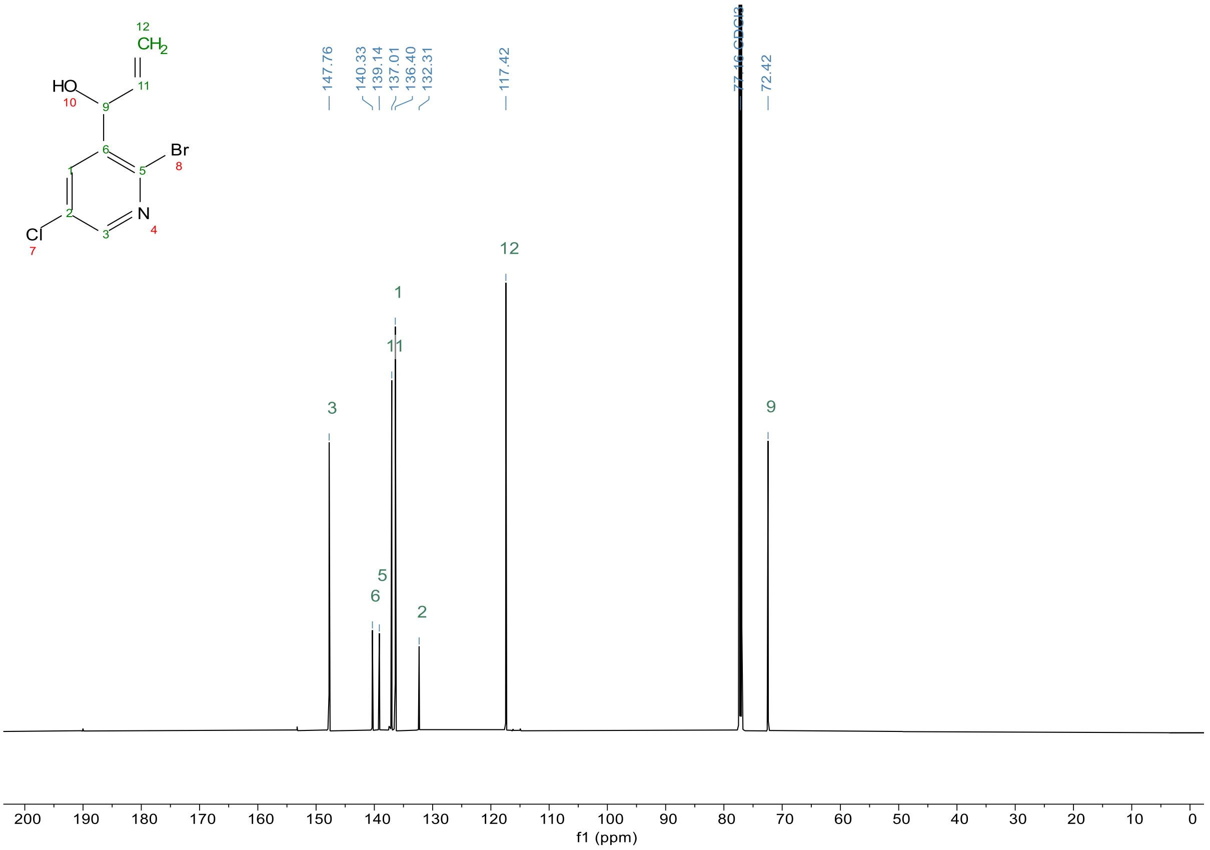

Fig.S103. ^{13}C NMR Spectrum of **S60** (Chloroform-d, 298 K).

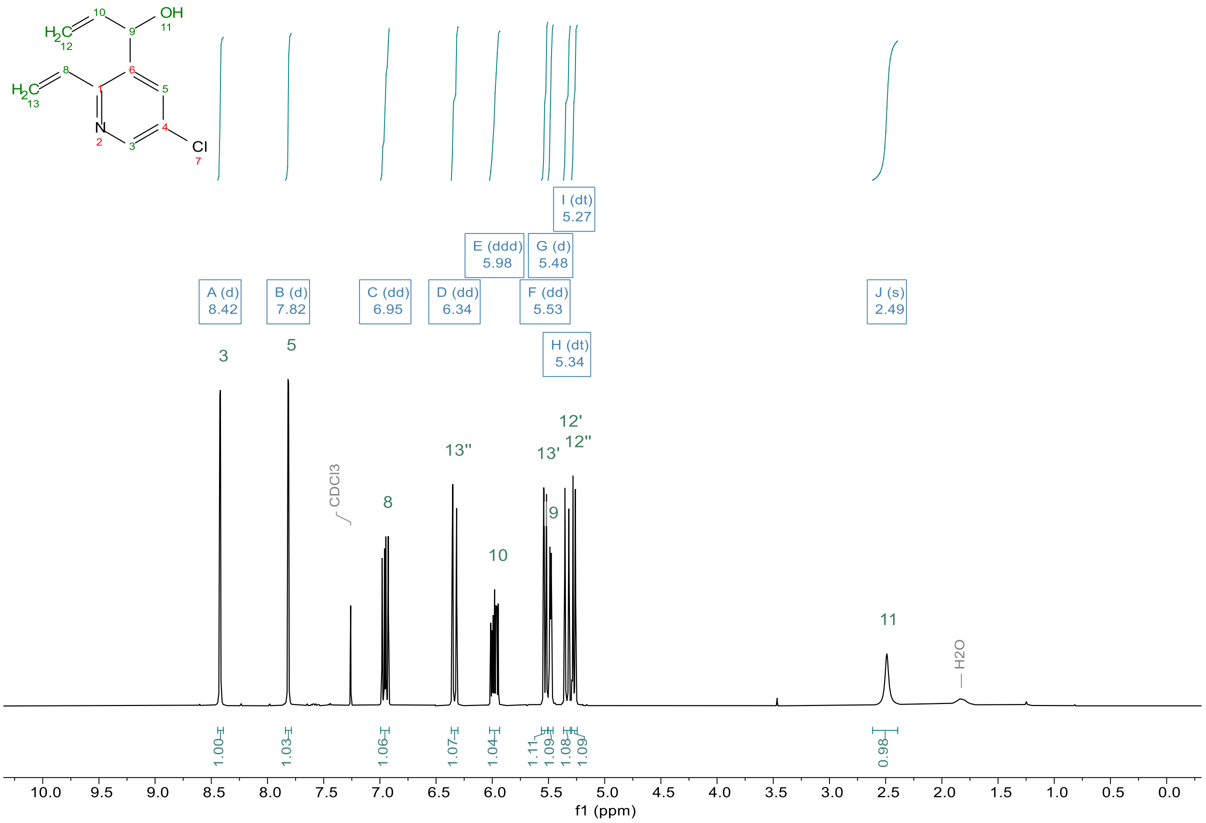

Fig.S104. ^{1}H NMR Spectrum of **S61** (Chloroform-d, 298 K).

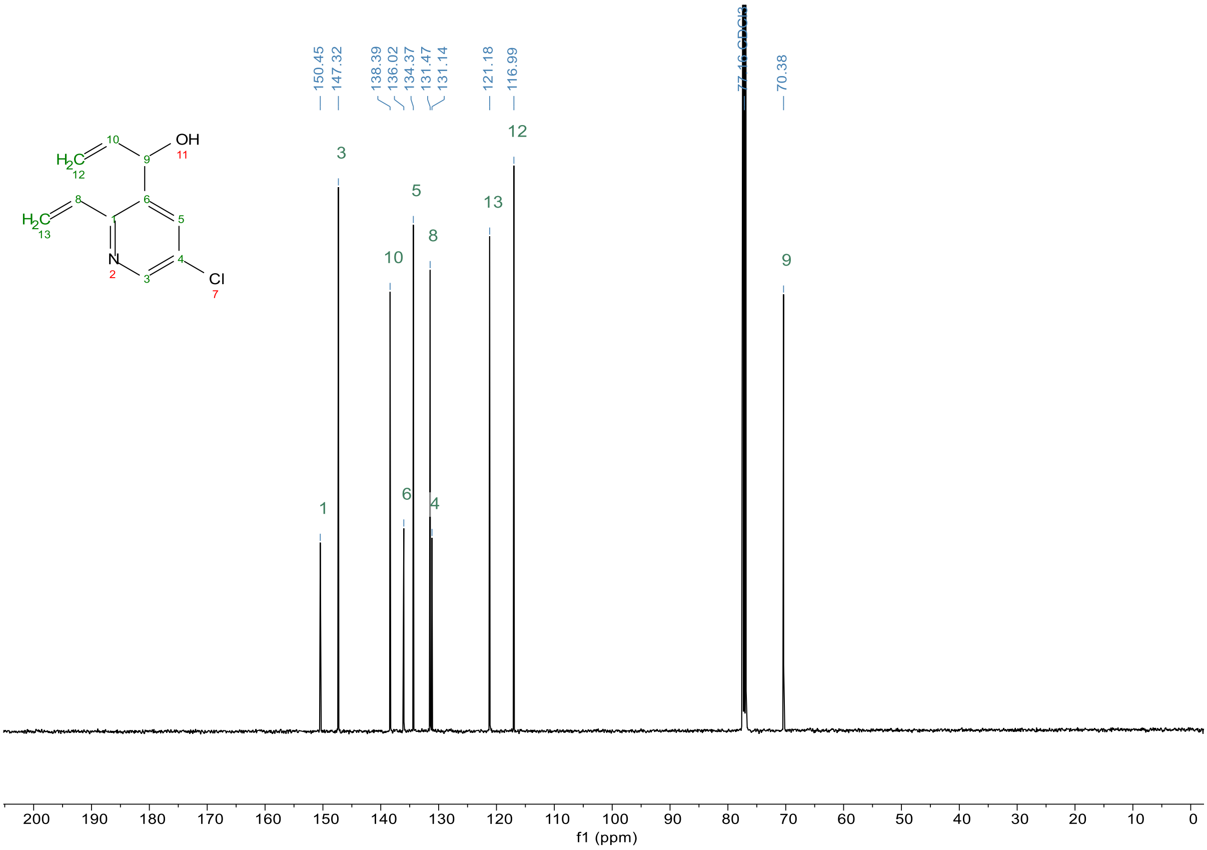

Fig.S105. ^{13}C NMR Spectrum of **S61** (Chloroform-d, 298 K).

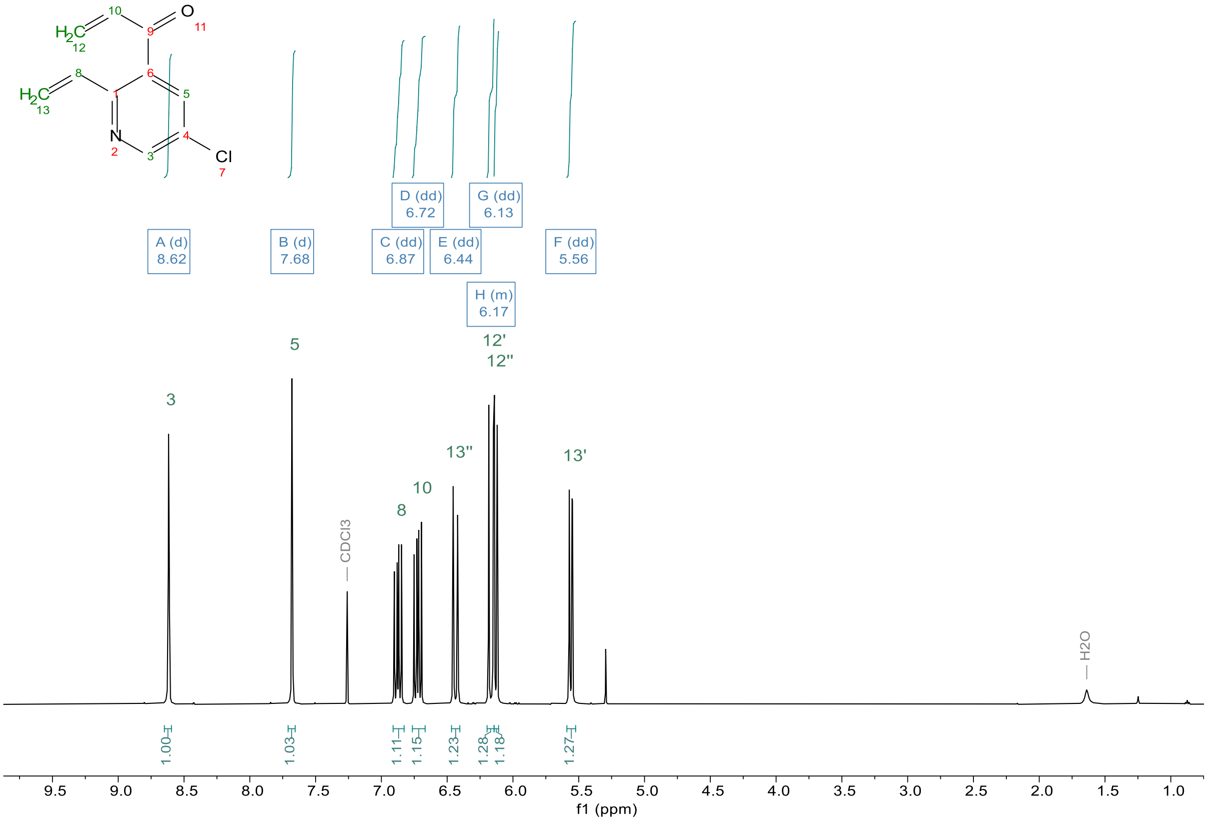

Fig.S106. ^{1}H NMR Spectrum of **S62** (Chloroform-d, 298 K).

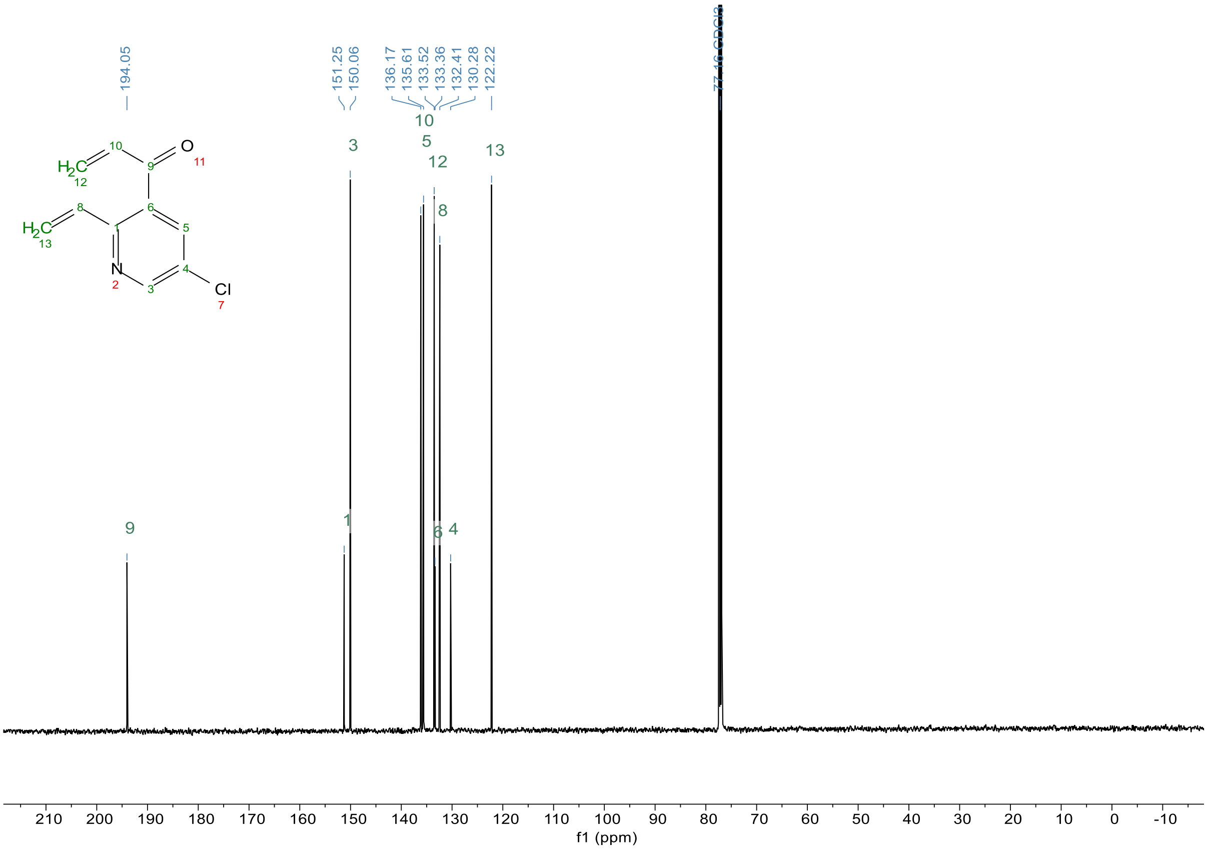

Fig.S107. ^{13}C NMR Spectrum of **S62** (Chloroform-d, 298 K).

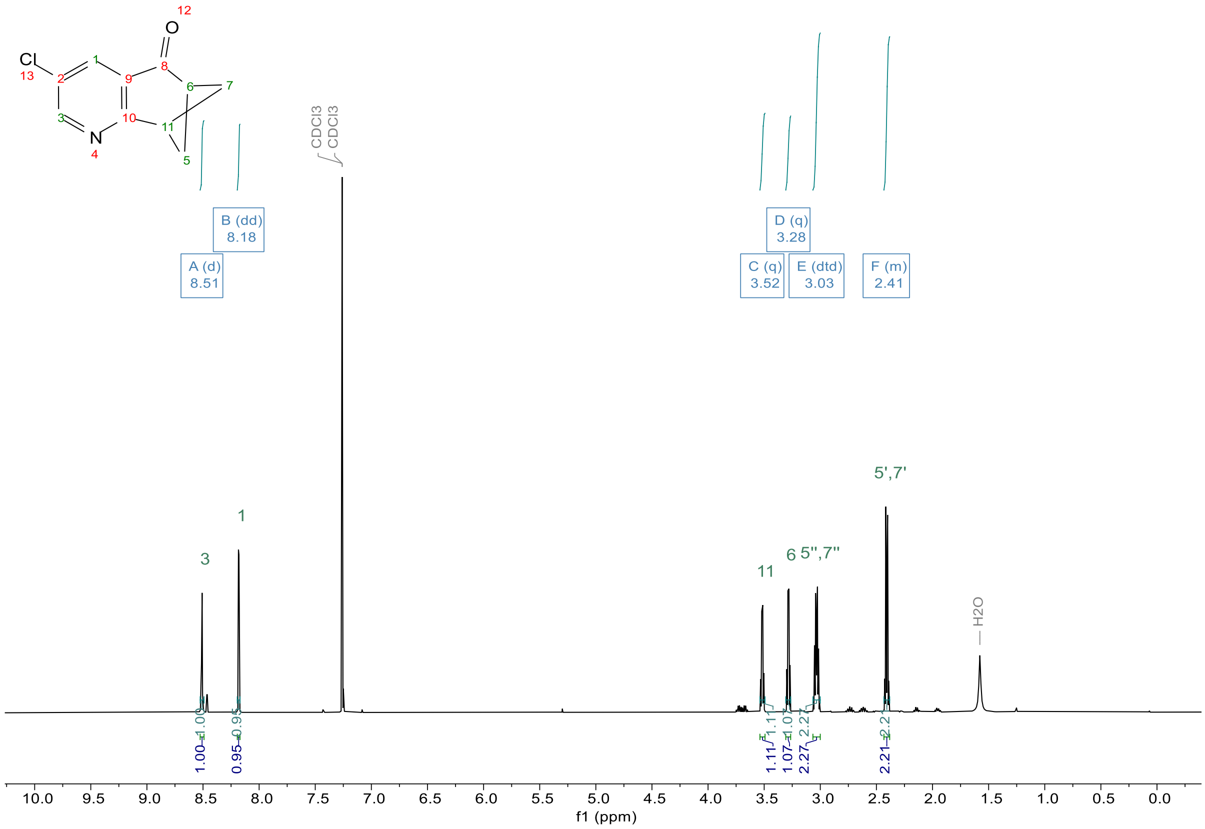

Fig.108. ^{1}H NMR Spectrum of **27** (Chloroform-d, 298 K).

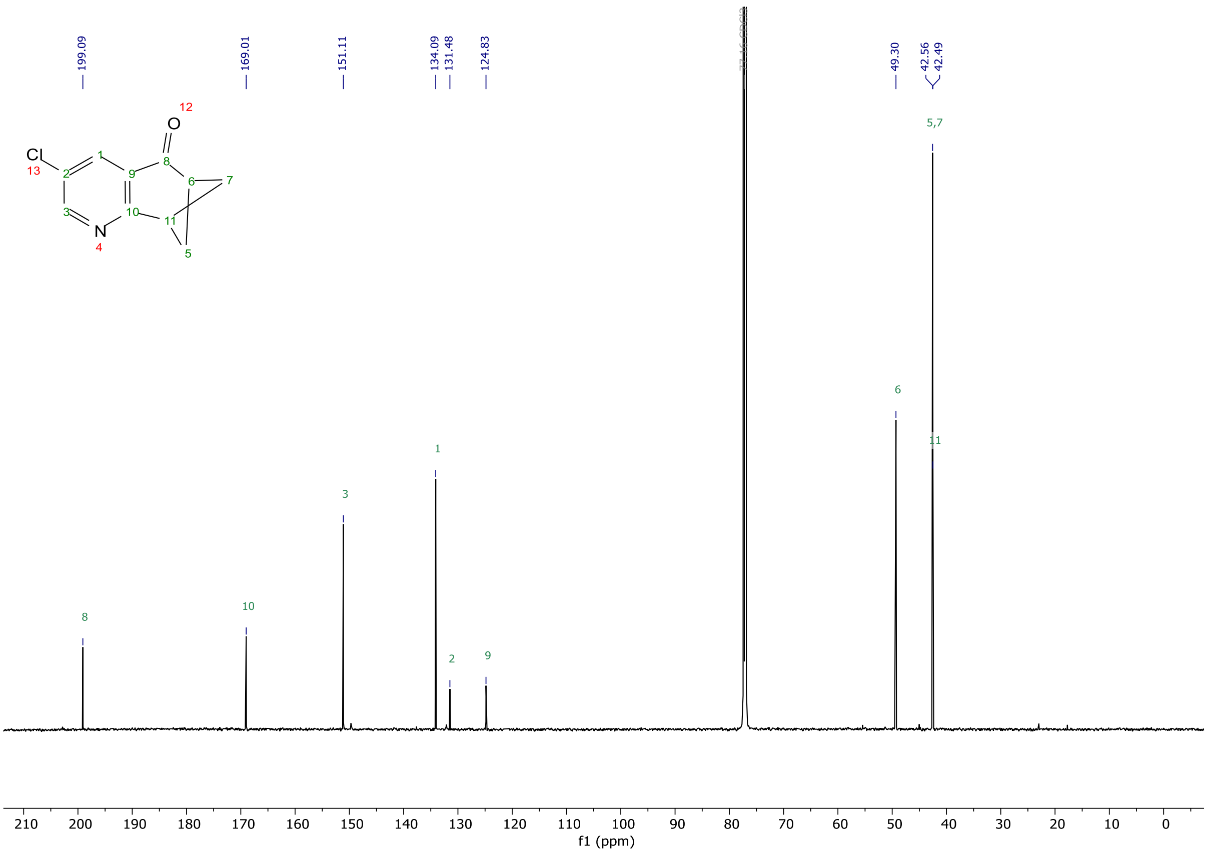

Fig.S109. ^{13}C NMR Spectrum of **27** (Chloroform-d, 298 K).

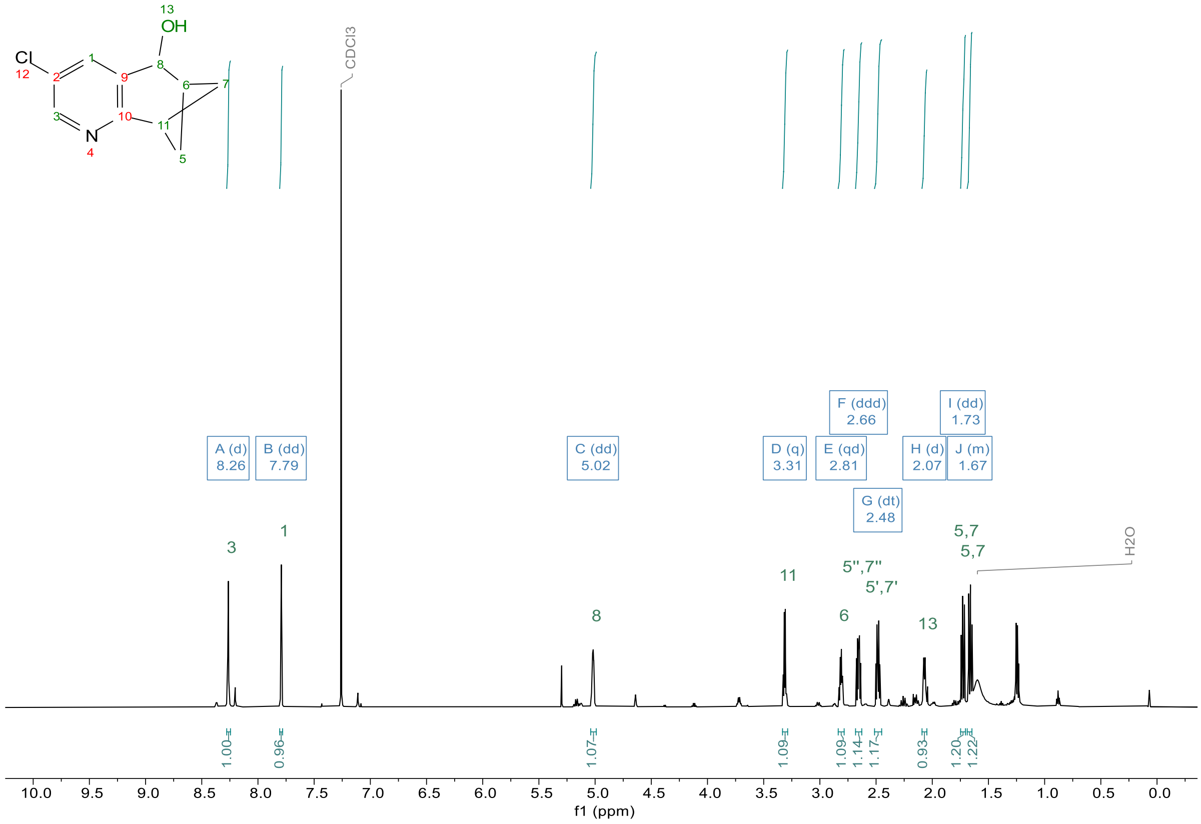

Fig.S110. ^{1}H NMR Spectrum of **S63** (Chloroform-d, 298 K).

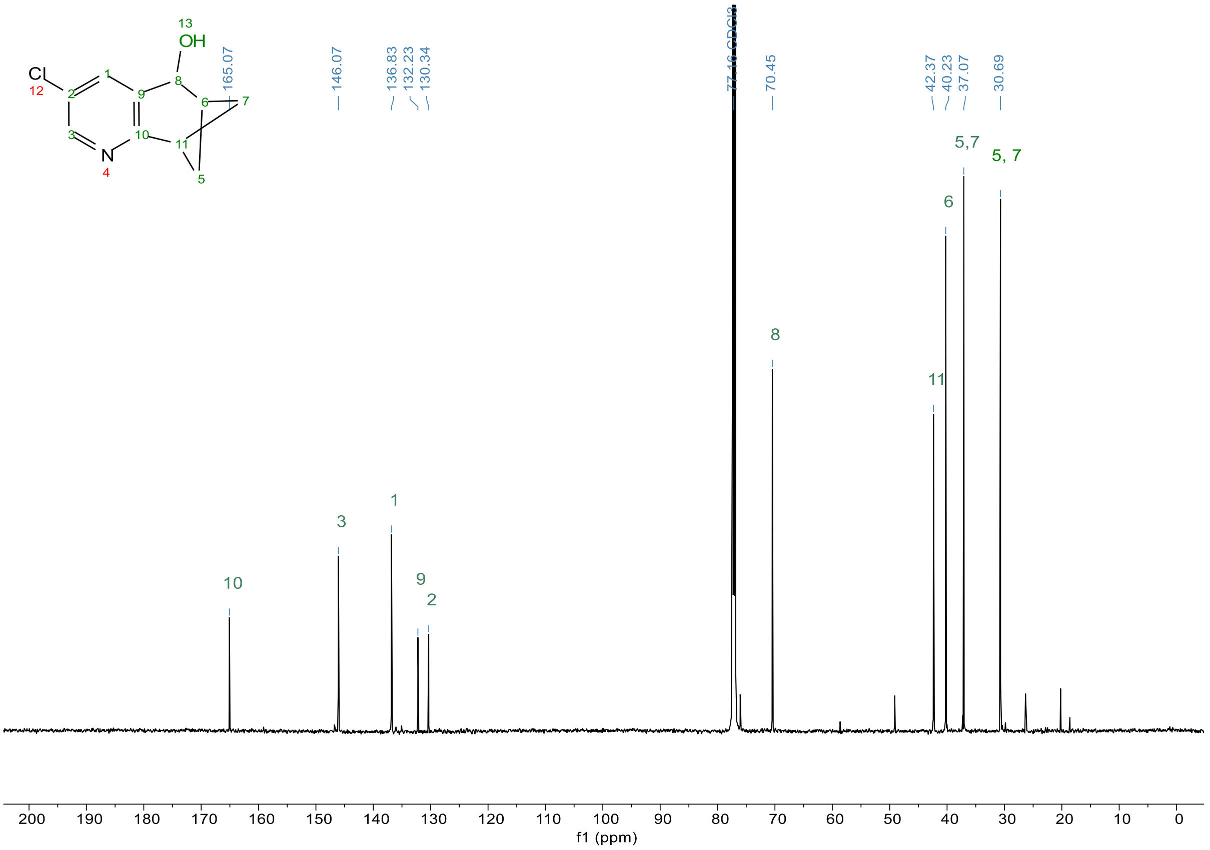

Fig.S111. ^{13}C NMR Spectrum of **S63** (Chloroform-d, 298 K).

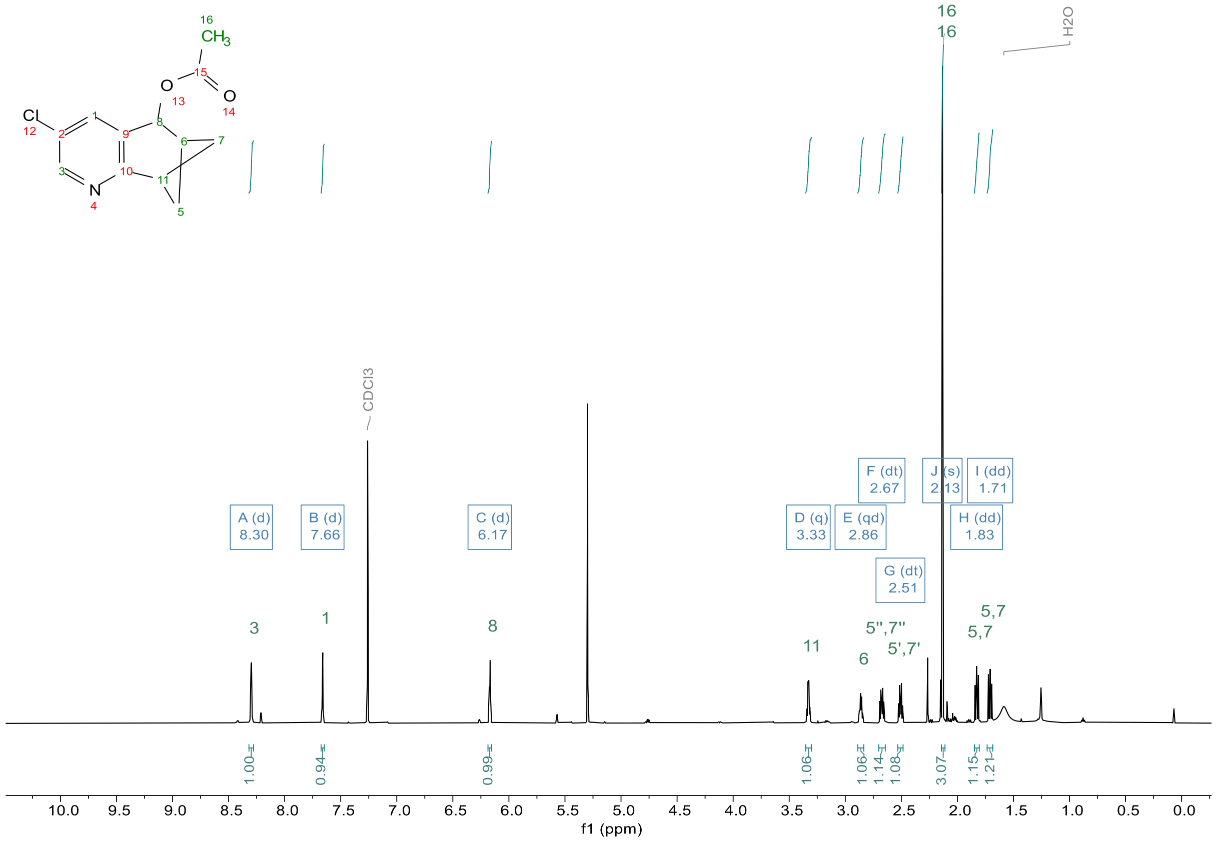

Fig.S112. ^{1}H NMR Spectrum of **S64** (Chloroform-d, 298 K).

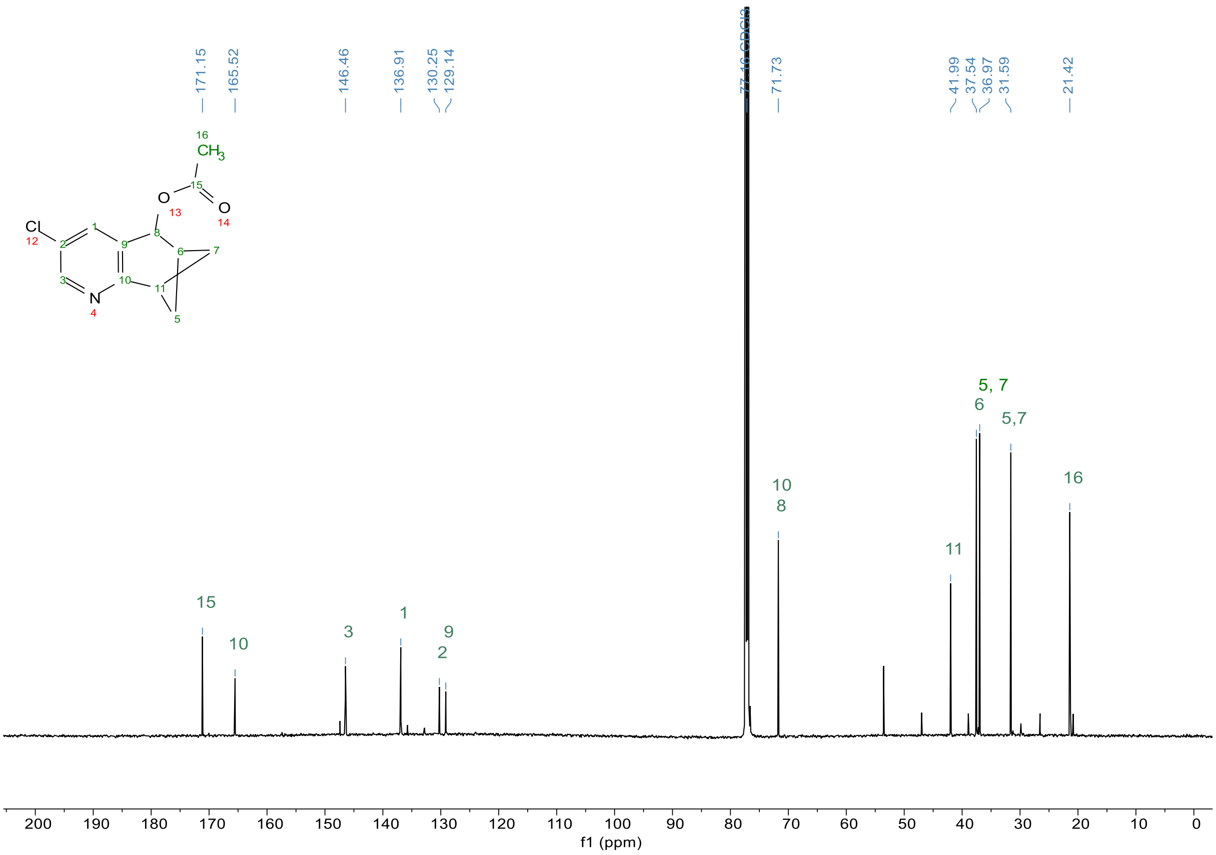

Fig.S113. ^{13}C NMR Spectrum of **S64** (Chloroform-d, 298 K).

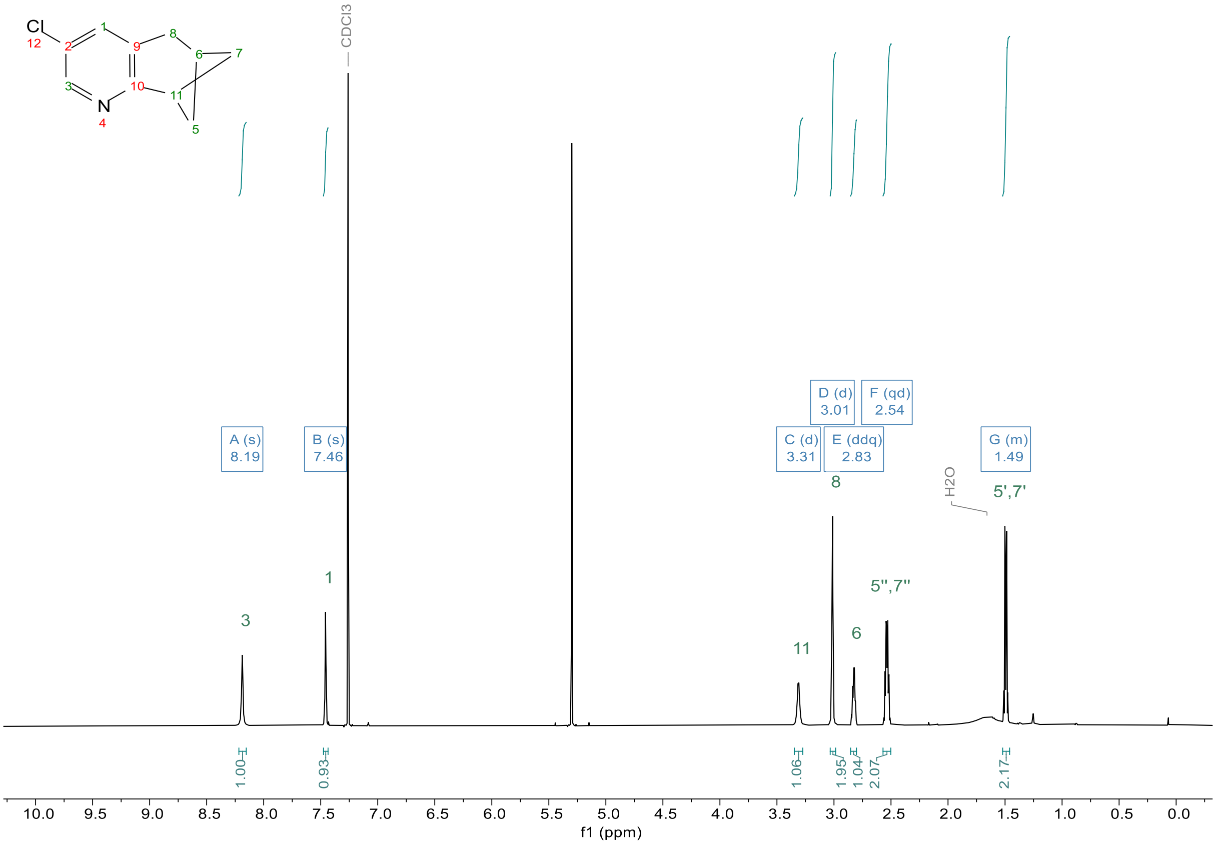

Fig.S114. ^{1}H NMR Spectrum of **32** (Chloroform-d, 298 K).

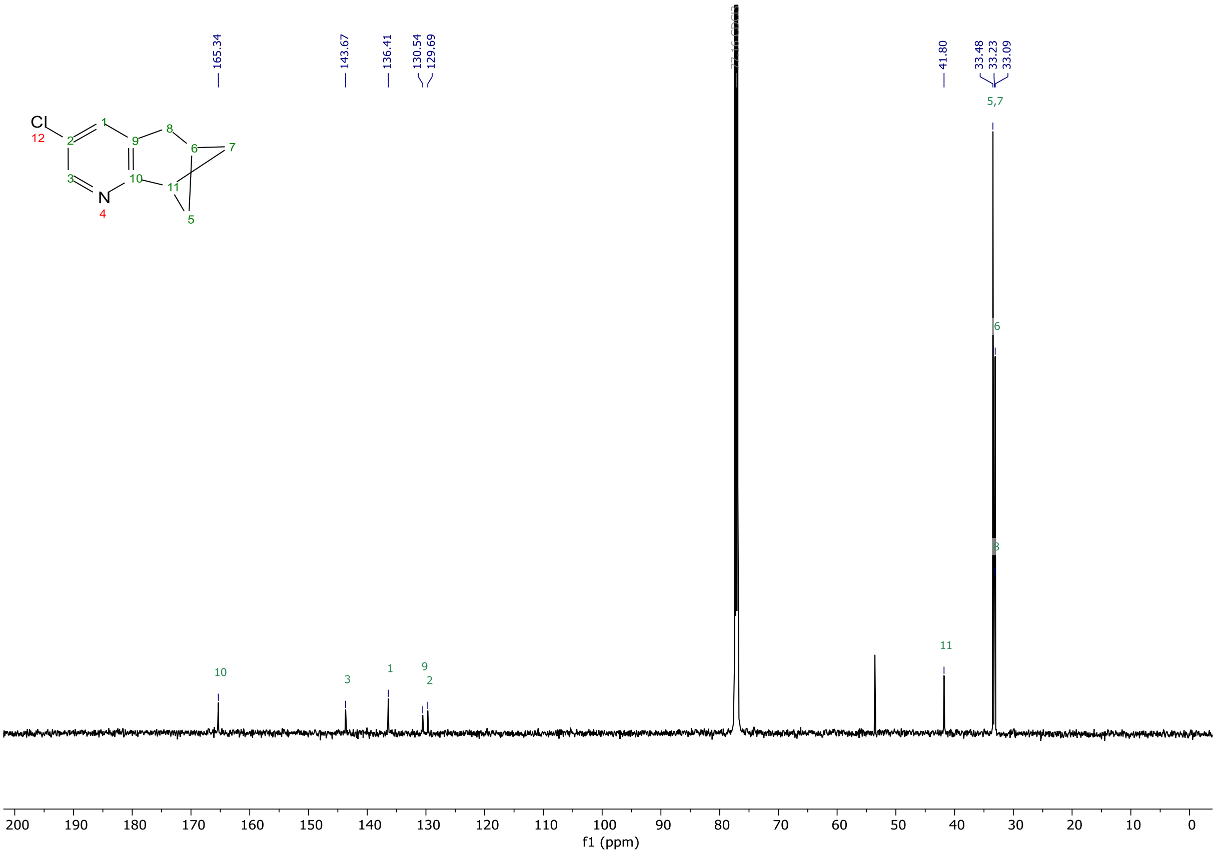

Fig.S115. ^{13}C NMR Spectrum of **32** (Chloroform-d, 298 K).

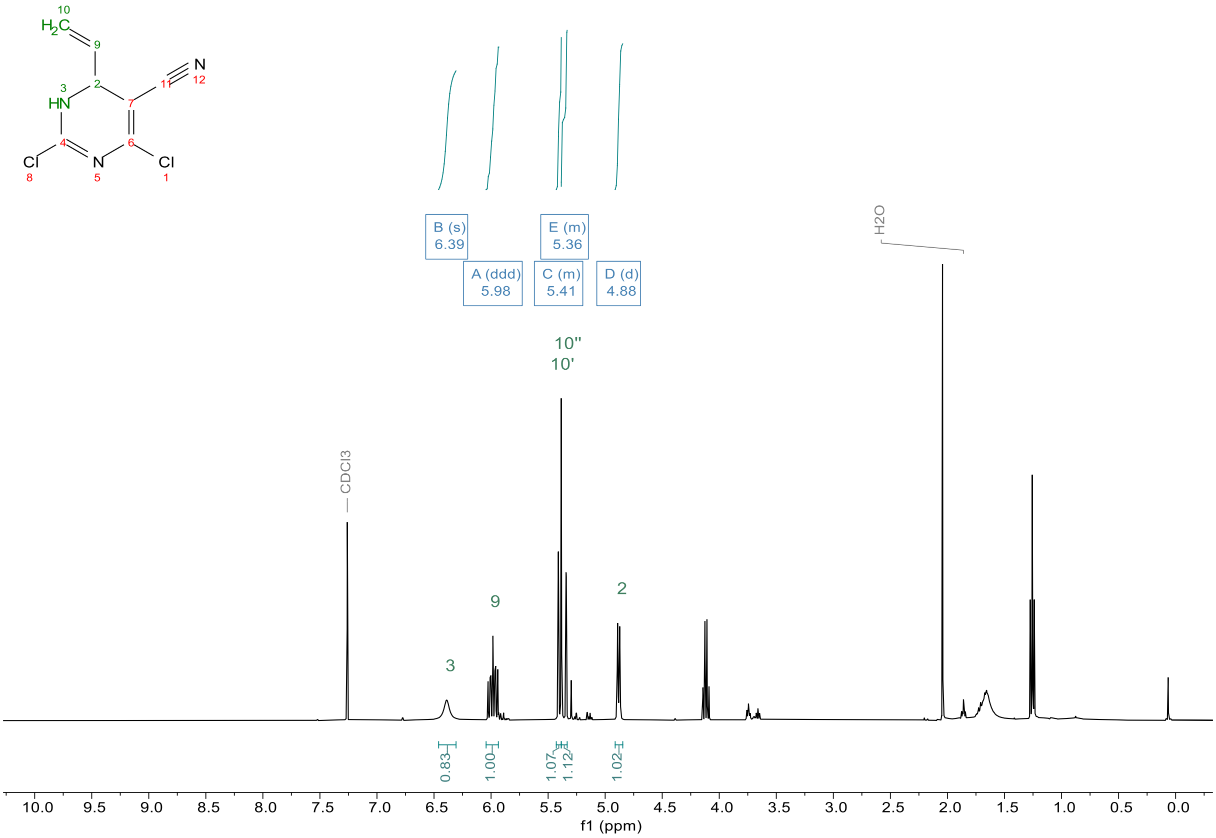

Fig.S116. Crude ^{1}H NMR Spectrum of **S67** (Chloroform-d, 298 K).

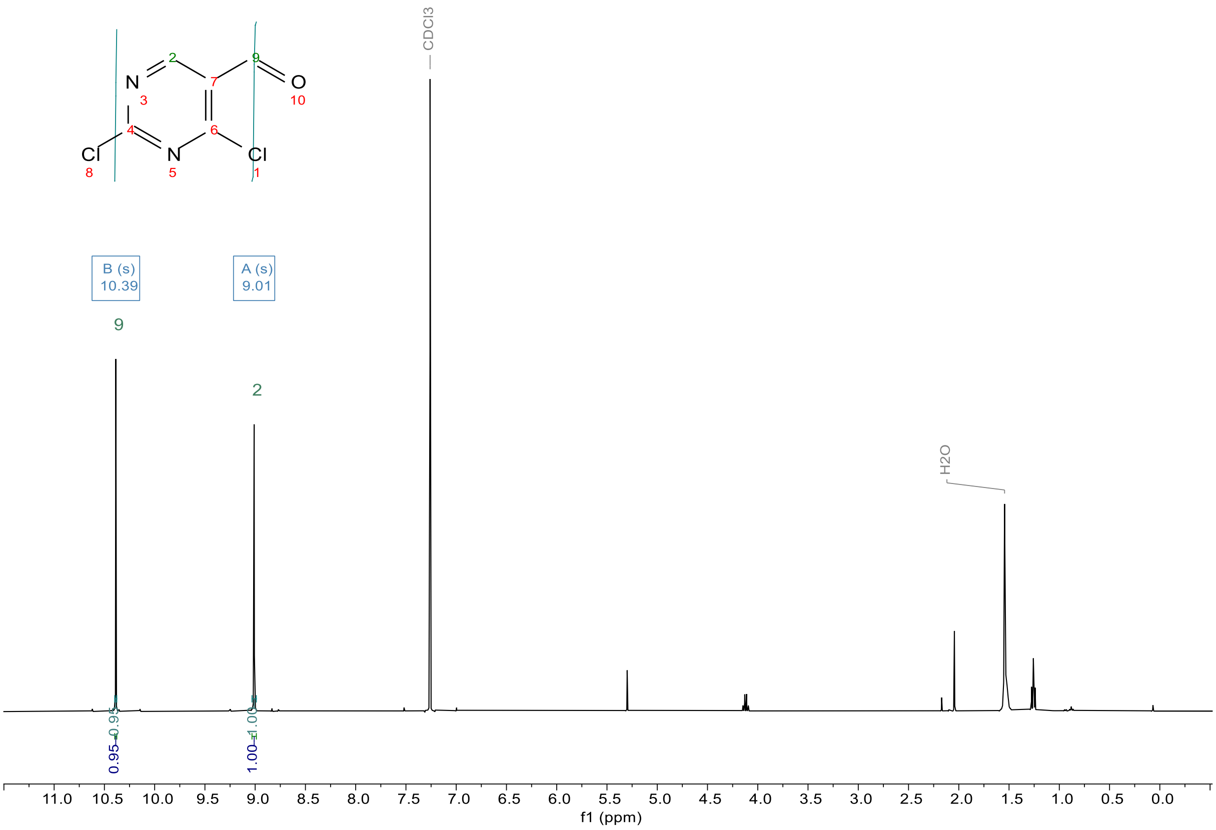

Fig.S117. ^{1}H NMR Spectrum of **S68** (Chloroform-d, 298 K).

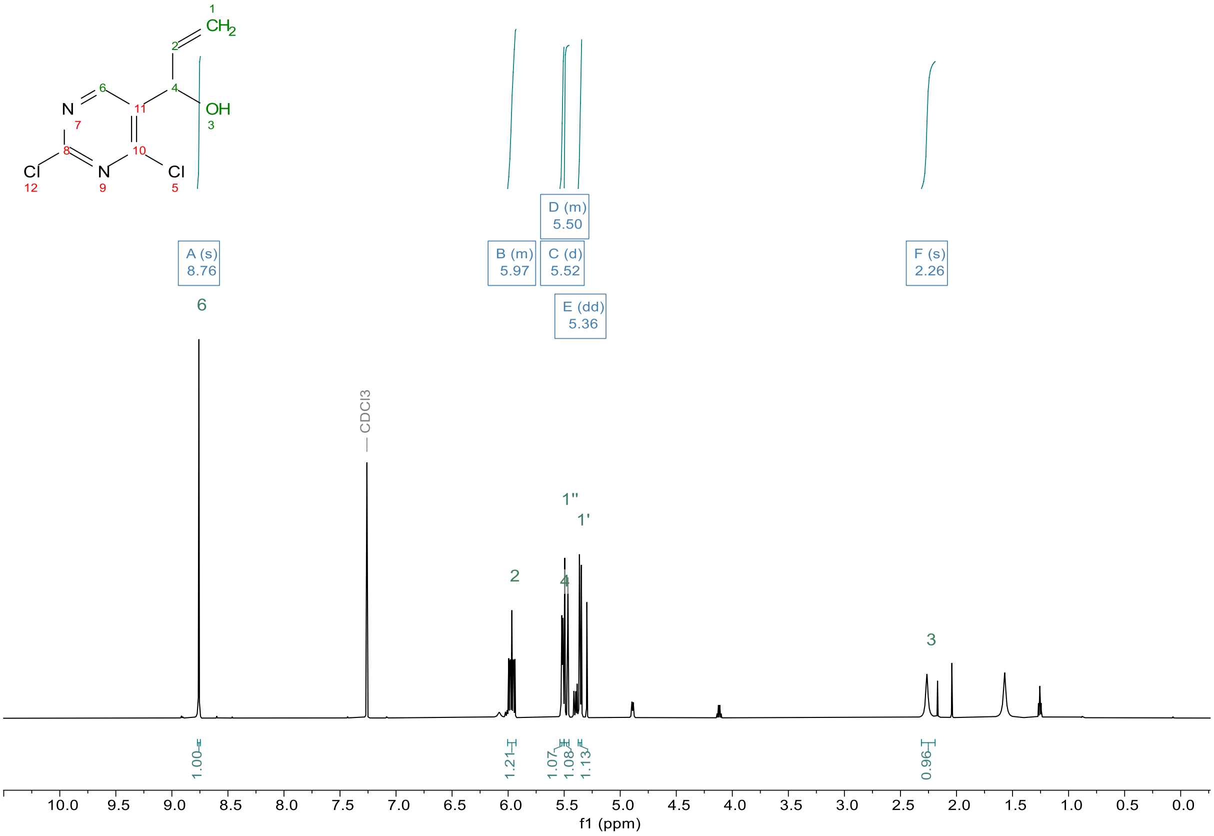

Fig.S118. ^{1}H NMR Spectrum of **S69** (Chloroform-d, 298 K).

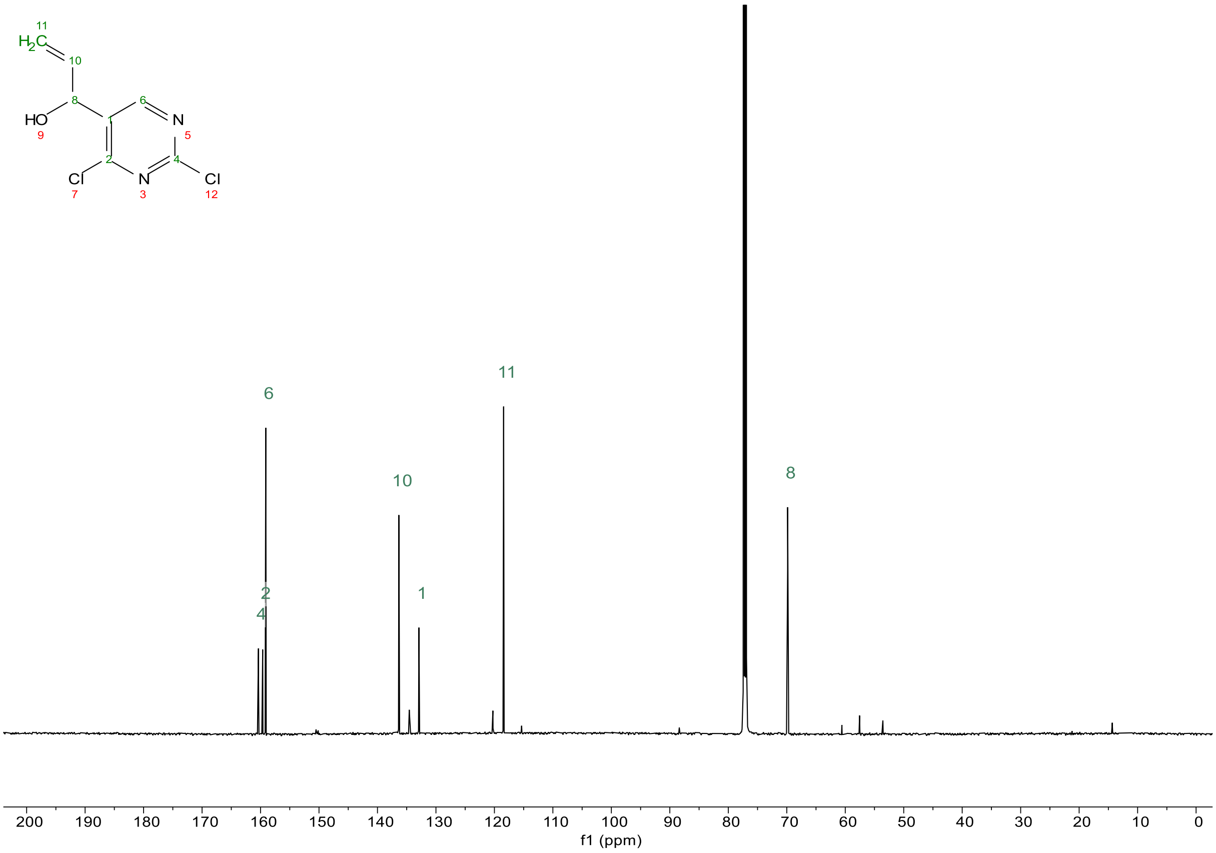

Fig.S119. ^{13}C NMR Spectrum of **S69** (Chloroform-d, 298 K).

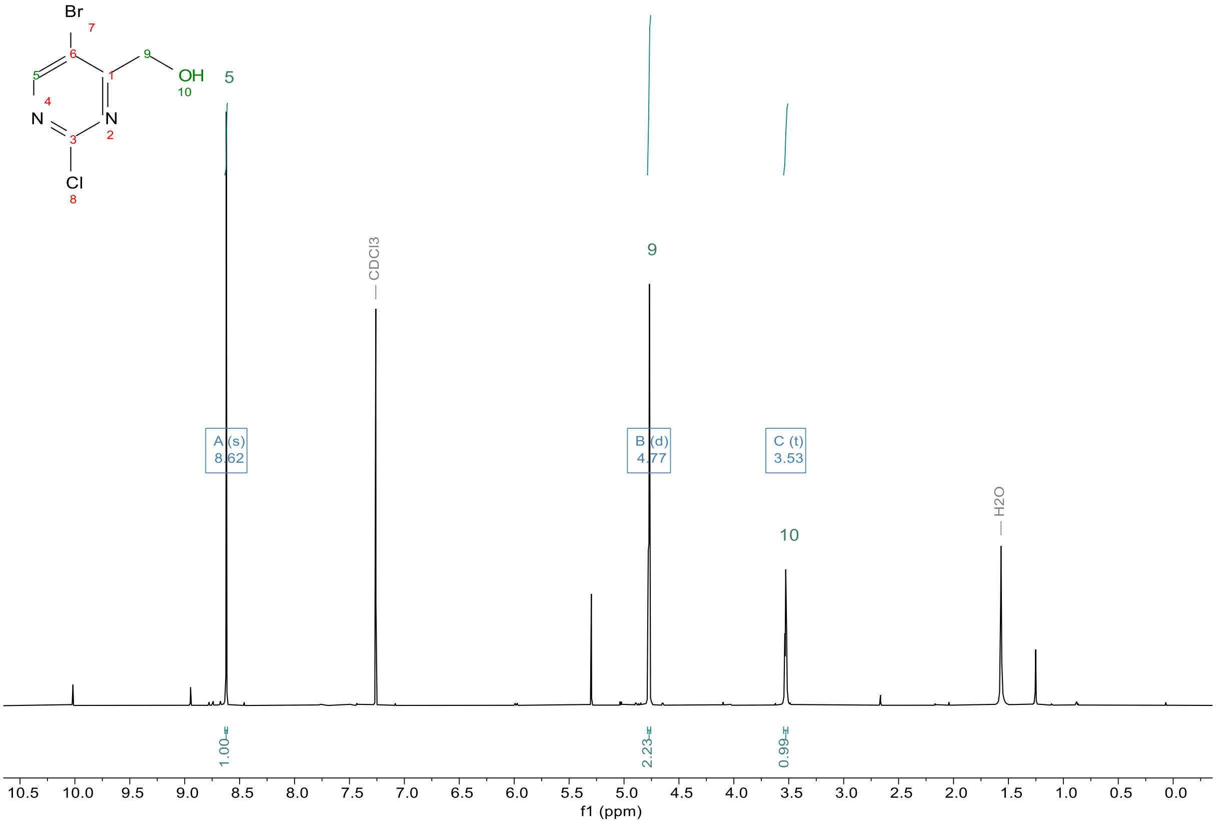

Fig.S120. ^{1}H NMR Spectrum of **S73** (Chloroform-d, 298 K).

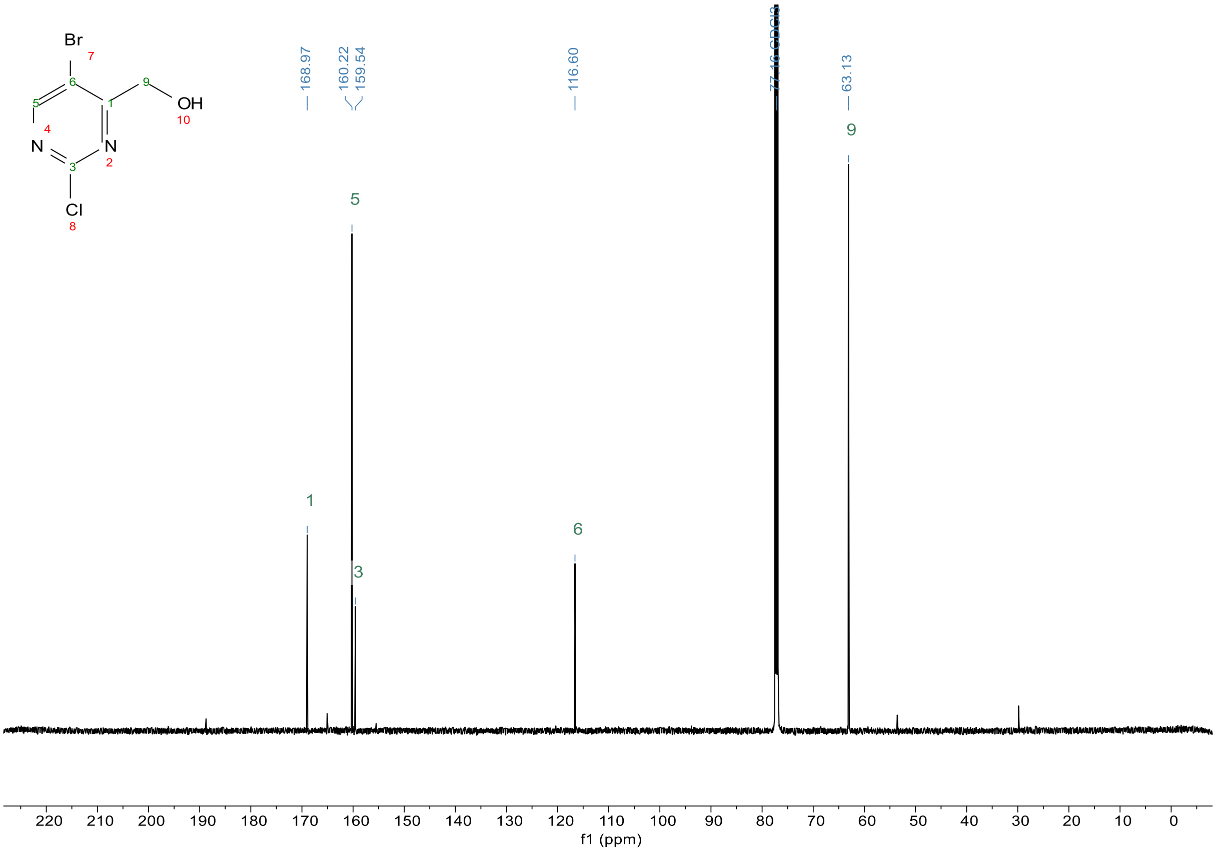

Fig.S121. ^{13}C NMR Spectrum of **S73** (Chloroform-d, 298 K).

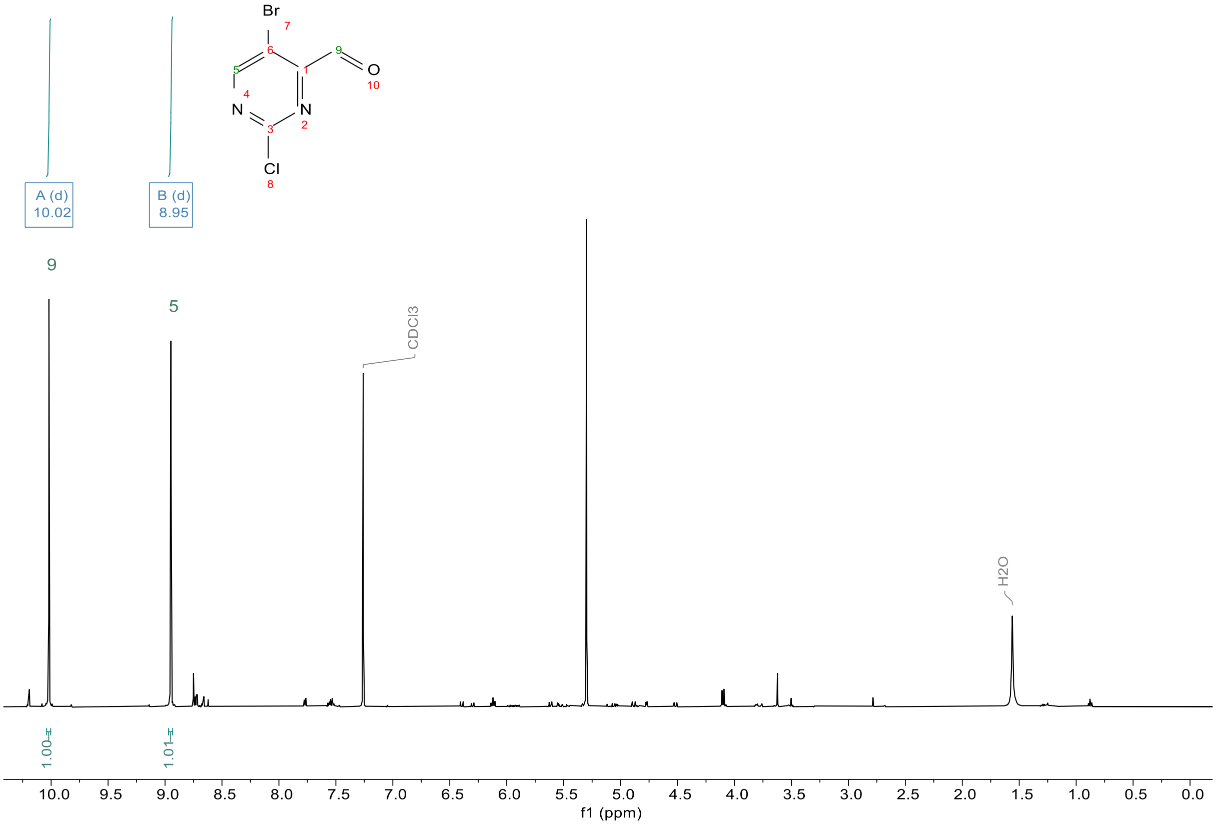

Fig.S122. ^{1}H NMR Spectrum of **S74** (Chloroform-d, 298 K).

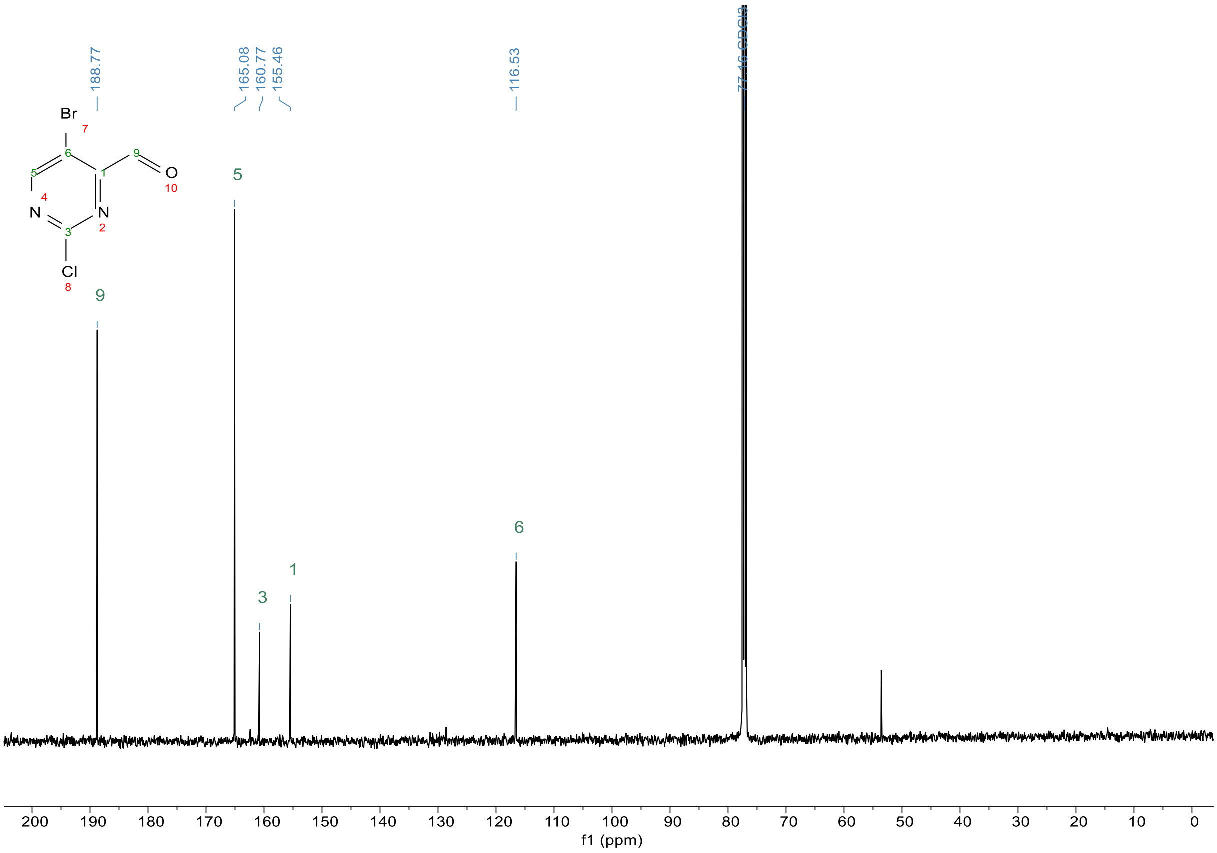

Fig.S123. ^{13}C NMR Spectrum of **S74** (Chloroform-d, 298 K).

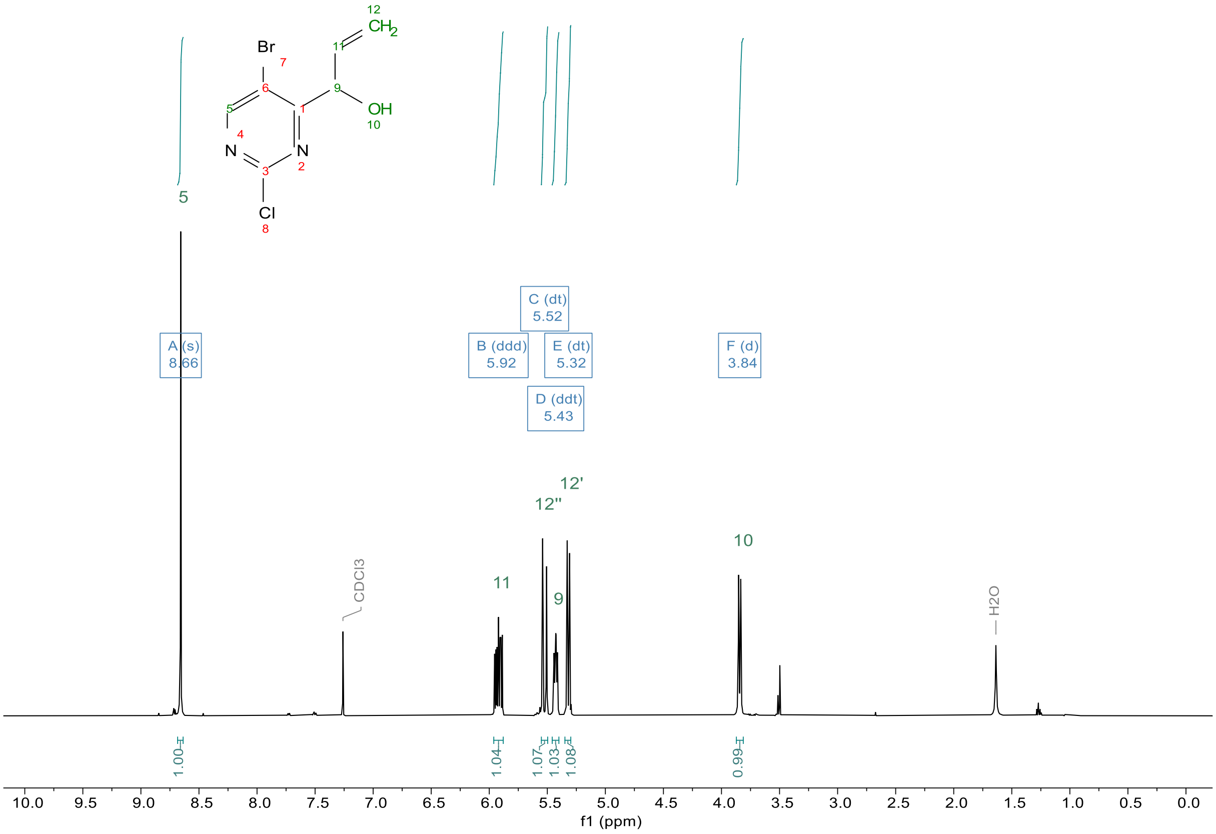

Fig.S124. ^{1}H NMR Spectrum of **S75** (Chloroform-d, 298 K).

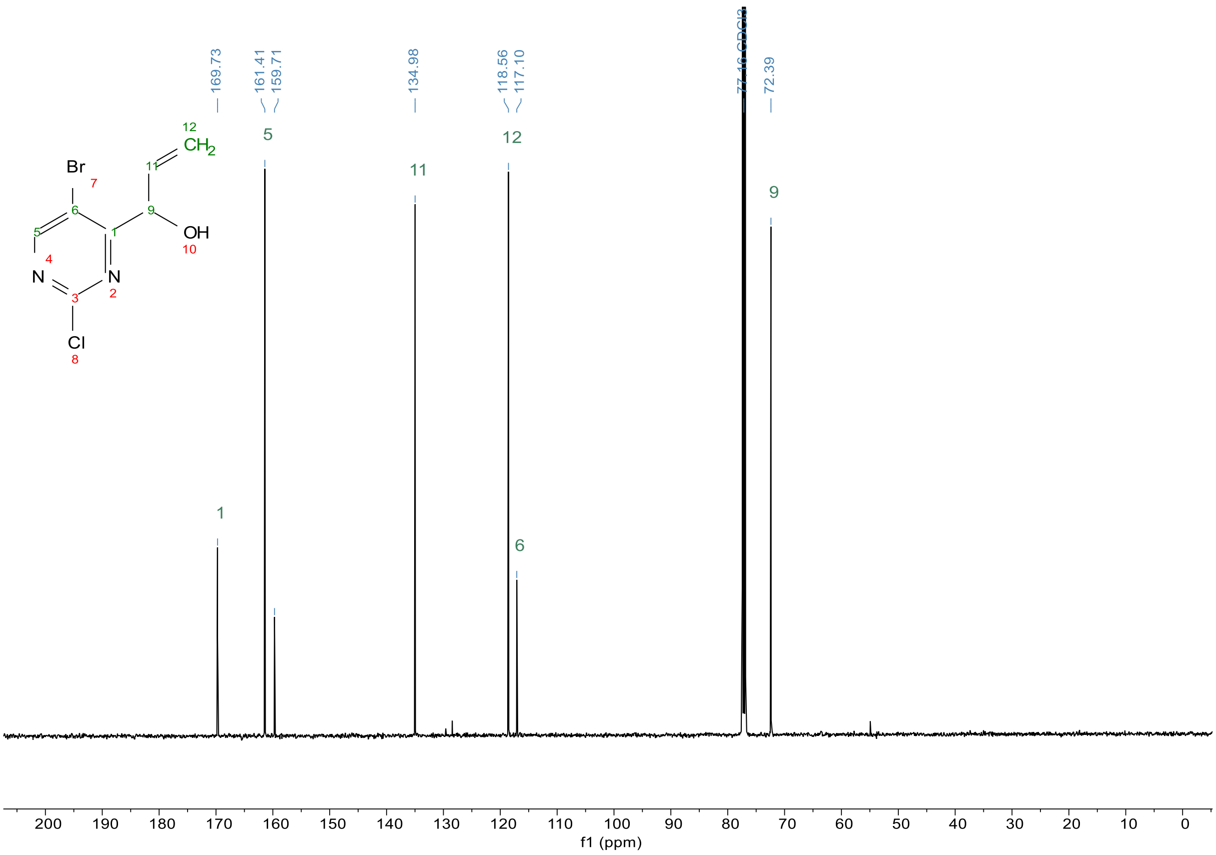

Fig.S125. ^{13}C NMR Spectrum of **S75** (Chloroform-d, 298 K).

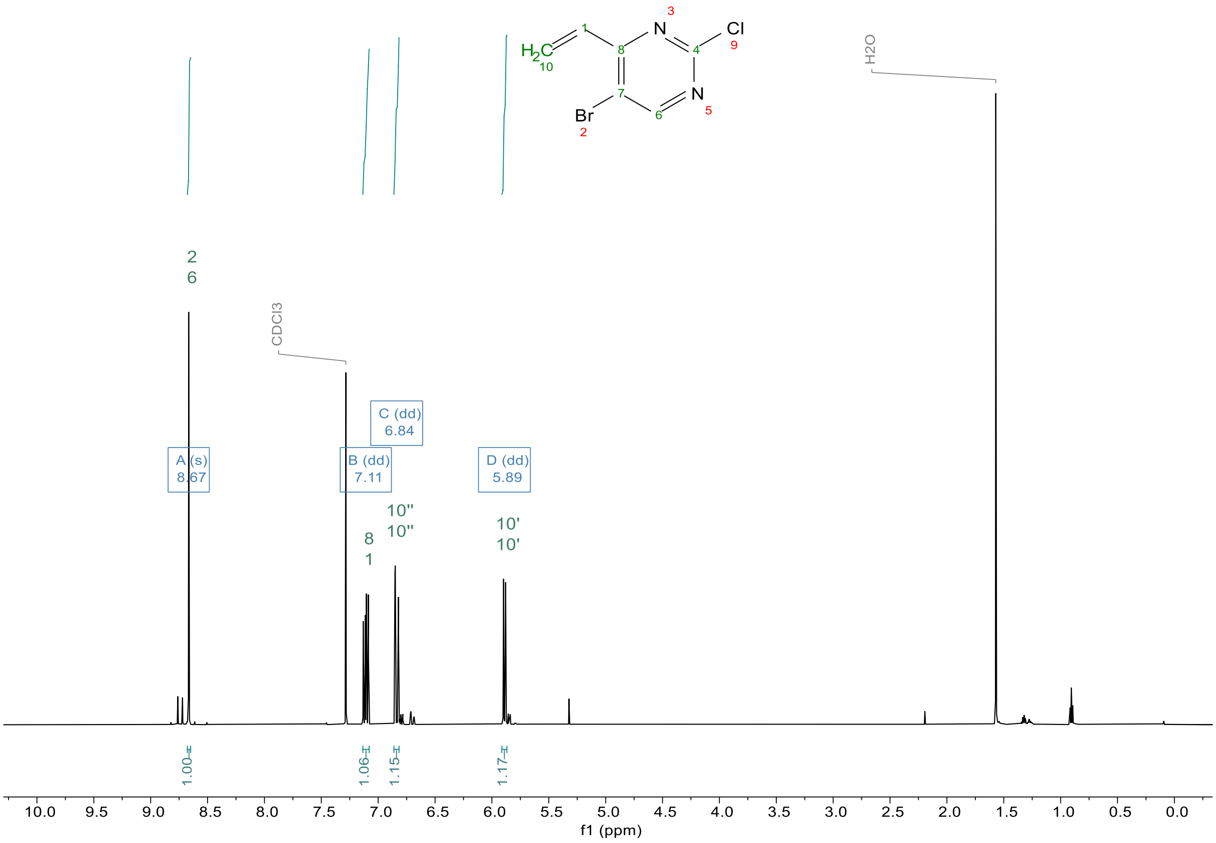

Fig.S126. ^{1}H NMR Spectrum of **S78** (Chloroform-d, 298 K).

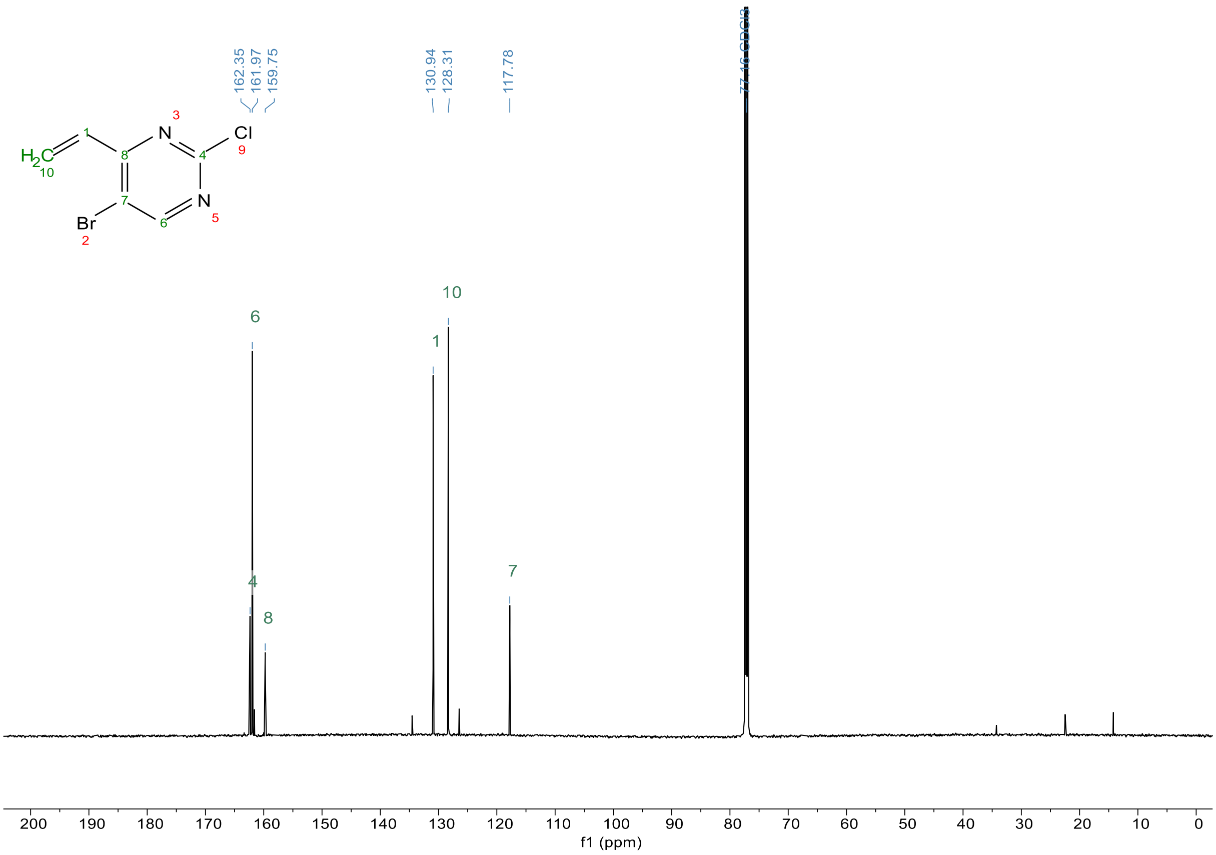

Fig.S127. ^{13}C NMR Spectrum of **S78** (Chloroform-d, 298 K).

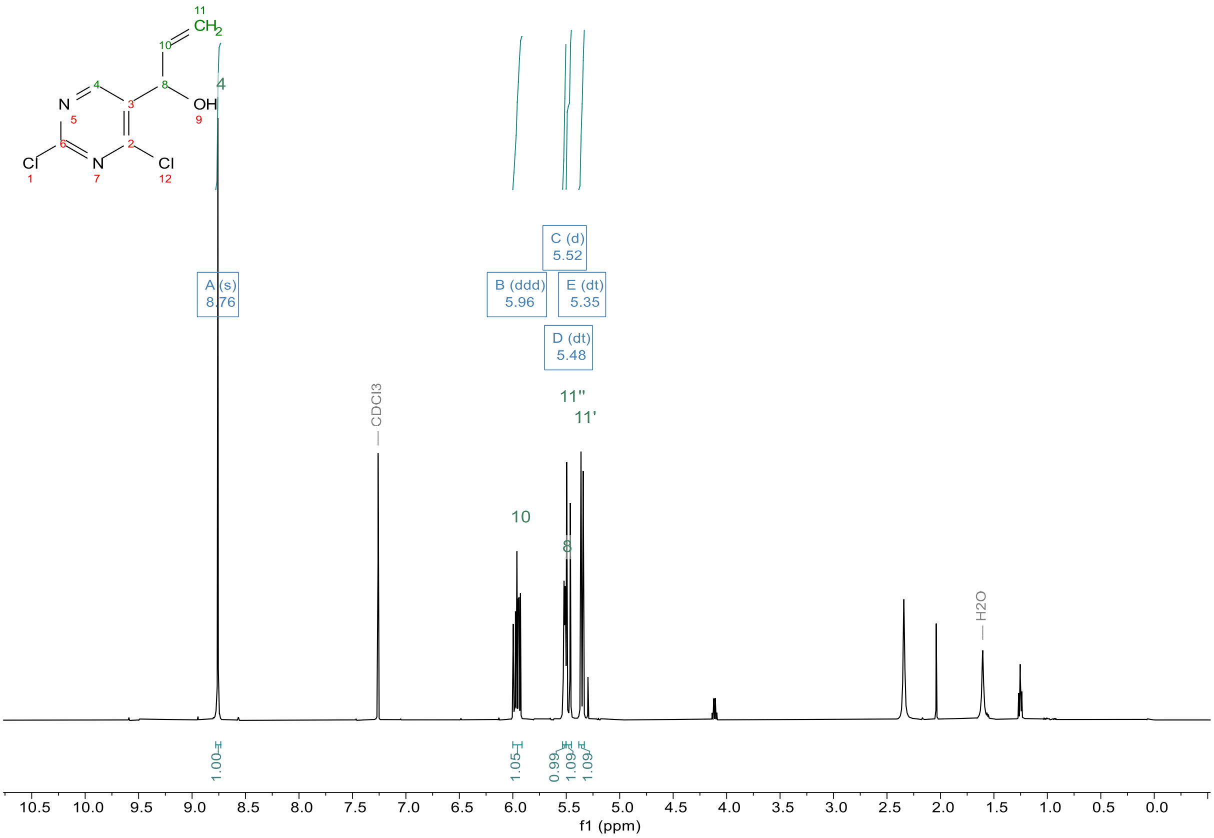

Fig.S128. ^{1}H NMR Spectrum of **S69** (Chloroform-d, 298 K).

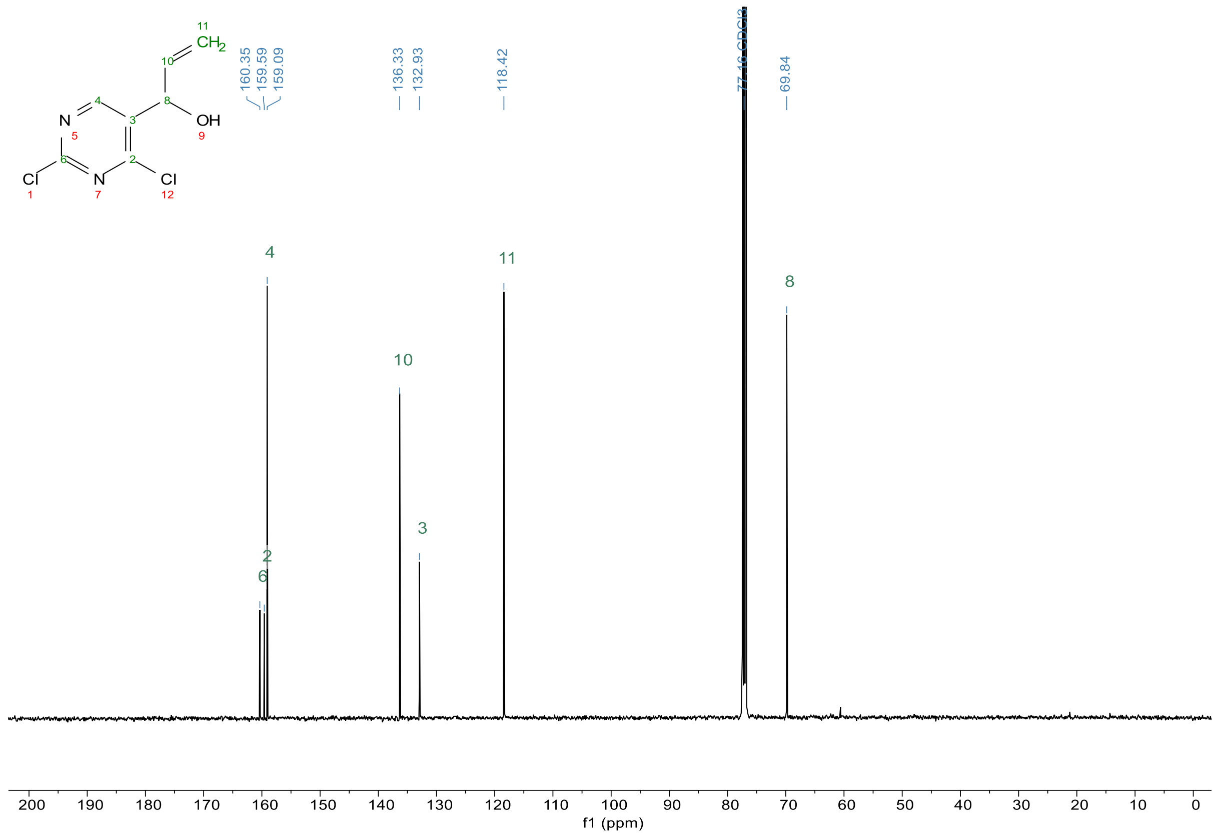

Fig.S129. ^{13}C NMR Spectrum of **S69** (Chloroform-d, 298 K).

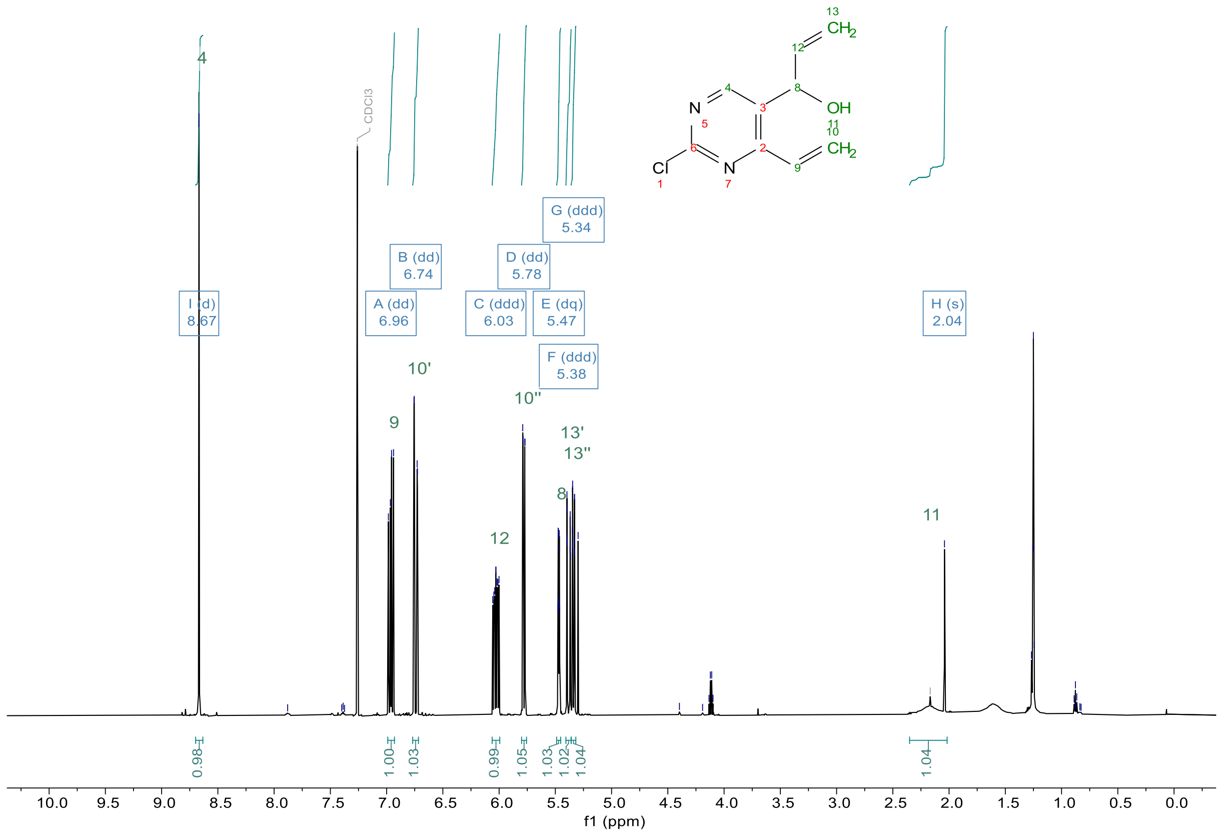

Fig.S130. ^{1}H NMR Spectrum of **S79** (Chloroform-d, 298 K).

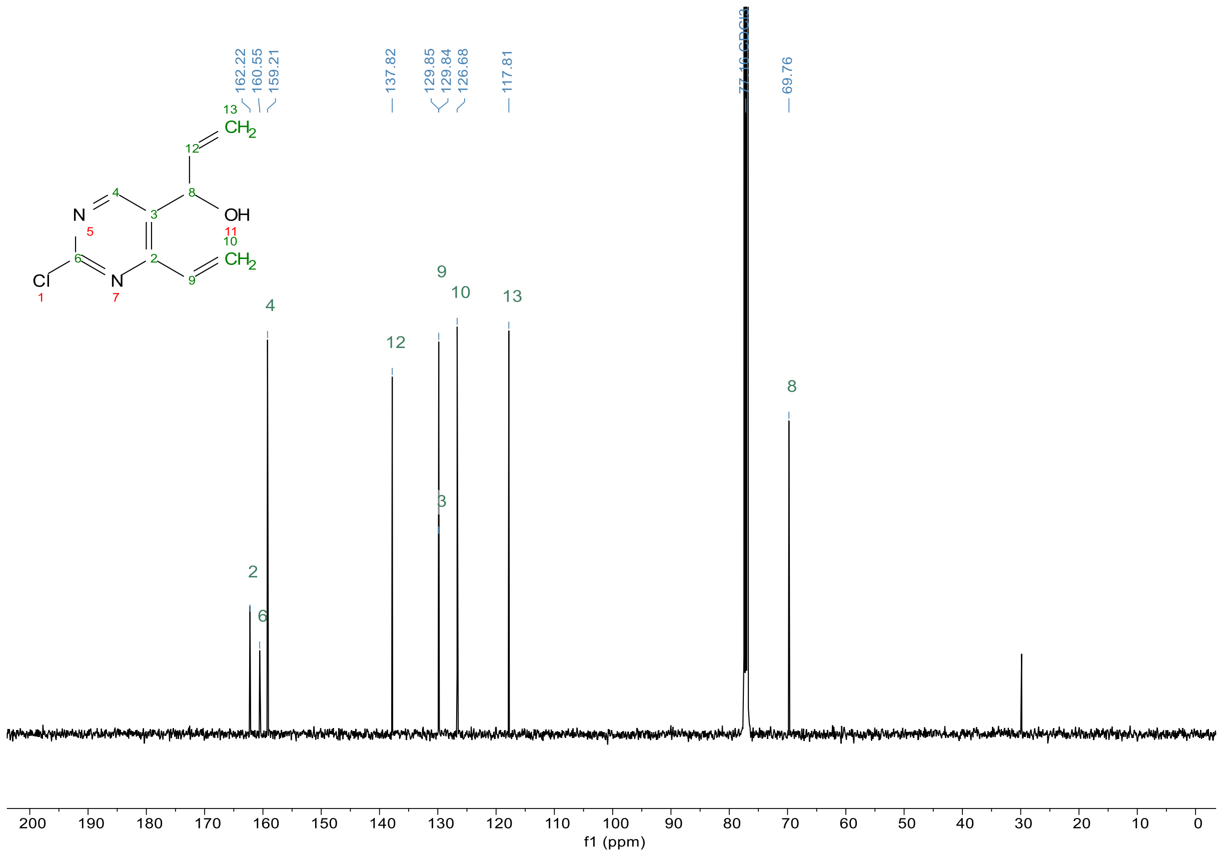

Fig.S131. ^{13}C NMR Spectrum of **S79** (Chloroform-d, 298 K).

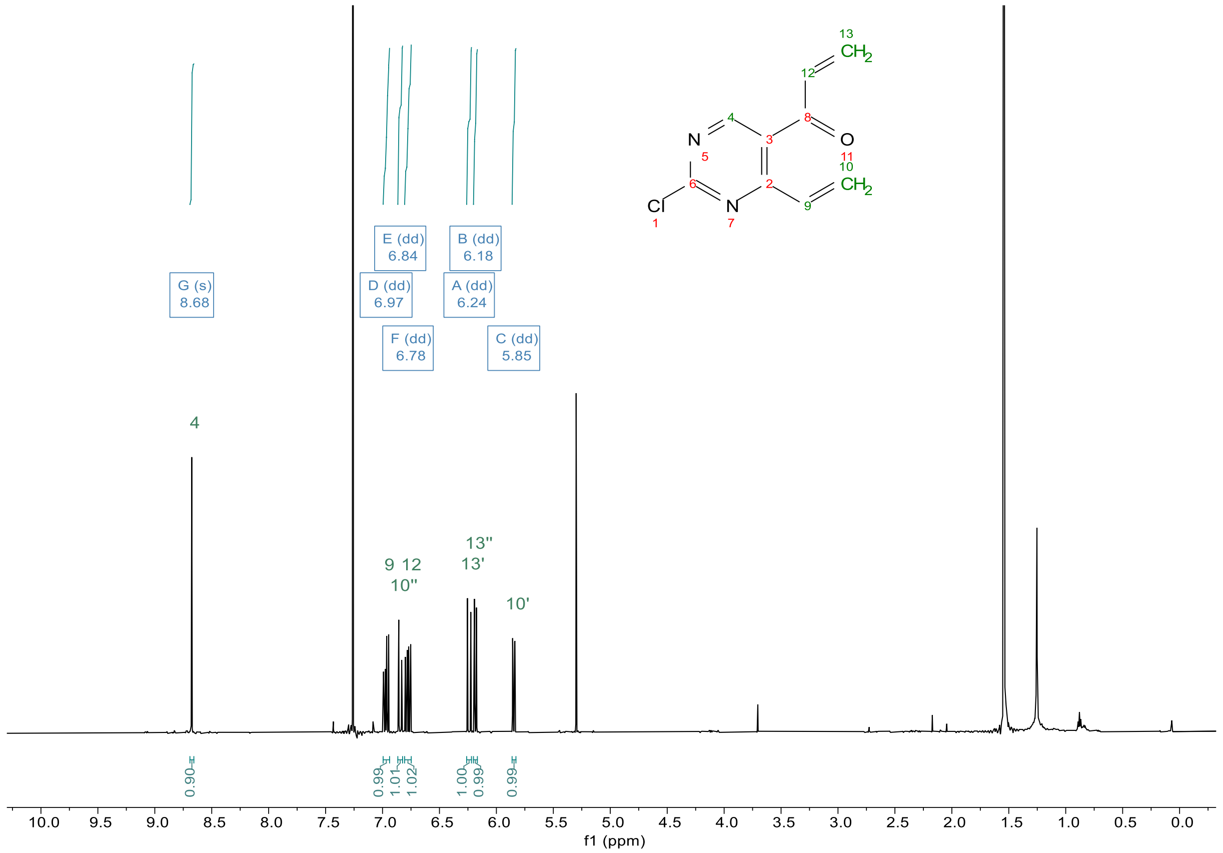

Fig.S132. ^{1}H NMR Spectrum of **S80** (Chloroform-d, 298 K).

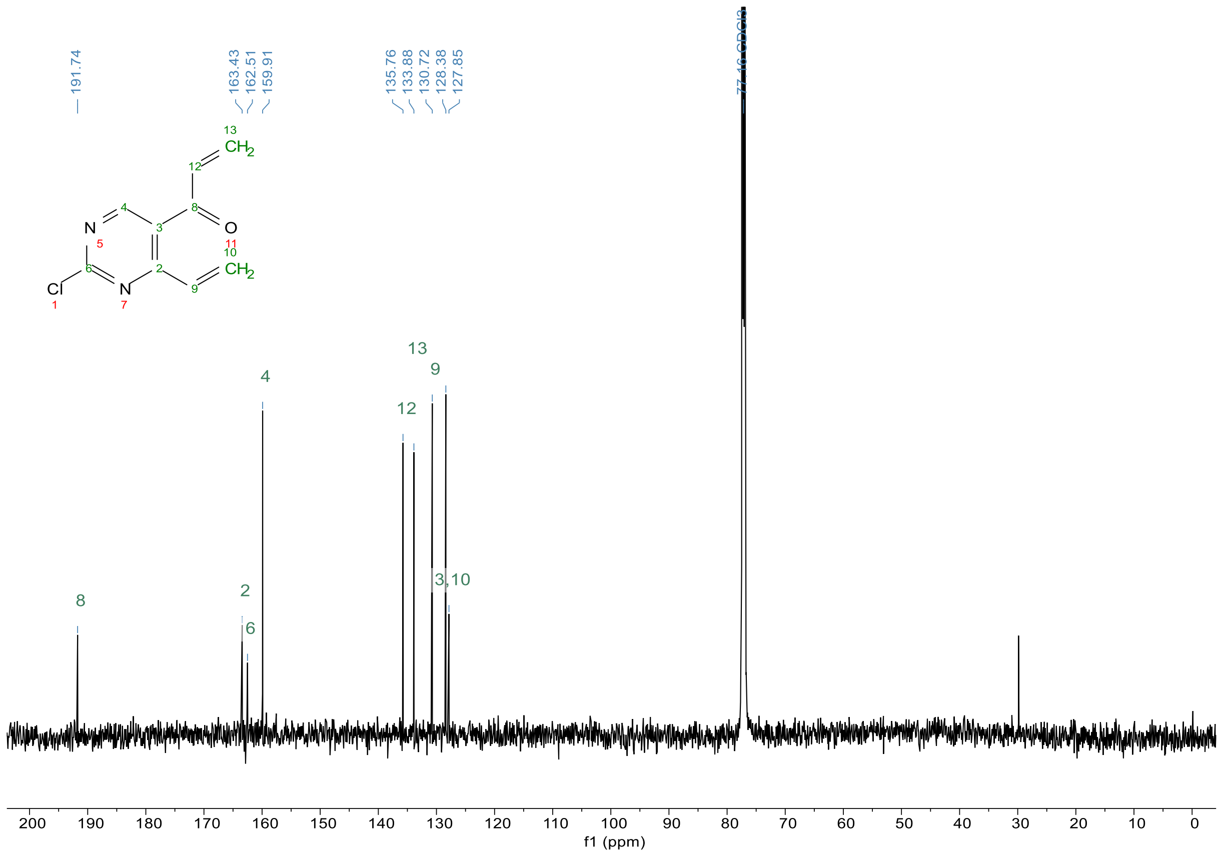

Fig.S133. ^{13}C NMR Spectrum of **S80** (Chloroform-d, 298 K).

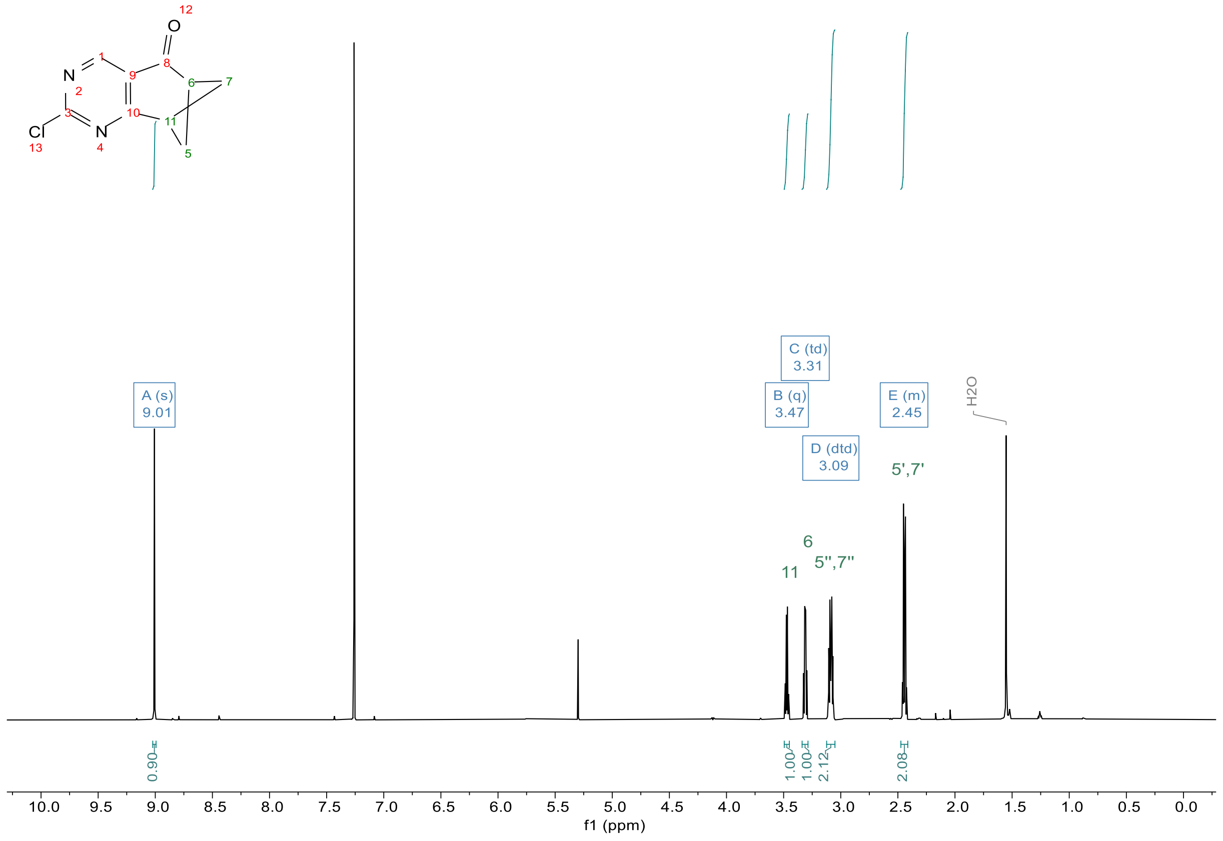

Fig.S134. ^{1}H NMR Spectrum of **28** (Chloroform-d, 298 K).

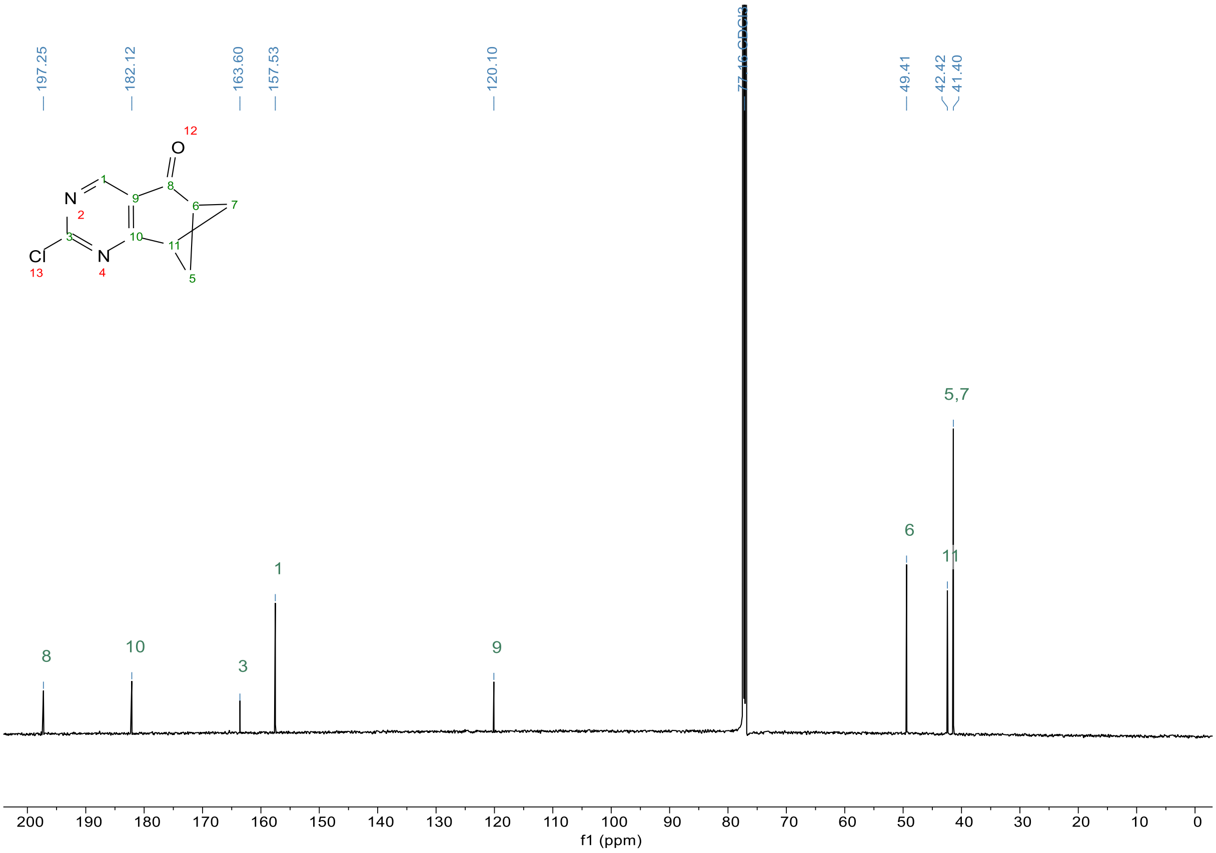

Fig.S135. ^{13}C NMR Spectrum of **28** (Chloroform-d, 298 K).

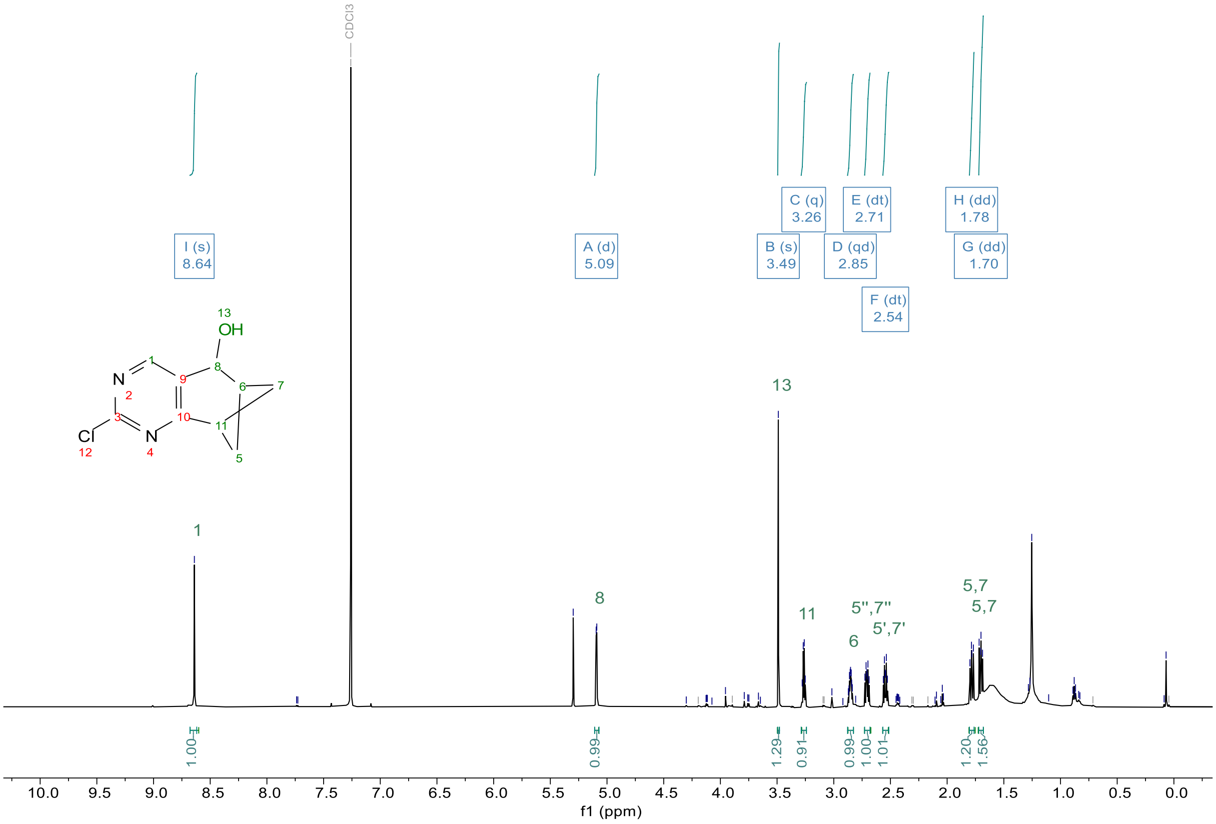

Fig.S136. ^{1}H NMR Spectrum of **31** (Chloroform-d, 298 K).

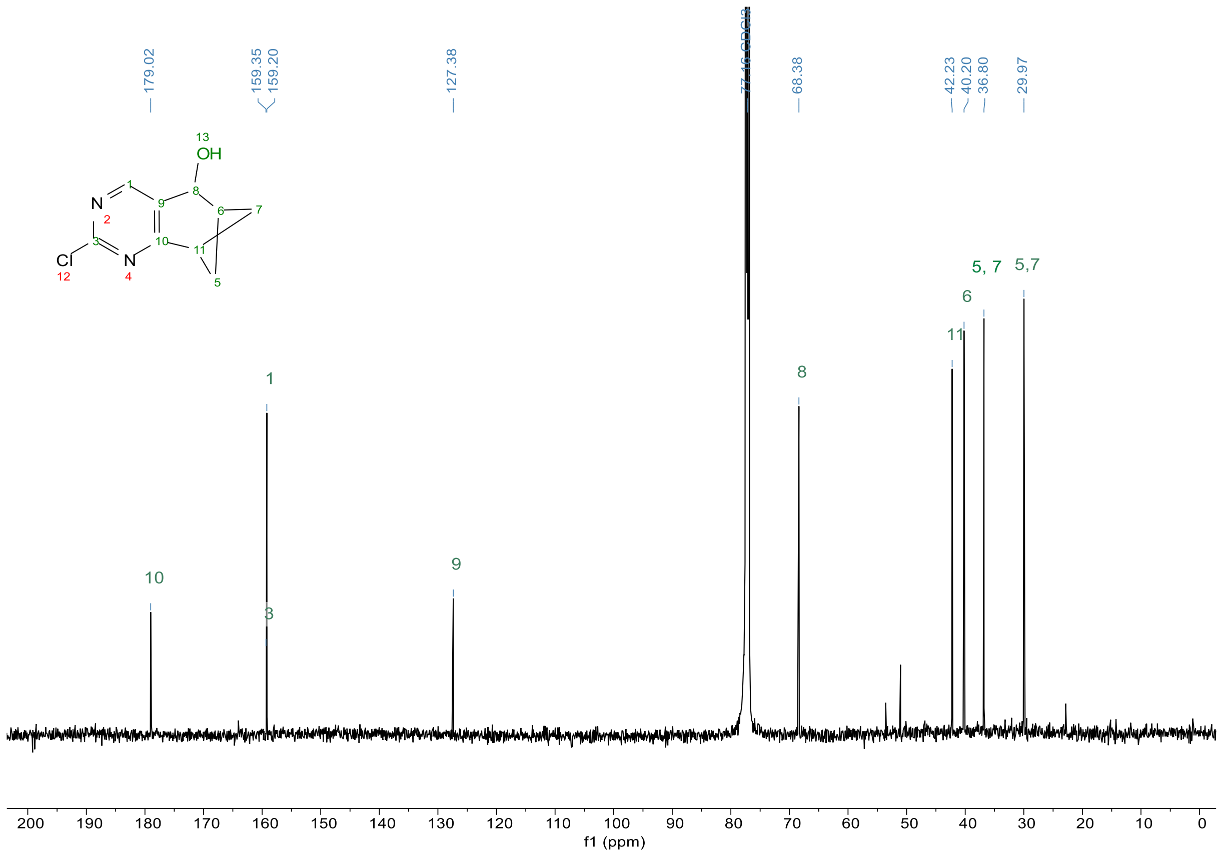

Fig.S137. ^{13}C NMR Spectrum of **31** (Chloroform-d, 298 K).

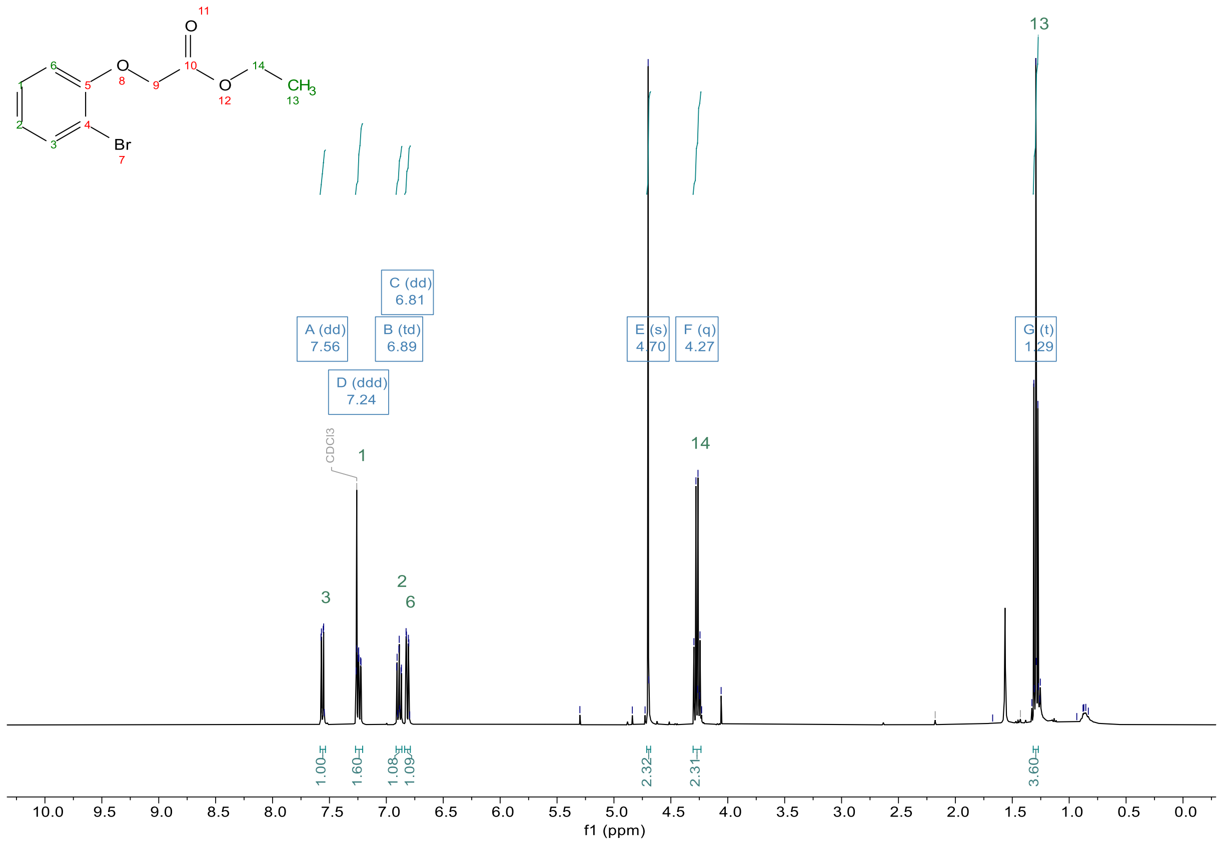

Fig.S138. ^{1}H NMR Spectrum of **S83** (Chloroform-d, 298 K).

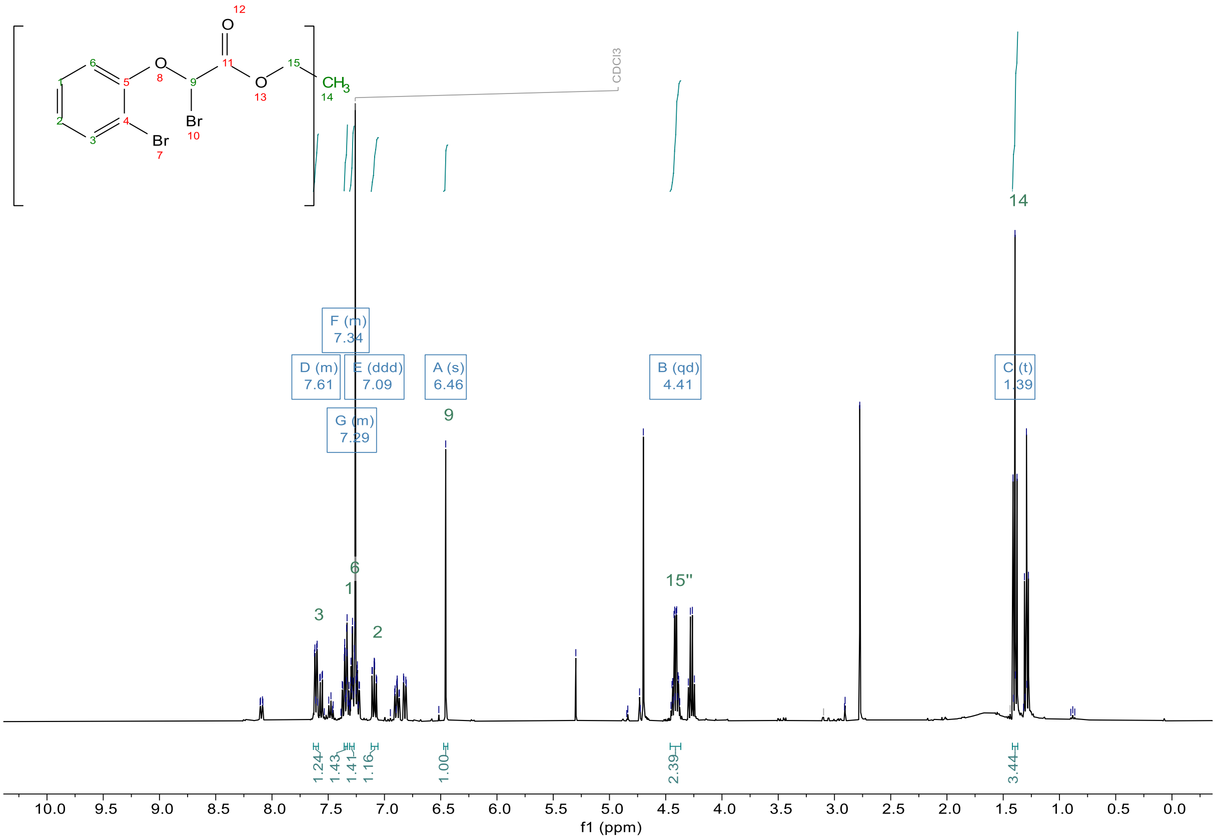

Fig.S139. Crude ^{1}H NMR Spectrum of **S84** (Chloroform-d, 298 K).

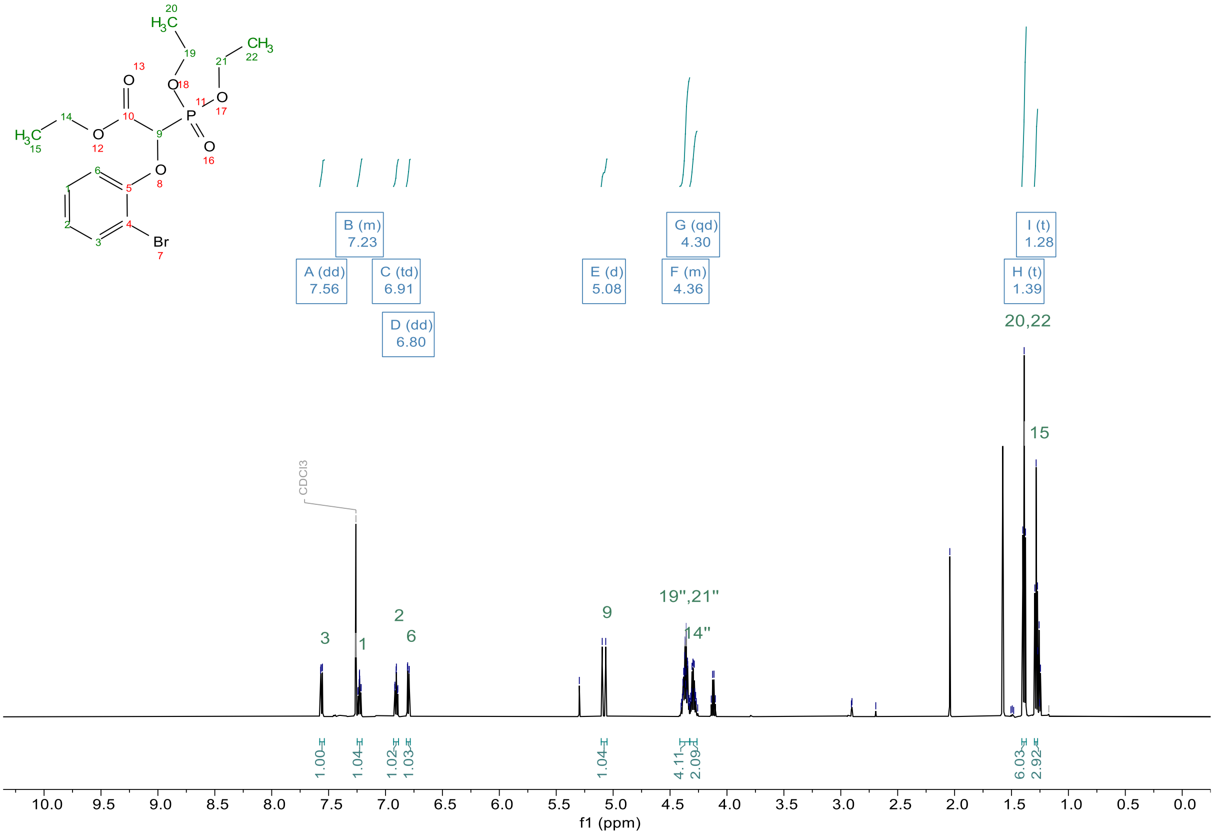

Fig.S140. ^{1}H NMR Spectrum of **S85** (Chloroform-d, 298 K).

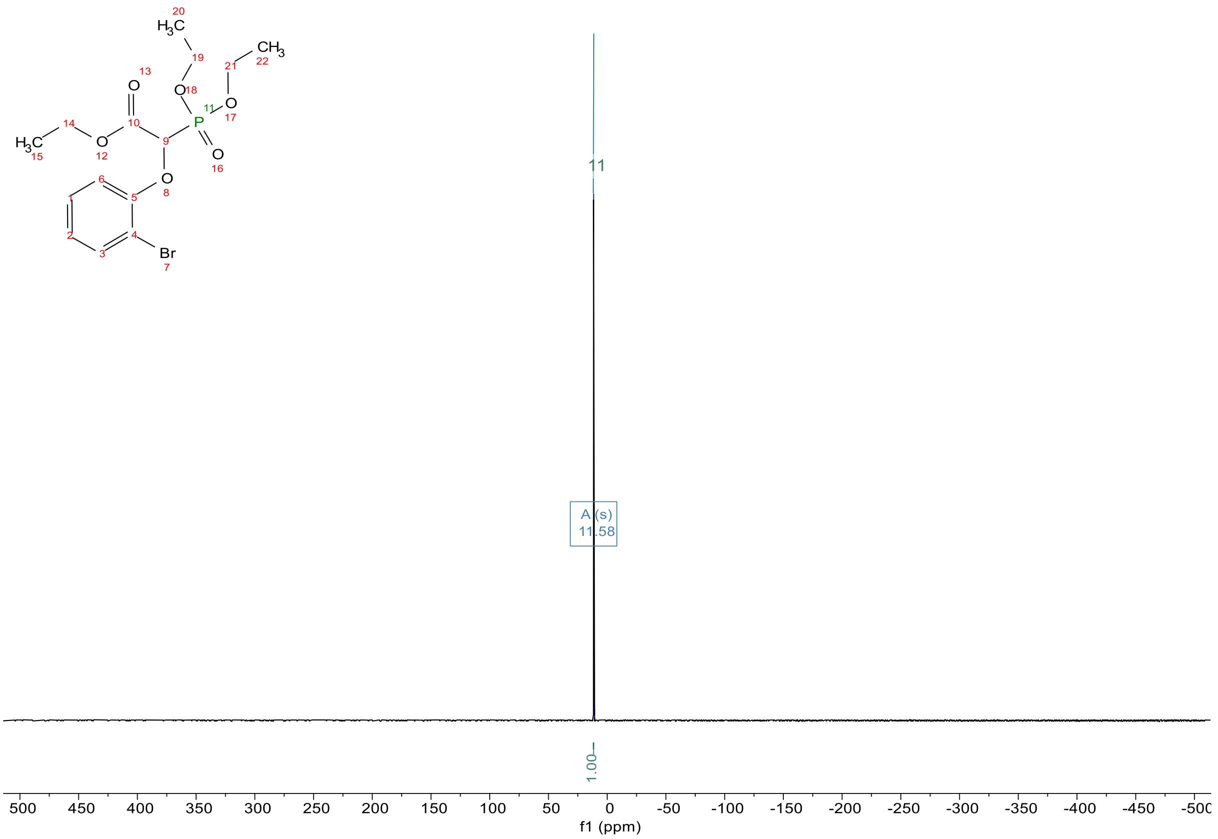

Fig.S141. ^{31}P NMR Spectrum of **S85** (Chloroform-d, 298 K).

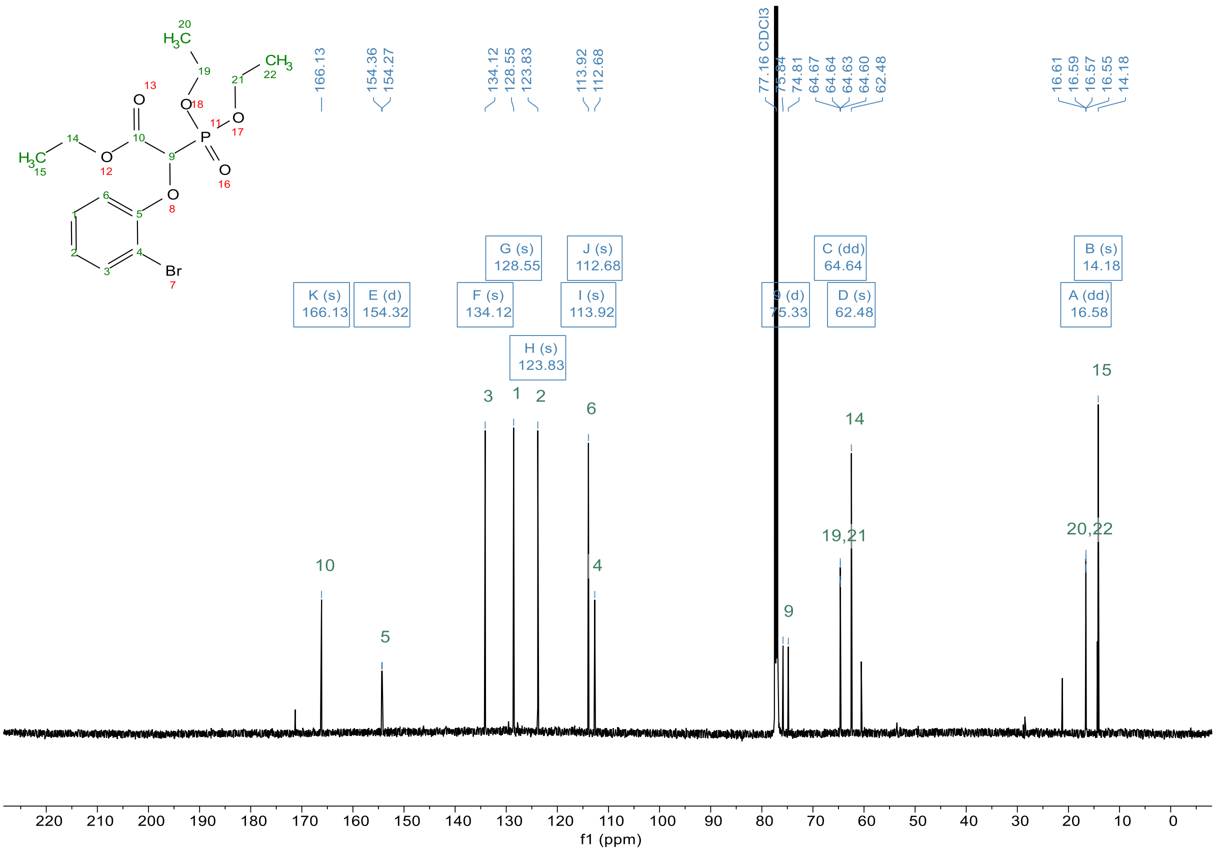

Fig.S142. ^{13}C NMR Spectrum of **S85** (Chloroform-d, 298 K).

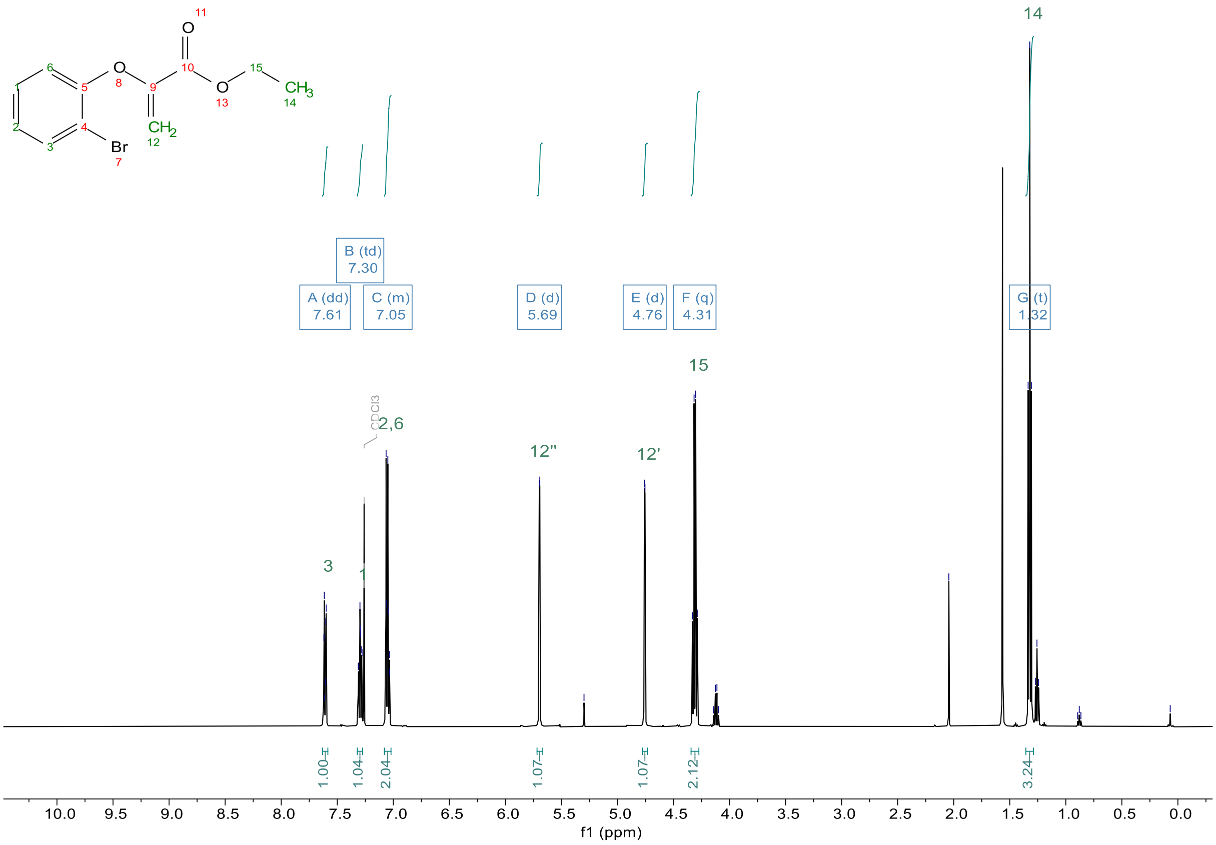

Fig.S143. ^{1}H NMR Spectrum of **S86** (Chloroform-d, 298 K).

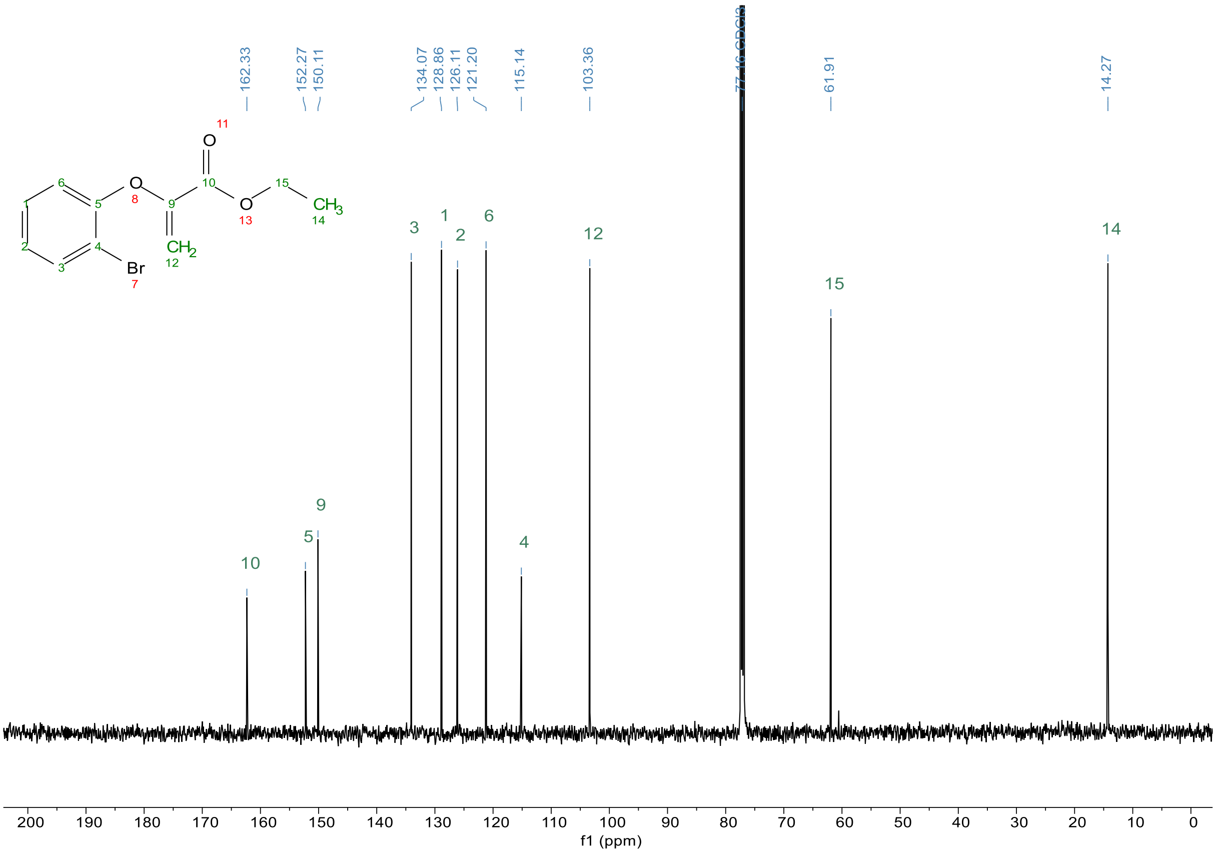

Fig.S144. ^{13}C NMR Spectrum of **S86** (Chloroform-d, 298 K).

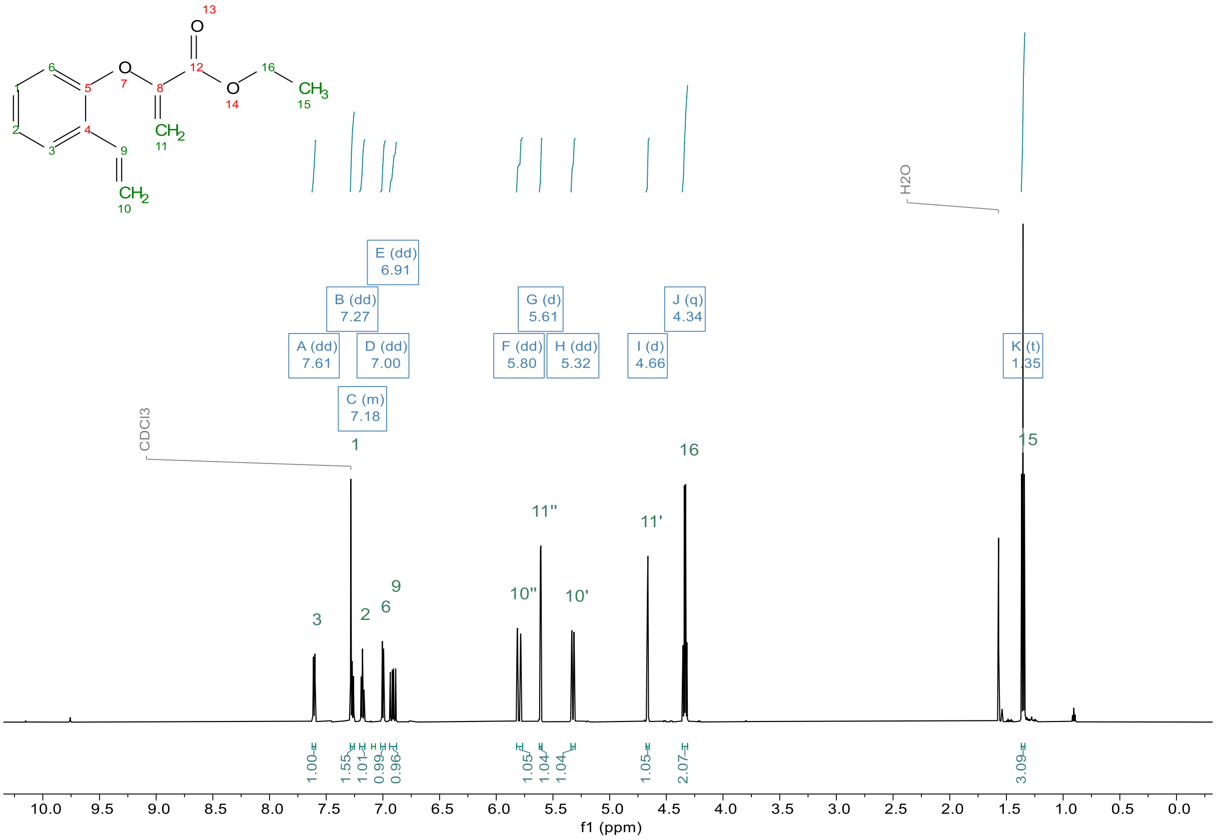

Fig.S145. ^{1}H NMR Spectrum of **S87** (Chloroform-d, 298 K).

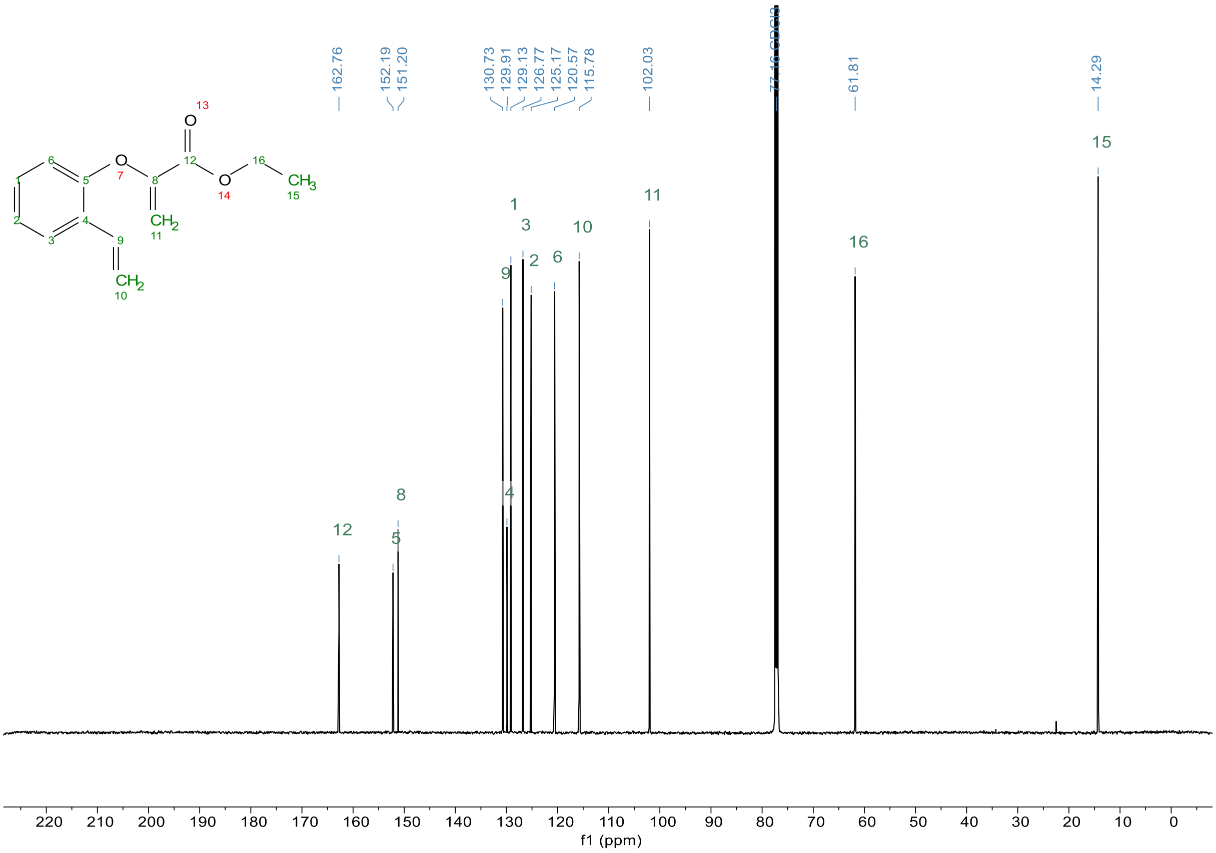

Fig.S146. ^{13}C NMR Spectrum of **S87** (Chloroform-d, 298 K).

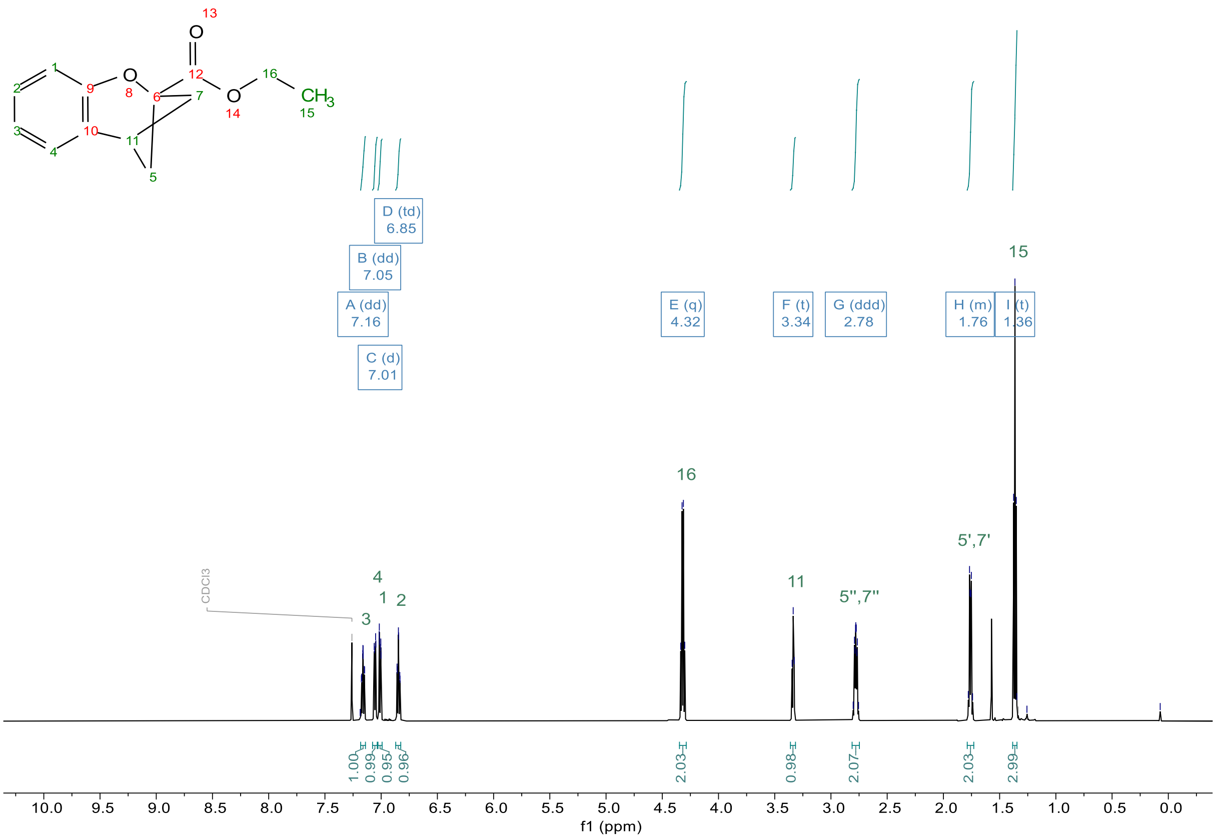

Fig.S147. ^{1}H NMR Spectrum of **29** (Chloroform-d, 298 K).

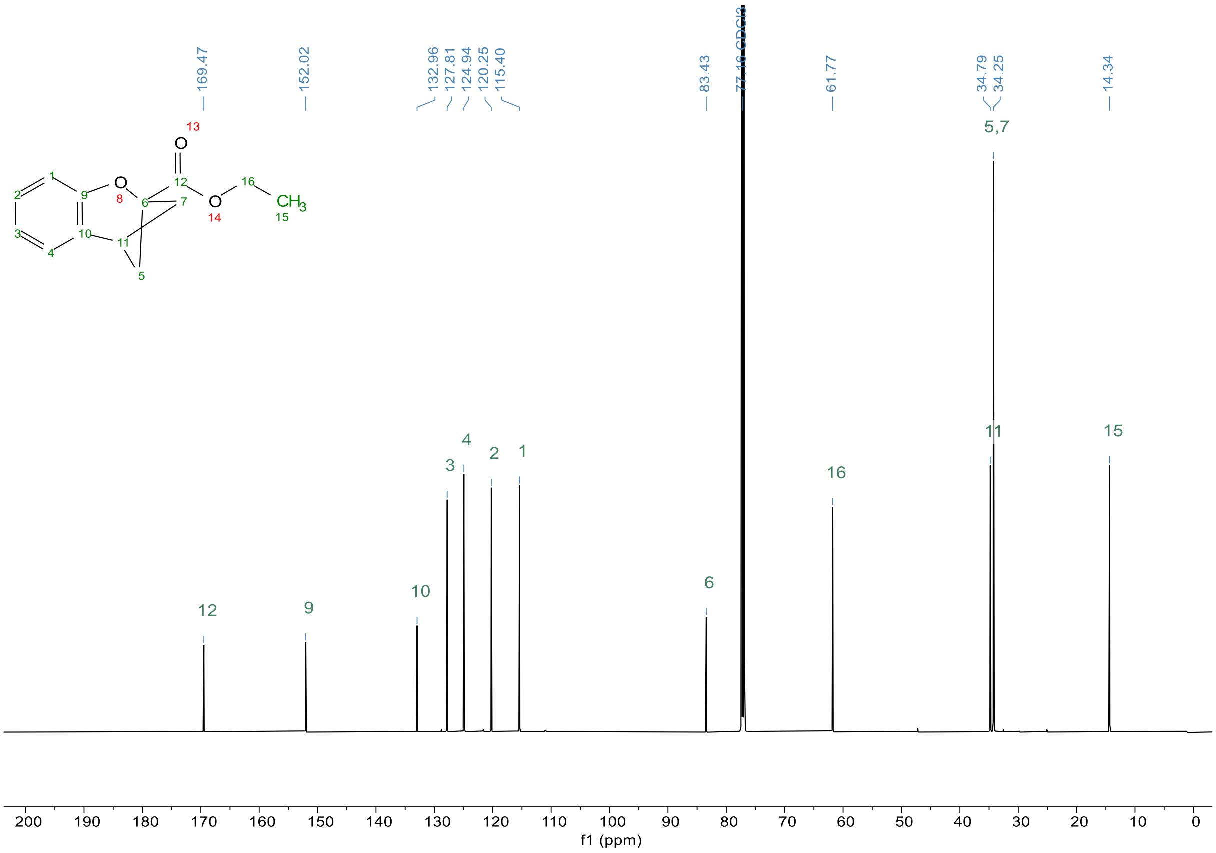

Fig.S148. ^{13}C NMR Spectrum of **29** (Chloroform-d, 298 K).

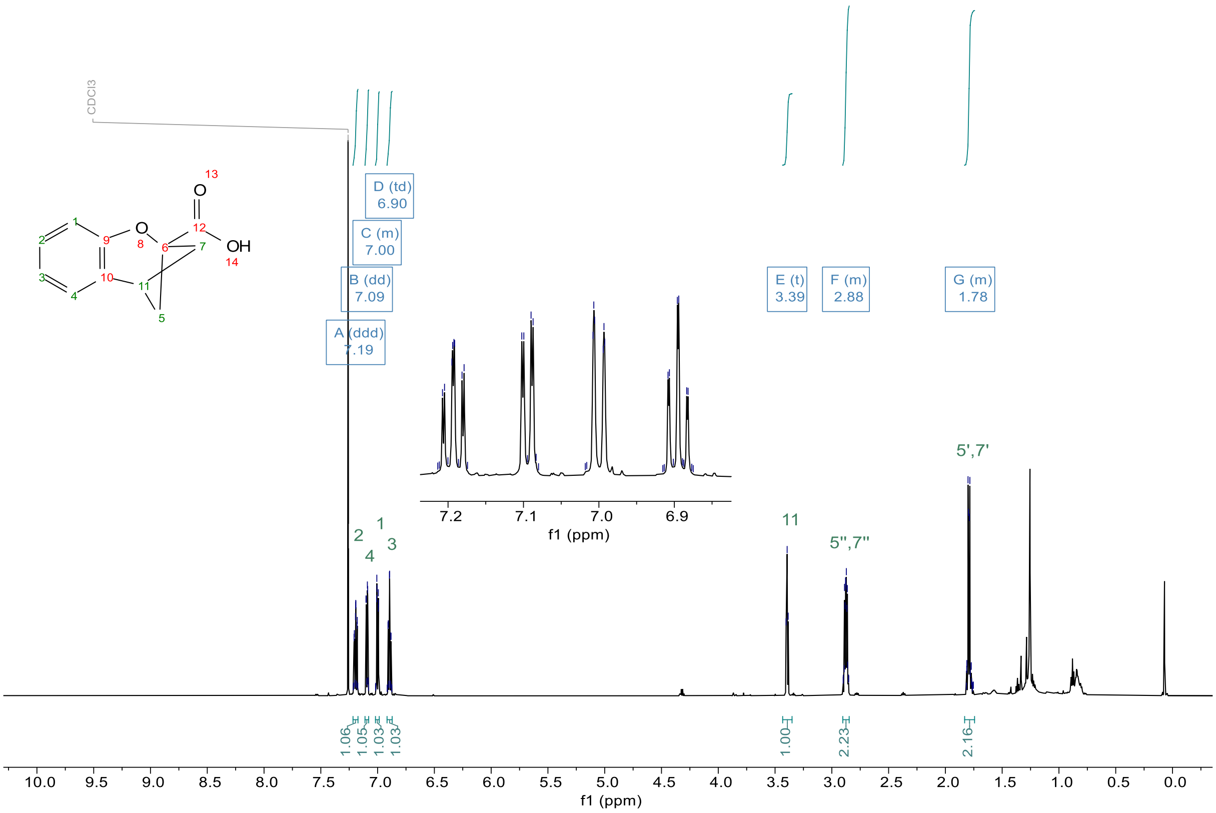

Fig.S149. ^{1}H NMR Spectrum of **47** (Chloroform-d, 298 K).

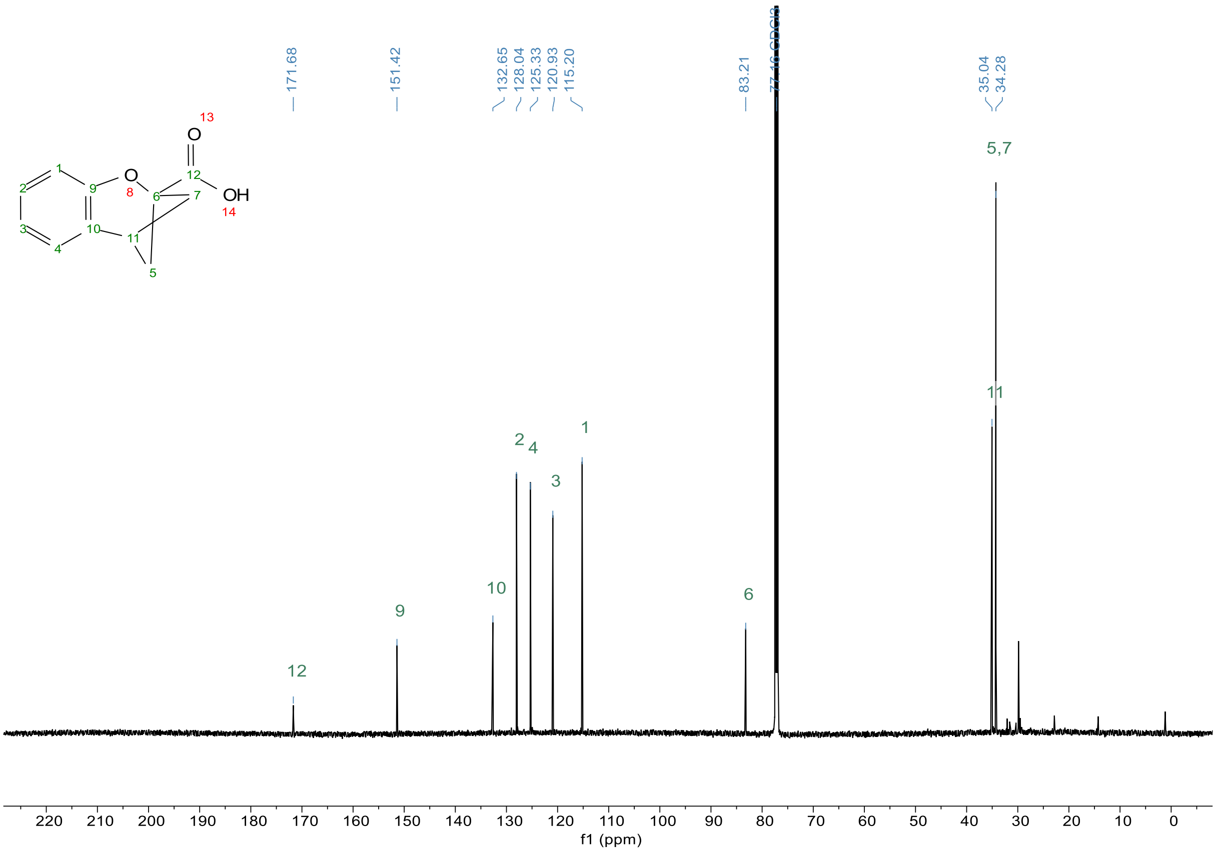

Fig.S150. ^{13}C NMR Spectrum of **47** (Chloroform-d, 298 K).

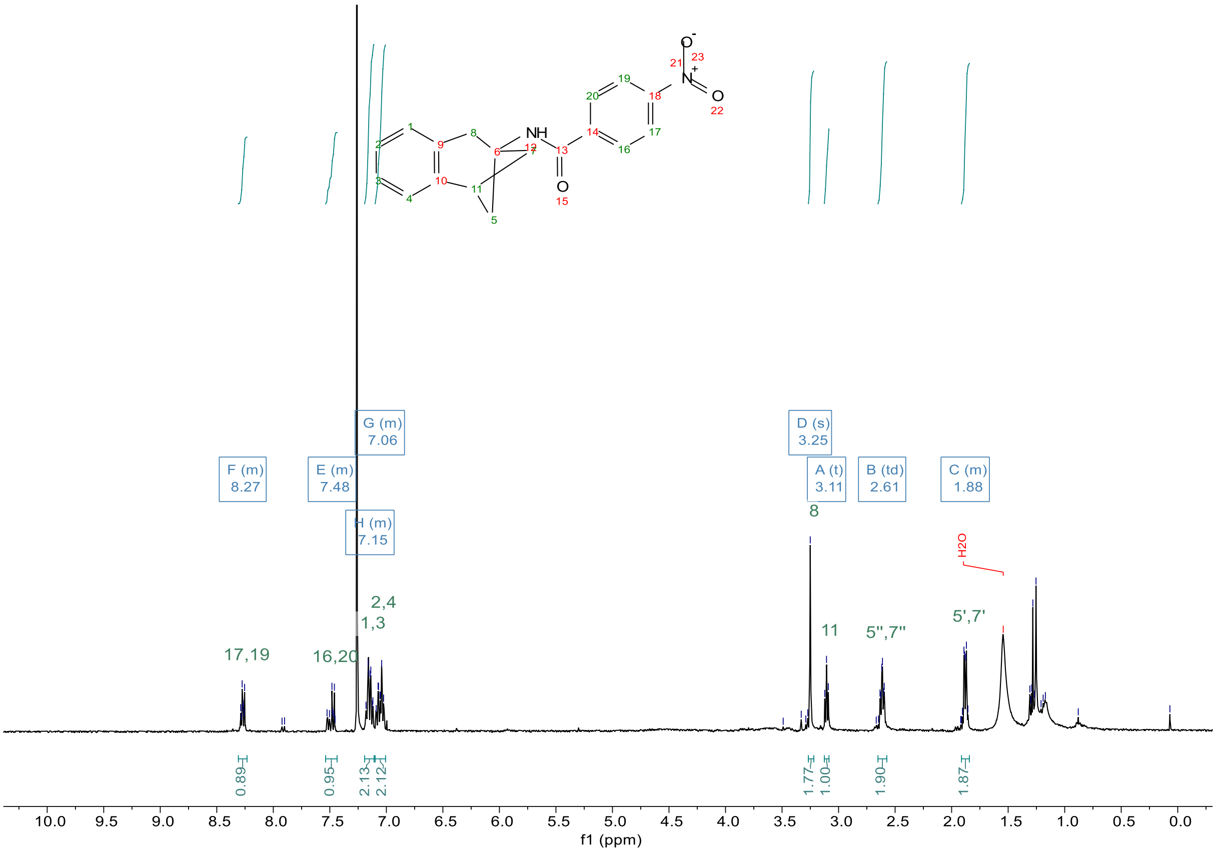

Fig.S151. ^{1}H NMR Spectrum of **S89** (Chloroform-d, 298 K).

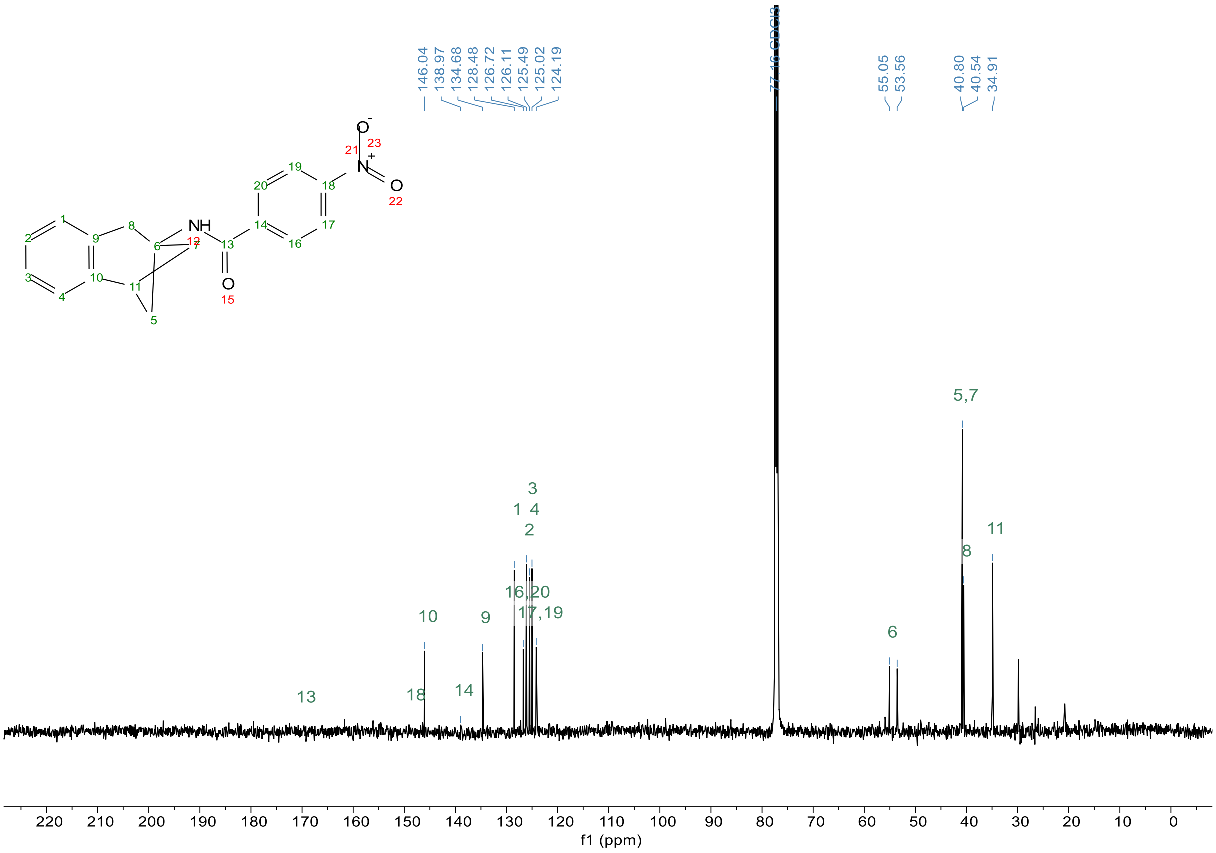

Fig.S152. ^{13}C NMR Spectrum of **S89** (Chloroform-d, 298 K).

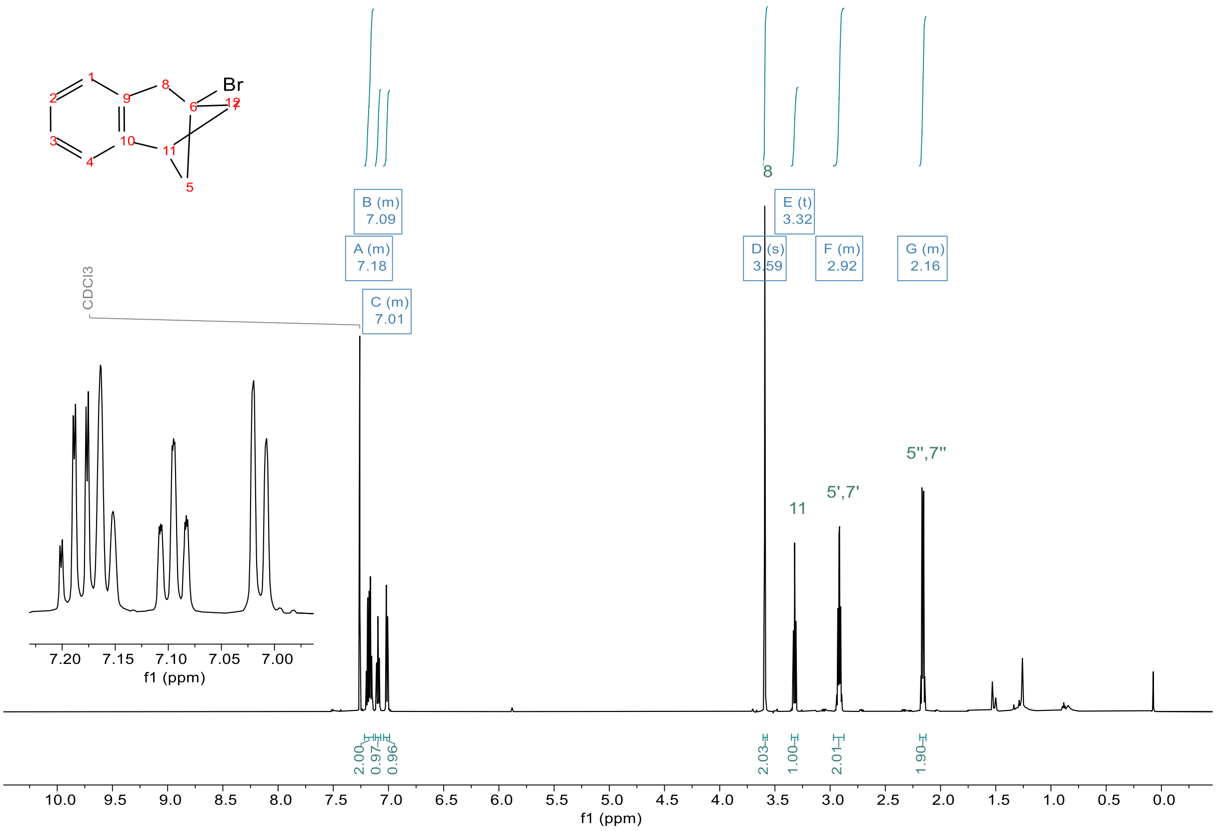

Fig.S153. ^{1}H NMR Spectrum of **38** (Chloroform-d, 298 K).

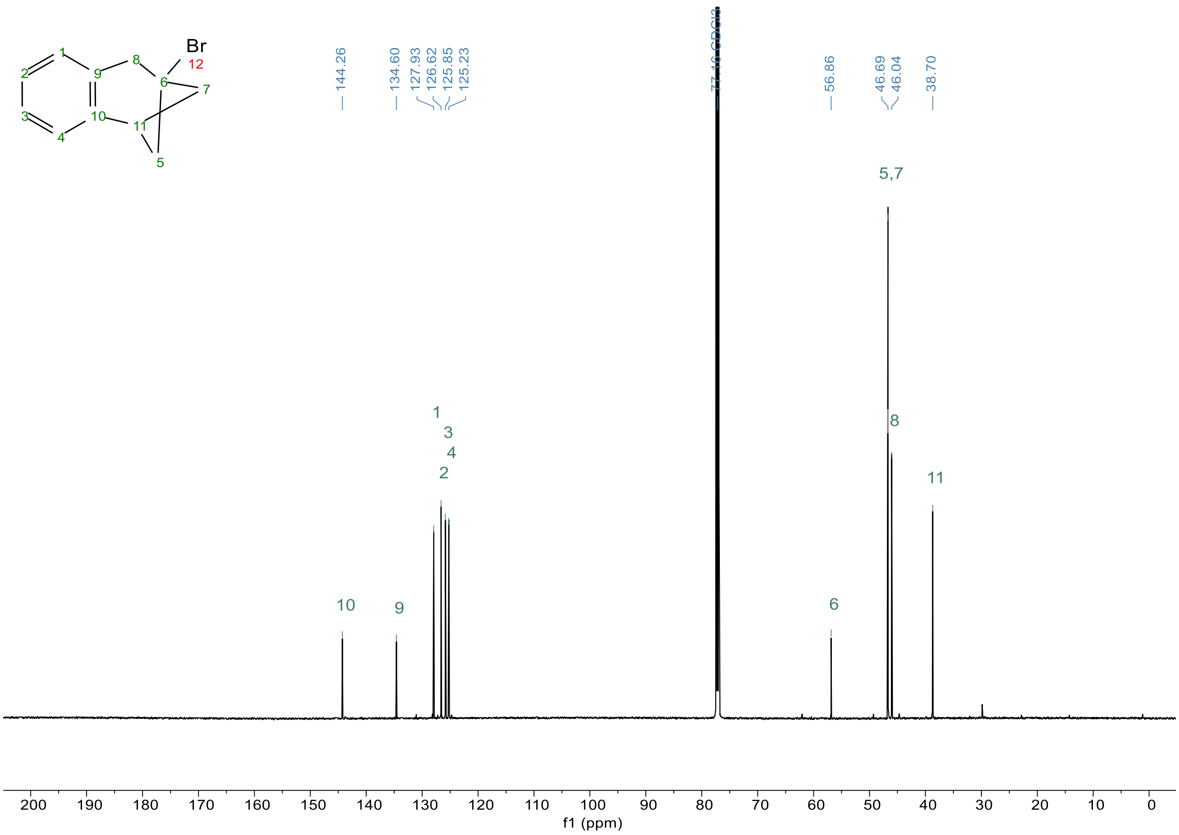

Fig.S154. ^{13}C NMR Spectrum of **38** (Chloroform-d, 298 K).

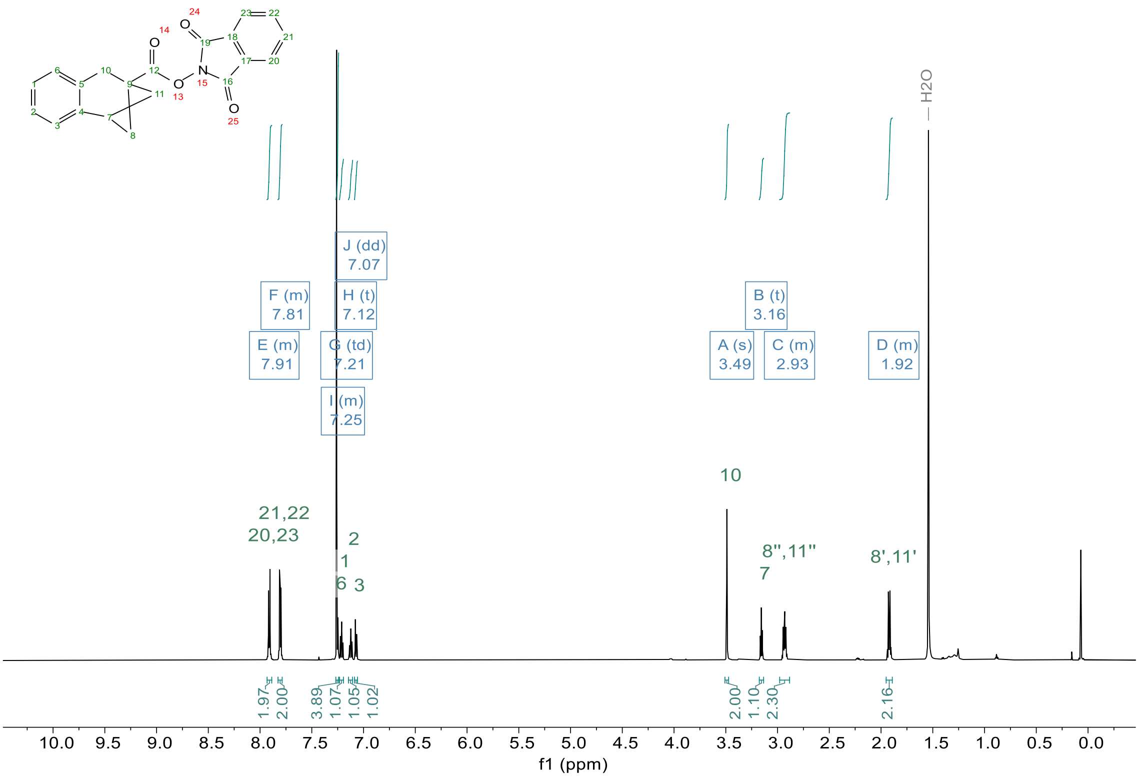

Fig.S155. ^{1}H NMR Spectrum of **39** (Chloroform-d, 298 K).

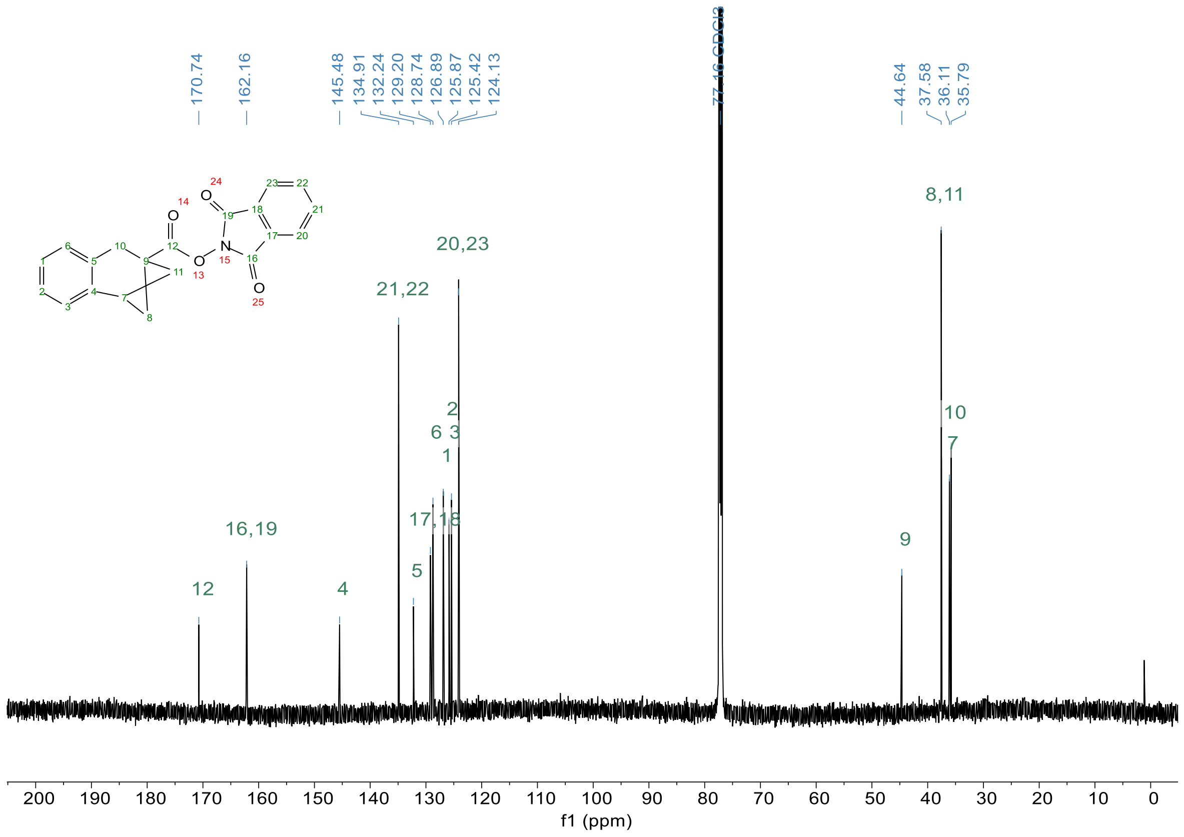

Fig.S156. ^{13}C NMR Spectrum of **39** (Chloroform-d, 298 K).

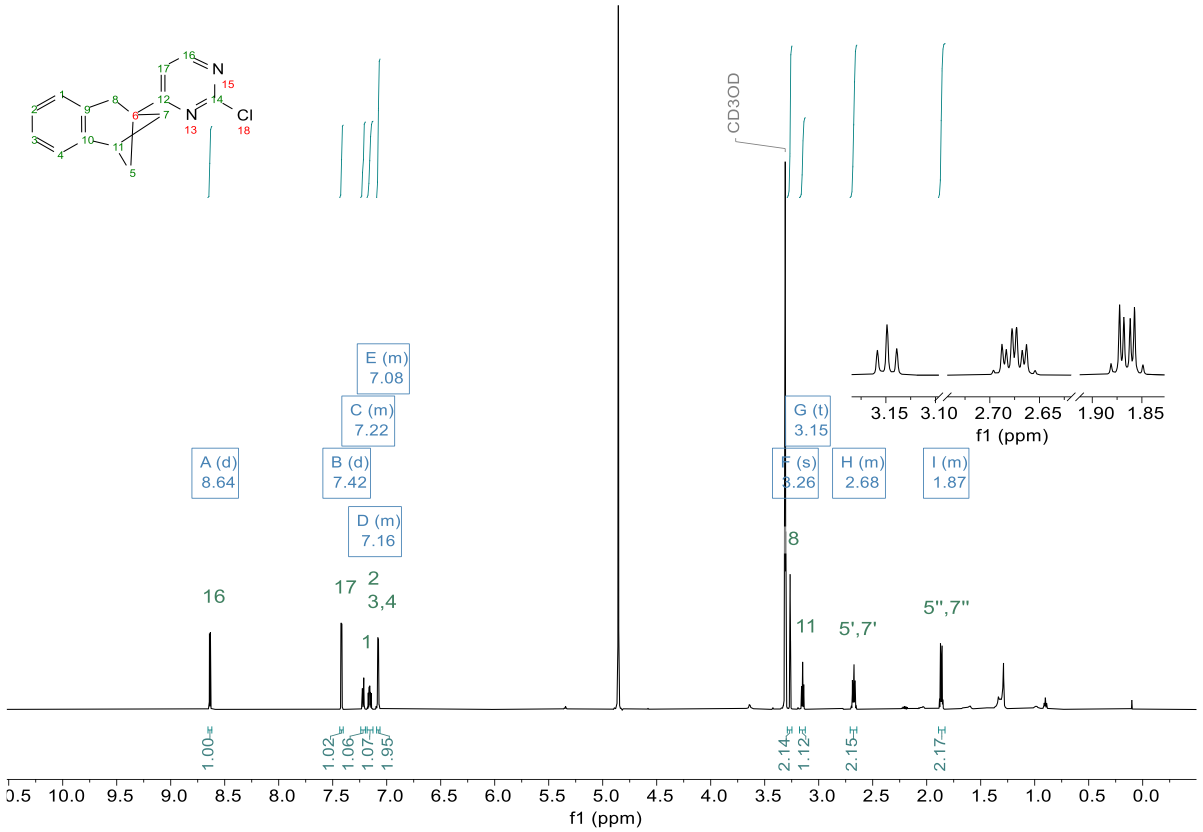

Fig.S157. ^{1}H NMR Spectrum of **40** (MeOD-d$_4$, 298 K).

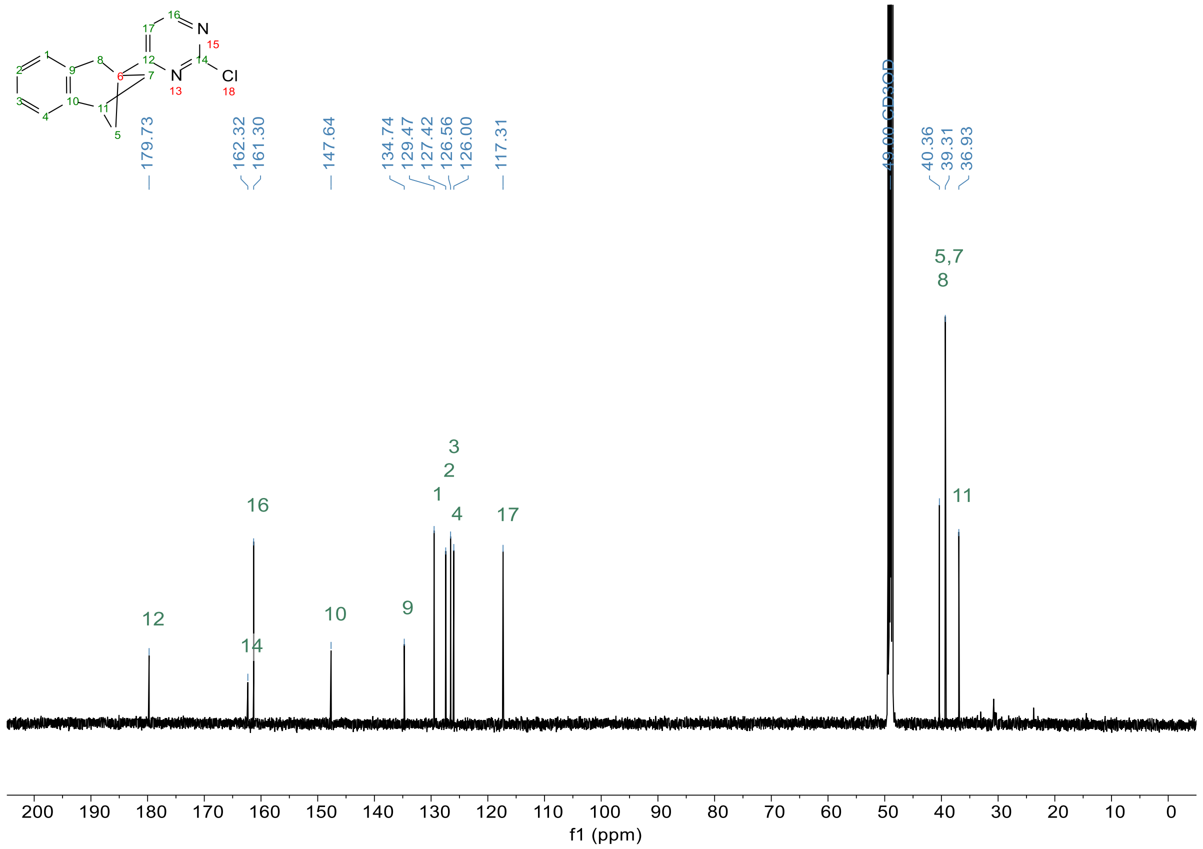

Fig.S158. ^{13}C NMR Spectrum of **40** (MeOD-d$_4$, 298 K).

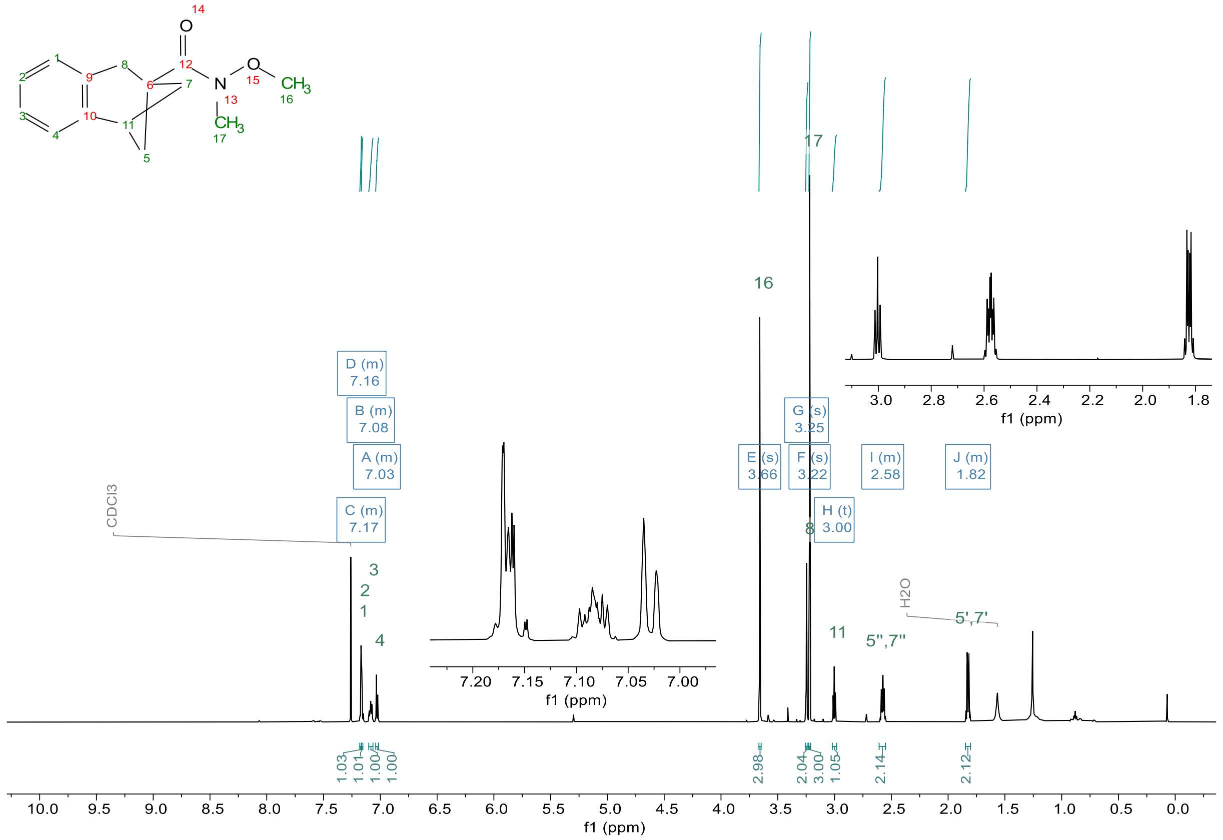

Fig.S159. ^{1}H NMR Spectrum of **41** (Chloroform-d, 298 K).

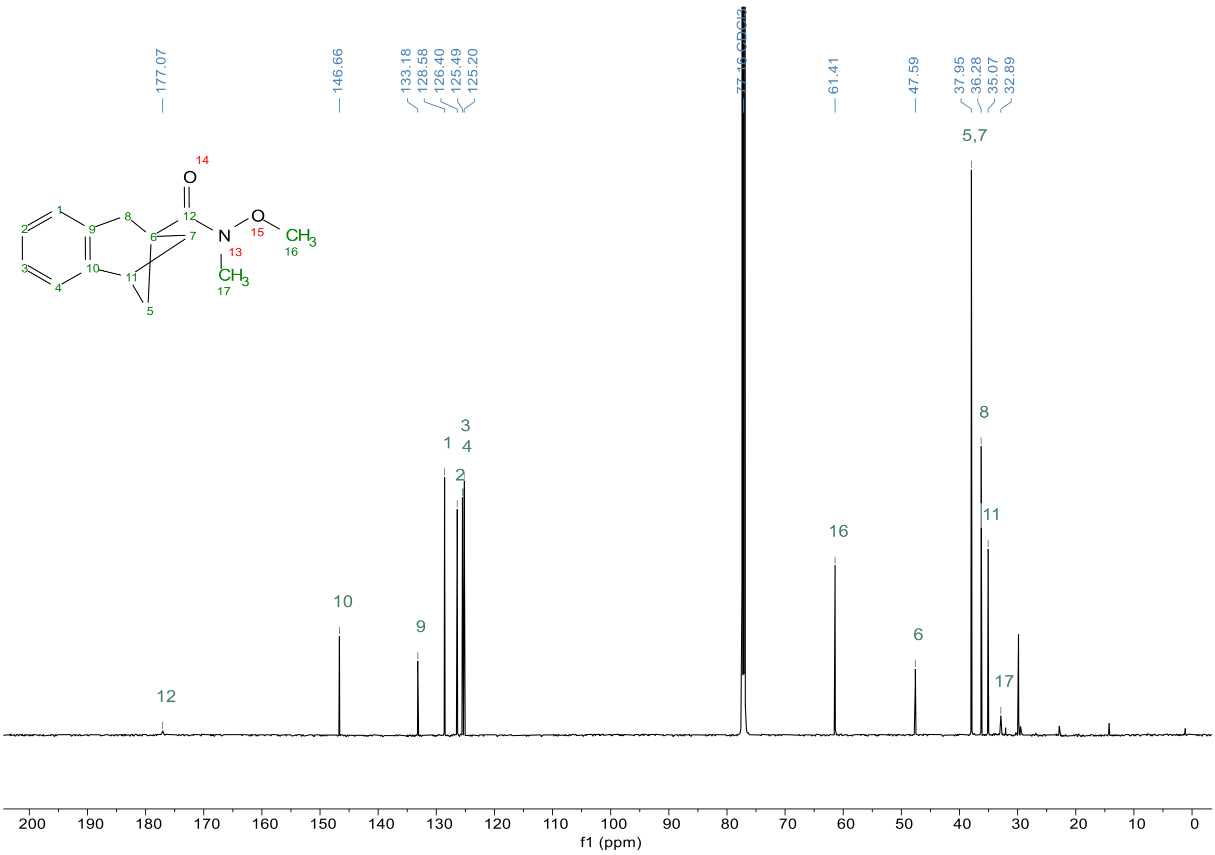

Fig.S160. ^{13}C NMR Spectrum of **41** (Chloroform-d, 298 K).

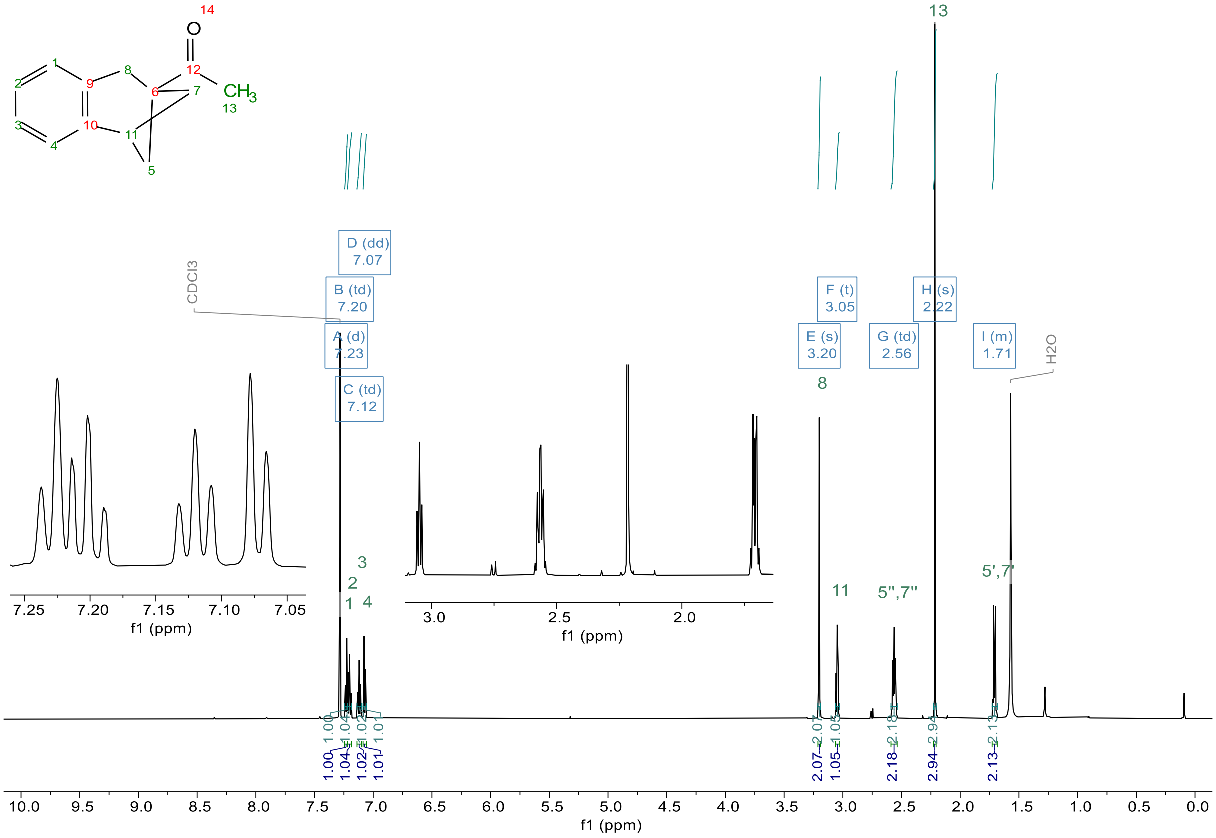

Fig.S161. ^{1}H NMR Spectrum of **42** (Chloroform-d, 298 K).

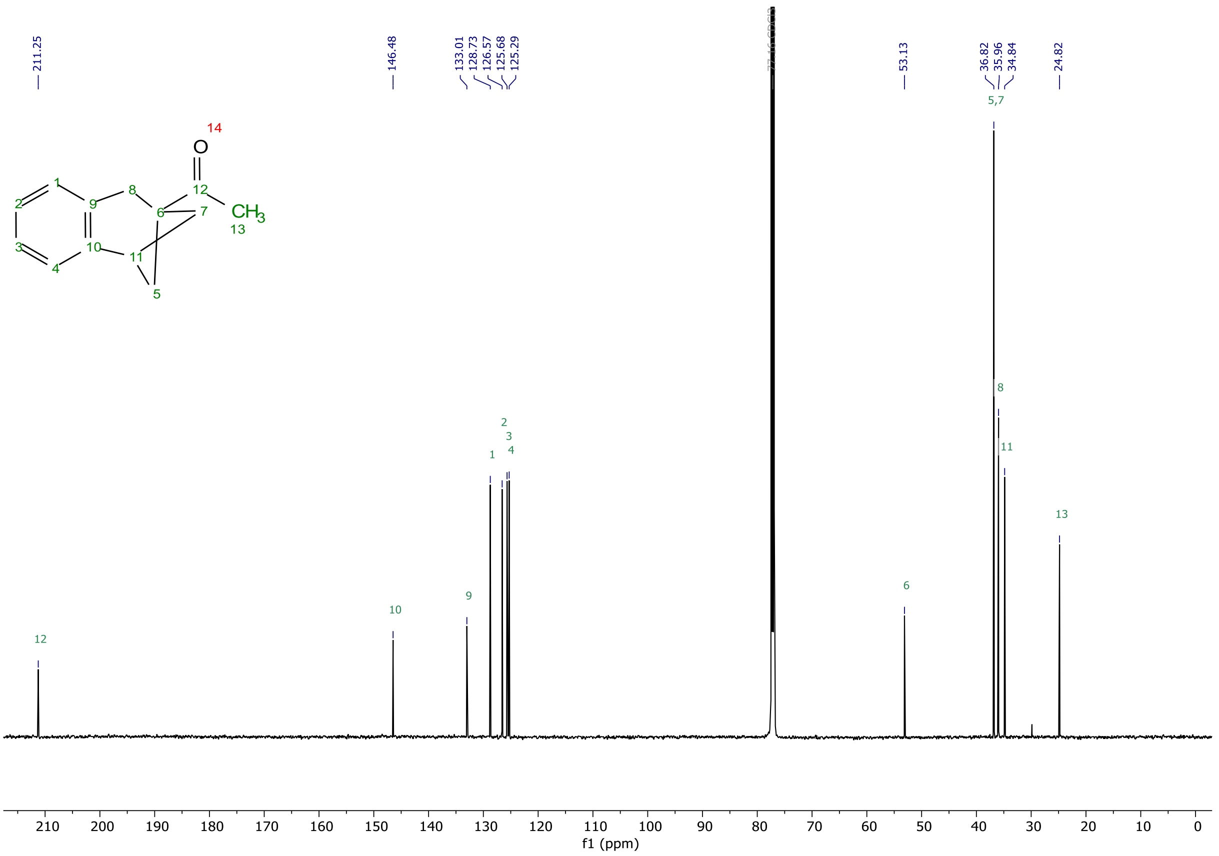

Fig.S162. ^{13}C NMR Spectrum of **42** (Chloroform-d, 298 K).

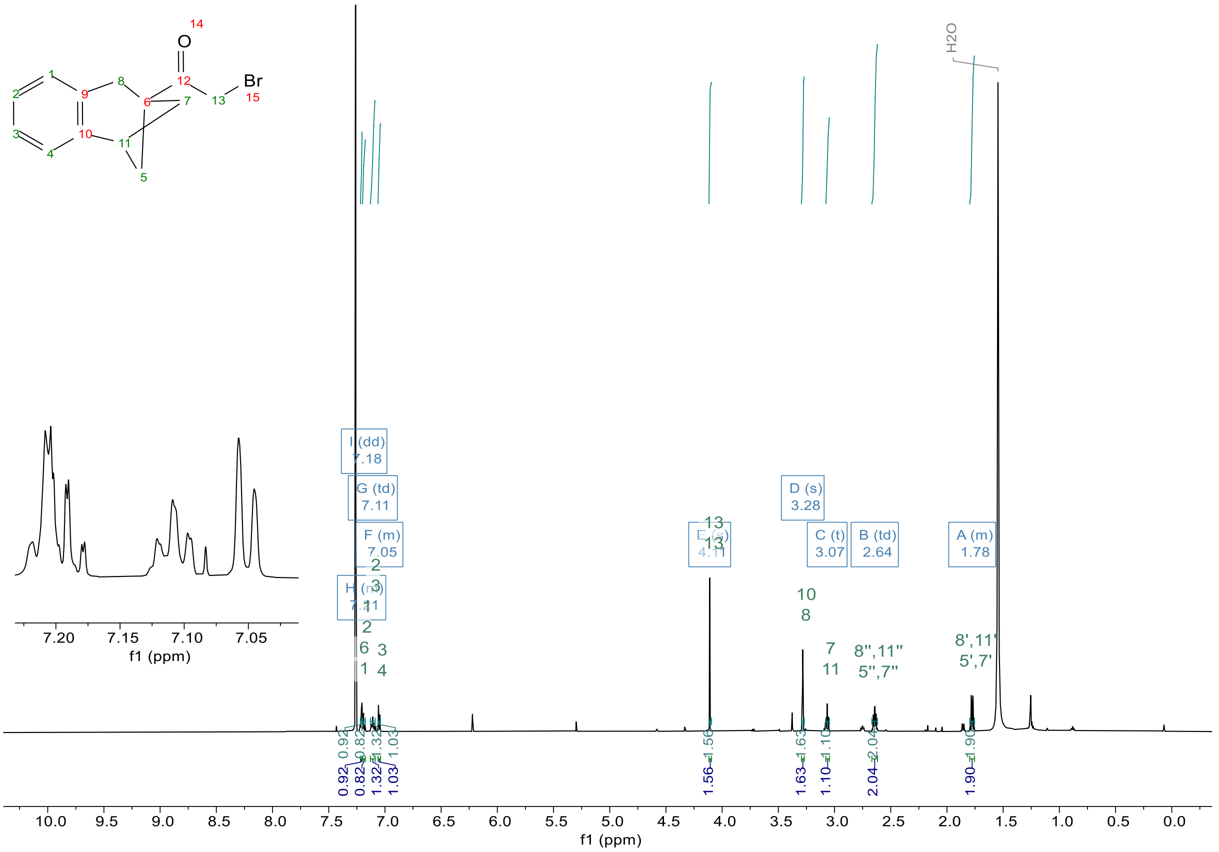

Fig.S163. ^{1}H NMR Spectrum of **43** (Chloroform-d, 298 K).

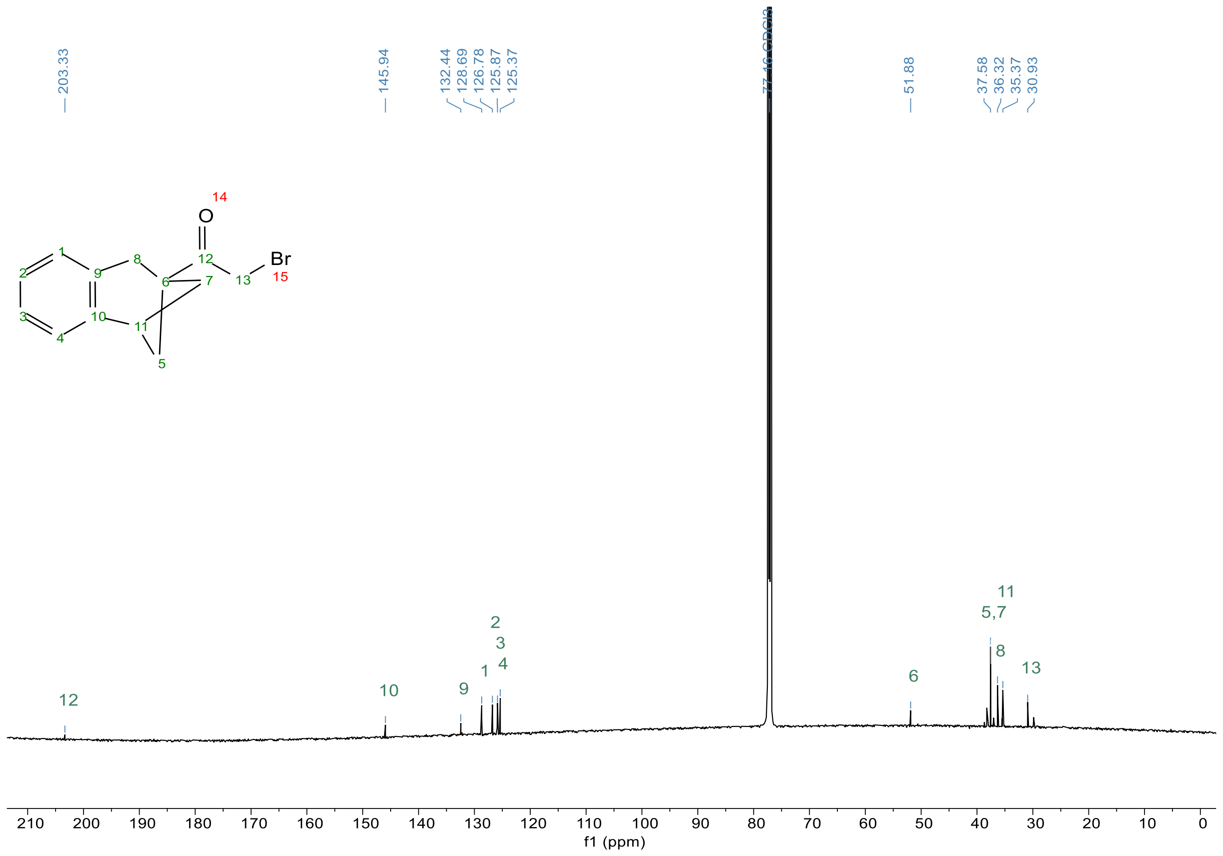

Fig.S164. ^{13}C NMR Spectrum of **43** (Chloroform-d, 298 K).

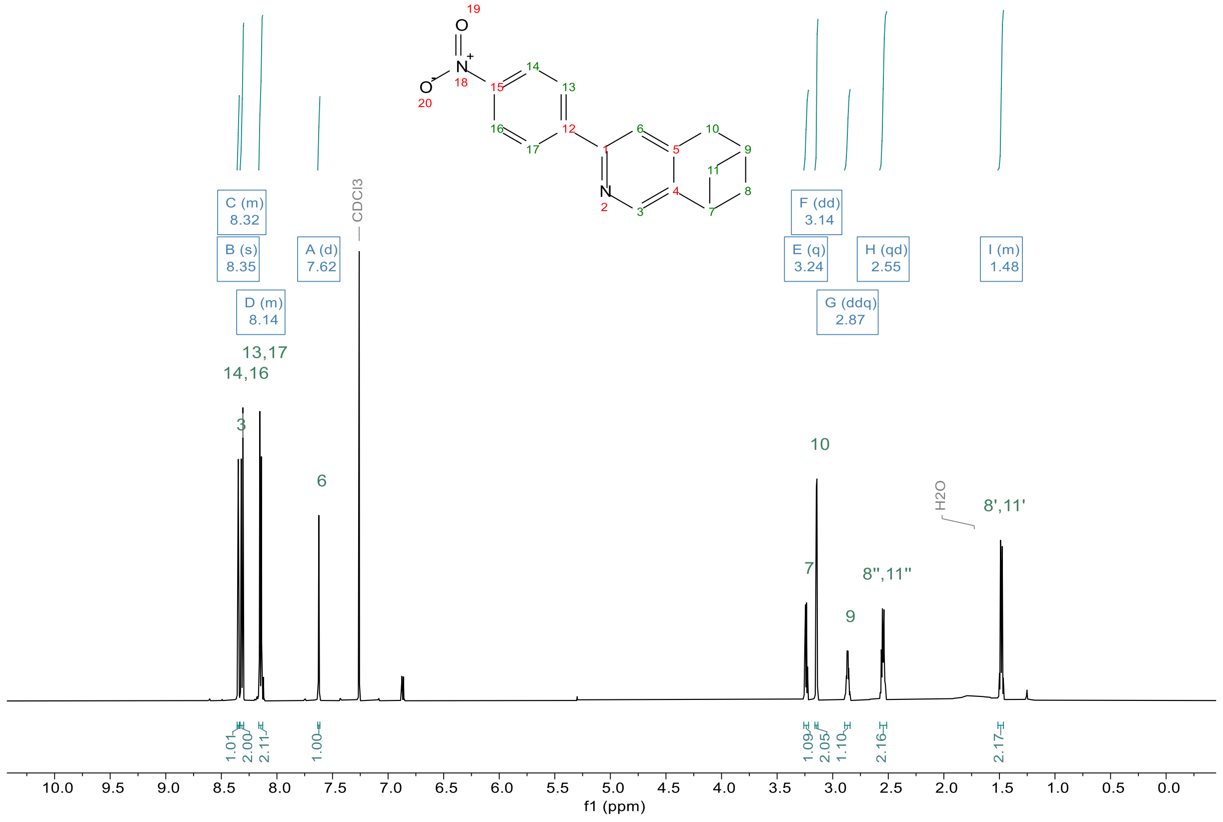

Fig.S165. ^{1}H NMR Spectrum of **35** (Chloroform-d, 298 K).

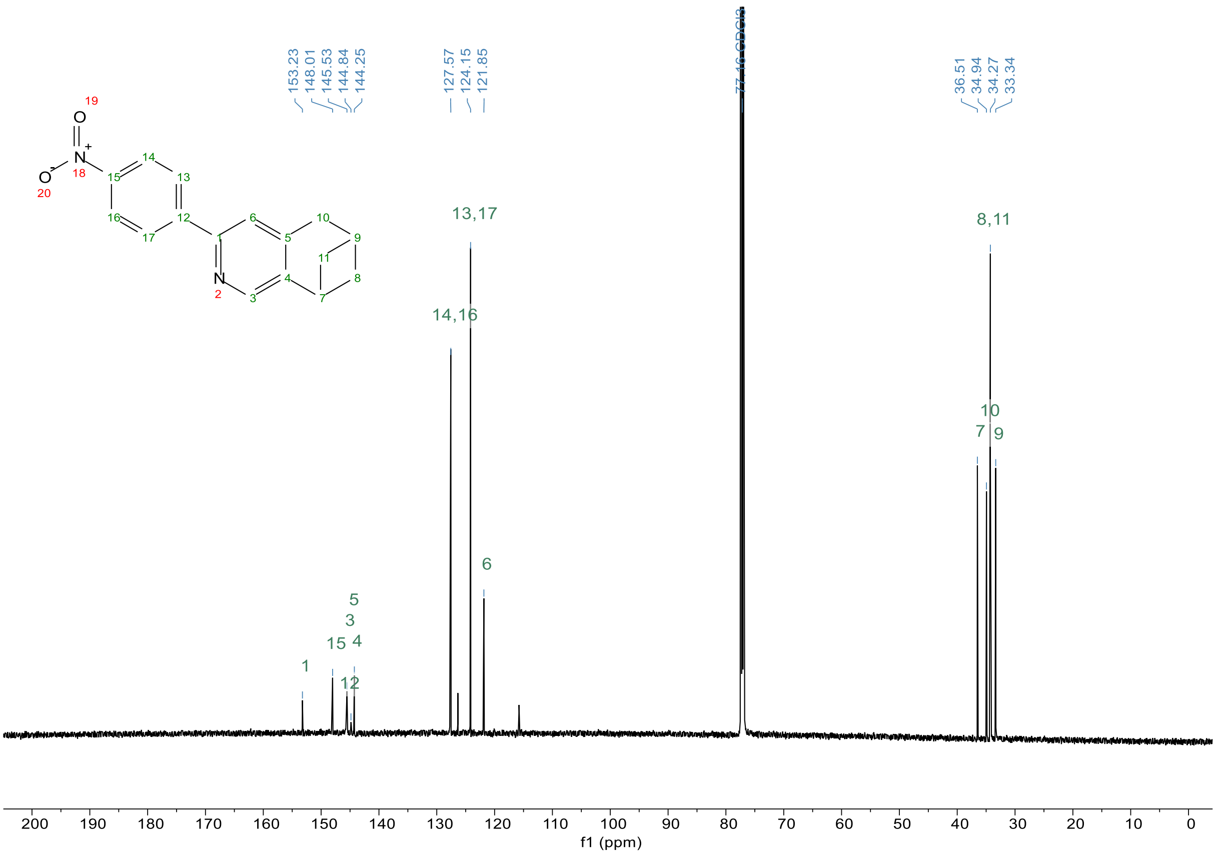

Fig.S166. ^{13}C NMR Spectrum of **35** (Chloroform-d, 298 K).

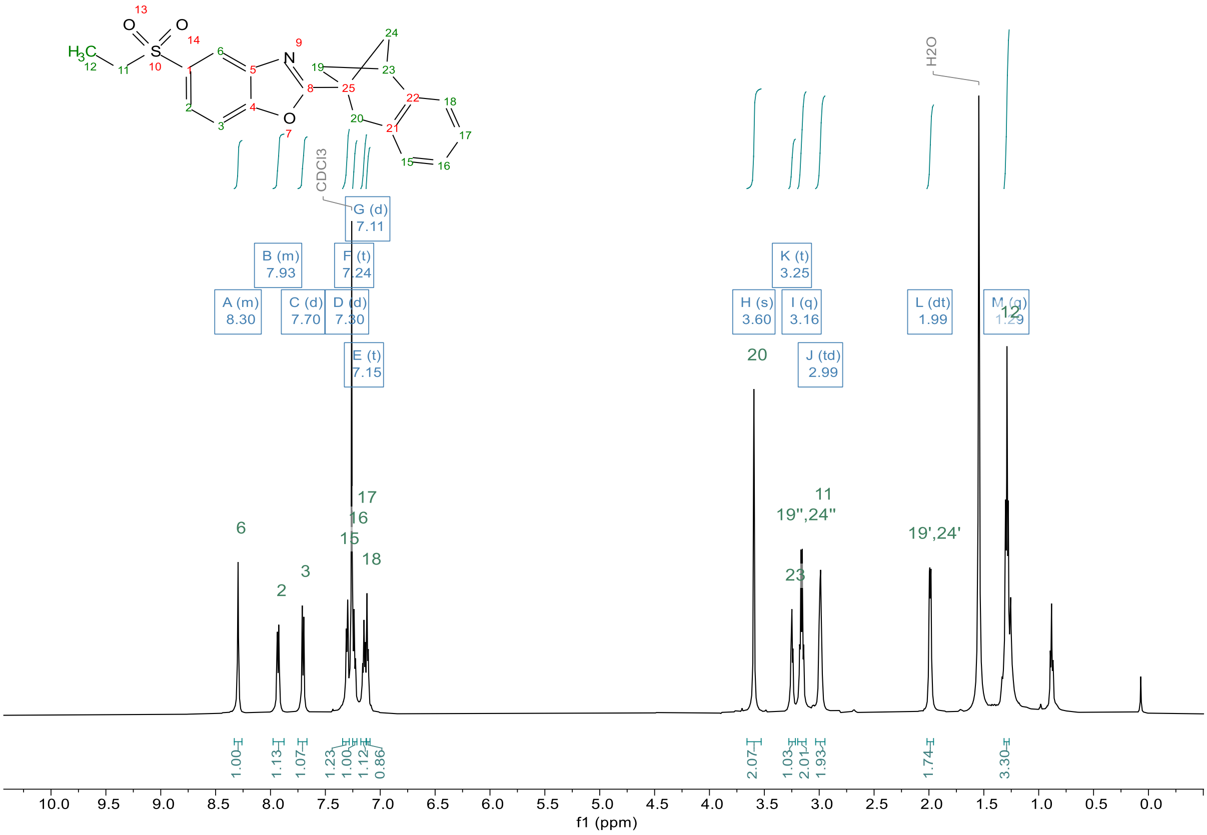

Fig.S167. ^{1}H NMR Spectrum of **50a** (Chloroform-d, 298 K).

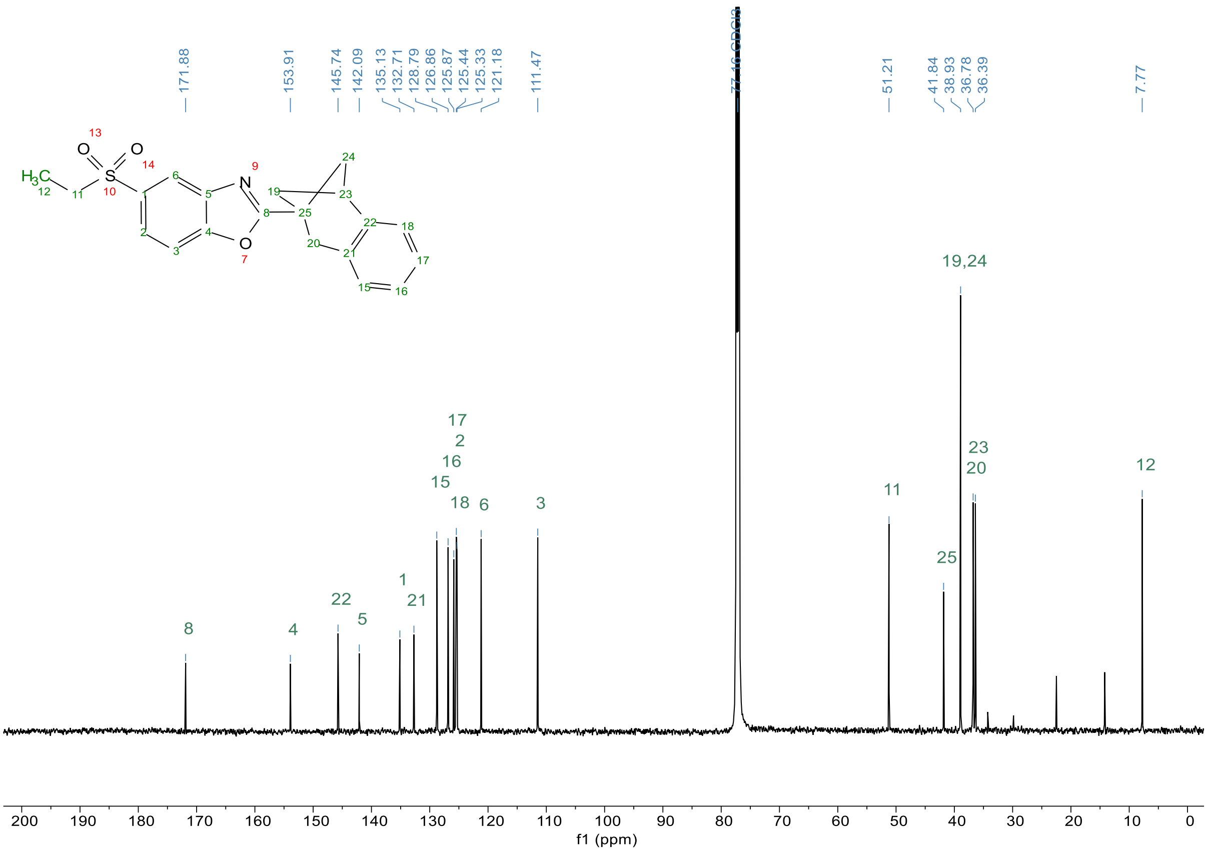

Fig.S168. ^{13}C NMR Spectrum of **50a** (Chloroform-d, 298 K).

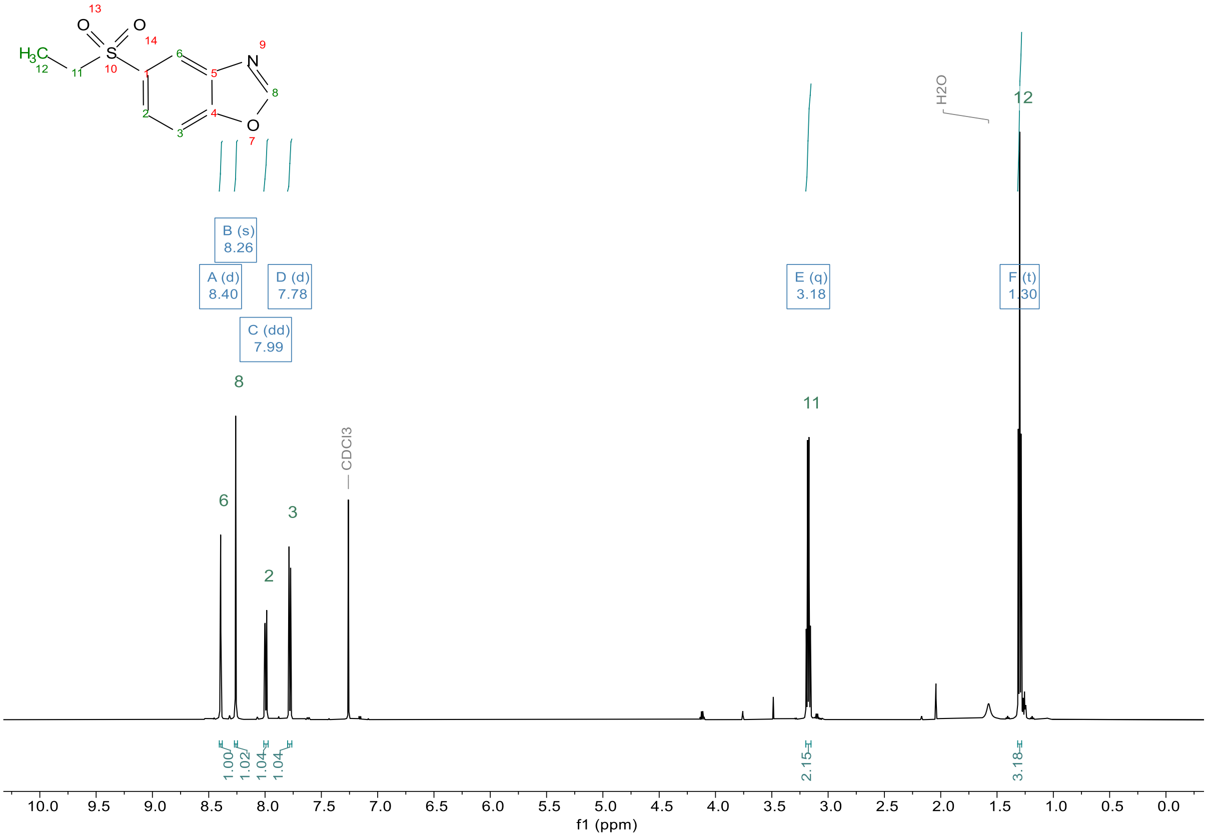

Fig.S169. ^{1}H NMR Spectrum of **S92** (Chloroform-d, 298 K).

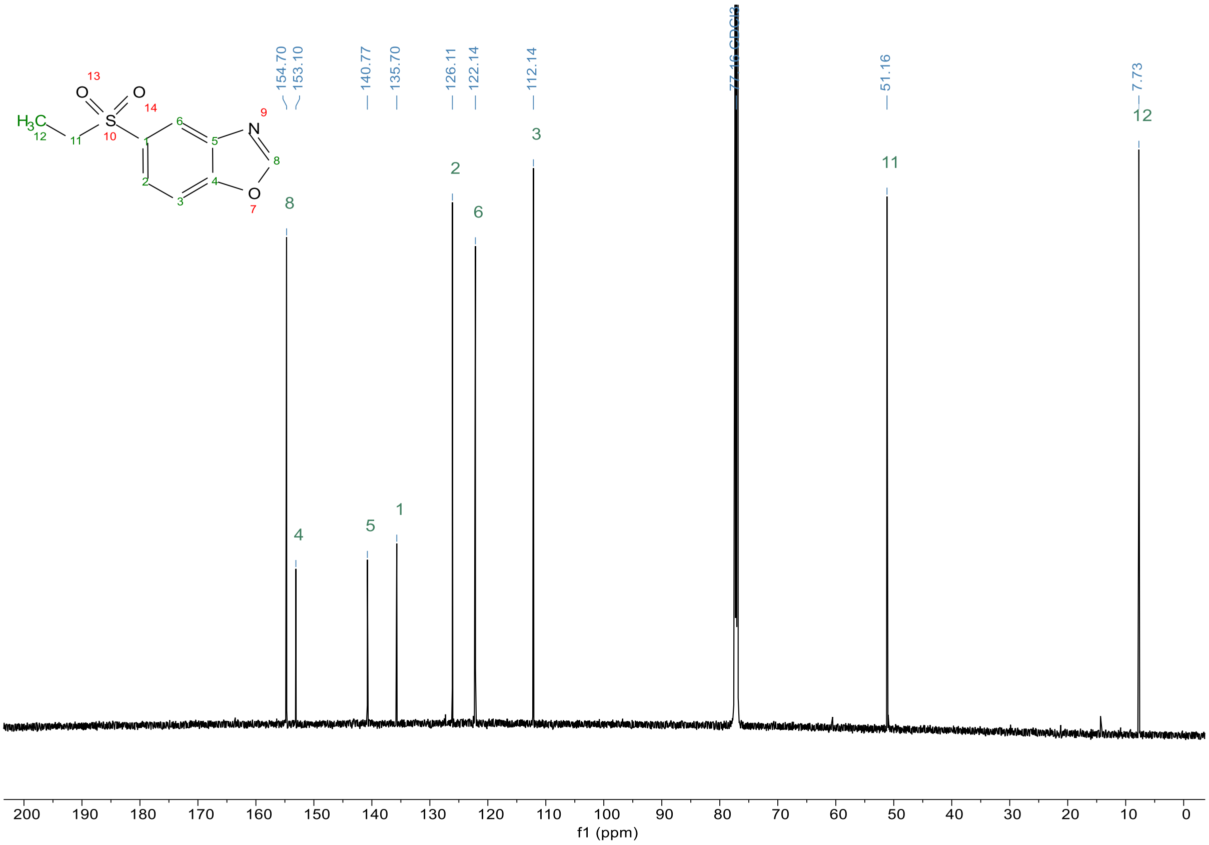

Fig.S170. ^{13}C NMR Spectrum of **S92** (Chloroform-d, 298 K).

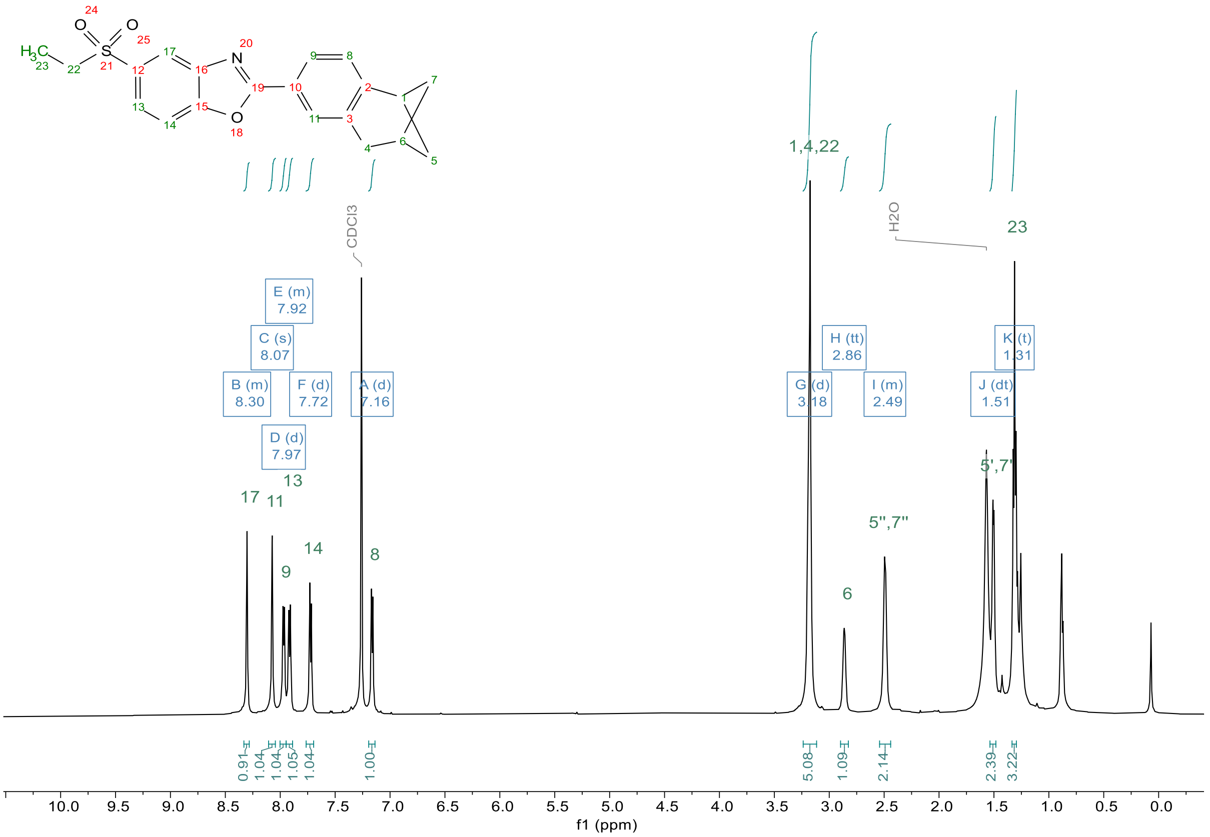

Fig.S171. ^{1}H NMR Spectrum of **50b** (Chloroform-d, 298 K).

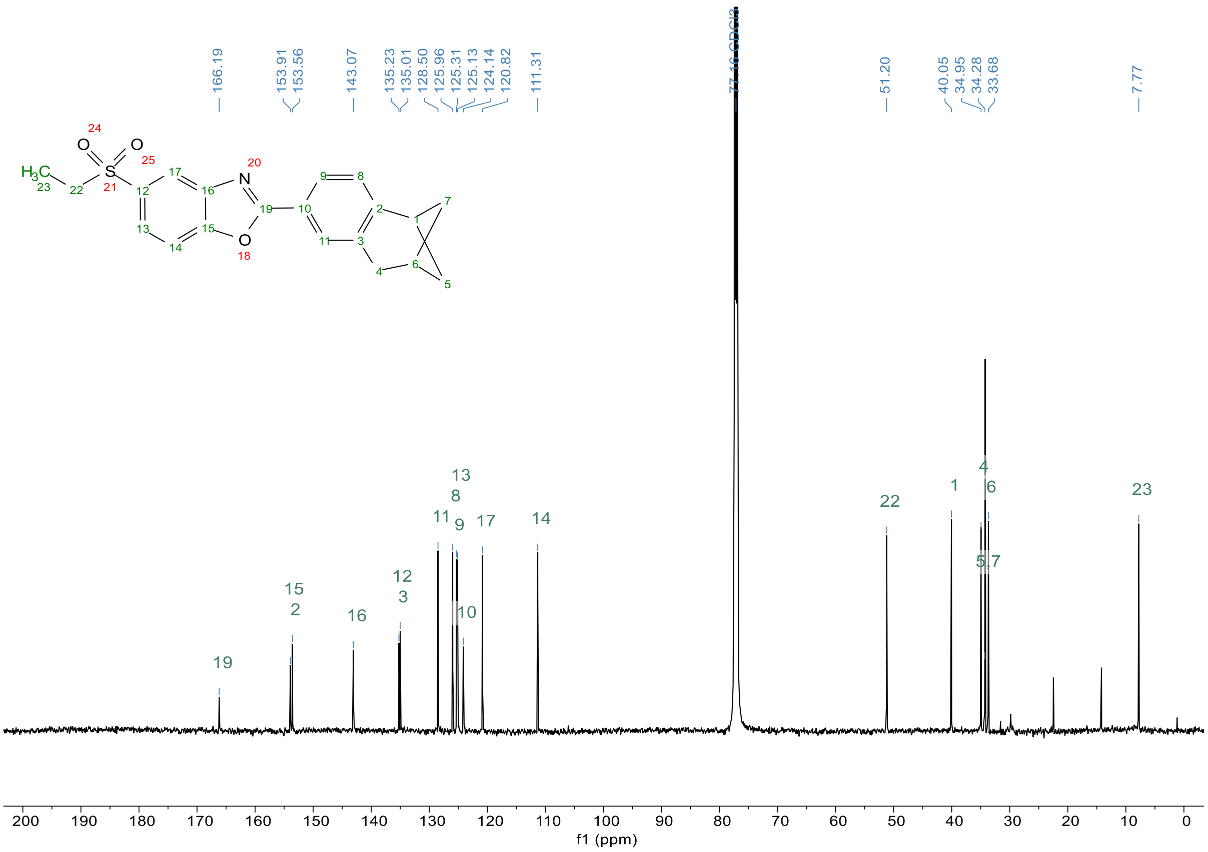

Fig.S172. ^{13}C NMR Spectrum of **50b** (Chloroform-d, 298 K).

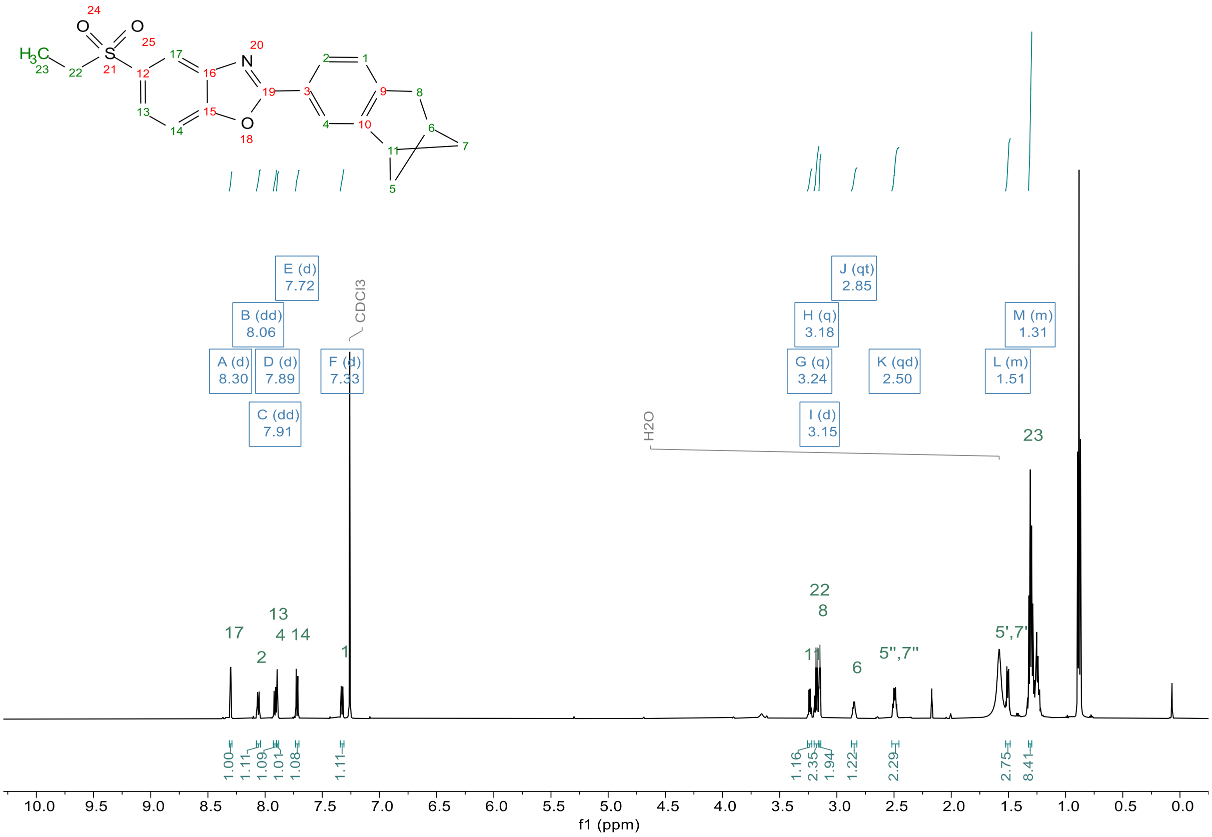

Fig.S173. ^{1}H NMR Spectrum of **50c** (Chloroform-d, 298 K).

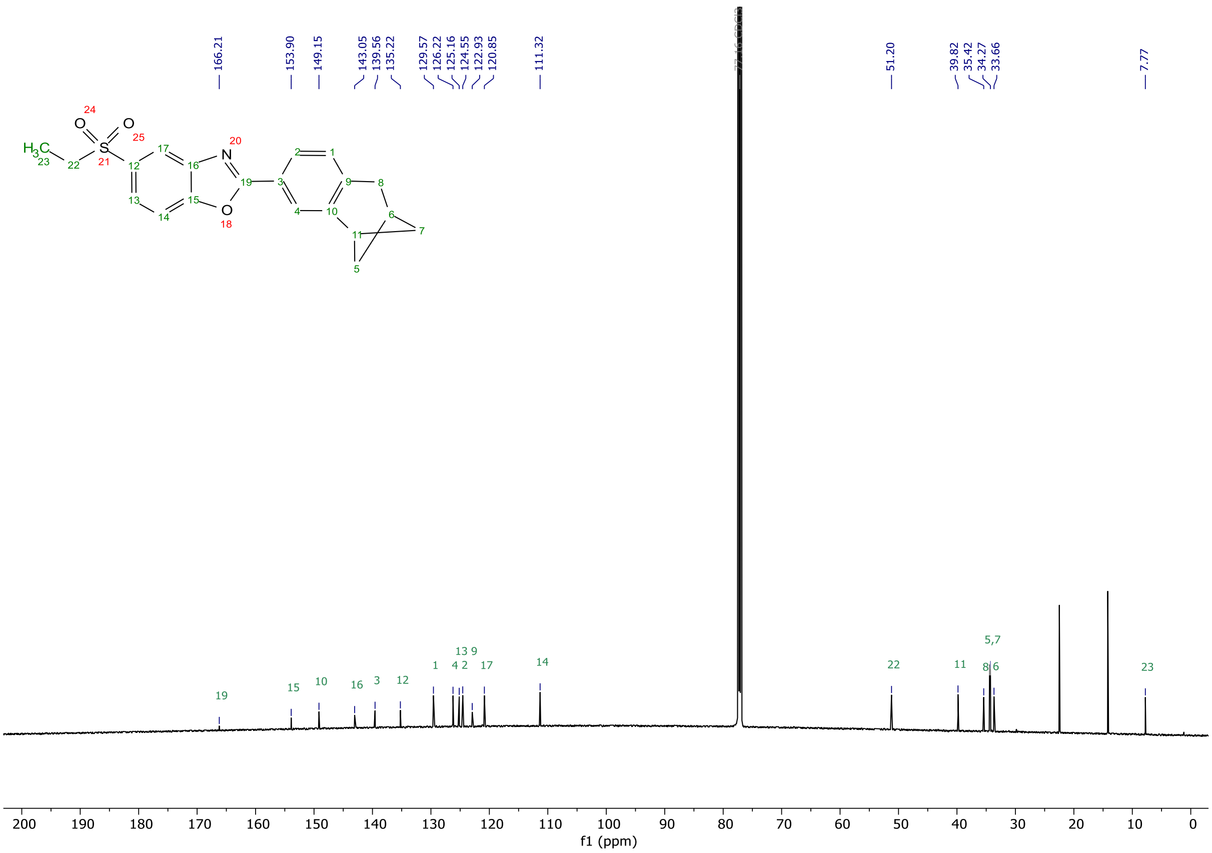

Fig.S174. ^{13}C NMR Spectrum of **50c** (Chloroform-d, 298 K).

5. Condition Screening for [2+2]

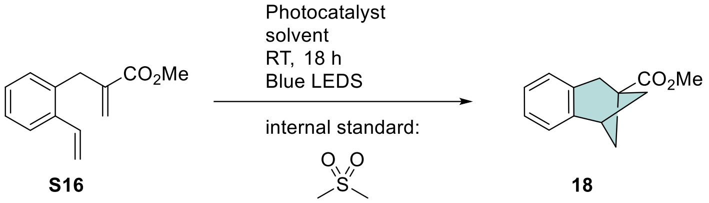

Entry	Photocatalyst	Solvent	Remaining S16	Yield of 18	Side product yield	SUM
1	$[Ir(dF(CF_3)ppy)_2(dtbpy)]PF_6$	CH_2Cl_2	0%	36%	0%	36%
2	$[Ir(dF(CF_3)ppy)_2(dtbpy)]PF_6$	MeCN	0%	30%	0%	30%
3	$[Ir(dF(CF_3)ppy)_2(dtbpy)]PF_6$	THF	0%	28%	0%	28%
4	$[Ir(dF(CF_3)ppy)_2(dtbpy)]PF_6$	DMSO	0%	33%	0%	33%
5	$[Ir(dF(CF_3)ppy)_2(dtbpy)]PF_6$	Acetone	0%	30%	0%	30%
6	$Ir(ppy)_3$	CH_2Cl_2	66%	0%	0%	66%
7	$Ir(dFppy)_3$	CH_2Cl_2	0%	40%	0%	40%
8	$[Ru(bpy)_3](PF_6)_2$	CH_2Cl_2	74%	0%	0%	74%
9*	$Ir(dFppy)_3$	CH_2Cl_2	0%	36%	0%	36%

Table S1. Condition screening for [2+2] reaction of **S16**. Conducted in NMR tube using Deuterated Solvents, 1 mol% Catalyst, 1 eq internal standard. *Reaction carried out on 30 mg scale in a microwave vial

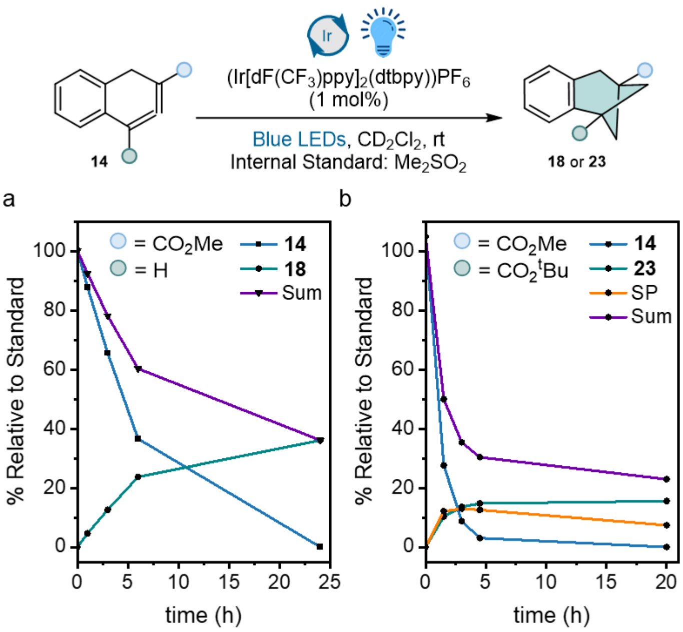

Fig.S175. Reaction progress of [2+2] cycloaddition with a) Monosubstituted and b) Disubstituted Bridgehead products . SP: Side Product, Sum: Sum of all observable components. Purple line denotes potential rates of polymerisation process, as these products are complex to integrate.

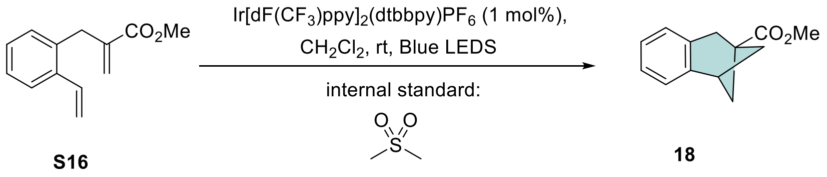

Entry	Change	Remaining S16	Yield of 18	Side product yield	SUM
1	0.1 M	0%	40%	0%	33%
2	0.005 M	0%	28%	0%	28%
3	15 mol% Pyrene, 48 h	0%	56%	0%	56%

Table S2. Attempts to optimise [2+2] reaction of **S16** with additives and dilution. Crude mixtures analysed by addition of internal standard. Sub-stoichiometric Pyrene marginally improved yields but required longer reaction times.

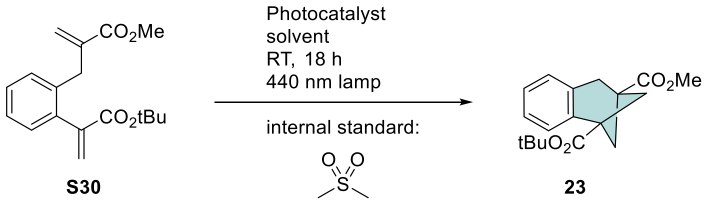

Entry	Photocatalyst	Solvent	Remaining 10	Yield of 11	Side product yield	SUM
1	[Ir(dF(CF$_3$)ppy)$_2$(dtbpy)]PF$_6$	CH$_2$Cl$_2$	0%	14%	10%	24%
2	[Ir(dF(CF$_3$)ppy)$_2$(dtbpy)]PF$_6$	MeOH	23%	5%	5%	33%

3	[Ir(dF(CF$_3$)ppy)$_2$(dtbpy)]PF$_6$	MeCN	1%	12%	4%	17%
4	[Ir(dF(CF$_3$)ppy)$_2$(dtbpy)]PF$_6$	THF	0%	7%	0%	7%
5	[Ir(dF(CF$_3$)ppy)$_2$(dtbpy)]PF$_6$	DMSO	0%	11%	0%	11%
6	[Ir(dF(CF$_3$)ppy)$_2$(dtbpy)]PF$_6$	Acetone	3%	11%	2%	16%
7	Ir(ppy)$_3$	CH_2Cl_2	85%	0%	0%	85%
8	Ir(dFppy)$_3$	CH_2Cl_2	9%	11%	8%	29%
9	[Ru(bpy)$_3$](PF6)$_2$	CH_2Cl_2	84%	0%	0%	84%

Table S3. Condition screening for [2+2] reaction of **S16**. Conducted in NMR tube using Deuterated Solvents, 1 mol% Catalyst, 1 eq internal standard.

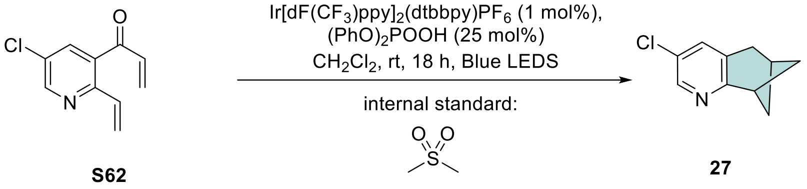

Entry	**Remaining S62**	**Yield of 27**	**Side product yield**	**SUM**
1	0%	31%	0%	31%

Table S4. [2+2] reaction of **S62**. Crude mixtures analysed by addition of internal standard.

6. Crystallographic Data

Crystals were grown by vapour diffusion. Compounds (2-5 mg) were dissolved in minimal amounts of $CDCl_3$, then filtered over cotton into a small vial. The vial was capped and punctured with a small needle, then placed into a larger vial containing pentane or hexane. The vials were stored undisturbed on the bench until suitable crystals formed:

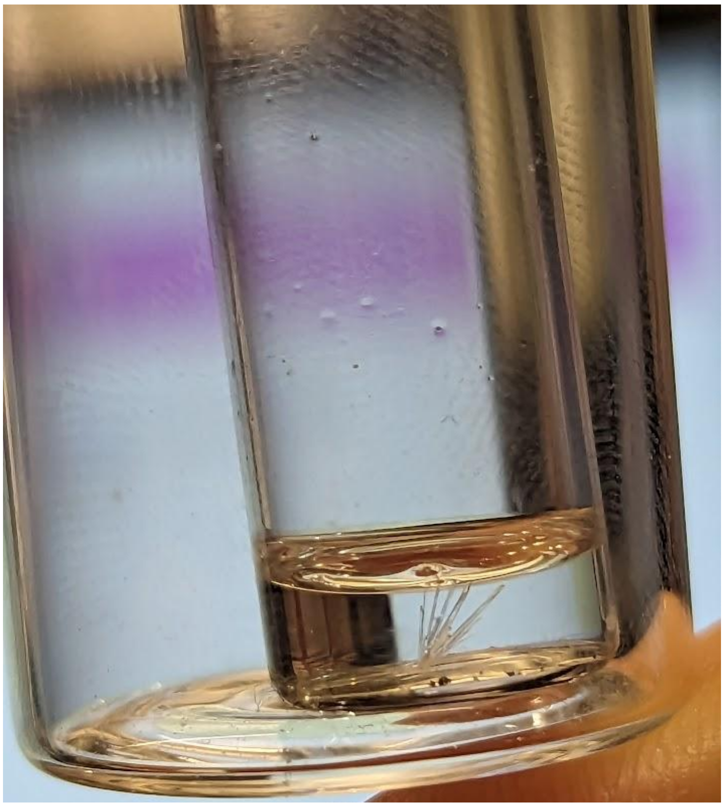

Fig.S176. Representative example of single crystal formation

Single crystal X-ray diffraction data was collected on a Rigaku Synergy-DW diffractometer at 100 K for all structures. CrysAlisPro was used for data integration and absorption correction. Structures were solved using 'Superflip'[15] before refinement with CRYSTALS [16-17] as per the SI (CIF). The crystallographic data have been deposited with the Cambridge Crystallographic Data Centre (CCDC 2368065-69), and copies of these data can be obtained free of charge from The Cambridge Crystallographic Data Centre via www.ccdc.cam.ac.uk/data_request/ cif.

X-Ray .cif files were opened in Pymol Educational Version. Bond angles and distances were meaured using the ‘measurement’ wizard and structural overlays were performed using the ‘pair-fit’ function in Pymol.

Table S5. Crystal data and structure refinement for **Ezutromid**.

Identification code	7794 [CCDC:2368069]	
Empirical formula	C19 H15 N O3 S	
Formula weight	337.40	
Temperature	100 K	
Wavelength	1.54184 Å	
Crystal system / Space group	Monoclinic	P 2_1/c
Unit cell dimensions	a = 7.74600(10) Å	a= 90°.
	b = 6.99840(10) Å	b= 97.1877(8)°.
	c = 29.2759(3) Å	g = 90°.
Volume	1574.56(3) $Å^3$	
Z	4	
Crystal size	0.30 x 0.06 x 0.04 mm^3	
Independent reflections	3225 [R(int) = 0.019]	
Completeness to theta = 74.263°	99.5 %	
Absorption correction	Semi-empirical from equivalents	
Max. and min. transmission	0.92 and 0.69	
Refinement method	Full-matrix least-squares on F^2	
Data / restraints / parameters	3224 / 528 / 374	
Goodness-of-fit on F^2	0.9978	
Final R indices [I>2sigma(I)]	R1 = 0.1368, wR2 = 0.3372	
R indices (all data)	R1 = 0.1400, wR2 = 0.3393	
Largest diff. peak and hole	1.39 and -0.77 e.$Å^{-3}$	

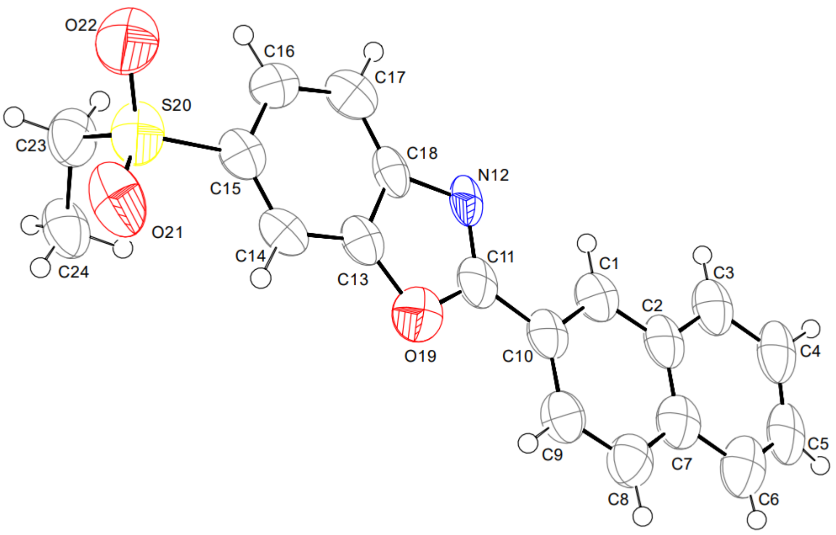

Please note that the full molecule is disordered over two positions.
Any numbering for the disorder is off-set by 100.

Table S6. Crystal data and structure refinement for **35**.

Identification code	7816 [CCDC:2368065]	
Empirical formula	C16 H14 N2 O2	
Formula weight	266.30	
Temperature	100 K	
Wavelength	1.54184 Å	
Crystal system / Space group	Triclinic	P -1
Unit cell dimensions	a = 5.91460(10) Å	a= 90.7247(11)°.
	b = 6.87670(10) Å	b= 93.7276(12)°.
	c = 16.3612(2) Å	g = 106.3785(12)°.
Volume	636.769(17) $Å^3$	
Z	2	
Crystal size	0.22 x 0.09 x 0.08 mm^3	
Independent reflections	2609 [R(int) = 0.021]	
Completeness to theta = 73.034°	99.8 %	
Absorption correction	Semi-empirical from equivalents	
Max. and min. transmission	0.94 and 0.92	
Refinement method	Full-matrix least-squares on F^2	
Data / restraints / parameters	2609 / 0 / 182	
Goodness-of-fit on F^2	1.0053	
Final R indices [I>2sigma(I)]	R1 = 0.0327, wR2 = 0.0932	
R indices (all data)	R1 = 0.0337, wR2 = 0.0944	
Extinction coefficient	21(3)	
Largest diff. peak and hole	0.23 and -0.24 e.$Å^{-3}$	

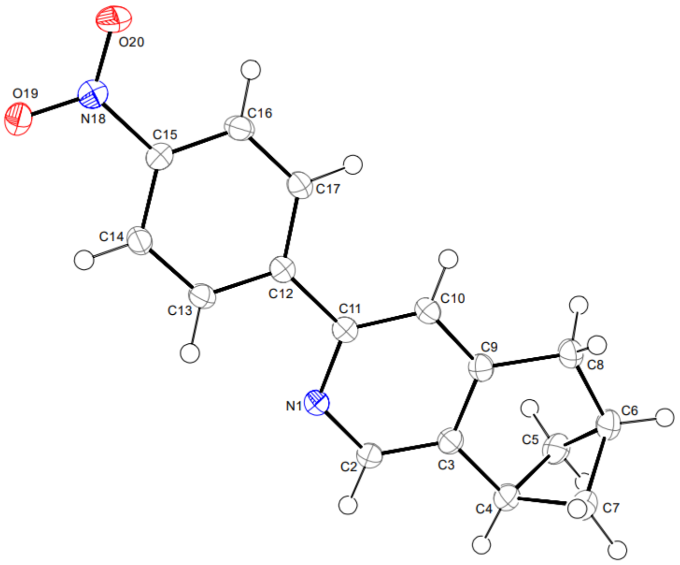

Table S7. Crystal data and structure refinement for **50a**.

Identification code	7790 [CCDC:2368067]	
Empirical formula	C20 H19 N O3 S	
Formula weight	353.44	
Temperature	100 K	
Wavelength	1.54184 Å	
Crystal system / Space group	Monoclinic	P 2_1/c
Unit cell dimensions	a = 15.25190(10) Å	a= 90°.
	b = 8.02790(10) Å	b= 116.3741(9)°.
	c = 15.59810(10) Å	g = 90°.
Volume	1711.05(3) $Å^3$	
Z	4	
Crystal size	0.20 x 0.08 x 0.03 mm^3	
Independent reflections	3506 [R(int) = 0.029]	
Completeness to theta = 74.255°	99.5 %	
Absorption correction	Semi-empirical from equivalents	
Max. and min. transmission	0.95 and 0.77	
Refinement method	Full-matrix least-squares on F^2	
Data / restraints / parameters	3506 / 0 / 227	
Goodness-of-fit on F^2	1.0013	
Final R indices [I>2sigma(I)]	R1 = 0.0280, wR2 = 0.0715	
R indices (all data)	R1 = 0.0289, wR2 = 0.0721	
Extinction coefficient	21(2)	
Largest diff. peak and hole	0.33 and -0.36 e.$Å^{-3}$	

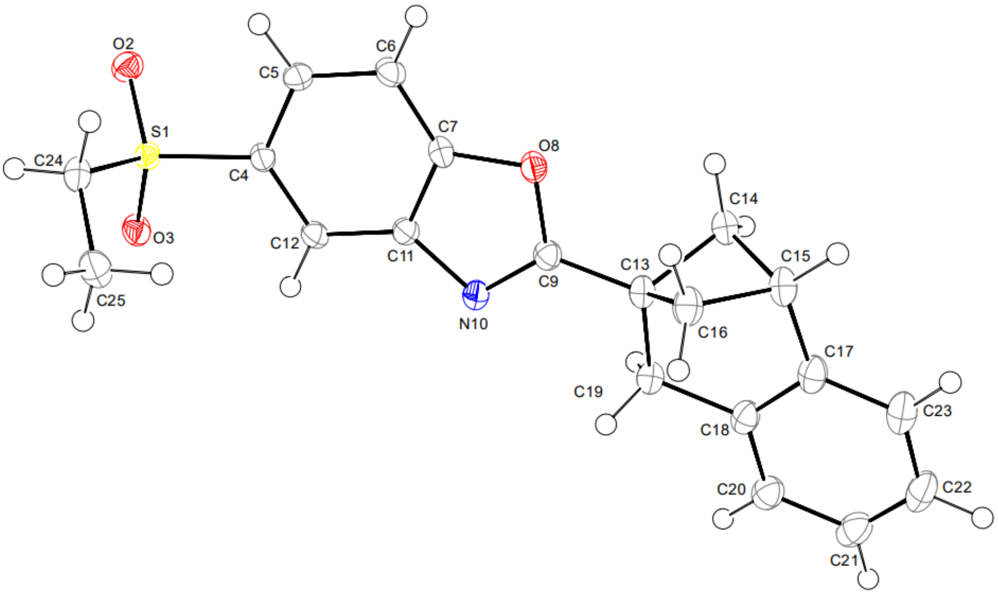

Table S8. Crystal data and structure refinement for **50b**.

Identification code	7795 [CCDC:2368068]	
Empirical formula	C20 H19 N O3 S	
Formula weight	353.44	
Temperature	100 K	
Wavelength	1.54184 Å	
Crystal system / Space group	Monoclinic	P 2_1/n
Unit cell dimensions	a = 15.78270(10) Å	a= 90°.
	b = 5.89870(10) Å	b= 111.2495(7)°.
	c = 19.13950(10) Å	g = 90°.
Volume	1660.69(3) $Å^3$	
Z	4	
Crystal size	0.20 x 0.07 x 0.02 mm^3	
Independent reflections	3413 [R(int) = 0.023]	
Completeness to theta = 73.960°	99.9 %	
Absorption correction	Semi-empirical from equivalents	
Max. and min. transmission	0.96 and 0.84	
Refinement method	Full-matrix least-squares on F^2	
Data / restraints / parameters	3413 / 0 / 227	
Goodness-of-fit on F^2	1.0013	
Final R indices [I>2sigma(I)]	R1 = 0.0294, wR2 = 0.0791	
R indices (all data)	R1 = 0.0301, wR2 = 0.0797	
Extinction coefficient	12(2)	
Largest diff. peak and hole	0.31 and -0.39 e.$Å^{-3}$	

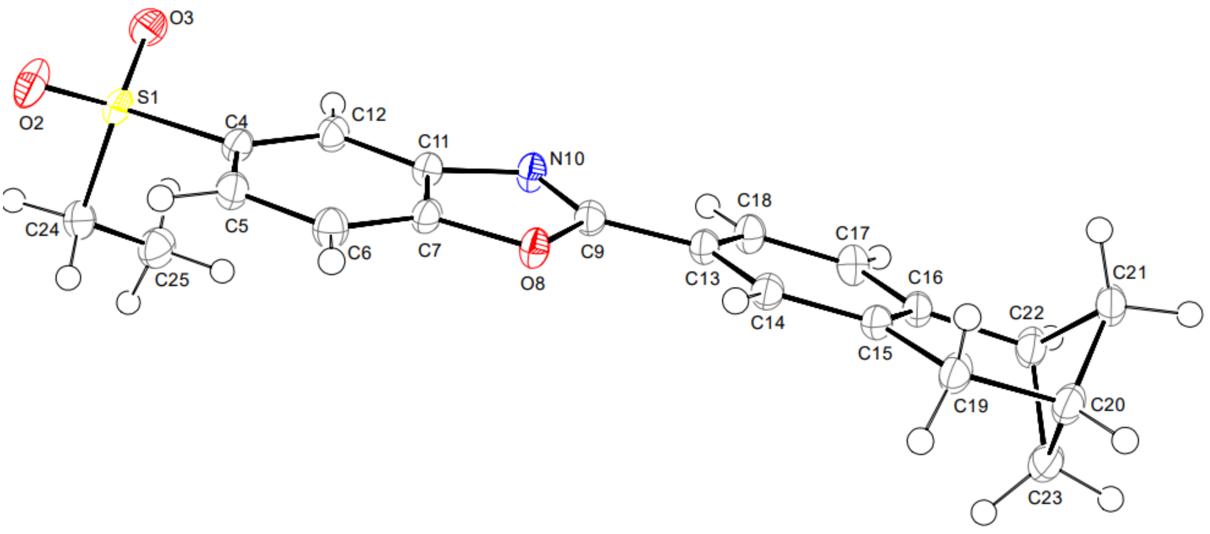

Table S8. Crystal data and structure refinement for **47**.

Identification code	7818 [CCDC:2368066]	
Empirical formula	C11 H10 O3	
Formula weight	190.20	
Temperature	100 K	
Wavelength	1.54184 Å	
Crystal system / Space group	Triclinic	P -1
Unit cell dimensions	a = 5.67790(10) Å	a= 91.408(2)°.
	b = 8.4064(2) Å	b= 97.816(2)°.
	c = 9.4532(2) Å	g = 99.627(2)°.
Volume	440.203(16) $Å^3$	
Z	2	
Crystal size	0.15 x 0.05 x 0.03 mm^3	
Independent reflections	1792 [R(int) = 0.022]	
Completeness to theta = 73.960°	99.7 %	
Absorption correction	Semi-empirical from equivalents	
Max. and min. transmission	0.97 and 0.78	
Refinement method	Full-matrix least-squares on F^2	
Data / restraints / parameters	1792 / 0 / 128	
Goodness-of-fit on F^2	0.9958	
Final R indices [I>2sigma(I)]	R1 = 0.0339, wR2 = 0.0921	
R indices (all data)	R1 = 0.0366, wR2 = 0.0942	
Extinction coefficient	8.5(19)	
Largest diff. peak and hole	0.26 and -0.20 e.$Å^{-3}$	

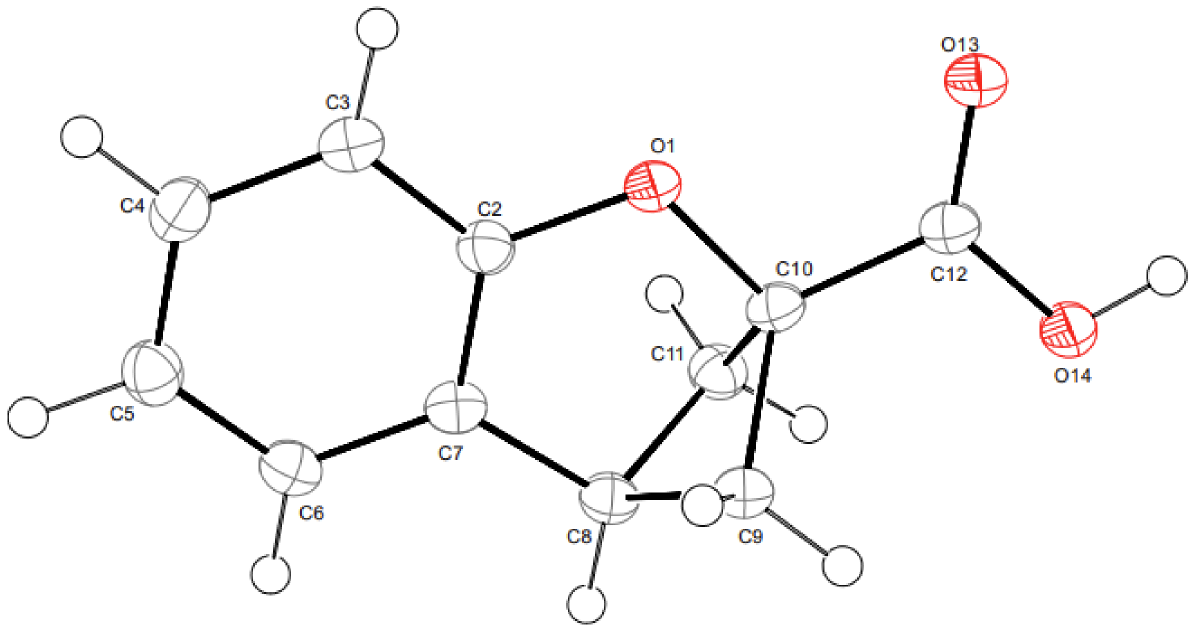

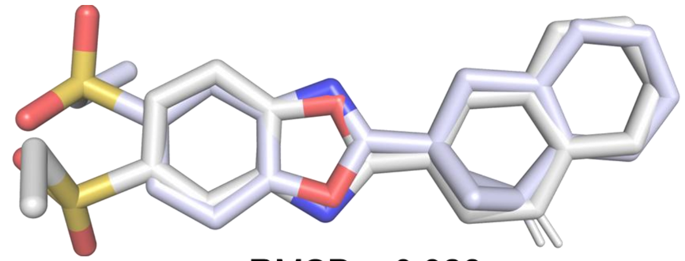

RMSD = 0.328

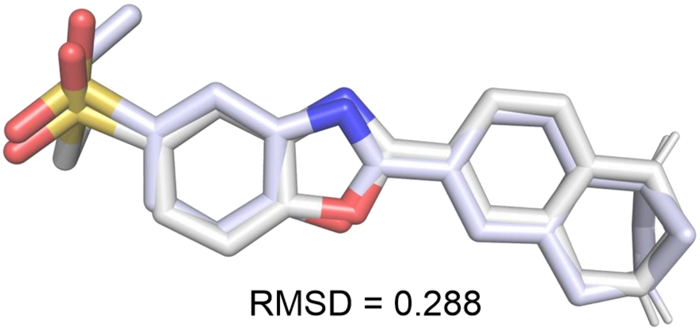

RMSD = 0.288

Fig.S177. Pair-fit of solid state structures of **50a** with **Ezutromid** (top) and **50b** with **Ezutromid** (bottom). All atoms were mapped within the benzoxazole unit and naphthalene portion of the molecule. The sulfone was omitted within the pair-fitting as the conformation varies significantly within each structure.

7. Biological Methods and Data

Cell Culture and Treatment

Human hepatocellular carcinoma (HepG2 - HB-8065) cell lines were acquired from Abcam (Cambridge, UK). Cells were cultured in Dulbecco's Modified Eagle Medium (DMEM) supplemented with 10% Fetal Bovine Serum (FBS) in 5% CO2 atmosphere at 37°C. For treatment, cells were seeded at 50.000 cells/well in 96-wells transparent plates and allowed to grow to 80% confluence. After 48 hours, cells were washed with PBS and test compounds (**50a-c**, i.a. concentration: 0.1, 1, or 10 µM with 1% DMSO; technical duplicate), positive control (**Ezutromid**, i.a. concentration: 0.1, 1, or 10 µM with 1% DMSO; technical duplicate) or vehicle (1% DMSO, technical quadruplicate) were administered and cells incubated for 4 h prior cell lysis and RNA extraction.

RNA extraction and cDNA synthesis: Total RNA was extracted using the MagMAX™-96 Total RNA Isolation Kit (Thermo Fisher Scientific, Waltham, MA, USA) following manufacturer's instructions. Briefly, cells were lysed using the provided lysis buffer and RNA was extracted using nucleic acid capturing magnetic beads. Genomic DNA was removed on-beads using the TurboDNAse kit (Thermo Fisher Scientific, Waltham, MA, USA), and RNA purified by repeated washing steps, before being eluted into 8-tube PCR strips, and stored at -20°C overnight . The quality and quantity of extracted RNA were assessed using a NanoDrop One Microvolume UV-Vis Spectrophotometer (Thermo Fisher Scientific, Waltham, MA, USA). Complementary DNA (cDNA) was synthesized from 200ng of total RNA using the High-Capacity cDNA Reverse Transcription Kit (Thermofisher) according to the manufacturer's protocol.

RT-qPCR analysis: RT-qPCR was performed using Fast SYBR™ Green Master Mix (Thermofisher Scientific) in 384-well plate on a LightCycler 480 II (Roche). Each reaction was run in duplicate using cDNA synthesized from two individual wells. Amplification was carried out using the following custom primer pairs obtained by Merck (Darmstadt, Germany): hS13 (Housekeeping Gene): Forward - CTGATCTTCCTGAAGATCTCTAC; Reverse - GGCAGAGGCTGTAGATGATTCA; hCYP1A1 (Gene of Interest) Forward - GCTCCAAGAGTCCACCCTTCCC, Reverse – CTGAGGTCTTGAGGCCCTGATTACC.

Data Analysis: Values obtained from qPCR amplification were analysed using the $2^{-\Delta\Delta CT}$ method to determine fold difference compared to the vehicle.[18] For enhancing data visualization, values were normalized against vehicle control and converted to percentage to provide a precise estimate of AhR inhibition as directly proportional to CYP1A1 downregulation. A total of 4 independent experiments were performed (n=4). One-way ANOVA followed by Dunnett's multiple comparisons test was performed using GraphPad Prism version 8.0.2 for Windows (GraphPad Software, Boston, MA, USA)

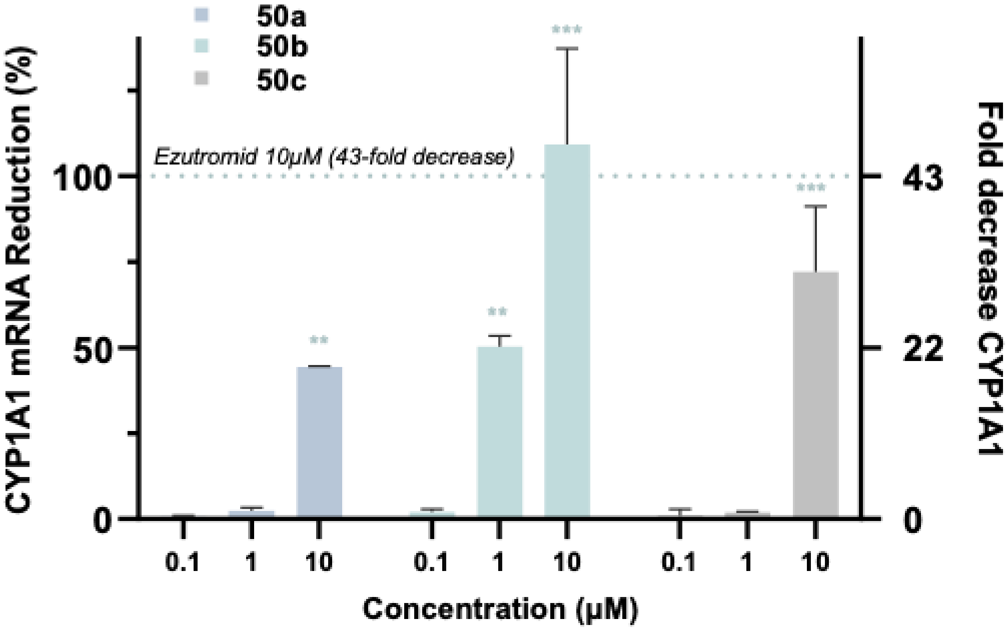

Fig.S178. Evaluation of 50a-c as AhR antagonists compared to Ezutromid. Data have been obtained by RT-qPCR, and analysed using the $2^{-\Delta\Delta Ct}$ method. Values obtained have been normalized to control (DMSO 1%), and presented as Mean + SEM (n=4). Statistical analysis was performed using the Ordinary One-way ANOVA followed by Dunnett's multiple comparisons test against Vehicle Control: **: $p < 0.005$; ****: $p < 0.0001$ (grey).

Metabolic stability

Metabolic stability of compounds was assessed in Mouse Liver Microsomes (MLM) by WuxiAppTech (Shangai, China). Briefly, ACN solutions of test compounds (**50a-c**, i.a concentration 1µM) were dispensed in in duplicate in a 96-well plate using an Apricot automation workstation. A solution of MLM (CD-1 Mouse, RILD, Cat. number LM-XS-02M) in PBS, with or without ANF, was added to each compound and plates were incubated at 37°C for 10 minutes. Reactions were then initiated by adding NADPH (i.a. 1mM, 0.5mM $MgCl_2$) and incubated for 0, 5, 15, 30, 45 or 60 minutes at 37 degrees (incubation at 0 minutes was performed by adding stop solution before activating the reaction). NADPH-independent metabolism was assessed for each condition by replacing NADPH solution with PBS buffer. Phenacetine, diclofenac and propafenone were used as controls.

After proper incubation time, reaction was stopped by addition of ice-cold ACN and the plates were sealed, shaken and centrifuged at 1800 x g and 4°C for 20 minutes. The supernatant was then subjected to LC/MS-MS analysis for residual drug quantitation using 250 nM tolbutamide and 250 nM labetalol as internal standards. Half-life ($t_{1/2}$) and CL_{int} (Intrinsic clearance) were calculated using standard pharmacokinetic calculations based on the decay of drug concentration over time.

MetID of 50b

The test compound **50b** at 10 µM was incubated with liver microsomes in the presence of NADPH at 37°C for 60 min. The positive control, 7-ethoxycoumarin (7-EC) at 10 µM, was run concurrently to

assess Phase I metabolic activities in liver microsomes. The results indicated that liver microsomes incubation system was reliable for metabolic study. After incubation, the samples were analyzed by LC-UV-MS. The structures of the metabolites were proposed based on the interpretation of their MS and MS2 data. In addition to the unchanged **50b** (MW = 353.44), a total of 5 metabolites of **50b** were detected and identified by LC-UV-MS^n (n=1~2) from human liver microsomes. The metabolites were assigned as below: M1: Di-oxygenation metabolite (MW = 385.43, P + 2O); M2, M3, M4 and M5: Mono-oxygenation metabolites (MW = 369.44, P + O). In human liver microsomes, **50b** and 5 metabolites (M1-M5) were detected. M4 was considered as the top metabolite with relative abundance of 16.16%, The relative abundance of the other metabolites was less than 9%. The parent, **50b**, accounted for 66.02% of the total drug related components in human liver microsomes after incubation at 37°C for 60 min.

Code	Theoretical Mass (*m/z*)	[M+H]$^+$ *m/z*	RT (min)	Relative Abundance (UV Peak Area %Total)	Metabolic Pathways
M1	386.1057	386.1064	7.32	0.52	Di-oxygenation (P + 2O)
M2	370.1108	370.1114	7.87	2.23	Mono-oxygenation (P + O)
M3	370.1108	370.1111	7.97	8.05	Mono-oxygenation (P + O)
M4	370.1108	370.1111	8.28	16.16	Mono-oxygenation (P + O)
M5	370.1108	370.1113	8.43	7.03	Mono-oxygenation (P + O)
**50b**	354.1159	354.1162	12.14	66.02	NA

Note: RT: Retention time of LC-MS; NA: Not applicable; P: Parent.

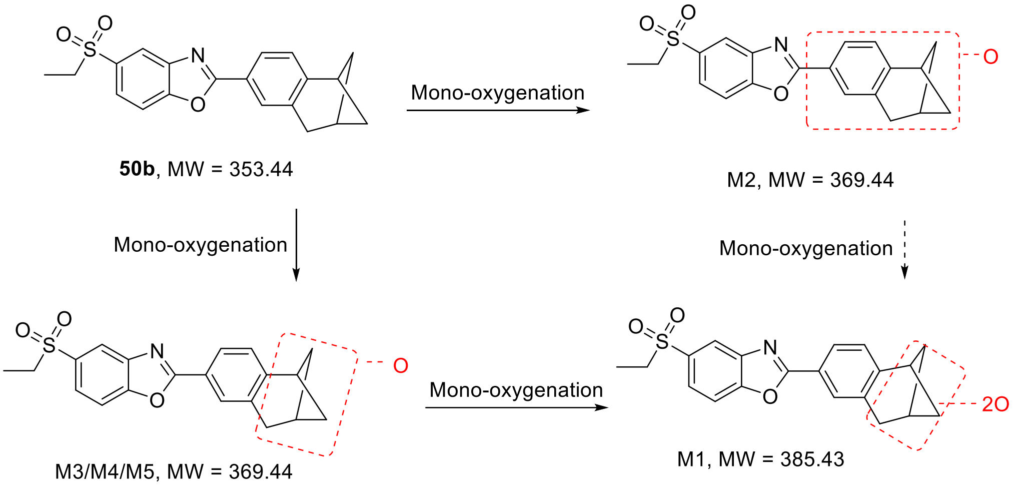

Fig. S179. Summary of **50b** and its metabolites. Liver Microsomes and proposed metabolic pathways of 50b in human liver microsomes

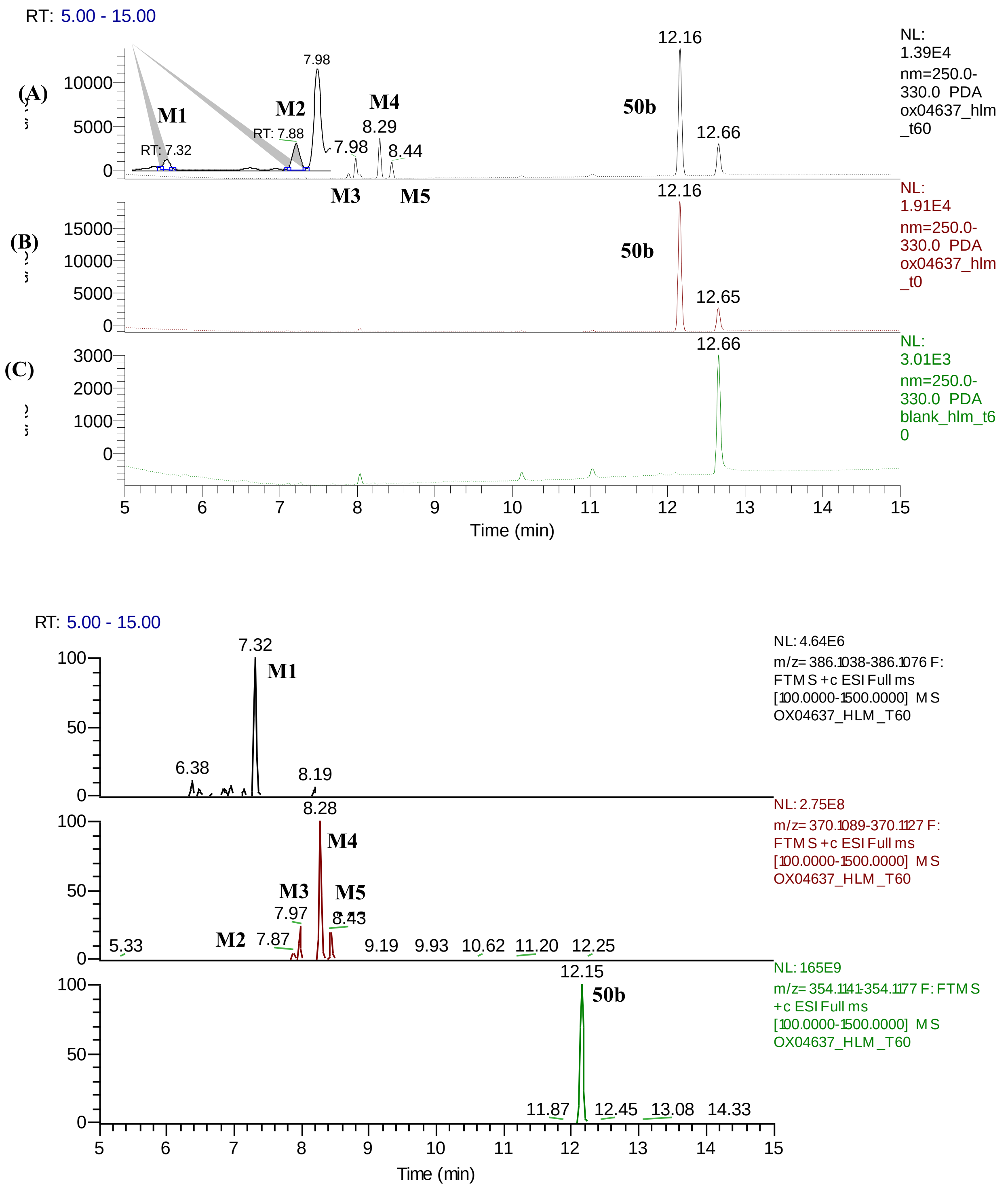

Fig. S180. LC-UV (λ: **Error! Reference source not found.**) (Top) and LC-HRMS (XICs) (Bottom) Chromatograms of **50b** and its Metabolites in Human Liver Microsomes Note: (A): LC-UV for T**Error! Reference source not found.**; (B): LC-UV for T0; (C): LC-UV for Blank

Solubility Assay

A 50 mM phosphate buffer (pH 7.4) was prepared by mixing 50 mM Na_2HPO_4 (3.549 g in 500 mL H_2O, pH ≈ 9.4) and 50 mM NaH_2PO_4 (3.000 g in 500 mL H_2O, pH ≈ 4.5). To obtain the working buffer, 15 mL of Na_2HPO_4 solution was transferred into a 50 mL tube and adjusted to pH 7.4 ± 0.05 with 50 mM NaH_2PO_4. For solubility determination, 10 µL of compound stock solution was added to each well of a 96-well plate, followed by 490 µL of buffer. Samples were vortexed for 2 min, shaken at 70 x g for 24 h at room temperature, and then centrifuged at 25 °C for 10 min (1800 x g). Supernatants were filtered through MultiScreen® Solvinert plates (Millipore/Merck) by centrifugation (≥ 5 min) and collected into a fresh 96-well plate. Filtrate concentrations were quantified by LC-UV using an ACQUITY UPLC BEH C18 (1.7 µm, 50 mm) column with Mobile Phase A (0.1% TFA, 5 mM NH_4OAc in H_2O/ACN 95:5 v/v) and Mobile Phase B (0.1% TFA, 5 mM NH_4OAc in H_2O/ACN 5:95 v/v).

All compounds displayed very low solubility below the detection threshold (**Table S9**, < 1.56 µM), indicating that other factors - such as overall lipophilicity and/or the ethyl sulfone functional group - likely limit aqueous solubility in this series. These results highlight the need for further optimisation to improve solubility alongside metabolic stability and biological activity.

Compound ID	Batch	Media	Kinetic Solubility (µM)	Kinetic Solubility (µg/mL)
50a	OX04620	50 mM PB (pH 7.4)	<1.56	<0.552
50b	OX04637	50 mM PB (pH 7.4)	<1.56	<0.552
Ezutromid	C1100	50 mM PB (pH 7.4)	<1.56	<0.527

Table S9. Kinetic Solubility Summary

8. Computational Studies

All calculations were carried out using the ORCA suite of programs (version 5.0.3).[19] Optimizations and single-point energy calculations were carried out using “VeryTight” convergence criteria, corresponding to tolerances of 10^{-9} Hartrees for the SCF energy change, and an energy change of $2 \cdot 10^{-7}$ Hartrees for the optimisation step.

The double-hybrid functional B2PLYP, in combination with D3-BJ dispersion correction,[20,21] was used as the central method for geometry optimisation being reliable employed in optimisation of organic molecules in combination with the basis sets def2-SVP[22] and def2-TZVPP. [22] The correlation integrals def2-TZVPP/C[23] and def2-SVP/C[24] were used as auxiliary basis set for the def2-TZVP and def2-SVP basis sets, respectively.

The software package CREST[25] was used in combination with the semiempirical quantum chemistry package xtb for conformational sampling of each structure. xtb calculations were performed employing the GFN2[26] semiempirical tight binding method in combination with the GBSA(water) solvent model. Relevant conformers were reoptimized at CPCM(water)-B2PLYP-D3BJ/def2-SVP level of theory (298 K / 1 M).[27]

The Domain-based Local Pair Natural Orbital coupled cluster method with singles, doubles and perturbative triples (DLPNO-CCSD(T)) was used as a final single point providing reliable electronic energies using the def2-TZVPP basis set. DLPNO-CCSD(T) calculations were run using "NormalPNO" cut-offs. [28]

Vibrational frequencies were computed at the optimization level of theory to confirm whether the structures correspond to minima. All intermediate structures were verified to be minima by the absence of imaginary frequencies upon calculation of the Hessian. Grimme's quasiRRHO approach was used to calculate free energies at 289 K. For calculating thermodynamic data, the python-script *OTherm.py*[29] was used with ω_0 = 100 cm^{-1} replacing harmonic oscillators with free-rotors below ω_0.[30]

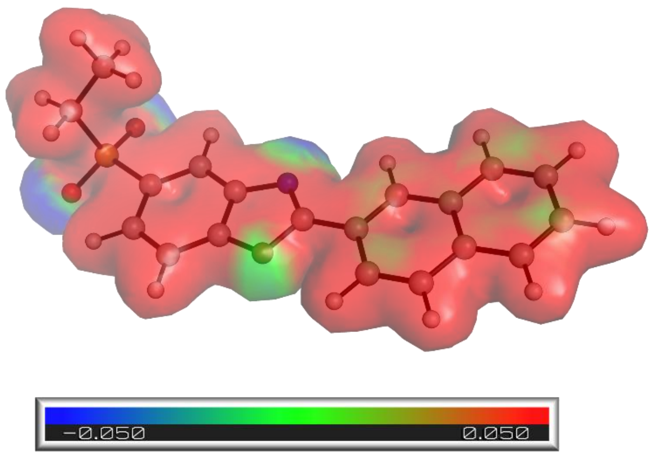

Figure S181. Electrostatic potential map for ezutromid at CPCM (water)-B2PLYP-D3BJ/def2-TZVPP//CPCM (water)-B2PLYP-D3BJ/def2-SVP level of theory with an isovalue of 0.02, from -0.05 to 0.05.

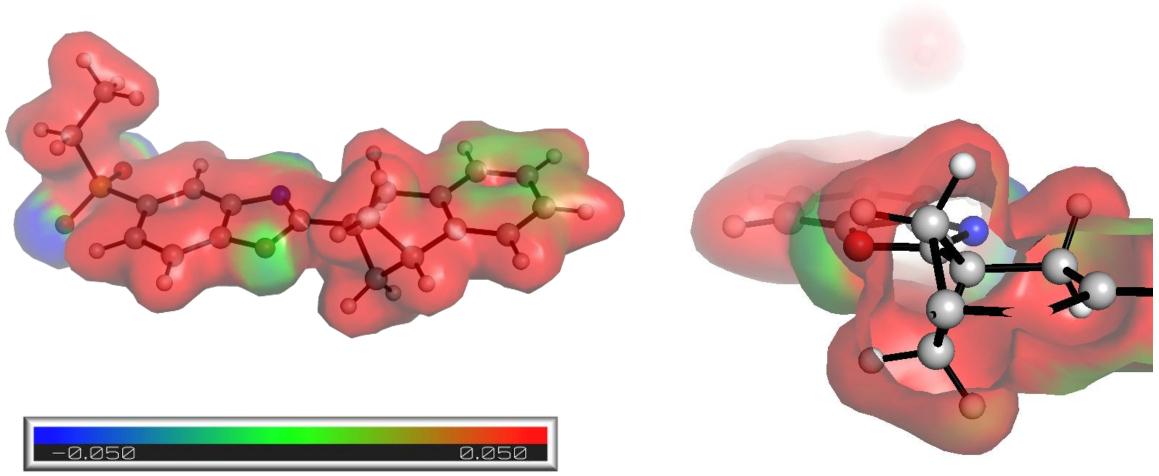

Figure S182. Electrostatic potential map for **50a,** with zoom-in to BCHep portion at CPCM (water)-B2PLYP-D3BJ/def2-TZVPP//CPCM (water)-B2PLYP-D3BJ/def2-SVP level of theory with an isovalue of 0.02, from -0.05 to 0.05.

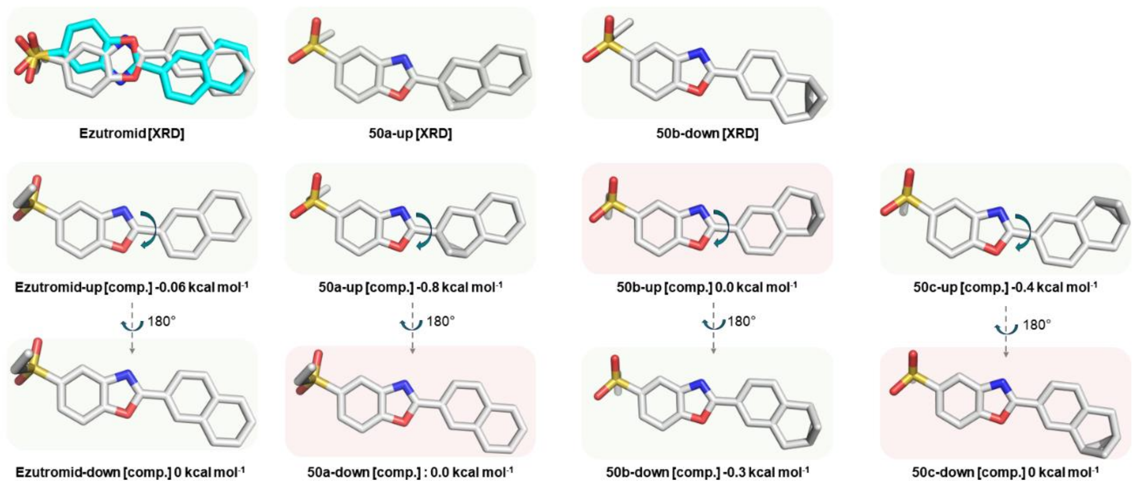

Figure S183. Top row: crystal structures of computed derivatives. Ezutromid contains two 'downwards' facing conformers, with opposing sulfone conformations. + structures of each derivative (up and down conformations). Middle/bottom row: computed structures of each derivative, where the naphthyl or BCHep unit is rotated by 180 degrees. Add the end Computations were performed at CPCM (water)-DLPNOCCSD(T)/def2-TZVPP/CPCM(water)-B2PLYP-D3BJ/def2-SVP level of theory (298.15 K / 1 M).

Compound	E at B2PLYP-D3BJ/def2-SVP / kcal mol^{-1}	G at B2PLYP-D3BJ/def2-SVP / kcal mol^{-1}	Eel at DLPNO-CCSD(T)/def2-TZVPP / kcal mol^{-1}	G(corrected) / kcal mol^{-1}	Difference / kcal mol^{-1}
**Ezutromid-up**	-1409.654492	-1409.397793	-1409.499453	-1409.242754	
**Ezutromid-down**	-1409.655035	-1409.397338	-1409.500359	-1409.242663	0.06
**50a-up**	-1450.056492	-1449.748824	-1449.918978	-1449.611309	
**50a-down**	-1450.057192	-1449.747686	-1449.919497	-1449.609991	0.83

**50b-up**	-1449.752416	-1450.058602	-1449.917819	-1449.611632	
**50b-down**	-1449.752446	-1450.058531	-1449.918176	-1449.612092	-0.29
**50c-up**	-1449.752529	-1450.05845	-1449.917884	-1449.612524	
**50c-down**	-1449.752983	-1450.058483	-1449.918024	-1449.611963	0.35

Table S10. Calculated energies of each conformer. Optimised at CPCM(water)-DLPNO-CCSD(T)/def2-TZVPP/CPCM(water)-B2PLYP-D3BJ/def2-SVP at 298.15 K / 1 M

To gain a deeper understanding of the conformational space available for Ezutromid and BCHep-Ezutromid and their interconversion, a torsional scan was performed at the CPCM(water)-B2PLYP-D3BJ/def2-SVP level of theory (Figure SXX). Stationary points, including minima Ezutromid-① and Ezutromid-② and the transition state between them, were characterised at the CPCM(water)-B2PLYP-D3BJ/def2-TZVPP//CPCM(water)-B2PLYP-D3BJ/def2-SVP (298 K / 1 M) level of theory.

The rotational PES of BCHep-Ezutromid is shallow, with an electronic activation energy ($\Delta E^{\ddagger}$) of no more than 1.3 kcal mol^{-1} between states. This contrasts with the parent Ezutromid, where torsional barriers are 6.7 kcal mol^{-1} between two nearly degenerate conformational states, Ezutromid-① and Ezutromid-②. These distinct conformational states of Ezutromid arise from the aromatic conjugation stabilisation present in the periplanar states Ezutromid-① and Ezutromid-② (Φ = 0.9°) but not in the saturated BCHep-analogue, explaining the shallow conformational landscape. Consequently, the bioisosteric BCHep-Ezutromid can be considered highly flexible, capable of adopting any torsional conformation along the phi dihedral without a significant energy penalty, unlike the parent Ezutromid. This flexibility might lead to more favourable binding energies of the bioisosteric compound in the drug target.

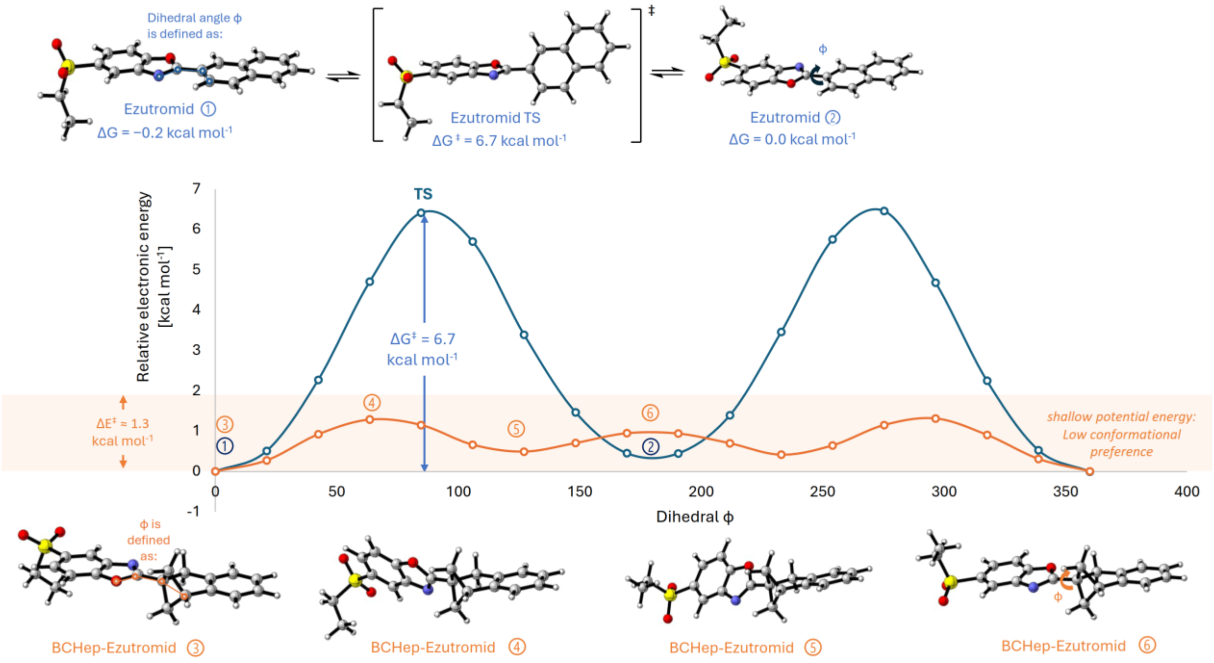

Figure S184. A torsional scan along the dihedral angle Φ between the oxazolyl-napthyl- and oxazolyl-BCHep bond of Ezutromid and BCHep-Ezutromid (highlighted in blue and orange, respectively). The scan was performed at the CPCM(water)-B2PLYP-D3BJ/def2-SVP level of

theory. Stationary points for Ezutromid were further characterised at CPCM(water)-B2PLYP-D3BJ/def2-TZVPP//CPCM(water)-B2PLYP-D3BJ/def2-SVP (298 K / 1 M) level of theory. energies in kcal mol^{-1}.For BCHep-Ezutromid, only the Φ-constrained optimized geometries are depicted. See Figure S180 for fully optimised stationary points.

Ezutromid-TS

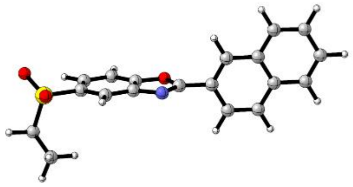

Electronic energy: −1409.64453836

Imaginary frequency: −30.98 cm^{-1}

O	-5.007764	-0.225286	1.758389
S	-5.025408	0.471455	0.456349
C	-3.333400	0.646920	-0.094167
C	-3.057563	1.585653	-1.111242
C	-1.753669	1.768569	-1.573340
C	-0.778803	0.984116	-0.962359
O	0.561219	0.956639	-1.197500
C	1.053727	0.019984	-0.317468
C	2.507557	-0.224981	-0.352124
C	3.356140	0.540856	0.428667
C	4.759321	0.317527	0.405422
C	5.654525	1.086735	1.201519
C	7.013286	0.847302	1.157775
C	7.535738	-0.170338	0.317151
C	6.691923	-0.933040	-0.465660
C	5.286282	-0.711558	-0.443545
C	4.387558	-1.480433	-1.235704
C	3.029382	-1.246660	-1.195226
N	0.158236	-0.538839	0.437454
C	-1.043542	0.052728	0.057759
C	-2.350396	-0.132343	0.518742
C	-5.811773	-0.609479	-0.748947
C	-5.260266	-2.023198	-0.739269
O	-5.674723	1.792577	0.353735
H	-3.872881	2.177332	-1.528430
H	-1.513682	2.488472	-2.355424
H	2.953126	1.324274	1.074403

H	5.246962	1.868790	1.845886
H	7.692825	1.441851	1.771138
H	8.612440	-0.348290	0.292537
H	7.090751	-1.717252	-1.112765
H	4.790848	-2.264773	-1.879805
H	2.345731	-1.841105	-1.803631
H	-2.592957	-0.835970	1.314159
H	-5.699199	-0.102077	-1.717195
H	-6.870159	-0.563554	-0.450177
H	-5.370283	-2.473606	0.255476
H	-4.198604	-2.042876	-1.021744
H	-5.817118	-2.633957	-1.462597

9. References

0. Kato, Y. & Mase, T. A novel reduction of pyridinemethanols by samarium diiodide. *Tetrahedron Lett.* **40**, 8823-8826 (1999).
1. Larionova, N. A., Ondozabal, J. M. & Cambeiro, X. C. Reduction of Electron-Deficient Alkenes Enabled by a Photoinduced Hydrogen Atom Transfer. *Adv. Synth. Catal.* **363**, 558-564 (2021).
2. Krafft, M. E., Song, E.-H. & Davoile, R. J. Intramolecular Morita–Baylis–Hillman adducts via sequential MBH and ring-closing-metathesis reactions. *Tetrahedron Lett.* **46**, 6359-6362 (2005).
3. Ramachandran, P. V. & Nicponski, D. R. Diastereoselective synthesis of α-(aminomethyl)-γ-butyrolactones via a catalyst-free aminolactonization. *Chem. Commun.* **50**, 15216-15219 (2014).
4. Bakthadoss, M., Reddy, T. T., Agarwal, V. & Sharada, D. S. Ester-directed orthogonal dual C–H activation and ortho aryl C–H alkenylation via distal weak coordination. *Chem. Commun.* **58**, 1406-1409 (2022).
5. Zhang, X. et al. Copper-mediated synthesis of drug-like bicyclopentanes. *Nature* **580**, 220-226 (2020).
6. Zhao, J. et al. Intramolecular Crossed [2+2] Photocycloaddition through Visible Light-Induced Energy Transfer. *J. Am. Chem. Soc.* **139**, 9807-9810 (2017).
7. Gonzalez-Gomez, J. C., Ramirez, N. P., Lana-Villarreal, T. & Bonete, P. A photoredox-neutral Smiles rearrangement of 2-aryloxybenzoic acids. *Org. Biomol. Chem.* **15**, 9680-9684 (2017).
8. Liu, Z.-C., Yue, W.-J. & Yin, L. Copper(I)-Catalyzed Asymmetric Synthesis of Unnatural α-Amino Acid Derivatives and Related Peptides Containing γ-(aza)Aryls. *J. Org. Chem.* **87**, 399-405 (2022).
9. Vautravers, N. R., Regent, D. D. & Breit, B. Inter- and intramolecular hydroacylation of alkenes employing a bifunctional catalyst system. *Chem. Commun*. **47**, 6635-6637 (2011).
10. Hyde, A. M. et al. Synthesis of the GPR40 Partial Agonist MK-8666 through a Kinetically Controlled Dynamic Enzymatic Ketone Reduction. *Org. Lett.* **18**, 5888-5891 (2016).
11. Xiang, B., Belyk, K. M., Reamer, R. A. & Yasuda, N. Discovery and Application of Doubly Quaternized Cinchona-Alkaloid-Based Phase-Transfer Catalysts. *Angew. Chem. Int. Ed.* **53**, 8375-8378 (2014).
12. Zhang, J. et al. Design and synthesis of 1H-pyrazolo[3,4-d]pyrimidine derivatives as hematopoietic progenitor kinase 1 (HPK1) inhibitors. *Bioorg. Chem*. **140**, 106811 (2023).
13. Mohammed, Y. H. E. et al. The Novel 4-Phenyl-2-Phenoxyacetamide Thiazoles modulates the tumor hypoxia leading to the crackdown of neoangiogenesis and evoking the cell death. *Eur. J. Med. Chem.*, **143**, 1826-1839 (2018).
14. Palatinus, L. Chapuis, G. SUPERFLIP - a computer program for the solution of crystal structures by charge flipping in arbitrary dimensions. *J. Appl. Cryst*. **40**, 786-790 (2007)
15. Parois, P., Cooper, R. I. Thompson, A. L. Crystal structures of increasingly large molecules: meeting the challenges with CRYSTALS software. *Chem. Cent. J*. **9** (30), 1-14 (2015)
16. Cooper, R. I, A. L. Thompson, A. L, Watkin, D. J., CRYSTALS Enhancements: Dealing with Hydrogen Atoms in Refinement, *J. Appl. Cryst*. **43**, 1100-1107 (2010)

17. Schmittgen, T. D., Livak, K. J., Analysis of relative gene expression data using real-time quantitative PCR and the 2(-Delta Delta C(T)) Method. *Methods*, **25**, 402-408 (2001)
18. Neese, F. Software update: The ORCA program system-Version 5.0. *WIREs Comput. Mol. Sci.,* **12**, e1606 (2022)
19. Grimme, S., Ehrlich, S. & Goerigk, L. Effect of the damping function in dispersion corrected density functional theory. *J. Comp. Chem.* **32**, 1456-1465 (2011).
20. Grimme, S., Antony, J., Ehrlich, S. & Krieg, H. A consistent and accurate ab initio parametrization of density functional dispersion correction (DFT-D) for the 94 elements H-Pu. *J. Chem. Phys.* **132**, 154104-1-19 (2010).
21. Weigend, F. & Ahlrichs, R. Balanced basis sets of split valence, triple zeta valence and quadruple zeta valence quality for H to Rn: Design and assessment of accuracy. *Phys. Chem. Phys.* **7**, 3297-3305 (2005).
22. Hellweg, A., Hättig, C., Höfener, S. & Klopper, W. Optimized accurate auxiliary basis sets for RI-MP2 and RI-CC2 calculations for the atoms Rb to Rn. *Theor. Chem. Acc.* **117**, 587-597 (2007)
23. Weigend, F. Accurate Coulomb-fitting basis sets for H to Rn. *Phys. Chem. Chem. Phys.* **8**, 1057 (2006)
24. Pracht, P., Bohle, F. & Grimme, S. Automated exploration of the low-energy chemical space with fast quantum chemical methods. *Phys. Chem. Chem. Phys.* **22**, 7169-7192 (2020).
25. Bannwarth, C., Ehlert, S. & Grimme, S. GFN2-xTB—An Accurate and Broadly Parametrized Self-Consistent Tight-Binding Quantum Chemical Method with Multipole Electrostatics and Density-Dependent Dispersion Contributions. *J. Chem. Theory Comput.* **15**, 1652-1671 (2019).
26. Barone, V. & Cossi, M. Quantum Calculation of Molecular Energies and Energy Gradients in Solution by a Conductor Solvent Model. *J. Phys. Chem. A,* **102**, 1995-2001 (1998).
27. Riplinger, C. & Neese, F. An efficient and near linear scaling pair natural orbital based local coupled cluster method. *J. Chem. Phys.* **138**, 034106 (2013).
28. Young, T. duartegroup/otherm, 2020 https://github.com/duartegroup/otherm
29. Grimme, S. Supramolecular Binding Thermodynamics by Dispersion-Corrected Density Functional Theory. *Chem. Eur. J.* **18**, 9955-9964 (2012).
